# Highly efficient synthesis and stereoselective migration reactions of chiral five-membered aza-spiroindolenines: scope and mechanistic understanding†

**DOI:** 10.1039/c6sc00176a

**Published:** 2016-03-29

**Authors:** Qing-Feng Wu, Chao Zheng, Chun-Xiang Zhuo, Shu-Li You

**Affiliations:** a State Key Laboratory of Organometallic Chemistry, Shanghai Institute of Organic Chemistry, Chinese Academy of Sciences 345 Lingling Lu Shanghai 200032 China zhengchao@sioc.ac.cn slyou@sioc.ac.cn http://shuliyou.sioc.ac.cn/

## Abstract

An Ir-catalyzed asymmetric synthesis of five-membered aza-spiroindolenines is achieved. Based on the detailed investigation of the reaction patterns of the aryl iminium migration, a one-pot asymmetric allylic dearomatization/migration sequence from racemic indole derivatives is realized, affording enantioenriched Pictet–Spengler-type products bearing an additional allylic stereogenic center adjacent to the C3 position of the indole core.

## Abstract 2

Ir-catalyzed asymmetric allylic dearomatization affords five-membered aza-spiroindolenines, from which enantioenriched Pictet–Spengler-type products are obtained *via* a highly stereoselective migration.

## Graphical abstract

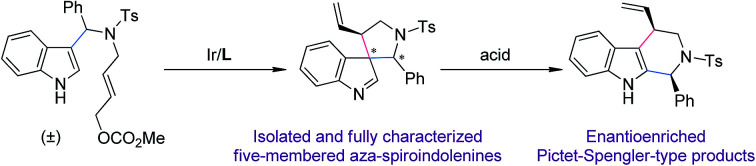

## Introduction

**Scheme 1:**
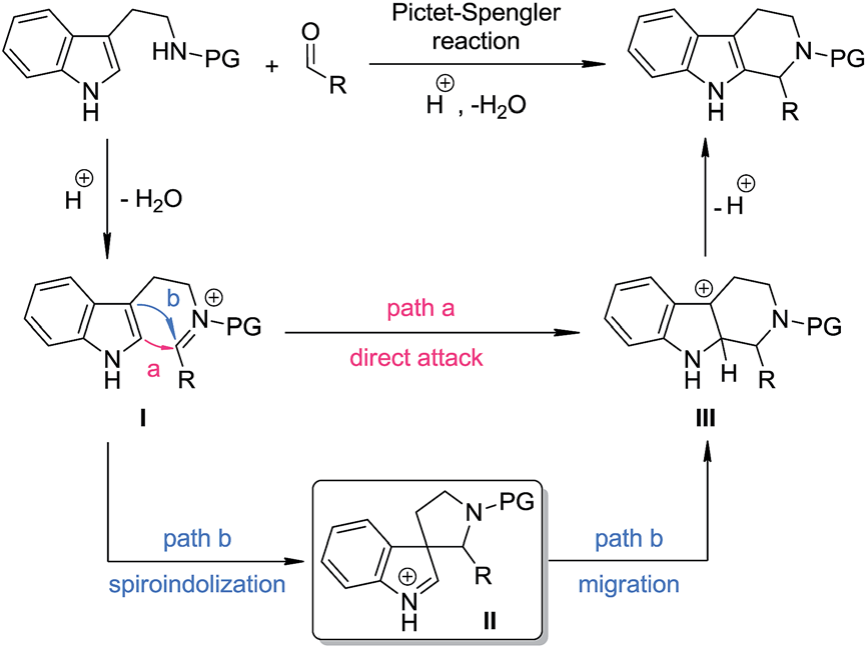
The possible pathways for Pictet–Spengler reactions.

The Pictet–Spengler reaction^1^ has long been recognized as one of the most powerful methods for the synthesis of tetrahydro-β-carbolines and other indole alkaloids with more complex structures.^2^ Despite their widespread use, mechanistically, Pictet–Spengler-type reactions still need further investigation.^1*c*^ In general, there are two possible pathways (Scheme 1): the reaction can occur through either the direct attack of the C2 position of indole towards the iminium moiety (path a) or the stepwise spiroindolization/migration sequence (path b). Although experimental evidence supporting the latter mechanism is documented a number of times in the literature,^3^ to the best of our knowledge, the five-membered aza-spiroindolenine, the key intermediate (II) for the asymmetric Pictet–Spengler reaction, has not been isolated yet.^4^

**Scheme 2:**
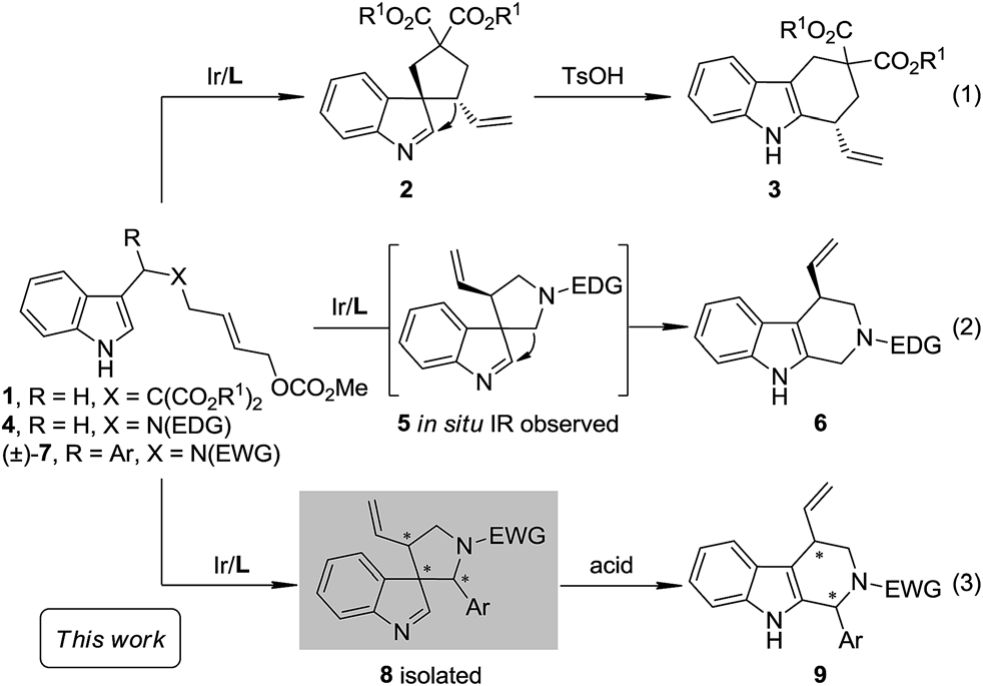
Ir-Catalyzed intramolecular asymmetric allylic dearomatization and subsequent migration.

As a part of our ongoing program of developing the catalytic asymmetric dearomatization (CADA) reaction,^5^ we reported in 2012 the enantioselective synthesis of carbocyclic spiroindolenines 2 with excellent enantioselectivity *via* Ir-catalyzed allylic dearomatization reactions of indole derivatives 1.^6–8^ Highly stereoselective allyl migration of 2 was achieved when treated with a catalytic amount of TsOH (eqn (1), Scheme 2).^9^ A mechanism involving a “three-center-two-electron” transition state is proposed according to DFT calculations and it is suggested that the electronic properties of the migratory group exert a great influence on the profile of the migration.^10^ Intriguingly, the reaction of the *N*-tethered substrate analogue 4 with an electron-donating group (EDG) attached to the nitrogen atom under Ir-catalysis yields the unexpected tetrahydro-β-carbolines 6, with the tether that was originally attached to the C3 position of indole moving to the C2 position. A dearomatized five-membered aza-spiroindolenine intermediate 5 was observed by *in situ* IR spectroscopy, but the attempts to isolate this intermediate were all unsuccessful (eqn (2), Scheme 2).^11–13^ Recently, by tuning the electronic properties of the *N*-linker, we achieved the synthesis of five-membered aza-spiroindolenines 8 with three contiguous stereogenic centers, from indole derivatives (±)-7 by Ir-catalysis. Upon isolation, 8 were exposed to acidic conditions, and the tetrahydro-β-carbolines 9 were delivered *via* aryl iminium migration (eqn (3), Scheme 2). Interestingly, the reaction pattern of the migration step highly depends on the relative stereochemistry of 8. Guided by further mechanistic investigations including DFT calculations, a one-pot asymmetric synthesis of Pictet–Spengler-type products 9, with two stereogenic centers, from (±)-7 was realized under enhanced reaction conditions. Herein, we report the full account of this study.

## Results and discussion

### Asymmetric synthesis of five-membered aza-spiroindolenines

Our study began by testing indol-3-yl phenyl allylic carbonates (±)-7 in Ir-catalyzed intramolecular allylic dearomatization reactions (Table 1).^14^ We envisioned that the addition of an electron-withdrawing group (EWG) to the *N*-linker might stabilize the desired five-membered aza-spiroindolenines 8 by reducing their activity toward the migration process. However, the existence of a benzylic substituent in 7 posed additional challenges for stereochemical control. Under the optimal conditions, three diastereoisomers of 8 were obtained in relatively good yields and excellent enantiomeric excess (ee). The structures and absolute configurations [(3*S*,8*R*,10*R*) for 8aA; (3*R*,8*S*,10*R*) for 8aB and (3*S*,8*S*,10*R*) for 8aC] were determined unambiguously by X-ray crystallographic analysis.^16^ When the protecting group on the nitrogen atom was Ts, the reactions of substrates bearing either an EDG (5-OMe, 7b and 6-Me, 7c) or an EWG (5-Br, 7d) on the indole core all led to their corresponding spiroindolenine products in excellent yields and ee. Substrates bearing various aryl groups (Ar) on the tether (7e–k) were well tolerated but showed very different activities. *p*-Tol substituted 7e gave the best diastereomeric ratio (dr) value of all of the N–Ts linked substrates tested. Notably, when an *o*-ClC_6_H_4_ substituted substrate 7j was used, the two diastereoisomers 8jA and 8jC could not be separated by column chromatography, giving a good combined isolated yield with an excellent ee. On the other hand, the reaction proceeded smoothly for the substrates containing different *N*-protecting groups (7l–q), affording the five-membered aza-spiroindolenine products in good yields and ee with a moderate to excellent dr. Among them, the *N*-Boc protected substrates bearing either an EDG (7o and 7p) or an EWG (7q) at various positions all gave a good dr and excellent ee.

**Table 1:** Scope of the Ir-catalyzed allylic dearomatization reaction^a^

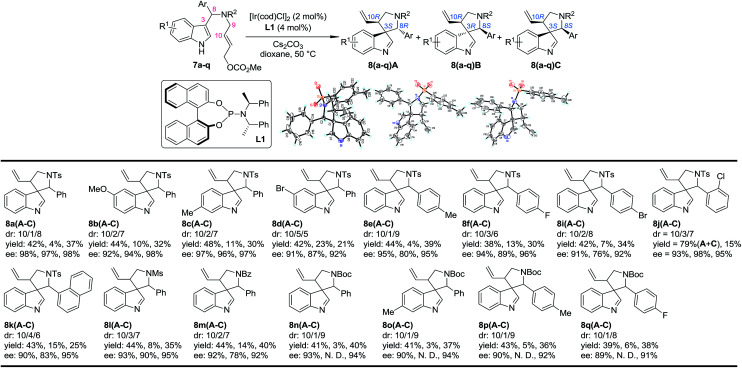

a Reaction conditions: 0.004 mmol of [Ir(cod)Cl]_2_, 0.008 mmol of L1 and 0.2 mmol of 7, 0.4 mmol of Cs_2_CO_3_ in dioxane (2.0 mL) at 50 °C. The catalyst was prepared *via*^*n*^PrNH_2_ activation.^15^ The dr values are determined by ^1^H NMR of the crude reaction mixture. The ee values are determined by HPLC analysis. The isolated yields are reported. N. D.: not determined.

### On the origin of the three diastereomers of the five-membered aza-spiroindolenines

**Scheme 3:**
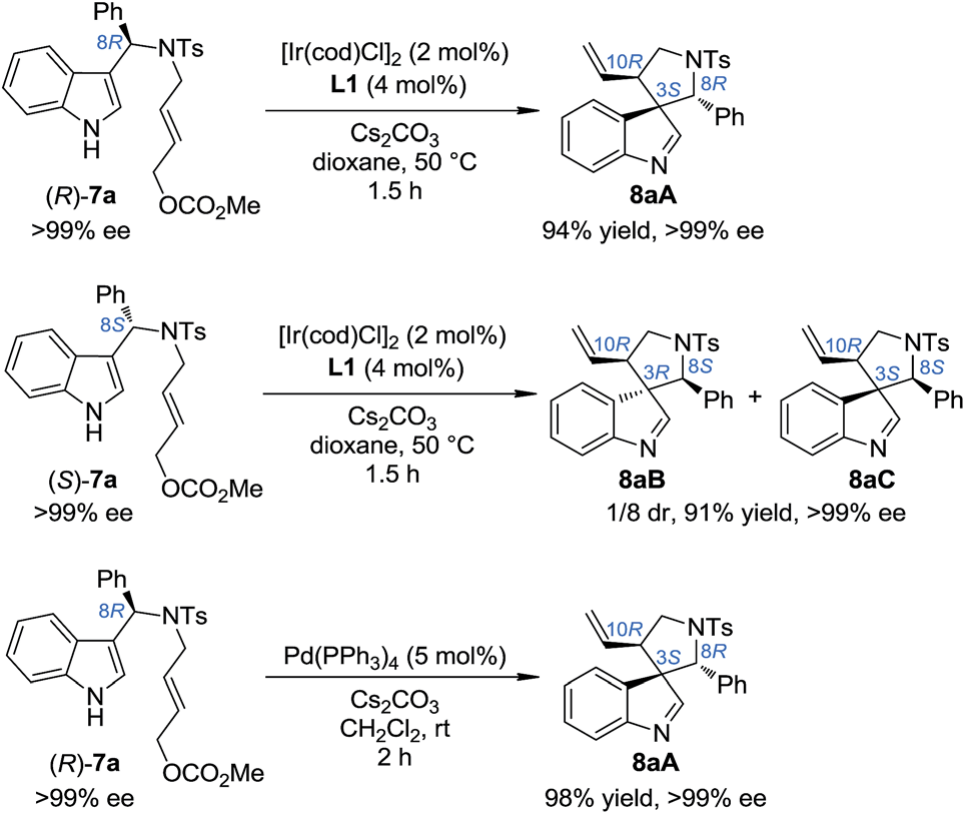
The reaction patterns of enantiopure substrates.

In order to gain deep insights into the origin of the three diastereoisomers of 8 from (±)-7, the enantiopure substrates (*R*)-7a and (*S*)-7a obtained by preparative HPLC were subjected to the standard dearomatization reaction conditions (Scheme 3). (*R*)-7a was converted to 8aA in a 94% yield and >99% ee. On the other hand, (*S*)-7a was transformed to 8aB and 8aC in a 1/8 dr and >99% ee. The ratio of the three diastereoisomers of 8a(A–C) and their stereochemistry are in agreement with the reaction results for (±)-7a (Table 1). In addition, 8aA could also be obtained from (*R*)-7a in 98% yield with >99% ee in 2 h, with Pd(PPh_3_)_4_ as an achiral catalyst.

### Stereoselective aryl iminium migration

**Scheme 4:**
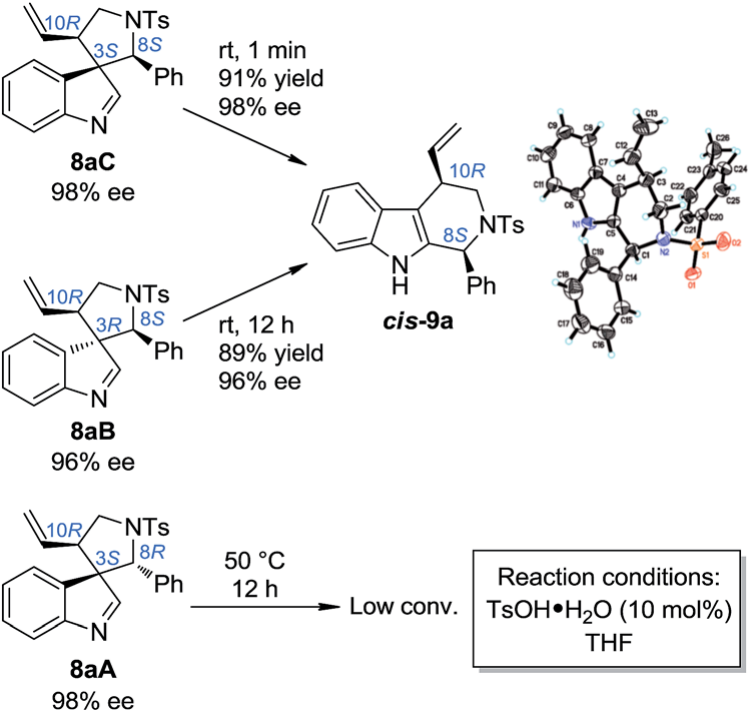
Regio- and stereoselective migration of the five-membered aza-spiroindolenines.

With the optimized method for the construction of the highly enantioenriched aryl substituted five-membered aza-spiroindolenines in hand, we next sought to identify conditions that are suitable for the subsequent migration process. It was quite interesting to find that the three diastereoisomers of 8a exhibit extremely different reactivities when exposed to our previously established migration conditions (10 mol% TsOH·H_2_O in THF at room temperature) (Scheme 4).^9^8aC was easily converted to tetrahydro-β-carboline *cis*-9a in a satisfactory yield and preserved ee within 1 minute. X-Ray crystallographic analysis of *cis*-9a showed that the aryl iminium moiety migrated to the C2 position of indole and the stereochemistry (8*S*,10*R*) of the benzylic position can be maintained, which is consistent with our understanding of the migration mechanism.^10^ The minor diastereoisomer 8aB also underwent similar regio- and stereoselective migration to afford *cis*-9a smoothly, albeit with a longer reaction time (12 h) at room temperature. However, the other major diastereoisomer 8aA remained intact even at an elevated temperature (50 °C).^17^

To test the generality of the regio- and stereoselective N–Ts aryl iminium migration reactions, the most reactive diastereoisomer 8C possessing different substituents was subjected to the above reaction conditions (Table 2). In all cases, the absolute configuration at the benzylic positions and the ee values of the compounds were well maintained, leading to the corresponding tetrahydro-β-carbolines *cis*-9 smoothly, in a highly enantioenriched form.

**Table 2:** Scope of the regio- and stereoselective migration^a^

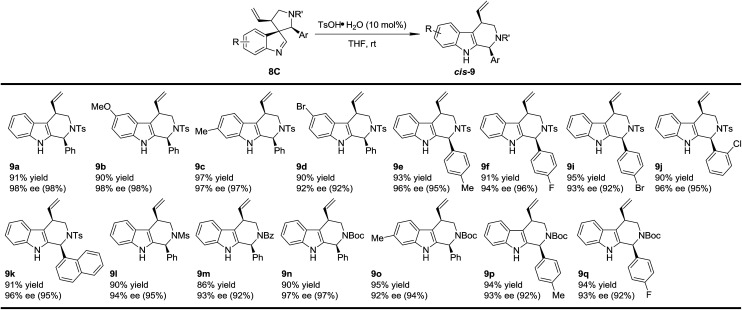

a Reaction conditions: 0.1 mmol of 8, 0.01 mmol of TsOH·H_2_O in THF (2 mL) at rt. The ee values are determined by HPLC analysis. The numbers in the parentheses are the ee values of the starting materials 8C. The isolated yields are reported.

### DFT calculations

**Fig. 1:**
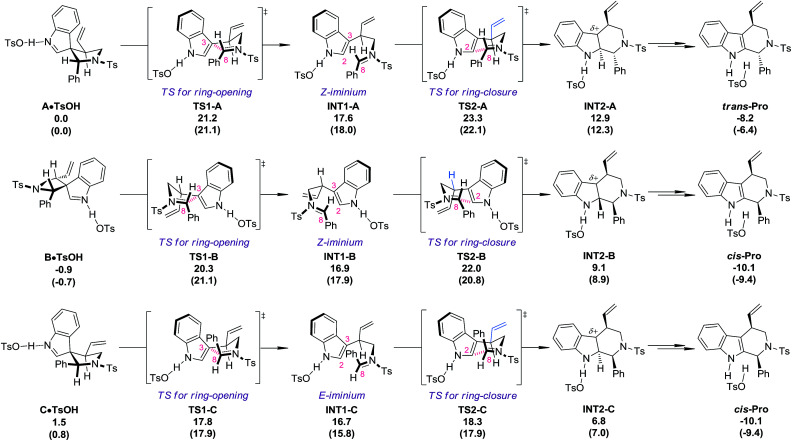
The reaction profiles for the TsOH catalyzed migration processes of the diastereoisomers of 8. Calculated at a PBE1PBE/6-311+G(d,p) level of theory. Δ*G*_THF_ and (Δ*E*_THF_) values are in kcal mol^−1^. The transition states of the deprotonation steps are not shown.

DFT calculations^18^ (PBE1PBE/6-311+G(d,p) level of theory) were carried out at this stage to investigate why the diastereoisomers of 8 exhibit dramatically different reactivities toward the migration process (Fig. 1). The complexes of the three diastereoisomers (8aA, 8aB and 8aC) with TsOH, named A·TsOH (0.0 kcal mol^−1^), B·TsOH (−0.9 kcal mol^−1^) and C·TsOH (1.5 kcal mol^−1^), respectively, were set as the starting points for their corresponding migration processes. All of the aryl N–Ts iminium migrations were characterized to be stepwise.^19^ The C·TsOH complex was the most prone to the reaction. The C3–C8 bond was cleaved *via* the ring-opening transition state TS1-C (17.8 kcal mol^−1^), leading to the key intermediate INT1-C (16.7 kcal mol^−1^) with an *E*-iminium moiety formed. Natural bond orbital (NBO) analysis of this intermediate revealed the existence of interactions between the indole ring and the N–Ts iminium part. According to the second-order perturbation theory analysis, the stabilization energy *E*(2) from the occupied π(C2–C3) orbital to the unoccupied π*(N–C8) orbital is estimated to be 5.69 kcal mol^−1^. It is speculated that this kind of interaction might account for the highly stereoselective migration phenomenon. Subsequently, the iminium carbon C8 is attacked by the indole C2 position *via* a chair-like six-membered-ring transition state TS2-C (18.3 kcal mol^−1^) to afford the protonated tetrahydro-β-carboline complex INT2-C (6.8 kcal mol^−1^). After the following deprotonation step (not shown in Fig. 1), the final complex *cis*-Pro (−10.1 kcal mol^−1^) is generated. For the second reactive species B·TsOH, a similar reaction profile was found, except a *Z*-iminium intermediate INT1-B (16.9 kcal mol^−1^) is involved, which makes the ring-closure (TS2-B, 22.0 kcal mol^−1^), the rate-limiting step, slightly higher in energy. If an *E*-iminium intermediate was forced to be generated from B·TsOH, the ring-closure step must proceed through an unfavorable boat-like six-membered-ring transition state which further raises the energetic barrier. The migration process of complex A·TsOH shares something in common with that of B·TsOH because an unfavorable *Z*-iminium intermediate (INT1-A, 17.6 kcal mol^−1^) is also generated. An additional detrimental factor for the migration process of A·TsOH was also identified in the key ring-closure transition state TS2-A (23.3 kcal mol^−1^). The vinyl moiety on the tether points toward the indole ring, which will cause a stronger steric repulsion compared with that observed in TS2-B where a hydrogen atom instead of the vinyl group is found in that place (highlighted in blue). As a result, TS2-A ranks as the most high-energy one among the three ring-closure transition states. In general, the order of reactivity toward the TsOH-catalyzed migration of the three diastereoisomers is qualitatively reproduced by computations. Besides, the calculated energetic barriers for the allyl migration are always higher than those of the aryl N–Ts iminium migration, which is also consistent with experimental observations.

### One-pot asymmetric allylic dearomatization/migration procedure

**Scheme 5:**
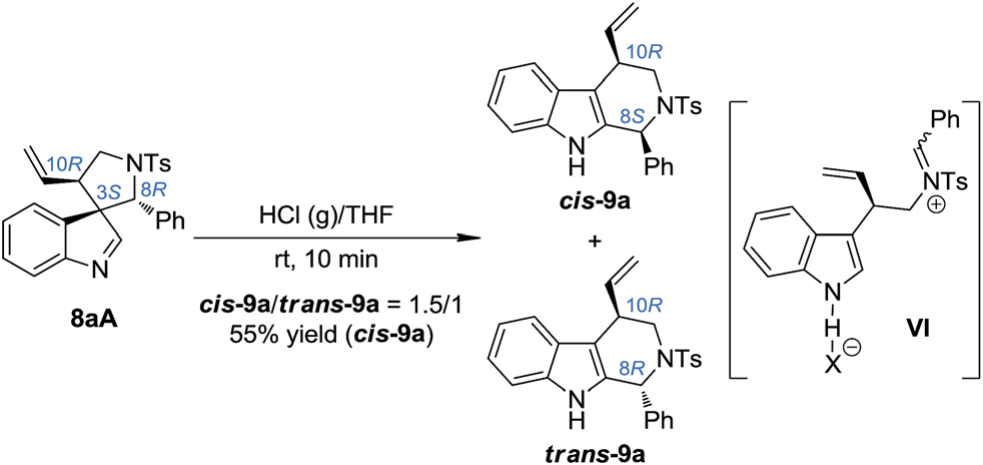
Migration reaction for the diastereoisomer 8aA.

**Scheme 6:**
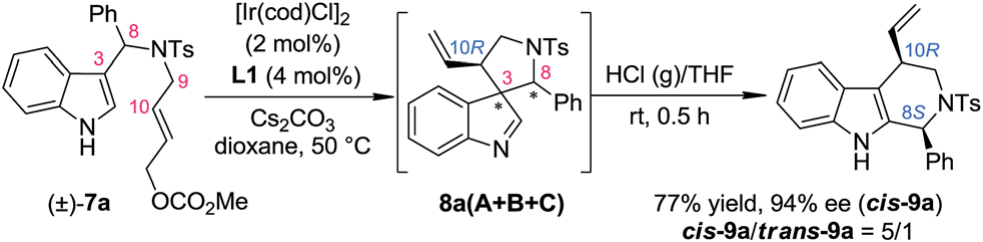
Optimized reaction conditions for the one-pot synthesis of tetrahydro-β-carbolines.

Based on the above mechanistic understanding, we decided to develop a one-pot highly stereoselective procedure to synthesize tetrahydro-β-carbolines 9 from (±)-7, which requires the full conversion of all of the three diastereoisomers of the intermediate 8. However, the unreactive 8A is the major challenge. Since the absolute configuration of the benzylic stereogenic center (C8) of 8A is the opposite of that of 8B and 8C, even if 8A could take part in the migration reaction, and at the same time all of the chiral information at the benzylic position was preserved, only a mixture of *cis*-9 and *trans*-9 with a 1 : 1 ratio could be obtained [the ratio of 8A : (8B + 8C) is about 1 : 1]. We speculated that using a stronger acid as the catalyst might be a possible solution to this issue. The reasons for this choice include: (1) the spiroindolenines can be better activated by stronger acids and thus the ring-opening step can be better facilitated; (2) stronger acids might lead to a more loosely bound ion-pair in INT-1, which in turn might weaken or even destroy the interaction between the indole ring and the aryl iminium moiety, thus reducing the possibility of the maintenance of the chiral information possessed by the migratory group. To our delight, we discovered that freshly prepared saturated HCl (g)/THF was an efficient acid system to promote the migration of 8aA, providing two diastereoisomers of the tetrahydro-β-carbolines *cis*-9a and *trans*-9a in a 1.5/1 dr (Scheme 5).^14^ Intriguingly, the major diastereoisomer obtained (*cis*-9a) was the same as that from 8aB or 8aC (8*S*,10*R*). This means that the absolute configuration of the benzylic stereogenic center of 8aA can indeed be inversed during the migration process in a more acidic medium, and a free iminium species (IV) might be formed during the migration. The diastereoselectivity of the migration step might be induced by the allylic stereogenic center adjacent to the C3 position of the indole core. Encouraged by these findings, we further identified enhanced reaction conditions for the one-pot synthesis of tetrahydro-β-carbolines 9 from (±)-7*via* a dearomatization/migration sequence.^14^ Under the optimal conditions, the desired product *cis*-9a was obtained in a 77% yield and good dr (*cis*-9a/*trans*-9a = 5/1) (Scheme 6).

With this newly established one-pot asymmetric allylic dearomatization/migration procedure, various substrates were tested to examine the generality of the reaction (Table 3). Reactions of substrates bearing either an EDG (5-OMe, 7b and 6-Me, 7c) or an EWG (5-Br, 7d) on the indole core all led to their corresponding Pictet–Spengler-type products in moderate dr and yields with excellent ee (3.8/1–4/1 dr, 62–70% yield, 92–94% ee). Substrates bearing aryl groups with various substituted patterns (*p*-F, 9f; *p*-Cl, 9g; *m*-Br, 9h; *p*-Br, 9i and *o*-Cl, 9j) on the tether underwent the dearomatization/migration reaction smoothly to give the tetrahydro-β-carbolines with satisfactory results (5/1–6/1 dr, 64–75% yields, 93–95% ee). The different *N*-protecting groups such as Ms and Boc (9l and 9n) were also tolerated, affording the corresponding products in good yields and enantioselectivity (92–93% ee), albeit with a decreased dr.

**Table 3:** Scope of the one-pot asymmetric allylic dearomatization/migration procedure^a^

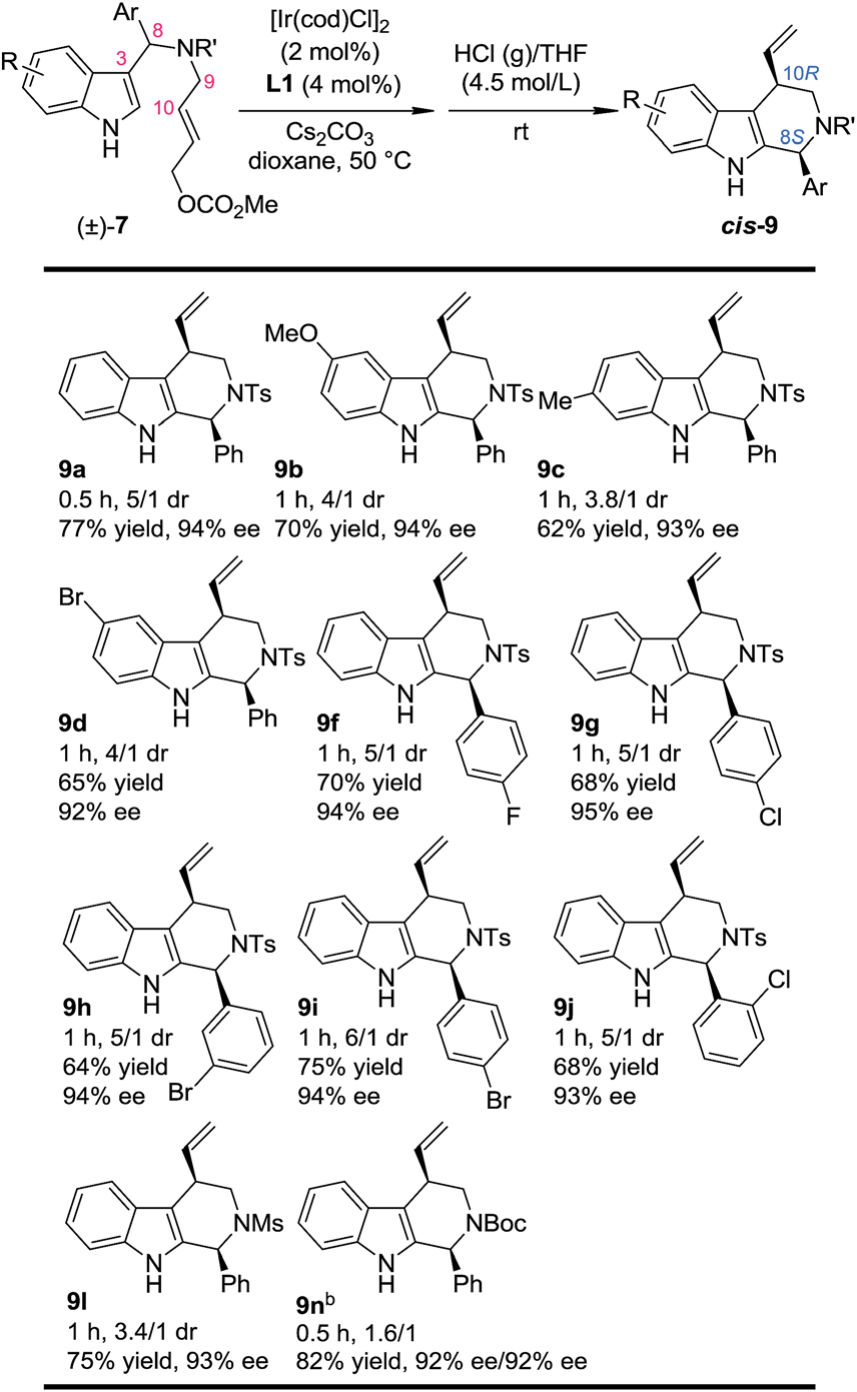

a Reaction conditions: for step 1: the same as shown in Table 1. For step 2: the reaction residue of step 1 containing intermediate 8 in HCl (g)/THF (4.0 mL, 4.5 mol L^−1^) at rt. The dr values are determined by ^1^H NMR of the crude reaction mixture. The ee values are determined by HPLC analysis. The isolated yields are reported.

b Combined yield of the two diastereoisomers.

## Conclusions

In summary, we have developed highly efficient synthesis and stereoselective migration reactions of enantioenriched five-membered aza-spiroindolenines on the basis of a deep understanding of the reaction mechanism and the rational design of the substrates. Ir-catalyzed intramolecular allylic dearomatization of the racemic indol-3-yl aryl substituted allylic carbonates led to the highly enantioenriched five-membered aza-spiroindolenines which are isolated and fully characterized for the first time. A detailed investigation of the reaction patterns of the three diastereoisomers paved the way to a highly regio- and stereoselective migration whose mechanism was further understood by DFT calculations. In addition, a one-pot asymmetric allylic dearomatization/migration sequence was developed under enhanced reaction conditions, providing a novel strategy to synthesize the Pictet–Spengler-type products with an additional allylic stereogenic center adjacent to the C3 position of indole.

## Supplementary Material

SC-007-C6SC00176A-s001

SC-007-C6SC00176A-s002
