# Supplementary material for: Highly efficient synthesis and stereoselective migration reactions of chiral five-membered aza-spiroindolenines: scope and mechanistic understanding
Source: Chem Sci. 2016 Mar 29;7(7):4453–9. doi: 10.1039/c6sc00176a (PMC6014299; doi:10.1039/c6sc00176a)
Supplement: SC-007-C6SC00176A-s001 [file SC-007-C6SC00176A-s001.pdf]

Electronic Supplementary Material (ESI) for Chemical Science.
This journal is © The Royal Society of Chemistry 2016

Electronic Supplementary Information for

Highly Efficient Syntheses and Stereoselective Migration Reactions of Chiral Five-Membered Aza-Spiroindolenines: Scope and Mechanistic Understanding

Qing-Feng Wu, Chao Zheng,* Chun-Xiang Zhuo and Shu-Li You*

State Key Laboratory of Organometallic Chemistry, Shanghai Institute of Organic Chemistry, Chinese Academy of Sciences, 345 Lingling Lu, Shanghai 200032, China.

E-mail: zhengchao@sioc.ac.cn (C.Z.) or slyou@sioc.ac.cn (S.-L.Y.)

Table of Contents

Experimental part

Tables for the optimization of reaction conditions……S2

General methods……S9

Experimental details and characterization data……S10

X-ray data and structure refinement……S52

NMR Spectra and HPLC traces……S64

References……S197

Computational part

Computational methods……S198

Calculated reaction profiles of the aryl N–Ts iminium migration processes of the isomers **8aA-C**……S199

NBO analyses of **INT1-A**, **INT1-B** and **INT1-C**……S201

3D structures of the calculated stationary points……S212

Cartesian coordinates of the calculated stationary points……S214

References……S250

Tables for the optimization of reaction conditions

Our studies began by testing racemic substrate **7a** with an Ir-catalytic system derived from $[Ir(cod)Cl]_2$ and various chiral phosphoramidite ligands[1] (Table S1). In the presence of 2 mol% of $[Ir(cod)Cl]_2$, 4 mol % of **L1**, and 2.0 equiv. of Cs_2CO_3, the reaction of **7a** in THF for 1.5 h gave the five-membered aza-spiroindolenine products **8a** in 10/1/8 dr and relatively good yields as well as excellent ees (**8aA**: 39% yield, 98% ee; **8aB**: 5% yield, 96% ee; **8aC**: 32% yield, 98% ee; entry 1). To be noted, the three diastereoisomers could be separated by silica gel column chromatography. The structures of these three diastereoisomers and their stereochemistry [(3*S*,8*R*,10*R*) for **8aA**; (3*R*,8*S*,10*R*) for **8aB**; (3*S*,8*S*,10*R*) for **8aC**] were determined unambiguously by the single crystal X-ray crystallographic analyses. Notably, the absolute configurations at the allylic position (C10) of the three diastereoisomers are the same. Screening the chiral phosphoramidite ligands (entries 1-8) led to the identification of Feringa ligand **L1** as the best one in terms of dr and ee values (entry 1). The 2-MeO substituted phosphoramidite ligand **L2** introduced by Alexakis and co-workers[2] could catalyze the reaction in decreased dr although with excellent enantioselectivity (entry 2). The 2-naphthyl substituted phosphoramidite ligand **L3** could give the products in similar dr and yield but slightly decreased ee (entry 3). Relatively poor enantioselective control when **L4** was used indicated that (S,S,S_a) is the matched chirality of ligands for this reaction (entry 4). Me-THQPhos **L5**[3] only gave the products in moderate selectivity (entry 5). Systematical evaluation of solvents and bases disclosed that the reaction in dioxane with Cs_2CO_3 gave the optimal results (10/1/8 dr; **8aA**: 42% yield, 98% ee; **8aB**: 4% yield, 97% ee; **8aC**: 37% yield, 98% ee; entry 18).

Table S1. Optimization of the Reaction Conditions for Ir-catalyzed Allylic Dearomatization Reaction.[a]

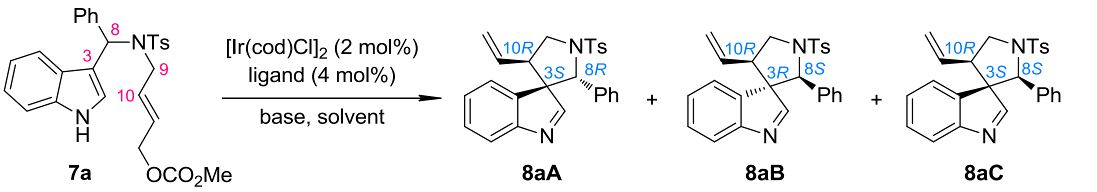

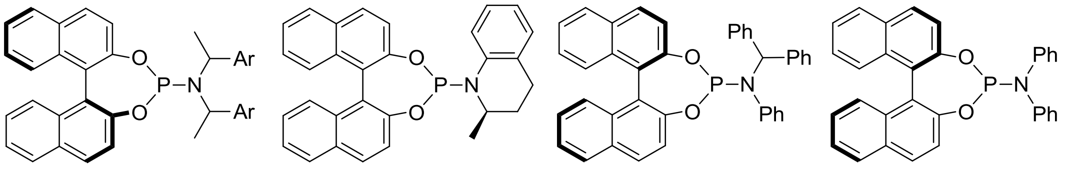

entry	ligand	solvent	base	temp (°C)	t (h)	*dr*[b] (**8aA/8aB/8aC**)	yield (%)[c]	*ee* (%)[d]
1	**L1**	THF	Cs_2CO_3	50	1.5	10/1/8	76	98/98/98
2	**L2**	THF	Cs_2CO_3	50	1.5	10/3/6	76	98/97/97
3	**L3**	THF	Cs_2CO_3	50	1.5	10/1/8	76	97/95/98
4	**L4**	THF	Cs_2CO_3	50	12	10/2/6	96	92/92/91
5	**L5**	THF	Cs_2CO_3	50	1.5	10/4/6	71	91/92/82
6	**L6**	THF	Cs_2CO_3	50	12	N. D.[e]	<5[f]	N. D.
7	**L7**	THF	Cs_2CO_3	50	12	10/5/2	70	95/95/88
8	**L8**	THF	Cs_2CO_3	50	12	N. D.	<5[f]	N. D.
9	**L1**	THF	K_3PO_4	50	3	10/2/6	83	98/97/99
10	**L1**	THF	KOAc	50	3	10/1/7	92	98/91/98
11	**L1**	THF	DABCO	50	3	10/5/4	83	97/95/98
12	**L1**	THF	DMAP	50	3	10/3/5	76	98/97/98
13	**L1**	THF	Et_3N	50	3	10/2/6	87	98/89/99/
14	**L1**	THF	DBU	50	3	10/2/7	70	98/99/99
15	**L1**	THF	DIEA	50	3	10/1/7	74	98/81/99
16	**L1**	THF	BSA	50	3	10/2/7	71	97/87/98
17	**L1**	DCM	Cs_2CO_3	reflux	3	10/5/4	87	85/80/84

18	**L1**	dioxane	Cs_2CO_3	50	1.5	10/1/8	83	98/97/98
19	**L1**	Et_2O	Cs_2CO_3	reflux	3	10/2/6	71	98/97/99

[a] Reaction conditions: 0.004 mmol of [Ir(cod)Cl]$_2$, 0.008 mmol of ligand, 0.2 mmol of **7a**, 0.4 mmol of base in solvent (2.0 mL). The catalyst was prepared *via* ^{n}PrNH$_2$ activation.[4] [b] Determined by ^{1}H NMR of the crude reaction mixture. [c] Isolated combined yield of the three diastereoisomers. [d] Determined by HPLC analysis. [e] N. D.: Not determined. [f] Conversion was determined by ^{1}H NMR.

After successfully realized highly efficient syntheses of the enantioenriched five-membered aza-spiroindolenines **8** and stereoselective migration of one of their diastereoisomers **8C**. We tried to develop a method for the direct conversion of all the three diastereoisomers of **8** to tetrahydro-*β*-carboline product. We suspected that if a stronger acid was used, the interaction between the indole ring and the aryl iminium moiety could be probably weakened or even destroyed, which might reduce the possibility of maintenance of the chiral information possessed by the migratory group. Bearing this idea in mind, we examined the reaction pattern of **8aA** under more acidic conditions (Table S2).

At the outset, we changed the solvent to non-polar CH_2Cl_2 using TsOH•H_2O as the acid. The reaction did not occur in 4 h under reflux condition (entry 2). When TFA was used, the desired product was observed at first then disappeared soon to give a messy reaction mixture (entry 3). Product **9a** might be unstable under strong acidic condition, which was proven by the decomposition of **9a** in TFA at room temperature. Benzoic acid and 4-nitrobenzoic acid were found to be not suitable for the reaction, leaving the starting material recovered (entries 4-5). A mixture of 38 wt% HCl (aq.) with THF in 1/2 ratio gave a moderate conversion in 5 h, but prolonged reaction led to decomposition of the product (entry 6). We were pleased to find that freshly prepared saturated HCl (g)/THF was an efficient acid system to promote the migration of 8aA, providing two diastereoisomers of the tetrahydro-*β*-carbolines *cis*-**9a** and *trans*-**9a** in 1.5/1 dr (entry 7). The absolute configuration of *trans*-**9a** was established to be (8*R*,10*R*).[5]

Table S2. Optimization of the Reaction Conditions for Migration of **8aA**.[a]

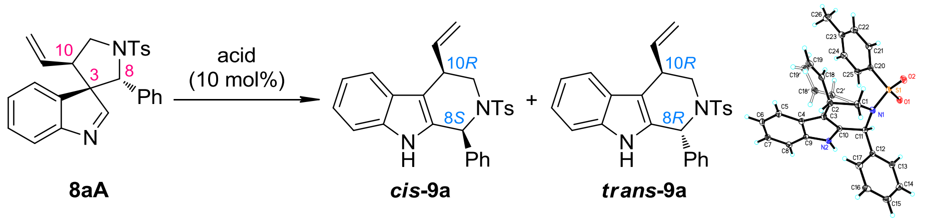

entry	acid	solvent	temp (℃)	t	*dr*[b]	conv. (%)[c]	yield (%)[d]
1	TsOH•H_2O	THF	50	12 h	N. D.[e]	<5	N. D.
2	TsOH•H_2O	CH_2Cl_2	reflux	4 h	N. D.	<5	N. D.
3	TFA[f]	none	rt	1 h	N. D.	100	mix

4	benzoic acid	CH_2Cl_2	reflux	3 h	N. D.	<5	N. D.
5	4-NBA[g]	CH_2Cl_2	reflux	3 h	N. D.	<5	N. D.
6	HCl (aq.)[h]	THF	50	5 h	N. D.	60	mix
7	HCl (g)/THF[i]	/	rt	10 min	1.5/1	N. D.	55

[a] Reaction conditions: 0.2 mmol of **8aA**, 0.02 mmol of acid, in solvent (2.0 mL). [b] Determined by ^{1}H NMR of the crude reaction mixture. [c] Determined by ^{1}H NMR. [d] Isolated yield of the major diastereoisomer. [e] N. D.: not determined. [f] TFA (2.0 mL). [g] 4-NBA = 4-nitrobenzoic acid. [h] 38 wt% HCl (aq.)/THF = 1/2, 2.0 mL. [i] Saturated HCl (g) in THF, c = 4.5 mol/mL.

Further screening of the reaction conditions for the one-pot dearomatization/migration sequence was carried out to search for the optimal results (Table S3). Freshly prepared saturated HCl (g) in various solvents were investigated to promote the migration process after removal of Cs_2CO_3 by celite upon the completion of the dearomatization reaction. Reaction using HCl (g)/THF as the acid finished in 0.5 h, giving the desired products *cis*-**9a** and *trans*-**9a** in 5/1 dr and 77% yield of the major diastereoisomer with excellent ee (entry 1). Reducing the reaction temperature to 0 ℃ led to a long reaction time and low yield because of the product decomposition (entry 2). When HCl (g)/Et_2O was used, reaction at room temperature gave the product in a decreased yield (60%, entry 3). Substrate **8a** has poor solubility in Et_2O at low temperature, therefore a low conversion was resulted in Et_2O at 0 ℃ (entry 4). Other acid systems screened did not efficiently promote the migration reaction (entries 5-7).

Table S3. Optimization of the Reaction Conditions for One-pot Asymmetric Allylic Dearomatization/Migration Procedure.[a]

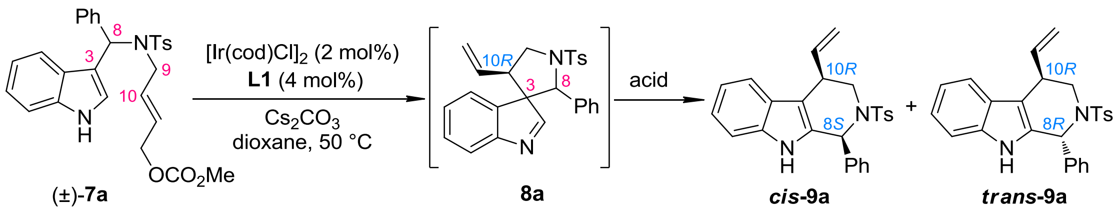

entry	acid[b]	temp (°C)	t (h)	dr[c]	conv. (%)[d]	yield (%)[e]	ee (%)[f]
1	HCl (g)/THF	rt	0.5	5/1	100	77	94
2	HCl (g)/THF	0	2	5/1	100	65	94
3	HCl (g)/Et_2O	rt	0.5	6.7/1	100	60	93
4	HCl (g)/Et_2O	0	2	7/1	70	N. D.[g]	N. D.
5	HCl (g)/dioxane	rt	2	17/1	63	N. D.	N. D.
6	HCl (g)/CH_2Cl_2	rt	2	24/1	50	N. D.	N. D.
7	HCl (g)/toluene	rt	2	15/1	50	N. D.	N. D.

[a] Reaction conditions: For step 1: 0.004 mmol of $[Ir(cod)Cl]_2$, 0.008 mmol of **L1**, 0.2 mmol of **7**, 0.4 mmol of Cs_2CO_3 in dioxane (2.0 mL) at 50 ℃. The catalyst was prepared via $^n PrNH_2$ activation.[4] For step 2: the dearomatized intermediate **8** (obtained in step 1) in solvent (4.0 mL) saturated with HCl (g). [b] Saturated HCl (g) in solvent. [c] Determined by ^{1}H NMR of the crude reaction mixture. [d] Determined

by ^{1}H NMR. [e] Isolated yield of the major diastereoisomer. [f] Determined by HPLC analysis. [g] N. D.: not determined.

General methods

Unless stated otherwise, all reactions were carried out in flame-dried glassware under a dry argon atmosphere. All solvents were purified and dried according to standard methods prior to use.

^{1}H and ^{13}C NMR spectra were recorded on a Varian instrument (400 MHz and 100 MHz, respectively) and internally referenced to tetramethylsilane signal or residual protio solvent signals. ^{19}F NMR spectra were recorded on Varian instrument (282 MHz and 376 MHz, respectively) and referenced relative to $CFCl_3$. Data for ^{1}H NMR are recorded as follows: chemical shift (δ, ppm), multiplicity (s = singlet, d = doublet, t = triplet, m = multiplet or unresolved, br = broad singlet, coupling constant(s) in Hz, integration). Data for ^{13}C NMR are reported in terms of chemical shift (δ, ppm).

The phosphoramidite ligands,[1a,6] the indolyl methanamines[7] and (*E*)-4-bromo-but-2-enyl methyl ester[8] were prepared according to the reported procedures.

Experimental details and characterization data

General Procedure for the Synthesis of the Substituted Allylic Carbonates

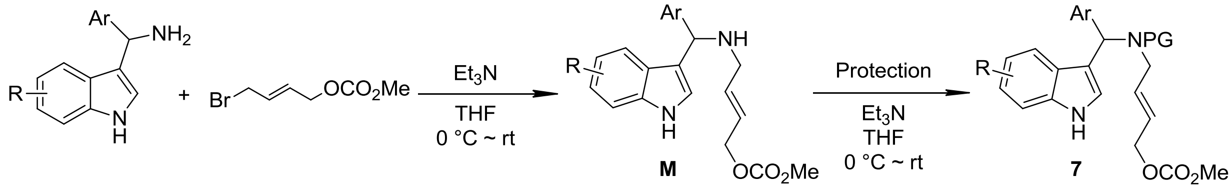

To a solution of the indolyl methanamine[7] (2 mmol, 1.0 equiv.) and Et_3N (0.46 mL, 3 mmol, 1.5 equiv.) in dry THF (50 mL), carbonic acid (*E*)-4-bromo-but-2-enyl methyl ester[8] (458 mg, 2.2 mmol, 1.1 equiv.) was added at 0 ℃. The ice bath was removed and the reaction mixture was stirred at room temperature for 24 h. After the reaction was complete (monitored by TLC), the crude reaction mixture was filtrated with celite and washed with EtOAc. The solvents were removed under reduced pressure. Then the residue was purified by silica gel column chromatography (PE/EA = 1/4) to afford the intermediate **M**.

To a solution of the intermediate **M** (1.0 mmol, 1.0 equiv.) and Et_3N (0.23 mL, 1.5 mmol, 1.5 equiv.) in dry THF (50 mL), the protecting group reagent [TsCl, $(Boc)_2O$, BzCl, and MsCl] (1.1 equiv.) was added at 0 ℃. The ice bath was removed and the reaction mixture was stirred at room temperature. After the reaction was complete (monitored by TLC), the crude reaction mixture was filtrated with celite and washed with EtOAc. The solvents were removed under reduced pressure. Then the residue was purified by silica gel column chromatography (PE/EA = 2/1) to afford the desired product **7**.

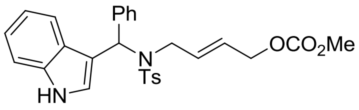

7a

Viscous yellow oil. ^{1}H NMR (300 MHz, $CDCl_3$) δ 2.33 (s, 3H), 3.67 (s, 3H), 3.89 (d, *J* = 6.0 Hz, 2H), 4.06-4.10 (m, 2H), 5.05 (dt, *J* = 15.3, 6.0 Hz, 1H), 5.21 (dt, *J* = 15.0, 6.0 Hz, 1H), 6.57 (d, *J* = 1.5 Hz,

1H), 6.63 (s, 1H), 6.91 (dd, *J* = 7.5, 7.2 Hz, 1H), 7.06-7.18 (m, 9H), 7.25 (d, *J* = 8.4 Hz, 1H), 7.57 (d, *J* = 8.1 Hz, 2H), 8.66 (br s, 1H); ^{13}C NMR (75 MHz, $CDCl_3$) δ 21.2, 46.6, 54.4, 58.6, 67.0, 111.2, 113.5, 119.1, 119.3, 121.9, 125.1, 125.2, 126.3, 127.1, 127.86, 127.92, 129.1, 131.8, 136.1, 137.3, 139.0, 142.9, 155.1; IR (thin film): ν_{max} (cm^{-1}) = 3389, 3029, 2956, 1748, 1598, 1448, 1267, 1158, 944, 747, 665, 545; LC-ESI-MS: 527 [M+Na]$^+$; HRMS (ESI) calcd for $C_{28}H_{28}N_2NaO_5S$ [M+Na]$^+$: 527.1611; Found: 527.1611.

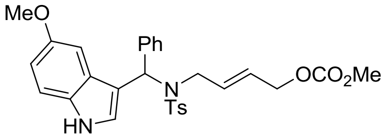

7b

Viscous yellow oil. ^{1}H NMR (300 MHz, $CDCl_3$) δ 2.34 (s, 3H), 3.66 (s, 3H), 3.70 (s, 3H), 3.90 (d, *J* = 6.0 Hz, 2H), 4.10-4.13 (m, 2H), 5.08 (dt, *J* = 15.6, 6.3 Hz, 1H), 5.26 (dt, *J* = 15.6, 6.3 Hz, 1H), 6.57 (d, *J* = 1.8 Hz, 1H), 6.61 (s, 1H), 6.69 (d, *J* = 1.8 Hz, 1H), 6.78 (dd, *J* = 8.7, 2.1 Hz, 1H), 7.11-7.21 (m, 8H), 7.58 (d, *J* = 8.7 Hz, 2H), 8.46 (br s, 1H); ^{13}C NMR (75 MHz, $CDCl_3$) δ 21.2, 46.8, 54.5, 55.5, 58.7, 67.1, 100.9, 111.9, 112.4, 113.5, 125.1, 125.9, 126.9, 127.1, 127.2, 127.9, 128.0, 129.1, 131.2, 131.9, 137.5, 139.1, 142.9, 153.8, 155.2; IR (thin film): ν_{max} (cm^{-1}) = 3396, 2931, 1747, 1625, 1586, 1486, 1442, 1329, 1261, 1211, 1155, 1089, 1050, 970, 792, 700, 661; HRMS (ESI) calcd for $C_{29}H_{34}N_3O_6S$ [M+NH_4]$^+$: 552.2163; Found: 552.2161.

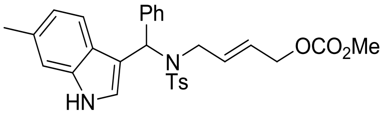

7c

Viscous yellow oil. ^{1}H NMR (300 MHz, $CDCl_3$) δ 2.33 (s, 3H), 2.35 (s, 3H), 3.67 (s, 3H), 3.88 (d, *J* = 5.7 Hz, 2H), 4.07-4.16 (m, 2H), 5.08 (dt, *J* = 15.3, 5.7 Hz, 1H), 5.22 (dt, *J* = 15.6, 5.7 Hz, 1H), 6.49 (s, 1H), 6.59 (s, 1H), 6.75 (d, *J* = 7.8 Hz, 1H), 6.98 (d, *J* = 8.4 Hz, 1H), 7.02 (s, 1H), 7.10-7.21 (m, 7H), 7.57 (d, *J* = 7.2 Hz, 2H), 8.52 (br s, 1H); ^{13}C NMR (75 MHz, $CDCl_3$) δ 21.1, 21.3, 46.6, 54.4, 58.7, 67.0, 111.1, 113.2, 118.8, 121.1, 124.1, 124.6, 125.1, 126.7, 127.0, 127.1, 127.9, 129.0, 131.6,

131.8, 136.5, 137.3, 142.8, 139.1, 155.1; IR (thin film): ν_{max} (cm^{-1}) = 3391, 2922, 1748, 1494, 1448, 1333, 1264, 1157, 1090, 944, 898, 701, 662; LC-ESI-MS: 541 $[M+Na]^+$; HRMS (ESI) calcd for $C_{29}H_{30}N_2NaO_5S$ $[M+Na]^+$: 541.1768; Found: 541.1761.

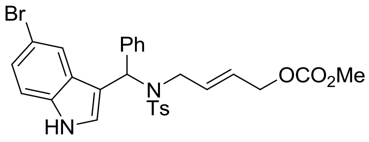

7d

Viscous yellow oil. ^{1}H NMR (400 MHz, $CDCl_3$) δ 2.40 (s, 3H), 3.72 (s, 3H), 3.91 (d, *J* = 6.0 Hz, 2H), 4.13-4.21 (m, 2H), 5.11 (dt, *J* = 15.6, 6.0 Hz, 1H), 5.23 (dt, *J* = 15.6, 6.4 Hz, 1H), 6.45 (s, 1H), 6.64 (d, *J* = 2.0 Hz, 1H), 7.05 (d, *J* = 1.2 Hz, 1H), 7.12-7.25 (m, 9H), 7.62 (d, *J* = 8.4 Hz, 2H), 8.55 (br s, 1H); ^{13}C NMR (100 MHz, $CDCl_3$) δ 21.5, 47.0, 54.7, 58.4, 67.2, 112.8, 113.0, 113.9, 121.6, 125.1, 125.6, 126.7, 127.2, 127.5, 128.0, 128.1, 128.2, 129.5, 131.8, 134.8, 137.3, 139.1, 143.4, 155.3; IR (thin film): ν_{max} (cm^{-1}) = 3373, 3028, 2923, 1748, 1598, 1450, 1333, 1266, 1157, 1266, 1089, 945, 757, 701; LC-ESI-MS: 605 $[M+Na]^+$; HRMS (ESI) calcd for $C_{28}H_{27}N_2NaO_5SBr$ $[M+Na]^+$: 605.0716; Found: 605.0714.

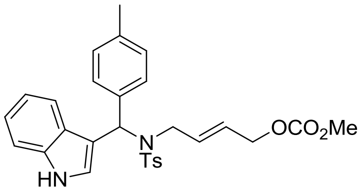

7e

Viscous yellow oil. ^{1}H NMR (400 MHz, $CDCl_3$) δ 2.31 (s, 3H), 2.38 (s, 3H), 3.72 (s, 3H), 3.90 (d, *J* = 6.0 Hz, 2H), 4.07-4.17 (m, 2H), 5.09 (dt, *J* = 15.6, 6.0 Hz, 1H), 5.22 (dt, *J* = 15.6, 6.4 Hz, 1H), 6.59 (s, 1H), 6.67 (d, *J* = 1.6 Hz, 1H), 6.95 (dd, *J* = 7.6, 7.2 Hz, 1H), 6.99-7.05 (m, 4H), 7.12-7.17 (m, 4H), 7.28 (dd, *J* = 7.6, 1.6 Hz, 1H), 7.60 (d, *J* = 8.4 Hz, 2H), 8.31 (br s, 1H); ^{13}C NMR (100 MHz, $CDCl_3$) δ 20.9, 21.4, 46.7, 54.6, 58.6, 67.3, 111.1, 114.3, 119.6, 122.1, 125.1, 125.2, 126.5, 127.4, 128.1, 128.8, 129.2, 129.6, 132.2, 136.0, 136.2, 137.0, 137.7, 142.9, 155.3; IR (thin film): ν_{max} (cm^{-1}) = 3388, 2924, 1747, 1598, 1513, 1333, 1264, 1157, 1090, 1013, 944, 815, 792, 745, 663; LC-ESI-MS: 541 $[M+Na]^+$;

HRMS (ESI) calcd for $C_{29}H_{30}N_2NaO_5S$ $[M+Na]^+$: 541.1768; Found: 541.1773.

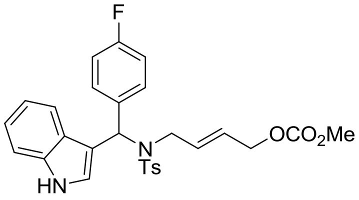

7f

Viscous yellow oil. ^{1}H NMR (300 MHz, $CDCl_3$) δ 2.40 (s, 3H), 3.73 (s, 3H), 3.91 (d, *J* = 6.0 Hz, 2H), 4.12-4.16 (m, 2H), 5.09 (dt, *J* = 15.6, 5.7 Hz, 1H), 5.24 (dt, *J* = 15.6, 6.3 Hz, 1H), 6.59-6.61 (m, 2H), 6.85-6.96 (m, 3H), 7.06-7.18 (m, 6H), 7.28 (d, *J* = 8.1 Hz, 1H), 7.59 (d, *J* = 8.1 Hz, 2H), 8.53 (br s, 1H); ^{13}C NMR (75 MHz, $CDCl_3$) δ 21.3, 46.7, 54.6, 58.1, 67.1, 111.3, 113.6, 114.9 (d, *J* = 21.4 Hz), 119.3, 119.6, 122.2, 125.1, 125.4, 126.2, 127.2, 129.3, 129.7 (d, *J* = 7.8 Hz), 131.8, 135.0, 136.2, 137.4, 143.2, 155.3, 161.9 (d, *J* = 244.8 Hz); IR (thin film): ν_{max} (cm^{-1}) = 3382, 2959, 2854, 1748, 1599, 1508, 1445, 1262, 1158, 1091, 1016, 794, 748, 663, 549; LC-ESI-MS: 545 $[M+Na]^+$; HRMS (ESI) calcd for $C_{28}H_{27}FN_2NaO_5S$ $[M+Na]^+$: 545.1517; Found: 545.1525.

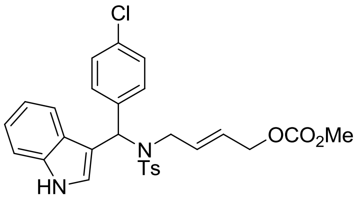

7g

Viscous yellow oil. ^{1}H NMR (300 MHz, $CDCl_3$) δ 2.37 (s, 3H), 3.70 (s, 3H), 3.86 (d, *J* = 6.0 Hz, 2H), 4.11 (t, *J* = 4.8 Hz, 2H), 5.07 (dt, *J* = 15.9, 5.7 Hz, 1H), 5.24 (dt, *J* = 15.6, 6.0 Hz, 1H), 6.56 (s, 2H), 6.92 (dd, *J* = 7.8, 6.9 Hz, 1H), 7.04-7.17 (m, 8H), 7.26 (d, *J* = 8.1 Hz, 1H), 7.58 (d, *J* = 8.1 Hz, 2H), 8.62 (br s, 1H); ^{13}C NMR (75 MHz, $CDCl_3$) δ 21.3, 46.7, 54.6, 58.0, 67.0, 111.3, 113.1, 119.0, 119.6, 122.2, 125.3, 125.4, 126.2, 127.1, 128.1, 129.3, 131.6, 132.9, 136.1, 137.2, 137.9, 143.2, 155.2; IR (thin film): ν_{max} (cm^{-1}) = 3385, 2959, 1746, 1490, 1443, 1334, 1262, 1156, 1090, 793, 662; HRMS (ESI) calcd for $C_{28}H_{31}ClN_3O_5S$ $[M+NH_4]^+$: 556.1658; Found: 556.1658.

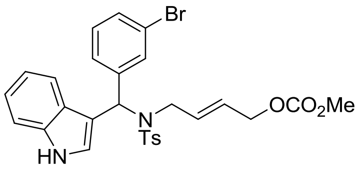

7h

Viscous yellow oil. ^{1}H NMR (300 MHz, $CDCl_3$) δ 2.38 (s, 3H), 3.70 (s, 3H), 3.88 (t, *J* = 6.0 Hz, 2H), 4.09 (t, *J* = 6.6 Hz, 2H), 5.04 (dt, *J* = 15.6, 6.0 Hz, 1H), 5.26 (dt, *J* = 15.3, 6.6 Hz, 1H), 6.58 (s, 2H), 6.96 (t, *J* = 7.5 Hz, 1H), 7.02-7.18 (m, 7H), 7.26-7.33 (m, 2H), 7.58 (d, *J* = 8.4 Hz, 2H), 8.56 (br s, 1H); ^{13}C NMR (75 MHz, $CDCl_3$) δ 21.4, 46.9, 54.6, 58.0, 67.1, 111.3, 113.3, 119.1, 119.8, 122.1, 122.3, 125.3, 125.4, 126.3, 126.6, 127.1, 129.3, 129.6, 130.2, 130.6, 131.7, 136.0, 137.0, 141.6, 143.3, 155.2; IR (thin film): ν_{max} (cm^{-1}) = 3402, 2974, 1746, 1441, 1333, 1264, 1155, 1089, 1048, 942, 792, 663; HRMS (ESI) calcd for $C_{28}H_{31}BrN_3O_5S$ $[M+NH_4]^+$: 600.1162; Found: 600.1151.

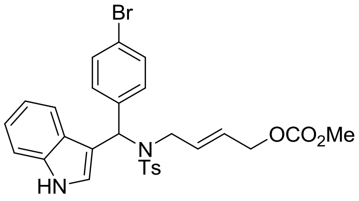

7i

Viscous yellow oil. ^{1}H NMR (300 MHz, $CDCl_3$) δ 2.40 (s, 3H), 3.73 (s, 3H), 3.88 (d, *J* = 6.6 Hz, 2H), 4.06-4.19 (m, 2H), 5.08 (dt, *J* = 15.6, 6.3 Hz, 1H), 5.21-5.30 (m, 1H), 6.55 (s, 1H), 6.62 (d, *J* = 2.1 Hz, 1H), 6.93-7.20 (m, 7H), 7.28-7.34 (m, 3H), 7.60 (d, *J* = 8.1 Hz, 2H), 8.40 (br s, 1H); ^{13}C NMR (75 MHz, $CDCl_3$) δ 21.4, 46.9, 54.7, 58.2, 67.1, 111.3, 113.5, 119.3, 119.9, 121.2, 122.4, 125.2, 125.5, 126.3, 127.3, 129.4, 129.8, 131.2, 131.8, 136.1, 137.4, 138.5, 143.3, 155.3; IR (thin film): ν_{max} (cm^{-1}) = 3377, 2957, 1746, 1598, 1448, 1332, 1256, 1555, 1088, 872, 733, 655, 613; HRMS (ESI) calcd for $C_{28}H_{27}BrN_2O_5S$ $[M+NH_4]^+$: 600.1162; Found: 600.1151.

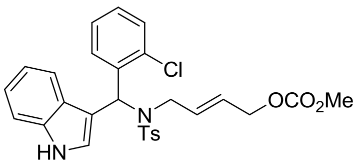

7j

Viscous yellow oil. ^{1}H NMR (300 MHz, $CDCl_3$) δ 2.36 (s, 3H), 3.69 (s, 3H), 3.84-4.03 (m, 2H), 4.09 (dd, *J* = 5.4, 5.1 Hz, 2H), 5.00 (dt, *J* = 15.6, 6.0 Hz, 1H), 5.35 (dt, *J* = 15.3, 6.3 Hz, 1H), 6.49 (d, *J* = 2.1 Hz, 1H), 6.80 (s, 1H), 6.87-6.97 (m, 2H), 7.03-7.16 (m, 5H), 7.22-7.26 (m, 3H), 7.59 (d, *J* = 8.1 Hz, 2H), 8.49 (br s, 1H); ^{13}C NMR (75 MHz, $CDCl_3$) δ 21.3, 47.6, 54.6, 55.9, 67.0, 111.3, 112.6, 118.6, 119.6, 122.2, 125.4, 125.5, 126.4, 127.3, 128.3, 128.8, 129.2, 129.5, 131.9, 133.2, 136.0, 136.9, 137.8, 143.1, 155.2; IR (thin film): ν_{max} (cm^{-1}) = 3397, 2955, 2924, 1747, 1547, 1442, 1336, 1263, 1157, 1089, 943, 744, 663; HRMS (ESI) calcd for $C_{28}H_{31}ClN_3O_5S$ $[M+NH_4]^+$: 556.1659; Found: 556.1667.

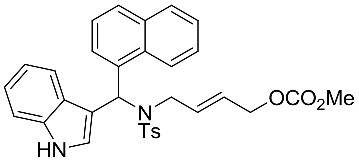

7k

Viscous yellow oil. ^{1}H NMR (400 MHz, $CDCl_3$) δ 2.33, 2.38 (s, 3H), 3.50-3.52 (m, 0.4H), 3.67, 3.73 (s, 3H), 3.81-4.45 (m, 4H), 4.76-4.83 (m, 1H), 5.01-5.18 (m, 1H), 5.60-5.63 (m, 0.4H), 6.33 (d, *J* = 2.0 Hz, 1H), 6.96 (t, *J* = 7.2 Hz, 1H), 7.04 (d, *J* = 8.0 Hz, 2H), 7.10-7.17 (m, 3H), 7.24 (d, *J* = 8.0 Hz, 2H), 7.31-7.41 (m, 3H), 7.50 (d, *J* = 8.4 Hz, 2H), 7.69 (d, *J* = 6.8 Hz, 1H), 7.79 (d, *J* = 8.0 Hz, 1H), 8.03 (d, *J* = 8.4 Hz, 1H), 8.23 (br s, 1H); ^{13}C NMR (100 MHz, $CDCl_3$) δ 21.3, 21.4, 44.3, 47.4, 54.5, 54.7, 55.7, 67.0, 111.1, 114.0, 119.1, 119.7, 122.3, 123.9, 124.6, 124.8, 125.2, 125.4, 125.5, 126.2, 126.4, 127.0, 127.4, 128.2, 128.4, 129.1, 129.6, 129.8, 131.1, 132.0, 133.6, 134.7, 136.0, 137.2, 142.9, 155.2; IR (thin film): ν_{max} (cm^{-1}) = 3398, 3031, 1748, 1598, 1509, 1334, 1268, 1158, 946, 863, 792, 771, 666; LC-ESI-MS: 577 $[M+H]^+$; HRMS (MALDI) calcd for $C_{32}H_{30}N_2O_5SNa$ $[M+Na]^+$: 577.1768; Found: 577.1773.

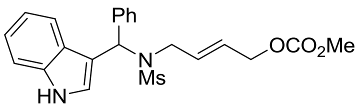

7l

Viscous yellow oil. ^{1}H NMR (400 MHz, $CDCl_3$) δ 2.73 (s, 3H), 3.70 (s, 3H), 3.95 (d, *J* = 3.2 Hz, 2H), 4.16-4.23 (m, 2H), 5.22-5.32 (m, 2H), 6.61 (s, 1H), 6.88 (d, *J* = 2.0 Hz, 1H), 7.04 (dt, *J* = 8.0, 0.8 Hz,

1H), 7.17 (ddd, J = 8.0, 6.8, 0.8 Hz, 1H), 7.30-7.35 (m, 4H), 7.37-7.39 (m, 2H), 7.45 (d, J = 8.0 Hz, 1H), 8.58 (br s, 1H); ^{13}C NMR (100 MHz, $CDCl_3$) δ 41.2, 46.8, 54.6, 58.7, 67.0, 111.4, 113.7, 119.4, 119.9, 122.4, 125.0, 126.3, 126.4, 127.7, 128.0, 128.5, 131.2, 136.3, 139.1, 155.2; IR (thin film): ν_{max} (cm^{-1}) = 3387， 2956， 1747， 1495， 1448， 1323， 1265， 1148， 963， 747; LC-ESI-MS: 451 $[M+Na]^+$; HRMS (ESI) calcd for $C_{22}H_{24}N_2NaO_5S$ $[M+Na]^+$: 451.1298; Found: 451.1303.

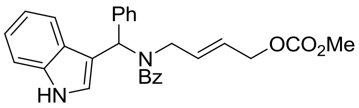

7m

Pale yellow solid. ^{1}H NMR (400 MHz, $CDCl_3$) δ 3.70 (s, 3H), 3.97-4.21 (m, 4H), 4.57-5.51 (m, 2H), 6.40-7.49 (m, 16H), 9.12 (br s, 1H); ^{13}C NMR (100 MHz, $CDCl_3$) δ 45.7, 54.5, 60.6, 67.5, 111.4, 114.3, 119.1, 119.5, 122.1, 124.6, 125.0, 126.3, 127.2, 127.6, 128.0, 128.35, 128.41, 129.7, 130.8, 136.3, 136.4, 139.3, 155.3, 172.2; IR (thin film): ν_{max} (cm^{-1}) = 3278, 3059, 2955, 1742, 1613, 1495, 1446, 1267, 1108, 945, 747, 701, 667; LC-ESI-MS: 477 $[M+Na]^+$; HRMS (ESI) calcd for $C_{28}H_{26}N_2NaO_4$ $[M+Na]^+$: 477.1785; Found: 477.1793; m.p. = 62-63 ^{o}C.

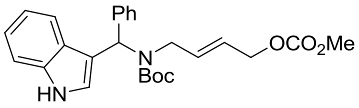

7n

Viscous yellow oil. ^{1}H NMR (300 MHz, $CDCl_3$) δ 1.46 (s, 9H), 3.67-3.76 (m, 4H), 3.92-3.97 (m, 1H), 4.09-4.22 (m, 2H), 5.07 (dt, J = 15.0, 5.7 Hz, 1H), 5.24 (br s, 1H), 6.86 (s, 1H), 7.08 (dd, J = 7.5, 6.9 Hz, 1H), 7.20 (dd, J = 7.8, 7.5 Hz, 1H), 7.30-7.41 (m, 8H), 8.12 (br s, 1H); ^{13}C NMR (75 MHz, $CDCl_3$) δ 28.4, 46.0, 54.6, 67.7, 80.0, 111.2, 115.4, 119.4, 119.7, 122.2, 124.1, 124.5, 127.0, 127.1, 127.6, 128.2, 132.6, 136.3, 155.4, 155.7; IR (thin film): ν_{max} (cm^{-1}) = 3306, 2929, 1747, 1664, 1445, 1366, 1255, 1161, 970, 887, 741, 699; HRMS (EI) calcd for $C_{26}H_{30}N_2O_5Na$ $[M+Na]^+$: 473.2047; Found: 473.2044.

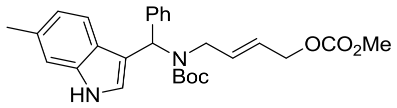

7o

Viscous yellow oil. ^{1}H NMR (400 MHz, $CDCl_3$) δ 1.46 (s, 9H), 2.42 (s, 3H), 3.72 (s, 3H), 3.70-3.78 (m, 1H), 3.89-3.92 (m, 1H), 4.14-4.23 (m, 2H), 5.10 (dt, *J* = 15.2, 6.0 Hz, 1H), 5.20 (br s, 1H), 6.70 (s, 1H), 6.88 (d, *J* = 8.0 Hz, 1H), 7.10 (s, 1H), 7.22-7.30 (m, 6H), 8.29 (br s, 1H); ^{13}C NMR (100 MHz, $CDCl_3$) δ 21.6, 28.4, 46.0, 54.6, 56.1, 67.7, 80.0, 111.1, 115.1, 119.1, 121.4, 123.9, 124.1, 124.9, 126.9, 127.5, 127.7, 128.1, 132.0, 132.7, 136.8, 155.4, 155.7; IR (thin film): ν_{max} (cm^{-1}) = 3322, 2959, 2920, 2856, 1749, 1667, 1448, 1266, 1165, 945, 802, 702, 631; LC-ESI-MS: 487 $[M+Na]^+$; HRMS (ESI) calcd for $C_{27}H_{32}N_2NaO_5$ $[M+Na]^+$: 487.2203; Found: 487.2214.

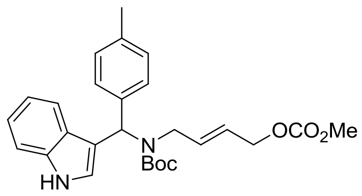

7p

Viscous yellow oil. ^{1}H NMR (400 MHz, $CDCl_3$) δ 1.47 (s, 9H), 2.34 (s, 3H), 3.71-3.77 (m, 4H), 3.90-3.93 (m, 1H), 4.13-4.20 (m, 2H), 5.07 (dt, *J* = 15.2, 6.4 Hz, 1H), 5.21 (br s, 1H), 6.65-6.91 (m, 2H), 7.05 (dd, *J* = 7.6, 7.2 Hz, 1H), 7.11-7.20 (m, 5H), 7.31 (d, *J* = 8.0 Hz, 1H), 7.38 (d, *J* = 8.0 Hz, 1H), 8.38 (br s, 1H); ^{13}C NMR (100 MHz, $CDCl_3$) δ 21.0, 28.4, 45.9, 54.6, 55.7, 67.7, 80.0, 111.1, 115.6, 119.5, 119.6, 122.2, 124.1, 124.4, 127.1, 127.5, 127.6, 128.9, 132.7, 136.3, 136.6, 155.4, 155.7; IR (thin film): ν_{max} (cm^{-1}) = 3317, 2963, 2928, 1750, 1667, 1447, 1263, 1164, 1021, 945, 794, 744; LC-ESI-MS: 487 $[M+Na]^+$; HRMS (ESI) calcd for $C_{27}H_{32}N_2NaO_5$ $[M+Na]^+$: 487.2203; Found: 487.2211.

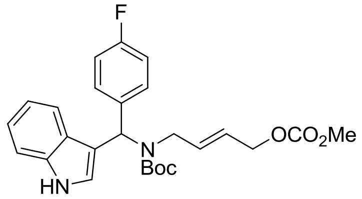

7q

Viscous yellow oil. ^{1}H NMR (400 MHz, $CDCl_3$) δ 1.47 (s, 9H), 3.70-3.78 (m, 4H), 3.87-3.91 (m, 1H), 4.14-4.23 (m, 2H), 5.09 (dt, *J* = 15.2, 6.4 Hz, 1H), 5.21 (br s, 1H), 6.63-6.88 (m, 2H), 6.97-7.02 (m, 2H), 7.06 (appt, *J* = 7.6 Hz, 1H), 7.17 (dt, *J* = 7.6, 0.8 Hz, 1H), 7.25-7.28 (m, 2H), 7.33 (dd, *J* = 7.6, 4.0 Hz, 2H), 8.46 (br s, 1H); ^{13}C NMR (100 MHz, $CDCl_3$) δ 28.3, 45.8, 54.5, 55.7, 67.5, 80.2, 111.3, 114.9 (d, *J* = 6.8 Hz), 115.0, 119.2, 119.6, 122.2, 124.3, 124.4, 126.8, 129.2 (d, *J* = 7.5 Hz), 132.3, 136.4, 155.3, 155.6, 161.8 (d, *J* = 243.5 Hz); IR (thin film): ν_{max} (cm^{-1}) = 3031, 2924, 1747, 1598, 1443, 1333, 1264, 1157, 1090, 792, 745, 663; LC-ESI-MS: 491 $[M+Na]^+$; HRMS (ESI) calcd for $C_{26}H_{29}FN_2NaO_5$ $[M+Na]^+$: 491.1953; Found: 491.1956.

General Procedure for Iridium-Catalyzed Asymmetric Allylic Alkylation of Indole Derivatives

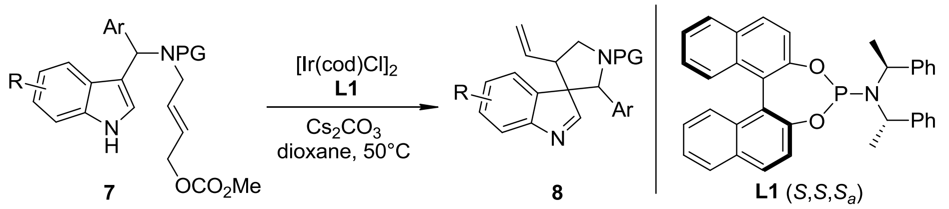

A flame-dried Schlenk tube was cooled to room temperature and filled with argon. To this flask were added $[Ir(cod)Cl]_2$ (2.7 mg, 0.004 mmol, 2 mol %), phosphoramidite ligand **L1** (4.2 mg, 0.008 mmol, 4 mol %), THF (0.5 mL) and n-propylamine (0.5 mL). The reaction mixture was heated at 50 ℃ for 30 min and then the volatile solvents were removed *in vacuo* to give a pale yellow solid. After that, allylic carbonate **7** (0.20 mmol, dissolved in 2.0 mL dioxane) and cesium carbonate (130.3 mg, 0.40 mmol, 200 mol %) were added. Then the reaction mixture was heated at 50 ℃. After the reaction was complete (monitored by TLC), the crude reaction mixture was filtrated with celite and washed with EtOAc. The solvents were removed under reduced pressure. The diastereomeric ratio was determined by ^{1}H NMR of the crude reaction mixture. Then the residue was purified by silica gel column chromatography (PE/EA = 2/1) to afford the desired product **8**.

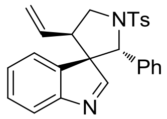

8aA

White solid, 42% yield, 98% ee [Daicel Chiralpak AD-H, *n*-hexane/2-propanol = 90/10, *v* = 1.0 mL·min^{-1}, λ = 254 nm, t (major) = 19.86 min, t (minor) = 30.76 min]; $[\alpha]_D^{20}$ = -11.3 (c = 1.0, $CHCl_3$). ^{1}H NMR (300 MHz, $CDCl_3$) δ 2.50 (s, 3H), 3.63-3.75 (m, 2H), 4.10-4.11 (m, 1H), 4.73-4.79 (m, 2H), 4.89-4.97 (m, 1H), 5.03 (s, 1H), 6.47 (d, *J* = 7.5 Hz, 1H), 6.99 (dd, *J* = 7.8, 7.2 Hz, 1H), 7.07 (s, 1H), 7.16 (br s, 2H), 7.31-7.35 (m, 6H), 7.56 (d, *J* = 7.5 Hz, 1H), 7.71 (d, *J* = 8.1 Hz, 2H); ^{13}C NMR (75 MHz, $CDCl_3$) δ 21.5, 44.9, 51.3, 67.9, 70.0, 119.7, 121.5, 122.2, 126.3, 126.4, 127.5, 128.1, 128.7, 129.8, 129.9, 135.2, 138.8, 140.2, 143.9, 155.2, 172.0; IR (thin film): ν_{max} (cm^{-1}) = 3853, 2978, 2897,

1653, 1558, 1352, 1168, 1093, 1045, 877, 775, 662; HRMS (ESI) calcd for $C_{26}H_{25}N_2O_2S$ $[M+H]^+$: 429.1631; Found: 429.1622; m.p. = 158-159 ℃.

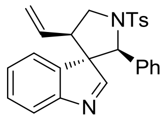

8aB

White solid, 4% yield, 97% ee [Daicel Chiralpak AD-H, *n*-hexane/2-propanol = 70/30, *v* = 1.0 mL·min^{-1}, λ = 254 nm, t (minor) = 9.72 min, t (major) = 15.93 min]; $[\alpha]_D^{20}$ = -16.0 (c = 0.25, $CHCl_3$). ^{1}H NMR (400 MHz, $CDCl_3$) δ 2.50 (s, 3H), 2.76-2.82 (m, 1H), 3.92 (appt, *J* = 12.0 Hz, 1H), 4.31 (dd, *J* = 12.0, 7.2 Hz, 1H), 4.71 (dt, *J* = 17.2, 1.2 Hz, 1H), 4.79 (dt, *J* = 10.4, 1.2 Hz, 1H), 5.09 (s, 1H), 5.13 (ddd, *J* = 17.2, 10.4, 7.2 Hz, 1H), 6.91 (appd, *J* = 7.2 Hz, 2H), 7.04-7.13 (m, 4H), 7.29 (ddd, *J* = 7.6, 7.2, 1.6 Hz, 1H), 7.34 (ddd, *J* = 7.6, 7.2, 1.2 Hz, 1H), 7.39-7.41 (m, 2H), 7.44-7.46 (m, 1H), 7.79 (d, *J* = 8.0 Hz, 2H), 7.90 (s, 1H); ^{13}C NMR (100 MHz, $CDCl_3$) δ 21.6, 50.0, 53.8, 70.1, 71.4, 118.8, 121.2, 121.4, 125.2, 126.7, 127.8, 127.9, 128.0, 129.1, 129.9, 134.3, 136.1, 136.4, 144.2, 155.7, 171.1; IR (thin film): ν_{max} (cm^{-1}) = 3066, 2924, 1596, 1555, 1457, 1351, 1165, 1093, 1049, 997, 932, 852, 812, 773, 747, 704, 662; HRMS (ESI) calcd for $C_{26}H_{25}N_2O_2S$ $[M+H]^+$: 429.1631; Found: 429.1619; m.p. = 149-150 ℃.

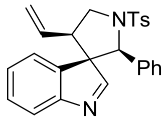

8aC

White solid, 37% yield, 98% ee [Daicel Chiralpak AD-H, *n*-hexane/2-propanol = 70/30, *v* = 1.0 mL·min^{-1}, λ = 254 nm, t (minor) = 11.30 min, t (major) = 23.35 min]; $[\alpha]_D^{20}$ = +82.9 (c = 1.0, $CHCl_3$). ^{1}H NMR (300 MHz, $CDCl_3$) δ 2.49 (s, 3H), 2.94-3.01 (m, 1H), 3.96 (appt, *J* = 11.1 Hz, 1H), 4.11-4.17 (m, 1H), 4.74-4.87 (m, 3H), 5.23 (s, 1H), 6.98-7.10 (m, 6H), 7.17 (dd, *J* = 7.8, 7.2 Hz, 1H), 7.26 (d, *J* = 7.5 Hz, 1H), 7.34-7.41 (m, 3H), 7.78 (d, *J* = 7.8 Hz, 2H), 7.87 (s, 1H); ^{13}C NMR (75 MHz, $CDCl_3$) δ 21.6, 47.0, 53.2, 66.4, 72.5, 119.1, 121.2, 125.2, 125.7, 126.1, 127.3, 127.5, 127.9, 128.5, 130.0, 130.6, 133.4, 134.5, 135.7, 144.3, 155.8, 171.7; IR (thin film): ν_{max} (cm^{-1}) = 3073, 2926, 1596, 1469,

1353, 1168, 1101, 1008, 934, 865, 775, 702, 663; HRMS (ESI) calcd for $C_{26}H_{25}N_2O_2S$ $[M+H]^+$: 429.1631; Found: 429.1627; m.p. = 150-151 ℃.

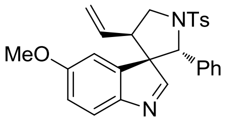

8bA

White solid, 44% yield, 92% ee [Daicel Chiralpak AD-H, *n*-hexane/2-propanol = 70/30, *v* = 1.0 mL·min^{-1}, λ = 254 nm, t (major) = 7.73 min, t (minor) = 10.44 min]; $[\alpha]_D^{20}$ = -35.8 (c = 1.0, $CHCl_3$). ^{1}H NMR (400 MHz, $CDCl_3$) δ 2.47 (s, 3H), 3.66-3.70 (m, 2H), 3.78 (s, 3H), 4.01-4.08 (m, 1H), 4.78-4.86 (m, 2H), 4.90-4.96 (m, 1H), 5.04 (s, 1H), 6.19 (d, *J* = 2.4 Hz, 1H), 6.83 (dd, *J* = 8.8, 2.8 Hz, 1H), 6.93 (s, 1H), 7.15 (d, *J* = 6.0 Hz, 2H), 7.29-7.33 (m, 5H), 7.45 (d, *J* = 8.4 Hz, 1H), 7.69 (d, *J* = 8.4 Hz, 2H); ^{13}C NMR (100 MHz, $CDCl_3$) δ 21.5, 45.1, 51.1, 55.7, 68.2, 70.1, 109.9, 112.4, 119.6, 121.7, 126.3, 127.4, 128.1, 128.7, 129.8, 130.1, 135.1, 140.2, 140.6, 144.2, 148.8, 158.8, 169.8; IR (thin film): ν_{max} (cm^{-1}) = 2921, 2851, 1717, 1619, 1594, 1351, 1169, 1094, 1032, 859, 815, 703, 663; LC-ESI-MS: 459 $[M+H]^+$; HRMS (ESI) calcd for $C_{27}H_{27}N_2O_3S$ $[M+H]^+$: 459.1737; Found: 459.1727; m.p. = 190-191 ℃.

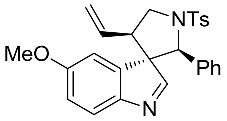

8bB

White solid, 10% yield, 94% ee [Daicel Chiralpak AD-H, *n*-hexane/2-propanol = 70/30, *v* = 1.0 mL·min^{-1}, λ = 254 nm, t (minor) = 11.62 min, t (major) = 21.23 min]; $[\alpha]_D^{20}$ = -64.0 (c = 1.0, $CHCl_3$). ^{1}H NMR (400 MHz, $CDCl_3$) δ 2.50 (s, 3H), 2.71-2.77 (m, 1H), 3.86 (s, 3H), 4.32 (dd, *J* = 12.0, 7.2 Hz, 1H), 4.74 (d, *J* = 17.2 Hz, 1H), 4.82 (d, *J* = 11.2 Hz, 1H), 5.04 (s, 1H), 5.14 (ddd, *J* = 17.2, 10.8, 7.2 Hz, 1H), 6.61 (d, *J* = 2.4 Hz, 1H), 6.84 (dd, *J* = 8.8, 2.8 Hz, 1H), 6.92 (appd, *J* = 7.2 Hz, 2H), 7.06-7.12 (m, 3H), 7.35 (d, *J* = 8.4 Hz, 1H), 7.40 (d, *J* = 8.4 Hz, 2H), 7.74 (s, 1H), 7.78 (d, *J* = 8.4 Hz, 2H); ^{13}C NMR (100 MHz, $CDCl_3$) δ 21.6, 50.5, 53.9, 55.7, 70.4, 71.4, 108.2, 113.2, 118.8, 121.9, 125.3, 127.87, 127.93, 128.1, 129.9, 130.0, 130.1, 136.5, 138.2, 144.3, 149.4, 159.0, 169.1; IR (thin film):

ν_{max} (cm^{-1}) = 2967, 2924, 1722, 1596, 1472, 1351, 1236, 1164, 1092, 1029, 854, 768, 702, 662; LC-ESI-MS: 459 [M+H]$^+$; HRMS (ESI) calcd for $C_{27}H_{27}N_2O_3S$ [M+H]$^+$: 459.1737; Found: 459.1737; m.p. = 161-162 ℃.

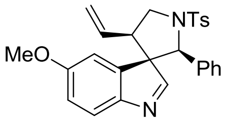

8bC

White solid, 32% yield, 98% ee [Daicel Chiralpak AD-H, *n*-hexane/2-propanol = 70/30, *v* = 1.0 mL·min^{-1}, λ = 254 nm, t (minor) = 14.04 min, t (major) = 20.70 min]; $[\alpha]_D^{20}$ = +46.4 (c = 0.5, $CHCl_3$). ^{1}H NMR (400 MHz, $CDCl_3$) δ 2.48 (s, 3H), 2.91-2.97 (m, 1H), 3.71 (s, 3H), 3.92 (t, *J* = 11.2 Hz, 1H), 4.12 (dd, *J* = 12.0, 8.0 Hz, 1H), 4.79 (dt, *J* = 11.6, 2.0 Hz, 1H), 4.86-4.97 (m, 2H), 5.23 (s, 1H), 6.67 (dd, *J* = 8.4, 2.4 Hz, 1H), 6.73 (d, *J* = 2.4 Hz, 1H), 6.99-7.05 (m, 5H), 7.24 (d, *J* = 8.4 Hz, 1H), 7.38 (d, *J* = 8.4 Hz, 2H), 7.71 (s, 1H), 7.77 (d, *J* = 8.4 Hz, 2H); ^{13}C NMR (100 MHz, $CDCl_3$) δ 21.6, 47.1, 53.1, 55.7, 66.3, 72.6, 112.4, 113.0, 119.0, 121.4, 126.2, 127.4, 127.5, 127.9, 129.9, 130.8, 133.7, 135.9, 136.2, 144.3, 149.7, 157.9, 169.8; IR (thin film): ν_{max} (cm^{-1}) = 2921, 2852, 1729, 1594, 1468, 1352, 1276, 1165, 1092, 1030, 932, 855, 703, 663; LC-ESI-MS: 459 [M+H]$^+$; HRMS (ESI) calcd for $C_{27}H_{27}N_2O_3S$ [M+H]$^+$: 459.1737; Found: 459.1729; m.p. = 123-124 ℃.

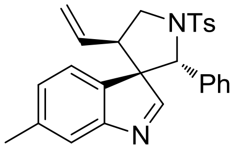

8cA

White solid, 48% yield, 97% ee [Daicel Chiralpak AD-H, *n*-hexane/2-propanol = 70/30, *v* = 1.0 mL·min^{-1}, λ = 254 nm, t (major) = 7.22 min, t (minor) = 10.38 min]; $[\alpha]_D^{20}$ = -13.7 (c = 1.0, $CHCl_3$). ^{1}H NMR (300 MHz, $CDCl_3$) δ 2.38 (s, 3H), 2.49 (s, 3H), 3.61-3.68 (m, 2H), 4.07-4.10 (m, 1H), 4.74-4.80 (m, 2H), 4.88-4.96 (m, 1H), 5.00 (s, 1H), 6.33 (d, *J* = 7.5 Hz, 1H), 6.79 (d, *J* = 7.5 Hz, 1H), 7.05 (s, 1H), 7.16 (br s, 2H), 7.26-7.37 (m, 6H), 7.70 (d, *J* = 8.4 Hz, 1H); ^{13}C NMR (75 MHz, $CDCl_3$) δ 21.4, 21.5, 44.9, 51.3, 68.1, 69.7, 119.4, 121.8, 122.2, 126.2, 127.0, 127.5, 128.0, 128.6, 129.7, 130.1,

135.2, 135.9, 138.7, 140.2, 143.8, 155.5, 172.2; IR (thin film): ν_{max} (cm^{-1}) = 2972, 2871, 1643, 1598, 1350, 1306, 1163, 1093, 1047, 1009, 926, 815, 772, 703, 660; HRMS (ESI) calcd for $C_{27}H_{27}N_2O_2S$ $[M+H]^+$: 443.1788; Found: 443.1782; m.p. = 138-139 ℃.

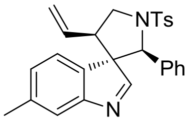

8cB

White solid, 11% yield, 96% ee [Daicel Chiralpak AD-H, *n*-hexane/2-propanol = 70/30, *v* = 1.0 mL · min^{-1}, λ = 254 nm, t (minor) = 10.64 min, t (major) = 14.05 min]; $[\alpha]_D^{20}$ = -33.4 (c = 1.0, $CHCl_3$). ^1H NMR (300 MHz, $CDCl_3$) δ 2.38 (s, 3H), 2.50 (s, 3H), 2.71-2.80 (m, 1H) 3.90 (t, *J* = 12.0 Hz, 1H), 4.29 (dd, *J* = 11.7, 7.2 Hz, 1H), 4.71 (d, *J* = 17.1 Hz, 1H), 4.79 (d, *J* = 10.2 Hz, 1H), 5.06 (s, 1H), 5.12 (ddd, *J* = 17.4, 10.5, 7.2 Hz, 1H), 6.90-6.98 (m, 3H), 7.04-7.14 (m, 4H), 7.25 (s, 1H), 7.40 (d, *J* = 8.1 Hz, 2H), 7.78 (d, *J* = 8.1 Hz, 2H), 7.87 (s, 1H); ^{13}C NMR (75 MHz, $CDCl_3$) δ 21.5, 21.6, 50.0, 53.8, 70.1, 71.1, 118.7, 120.8, 122.2, 125.3, 127.5, 127.8, 127.9, 128.0, 129.9, 130.0, 133.0, 134.4, 136.5, 139.2, 144.2, 156.0, 171.3; IR (thin film): ν_{max} (cm^{-1}) = 2924, 2854, 1715, 1619, 1454, 1352, 1164, 1093, 1005, 855, 815, 701, 661; HRMS (ESI) calcd for $C_{27}H_{27}N_2O_2S$ $[M+H]^+$: 443.1788; Found: 443.1788; m.p. = 170-171 ℃.

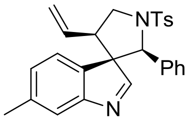

8cC

White solid, 30% yield, 97% ee [Daicel Chiralpak AD-H, *n*-hexane/2-propanol = 70/30, *v* = 1.0 mL · min^{-1}, λ = 254 nm, t (minor) = 10.33 min, t (major) = 15.38 min]; $[\alpha]_D^{20}$ = +83.3 (c = 1.0, $CHCl_3$). ^1H NMR (300 MHz, $CDCl_3$) δ 2.25 (s, 3H), 2.49 (s, 3H), 2.92-2.96 (m, 1H), 3.93 (t, *J* = 11.4 Hz, 1H), 4.12 (dd, *J* = 11.7, 7.8 Hz, 1H), 4.74-4.88 (m, 3H), 5.20 (s, 1H), 6.88 (d, *J* = 7.8 Hz, 1H), 6.98-7.05 (m, 5H), 7.11 (d, *J* = 7.8 Hz, 1H), 7.15 (s, 1H), 7.39 (d, *J* = 7.8 Hz, 2H), 7.77 (d, *J* = 8.4 Hz, 2H), 7.82 (s, 1H); ^{13}C NMR (100 MHz, $CDCl_3$) δ 21.3, 21.6, 47.1, 53.2, 66.5, 72.2, 118.9, 121.9, 124.8, 126.2, 126.4, 127.3, 127.4, 127.9, 129.9, 130.8, 131.4, 133.6, 135.9, 138.5, 144.3, 156.1, 171.8; IR (thin film):

ν_{max} (cm^{-1}) = 2925, 1712, 1620, 1597, 1454, 1350, 1163, 1092, 990, 815, 701, 662; LC-ESI-MS: 443 $[M+H]^+$; HRMS (ESI) calcd for $C_{27}H_{26}N_2NaO_2S$ $[M+Na]^+$: 465.1607; Found: 465.1624; m.p. = 144-145 ℃.

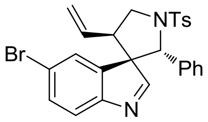

8dA

White solid, 42% yield, 91% ee [Daicel Chiralpak AD-H, *n*-hexane/2-propanol = 70/30, *v* = 1.0 mL·min^{-1}, λ = 254 nm, t (major) = 8.48 min, t (minor) = 12.48 min]; $[\alpha]_D^{20}$ = -12.4 (c = 1.0, $CHCl_3$). ^{1}H NMR (400 MHz, $CDCl_3$) δ 2.51 (s, 3H), 3.53 (dd, *J* = 10.8, 9.2 Hz, 1H), 3.68-3.75 (m, 1H), 4.18 (dd, *J* = 9.2, 7.2 Hz, 1H), 4.70-4.83 (m, 2H), 4.93-4.98 (m, 2H), 6.37 (d, *J* = 1.6 Hz, 1H), 7.02 (s, 1H), 7.18-7.47 (m, 9H), 7.77 (d, *J* = 8.0 Hz, 2H); ^{13}C NMR (100 MHz, $CDCl_3$) δ 22.0, 44.9, 51.4, 67.8, 70.8, 120.2, 120.4, 122.8, 125.3, 126.2, 127.4, 128.3, 128.9, 129.3, 130.3, 132.0, 134.4, 140.4, 141.2, 144.6, 154.2, 172.5; IR (thin film): ν_{max} (cm^{-1}) = 3085, 2924, 1642, 1598, 1494, 1353, 1167, 1097, 1009, 857, 703, 672; HRMS (MALDI) calcd for $C_{26}H_{24}N_2O_2SBr$ $[M+H]^+$: 507.0736; Found: 507.0734; m.p. = 160-161 ℃.

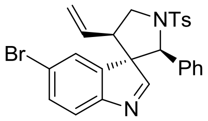

8dB

White solid, 23% yield, 87% ee [Daicel Chiralpak AD-H, *n*-hexane/2-propanol = 70/30, *v* = 1.0 mL·min^{-1}, λ = 254 nm, t (minor) = 10.03 min, t (major) = 18.37 min]; $[\alpha]_D^{20}$ = -83.5 (c = 1.0, $CHCl_3$). ^{1}H NMR (400 MHz, $CDCl_3$) δ 2.53 (s, 3H), 2.72-2.78 (m, 1H) 3.91 (t, *J* = 11.6 Hz, 1H), 4.33 (dd, *J* = 12.4, 7.6 Hz, 1H), 4.76 (d, *J* = 17.2 Hz, 1H), 4.84 (d, *J* = 10.4 Hz, 1H), 5.03 (s, 1H), 5.14 (ddd, *J* = 17.6, 10.8, 7.6 Hz, 1H), 6.93 (d, *J* = 7.2 Hz, 1H), 7.08-7.14 (m, 4H), 7.32 (d, *J* = 8.0 Hz, 1H), 7.43 (d, *J* = 8.4 Hz, 2H), 7.47 (dd, *J* = 8.0, 2.0 Hz, 1H), 7.79 (d, *J* = 8.0 Hz, 2H), 7.83 (s, 1H); ^{13}C NMR (100 MHz, $CDCl_3$) δ 21.7, 50.4, 54.0, 69.8, 71.8, 119.2, 120.5, 122.8, 124.7, 125.2, 127.8, 128.1, 128.2, 129.6, 130.1, 132.2, 134.2, 136.4, 139.0, 144.6, 154.5, 171.7; IR (thin film): ν_{max} (cm^{-1}) = 2916, 2851,

1775, 1739, 1598, 1451, 1354, 1166, 1006, 855, 817, 704, 677, 659, 630; LC-ESI-MS: 507 $[M+H]^+$; HRMS (MALDI) calcd for $C_{26}H_{24}N_2O_2SBr$ $[M+H]^+$: 507.0736; Found: 507.0726; m.p. = 85-86 ℃.

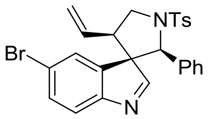

8dC

White solid, 21% yield, 92% ee [Daicel Chiralpak AD-H, *n*-hexane/2-propanol = 70/30, *v* = 1.0 mL · min^{-1}, λ = 254 nm, t (minor) = 12.92 min, t (major) = 28.67 min]; $[\alpha]_D^{20}$ = +100.0 (c = 0.3, $CHCl_3$). ^{1}H NMR (400 MHz, $CDCl_3$) δ 2.49 (s, 3H), 2.98-3.04 (m, 1H), 3.89 (t, *J* = 11.2 Hz, 1H), 4.15 (dd, *J* = 12.0, 8.0 Hz, 1H), 4.80-4.83 (m, 1H), 4.88-4.92 (m, 2H), 5.23 (s, 1H), 7.02 (s, 5H), 7.21 (d, *J* = 8.0 Hz, 1H), 7.29 (dd, *J* = 8.0, 2.0 Hz, 1H), 7.34 (d, *J* = 2.0 Hz, 1H), 7.39 (d, *J* = 8.0 Hz, 2H), 7.77 (d, *J* = 8.0 Hz, 2H), 7.85 (s, 1H); ^{13}C NMR (100 MHz, $CDCl_3$) δ 21.6, 47.1, 53.2, 66.3, 73.1, 119.55, 119.65, 122.5, 126.0, 127.4, 127.5, 127.7, 128.0, 128.5, 130.0, 130.3, 131.6, 133.5, 135.4, 136.8, 144.5, 154.8, 172.0; IR (thin film): ν_{max} (cm^{-1}) = 3366, 2922, 2851, 1735, 1470, 1455, 1350, 1164, 1093, 1043, 993, 815, 704, 664; LC-ESI-MS: 507 $[M+H]^+$; HRMS (MALDI) calcd for $C_{26}H_{24}N_2O_2SBr$ $[M+H]^+$: 507.0736; Found: 507.0749; m.p. = 181-182 ℃.

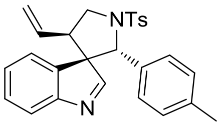

8eA

White solid, 44% yield, 95% ee [Daicel Chiralpak AD-H, *n*-hexane/2-propanol = 70/30, *v* = 0.7 mL · min^{-1}, λ = 254 nm, t (major) = 11.24 min, t (minor) = 15.04 min]; $[\alpha]_D^{20}$ = +36.0 (c = 0.25, $CHCl_3$). ^{1}H NMR (400 MHz, $CDCl_3$) δ 2.33 (s, 3H), 2.48 (s, 3H), 3.60-3.71 (m, 2H), 4.08 (dd, *J* = 7.6, 6.4 Hz, 1H), 4.69-4.77 (m, 2H), 4.87-4.93 (m, 1H), 4.97 (s, 1H), 6.43 (d, *J* = 7.2 Hz, 1H), 6.95 (appt, *J* = 8.0 Hz, 1H), 7.02-7.05 (m, 2H), 7.09 (s, 1H), 7.11 (d, *J* = 8.0 Hz, 2H), 7.28-7.33 (m, 3H), 7.53 (d, *J* = 7.6 Hz, 1H), 7.69 (d, *J* = 8.0 Hz, 2H); ^{13}C NMR (100 MHz, $CDCl_3$) δ 21.1, 21.5, 44.9, 51.3, 67.9, 70.2, 119.5, 121.5, 122.2, 126.2, 126.3, 127.5, 128.6, 129.4, 129.8, 130.1, 135.3, 137.3, 137.8, 138.9, 143.8, 155.2, 172.2; IR (thin film): ν_{max} (cm^{-1}) = 3025, 2922, 1597, 1557, 1494, 1455, 1352, 1208, 1166, 1101,

933, 816, 761, 662, 589, 550; LC-ESI-MS: 443 [M+H]$^+$; HRMS (ESI) calcd for $C_{27}H_{26}N_2NaO_2S$ [M+Na]$^+$: 465.1607; Found: 465.1616; m.p. = 171-172 ℃.

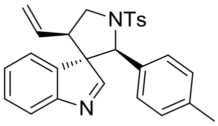

8eB

White solid, 4% yield, 80% ee [Daicel Chiralpak AD-H, *n*-hexane/2-propanol = 70/30, *v* = 0.7 mL·min^{-1}, λ = 254 nm, t (major) = 13.74 min, t (minor) = 15.22 min]; $[\alpha]_D^{20}$ = +40.0 (c = 0.1, $CHCl_3$). ^{1}H NMR (400 MHz, $CDCl_3$) δ 2.20 (s, 3H), 2.51 (s, 3H), 2.76-2.81 (m, 1H) 3.92 (t, *J* = 12.0 Hz, 1H), 4.29 (dd, *J* = 12.0, 7.2 Hz, 1H), 4.71 (d, *J* = 17.2 Hz, 1H), 4.79 (d, *J* = 10.4 Hz, 1H), 5.04 (s, 1H), 5.13 (ddd, *J* = 17.2, 10.4, 7.2 Hz, 1H), 6.79 (d, *J* = 7.6 Hz, 2H), 6.87 (d, *J* = 8.0 Hz, 2H), 7.08 (d, *J* = 7.2 Hz, 1H), 7.29-7.36 (m, 2H), 7.40 (d, *J* = 8.0 Hz, 2H), 7.45 (d, *J* = 7.6 Hz, 1H), 7.78 (d, *J* = 8.0 Hz, 2H), 7.92 (s, 1H); ^{13}C NMR (100 MHz, $CDCl_3$) δ 21.0, 21.7, 49.9, 53.8, 70.0, 71.4, 118.8, 121.2, 121.4, 125.1, 126.7, 127.8, 128.8, 129.0, 129.9, 130.3, 133.4, 134.4, 136.2, 137.6, 144.2, 155.7, 171.3; IR (thin film): ν_{max} (cm^{-1}) = 2958, 1596, 1455, 1351, 1165, 1093, 1000, 858, 762, 708, 663; LC-ESI-MS: 443 [M+H]$^+$; HRMS (ESI) calcd for $C_{27}H_{26}N_2NaO_2S$ [M+Na]$^+$: 465.1607; Found: 465.1603; m.p. = 174-175 ℃.

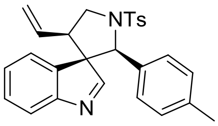

8eC

White solid, 39% yield, 95% ee [Daicel Chiralpak AD-H, *n*-hexane/2-propanol = 70/30, *v* = 1.0 mL·min^{-1}, λ = 254 nm, t (minor) = 7.78 min, t (major) = 13.51 min]; $[\alpha]_D^{20}$ = +69.0 (c = 0.25, $CHCl_3$). ^{1}H NMR (400 MHz, $CDCl_3$) δ 2.12 (s, 3H), 2.49 (s, 3H), 2.93-2.99 (m, 1H), 3.95 (appt, *J* = 12.0 Hz, 1H), 4.09-4.13 (m, 1H), 4.73-4.77 (m, 1H), 4.84-4.87 (m, 2H), 5.15 (s, 1H), 6.79 (d, *J* = 8.4 Hz, 2H), 6.93 (d, *J* = 8.0 Hz, 2H), 7.10 (dt, *J* = 7.2, 0.8 Hz, 1H), 7.19 (dt, *J* = 7.6, 1.2 Hz, 1H), 7.29 (d, *J* = 7.2 Hz, 1H), 7.36-7.40 (m, 3H), 7.76 (d, *J* = 8.4 Hz, 2H), 7.83 (s, 1H); ^{13}C NMR (100 MHz, $CDCl_3$) δ 21.0, 21.6, 46.9, 53.2, 66.5, 72.6, 119.0, 121.3, 125.3, 125.7, 126.2, 127.9, 128.1, 128.5, 129.9, 130.8,

132.6, 133.5, 134.6, 137.1, 144.3, 155.9, 171.7; IR (thin film): ν_{max} (cm^{-1}) = 3026, 2923, 1597, 1515, 1353, 1221, 1166, 1001, 819, 690, 663, 578, 544; MS (EI, *m/z*, rel. intensity) 442 ($[M]^+$, 22), 259 (100); HRMS (EI) calcd for $C_{27}H_{26}N_2O_2S$ $[M]^+$: 442.1715; Found: 442.1714; m.p. = 142-143 ℃.

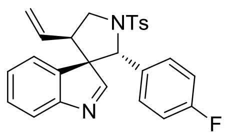

8fA

White solid, 38% yield, 94% ee [Daicel Chiralpak AD-H, *n*-hexane/2-propanol = 70/30, *v* = 1.0 mL · min^{-1}, λ = 254 nm, t (major) = 8.18 min, t (minor) = 12.90 min]; $[\alpha]_D^{20}$ = +1.3 (c = 1.0, $CHCl_3$). ^1H NMR (400 MHz, $CDCl_3$) δ 2.51 (s, 3H), 3.65 (d, *J* = 4.4 Hz, 2H), 4.09-4.11 (m, 1H), 4.74-4.81 (m, 2H), 4.92-4.96 (m, 1H), 4.99 (s, 1H), 6.41 (d, *J* = 7.6 Hz, 1H), 6.96-7.04 (m, 3H), 7.10 (s, 1H), 7.14 (br s, 2H), 7.31-7.36 (m, 3H), 7.56 (d, *J* = 8.4 Hz, 1H), 7.71 (d, *J* = 8.4 Hz, 2H); ^{13}C NMR (100 MHz, $CDCl_3$) δ 21.5, 44.9, 51.4, 67.4, 70.1, 115.7 (d, *J* = 21.6 Hz), 119.8, 121.6, 122.2, 126.5, 127.5, 128.0 (d, *J* = 8.2 Hz), 128.8, 129.8, 129.9, 135.1, 136.1, 138.7, 144.1, 155.2, 162.4 (d, *J* = 246.1 Hz), 171.7; ^{19}F NMR (376 MHz, $CDCl_3$) δ -113.9 (m); IR (thin film): ν_{max} (cm^{-1}) = 3418, 2922, 1599, 1557, 1509, 1351, 1224, 1167, 1095, 933, 757, 663, 589, 550; LC-ESI-MS: 447 $[M+H]^+$; HRMS (ESI) calcd for $C_{26}H_{24}FN_2O_2S$ $[M+H]^+$: 447.1537; Found: 447.1552; m.p. = 169-170 ℃.

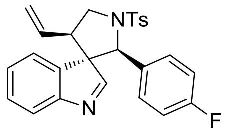

8fB

Viscous pale yellow oil, 13% yield, 91% ee [Daicel Chiralpak IC, *n*-hexane/2-propanol = 90/10, *v* = 1.0 mL · min^{-1}, λ = 254 nm, t (minor) = 53.40 min, t (major) = 58.82 min]; $[\alpha]_D^{20}$ = -26.0 (c = 0.5, $CHCl_3$). ^1H NMR (400 MHz, $CDCl_3$) δ 2.51 (s, 3H), 2.76-2.83 (m, 1H) 3.92 (t, *J* = 12.0 Hz, 1H), 4.31 (dd, *J* = 12.4, 7.6 Hz, 1H), 4.72 (d, *J* = 17.2 Hz, 1H), 4.81 (d, *J* = 10.4 Hz, 1H), 5.04 (s, 1H), 5.12 (ddd, *J* = 17.2, 10.4, 7.2 Hz, 1H), 6.75 (t, *J* = 8.4 Hz, 2H), 6.87 (dd, *J* = 8.0, 6.4 Hz, 2H), 7.11 (d, *J* = 7.2 Hz, 1H), 7.29-7.36 (m, 2H), 7.41 (d, *J* = 8.4 Hz, 2H), 7.47 (d, *J* = 8.0 Hz, 1H), 7.77 (d, *J* = 8.4 Hz, 2H),

7.91 (s, 1H); ^{13}C NMR (100 MHz, $CDCl_3$) δ 21.7, 49.8, 53.8, 69.6, 71.4, 115.0 (d, *J* = 20.8 Hz), 119.0, 121.2, 121.6, 126.9, 127.0, 127.0, 127.8, 129.3, 129.8, 129.9, 132.1, 134.2, 135.7, 144.4, 155.7, 162.2 (d, *J* = 243.9 Hz), 170.9; IR (thin film): ν_{max} (cm^{-1}) = 3028, 2925, 1599, 1556, 1509, 1352, 1224, 1167, 1093, 932, 761, 663, 578; LC-ESI-MS: 447 $[M+H]^+$; HRMS (ESI) calcd for $C_{26}H_{24}FN_2O_2S$ $[M+H]^+$: 447.1537; Found: 447.1538.

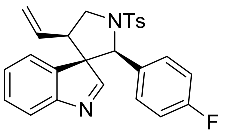

8fC

White solid, 30% yield, 96% ee [Daicel Chiralpak AD-H, *n*-hexane/2-propanol = 70/30, *v* = 1.0 mL·min^{-1}, λ = 254 nm, t (minor) = 7.66 min, t (major) = 13.79 min]; $[\alpha]_D^{20}$ = +84.8 (c = 0.5, $CHCl_3$). ^{1}H NMR (400 MHz, $CDCl_3$) δ 2.50 (s, 3H), 2.96-3.01 (m, 1H), 3.95 (appt, *J* = 10.8 Hz, 1H), 4.11-4.14 (m, 1H), 4.76-4.79 (m, 1H), 4.85-4.88 (m, 2H), 5.18 (s, 1H), 6.67 (t, *J* = 8.8 Hz, 2H), 7.01 (dd, *J* = 8.0, 5.6 Hz, 2H), 7.11 (dt, *J* = 7.6, 1.2 Hz, 1H), 7.20 (dt, *J* = 7.6, 1.2 Hz, 1H), 7.25-7.30 (m, 1H), 7.36-7.41 (m, 3H), 7.75 (d, *J* = 8.4 Hz, 2H), 7.85 (s, 1H); ^{13}C NMR (100 MHz, $CDCl_3$) δ 21.7, 46.9, 53.2, 65.9, 72.6, 114.3 (d, *J* = 21.6 Hz), 119.2, 121.5, 125.2, 125.9, 127.8 (d, *J* = 8.2 Hz), 127.9, 128.7, 130.0, 130.5, 131.5, 133.4, 134.3, 144.5, 155.9, 162.0 (d, *J* = 232.7 Hz), 171.5; IR (thin film): ν_{max} (cm^{-1}) = 2916, 2851, 2361, 1734, 1509, 1351, 1223, 1093, 994, 931, 663, 617; MS (EI, *m/z*, rel. intensity) 446 ($[M]^+$, 21), 263 (100); HRMS (EI) calcd for $C_{26}H_{23}N_2O_2FS$ $[M]^+$: 446.1464; Found: 446.1460; m.p. = 147-148 ℃.

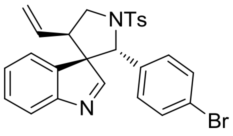

8iA

White solid, 42% yield, 91% ee [Daicel Chiralpak AD-H, *n*-hexane/2-propanol = 70/30, *v* = 1.0 mL·min^{-1}, λ = 254 nm, t (major) = 9.33 min, t (minor) = 18.99 min]; $[\alpha]_D^{20}$ = +41.9 (c = 1.0, $CHCl_3$). ^{1}H NMR (400 MHz, $CDCl_3$) δ 2.51 (s, 3H), 3.59-3.67 (m, 2H), 4.09 (dd, *J* = 7.6, 5.6 Hz, 1H), 4.70-

4.81 (m, 2H), 4.91 (d, *J* = 2.8 Hz, 1H), 4.95 (s, 1H), 6.38 (d, *J* = 7.2 Hz, 1H), 6.97 (dt, *J* = 7.6, 0.8 Hz, 1H), 7.06 (br s, 2H), 7.11 (s, 1H), 7.31-7.37 (m, 3H), 7.46 (d, *J* = 8.8 Hz, 2H), 7.56 (d, *J* = 7.6 Hz, 1H), 7.71 (d, *J* = 8.0 Hz, 2H); ^{13}C NMR (100 MHz, $CDCl_3$) δ 21.6, 45.0, 51.4, 67.4, 69.9, 119.9, 121.6, 122.1, 122.2, 126.5, 127.5, 128.0, 128.8, 129.7, 129.9, 131.9, 134.9, 138.6, 139.3, 144.2, 155.1, 171.6; IR (thin film): ν_{max} (cm^{-1}) = 3424, 2974, 1644, 1383, 1305, 1163, 1128, 949, 816, 657; HRMS (MALDI) calcd for $C_{26}H_{24}N_2O_2SBr$ [M+H]$^+$: 507.0736; Found: 507.0726; m.p. = 181-182 ℃.

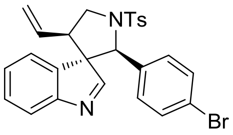

8iB

Viscous pale yellow oil, 7% yield, 76% ee [Daicel Chiralpak AD-H, *n*-hexane/2-propanol = 70/30, *v* = 1.0 mL · min^{-1}, λ = 254 nm, t (major) = 10.70 min, t (minor) = 14.29 min]; $[\alpha]_D^{20}$ = -47.0 (c = 0.5, $CHCl_3$). ^{1}H NMR (400 MHz, $CDCl_3$) δ 2.52 (s, 3H), 2.76-2.83 (m, 1H) 3.91 (t, *J* = 11.6 Hz, 1H), 4.29 (dd, *J* = 12.0, 7.6 Hz, 1H), 4.72 (d, *J* = 17.2 Hz, 1H), 4.81 (d, *J* = 10.4 Hz, 1H), 5.01 (s, 1H), 5.12 (ddd, *J* = 17.2, 10.4, 6.8 Hz, 1H), 6.78 (d, *J* = 8.0 Hz, 2H), 7.10 (d, *J* = 7.6 Hz, 1H), 7.19 (d, *J* = 8.4 Hz, 2H), 7.29-7.42 (m, 5H), 7.48 (d, *J* = 7.6 Hz, 1H), 7.77 (d, *J* = 8.4 Hz, 2H), 7.90 (s, 1H); ^{13}C NMR (100 MHz, $CDCl_3$) δ 21.7, 49.8, 53.7, 69.7, 71.2, 119.1, 121.2, 121.7, 121.9, 127.0, 127.4, 127.8, 129.3, 129.7, 130.0, 131.2, 134.1, 135.5, 135.6, 144.5, 155.7, 170.7; IR (thin film): ν_{max} (cm^{-1}) = 3027, 2923, 2851, 1595, 1488, 1353, 1230, 1166, 1094, 1010, 815, 753, 662, 578; HRMS (MALDI) calcd for $C_{26}H_{24}N_2O_2SBr$ [M+H]$^+$: 507.0736; Found: 507.0730.

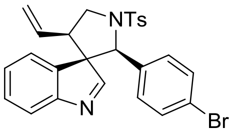

8iC

White solid, 34% yield, 92% ee [Daicel Chiralpak AD-H, *n*-hexane/2-propanol = 70/30, *v* = 1.0 mL · min^{-1}, λ = 254 nm, t (minor) = 9.89 min, t (major) = 20.81 min]; $[\alpha]_D^{20}$ = +114.2 (c = 0.5, $CHCl_3$). ^{1}H NMR (400 MHz, $CDCl_3$) δ 2.50 (s, 3H), 2.95-3.01 (m, 1H), 3.94 (t, *J* = 11.6 Hz, 1H), 4.11-4.15 (m,

1H), 4.76-4.79 (m, 1H), 4.83-4.86 (m, 2H), 5.15 (s, 1H), 6.92 (d, *J* = 7.6 Hz, 2H), 7.10-7.13 (m, 3H), 7.21 (dt, *J* = 7.6, 1.2 Hz, 1H), 7.26 (d, *J* = 7.2 Hz, 1H), 7.37-7.41 (m, 3H), 7.75 (d, *J* = 8.4 Hz, 2H), 7.85 (s, 1H); ^{13}C NMR (100 MHz, $CDCl_3$) δ 21.6, 47.0, 53.1, 66.0, 72.3, 119.3, 121.4, 121.5, 125.1, 125.9, 127.9, 128.8, 130.0, 130.4, 130.5, 133.3, 134.1, 134.9, 144.6, 155.8, 171.3; IR (thin film): ν_{max} (cm^{-1}) = 2955, 2923, 2851, 1596, 1488, 1352, 1163, 1010, 804, 755, 661, 586, 573, 550; MS (EI, *m/z*, rel. intensity) 446 ([M]$^+$, 21), 263 (100); HRMS (MALDI) calcd for $C_{26}H_{24}N_2O_2SBr$ [M+H]$^+$: 507.0736; Found: 507.0750; m.p. = 171-172 ℃.

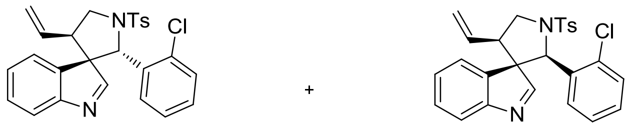

8jA + 8jC

1:0.67

8jA and **8jC** are inseparable diastereoisomers via column chromatography, the ratio of **8jA** and **8jC** is 1:0.67. The ^{1}H NMR, ^{13}C NMR and HPLC conditions of **8jA** and **8jC** are summarized as follow: ^{1}H NMR (400 MHz, $CDCl_3$) δ 2.49-2.52 (m, 5.5H) 2.98-2.93 (m, 0.65H), 3.54-3.67 (m, 2.2H), 3.98-4.16 (m, 2.6H), 4.61-4.94 (m, 5.5H), 5.41 (s, 1H), 5.65 (s, 0.7H), 6.07 (d, *J* = 7.2 Hz, 1.0H), 6.86-7.08 (m, 5.1H), 7.26-7.53 (m, 11H), 7.54 (d, *J* = 7.6 Hz, 1.2H), 7.67-7.61 (m, 2.0H), 7.85-7.77 (m, 4.6H); ^{13}C NMR (100 MHz, $CDCl_3$) δ 21.5, 45.0, 47.0, 51.6, 53.1, 63.1, 64.2, 69.4, 71.9, 119.1, 119.8, 121.3, 121.4, 121.7, 125.4, 125.6, 126.1, 127.0, 127.7, 127.9, 128.0, 128.6, 128.8, 129.1, 129.3, 129.6, 130.0, 130.3, 130.8, 132.0, 132.3, 133.6, 134.2, 137.8, 138.5, 144.2, 144.5, 155.2, 156.0, 171.1, 172.0; **8jA** 93% ee [Daicel Chiralpak AD-H, *n*-hexane/2-propanol = 70/30, *v* = 1.0 mL · min^{-1}, λ = 254 nm, t (major) = 6.47 min, t (minor) = 13.95 min]. **8jC** 95% ee [Daicel Chiralpak AD-H, *n*-hexane/2-propanol = 70/30, *v* = 1.0 mL · min^{-1}, λ = 254 nm, t (minor) = 8.07 min, t (major) = 9.45 min].

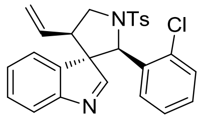

8jB

Viscous pale yellow oil, 15% yield, 98% ee [Daicel Chiralpak AD-H, *n*-hexane/2-propanol = 70/30, *v* = 1.0 mL · min^{-1}, λ = 254 nm, t (minor) = 8.68 min, t (minor) = 20.00 min]; $[\alpha]_D^{20}$ = -45.9 (c = 1.0,

$CHCl_3$). ^{1}H NMR (400 MHz, $CDCl_3$) δ 2.51 (s, 3H), 2.74-2.80 (m, 1H) 3.98 (dd, *J* = 12.0, 10.8 Hz, 1H), 4.27 (dd, *J* = 12.0, 5.7 Hz, 1H), 4.74 (dt, *J* = 17.2, 1.2 Hz, 1H), 4.83 (dt, *J* = 10.4, 0.8 Hz, 1H), 5.24 (ddd, *J* = 17.2, 10.4, 7.2 Hz, 1H), 5.63 (s, 1H), 6.93 (d, *J* = 7.2 Hz, 1H), 7.03-7.10 (m, 2H), 7.16-7.23 (m, 2H), 7.30 (dt, *J* = 7.6, 0.8 Hz, 1H), 7.41-7.44 (m, 3H), 7.69 (dd, *J* = 8.0, 1.2 Hz, 1H), 7.83 (d, *J* = 8.0 Hz, 2H), 7.94 (s, 1H); ^{13}C NMR (100 MHz, $CDCl_3$) δ 21.6, 50.8, 53.9, 65.4, 70.5, 118.9, 121.3, 122.2, 126.4, 126.6, 128.1, 128.8, 128.9, 129.6, 130.0, 130.4, 132.2, 133.8, 135.3, 136.4, 144.4, 155.2, 170.7; IR (thin film): ν_{max} (cm^{-1}) = 3064, 2922, 2851, 1721, 1459, 1164, 1093, 854, 775, 663; HRMS (MALDI) calcd for $C_{26}H_{24}N_2O_2SCl$ $[M+H]^+$: 463.1242; Found: 463.1229.

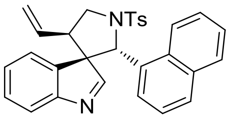

8kA

White solid, 43% yield, 90% ee [Daicel Chiralpak AD-H, *n*-hexane/2-propanol = 70/30, *v* = 1.0 mL · min^{-1}, λ = 254 nm, t (major) = 6.98 min, t (minor) = 15.07 min]; $[\alpha]_D^{20}$ = -37.8 (c = 1.0, $CHCl_3$). ^{1}H NMR (400 MHz, $CDCl_3$) δ 2.51 (s, 3H), 3.69-3.78 (m, 2H), 4.16 (dd, *J* = 7.2, 6.0 Hz, 1H), 4.65-4.77 (m, 2H), 4.89 (dd, *J* = 15.6, 2.4 Hz, 1H), 5.89 (s, 1H), 6.57 (d, *J* = 7.6 Hz, 1H), 6.77 (s, 1H), 7.04-7.08 (m, 2H), 7.25-7.47 (m, 6H), 7.59 (d, *J* = 7.6 Hz, 1H), 7.63 (d, *J* = 6.8 Hz, 1H), 7.73 (d, *J* = 8.0 Hz, 2H), 7.82 (d, *J* = 8.4 Hz, 1H), 7.86 (d, *J* = 8.4 Hz, 1H); ^{13}C NMR (100 MHz, $CDCl_3$) δ 21.6, 45.7, 51.2, 64.2, 69.9, 119.8, 121.7, 122.0, 122.3, 124.3, 125.0, 126.1, 126.5, 126.7, 127.6, 128.8, 128.9, 129.6, 129.9, 130.0, 133.6, 135.20, 135.25, 138.6, 144.0, 155.4, 172.3; IR (thin film): ν_{max} (cm^{-1}) = 3062, 2924, 1598, 1452, 1351, 1165, 1101, 1025, 838, 775, 707, 664; LC-ESI-MS: 479 $[M+H]^+$; HRMS (MALDI) calcd for $C_{30}H_{27}N_2O_2S$ $[M+H]^+$: 479.1788; Found: 479.1774; m.p. = 65-66 ℃.

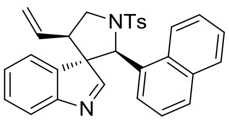

8kB

Viscous pale yellow oil, 15% yield, 83% ee [Daicel Chiralpak AD-H, *n*-hexane/2-propanol = 70/30, *v* = 1.0 mL · min^{-1}, λ = 254 nm, t (minor) = 8.98 min, t (major) = 31.43 min]; $[\alpha]_D^{20}$ = -51.6 (c = 0.5,

$CHCl_3$). ^{1}H NMR (400 MHz, $CDCl_3$) δ 2.50 (s, 3H), 2.92 (dd, *J* = 11.6, 7.2 Hz, 1H), 4.02 (dd, *J* = 10.4, 2.0 Hz, 1H), 4.44 (dd, *J* = 11.6, 6.8 Hz, 1H), 4.76 (dt, *J* = 17.2, 1.2 Hz, 1H), 4.84 (d, *J* = 10.4 Hz, 1H), 5.35 (ddd, *J* = 17.6, 10.4, 7.6 Hz, 1H), 5.99 (s, 1H), 6.87 (d, *J* = 8.4 Hz, 1H), 6.94-6.99 (m, 2H), 7.23-7.30 (m, 4H), 7.38-7.42 (m, 3H), 7.59 (s, 1H), 7.68 (t, *J* = 8.4 Hz, 2H), 7.79 (d, *J* = 8.0 Hz, 2H), 7.84 (d, *J* = 7.2 Hz, 1H); ^{13}C NMR (75 MHz, $CDCl_3$) δ 21.6, 52.0, 54.1, 65.2, 70.4, 118.7, 121.1, 121.6, 122.2, 123.9, 124.9, 125.3, 125.4, 126.8, 127.8, 128.4, 128.6, 129.0, 129.9, 130.1, 130.9, 133.6, 134.1, 134.8, 138.4, 144.3, 154.8, 172.0; IR (thin film): ν_{max} (cm^{-1}) = 3067, 2926, 1597, 1458, 1352, 1165, 1094, 989, 933, 772, 663; HRMS (ESI) calcd for $C_{30}H_{27}N_2O_2S$ $[M+H]^+$: 479.1788; Found: 479.1776.

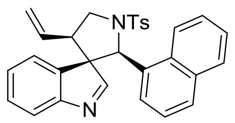

8kC

White solid, 25% yield, 95% ee [Daicel Chiralpak AD-H, *n*-hexane/2-propanol = 70/30, *v* = 1.0 mL·min^{-1}, λ = 254 nm, t (minor) = 9.60 min, t (major) = 15.33 min]; $[\alpha]_D^{20}$ = +83.4 (c = 0.5, $CHCl_3$). ^{1}H NMR (400 MHz, $CDCl_3$) δ 2.49 (s, 3H), 3.02 (dd, *J* = 16.4, 8.4 Hz, 1H), 4.04 (dd, *J* = 11.6, 8.8 Hz, 1H), 4.24 (dd, *J* = 11.6, 7.2 Hz, 1H), 4.80 (d, *J* = 10.0 Hz, 1H), 4.82 (d, *J* = 17.2 Hz, 1H), 5.16 (ddd, *J* = 16.8, 10.0, 8.0 Hz, 1H), 6.06 (s, 1H), 6.68 (d, *J* = 7.6 Hz, 1H), 6.76 (t, *J* = 7.2 Hz, 1H), 7.00 (dt, *J* = 7.2, 0.8 Hz, 1H), 7.18-7.32 (m, 5H), 7.38 (d, *J* = 8.4 Hz, 2H), 7.57 (d, *J* = 8.4 Hz, 1H), 7.64 (d, *J* = 8.0 Hz, 1H), 7.77 (d, *J* = 8.4 Hz, 2H), 7.82 (s, 1H), 7.83 (d, *J* = 11.2 Hz, 1H); ^{13}C NMR (100 MHz, $CDCl_3$) δ 21.6, 48.0, 53.4, 61.7, 71.5, 118.9, 121.0, 121.6, 124.4, 125.11, 125.14, 125.66, 125.75, 125.8, 127.9, 128.1, 128.2, 128.7, 130.0, 130.5, 131.8, 132.6, 133.1, 133.8, 134.2, 144.4, 155.5, 172.6; IR (thin film): ν_{max} (cm^{-1}) = 3382, 3251, 2980, 1644, 1383, 1086, 1045, 924, 878; LC-ESI-MS: 479 $[M+H]^+$; HRMS (MALDI) calcd for $C_{30}H_{27}N_2O_2S$ $[M+H]^+$: 479.1788; Found: 479.1782; m.p. = 144-145 ℃.

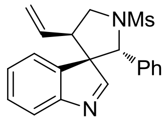

8lA

White solid, 44% yield, 93% ee [Daicel Chiralpak AD-H, *n*-hexane/2-propanol = 70/30, *v* = 1.0

mL · min^{-1}, λ = 254 nm, t (major) = 6.14 min, t (minor) = 7.85 min]; $[\alpha]_D^{20}$ = -9.3 (c = 1.0, $CHCl_3$). ^{1}H NMR (400 MHz, $CDCl_3$) δ 2.86 (s, 3H), 3.79 (ddd, *J* = 15.2, 10.0, 7.6 Hz, 1H), 4.00-4.11 (m, 2H), 4.85 (dd, *J* = 9.2, 2.8 Hz, 1H), 4.93-5.02 (m, 2H), 5.19 (s, 1H), 7.16 (s, 1H), 7.24 (d, *J* = 6.8 Hz, 2H), 7.34-7.45 (m, 5H), 7.62 (t, *J* = 8.0 Hz, 2H); ^{13}C NMR (100 MHz, $CDCl_3$) δ 41.1, 45.4, 51.3, 67.6, 69.8, 119.7, 121.6, 122.7, 126.3, 127.1, 128.6, 128.9, 129.1, 130.2, 138.7, 139.7, 155.2, 171.5; IR (thin film): ν_{max} (cm^{-1}) = 2955, 2923, 2851, 1557, 1452, 1328, 1148, 1017, 961, 789, 707, 550; MS (EI, *m/z*, rel. intensity) 352 ([M]$^+$, 7), 118 (100); HRMS (EI) calcd for $C_{20}H_{20}N_2O_2S$ [M]$^+$: 352.1245; Found: 352.1241; m.p. = 174-175 ℃.

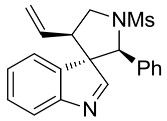

8lB

White solid, 8% yield, 90% ee [Daicel Chiralpak AD-H, *n*-hexane/2-propanol = 70/30, *v* = 1.0 mL · min^{-1}, λ = 254 nm, t (minor) = 8.10 min, t (major) = 16.68 min]; $[\alpha]_D^{20}$ = -139.0 (c = 0.25, $CHCl_3$). ^{1}H NMR (400 MHz, $CDCl_3$) δ 2.87 (s, 3H), 3.39-3.45 (m, 1H), 3.88 (t, *J* = 12.0 Hz, 1H), 4.56 (dd, *J* = 12.0, 8.0 Hz, 1H), 4.86 (d, *J* = 6.4 Hz, 1H), 4.89 (s, 1H), 5.23 (ddd, *J* = 16.8, 10.8, 7.2 Hz, 1H), 5.36 (s, 1H), 6.97 (d, *J* = 6.0 Hz, 2H), 7.11-7.16 (m, 3H), 7.38-7.40 (m, 2H), 7.47-7.49 (m, 1H), 7.56-7.58 (m, 1H), 8.03 (s, 1H); ^{13}C NMR (100 MHz, $CDCl_3$) δ 40.5, 51.0, 53.0, 70.0, 71.8, 119.3, 121.5, 121.7, 125.6, 127.0, 128.4, 129.2, 129.8, 135.8, 136.0, 155.7, 170.8; IR (thin film): ν_{max} (cm^{-1}) = 3316, 2923, 2851, 1455, 1342, 1153, 1018, 962, 783; LC-ESI-MS: 353 [M+H]$^+$; HRMS (ESI) calcd for $C_{20}H_{21}N_2O_2S$ [M+H]$^+$: 353.1318; Found: 353.1331; m.p. = 136-137 ℃.

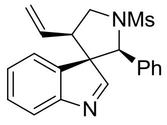

8lC

White solid, 35% yield, 95% ee [Daicel Chiralpak AD-H, *n*-hexane/2-propanol = 70/30, *v* = 1.0 mL · min^{-1}, λ = 254 nm, t (minor) = 8.75 min, t (major) = 11.00 min]; $[\alpha]_D^{20}$ = +54.5 (c = 1.0, $CHCl_3$). ^{1}H NMR (400 MHz, $CDCl_3$) δ 2.88 (s, 3H), 3.59-3.66 (m, 1H), 3.90 (t, *J* = 11.6 Hz, 1H), 4.39 (dd, *J* =

11.6, 8.0 Hz, 1H), 4.85 (dd, J = 10.0, 1.6 Hz, 1H), 4.89-4.97 (m, 1H), 5.04 (dd, J = 16.8, 1.6 Hz, 1H), 5.59 (s, 1H), 7.00-7.12 (m, 6H), 7.20 (dt, J = 7.6, 1.2 Hz, 1H), 7.31 (d, J = 7.6 Hz, 1H), 7.39 (d, J = 8.0 Hz, 1H), 8.16 (s, 1H); ^{13}C NMR (100 MHz, $CDCl_3$) δ 39.5, 48.1, 52.4, 66.1, 73.0, 119.4, 121.4, 125.1, 125.6, 126.1, 127.7, 127.9, 128.6, 130.4, 134.4, 135.4, 156.0, 171.9; IR (thin film): ν_{max} (cm^{-1}) = 2924, 2852, 1642, 1559, 1454, 1332, 1150, 991, 932, 812, 758, 703; MS (EI, *m/z*, rel. intensity) 352 ([M]$^+$, 36), 244 (100); HRMS (EI) calcd for $C_{20}H_{20}N_2O_2S$ [M]$^+$: 352.1245; Found: 352.1240; m.p. = 164-165 ℃.

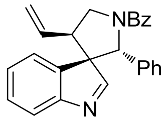

8mA

White solid, 44% yield, 92% ee [Daicel Chiralpak AD-H, *n*-hexane/2-propanol = 70/30, v = 1.0 mL·min^{-1}, λ = 254 nm, t (minor) = 9.21 min, t (major) = 11.60 min]; $[\alpha]_D^{20}$ = +7.0 (c = 1.0, $CHCl_3$). ^{1}H NMR (400 MHz, $CDCl_3$) δ 3.76-3.83 (m, 1H), 4.27-4.39 (m, 2H), 4.82-4.85 (m, 2H), 4.91-5.03 (m, 2H), 7.09-7.14 (m, 5H), 7.22-7.46 (m, 9H), 7.63 (d, J = 7.6 Hz, 1H); ^{13}C NMR (100 MHz, $CDCl_3$) δ 43.3, 50.4, 68.9, 70.2, 119.3, 121.7, 122.1, 126.3, 126.6, 127.0, 128.1, 128.4, 128.8, 129.1, 130.1, 131.0, 135.7, 139.0, 140.3, 155.4, 171.4, 171.7; IR (thin film): ν_{max} (cm^{-1}) = 3069, 2935, 2882, 1627, 1553, 1398, 1119, 998, 953, 822, 778, 762, 699, 637; MS (EI, *m/z*, rel. intensity) 378 ([M]$^+$, 78), 105 (100); HRMS (EI) calcd for $C_{26}H_{22}N_2O$ [M]$^+$: 378.1732; Found: 378.1736; m.p. = 121-122 ℃.

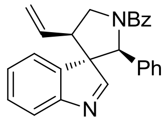

8mB

Viscous pale yellow oil, 12% yield, 78% ee [Daicel Chiralpak IC, *n*-hexane/2-propanol = 70/30, v = 1.0 mL·min^{-1}, λ = 254 nm, t (minor) = 11.47 min, t (major) = 14.62 min]; $[\alpha]_D^{20}$ = -112.5 (c = 0.5, $CHCl_3$). ^{1}H NMR (400 MHz, $CDCl_3$) δ 3.35-3.41 (m, 1H), 4.21-4.30 (m, 2H), 4.78 (d, J = 16.0 Hz, 1H), 4.81 (d, J = 9.6 Hz, 1H), 5.23 (ddd, J = 17.6, 10.0, 8.0 Hz, 1H), 6.87 (d, J = 3.6 Hz, 2H), 7.09-7.10 (m, 3H), 7.40-7.42 (m, 2H), 7.50-7.61 (m, 5H), 7.82 (d, J = 6.4 Hz, 2H), 8.04 (s, 1H); IR (thin

film): ν_{max} (cm^{-1}) = 3418, 2955, 2923, 2851, 1635, 1463, 1394, 1079, 1024, 932, 797, 718; MS (EI, *m/*z, rel. intensity) 378 ([M]$^+$, 90), 105 (100); HRMS (EI) calcd for $C_{26}H_{22}N_2O$ [M]$^+$: 378.1732; Found: 378.1728.

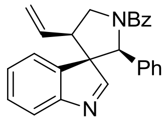

8mC

White solid, 37% yield, 92% ee [Daicel Chiralpak AD-H, *n*-hexane/2-propanol = 70/30, *v* = 1.0 mL·min^{-1}, λ = 254 nm, t (minor) = 7.12 min, t (major) = 8.53 min]; $[\alpha]_D^{20}$ = -77.9 (c = 1.0, $CHCl_3$). ^{1}H NMR (400 MHz, $CDCl_3$) δ 3.54-3.61 (m, 1H), 4.09-4.14 (m, 1H), 4.24-4.29 (m, 1H), 4.77-4.99 (m, 3H), 6.02 (s, 1H), 6.93-7.08 (m, 5H), 7.13-7.29 (m, 3H), 7.43 (d, *J* = 7.6 Hz, 1H), 7.51-7.52 (m, 4H), 7.84 (d, *J* = 4.8 Hz, 2H), 8.25 (s, 1H); ^{13}C NMR (100 MHz, $CDCl_3$) δ 48.3, 55.2, 60.3, 63.6, 71.3, 119.2, 121.3, 124.4, 124.8, 125.4, 126.6, 127.0, 127.8, 128.2, 128.3, 128.5, 130.4, 131.2, 135.3, 135.7, 136.2, 156.0, 171.2, 173.0; IR (thin film): ν_{max} (cm^{-1}) = 3081, 2927, 1724, 1638, 1450, 1388, 1281, 1177, 991, 796, 748, 700; MS (EI, *m/*z, rel. intensity) 378 ([M]$^+$, 76), 105 (100); HRMS (EI) calcd for $C_{26}H_{22}N_2O$ [M]$^+$: 378.1732; Found: 378.1735; m.p. = 164-165 °C.

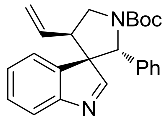

8nA

White solid, 41% yield, 95% ee [Daicel Chiralpak AD-H, *n*-hexane/2-propanol = 90/10, *v* = 1.0 mL·min^{-1}, λ = 254 nm, t (major) = 5.46 min, t (minor) = 6.14 min]; $[\alpha]_D^{20}$ = -10.3 (c = 1.0, $CHCl_3$). ^{1}H NMR (400 MHz, $CDCl_3$) δ 1.21 (s, 6H), 1.54 (s, 3H), 3.61-3.67 (m, 1H), 3.82-3.87 (m, 1H), 4.19-4.24 (m, 1H), 4.80-5.12 (m, 4H), 7.13-7.14 (m, 2H), 7.29-7.45 (m, 7H), 7.65 (d, *J* = 7.6 Hz, 1H); ^{13}C NMR (100 MHz, $CDCl_3$) δ 28.0, 28.5, 43.6, 44.6, 50.0, 50.4, 66.2, 66.6, 69.4, 70.2, 80.2, 80.4, 119.0, 121.6, 122.4, 125.9, 126.7, 127.8, 128.6, 128.7, 131.2, 139.7, 140.9, 154.1, 155.3, 172.5; IR (thin film): ν_{max} (cm^{-1}) = 2974, 2930, 1699, 1454, 1389, 1175, 1126, 779, 686; LC-ESI-MS: 375 [M+H]$^+$; HRMS (MALDI) calcd for $C_{24}H_{26}N_2NaO_2$ [M+Na]$^+$: 397.1887; Found: 397.1897; m.p. = 175-176 °C.

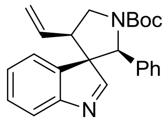

8nC

White solid, 40% yield, 97% ee [Daicel Chiralpak AD-H, *n*-hexane/2-propanol = 90/10, *v* = 0.6 mL·min^{-1}, λ = 254 nm, t (minor) = 16.99 min, t (major) = 18.01 min]; $[\alpha]_D^{20}$ = +8.7 (c = 1.0, $CHCl_3$). ^{1}H NMR (400 MHz, $CDCl_3$) δ 1.13 (br s, 6H), 1.50 (br s, 3H), 3.54-3.59 (m, 1H), 3.92 (t, *J* = 10.8 Hz, 1H), 4.25-4.31 (m, 1H), 4.80-4.83 (m, 1H), 4.93-5.03 (m, 2H), 5.45 (s, 1H), 6.91-7.06 (m, 6H), 7.15 (dt, *J* = 7.6, 1.2 Hz, 1H), 7.22 (s, 1H), 7.37 (d, *J* = 8.0 Hz, 1H), 8.17 (s, 1H); ^{13}C NMR (100 MHz, $CDCl_3$) δ 27.9, 46.9, 51.5, 64.1, 72.3, 80.4, 118.6, 121.1, 125.2, 125.4, 126.8, 127.3, 128.2, 131.4, 135.2, 137.6, 154.9, 155.9, 172.6; IR (thin film): ν_{max} (cm^{-1}) = 2957, 2924, 1697, 1454, 1388, 1366, 1169, 773, 702; LC-ESI-MS: 375 [M+H]$^+$; HRMS (MALDI) calcd for $C_{24}H_{26}N_2NaO_2$ [M+Na]$^+$: 397.1887; Found: 397.1898; m.p. = 128-129 °C.

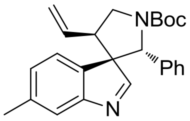

8oA

White solid, 41% yield, 90% ee [Daicel Chiralpak AD-H, *n*-hexane/2-propanol = 90/10, *v* = 0.6 mL·min^{-1}, λ = 254 nm, t (major) = 9.95 min, t (minor) = 10.85 min]; $[\alpha]_D^{20}$ = -24.4 (c = 1.0, $CHCl_3$). ^{1}H NMR (400 MHz, $CDCl_3$) δ 1.21 (s, 6H), 1.54 (s, 3H), 2.45 (s, 3H), 3.58-3.64 (m, 1H), 3.75-3.86 (m, 1H), 4.17-4.22 (m, 1H), 4.80-4.83 (m, 1H), 4.92-5.09 (m, 3H), 7.12-7.14 (m, 3H), 7.24-7.39 (m, 5H), 7.46 (s, 1H); ^{13}C NMR (100 MHz, $CDCl_3$) δ 21.5, 28.0, 28.5, 43.6, 44.5, 50.0, 50.5, 66.4, 66.8, 69.8, 80.1, 118.9, 122.0, 122.3, 125.9, 127.3, 127.7, 128.6, 131.4, 136.8, 138.8, 140.9, 154.2, 155.7, 172.7; IR (thin film): ν_{max} (cm^{-1}) = 2977, 2929, 1695, 1556, 1477, 1453, 1385, 1169, 1122, 898, 861, 749, 702, 665; LC-ESI-MS: 389 [M+H]$^+$; HRMS (MALDI) calcd for $C_{25}H_{28}N_2NaO_2$ [M+Na]$^+$: 411.2043; Found: 411.2060; m.p. = 165-166 °C.

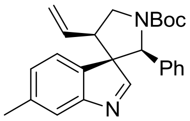

8oC

White solid, 37% yield, 94% ee [Daicel Chiralpak AD-H, *n*-hexane/2-propanol = 90/10, *v* = 0.8 mL·min^{-1}, λ = 254 nm, t (minor) = 12.95 min, t (major) = 15.63 min]; $[\alpha]_D^{20}$ = -5.1 (c = 1.0, $CHCl_3$). ^{1}H NMR (400 MHz, $CDCl_3$) δ 1.12 (br s, 6H), 1.51 (br s, 3H), 2.25 (s, 3H), 3.53 (br s, 1H), 3.90 (t, *J* = 11.2 Hz, 1H), 4.24-4.28 (br s, 1H), 4.79-4.82 (m, 1H), 4.97-5.00 (m, 2H), 5.42 (br s, 1H), 6.83-7.01 (m, 6H), 7.07-7.08 (m, 1H), 7.18 (s, 1H), 8.14 (s, 1H); ^{13}C NMR (100 MHz, $CDCl_3$) δ 21.3, 27.8, 47.0, 51.4, 64.2, 72.2, 80.3, 118.5, 121.8, 124.8, 125.3, 126.1, 126.7, 127.2, 131.6, 132.1, 138.1, 154.9, 156.2, 172.7; IR (thin film): ν_{max} (cm^{-1}) = 2954, 2924, 2853, 1695, 1455, 1384, 1167, 1125, 990, 924, 701; LC-ESI-MS: 389 $[M+H]^+$; HRMS (MALDI) calcd for $C_{25}H_{28}N_2NaO_2$ $[M+Na]^+$: 411.2043; Found: 411.2056; m.p. = 143-144 ℃.

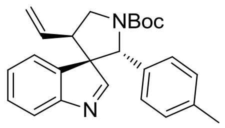

8pA

Pale yellow solid, 43% yield, 90% ee [Daicel Chiralpak AD-H, *n*-hexane/2-propanol = 90/10, *v* = 0.8 mL·min^{-1}, λ = 254 nm, t (major) = 6.80 min, t (minor) = 7.42 min]; $[\alpha]_D^{20}$ = -13.1 (c = 1.0, $CHCl_3$). ^{1}H NMR (400 MHz, $CDCl_3$) δ 1.22 (s, 6H), 1.54 (s, 3H), 2.37 (s, 3H), 3.60-3.66 (m, 1H), 3.75-3.86 (m, 1H), 4.13-4.22 (m, 1H), 4.78-4.81 (m, 1H), 4.93-5.08 (m, 3H), 7.01-7.03 (m, 2H), 7.17 (d, *J* = 8.0 Hz, 2H), 7.31-7.37 (m, 3H), 7.41-7.44 (m, 1H), 7.64 (d, *J* = 7.6 Hz, 1H); ^{13}C NMR (100 MHz, $CDCl_3$) δ 21.1, 27.9, 28.1, 28.5, 43.6, 44.6, 49.9, 50.4, 66.1, 66.4, 69.5, 70.3, 80.1, 118.9, 121.5, 122.4, 125.8, 126.7, 128.6, 129.3, 129.5, 131.3, 137.4, 137.8, 139.6, 139.8, 154.2, 155.4, 172.7; IR (thin film): ν_{max} (cm^{-1}) = 2975, 2929, 1696, 1384, 1170, 1123, 924, 899, 815, 703, 611; LC-ESI-MS: 389 $[M+H]^+$; HRMS (MALDI) calcd for $C_{25}H_{28}N_2NaO_2$ $[M+Na]^+$: 411.2043; Found: 411.2061; m.p. = 187-188 ℃.

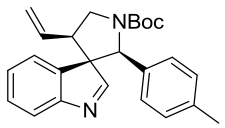

8pC

Pale yellow solid, 36% yield, 92% ee [Daicel Chiralpak AD-H, *n*-hexane/2-propanol = 90/10, *v* = 1.0 mL·min^{-1}, λ = 254 nm, t (minor) = 7.02 min, t (major) = 10.71 min]; $[\alpha]_D^{20}$ = +51.0 (c = 1.0, $CHCl_3$). ^1H NMR (400 MHz, $CDCl_3$) δ 1.14 (br s, 6H), 1.51 (br s, 3H), 2.11 (s, 3H), 3.51-3.56 (m, 1H), 3.91 (t, *J* = 11.2 Hz, 1H), 4.22-4.30 (m, 1H), 4.79-4.82 (m, 1H), 4.97-5.02 (m, 2H), 5.41 (s, 1H), 6.78 (s, 4H), 7.05 (t, *J* = 7.6 Hz, 1H), 7.16 (dt, *J* = 7.6, 1.2 Hz, 1H), 7.21-7.23 (m, 1H), 7.39 (d, *J* = 7.2 Hz, 1H), 8.16 (s, 1H); ^{13}C NMR (100 MHz, $CDCl_3$) δ 20.9, 27.9, 46.9, 51.5, 64.0, 67.9, 80.3, 118.5, 121.1, 125.2, 125.3, 125.4, 127.9, 128.1, 131.5, 135.4, 136.2, 154.9, 155.9, 172.8; IR (thin film): ν_{max} (cm^{-1}) = 3306, 2955, 2923, 2851, 1694, 1462, 1414, 1366, 1165, 1090, 1019, 893, 765, 743; LC-ESI-MS: 389 $[M+H]^+$; HRMS (MALDI) calcd for $C_{25}H_{28}N_2NaO_2$ $[M+Na]^+$: 411.2043; Found: 411.2056; m.p. = 120-121 ℃.

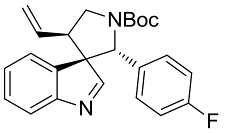

8qA

White solid, 39% yield, 89% ee [Daicel Chiralpak AD-H, *n*-hexane/2-propanol = 70/30, *v* = 1.0 mL·min^{-1}, λ = 254 nm, t (major) = 5.21 min, t (minor) = 5.76 min]; $[\alpha]_D^{20}$ = -17.9 (c = 1.0, $CHCl_3$). ^1H NMR (400 MHz, $CDCl_3$) δ 1.22 (s, 6H), 1.54 (s, 3H), 3.57-3.62 (m, 1H), 3.80-3.87 (m, 1H), 4.11-4.22 (m, 1H), 4.83-5.10 (m, 4H), 7.05-7.12 (m, 4H), 7.27-7.43 (m, 4H), 7.65 (d, *J* = 7.6 Hz, 1H); ^{13}C NMR (100 MHz, $CDCl_3$) δ 28.0, 28.4, 43.6, 44.6, 49.9, 50.4, 65.7, 65.9, 70.2, 80.4, 80.6, 115.6 (d, *J* = 21.5 Hz), 119.1, 121.6, 122.4, 126.8, 127.4 (d, *J* = 7.4 Hz), 128.8, 131.1, 136.7, 139.5, 154.0, 155.3, 162.2 (d, *J* = 244.7 Hz), 172.2; IR (thin film): ν_{max} (cm^{-1}) = 3357, 2974, 2886, 1651, 1454, 1381, 1087, 1045, 880, 805; LC-ESI-MS: 393 $[M+H]^+$; HRMS (ESI) calcd for $C_{24}H_{26}FN_2O_2$ $[M+H]^+$: 393.1973; Found: 393.1987; m.p. = 212-213 ℃.

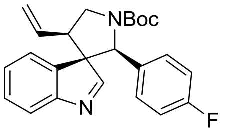

8qC

White solid, 38% yield, 91% ee [Daicel Chiralpak AD-H, *n*-hexane/2-propanol = 90/10, v = 1.0 mL·min^{-1}, λ = 254 nm, t (minor) = 12.33 min, t (major) = 13.40 min]; $[\alpha]_D^{20}$ = +7.5 (c = 1.0, $CHCl_3$). ^{1}H NMR (400 MHz, $CDCl_3$) δ 1.16 (br s, 6H), 1.48 (br s, 3H), 3.54-3.60 (m, 1H), 3.91 (t, *J* = 11.2 Hz, 1H), 4.27 (br s, 1H), 4.80-4.83 (m, 1H), 4.97-5.04 (m, 2H), 5.44 (s, 1H), 6.68 (t, *J* = 8.4 Hz, 2H), 6.88 (br s, 2H), 7.06 (t, *J* = 7.6 Hz, 1H), 7.16-7.21 (m, 2H), 7.40 (d, *J* = 7.6 Hz, 1H), 8.16 (s, 1H); ^{13}C NMR (100 MHz, $CDCl_3$) δ 27.9, 46.8, 51.5, 63.6, 72.4, 80.5, 114.2 (d, *J* = 22.3 Hz), 118.7, 121.3, 125.1, 125.5, 126.6, 128.3, 131.3, 135.1, 154.9, 155.9, 161.4 (d, *J* = 243.2 Hz), 172.4; IR (thin film): ν_{max} (cm^{-1}) = 2976, 2927, 1694, 1607, 1510, 1384, 1227, 1171, 1125, 838, 788, 758; LC-ESI-MS: 393 $[M+H]^+$; HRMS (ESI) calcd for $C_{24}H_{26}FN_2O_2$ $[M+H]^+$: 393.1973; Found: 393.1982; m.p. = 136-137 ℃.

General Procedure for the Migration of Spiroindolenine

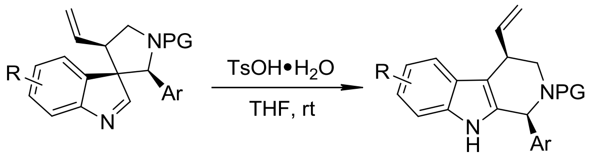

8C *cis*-**9**

To a solution of spiroindolenine **8C** (0.1 mmol, 1.0 equiv) in anhydrous THF (2 mL), *p*-toluenesulfonic acid (1.9 mg, 0.01 mmol, 0.1 equiv.) was added. The reaction mixture was stirred at room temperature. After the reaction was complete (monitored by TLC), the solvents were removed under reduced pressure. Then the residue was purified by silica gel column chromatography (PE/EA = 5/1) to afford the desired product *cis*-**9**.

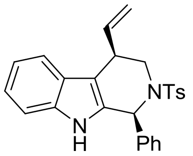

cis-**9a**

White solid, 91% yield, 98% ee [Daicel Chiralpak IC, *n*-hexane/2-propanol = 70/30, *v* = 1.0 mL · min^{-1}, λ = 254 nm, t (minor) = 5.07 min, t (major) = 5.82 min]; $[\alpha]_D^{20}$ = +40.5 (c = 1.0, $CHCl_3$). ^{1}H NMR (300 MHz, $CDCl_3$) δ 2.24 (s, 3H), 2.97 (dd, *J* = 14.4, 11.1 Hz, 1H), 3.43-3.52 (m, 1H), 3.84 (dd, *J* = 14.4, 5.7 Hz, 1H), 5.24 (d, *J* = 10.2 Hz, 1H), 5.33 (d, *J* = 17.1 Hz, 1H), 5.74 (ddd, *J* = 17.1, 10.2, 9.9 Hz, 1H), 6.29 (s, 1H), 7.00-7.05 (m, 3H), 7.14 (appt, *J* = 7.2 Hz, 1H), 7.24-7.29 (m, 6H), 7.48 (d, *J* = 8.1 Hz, 1H), 7.55 (d, *J* = 8.1 Hz, 2H), 7.89 (br s, 1H); ^{13}C NMR (75 MHz, $CDCl_3$) δ 21.3, 37.4, 44.7, 55.6, 110.9, 111.4, 117.9, 119.4, 119.9, 122.1, 126.4, 126.8, 128.4, 128.6, 129.3, 130.4, 136.1, 137.3, 138.0, 138.8, 143.3; IR (thin film): ν_{max} (cm^{-1}) = 3376, 2925, 1495, 1444, 1322, 1250, 1158, 1091, 1042, 920, 811, 754, 679, 659; HRMS (ESI) calcd for $C_{26}H_{25}N_2O_2S$ $[M+H]^+$: 429.1631; Found: 429.1622; m.p. = 246-247 ℃.

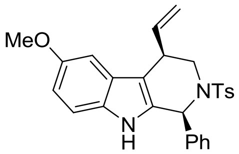

cis-**9b**

White solid, 90% yield, 98% ee [Daicel Chiralpak IC, *n*-hexane/2-propanol = 90/10, *v* = 1.0 mL · min^{-1}, λ = 254 nm, t (minor) = 20.22 min, t (major) = 30.01 min]; $[\alpha]_D^{20}$ = +19.5 (c = 1.0, $CHCl_3$). ^{1}H NMR (400 MHz, $CDCl_3$) δ 2.26 (s, 3H), 2.97 (dd, *J* = 14.4, 10.8 Hz, 1H), 3.43-3.50 (m, 1H), 3.78 (s, 3H), 3.83 (dd, *J* = 14.4, 6.0 Hz, 1H), 5.24 (dd, *J* = 10.0, 1.6 Hz, 1H), 5.33 (d, *J* = 16.4 Hz, 1H), 5.73 (ddd, *J* = 17.2, 10.0, 8.8 Hz, 1H), 6.26 (s, 1H), 6.80 (dd, *J* = 8.8, 2.4 Hz, 1H), 6.95 (d, *J* = 2.4 Hz, 1H), 7.05 (d, *J* = 8.0 Hz, 2H), 7.14 (d, *J* = 8.8 Hz, 1H), 7.25-7.30 (m, 5H), 7.55 (d, *J* = 8.4 Hz, 2H), 7.75 (br s, 1H); ^{13}C NMR (100 MHz, $CDCl_3$) δ 21.3, 37.5, 44.8, 55.7, 55.9, 102.5, 111.2, 111.5, 111.7, 117.8, 126.86, 126.94, 128.4, 128.6, 129.3, 131.3, 137.5, 138.0, 138.9, 143.3, 153.8; IR (thin film): ν_{max} (cm^{-1}) = 3343, 2924, 1639, 1597, 1494, 1454, 1330, 1155, 1090, 1054, 915, 804, 740, 662; LC-ESI-MS: 459 $[M+H]^+$; HRMS (ESI) calcd for $C_{27}H_{27}N_2O_3S$ $[M+H]^+$: 459.1737; Found: 459.1725; m.p. = 180-181 ℃.

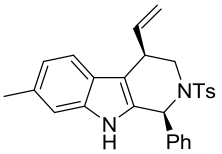

cis-**9c**

White solid, 97% yield, 97% ee [Daicel Chiralpak AD-H, *n*-hexane/2-propanol = 70/30, *v* = 1.0 mL · min^{-1}, λ = 254 nm, t (minor) = 9.79 min, t (major) = 37.31 min]; $[\alpha]_D^{20}$ = +22.8 (c = 1.0, $CHCl_3$). ^{1}H NMR (400 MHz, $CDCl_3$) δ 2.27 (s, 3H), 2.42 (s, 3H), 2.97 (dd, *J* = 14.4, 10.8 Hz, 1H), 3.42-3.49 (m, 1H), 3.83 (dd, *J* = 14.4, 6.0 Hz, 1H), 5.23 (dd, *J* = 10.0, 1.6 Hz, 1H), 5.32 (dd, *J* = 16.8, 1.6 Hz, 1H), 5.74 (ddd, *J* = 17.2, 10.0, 8.8 Hz, 1H), 6.27 (s, 1H), 6.87 (d, *J* = 7.2 Hz, 1H), 7.06 (s, 2H), 7.08 (s, 1H), 7.27-7.31 (m, 5H), 7.37 (d, *J* = 7.6 Hz, 1H), 7.57 (d, *J* = 8.4 Hz, 2H), 7.68 (br s, 1H); ^{13}C NMR (100 MHz, $CDCl_3$) δ 21.4, 21.7, 37.4, 44.7, 55.6, 110.9, 111.5, 117.8, 119.6, 121.2, 124.2, 126.8, 128.4, 128.59, 128.63, 129.4, 129.6, 132.1, 136.6, 137.5, 138.1, 138.9, 143.3; IR (thin film): ν_{max} (cm^{-1}) = 3391, 3028, 2958, 2855, 1627, 1599, 1493, 1453, 1333, 1158, 1091, 992, 921, 808, 700, 662; HRMS (ESI) calcd for $C_{27}H_{27}N_2O_2S$ $[M+H]^+$: 443.1788; Found: 443.1780; m.p. = 191-192 ℃.

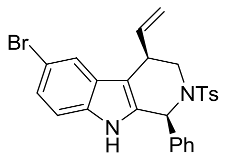

cis-**9d**

White solid, 90% yield, 92% ee [Daicel Chiralpak IC, *n*-hexane/2-propanol = 70/30, *v* = 0.4 mL·min^{-1}, λ = 254 nm, t (minor) = 11.29 min, t (major) = 12.12 min]; $[\alpha]_D^{20}$ = +11.2 (c = 0.5, $CHCl_3$). ^{1}H NMR (400 MHz, $CDCl_3$) δ 2.28 (s, 3H), 2.95 (dd, *J* = 14.8, 10.8 Hz, 1H), 3.39-3.46 (m, 1H), 3.83 (dd, *J* = 14.8, 6.4 Hz, 1H), 5.27 (dd, *J* = 10.0, 1.6 Hz, 1H), 5.33 (d, *J* = 17.2 Hz, 1H), 5.69 (ddd, *J* = 17.2, 10.0, 8.4 Hz, 1H), 6.29 (s, 1H), 7.05 (d, *J* = 8.0 Hz, 2H), 7.12 (d, *J* = 8.4 Hz, 1H), 7.21-7.26 (m, 3H), 7.29-7.33 (m, 3H), 7.54 (d, *J* = 8.4 Hz, 2H), 7.57 (d, *J* = 1.6 Hz, 1H), 7.93 (br s, 1H); ^{13}C NMR (100 MHz, $CDCl_3$) δ 21.4, 37.3, 44.6, 55.5, 111.2, 112.3, 112.8, 118.5, 122.4, 125.0, 126.8, 128.1, 128.5, 128.6, 128.7, 129.4, 131.8, 134.8, 137.3, 137.4, 138.5, 143.5; IR (thin film): ν_{max} (cm^{-1}) = 3366, 2978, 1645, 1454, 1159, 1088, 1045, 924, 878, 742, 664; LC-ESI-MS: 507 [M+H]$^+$; HRMS (MALDI) calcd for $C_{26}H_{24}N_2O_2SBr$ [M+H]$^+$: 507.0736; Found: 507.0723; m.p. = 228-229 ℃.

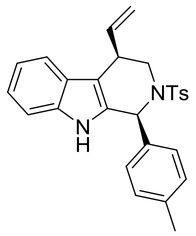

cis-**9e**

White solid, 93% yield, 96% ee [Daicel Chiralpak IC, *n*-hexane/2-propanol = 80/20, *v* = 0.8 mL·min^{-1}, λ = 254 nm, t (minor) = 6.95 min, t (major) = 8.68 min]; $[\alpha]_D^{20}$ = +39.0 (c = 0.25, $CHCl_3$). ^{1}H NMR (400 MHz, $CDCl_3$) δ 2.26 (s, 3H), 2.33 (s, 3H), 2.99 (dd, *J* = 14.4, 11.2 Hz, 1H), 3.45-3.52 (m, 1H), 3.83 (dd, *J* = 14.4, 6.4 Hz, 1H), 5.24 (dd, *J* = 10.0, 1.6 Hz, 1H), 5.33 (dd, *J* = 17.2, 1.6 Hz, 1H), 5.74 (ddd, *J* = 17.2, 10.0, 8.8 Hz, 1H), 6.26 (s, 1H), 7.01-7.18 (m, 8H), 7.25 (d, *J* = 8.0 Hz, 1H), 7.49 (d, *J* = 8.4 Hz, 1H), 7.57 (d, *J* = 8.4 Hz, 2H), 7.79 (br s, 1H); ^{13}C NMR (100 MHz, $CDCl_3$) δ 21.1, 21.4, 37.5, 44.7, 55.4, 110.9, 111.5, 117.8, 119.4, 119.9, 122.1, 126.4, 126.9, 128.5, 129.3, 130.6, 135.9, 136.1, 137.5, 138.1, 138.4, 143.3; IR (thin film): ν_{max} (cm^{-1}) = 3389, 3027, 2922, 1597, 1511, 1454,

1330, 1180, 1091, 992, 928, 751, 680, 661, 547; LC-ESI-MS: 443 [M+H]$^+$; HRMS (ESI) calcd for $C_{27}H_{26}N_2NaO_2S$ [M+Na]$^+$: 465.1607; Found: 465.1613; m.p. = 183-184 ℃.

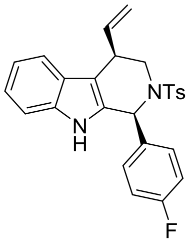

cis-**9f**

White solid, 91% yield, 94% ee [Daicel Chiralpak IC, *n*-hexane/2-propanol = 80/20, *v* = 0.6 mL·min^{-1}, λ = 254 nm, t (minor) = 8.77 min, t (major) = 9.54 min]; $[\alpha]_D^{20}$ = +31.5 (c = 0.5, $CHCl_3$). ^{1}H NMR (400 MHz, $CDCl_3$) δ 2.27 (s, 3H), 2.95 (dd, *J* = 14.4, 10.8 Hz, 1H), 3.44-3.50 (m, 1H), 3.85 (dd, *J* = 14.8, 6.0 Hz, 1H), 5.25 (dd, *J* = 9.6, 1.2 Hz, 1H), 5.33 (ddd, *J* = 17.2, 1.6, 0.8 Hz, 1H), 5.74 (ddd, *J* = 17.2, 10.0, 8.8 Hz, 1H), 6.28 (s, 1H), 6.98 (t, *J* = 8.8 Hz, 2H), 7.02-7.09 (m, 3H), 7.16 (dd, *J* = 7.2, 1.2 Hz, 1H), 7.25-7.29 (m, 3H), 7.49 (d, *J* = 8.0 Hz, 1H), 7.57 (d, *J* = 8.0 Hz, 2H), 7.80 (br s, 1H); ^{13}C NMR (75 MHz, $CDCl_3$) δ 21.4, 37.4, 44.6, 54.9, 110.9, 111.7, 115.5 (d, *J* = 21.7 Hz), 118.0, 119.6, 120.0, 122.4, 126.3, 126.8, 129.4, 130.1, 130.4 (d, *J* = 8.0 Hz), 134.7 (d, *J* = 2.9 Hz), 136.1, 137.4, 137.8, 143.5, 162.7 (d, *J* = 246.4 Hz); IR (thin film): ν_{max} (cm^{-1}) = 3390, 2924, 1603, 1507, 1338, 1225, 1159, 1091, 928, 752, 680, 592, 547; LC-ESI-MS: 447 [M+H]$^+$; HRMS (ESI) calcd for $C_{26}H_{24}FN_2O_2S$ [M+H]$^+$: 447.1537; Found: 447.1548; m.p. = 219-220 ℃.

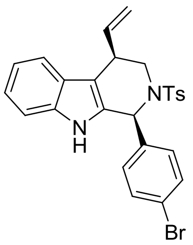

cis-**9i**

White solid, 95% yield, 93% ee [Daicel Chiralpak IC, *n*-hexane/2-propanol = 80/20, *v* = 0.6 mL·min^{-1}, λ = 254 nm, t (minor) = 10.01 min, t (major) = 10.99 min]; $[\alpha]_D^{20}$ = +40.1 (c = 0.5, $CHCl_3$). ^{1}H NMR (300 MHz, $CDCl_3$) δ 2.27 (s, 3H), 2.93 (dd, *J* = 14.7, 10.8 Hz, 1H), 3.41-3.50 (m, 1H), 3.85 (dd, *J* =

14.7, 6.3 Hz, 1H), 5.25 (d, *J* = 9.9 Hz, 1H), 5.32 (d, *J* = 17.1 Hz, 1H), 5.72 (dt, *J* = 17.1, 9.0 Hz, 1H), 6.24 (s, 1H), 7.01-7.08 (m, 3H), 7.14-7.18 (m, 3H), 7.26 (s, 1H), 7.42 (d, *J* = 8.1 Hz, 2H), 7.48 (d, *J* = 7.8 Hz, 1H), 7.56 (d, *J* = 8.4 Hz, 2H), 7.87 (br s, 1H); ^{13}C NMR (75 MHz, $CDCl_3$) δ 21.4, 37.3, 44.7, 55.0, 110.9, 111.7, 118.1, 119.6, 120.0, 122.4, 122.7, 126.3, 126.8, 129.4, 129.7, 130.2, 131.7, 136.1, 137.2, 137.8, 137.9, 143.5; IR (thin film): ν_{max} (cm^{-1}) = 3384, 2921, 2851, 1486, 1337, 1158, 1091, 1011, 929, 754, 662, 584; HRMS (MALDI) calcd for $C_{26}H_{24}N_2O_2SBr$ $[M+H]^+$: 507.0736; Found: 507.0729; m.p. = 172-173 ℃.

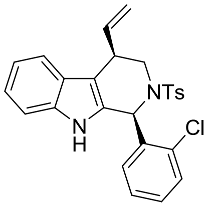

cis-**9j**

White solid, 90% yield, 99% ee [Daicel Chiralpak AD-H, *n*-hexane/2-propanol = 70/30, *v* = 1.0 mL·min^{-1}, λ = 254 nm, t (minor) = 8.00 min, t (major) = 9.07 min]; $[\alpha]_D^{20}$ = +137.1 (c = 0.5, $CHCl_3$). ^{1}H NMR (400 MHz, $CDCl_3$) δ 2.31 (s, 3H), 3.33 (dd, *J* = 14.0, 9.2 Hz, 1H), 3.69 (dd, *J* = 14.8, 8.8 Hz, 1H), 3.83 (dd, *J* = 13.6, 5.6 Hz, 1H), 5.29 (d, *J* = 10.0 Hz, 1H), 5.37 (d, *J* = 17.2 Hz, 1H), 5.89 (ddd, *J* = 17.2, 9.2, 8.4 Hz, 1H), 6.63 (s, 1H), 7.02-7.16 (m, 6H), 7.20-7.25 (m, 2H), 7.45 (d, *J* = 8.0 Hz, 1H), 7.51 (d, *J* = 8.0 Hz, 1H), 7.64 (d, *J* = 8.4 Hz, 2H), 7.87 (br s, 1H); ^{13}C NMR (100 MHz, $CDCl_3$) δ 21.4, 37.5, 46.7, 52.9, 110.9, 111.0, 117.9, 119.6, 119.7, 122.4, 126.3, 127.0, 127.4, 129.3, 129.5, 130.1, 130.2, 130.8, 133.1, 136.2, 136.4, 137.3, 138.1, 143.5; IR (thin film): ν_{max} (cm^{-1}) = 3372, 2956, 2925, 2854, 1464, 1343, 1159, 1090, 921, 813, 663; HRMS (ESI) calcd for $C_{26}H_{24}ClN_2O_2S$ $[M+H]^+$: 463.1242; Found: 463.1229; m.p. = 208-209 ℃.

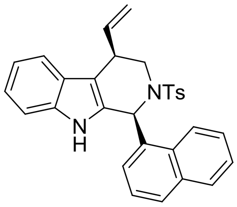

cis-**9k**

White solid, 91% yield, 96% ee [Daicel Chiralpak IC, *n*-hexane/2-propanol = 70/30, *v* = 0.6 mL·min^{-1}, λ = 254 nm, t (major) = 8.16 min, t (minor) = 9.60 min]; $[\alpha]_D^{20}$ = +7.3 (c = 0.5, $CHCl_3$). ^{1}H NMR (400 MHz, $CDCl_3$) δ 2.14 (s, 3H), 3.04 (dd, *J* = 14.4, 10.4 Hz, 1H), 3.59-3.70 (m, 2H), 5.20 (dd, *J* = 9.6, 1.6 Hz, 1H), 5.32 (ddd, *J* = 16.8, 1.6, 0.8 Hz, 1H), 5.75 (ddd, *J* = 17.2, 10.0, 8.4 Hz, 1H), 6.89 (d, *J* = 8.0 Hz, 2H), 6.99 (d, *J* = 7.2 Hz, 1H), 7.04 (dt, *J* = 8.0, 1.2 Hz, 1H), 7.10 (s, 1H), 7.15 (dt, *J* = 6.8, 1.2 Hz, 1H), 7.22-7.26 (m, 1H), 7.40 (d, *J* = 8.0 Hz, 1H), 7.53-7.58 (m, 3H), 7.65 (dt, *J* = 6.8, 1.2 Hz, 1H), 7.79 (s, 1H), 7.80 (d, *J* = 8.0 Hz, 1H), 7.89 (d, *J* = 8.0 Hz, 1H), 8.93 (d, *J* = 8.8 Hz, 1H); ^{13}C NMR (100 MHz, $CDCl_3$) δ 21.2, 36.7, 44.6, 53.1, 110.9, 111.8, 117.4, 119.5, 119.6, 122.2, 124.3, 124.6, 126.2, 126.5, 127.2, 128.2, 128.6, 128.9, 129.6, 130.7, 133.7, 134.1, 136.1, 136.4, 138.4, 143.5; IR (thin film): ν_{max} (cm^{-1}) = 3396, 3057, 2925, 1453, 1337, 1157, 1089, 914, 801, 663; LC-ESI-MS: 479 $[M+H]^+$; HRMS (MALDI) calcd for $C_{30}H_{27}N_2O_2S$ $[M+H]^+$: 479.1788; Found: 479.1787; m.p. = 211-212 ℃.

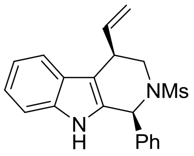

cis-**9l**

White solid, 90% yield, 94% ee [Daicel Chiralpak IC, *n*-hexane/2-propanol = 70/30, *v* = 1.0 mL·min^{-1}, λ = 254 nm, t (minor) = 5.47 min, t (major) = 7.94 min]; $[\alpha]_D^{20}$ = +10.4 (c = 1.0, $CHCl_3$). ^{1}H NMR (400 MHz, $CDCl_3$) δ 2.65 (s, 3H), 3.01 (dt, *J* = 12.4, 2.8 Hz, 1H), 3.87-3.94 (m, 2H), 5.34 (d, *J* =11.2 Hz, 1H), 5.49 (d, *J* = 17.2 Hz, 1H), 5.84 (ddd, *J* = 17.2, 10.0, 8.8 Hz, 1H), 6.13 (s, 1H), 7.11 (t, *J* = 7.2 Hz, 1H), 7.20 (d, *J* = 7.6 Hz, 1H), 7.31 (d, *J* = 8.0 Hz, 1H), 7.34 (apps, 5H), 7.67 (d, *J* = 8.0 Hz, 1H), 7.91 (br s, 1H); ^{13}C NMR (100 MHz, $CDCl_3$) δ 38.2, 39.8, 44.5, 55.6, 111.1, 111.4, 118.4, 119.8, 120.1, 122.5, 126.4, 128.7, 128.8, 130.3, 136.3, 137.7, 138.1; IR (thin film): ν_{max} (cm^{-1}) = 3391, 2924, 2853, 1492, 1330, 1150, 992, 818, 747, 700; MS (EI, *m/*z, rel. intensity) 352 ($[M]^+$, 38), 244 (100); HRMS (EI) calcd for $C_{20}H_{20}N_2O_2S$ $[M]^+$: 352.1245; Found: 352.1242; m.p. = 180-181 ℃.

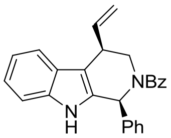

cis-**9m**

White solid, 86% yield, 93% ee [Daicel Chiralcel OD-H, *n*-hexane/2-propanol = 95/5, *v* = 0.6 mL · min^{-1}, λ = 214 nm, t (minor) = 26.96 min, t (major) = 37.21 min]; $[\alpha]_D^{20}$ = +37.6 (c = 0.5, $CHCl_3$). ^{1}H NMR (400 MHz, $CDCl_3$) δ 3.10 (t, *J* = 11.6 Hz, 1H), 3.63-3.65 (m, 1H), 3.82-3.84 (m, 1H), 5.23 (d, *J* = 9.6 Hz, 1H), 5.36 (d, *J* = 16.8 Hz, 1H), 5.79 (dt, *J* = 17.6, 9.2 Hz, 1H), 7.08 (t, *J* = 7.6 Hz, 1H), 7.16 (dt, *J* = 7.2, 0.8 Hz, 2H), 7.24-7.26 (m, 2H), 7.31-7.32 (m, 3H), 7.38-7.44 (m, 5H), 7.63 (d, *J* = 8.0 Hz, 1H), 8.50 (br s, 1H); ^{13}C NMR (75 MHz, $CDCl_3$) δ 39.9, 46.8, 52.0, 111.2, 117.7, 119.4, 119.7, 122.0, 126.4, 126.5, 128.2, 128.6, 128.8, 129.8, 131.4, 136.0, 136.5, 138.0, 139.6, 170.5; IR (thin film): ν_{max} (cm^{-1}) = 3389, 3258, 2924, 2854, 1613, 1576, 1456, 1430, 1293, 1161, 919, 742, 699; MS (EI, *m/z*, rel. intensity) 378 ([M]$^+$, 86), 105 (100); HRMS (EI) calcd for $C_{26}H_{22}N_2O$ [M]$^+$: 378.1732; Found: 378.1729. ; m.p. = 180-181 ℃.

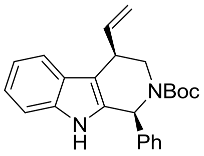

cis-**9n**

White solid, 90% yield, 97% ee [Daicel Chiralpak AD-H, *n*-hexane/2-propanol = 90/10, *v* = 1.0 mL · min^{-1}, λ = 254 nm, t (minor) = 9.69 min, t (major) = 22.60 min]; $[\alpha]_D^{20}$ = +150.0 (c = 0.2, $CHCl_3$). ^{1}H NMR (400 MHz, $CDCl_3$) δ 1.51 (s, 9H), 2.85 (dd, *J* = 13.6, 10.8 Hz, 1H), 3.84 (br s, 1H), 4.03-4.26 (m, 1H), 5.31 (dd, *J* = 10.4, 2.0 Hz, 1H), 5.49 (dd, *J* = 17.2, 1.2 Hz, 1H), 5.86-5.94 (m, 1H), 6.30-6.49 (m, 1H), 7.08 (dt, *J* = 7.2, 1.2 Hz, 1H), 7.16 (dt, *J* = 7.2, 1.2 Hz, 1H), 7.27-7.34 (m, 6H), 7.68 (d, *J* = 7.6 Hz, 1H), 7.78-7.87 (m, 1H); ^{13}C NMR (100 MHz, $CDCl_3$) δ 28.5, 39.1, 43.1, 44.1, 53.4, 54.4, 80.4, 110.9, 112.0, 115.2, 117.5, 119.4, 119.9, 121.9, 126.5, 128.0, 128.3, 128.5, 131.8, 136.3, 138.6, 140.1, 154.8; IR (thin film): ν_{max} (cm^{-1}) = 3327, 2956, 2924, 2854, 1731, 1673, 1456, 1414, 1366, 1257, 1166, 891, 764, 700; LC-ESI-MS: 375 [M+H]$^+$; HRMS (MALDI) calcd for $C_{24}H_{26}N_2NaO_2$ [M+Na]$^+$: 397.1887; Found: 397.1900; m.p. = 160-161 ℃.

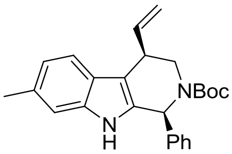

cis-**9o**

White solid, 95% yield, 92% ee [Daicel Chiralpak AD-H, *n*-hexane/2-propanol = 90/10, *v* = 1.0 mL·min^{-1}, λ = 254 nm, t (minor) = 10.08 min, t (major) = 27.58 min]; $[\alpha]_D^{20}$ = +73.3 (c = 1.0, $CHCl_3$). ^{1}H NMR (400 MHz, $CDCl_3$) δ 1.51 (s, 9H), 2.43 (s, 3H), 2.82 (dd, *J* = 13.2, 10.8 Hz, 1H), 3.81 (s, 1H), 4.03-4.22 (m, 1H), 5.29 (dd, *J* = 10.0, 2.4 Hz, 1H), 5.47 (d, *J* = 16.4 Hz, 1H), 5.84-5.93 (m, 1H), 6.25-6.48 (m, 1H), 6.91 (d, *J* = 7.2 Hz, 1H), 7.07 (s, 1H), 7.28-7.32 (m, 5H), 7.54 (d, *J* = 8.0 Hz, 1H), 7.66-7.75 (m, 1H); ^{13}C NMR (100 MHz, $CDCl_3$) δ 21.6, 21.7, 27.6, 28.5, 29.4, 29.7, 39.0, 43.1, 44.1, 53.4, 54.5, 80.3, 110.9, 112.2, 117.4, 118.3, 119.6, 121.2, 124.4, 128.1, 128.5, 131.1, 131.8, 136.8, 138.7, 140.2, 154.8; IR (thin film): ν_{max} (cm^{-1}) = 3360, 2974, 2926, 2857, 1666, 1454, 1413, 1302, 1160, 1089, 1046, 882, 764, 699; HRMS (ESI) calcd for $C_{25}H_{29}N_2O_2$ [M+H]$^+$: 389.2224; Found: 389.2217; m.p. = 190-191 ℃.

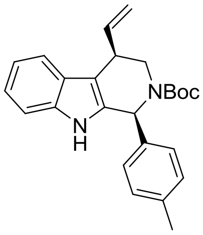

cis-**9p**

White solid, 94% yield, 93% ee [Daicel Chiralpak AD-H, *n*-hexane/2-propanol = 90/10, *v* = 1.0 mL·min^{-1}, λ = 254 nm, t (minor) = 16.40 min, t (major) = 26.82 min]; $[\alpha]_D^{20}$ = +95.8 (c = 1.0, $CHCl_3$). ^{1}H NMR (400 MHz, $CDCl_3$) δ 1.50 (s, 9H), 2.33 (s, 3H), 2.84 (dd, *J* = 13.2, 10.8 Hz, 1H), 3.82 (br s, 1H), 4.02-4.26 (m, 1H), 5.30 (dd, *J* = 10.0, 2.0 Hz, 1H), 5.48 (dd, *J* = 17.2, 1.6 Hz, 1H), 5.85-5.94 (m, 1H), 6.27-6.47 (m, 1H), 7.05-7.13 (m, 3H), 7.16 (dd, *J* = 7.2, 1.2 Hz, 1H), 7.21 (d, *J* = 8.4 Hz, 2H), 7.27 (d, *J* = 8.0 Hz, 1H), 7.67 (d, *J* = 8.0 Hz, 1H), 7.77-7.89 (m, 1H); ^{13}C NMR (100 MHz, $CDCl_3$) δ 21.1, 28.5, 39.1, 43.1, 44.0, 53.2, 54.2, 80.4, 110.9, 112.4, 117.5, 119.4, 119.6, 120.0, 121.9, 126.6, 128.3, 128.9, 129.2, 132.1, 136.3, 137.1, 137.2, 137.9, 138.7, 154.6; IR (thin film): ν_{max} (cm^{-1}) = 3306,

2974, 2925, 1667, 1454, 1414, 1366, 1284, 1162, 1047, 864, 766, 672; HRMS (ESI) calcd for $C_{25}H_{29}N_2O_2$ $[M+H]^+$: 389.2224; Found: 389.2218; m.p. = 137-138 ℃.

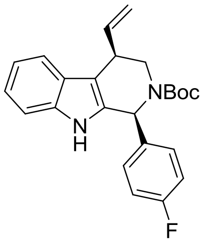

cis-**9q**

Pale yellow solid, 94% yield, 93% ee [Daicel Chiralpak AD-H, *n*-hexane/2-propanol = 90/10, *v* = 1.0 mL·min^{-1}, λ = 254 nm, t (minor) = 8.94 min, t (major) = 18.62 min]; $[\alpha]_D^{20}$ = +42.4 (c = 1.0, $CHCl_3$). ^{1}H NMR (400 MHz, $CDCl_3$) δ 1.51 (s, 9H), 2.79 (dd, *J* = 13.6, 10.8 Hz, 1H), 3.83 (br s, 1H), 4.03-4.26 (m, 1H), 5.32 (dd, *J* = 10.0, 1.6 Hz, 1H), 5.48 (dd, *J* = 17.2, 0.8 Hz, 1H), 5.84-5.93 (m, 1H), 6.27-6.48 (m, 1H), 6.98 (t, *J* = 8.4 Hz, 2H), 7.08 (dt, *J* = 8.0, 0.8 Hz, 1H), 7.16 (dt, *J* = 7.2, 0.8 Hz, 1H), 7.27-7.30 (m, 3H), 7.67 (d, *J* = 8.0 Hz, 1H), 7.79-8.06 (m, 1H); ^{13}C NMR (100 MHz, $CDCl_3$) δ 28.5, 39.0, 43.8, 53.1, 80.6, 110.9, 115.4 (d, *J* = 21.6 Hz), 117.6, 119.6, 120.1, 122.1, 126.6, 126.7, 130.1, 131.6, 136.01, 136.04, 136.4, 138.5, 162.5 (d, *J* = 246.1 Hz); IR (thin film): ν_{max} (cm^{-1}) = 3316, 2955, 2925, 2853, 1671, 1507, 1455, 1415, 1225, 1157, 1015, 893, 744; LC-ESI-MS: 393 $[M+H]^+$; HRMS (ESI) calcd for $C_{24}H_{26}FN_2O_2$ $[M+H]^+$: 393.1973; Found: 393.1986; m.p. = 158-159 ℃.

General Procedure for the One-Pot Dearomatization/Migration Process

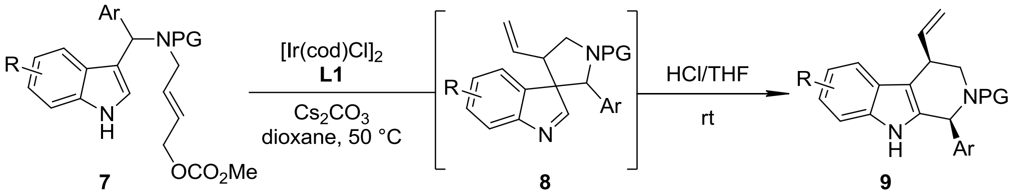

A flame-dried Schlenk tube was cooled to room temperature and filled with argon. To this flask were added $[Ir(cod)Cl]_2$ (2.7 mg, 0.004 mmol, 2 mol%), phosphoramidite ligand **L1** (4.2 mg, 0.008 mmol, 4 mol%), THF (0.5 mL) and *n*-propylamine (0.5 mL). The reaction mixture was heated at 50 ℃ for 30 min and then the volatile solvents were removed *in vacuo* to give a pale yellow solid. After that, allylic carbonate **7** (0.20 mmol, dissolved in 2.0 mL dioxane) and cesium carbonate (130.3 mg, 0.40 mmol, 200 mol%) were added. The reaction mixture was heated at 50 ℃. After the reaction was complete (monitored by TLC), the crude reaction mixture was filtrated with celite and washed with EtOAc. The solvents were removed under reduced pressure to give the desired dearomatized intermediate **8**, which has three diastereoisomers and was subjected to the migration process.

HCl/THF (4.0 mL, 4.5 mol/L) was added to the dearomatized intermediate **8**. The reaction mixture was stirred at room temperature. After the reaction was complete (monitored by TLC), the reaction mixture was quenched with saturated aqueous $NaHCO_3$, then diluted with water and EtOAc. The organic layer was separated and washed sequentially with water and brine, and dried over Na_2SO_4. The solvents were removed under reduced pressure. Then the residue was purified by silica gel column chromatography (PE/EA = 5/1) to afford the desired product **9**.

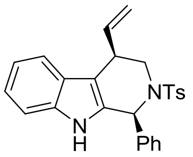

cis-**9a**

For characterization data of *cis*-**9a**, please see Page S40

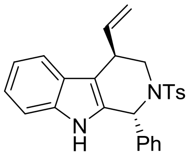

trans-**9a**

White solid. ¹H NMR (400 MHz, $CDCl_3$) δ 2.32 (s, 3H), 3.59 (dd, *J* = 13.6, 4.0 Hz, 1H), 3.73-3.76 (m, 1H), 3.89 (d, *J* = 14.0 Hz, 1H), 4.98 (d, *J* = 10.0 Hz, 1H), 5.08 (d, *J* = 16.8 Hz, 1H), 5.82 (ddd, *J* = 17.2, 10.0, 7.2 Hz, 1H), 6.24 (s, 1H), 7.06 (d, *J* = 8.8 Hz, 2H), 7.10 (dt, *J* = 8.0, 0.8 Hz, 1H), 7.16 (t, *J* = 8.0 Hz, 1H), 7.21-7.27 (m, 6H), 7.39 (d, *J* = 8.0 Hz, 2H), 7.50 (d, *J* = 7.6 Hz, 1H), 7.64 (br s, 1H); ¹³C NMR (100 MHz, $CDCl_3$) δ 21.4, 37.4, 45.5, 56.1, 110.9, 111.1, 115.6, 118.8, 119.8, 122.4, 126.3, 127.2, 128.6, 128.8, 129.1, 131.4, 136.3, 137.7, 138.5, 142.8; IR (thin film): ν_{max} (cm^{-1}) = 3014, 2900, 1777, 1564, 1340, 1217, 994, 862, 761, 666; HRMS (ESI) calcd for $C_{26}H_{24}N_2O_2S$ $[M]^+$: 428.1558; Found: 428.1549; m.p. = 222-223 ℃.

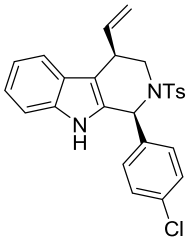

cis-**9g**

White solid, 68% yield, 95% ee [Daicel Chiralpak IC, *n*-hexane/2-propanol = 80/20, *v* = 0.8 mL·min^{-1}, λ = 254 nm, t (minor) = 6.28 min, t (major) = 6.71 min]; $[\alpha]_D^{20}$ = +18.0 (c = 0.3, $CHCl_3$). ¹H NMR (400 MHz, $CDCl_3$) δ 2.22 (s, 3H), 2.90 (dd, *J* = 14.4, 10.8 Hz, 1H), 3.39-3.45 (m, 1H), 3.83 (dd, *J* = 14.8, 6.4 Hz, 1H), 5.23 (d, *J* = 10.0 Hz, 1H), 5.30 (d, *J* = 17.2 Hz, 1H), 5.70 (dt, *J* = 16.8, 8.4 Hz, 1H), 6.25 (s, 1H), 7.00-7.03 (m, 3H), 7.12 (t, *J* = 7.2 Hz, 1H), 7.19-7.25 (m, 5H), 7.45 (d, *J* = 8.0 Hz, 1H), 7.51 (d, *J* = 8.0 Hz, 2H), 8.16 (br s, 1H); ¹³C NMR (100 MHz, $CDCl_3$) δ 21.3, 37.2, 44.7, 55.0, 111.0, 111.3, 117.9, 119.4, 119.8, 122.2, 126.2, 126.6, 128.7, 129.4, 129.78, 129.84, 134.3, 136.2, 137.0, 137.4, 137.8, 143.5; IR (thin film): ν_{max} (cm^{-1}) = 3016, 2928, 2856, 1464, 1217, 1159, 994, 929, 764, 668; HRMS (ESI) calcd for $C_{26}H_{24}ClN_2O_2S$ $[M+H]^+$: 463.1242; Found: 463.1229; m.p. = 179-180 ℃.

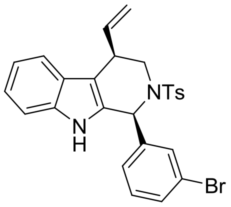

cis-**9h**

White solid, 64% yield, 94% ee [Daicel Chiralpak AD-H, *n*-hexane/2-propanol = 80/20, *v* = 1.0 mL·min^{-1}, λ = 254 nm, t (minor) = 7.49 min, t (major) = 16.65 min]; $[\alpha]_D^{20}$ = +28.6 (c = 1.0, $CHCl_3$). ^{1}H NMR (400 MHz, $CDCl_3$) δ 2.23 (s, 3H), 2.93 (dd, *J* = 14.4, 11.2 Hz, 1H), 3.42-3.48 (m, 1H), 3.87 (dd, *J* = 14.4, 5.6 Hz, 1H), 5.24 (d, *J* = 10.0 Hz, 1H), 5.32 (d, *J* = 17.2 Hz, 1H), 5.72 (dt, *J* = 17.2, 8.8 Hz, 1H), 6.22 (s, 1H), 7.02 (appd, *J* = 7.2 Hz, 3H), 7.13 (appt, *J* = 8.0 Hz, 2H), 7.21 (d, *J* = 7.6 Hz, 1H), 7.25 (d, *J* = 8.4 Hz, 1H), 7.35 (s, 1H), 7.40 (d, *J* = 8.0 Hz, 1H), 7.45 (d, *J* = 7.6 Hz, 1H), 7.52 (d, *J* = 7.2 Hz, 2H), 8.11 (br s, 1H); ^{13}C NMR (100 MHz, $CDCl_3$) δ 21.3, 37.3, 44.8, 55.2, 111.1, 111.5, 118.0, 119.4, 119.8, 122.3, 122.7, 126.2, 126.7, 127.1, 129.4, 129.5, 130.1, 131.3, 131.5, 136.2, 137.0, 137.8, 141.1, 143.5; IR (thin film): ν_{max} (cm^{-1}) = 3387, 2946, 1594, 1462, 1424, 1337, 1217, 1157, 996, 929, 763, 664; HRMS (ESI) calcd for $C_{26}H_{24}BrN_2O_2S$ $[M+H]^+$: 507.0736; Found: 507.0723; m.p. = 171-172 ℃.

X-ray data and structure refinement

X-ray data and structure refinement for 8aA

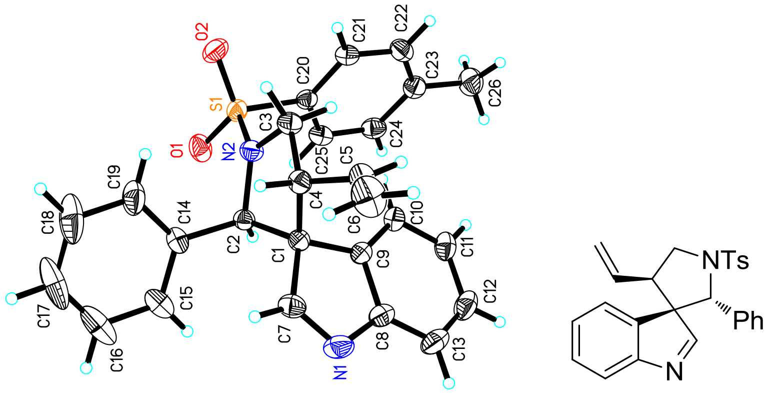

8aA

Table S4. Crystal data and structure refinement for cd211173.

CCDC No.	1018536	
Identification code	cd211173	
Empirical formula	C26 H24 N2 O2 S	
Formula weight	428.53	
Temperature	293(2) K	
Wavelength	0.71073 A	
Crystal system, space group	Orthorhombic, P2(1)2(1)2(1)	
Unit cell dimensions	a = 10.3771(7) A	alpha = 90 deg.
	b = 13.7178(9) A	beta = 90 deg.
	c = 15.7246(10) A	gamma = 90 deg.
Volume	2238.4(3) A^3	
Z, Calculated density	4, 1.272 Mg/m^3	
Absorption coefficient	0.170 mm^-1	
F(000)	904	
Crystal size	0.369 x 0.311 x 0.257 mm	
Theta range for data collection	1.97 to 26.00 deg.	
Limiting indices	-12<=h<=12, -16<=k<=16, -14<=l<=19	
Reflections collected / unique	12247 / 4376 [R(int) = 0.0248]	
Completeness to theta = 26.00	99.9 %	
Absorption correction	Empirical	
Max. and min. Transmission	1.00000 and 0.79637	
Refinement method	Full-matrix least-squares on F^2	

Data / restraints / parameters	4376 / 23 / 281
Goodness-of-fit on F^2	1.051
Final R indices [I>2sigma(I)]	R1 = 0.0369, wR2 = 0.0955
R indices (all data)	R1 = 0.0390, wR2 = 0.0970
Absolute structure parameter	0.02(6)
Largest diff. peak and hole	0.181 and -0.211 e.A^-3

X-ray data and structure refinement for 8aB

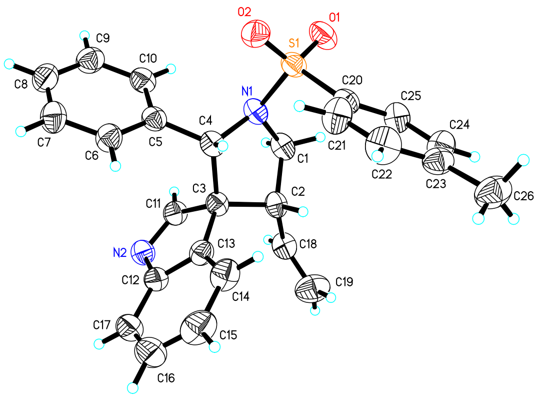

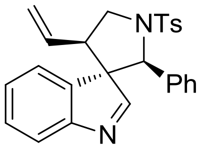

8aB

Table S5. Crystal data and structure refinement for cd211368.

CCDC No.	1018537	
Identification code	cd211368	
Empirical formula	C26 H24 N2 O2 S	
Formula weight	428.53	
Temperature	293(2) K	
Wavelength	0.71073 A	
Crystal system, space group	Monoclinic, P2(1)	
Unit cell dimensions	a = 10.6807(10) A	alpha = 90 deg.
	b = 8.6932(8) A	beta = 101.848(2) deg.
	c = 12.7653(12) A	gamma = 90 deg.
Volume	1160.00(19) A^3	
Z, Calculated density	2, 1.227 Mg/m^3	
Absorption coefficient	0.164 mm^-1	
F(000)	452	
Crystal size	0.318 x 0.256 x 0.217 mm	
Theta range for data collection	1.95 to 26.00 deg.	
Limiting indices	-13<=h<=9, -9<=k<=10, -15<=l<=15	
Reflections collected / unique	6342 / 3925 [R(int) = 0.0190]	
Completeness to theta = 26.00	99.7 %	
Absorption correction	Empirical	
Max. and min. Transmission	1.00000 and 0.71569	
Refinement method	Full-matrix least-squares on F^2	
Data / restraints / parameters	3925 / 2 / 281	
Goodness-of-fit on F^2	1.046	
Final R indices [I>2sigma(I)]	R1 = 0.0399, wR2 = 0.1007	
R indices (all data)	R1 = 0.0475, wR2 = 0.1064	

Absolute structure parameter	0.18(7)
Largest diff. peak and hole	0.172 and -0.132 e.A^-3

X-ray data and structure refinement for 8aC

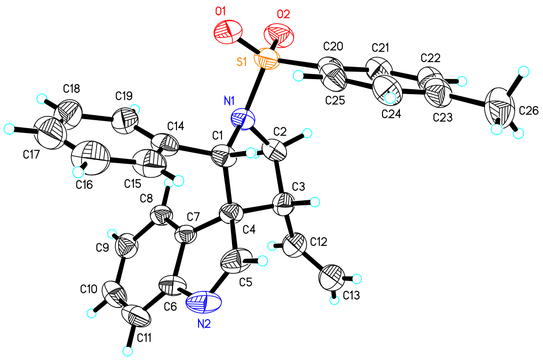

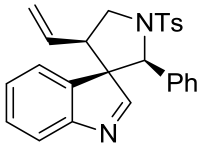

8aC

Table S6. Crystal data and structure refinement for cd211213.

CCDC No.	1018538	
Identification code	cd211213	
Empirical formula	C26 H25 N2 O2.50 S	
Formula weight	437.54	
Temperature	293(2) K	
Wavelength	0.71073 A	
Crystal system, space group	Orthorhombic, P2(1)2(1)2(1)	
Unit cell dimensions	a = 6.7630(19) A	alpha = 90 deg.
	b = 11.797(3) A	beta = 90 deg.
	c = 28.909(8) A	gamma = 90 deg.
Volume	2306.4(11) A^3	
Z, Calculated density	4, 1.260 Mg/m^3	
Absorption coefficient	0.168 mm^-1	
F(000)	924	
Crystal size	0.295 x 0.231 x 0.106 mm	
Theta range for data collection	1.86 to 25.50 deg.	
Limiting indices	-7<=h<=8, -14<=k<=13, -30<=l<=35	
Reflections collected / unique	12135 / 4279 [R(int) = 0.0351]	
Completeness to theta = 25.50	99.9 %	
Absorption correction	Empirical	
Max. and min. Transmission	1.00000 and 0.57216	
Refinement method	Full-matrix least-squares on F^2	
Data / restraints / parameters	4279 / 3 / 289	
Goodness-of-fit on F^2	0.959	
Final R indices [I>2sigma(I)]	R1 = 0.0446, wR2 = 0.1000	
R indices (all data)	R1 = 0.0584, wR2 = 0.1057	

Absolute structure parameter	-0.08(8)
argest diff. peak and hole	0.184 and -0.252 e.A^-3

X-ray data and structure refinement for cis-9a

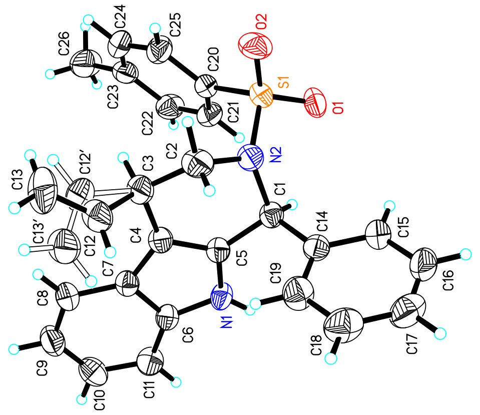

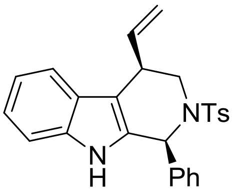

cis-**9a**

Table S7. Crystal data and structure refinement for cd211163.

CCDC No.	1018540	
Identification code	cd211163	
Empirical formula	C26 H24 N2 O2 S	
Formula weight	428.53	
Temperature	293(2) K	
Wavelength	0.71073 A	
Crystal system, space group	Orthorhombic, P2(1)2(1)2(1)	
Unit cell dimensions	a = 11.8055(8) A	alpha = 90 deg.
	b = 13.0054(9) A	beta = 90 deg.
	c = 14.4044(10) A	gamma = 90 deg.
Volume	2211.6(3) A^3	
Z, Calculated density	4, 1.287 Mg/m^3	
Absorption coefficient	0.172 mm^-1	
F(000)	904	
Crystal size	0.318 x 0.269 x 0.213 mm	
Theta range for data collection	2.11 to 26.00 deg.	
Limiting indices	-14<=h<=13, -16<=k<=15, -17<=l<=11	
Reflections collected / unique	12053 / 4345 [R(int) = 0.0172]	
Completeness to theta = 26.00,	100.0 %	
Absorption correction	Empirical	
Max. and min. Transmission	1.00000 and 0.83703	
Refinement method	Full-matrix least-squares on F^2	
Data / restraints / parameters	4345 / 24 / 307	
Goodness-of-fit on F^2	1.042	
Final R indices [I>2sigma(I)]	R1 = 0.0394, wR2 = 0.0994	

R indices (all data)	R1 = 0.0446, wR2 = 0.1036
Absolute structure parameter	0.05(7)
Largest diff. peak and hole	0.179 and -0.126 e.A^-3

X-ray data and structure refinement for trans-9a

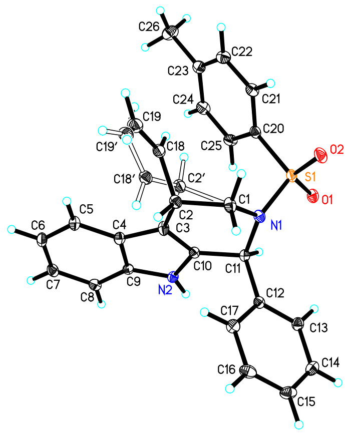

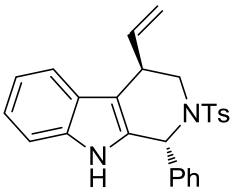

***trans*-9a**

Table S8. Crystal data and structure refinement for mo_dm12144_0m.

CCDC No.	1018541	
Identification code	mo_dm12144_0m	
Empirical formula	C26 H24 N2 O2 S	
Formula weight	428.53	
Temperature	133(2) K	
Wavelength	0.71073 A	
Crystal system, space group	Monoclinic, P2(1)/c	
Unit cell dimensions	a = 12.3031(16) A	alpha = 90 deg.
	b = 10.1584(13) A	beta = 95.303(2) deg.
	c = 17.270(2) A	gamma = 90 deg.
Volume	2149.2(5) A^3	
Z, Calculated density	4, 1.324 Mg/m^3	
Absorption coefficient	0.177 mm^-1	
F(000)	904	
Crystal size	0.30 x 0.15 x 0.04 mm	
Theta range for data collection	2.33 to 27.00 deg.	
Limiting indices	-15<=h<=15, -12<=k<=12, -20<=l<=22	
Reflections collected / unique	15834 / 4676 [R(int) = 0.0411]	
Completeness to theta = 27.00	99.9 %	
Absorption correction	Semi-empirical from equivalents	
Max. and min. Transmission	0.9930 and 0.9489	
Refinement method	Full-matrix least-squares on F^2	
Data / restraints / parameters	4676 / 74 / 308	
Goodness-of-fit on F^2	0.951	
Final R indices [I>2sigma(I)]	R1 = 0.0493, wR2 = 0.1068	

R indices (all data)	R1 = 0.0672, wR2 = 0.1151
Largest diff. peak and hole	0.347 and -0.392 e.A^-3

X-ray data and structure refinement for cis-9l

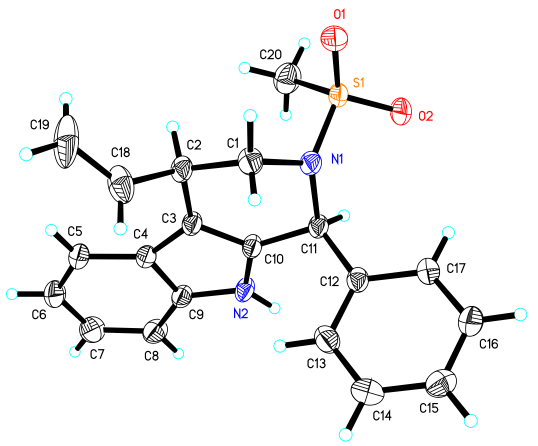

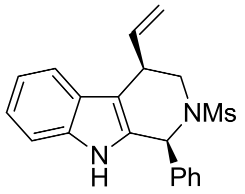

***cis*-9l**

Table S9. Crystal data and structure refinement for mo_dm11493_0m.

CCDC No.	1018539	
Identification code	mo_dm11493_0m	
Empirical formula	C20 H20 N2 O2 S	
Formula weight	352.44	
Temperature	296(2) K	
Wavelength	0.71073 A	
Crystal system, space group	Orthorhombic, P2(1)2(1)2(1)	
Unit cell dimensions	a = 8.4047(7) A	alpha = 90 deg.
	b = 8.8853(8) A	beta = 90 deg.
	c = 24.000(2) A	gamma = 90 deg.
Volume	1792.3(3) A^3	
Z, Calculated density	4, 1.306 Mg/m^3	
Absorption coefficient	0.196 mm^-1	
F(000)	744	
Crystal size	0.25 x 0.20 x 0.15 mm	
Theta range for data collection	1.70 to 28.99 deg.	
Limiting indices	-11<=h<=10, -12<=k<=12, -32<=l<=32	
Reflections collected / unique	16132 / 4755 [R(int) = 0.0274]	
Completeness to theta = 28.99	99.7 %	
Absorption correction	Semi-empirical from equivalents	
Max. and min. Transmission	0.9712 and 0.9526	
Refinement method	Full-matrix least-squares on F^2	
Data / restraints / parameters	4755 / 0 / 227	
Goodness-of-fit on F^2	1.049	
Final R indices [I>2sigma(I)]	R1 = 0.0373, wR2 = 0.0967	

R indices (all data)	R1 = 0.0463, wR2 = 0.1019
Absolute structure parameter	0.07(6)
Largest diff. peak and hole	0.236 and -0.253 e.A^-3

NMR Spectra and HPLC traces

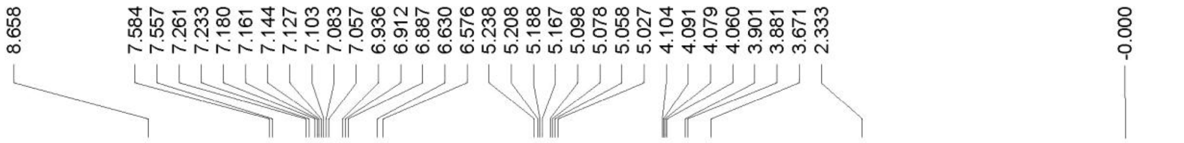

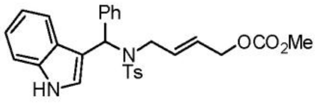

7a

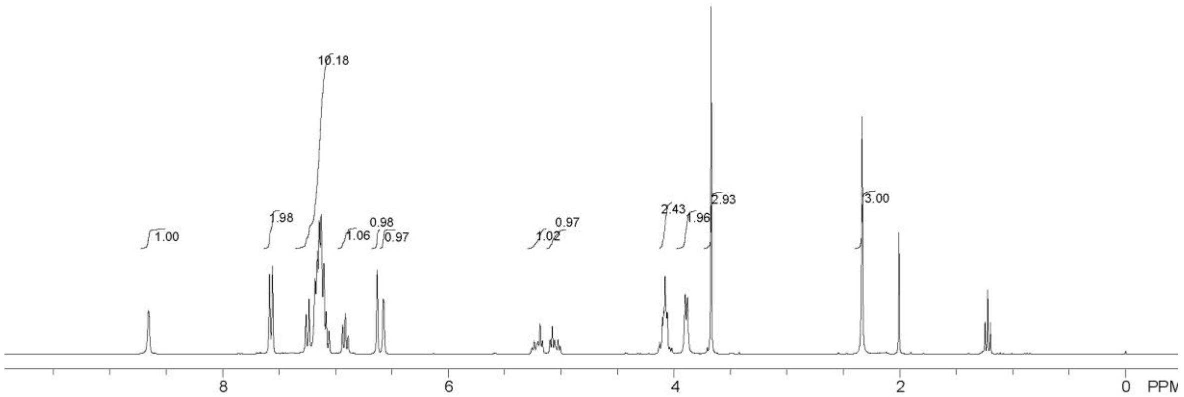

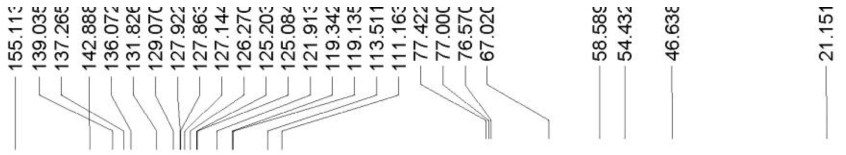

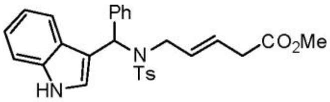

7a

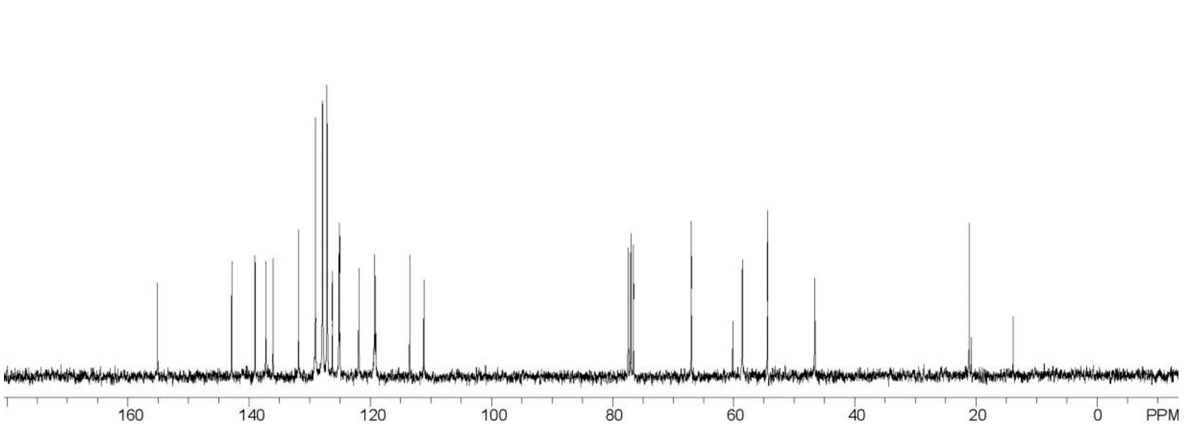

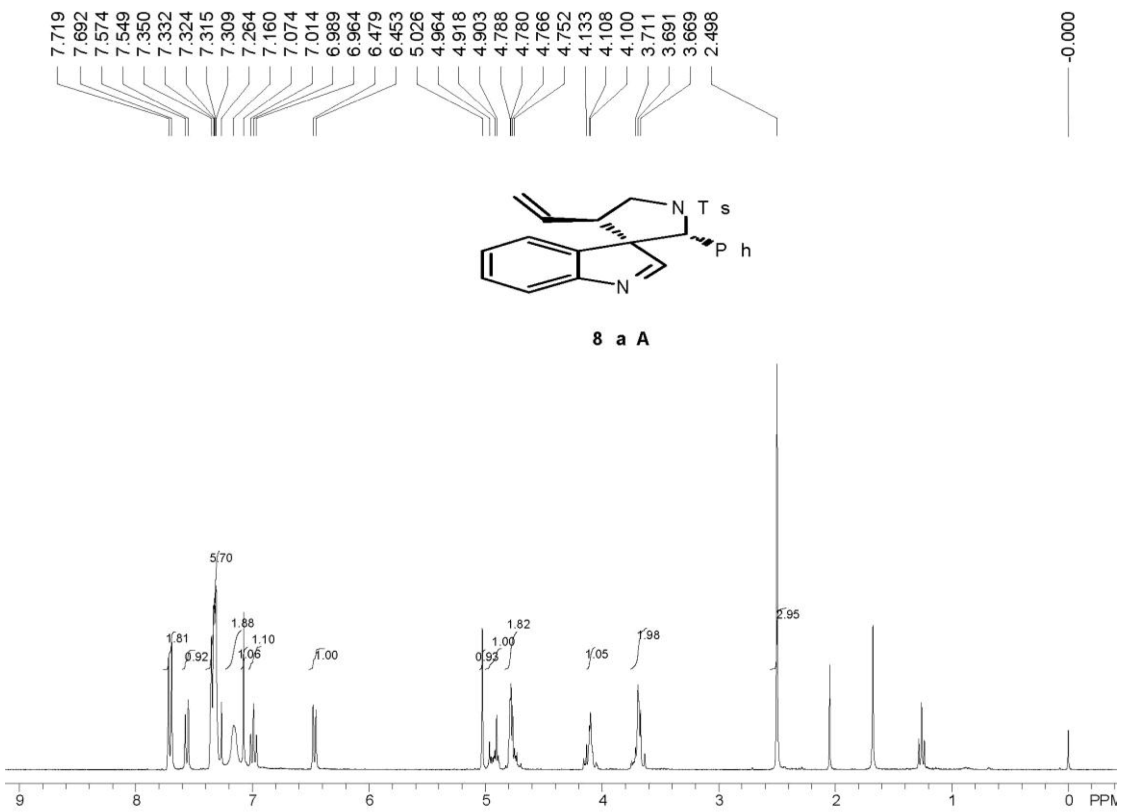
N Ts
P h
8 a A
PPM

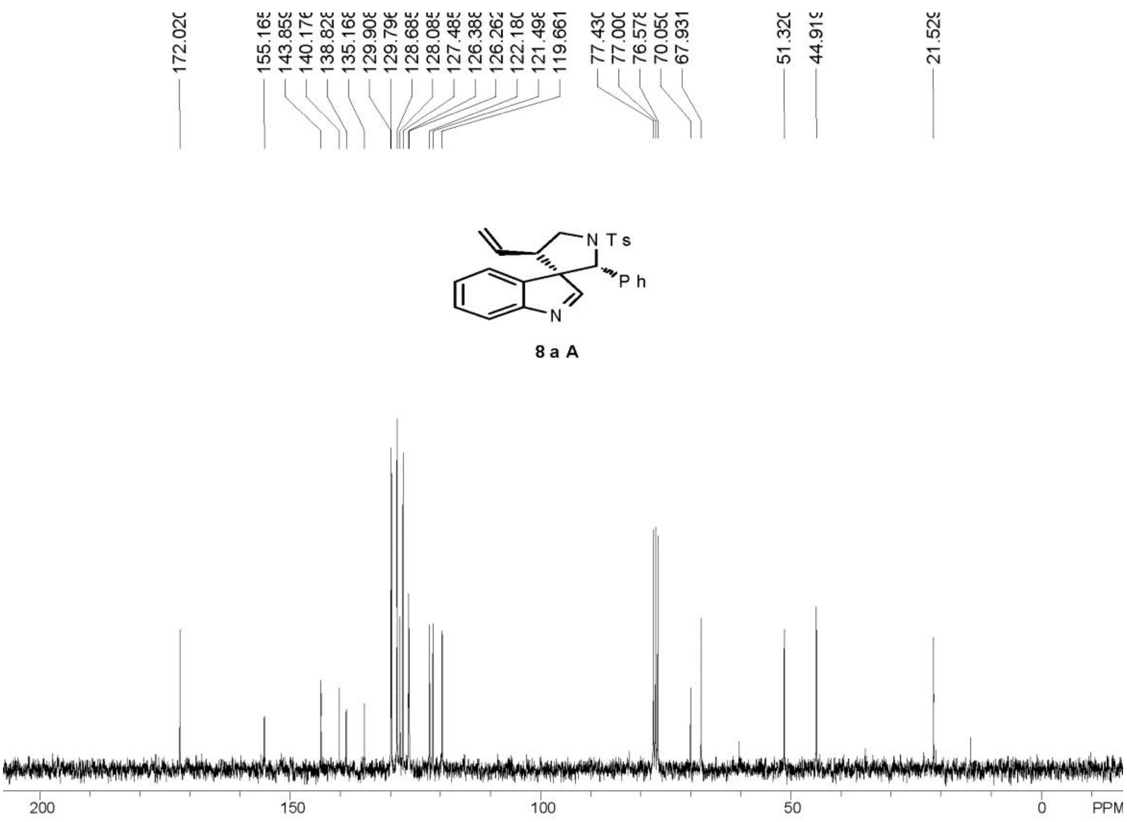
N Ts
Ph
8 a A
PPM

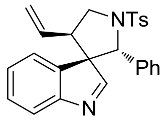

8aA

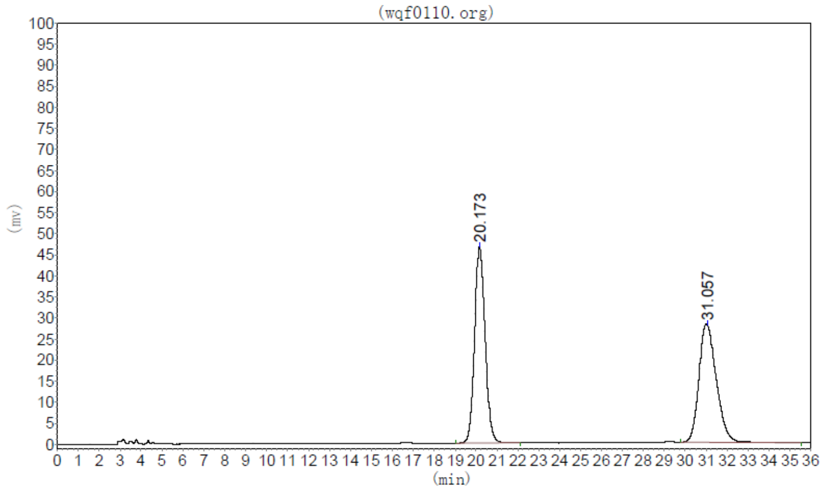

Peak No.	R. Time	Peak Height	Peak Area	Percent
1	20.173	46593.820	1631733.625	49.9968
2	31.057	28125.666	1631939.375	50.0032
Total		74719.486	3263673.000	100.0000

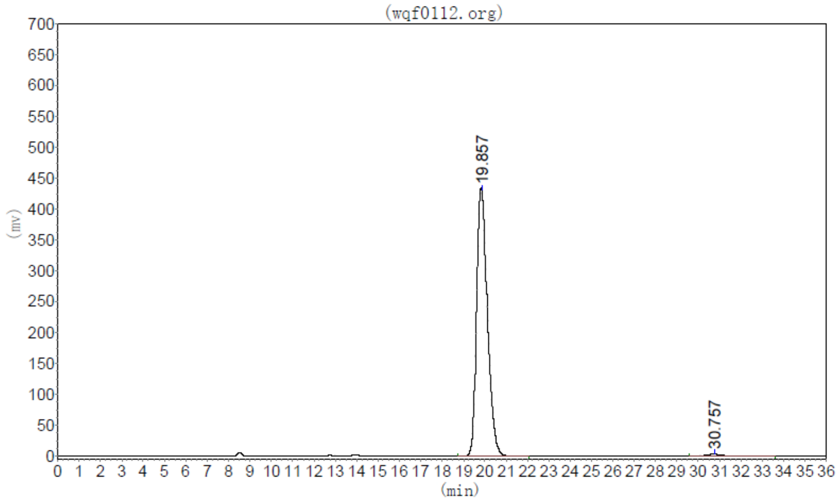

Peak No.	R. Time	Peak Height	Peak Area	Percent
1	19.857	433772.469	15212509.000	98.7944
2	30.757	3435.000	185642.094	1.2056
Total		437207.469	15398151.094	100.0000

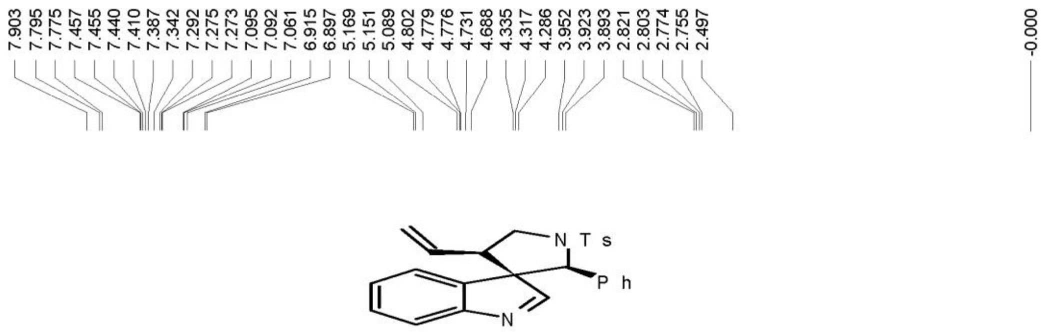
7.903 7.795 7.775 7.457 7.455 7.440 7.410 7.387 7.342 7.292 7.275 7.273 7.095 7.092 7.061 6.915 6.897 5.169 5.151 5.089 4.802 4.779 4.776 4.731 4.688 4.335 4.317 4.286 3.952 3.923 3.893 2.821 2.803 2.774 2.755 2.497
-0.000
N Ts
P h
N

8 a B

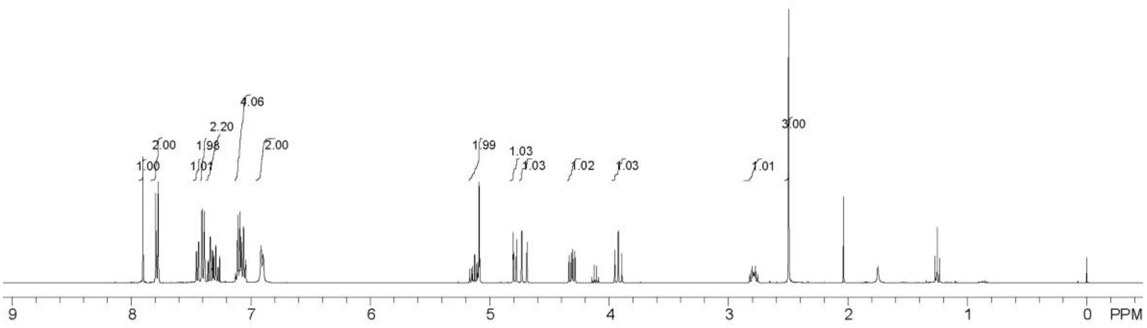
1.00 2.00 1.01 1.98 2.20 4.06 2.00 1.99 1.03 1.03 1.02 1.03 1.01 3.00
9 8 7 6 5 4 3 2 1 0 PPM

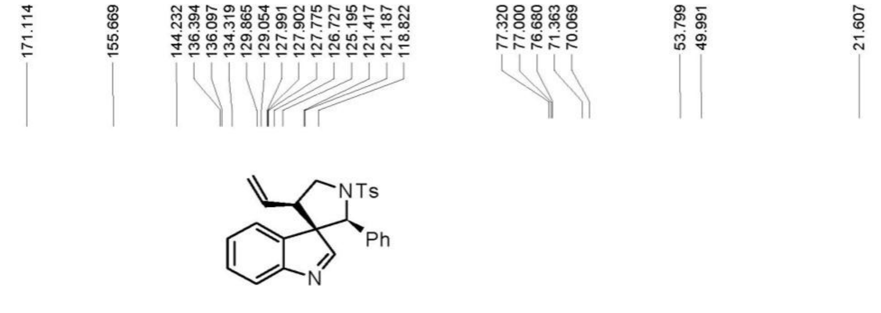
171.114
155.669
144.232 136.394 136.097 134.319 129.865 129.054 127.991 127.902 127.775 126.727 125.195 121.417 121.187 118.822
77.320 77.000 76.680 71.363 70.069
53.799 49.991
21.607
NTs
Ph
N

8aB

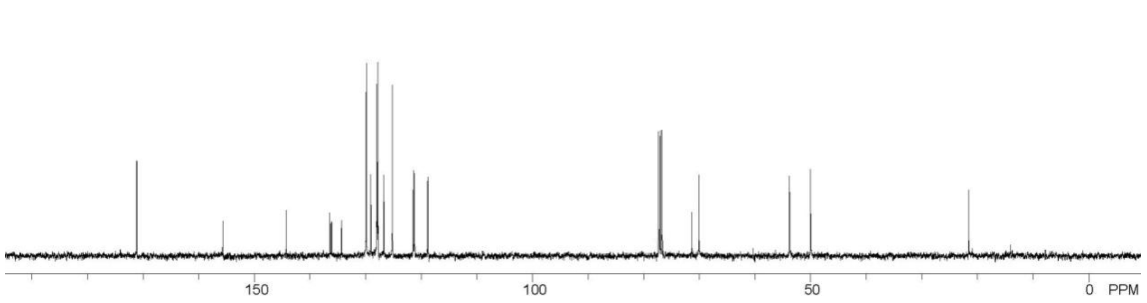
150 100 50 0 PPM

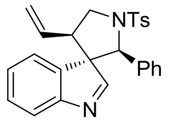

8aB

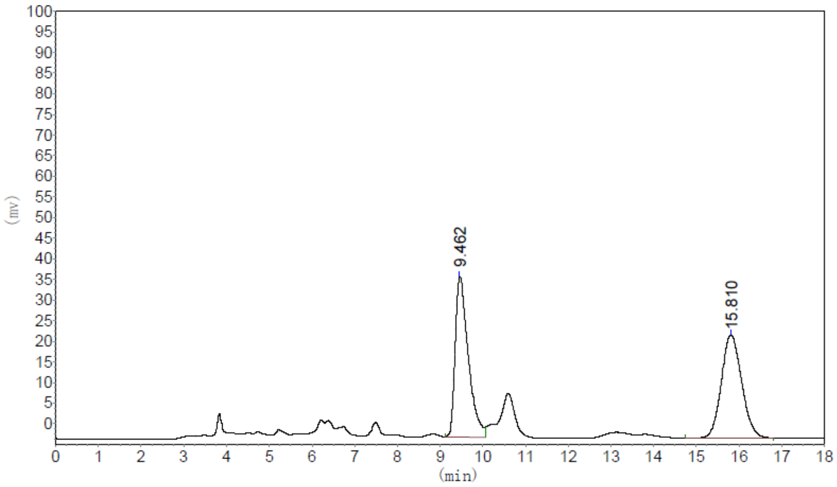

Peak No.	R. Time	Peak Height	Peak Area	Percent
1	9. 462	38881. 449	824605. 563	50. 0749
2	15. 810	24970. 795	822138. 063	49. 9251
Total		63852. 244	1646743. 625	100. 0000

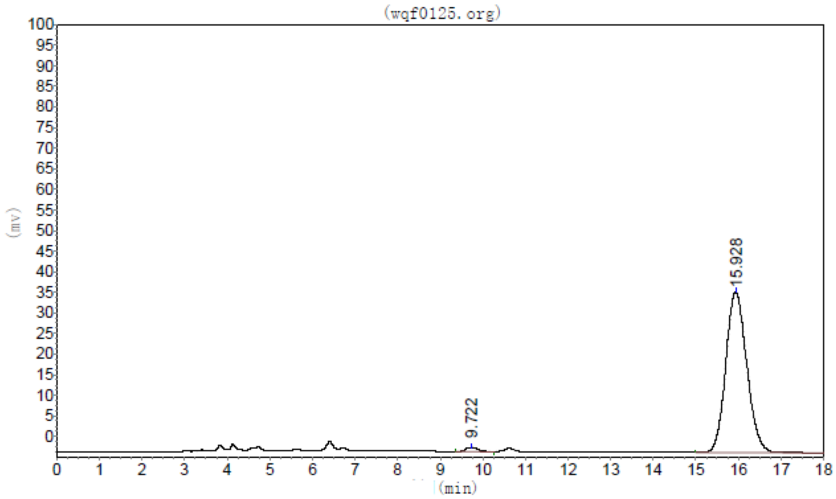

Peak No.	R. Time	Peak Height	Peak Area	Percent
1	9. 722	1037. 748	22635. 838	1. 6579
2	15. 928	39011. 168	1342696. 250	98. 3421
Total		40048. 916	1365332. 088	100. 0000

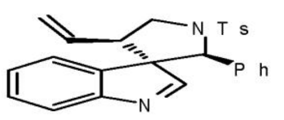

8 a C

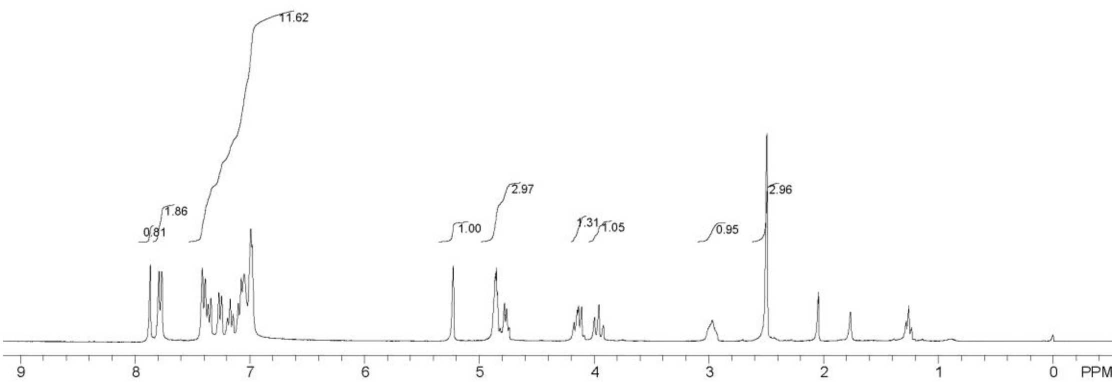
7.866
7.792
7.770
7.413
7.389
7.339
7.245
7.195
7.170
7.145
7.050
6.992
6.979
5.227
4.874
4.865
4.850
4.762
4.744
4.740
4.174
4.135
4.108
3.995
3.957
3.918
2.987
2.971
2.494
-0.000
11.62
1.86
0.81
2.97
1.00
1.31
1.05
0.95
2.96
9
8
7
6
5
4
3
2
1
0
PPM

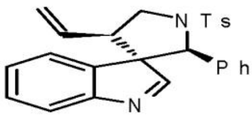

8 a C

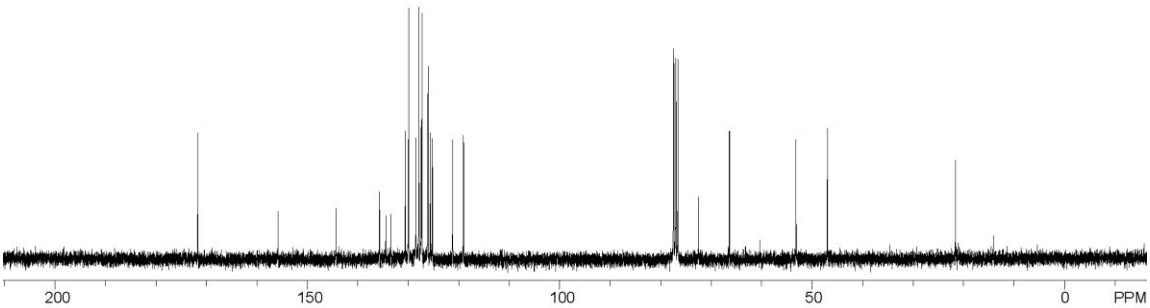
171.701
155.831
135.723
144.340
134.471
133.449
130.604
129.952
128.488
127.907
127.455
127.314
126.106
125.654
125.247
121.209
119.068
77.430
77.000
76.578
72.529
66.405
53.210
47.001
21.633
200
150
100
50
0
PPM

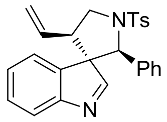

8aC

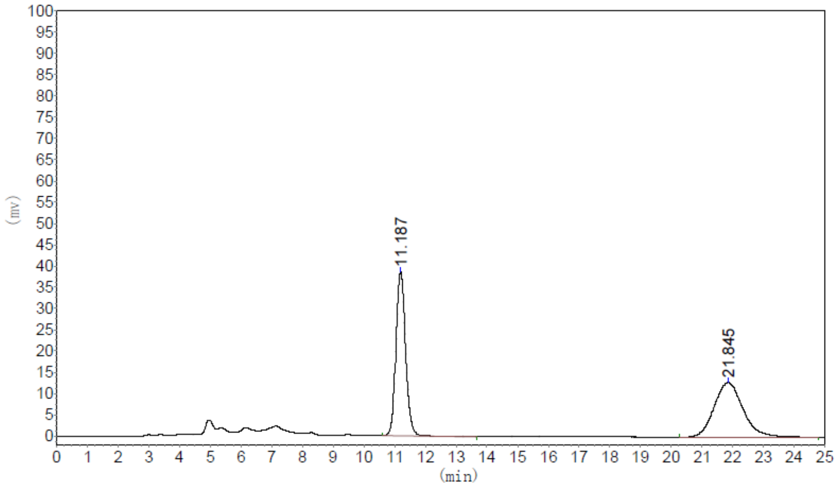

Peak No.	R. Time	Peak Height	Peak Area	Percent
1	11. 187	38456. 059	838528. 875	50. 1259
2	21. 845	12826. 574	834315. 313	49. 8741
Total		51282. 633	1672844. 188	100. 0000

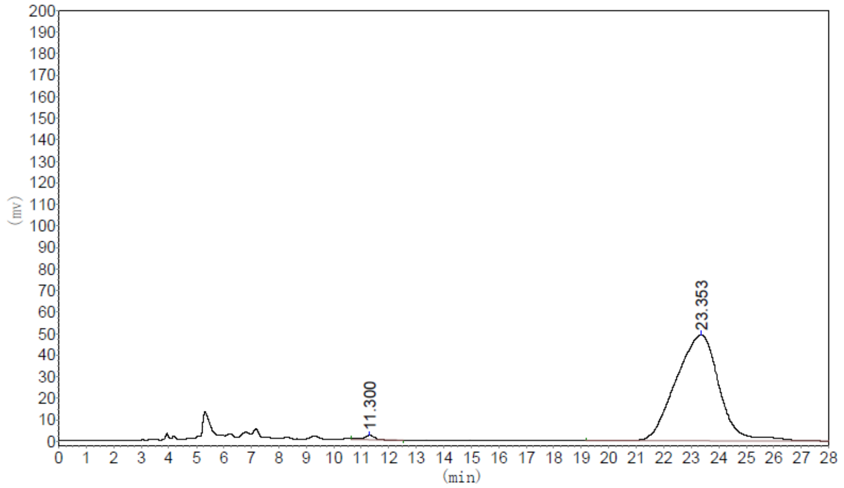

Peak No.	R. Time	Peak Height	Peak Area	Percent
1	11. 300	2069. 493	65054. 617	1. 1996
2	23. 353	49098. 984	5358090. 000	98. 8004
Total		51168. 477	5423144. 617	100. 0000

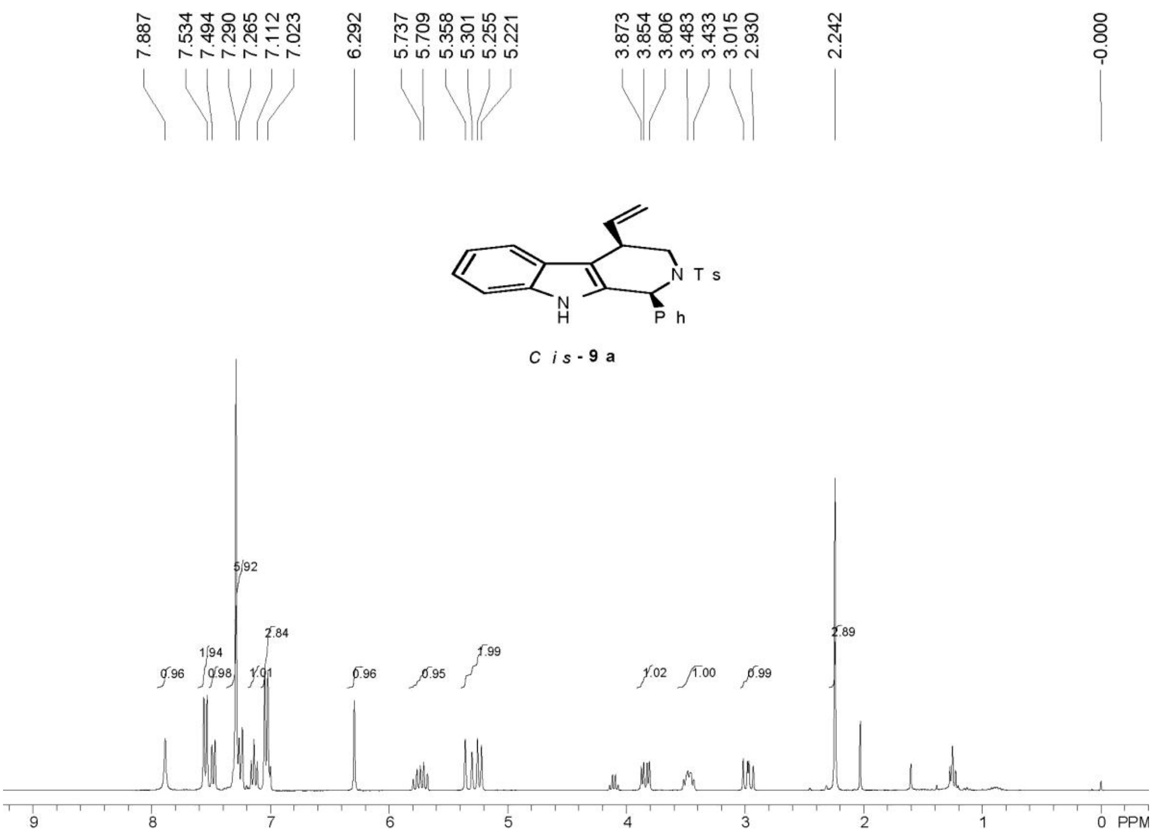
7.887
7.534
7.494
7.290
7.265
7.112
7.023
6.292
5.737
5.709
5.358
5.301
5.255
5.221
3.873
3.854
3.806
3.483
3.433
3.015
2.930
2.242
-0.000
NTs
N
H
Ph
Cis-9a
0.96
1.94
0.98
5.92
1.01
2.84
0.96
0.95
1.99
1.02
1.00
0.99
2.89
9
8
7
6
5
4
3
2
1
0 PPM

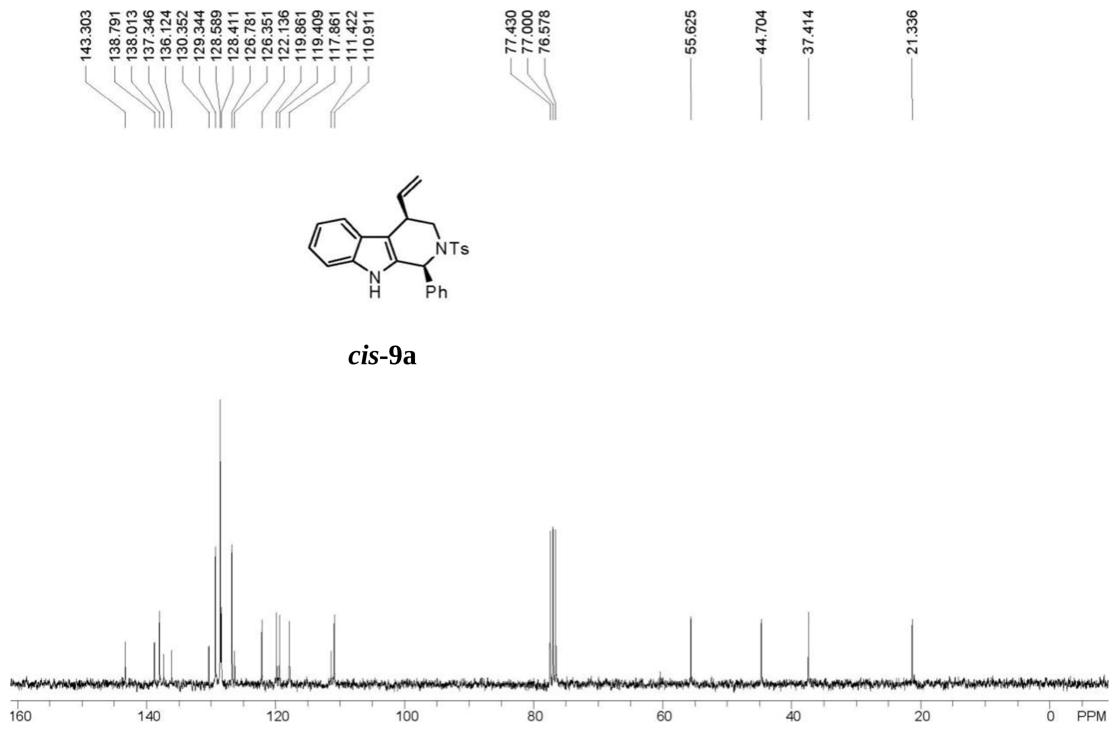
143.303
138.791
138.013
137.346
136.124
130.352
129.344
128.589
128.411
126.781
126.351
122.136
119.861
119.409
117.861
111.422
110.911
77.430
77.000
76.578
55.625
44.704
37.414
21.336
NTs
N
H
Ph
cis-9a
160
140
120
100
80
60
40
20
0
PPM

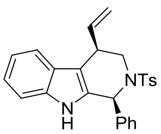

***cis*-9a**

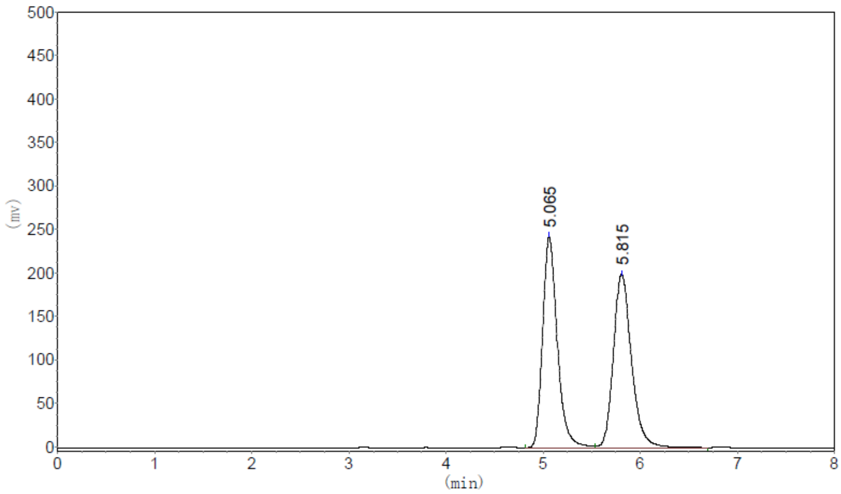

Peak No.	R. Time	Peak Height	Peak Area	Percent
1	5.065	242348.844	2547116.250	49.5569
2	5.815	199218.109	2592668.250	50.4431
Total		441566.953	5139784.500	100.0000

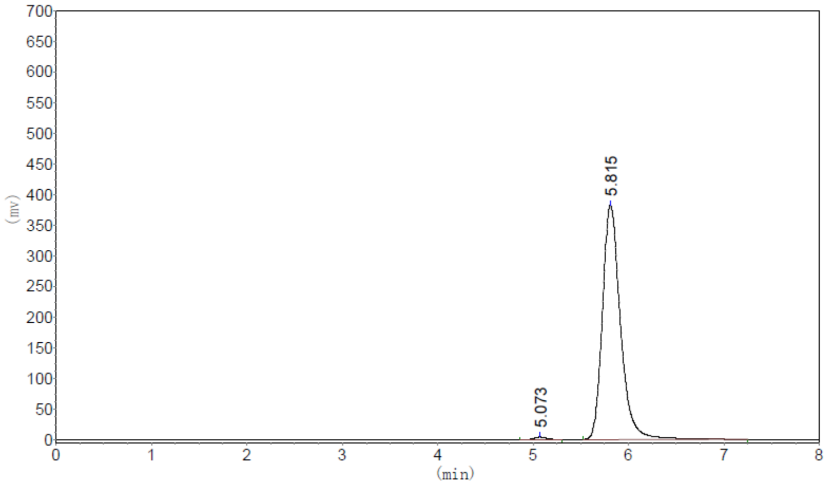

Peak No.	R. Time	Peak Height	Peak Area	Percent
1	5.073	4263.279	43089.738	0.8487
2	5.815	382014.375	5034179.500	99.1513
Total		386277.654	5077269.238	100.0000

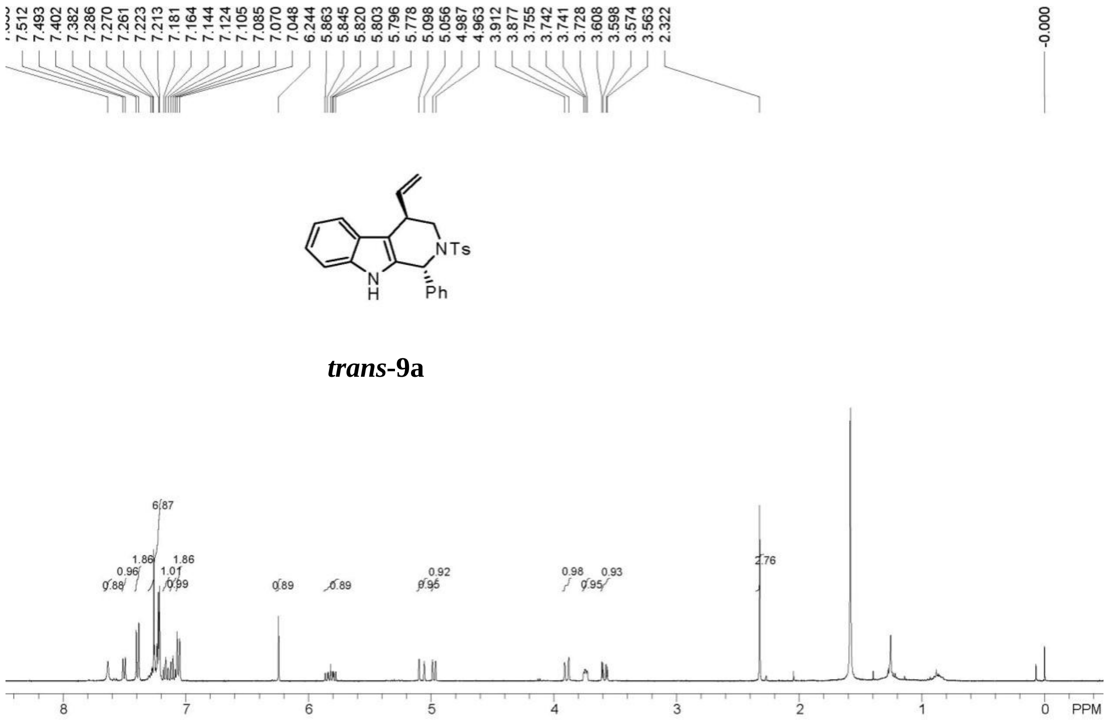

***trans*-9a**

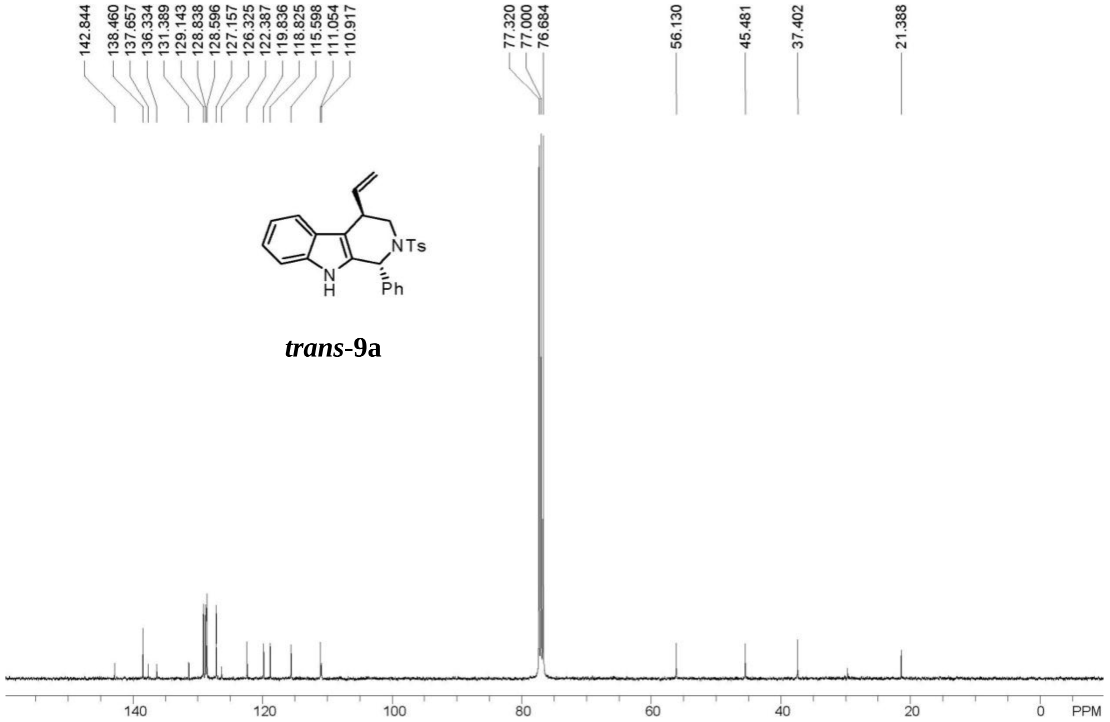

***trans*-9a**

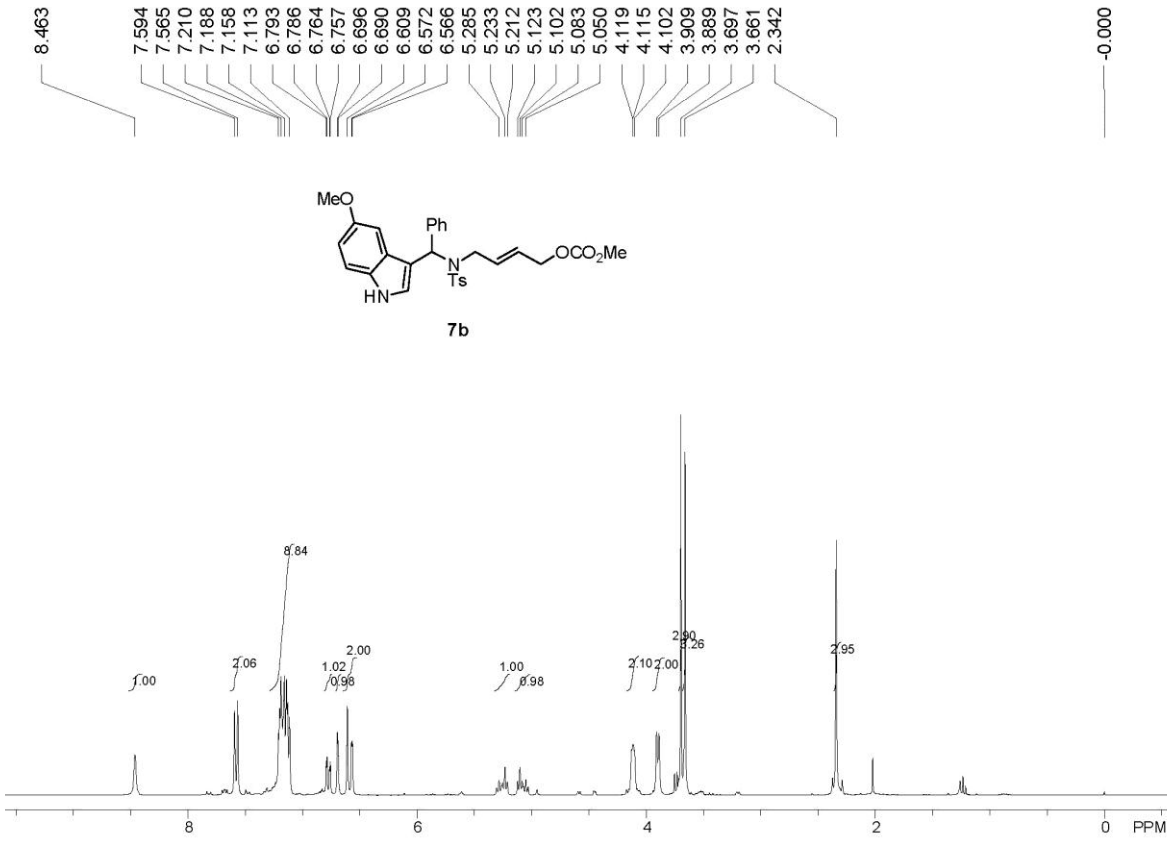
MeO
Ph
N
OCO2Me
Ts
HN
7b
PPM

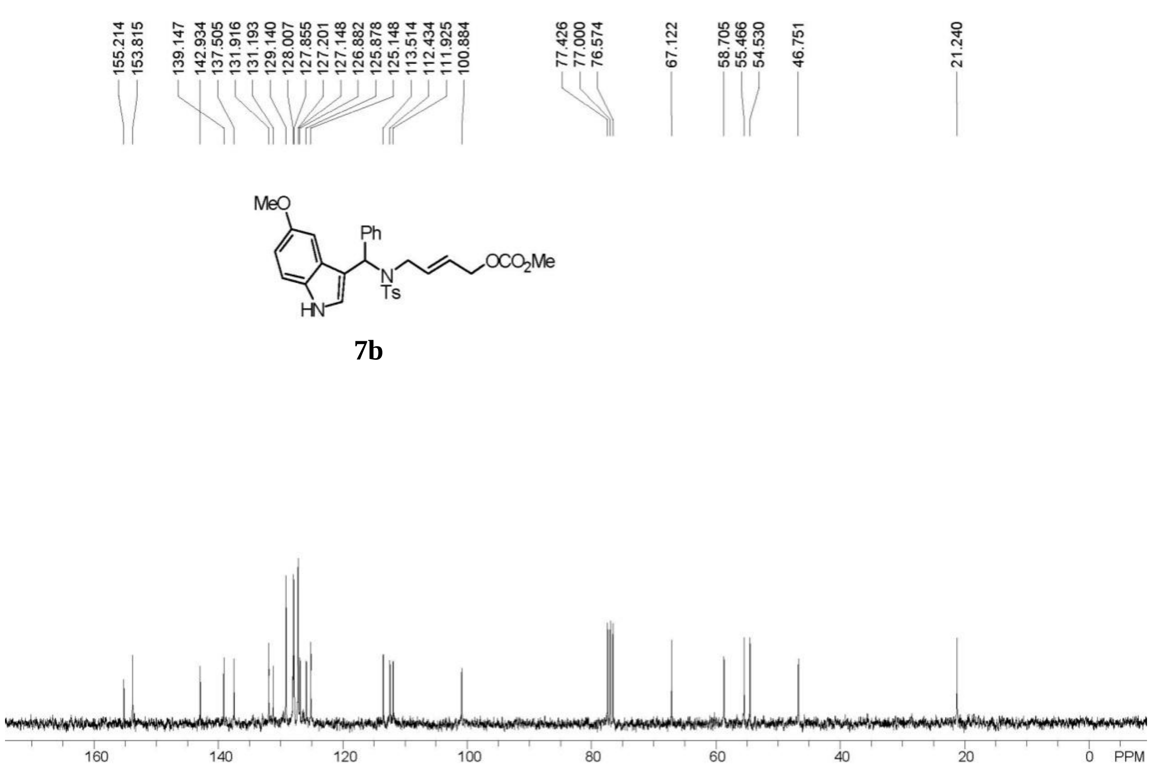
MeO
Ph
N
OCO2Me
Ts
HN
7b
PPM

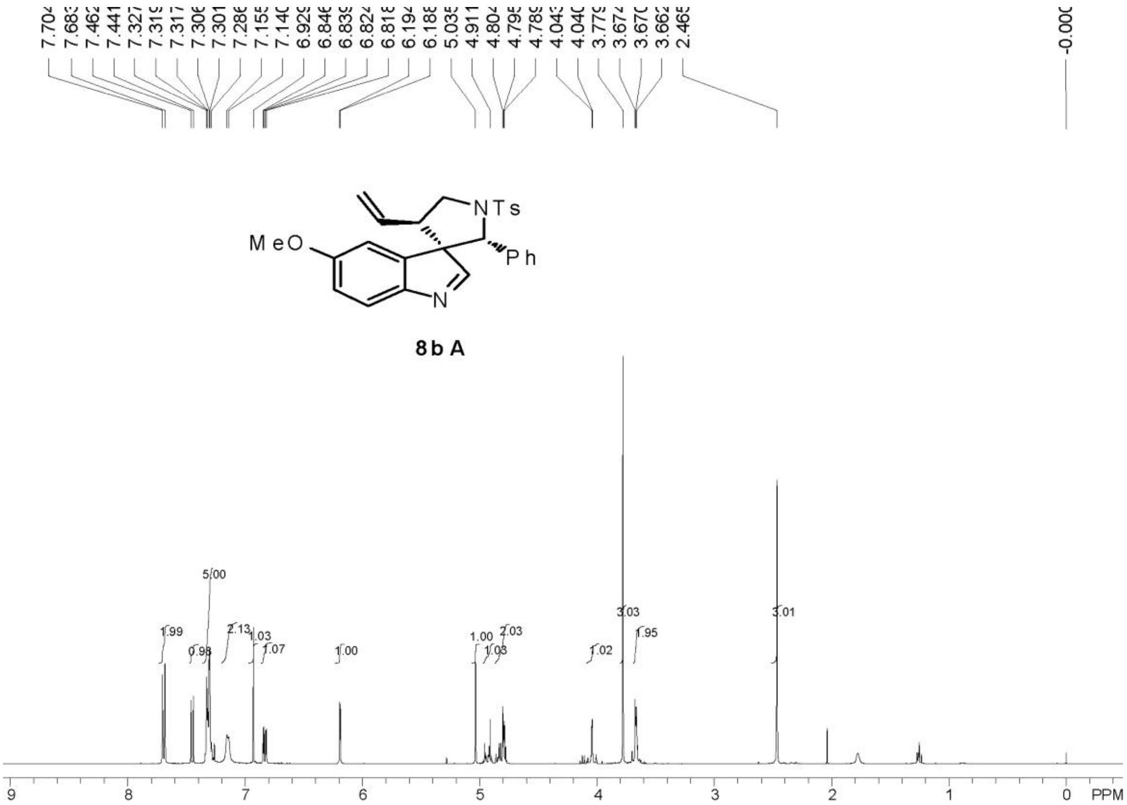
7.704
7.683
7.462
7.441
7.327
7.319
7.306
7.301
7.286
7.155
7.140
6.929
6.846
6.839
6.824
6.818
6.194
6.188
5.035
4.911
4.804
4.795
4.789
4.043
4.040
3.779
3.674
3.670
3.662
2.465
-0.000
MeO
NTs
Ph
N
8b A
1.99
0.98
5.00
2.13
1.03
1.07
1.00
1.00
1.03
2.03
1.02
3.03
1.95
3.01
9
8
7
6
5
4
3
2
1
0
PPM

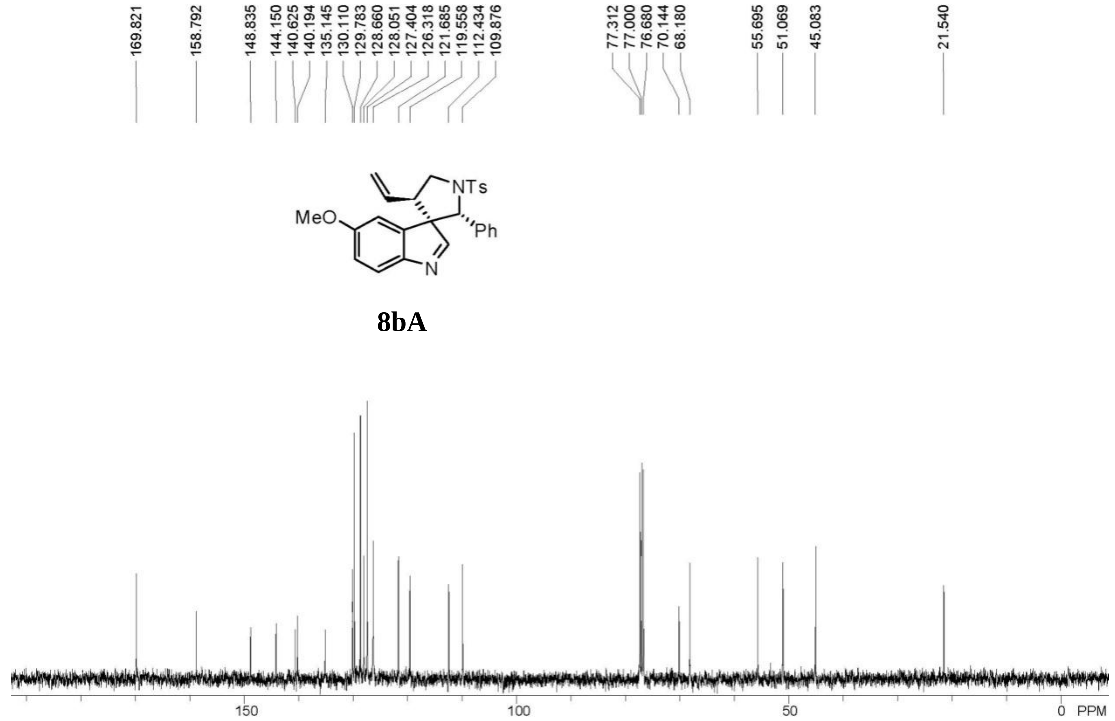
169.821
158.792
148.835
144.150
140.625
140.194
135.145
130.110
129.783
128.660
128.051
127.404
126.318
121.685
119.558
112.434
109.876
77.312
77.000
76.680
70.144
68.180
55.695
51.069
45.083
21.540
MeO
NTs
Ph
N
8bA
150
100
50
0
PPM

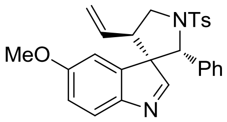

8bA

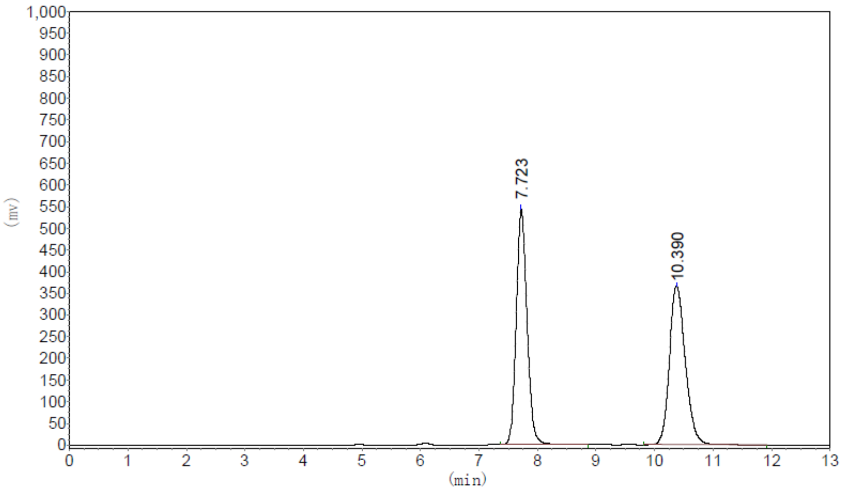

Peak No.	R. Time	Peak Height	Peak Area	Percent
1	7.723	540709.125	7116276.500	50.2335
2	10.390	366889.781	7050109.500	49.7665
Total		907598.906	14166386.000	100.0000

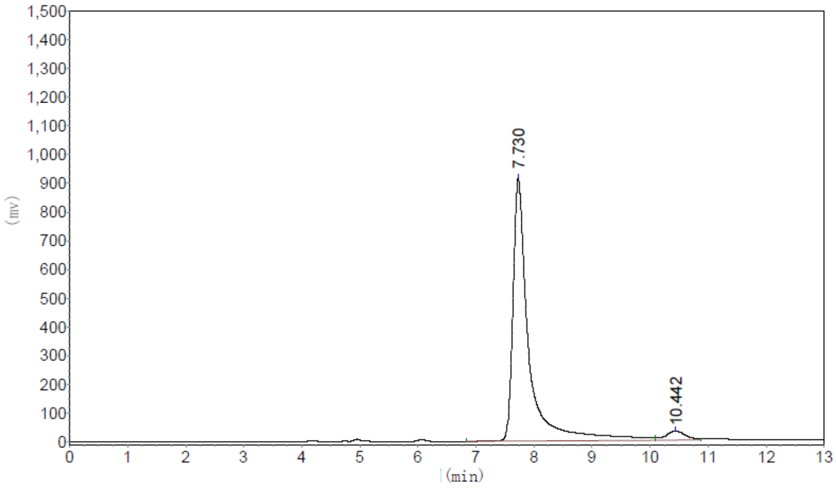

Peak No.	R. Time	Peak Height	Peak Area	Percent
1	7.730	915169.875	17413958.000	95.7883
2	10.442	33169.039	765674.438	4.2117
Total		948338.914	18179632.438	100.0000

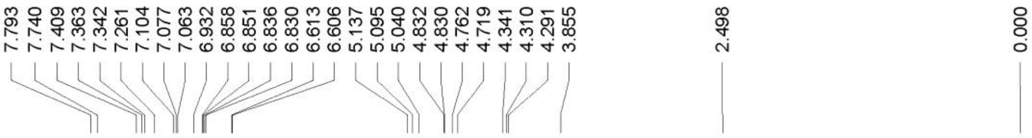
7.793 7.740 7.409 7.363 7.342 7.261 7.104 7.077 7.063 6.932 6.858 6.851 6.836 6.830 6.613 6.606 5.137 5.095 5.040 4.832 4.830 4.762 4.719 4.341 4.310 4.291 3.855
2.498
0.000

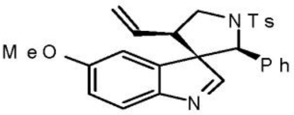
MeO
NTs
Ph
N

8 b B

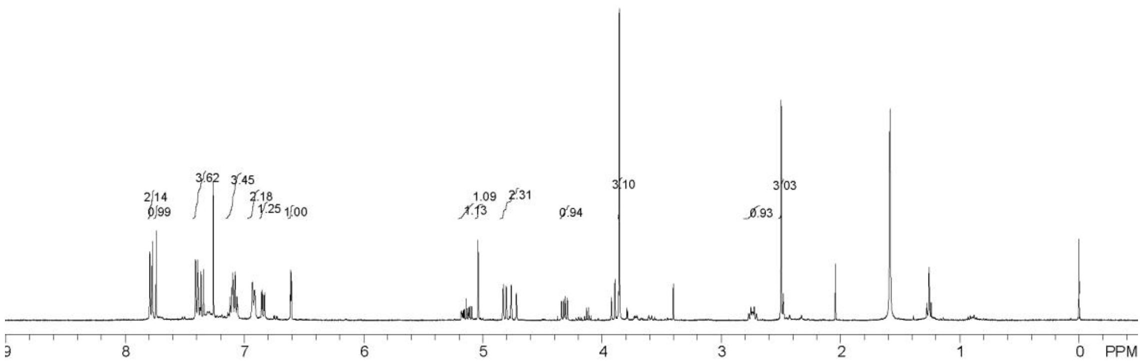
2.14
0.99
3.62
3.45
2.18
1.25
1.00
1.09
1.13
2.31
0.94
3.10
0.93
3.03
9 8 7 6 5 4 3 2 1 0 PPM

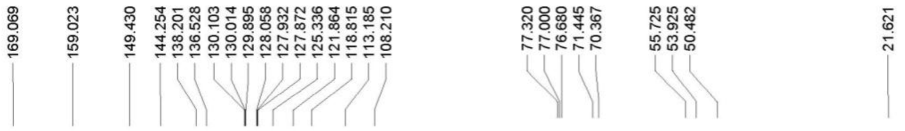
169.069
159.023
149.430
144.254 138.201 136.528 130.103 130.014 129.895 128.058 127.932 127.872 125.336 121.864 118.815 113.185 108.210
77.320 77.000 76.680 71.445 70.367
55.725 53.925 50.482
21.621

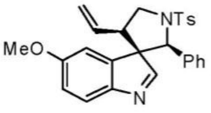
MeO
NTs
Ph
N

8bB

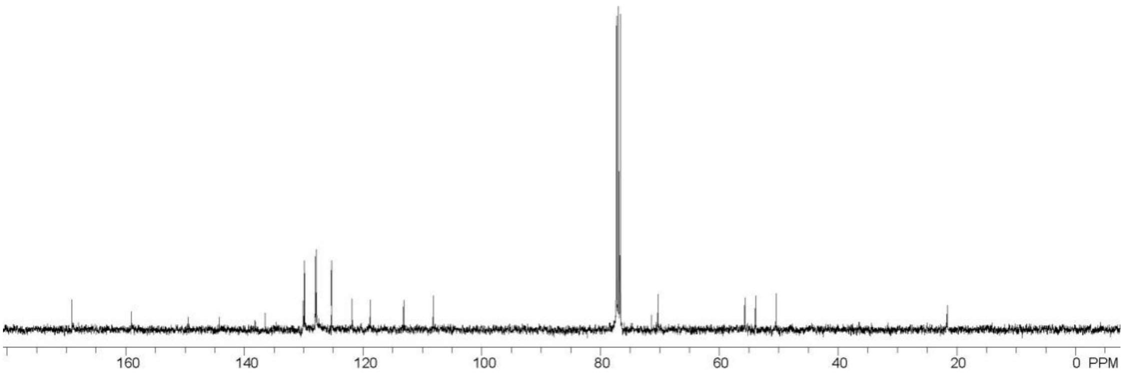
160 140 120 100 80 60 40 20 0 PPM

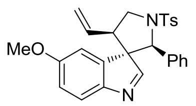

8bB

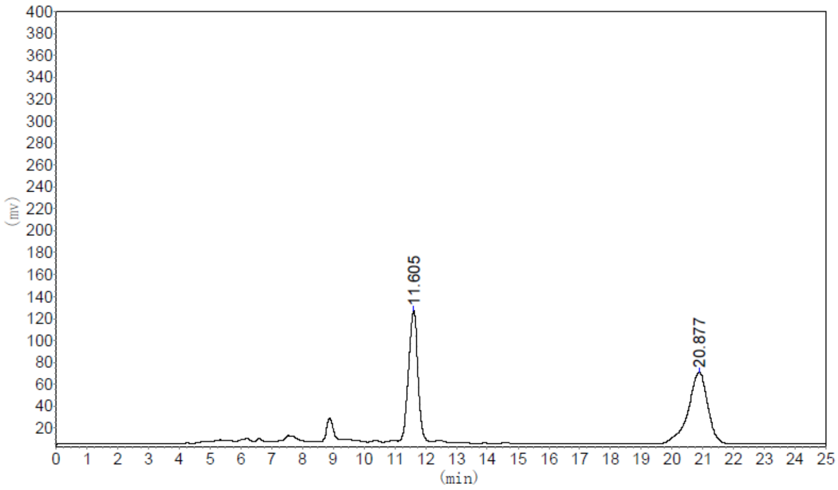

Peak No.	R. Time	Peak Height	Peak Area	Percent
1	11. 605	118977. 305	2467633. 500	49. 8051
2	20. 877	62457. 961	2486948. 250	50. 1949
Total		181435. 266	4954581. 750	100. 0000

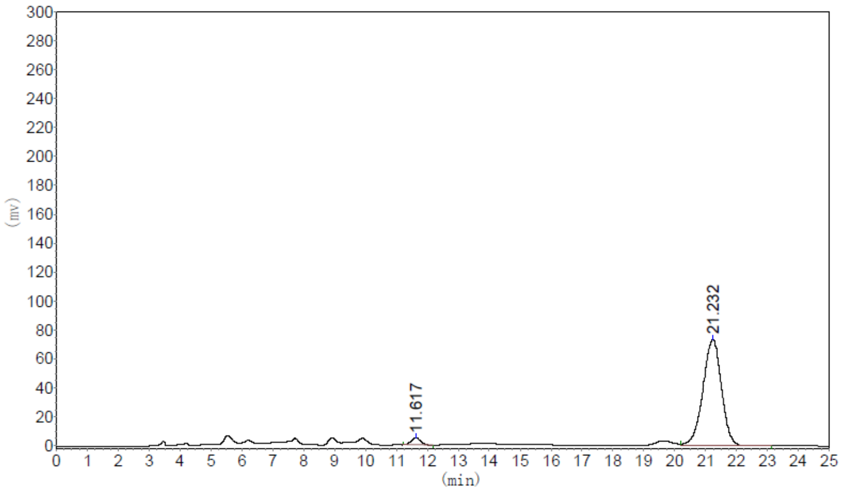

Peak No.	R. Time	Peak Height	Peak Area	Percent
1	11. 617	4817. 790	101250. 797	3. 1721
2	21. 232	73813. 289	3090687. 500	96. 8279
Total		78631. 079	3191938. 297	100. 0000

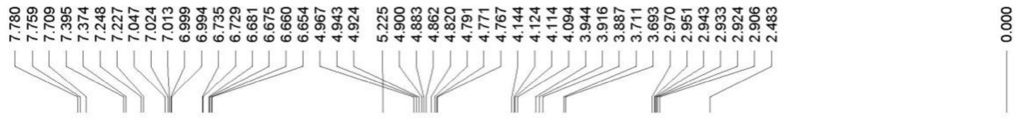
7.780
7.759
7.709
7.395
7.374
7.248
7.227
7.047
7.024
7.013
6.999
6.994
6.735
6.729
6.681
6.675
6.660
6.654
4.967
4.943
4.924
5.225
4.900
4.883
4.862
4.820
4.791
4.771
4.767
4.144
4.124
4.114
4.094
3.944
3.916
3.887
3.711
3.693
2.970
2.951
2.943
2.933
2.924
2.906
2.483
0.000

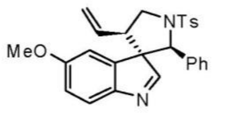
NTs
MeO
Ph
N

8bC

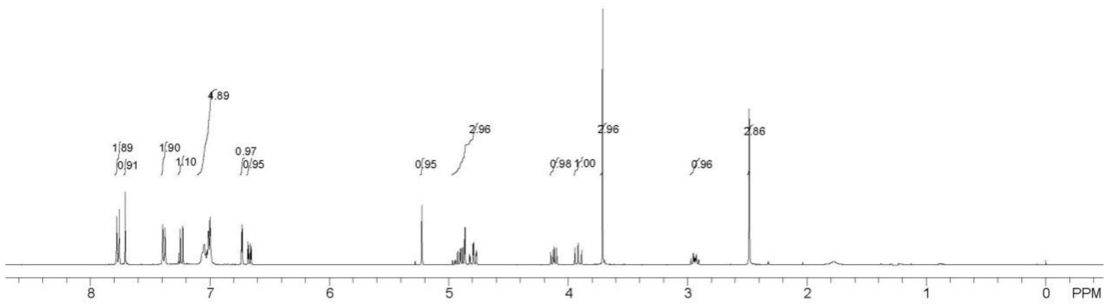
1.89
0.91
1.90
1.10
4.89
0.97
0.95
0.95
2.96
0.98
1.00
2.96
0.96
2.86
8
7
6
5
4
3
2
1
0
PPM

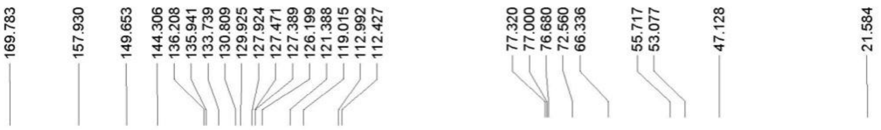
169.783
157.930
149.653
144.306
136.208
135.941
133.739
130.809
129.925
127.924
127.471
127.389
126.199
121.388
119.015
112.992
112.427
77.320
77.000
76.680
72.560
66.336
55.717
53.077
47.128
21.584

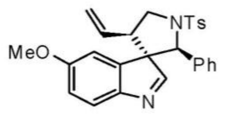
NTs
MeO
Ph
N

8bC

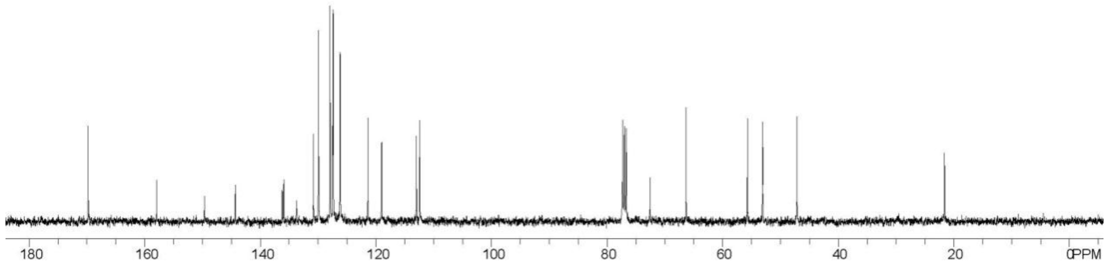
180
160
140
120
100
80
60
40
20
0
PPM

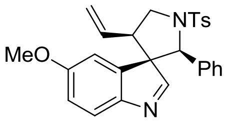

8bC

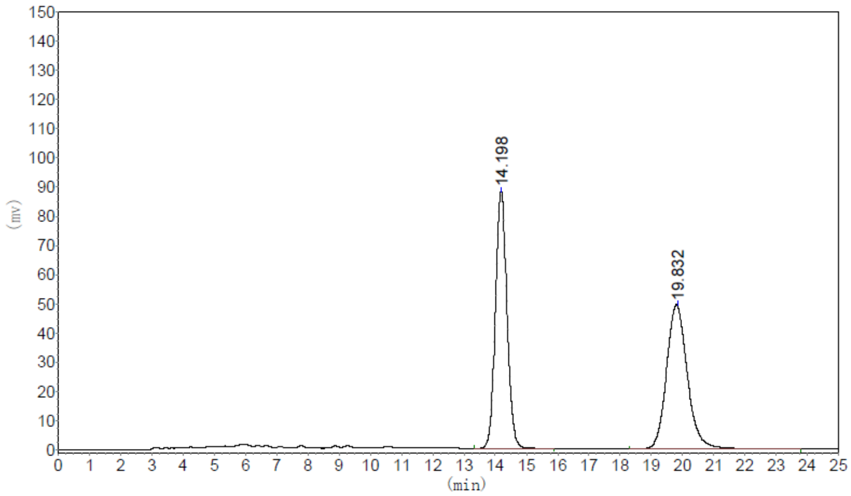

Peak No.	R. Time	Peak Height	Peak Area	Percent
1	14. 198	87976. 633	2260111. 750	49. 7934
2	19. 832	49305. 832	2278870. 500	50. 2066
Total		137282. 465	4538982. 250	100. 0000

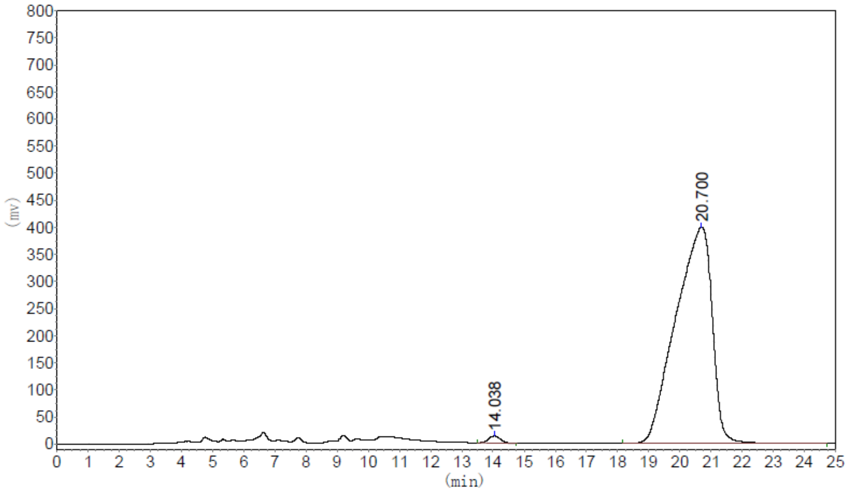

Peak No.	R. Time	Peak Height	Peak Area	Percent
1	14. 038	13490. 872	351391. 938	1. 0728
2	20. 700	399616. 719	32404534. 000	98. 9272
Total		413107. 591	32755925. 938	100. 0000

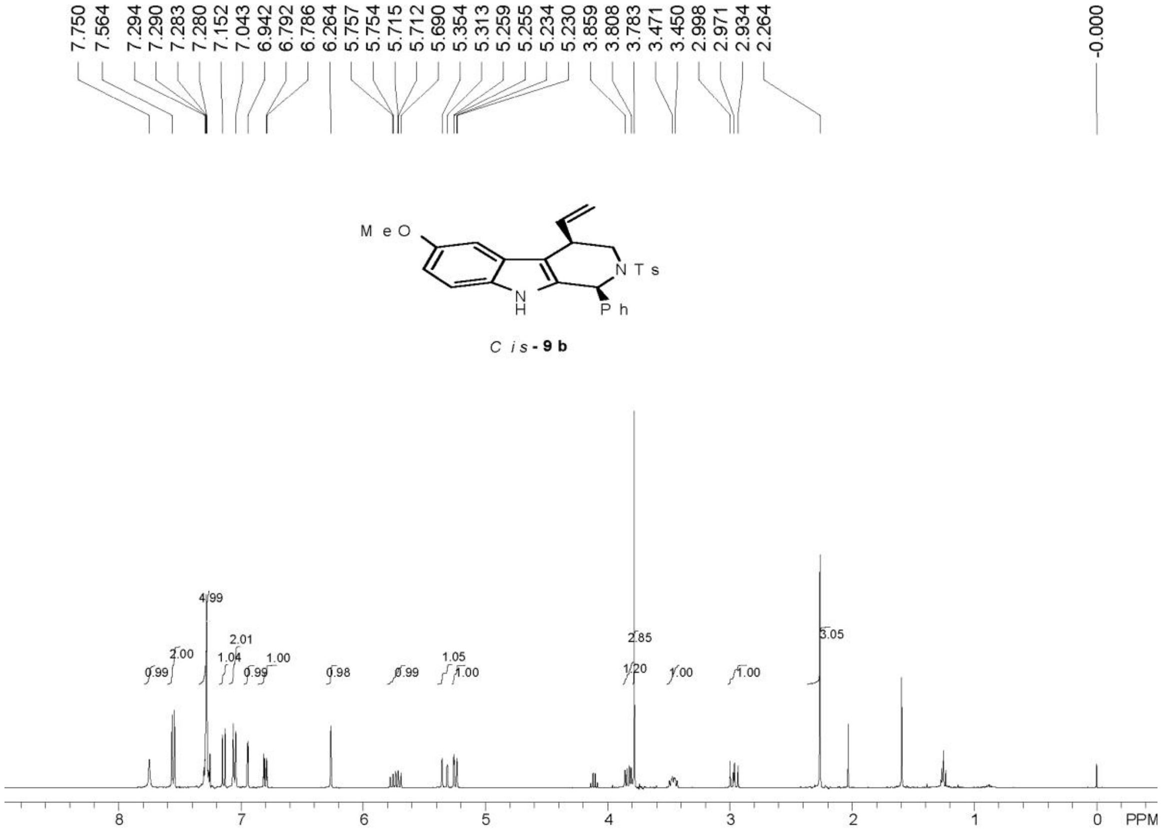
7.750 7.564 7.294 7.290 7.283 7.280 7.152 7.043 6.942 6.792 6.786 6.264 5.757 5.754 5.715 5.712 5.690 5.354 5.313 5.259 5.255 5.234 5.230 3.859 3.808 3.783 3.471 3.450 2.998 2.971 2.934 2.264
-0.000
MeO
NTs
N
H
Ph
Cis-9b
0.99 2.00 4.99 1.04 2.01 0.99 1.00 0.98 0.99 1.05 1.00 1.20 2.85 1.00 1.00 3.05
8 7 6 5 4 3 2 1 0 PPM

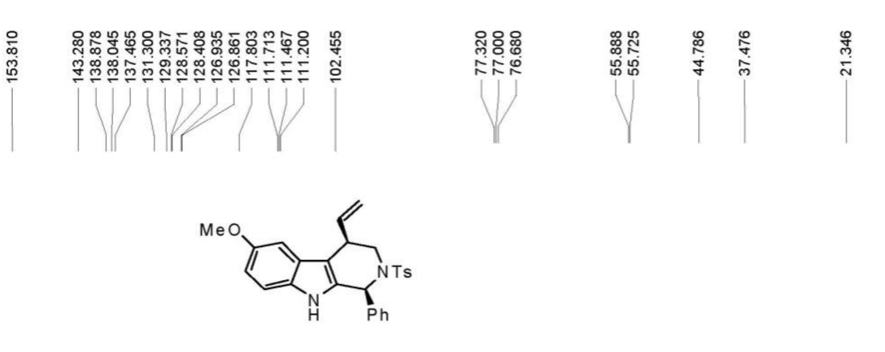
153.810
143.280 138.878 138.045 137.465 131.300 129.337 128.571 128.408 126.935 126.861 117.803 111.713 111.467 111.200
102.455
77.320 77.000 76.680
55.888 55.725
44.786
37.476
21.346
MeO
NTs
N
H
Ph

***cis*-9b**

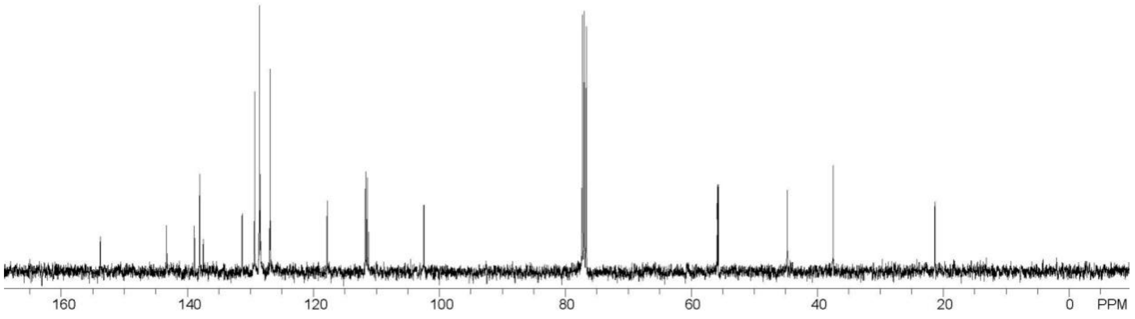
160 140 120 100 80 60 40 20 0 PPM

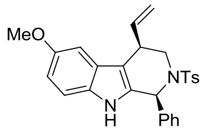

***cis*-9b**

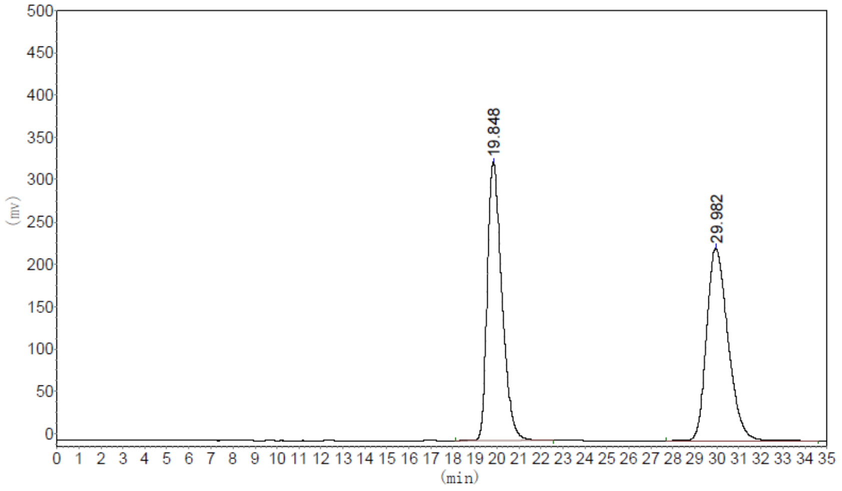

Peak No.	R. Time	Peak Height	Peak Area	Percent
1	19.848	328925.906	15147290.000	49.6639
2	29.982	227404.766	15352333.000	50.3361
Total		556330.672	30499623.000	100.0000

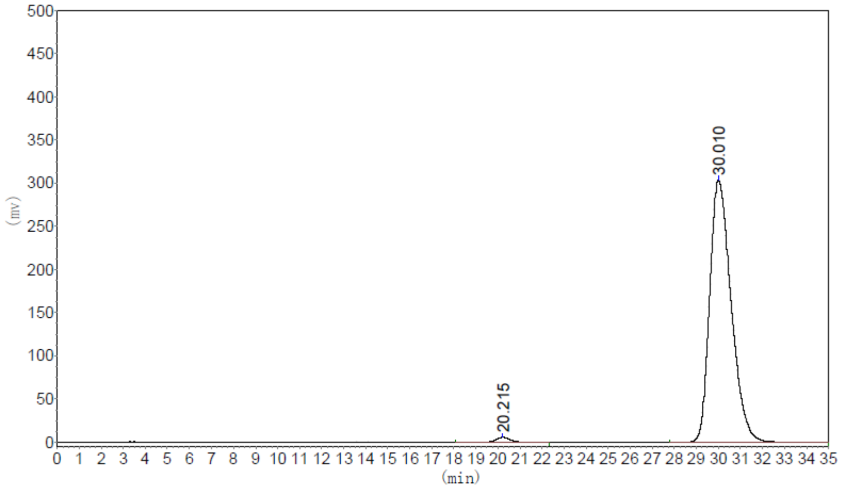

Peak No.	R. Time	Peak Height	Peak Area	Percent
1	20.215	5566.340	260036.906	1.2479
2	30.010	303660.031	20577094.000	98.7521
Total		309226.371	20837130.906	100.0000

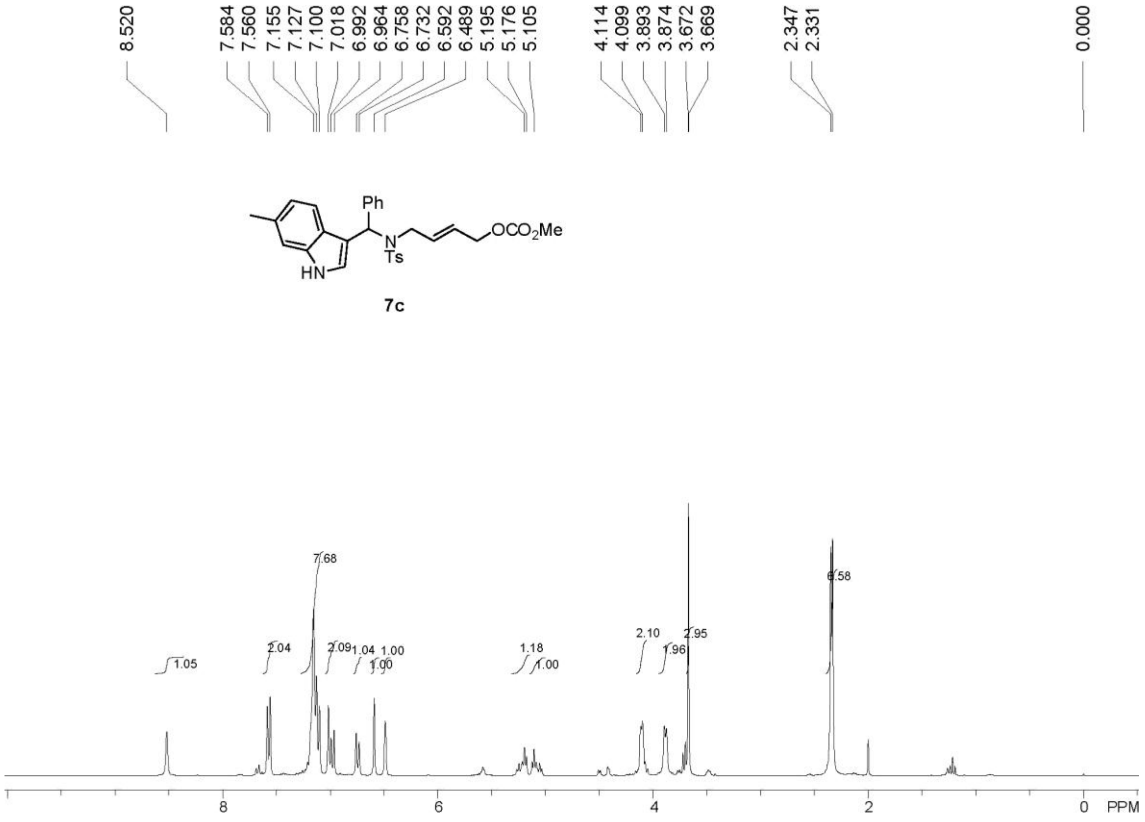
8.520
7.584
7.560
7.155
7.127
7.100
7.018
6.992
6.964
6.758
6.732
6.592
6.489
5.195
5.176
5.105
4.114
4.099
3.893
3.874
3.672
3.669
2.347
2.331
0.000
1.05
2.04
7.68
2.09
1.04
1.00
1.00
1.18
1.00
2.10
1.96
2.95
6.58
8
6
4
2
0
PPM
Ph
N
Ts
OCO2Me
HN

7c

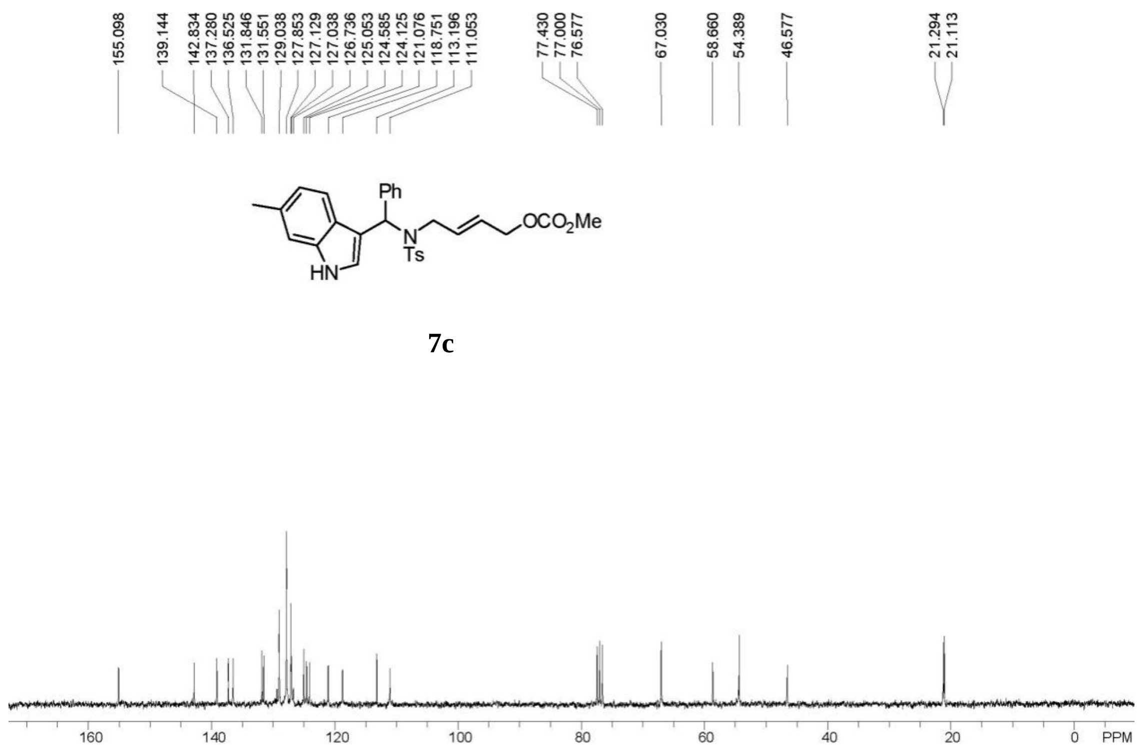
155.098
139.144
142.834
137.280
136.525
131.846
131.551
129.038
127.853
127.129
127.038
126.736
125.053
124.585
124.125
121.076
118.751
113.196
111.053
77.430
77.000
76.577
67.030
58.660
54.389
46.577
21.294
21.113
160
140
120
100
80
60
40
20
0
PPM
Ph
N
Ts
OCO2Me
HN

7c

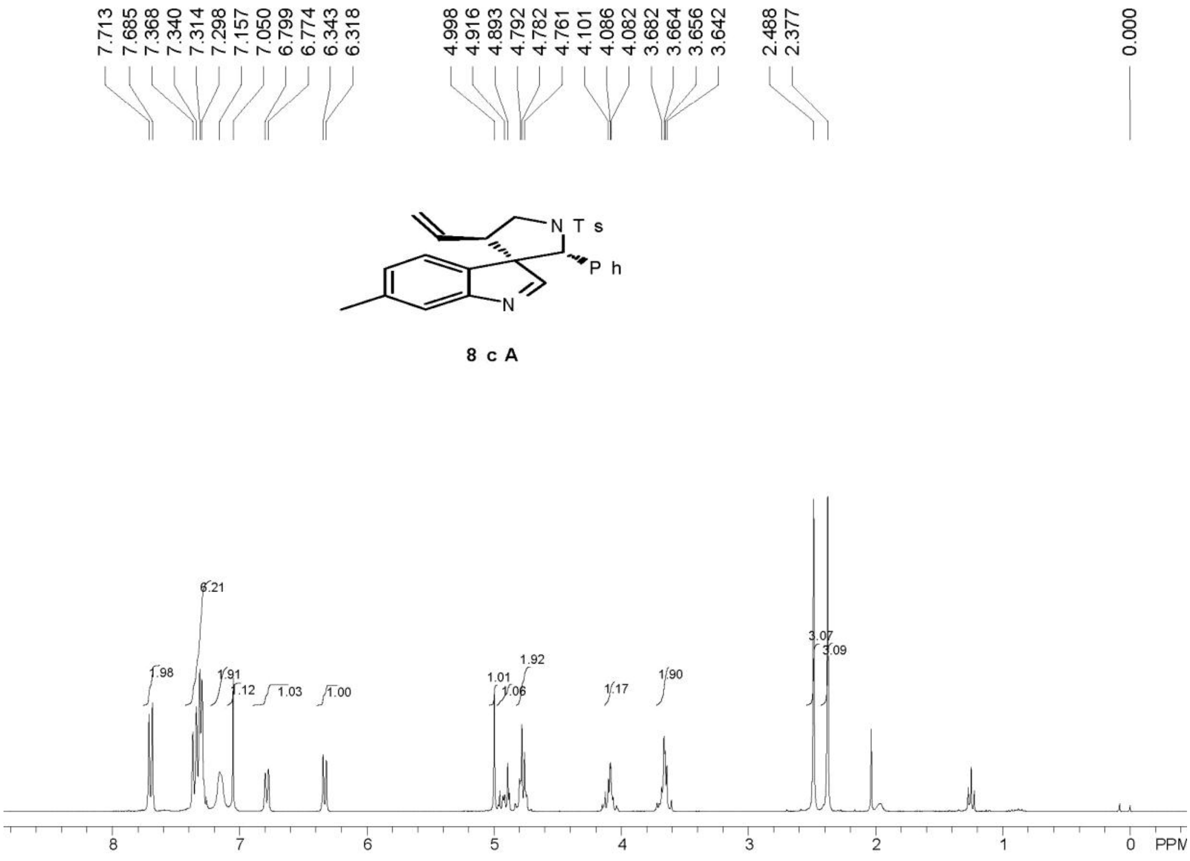
7.713 7.685 7.368 7.340 7.314 7.298 7.157 7.050 6.799 6.774 6.343 6.318
4.998 4.916 4.893 4.792 4.782 4.761 4.101 4.086 4.082 3.682 3.664 3.656 3.642
2.488 2.377
0.000
N Ts
Ph
N
8cA
1.98 6.21 1.91 1.12 1.03 1.00 1.01 1.06 1.92 1.17 1.90 3.07 3.09
8 7 6 5 4 3 2 1 0 PPM

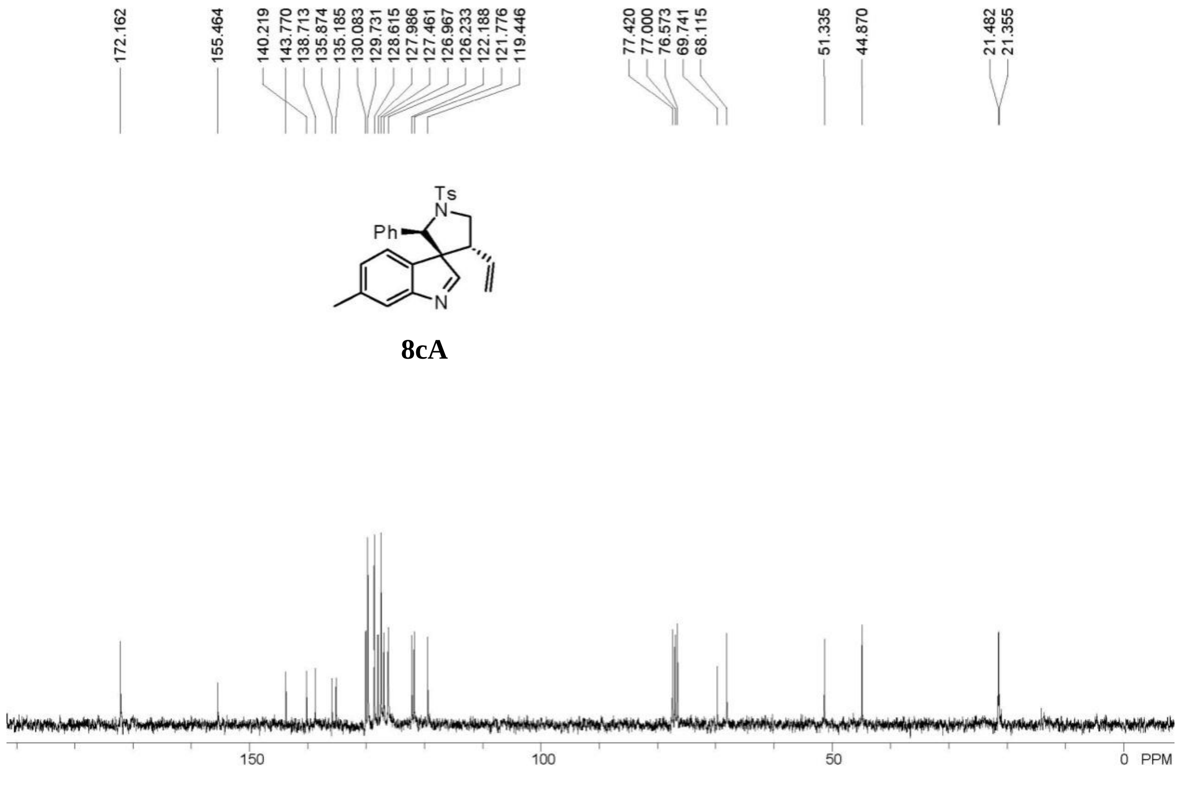
172.162
155.464
140.219 143.770 138.713 135.874 135.185 130.083 129.731 128.615 127.986 127.461 126.967 126.233 122.188 121.776 119.446
77.420 77.000 76.573 69.741 68.115
51.335 44.870
21.482 21.355
Ts
N
Ph
N
8cA
150 100 50 0 PPM

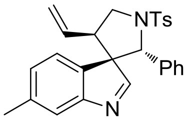

8cA

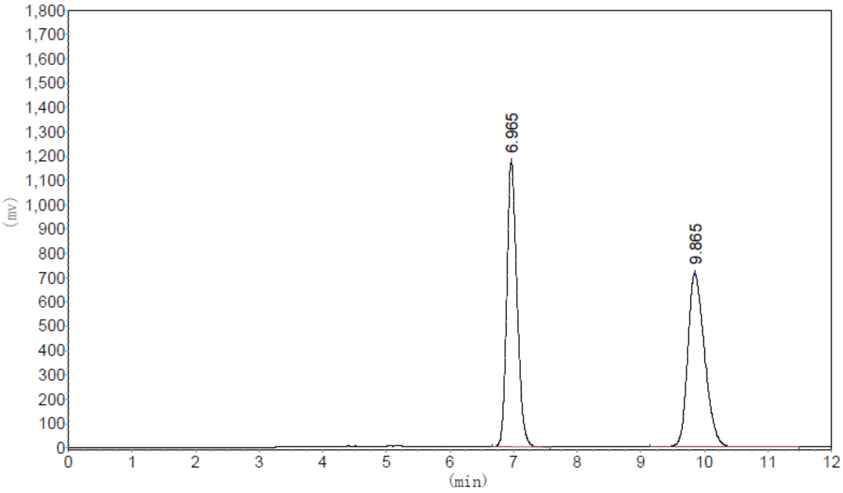

Peak No.	R. Time	Peak Height	Peak Area	Percent
1	6. 965	1162275. 375	13140004. 000	49. 7458
2	9. 865	716888. 938	13274310. 000	50. 2542
Total		1879164. 313	26414314. 000	100. 0000

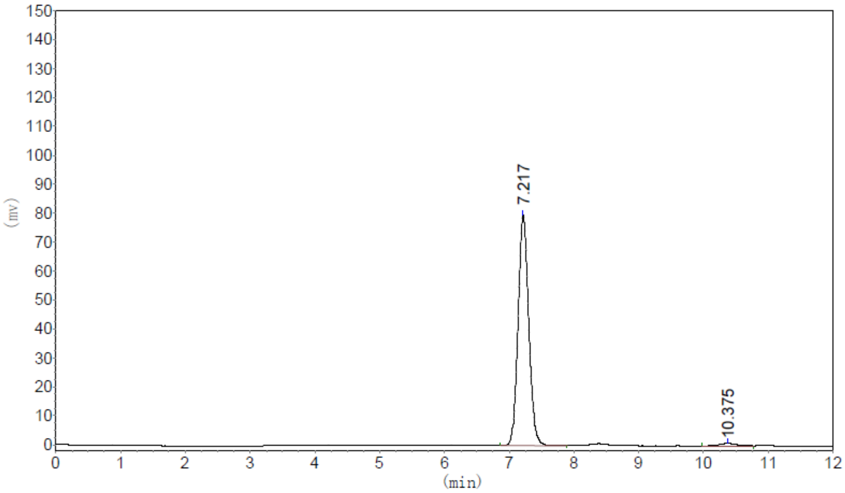

Peak No.	R. Time	Peak Height	Peak Area	Percent
1	7. 217	79879. 766	911817. 563	98. 4273
2	10. 375	807. 526	14569. 095	1. 5727
Total		80687. 292	926386. 657	100. 0000

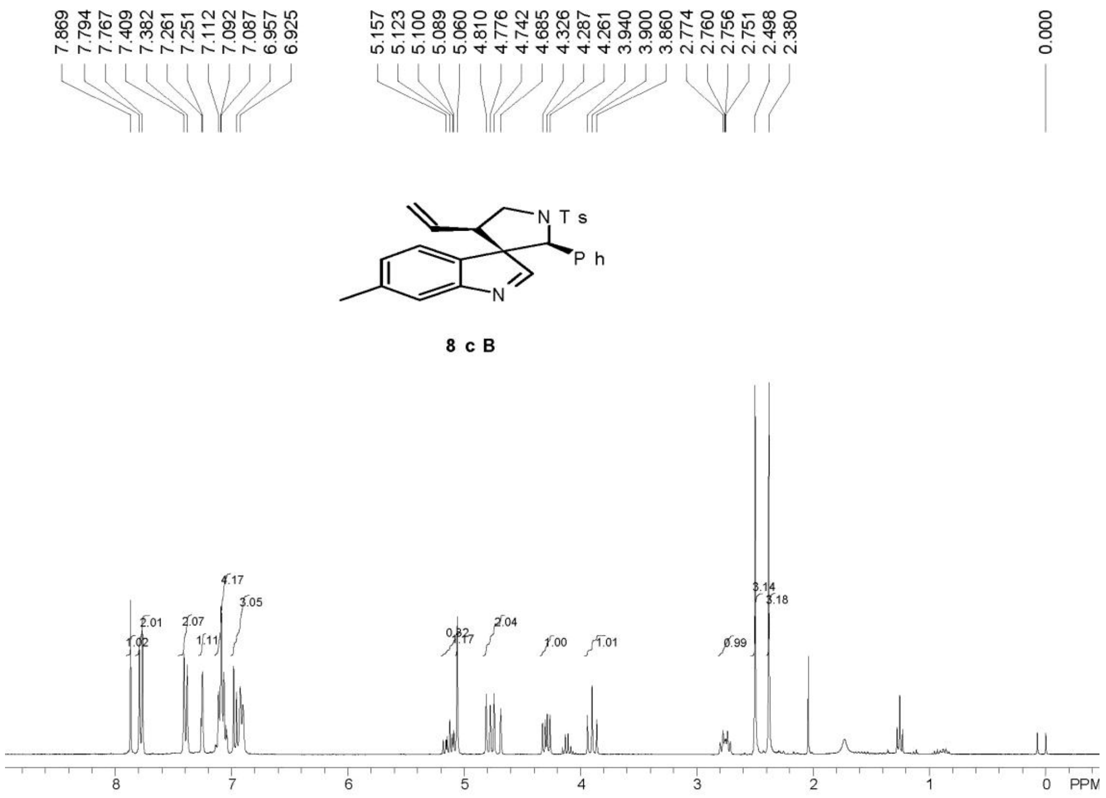
7.869
7.794
7.767
7.409
7.382
7.261
7.251
7.112
7.092
7.087
6.957
6.925
5.157
5.123
5.100
5.089
5.060
4.810
4.776
4.742
4.685
4.326
4.287
4.261
3.940
3.900
3.860
2.774
2.760
2.756
2.751
2.498
2.380
0.000
N Ts
P h
N
8 c B
PPM

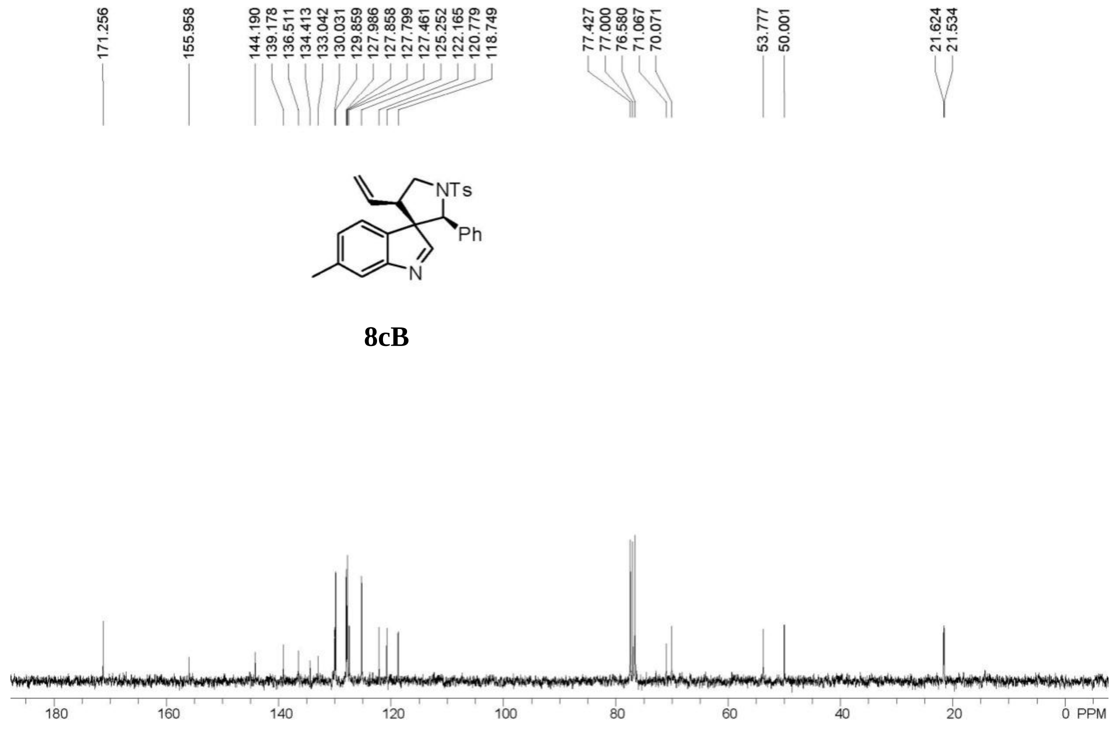
171.256
155.958
144.190
139.178
136.511
134.413
133.042
130.031
129.859
127.986
127.858
127.799
127.461
125.252
122.165
120.779
118.749
77.427
77.000
76.580
71.067
70.071
53.777
50.001
21.624
21.534
NTs
Ph
N
8cB
PPM

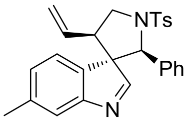

8cB

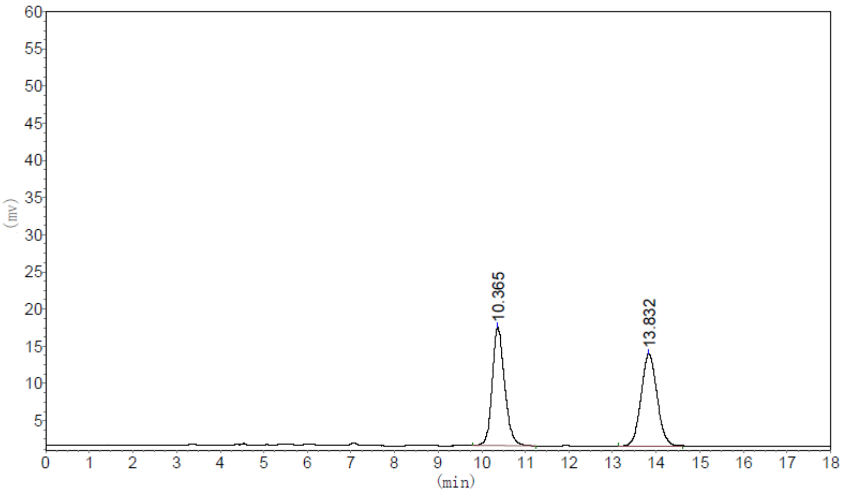

Peak No.	R. Time	Peak Height	Peak Area	Percent
1	10. 365	15969. 237	315543. 250	50. 2344
2	13. 832	12384. 750	312598. 906	49. 7656
Total		28353. 987	628142. 156	100. 0000

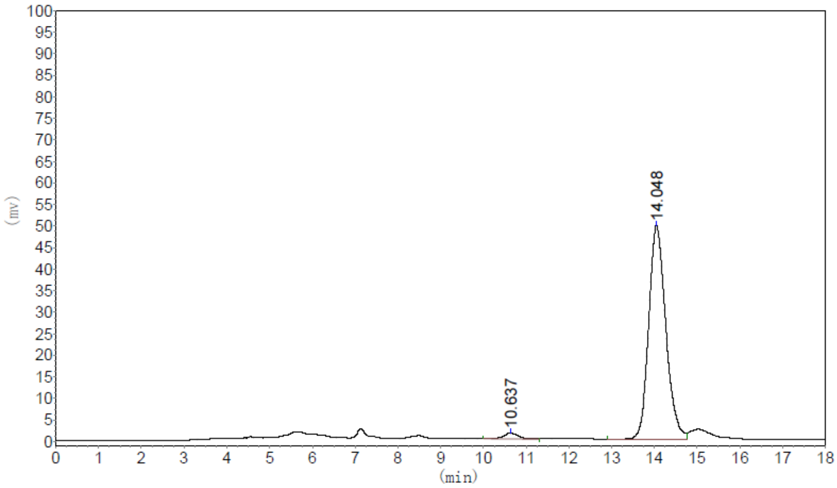

Peak No.	R. Time	Peak Height	Peak Area	Percent
1	10. 637	1276. 857	29526. 777	2. 1040
2	14. 048	49514. 367	1373817. 125	97. 8960
Total		50791. 224	1403343. 902	100. 0000

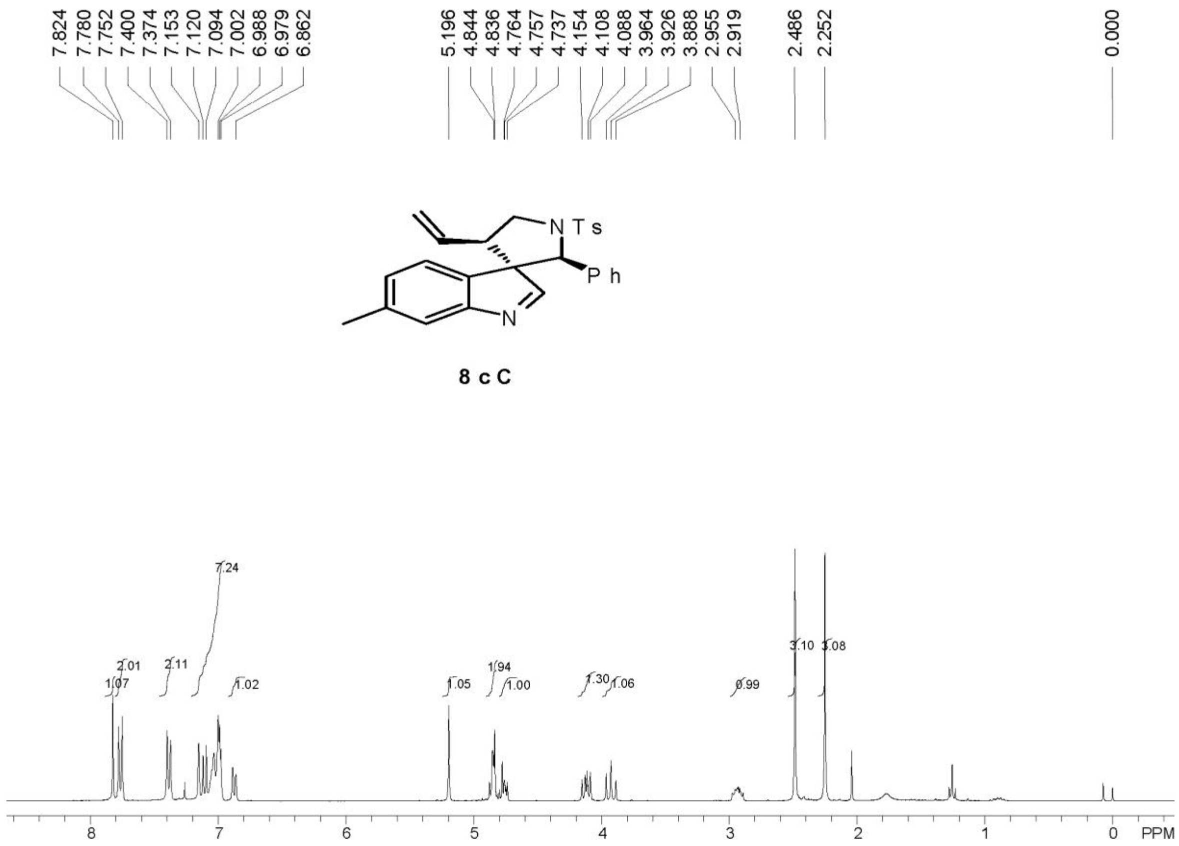
7.824
7.780
7.752
7.400
7.374
7.153
7.120
7.094
7.002
6.988
6.979
6.862
5.196
4.844
4.836
4.764
4.757
4.737
4.154
4.108
4.088
3.964
3.926
3.888
2.955
2.919
2.486
2.252
0.000
NTs
Ph
N
1.07
2.01
2.11
7.24
1.02
1.05
1.94
1.00
1.30
1.06
0.99
3.10
3.08
8
7
6
5
4
3
2
1
0
PPM

8 c C

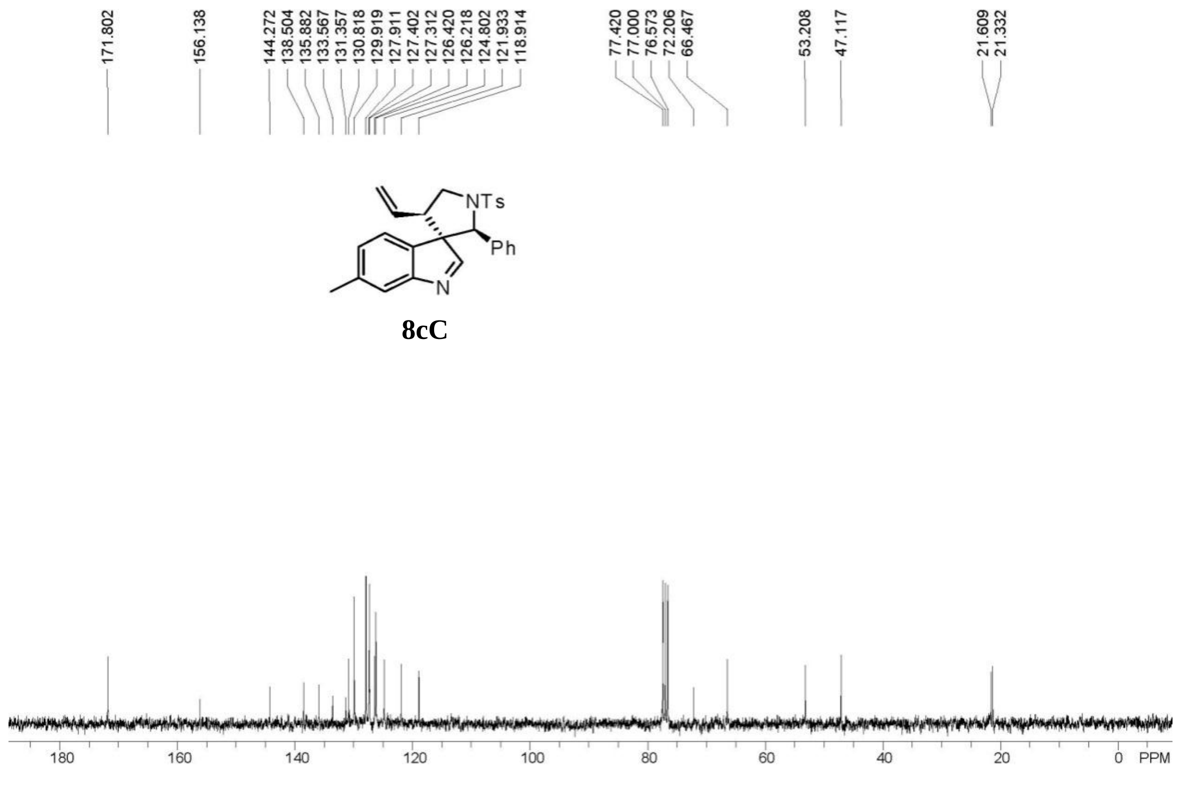
171.802
156.138
144.272
138.504
135.882
133.567
131.357
130.818
129.919
127.911
127.402
127.312
126.420
126.218
124.802
121.933
118.914
77.420
77.000
76.573
72.206
66.467
53.208
47.117
21.609
21.332
NTs
Ph
N
180
160
140
120
100
80
60
40
20
0
PPM

8cC

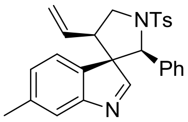

8cC

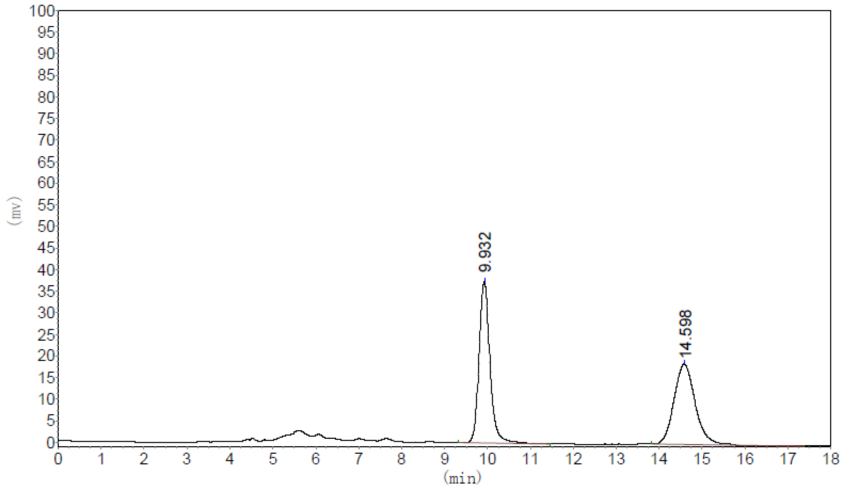

Peak No.	R. Time	Peak Height	Peak Area	Percent
1	9. 932	37385. 430	665337. 188	50. 1577
2	14. 598	18775. 295	661152. 875	49. 8423
Total		56160. 725	1326490. 063	100. 0000

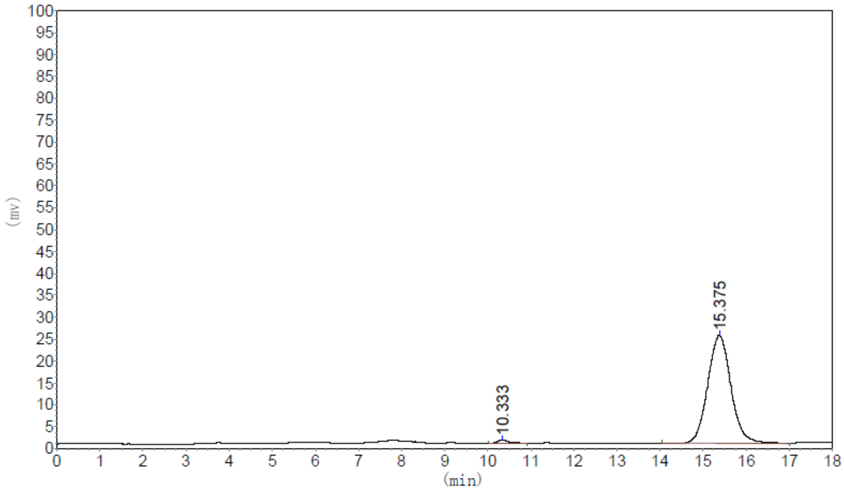

Peak No.	R. Time	Peak Height	Peak Area	Percent
1	10. 333	711. 000	14429. 507	1. 5400
2	15. 375	24627. 164	922553. 250	98. 4600
Total		25338. 164	936982. 757	100. 0000

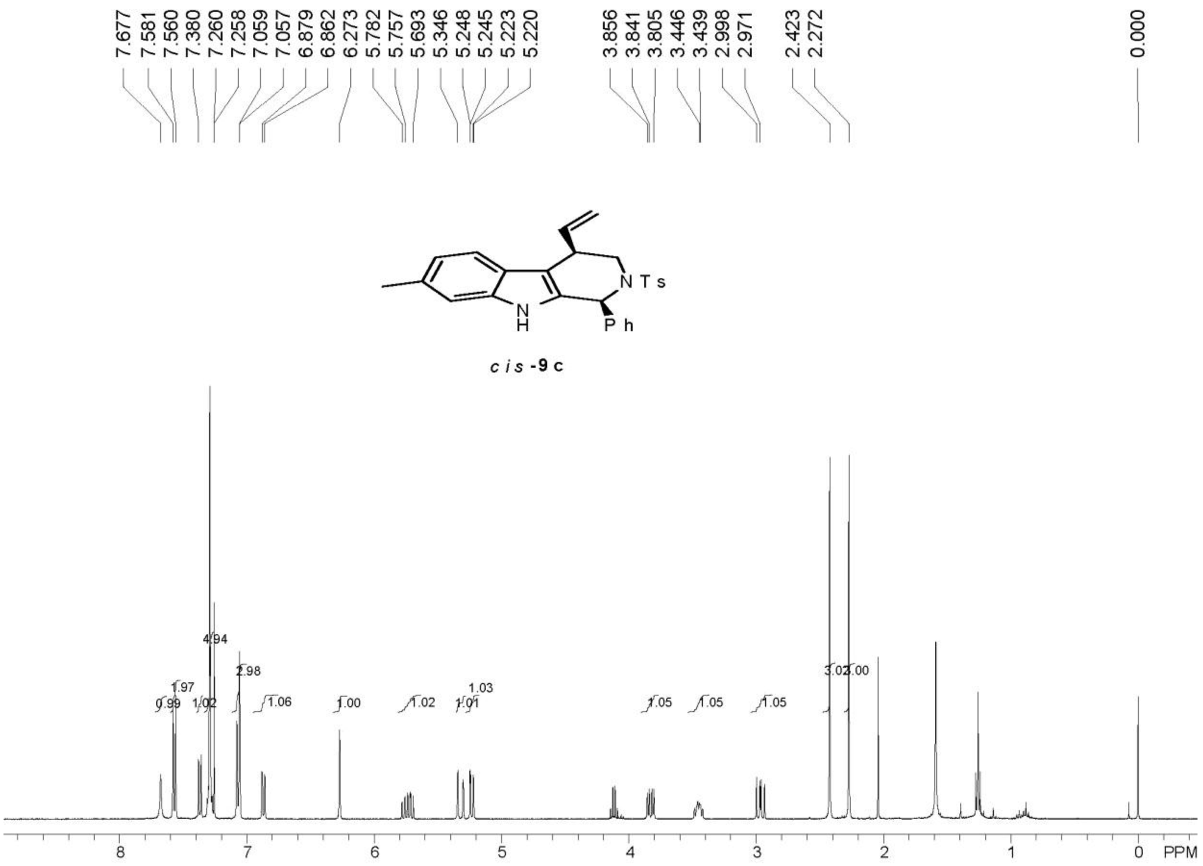
7.677 7.581 7.560 7.380 7.260 7.258 7.059 7.057 6.879 6.862 6.273 5.782 5.757 5.693 5.346 5.248 5.245 5.223 5.220
3.856 3.841 3.805 3.446 3.439 2.998 2.971
2.423 2.272
0.000
NTs
H
Ph
cis-9c
4.94
1.97
2.98
0.99 1.02 1.06 1.00 1.02 1.01 1.03 1.05 1.05 1.05 3.02 3.00
8 7 6 5 4 3 2 1 0 PPM

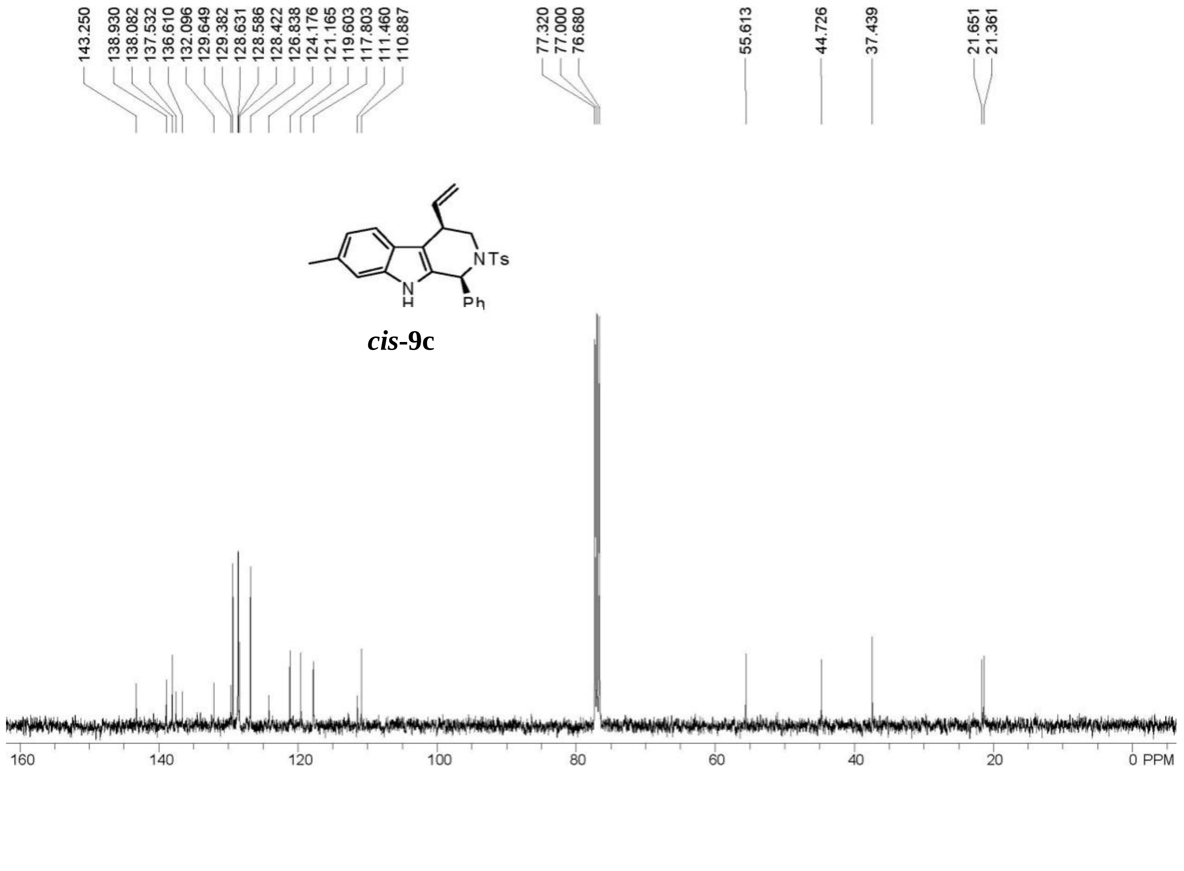
143.250 138.930 138.082 137.532 136.610 132.096 129.649 129.382 128.631 128.586 128.422 126.838 124.176 121.165 119.603 117.803 111.460 110.887
77.320 77.000 76.680
55.613
44.726
37.439
21.651 21.361
NTs
H
Ph
cis-9c
160 140 120 100 80 60 40 20 0 PPM

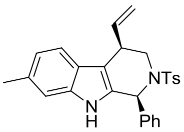

***cis*-9c**

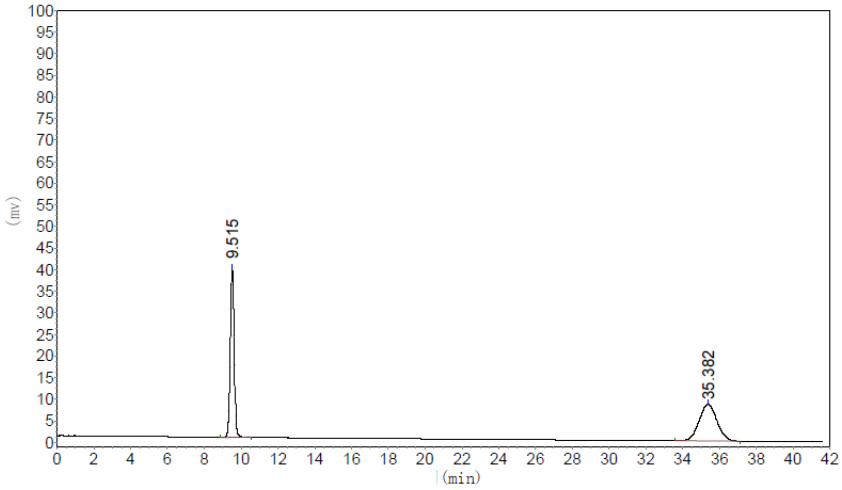

Peak No.	R. Time	Peak Height	Peak Area	Percent
1	9.515	39070.129	573467.813	50.1281
2	35.382	8468.283	570536.813	49.8719
Total		47538.412	1144004.625	100.0000

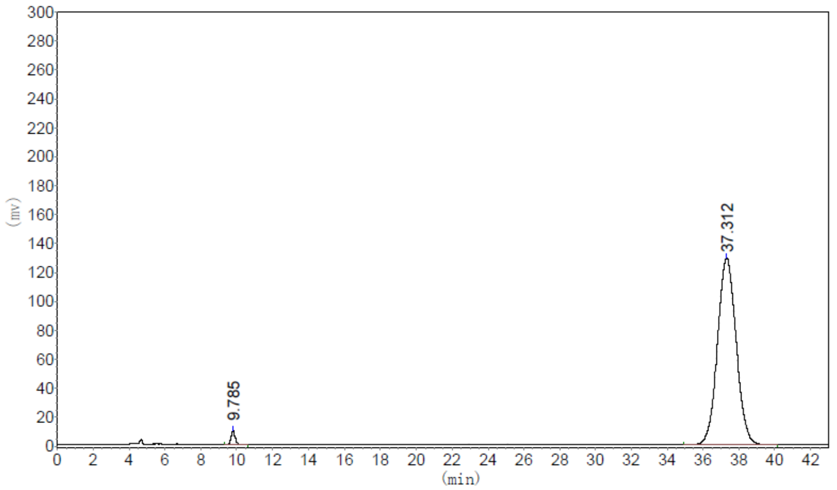

Peak No.	R. Time	Peak Height	Peak Area	Percent
1	9.785	9537.067	152081.344	1.6127
2	37.312	128784.641	9278389.000	98.3873
Total		138321.708	9430470.344	100.0000

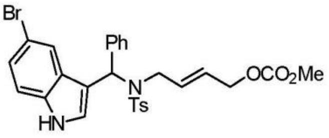
Br
Ph
N
Ts
HN
OCO2Me

7d

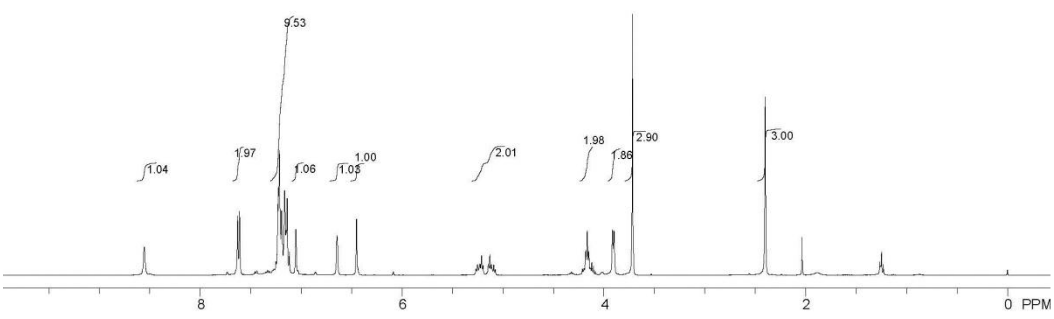
8.554
7.631
7.610
7.250
7.233
7.226
7.216
7.195
7.164
7.150
7.139
7.117
7.055
7.052
6.647
6.642
6.452
5.253
5.230
5.214
5.198
5.146
5.131
5.116
5.092
4.197
4.180
4.166
4.151
4.140
4.133
3.915
3.900
3.719
2.402
-0.000
1.04
1.97
9.53
1.06
1.03
1.00
2.01
1.98
1.86
2.90
3.00
8
6
4
2
0 PPM

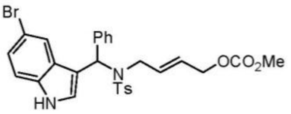
Br
Ph
N
Ts
HN
OCO2Me

7d

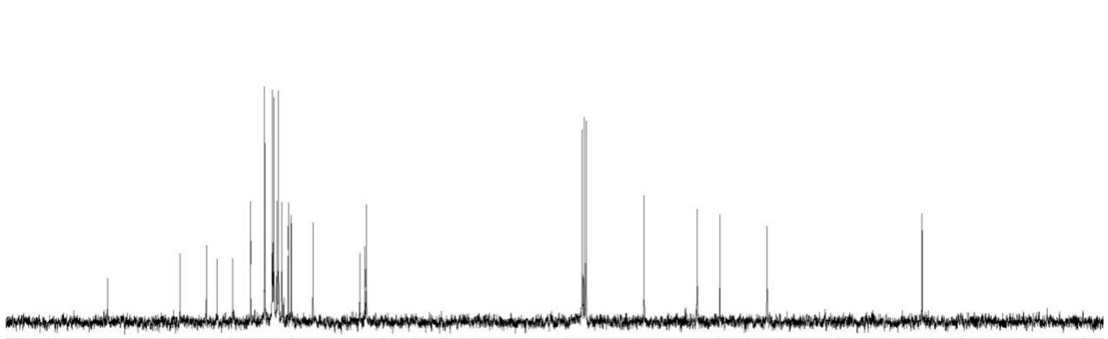
155.312
139.056
143.407
137.316
134.773
131.806
129.493
128.222
128.140
127.961
127.485
127.240
126.667
125.619
125.135
121.573
113.869
113.007
112.791
77.320
77.000
76.680
67.154
58.431
54.691
46.965
21.525
160
140
120
100
80
60
40
20
0 PPM

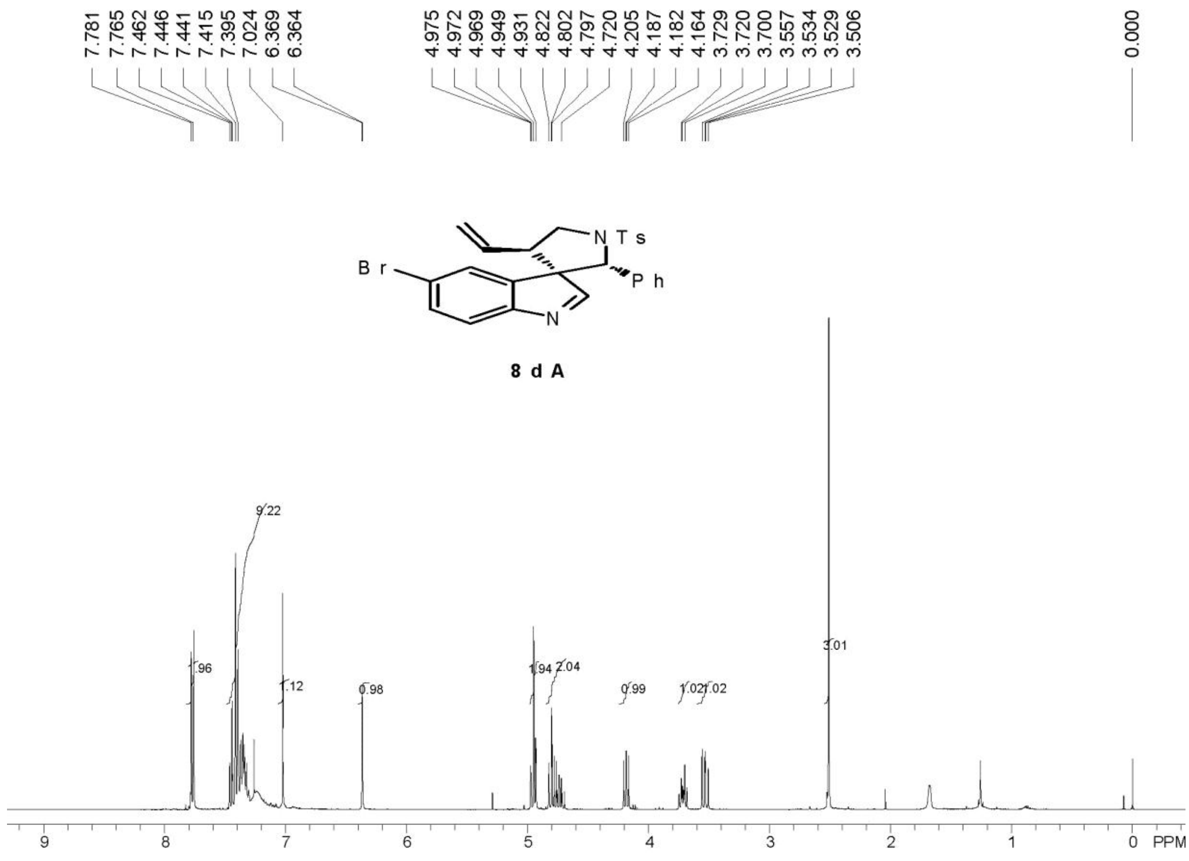
7.781 7.765 7.462 7.446 7.441 7.415 7.395 7.024 6.369 6.364
4.975 4.972 4.969 4.949 4.931 4.822 4.802 4.797 4.720 4.205 4.187 4.182 4.164 3.729 3.720 3.700 3.557 3.534 3.529 3.506
0.000
Br
NTs
Ph
N
8 d A
9.22
1.96
1.12
0.98
1.94
2.04
0.99
1.02
1.02
3.01
9 8 7 6 5 4 3 2 1 0 PPM

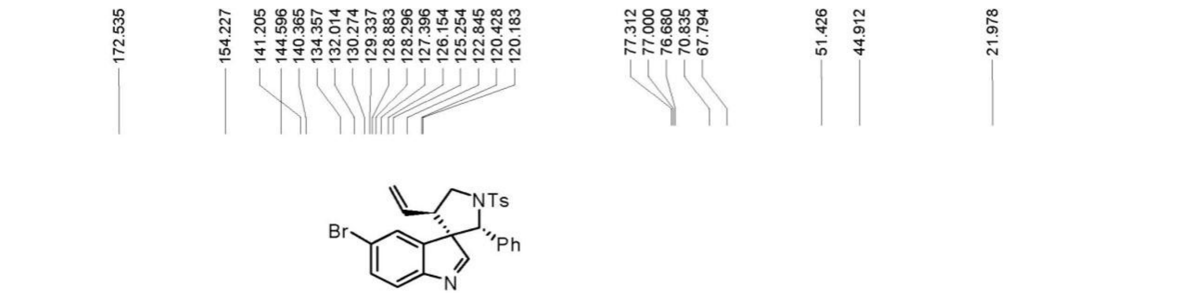
172.535
154.227
141.205 144.596 140.365 134.357 132.014 130.274 129.337 128.883 128.296 127.396 126.154 125.254 122.845 120.428 120.183
77.312 77.000 76.680 70.835 67.794
51.426
44.912
21.978
Br
NTs
Ph
N
150 100 50 0 PPM

8dA

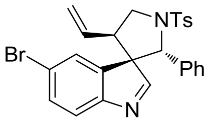

8dA

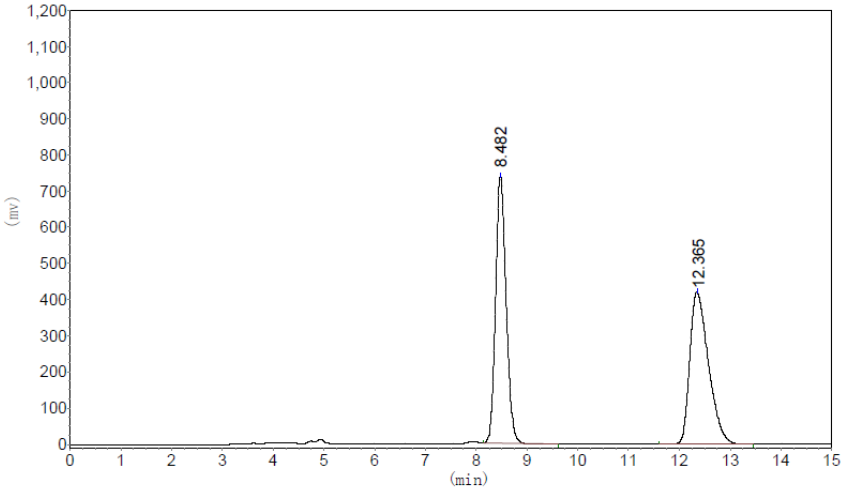

Peak No.	R. Time	Peak Height	Peak Area	Percent
1	8. 482	738700. 000	10829326. 000	49. 9627
2	12. 365	420649. 906	10845502. 000	50. 0373
Total		1159349. 906	21674828. 000	100. 0000

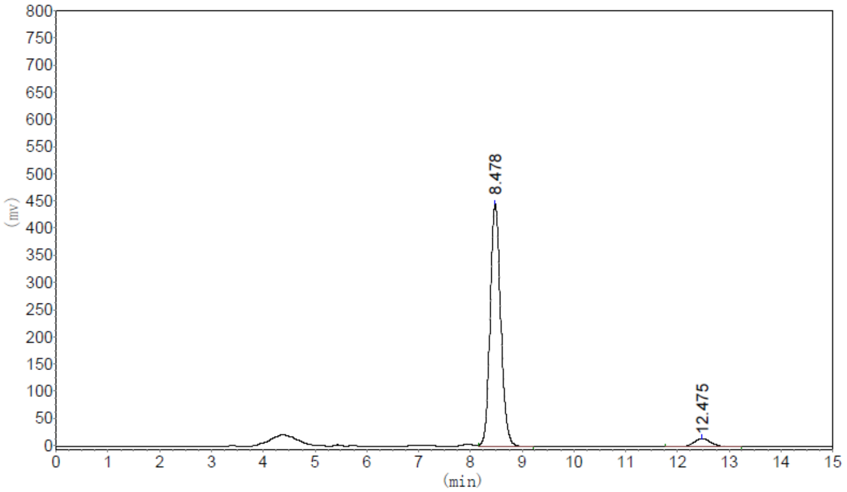

Peak No.	R. Time	Peak Height	Peak Area	Percent
1	8. 478	445193. 281	6314685. 000	95. 3317
2	12. 475	14071. 311	309221. 500	4. 6683
Total		459264. 592	6623906. 500	100. 0000

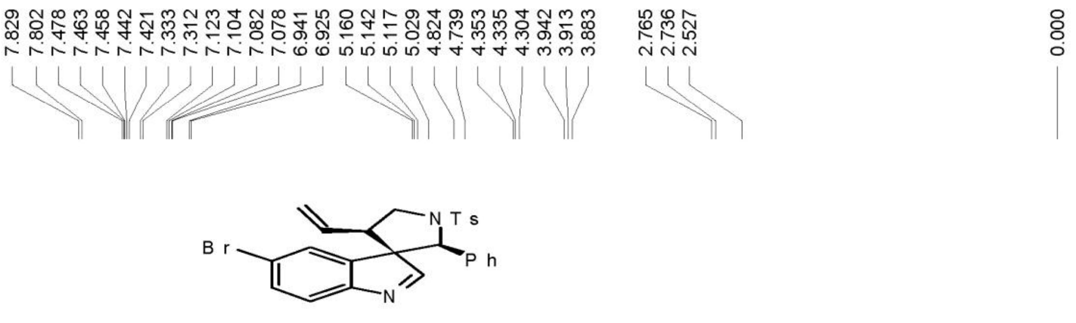
7.829 7.802 7.478 7.463 7.458 7.442 7.421 7.333 7.312 7.123 7.104 7.082 7.078 6.941 6.925 5.160 5.142 5.117 5.029 4.824 4.739 4.353 4.335 4.304 3.942 3.913 3.883 2.765 2.736 2.527 0.000
Br
NTs
Ph
N

8dB

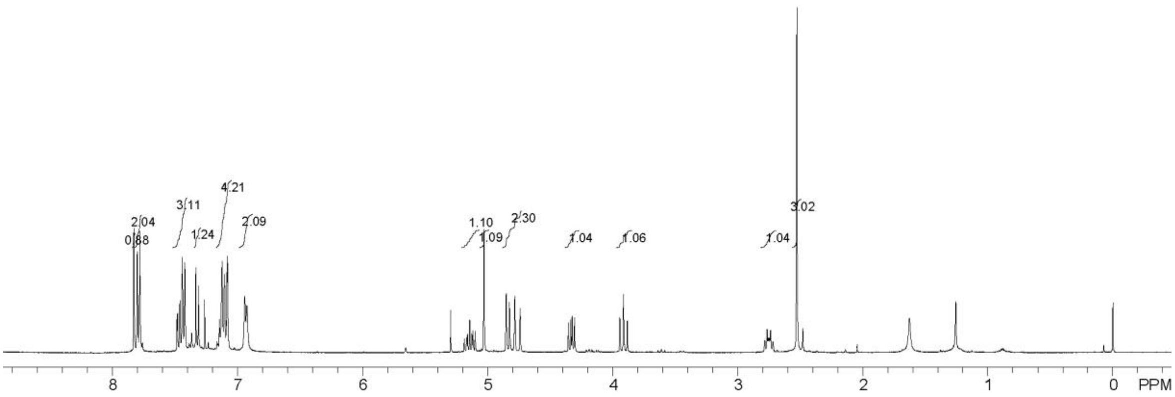
2.04
0.88
3.11
1.24
4.21
2.09
1.10
1.09
2.30
1.04
1.06
1.04
3.02
PPM

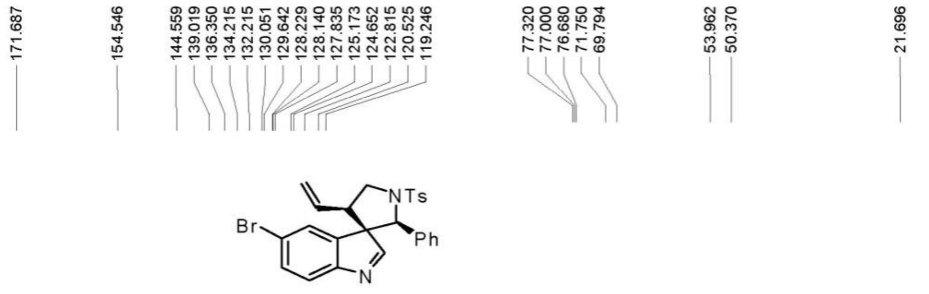
171.687 154.546 144.559 139.019 136.350 134.215 132.215 130.051 129.642 128.229 128.140 127.835 125.173 124.652 122.815 120.525 119.246 77.320 77.000 76.680 71.750 69.794 53.962 50.370 21.696
Br
NTs
Ph
N

8dB

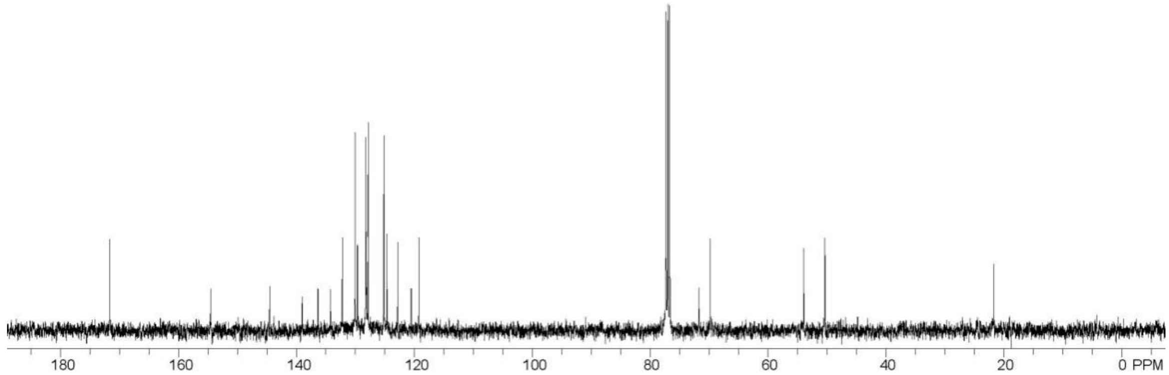
PPM

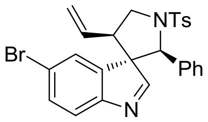

8dB

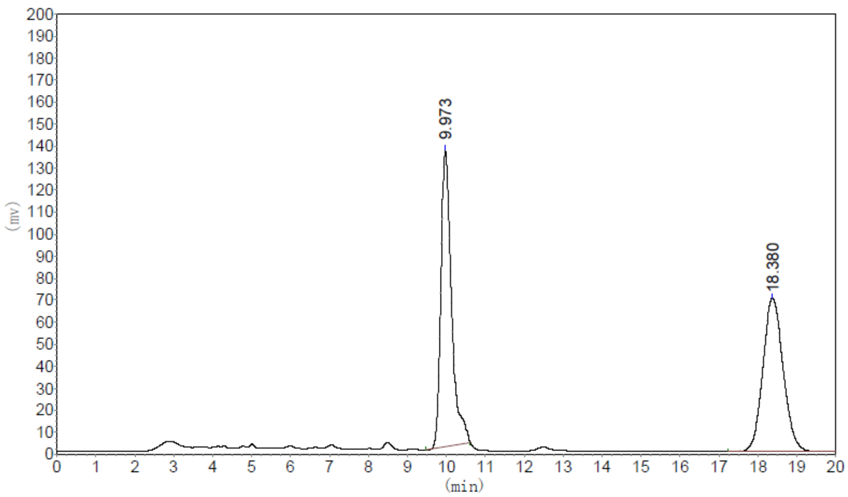

Peak No.	R. Time	Peak Height	Peak Area	Percent
1	9. 973	134969. 500	2558743. 250	50. 4761
2	18. 380	69697. 734	2510473. 750	49. 5239
Total		204667. 234	5069217. 000	100. 0000

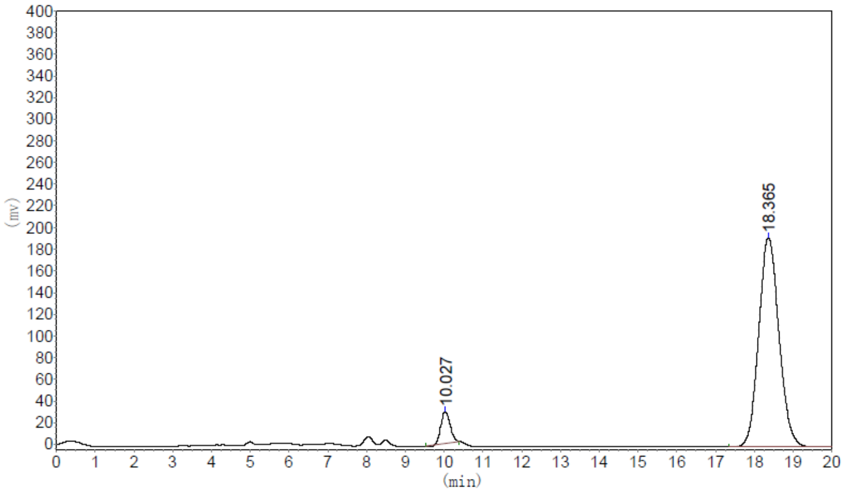

Peak No.	R. Time	Peak Height	Peak Area	Percent
1	10. 027	29407. 998	471316. 719	6. 3237
2	18. 365	193138. 531	6981887. 000	93. 6763
Total		222546. 529	7453203. 719	100. 0000

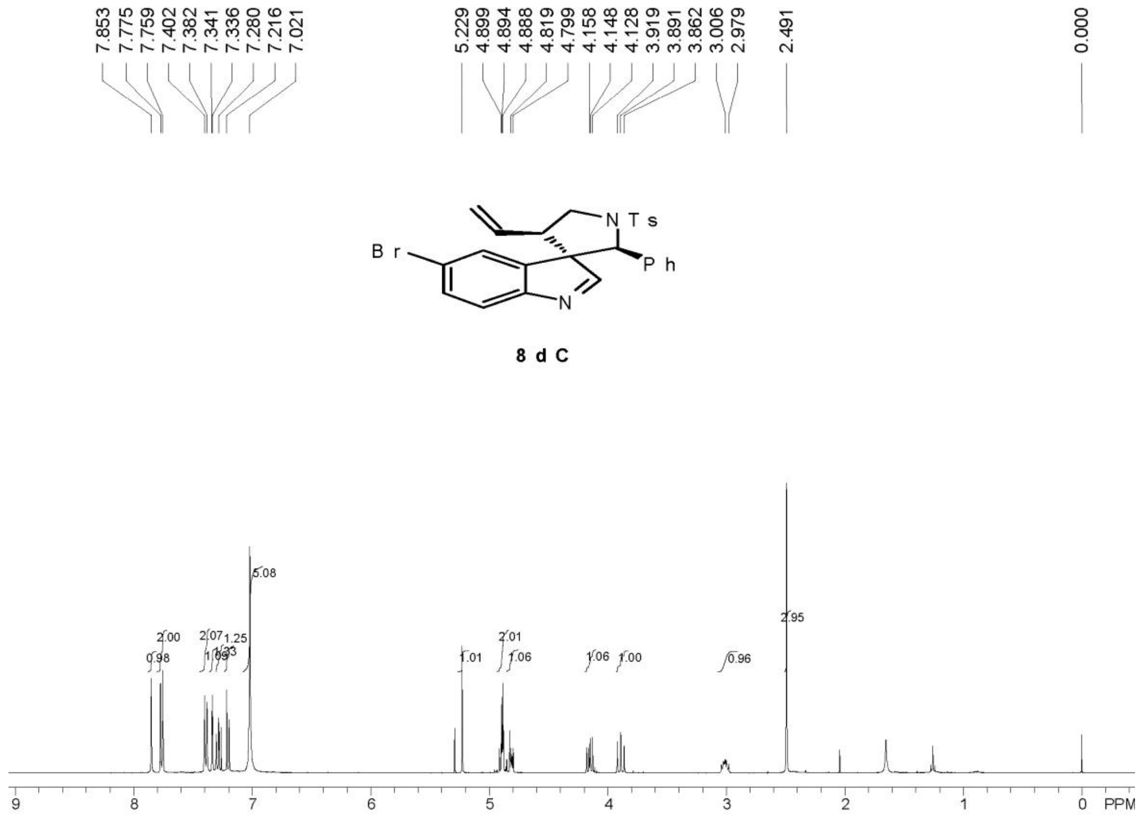

8dC

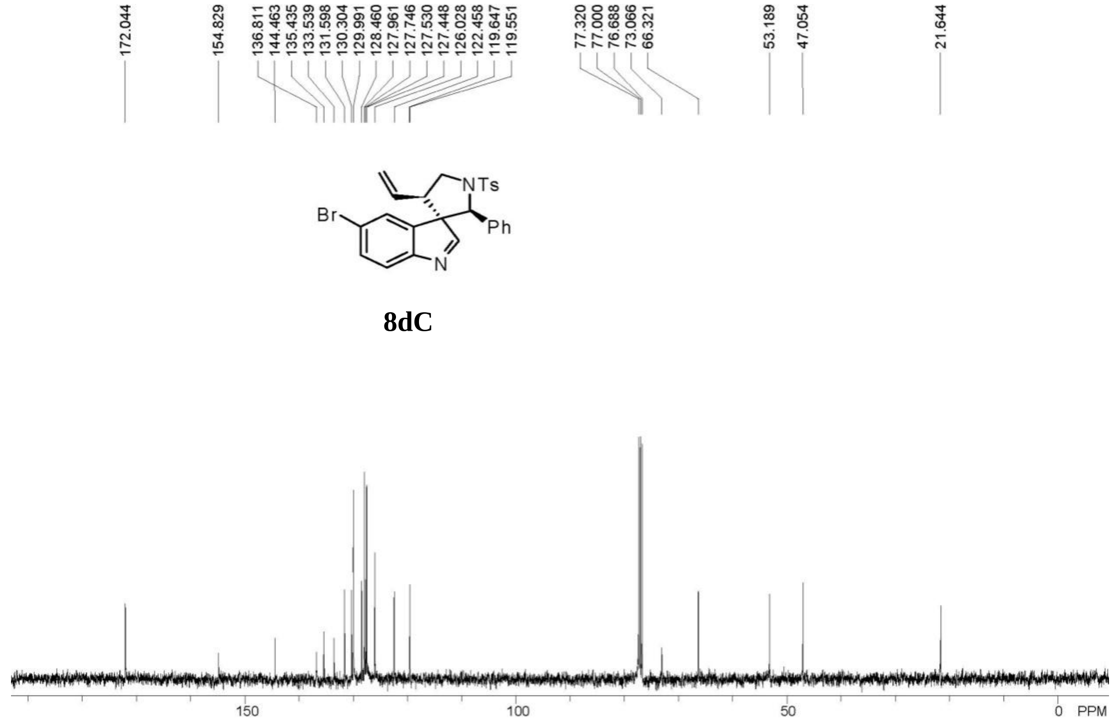

8dC

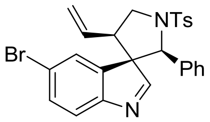

8dC

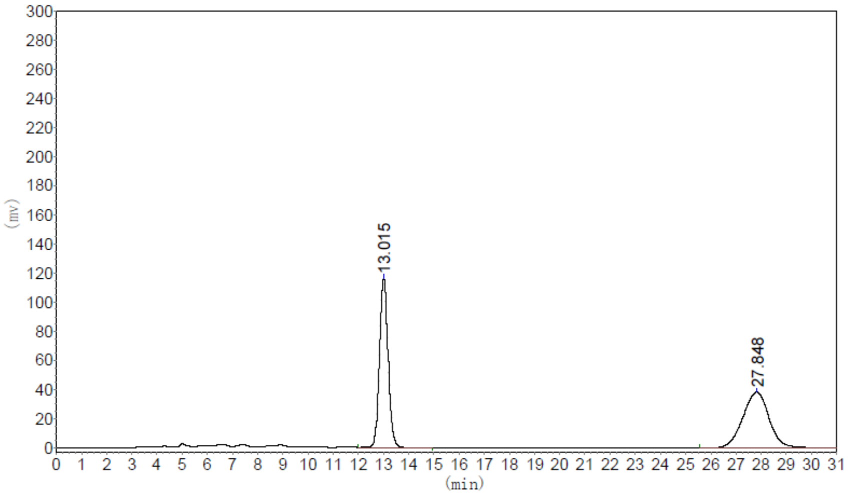

Peak No.	R. Time	Peak Height	Peak Area	Percent
1	13. 015	116158. 125	2752610. 500	50. 1257
2	27. 848	37852. 113	2738809. 250	49. 8743
Total		154010. 238	5491419. 750	100. 0000

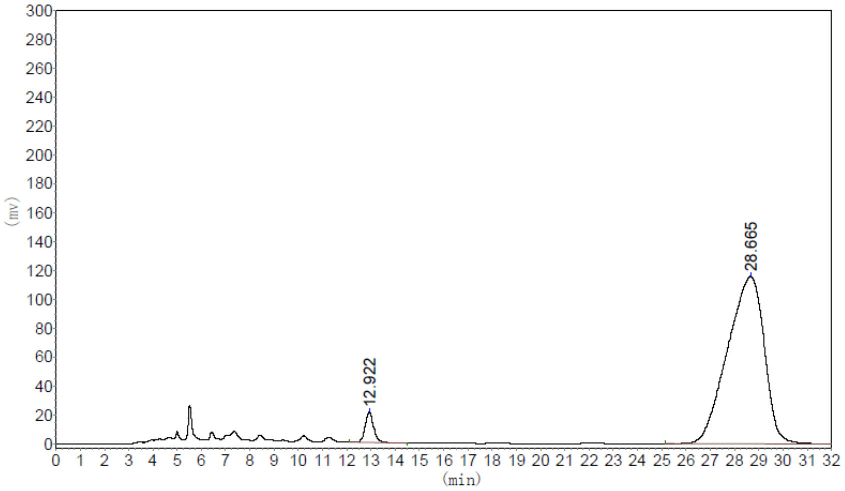

Peak No.	R. Time	Peak Height	Peak Area	Percent
1	12. 922	20890. 098	513834. 906	4. 0389
2	28. 665	115785. 063	12208291. 000	95. 9611
Total		136675. 160	12722125. 906	100. 0000

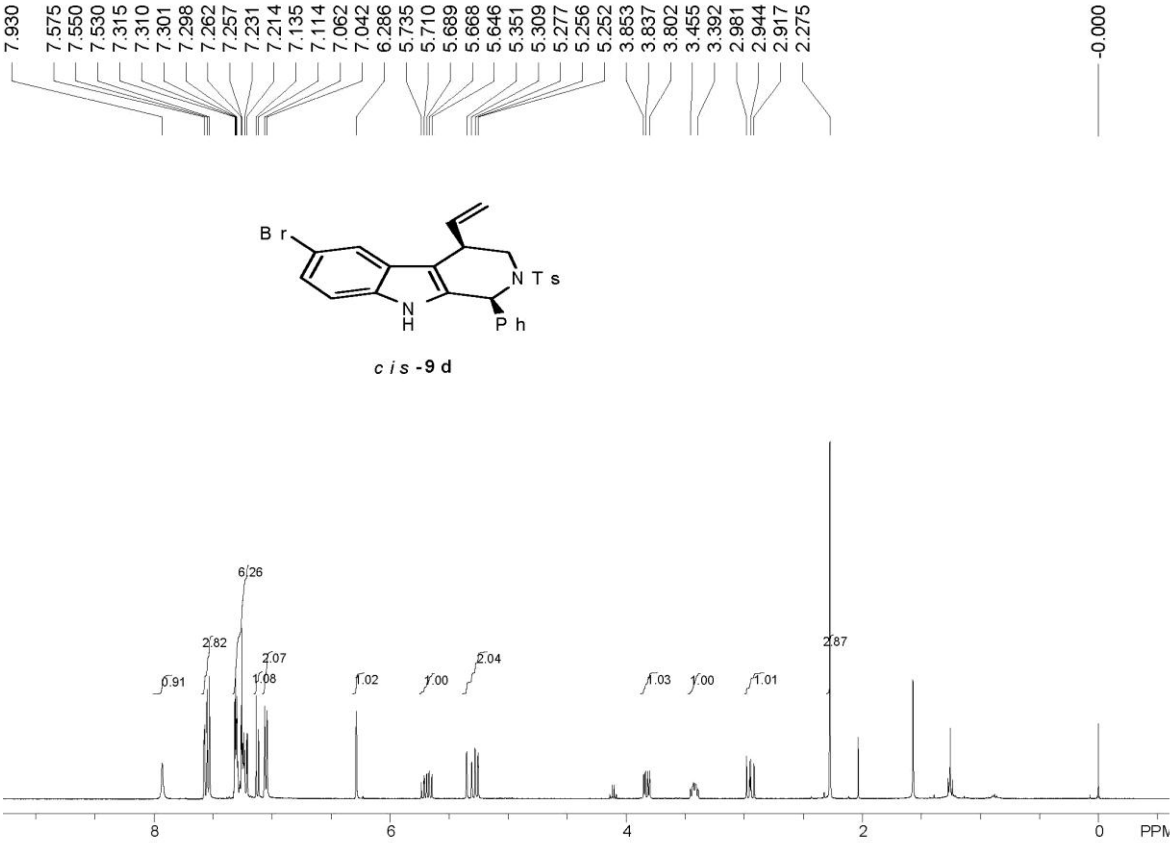
7.930
7.575
7.550
7.530
7.315
7.310
7.301
7.298
7.262
7.257
7.231
7.214
7.135
7.114
7.062
7.042
6.286
5.735
5.710
5.689
5.668
5.646
5.351
5.309
5.277
5.256
5.252
3.853
3.837
3.802
3.455
3.392
2.981
2.944
2.917
2.275
-0.000
Br
NTs
N
H
Ph
cis-9d
6.26
2.82
2.07
1.08
0.91
1.02
1.00
2.04
1.03
1.00
1.01
2.87
8
6
4
2
0
PPM

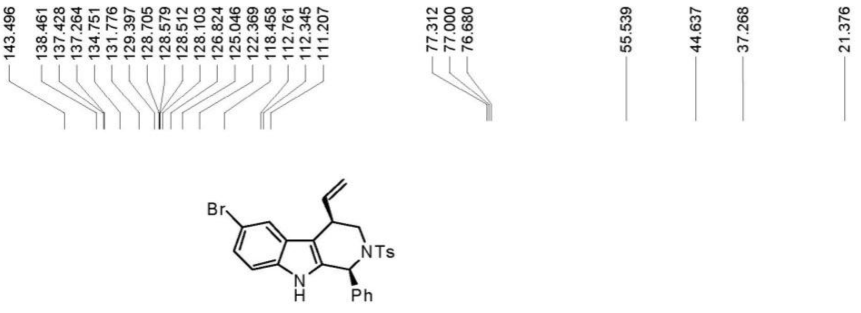
143.496
138.461
137.428
137.264
134.751
131.776
129.397
128.705
128.579
128.512
128.103
126.824
125.046
122.369
118.458
112.761
112.345
111.207
77.312
77.000
76.680
55.539
44.637
37.268
21.376
Br
NTs
N
H
Ph

***cis*-9d**

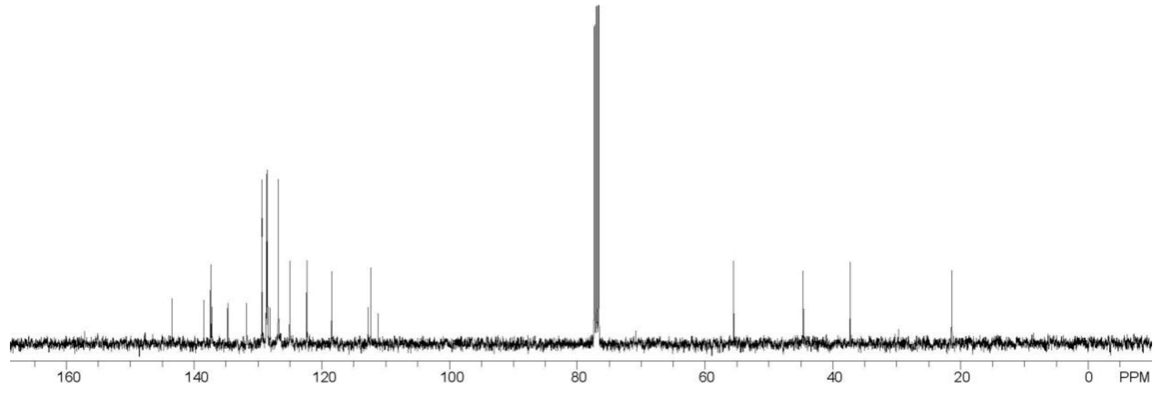
160
140
120
100
80
60
40
20
0
PPM

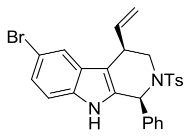

***cis*-9d**

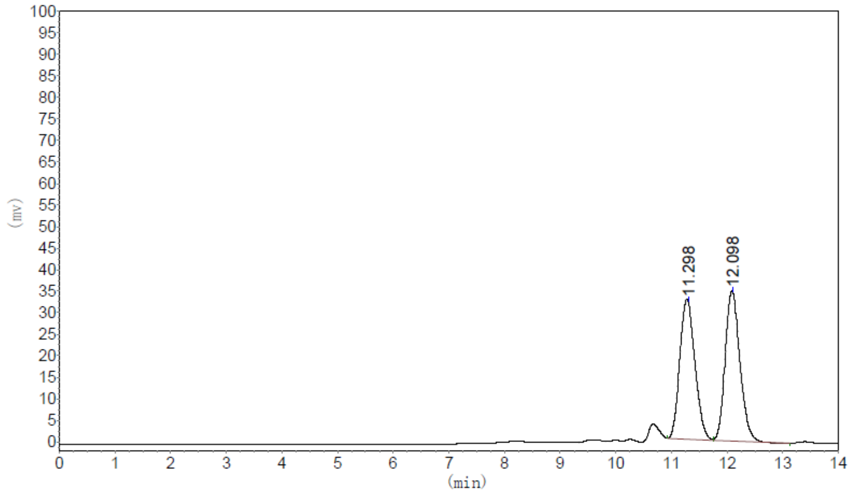

Peak No.	R. Time	Peak Height	Peak Area	Percent
1	11. 298	33242. 793	656401. 313	49. 6244
2	12. 098	35162. 344	666338. 438	50. 3756
Total		68405. 137	1322739. 750	100. 0000

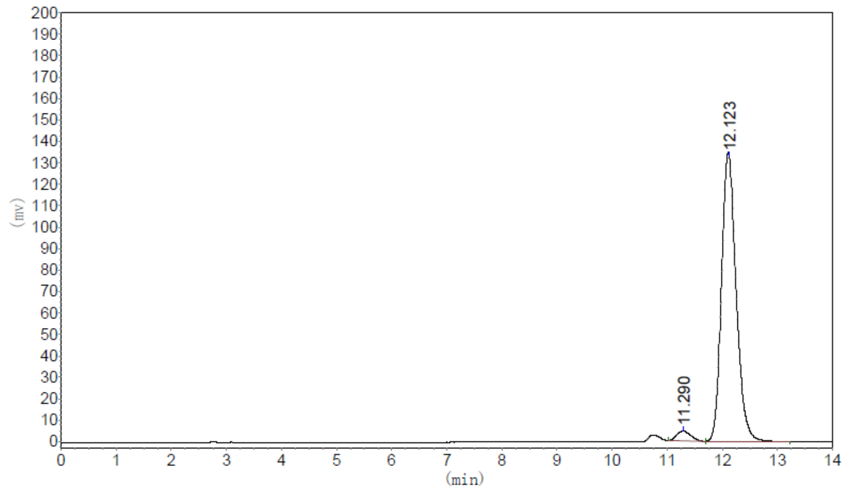

Peak No.	R. Time	Peak Height	Peak Area	Percent
1	11. 290	5160. 180	100787. 461	3. 8721
2	12. 123	134414. 922	2502115. 000	96. 1279
Total		139575. 102	2602902. 461	100. 0000

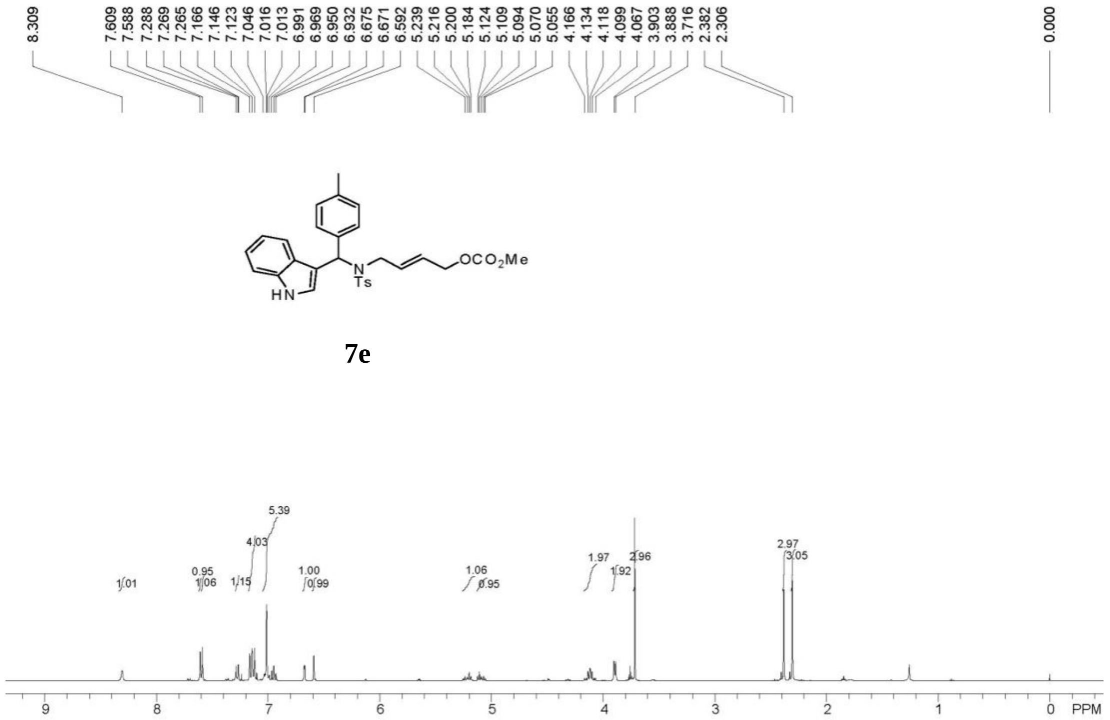
8.309
7.609
7.588
7.288
7.269
7.265
7.166
7.146
7.123
7.046
7.016
7.013
6.991
6.969
6.950
6.932
6.675
6.671
6.592
5.239
5.216
5.200
5.184
5.124
5.109
5.094
5.070
5.055
4.166
4.134
4.118
4.099
4.067
3.903
3.888
3.716
2.382
2.306
0.000
HN
N
Ts
OCO2Me
1.01
0.95
1.06
1.15
4.03
5.39
1.00
0.99
1.06
0.95
1.97
1.92
2.96
2.97
3.05
9
8
7
6
5
4
3
2
1
0 PPM

7e

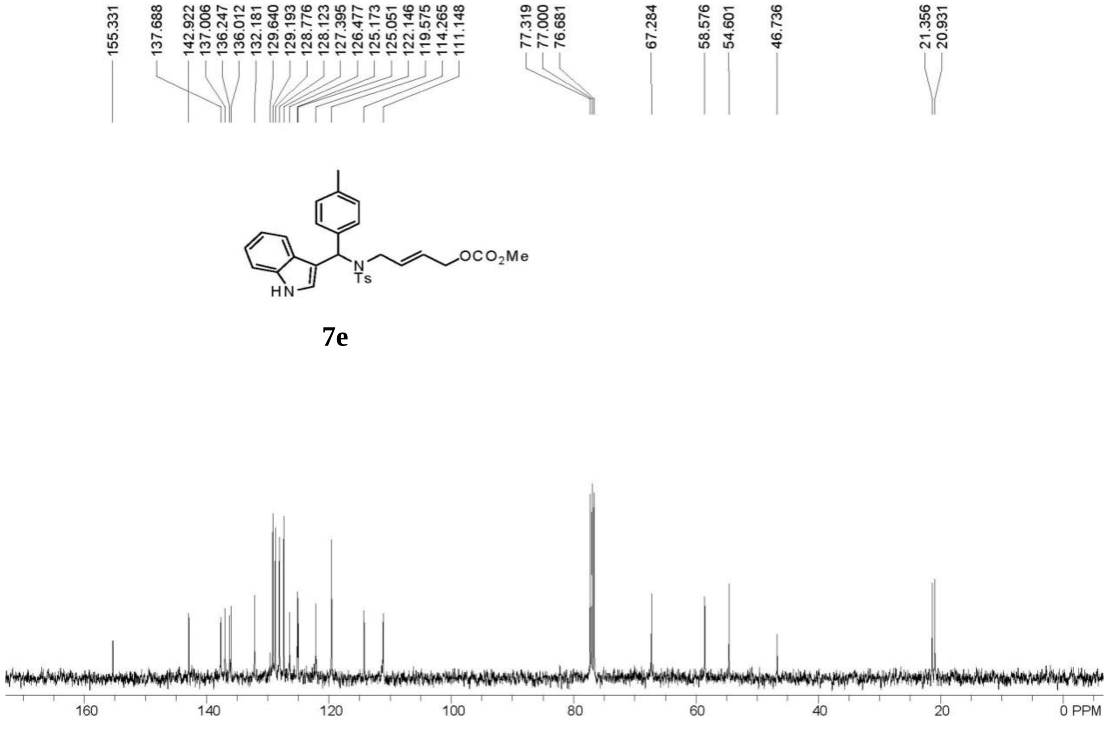
155.331
137.688
142.922
137.006
136.247
136.012
132.181
129.640
129.193
128.776
128.123
127.395
126.477
125.173
125.051
122.146
119.575
114.265
111.148
77.319
77.000
76.681
67.284
58.576
54.601
46.736
21.356
20.931
HN
N
Ts
OCO2Me
160
140
120
100
80
60
40
20
0 PPM

7e

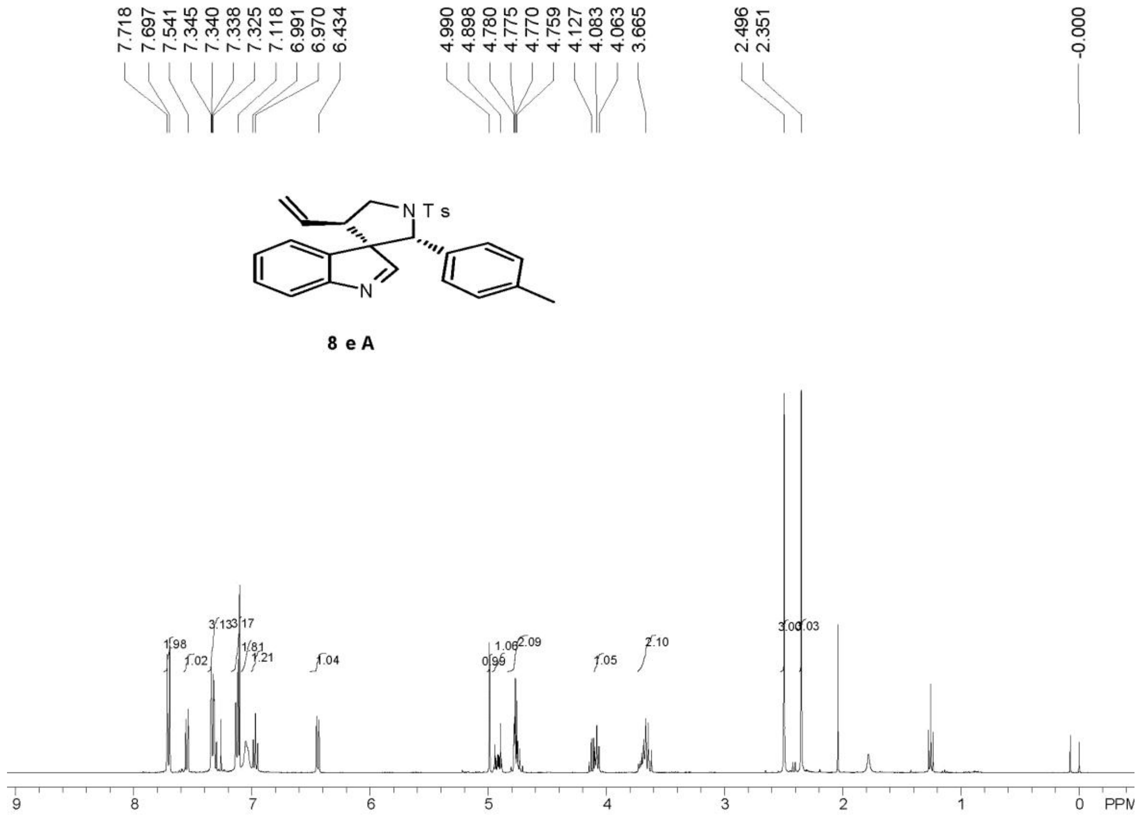
7.718
7.697
7.541
7.345
7.340
7.338
7.325
7.118
6.991
6.970
6.434
4.990
4.898
4.780
4.775
4.770
4.759
4.127
4.083
4.063
3.665
2.496
2.351
-0.000
NTs
N
1.98
1.02
3.13
3.17
1.81
1.21
1.04
0.99
1.06
2.09
1.05
2.10
3.00
3.03
PPM

8 e A

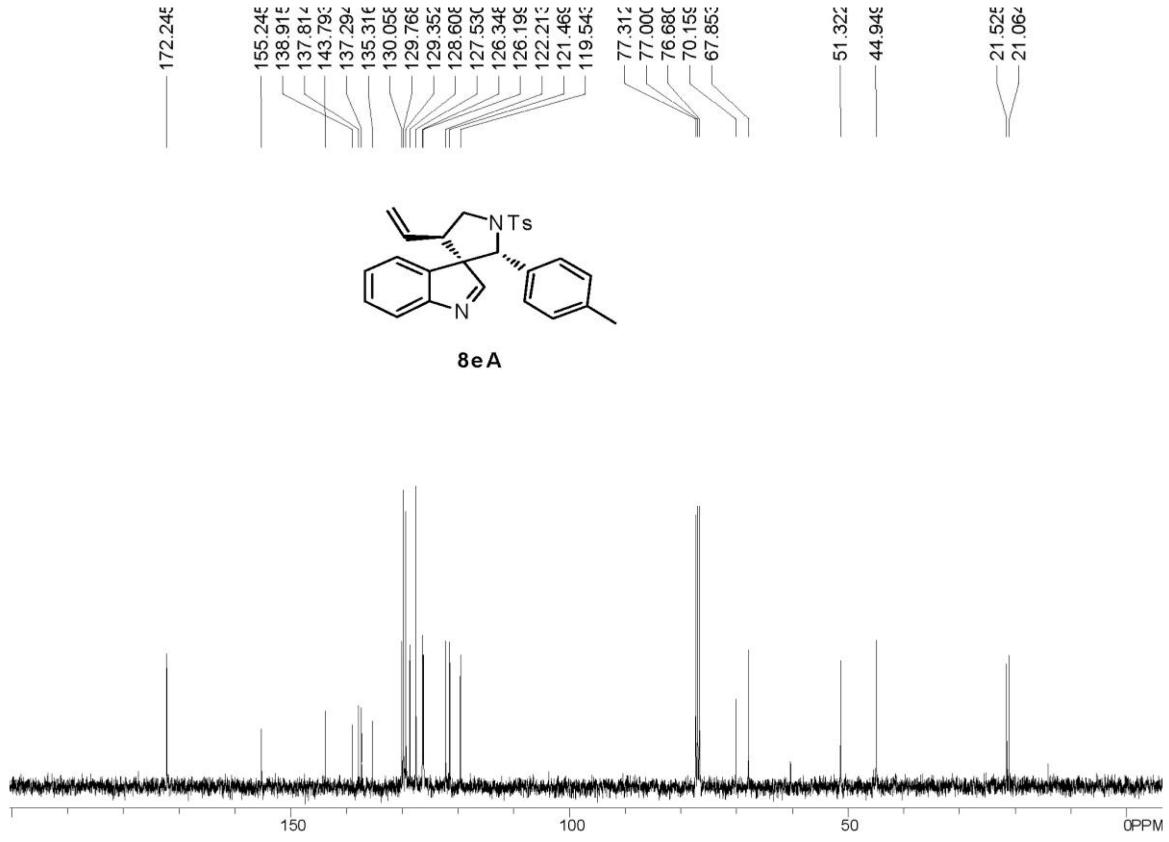
172.245
155.245
138.915
137.814
143.793
137.294
135.316
130.058
129.768
129.352
128.608
127.530
126.348
126.199
122.213
121.469
119.543
77.312
77.000
76.680
70.159
67.853
51.322
44.949
21.525
21.064
NTs
N
150
100
50
0PPM

8eA

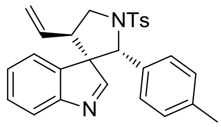

8eA

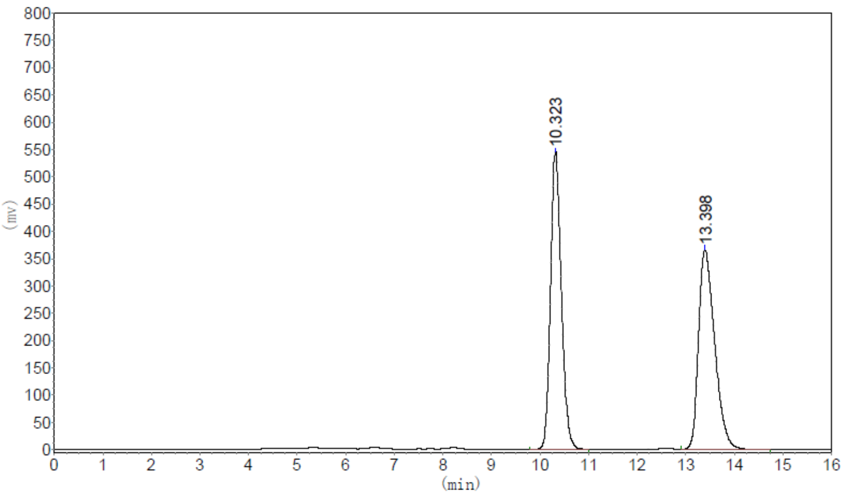

Peak No.	R. Time	Peak Height	Peak Area	Percent
1	10. 323	544920. 750	8426328. 000	49. 9966
2	13. 398	365959. 063	8427465. 000	50. 0034
Total		910879. 813	16853793. 000	100. 0000

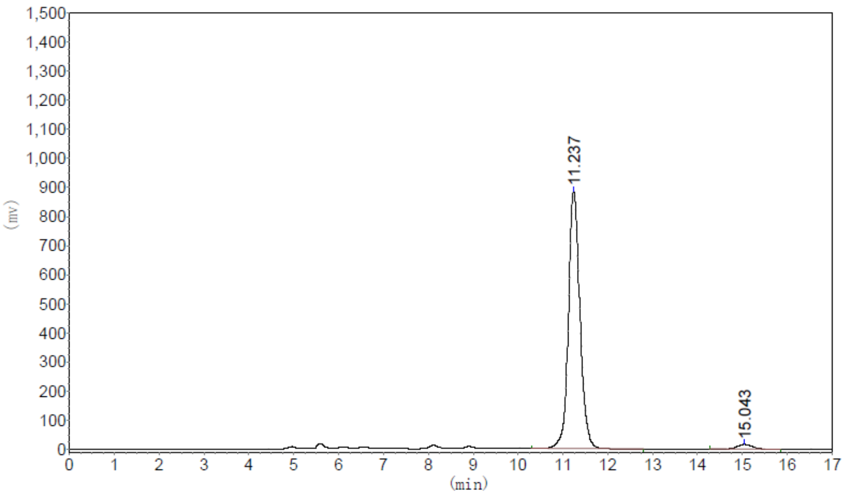

Peak No.	R. Time	Peak Height	Peak Area	Percent
1	11. 237	884784. 688	15739369. 000	97. 5497
2	15. 043	16028. 739	395341. 688	2. 4503
Total		900813. 427	16134710. 688	100. 0000

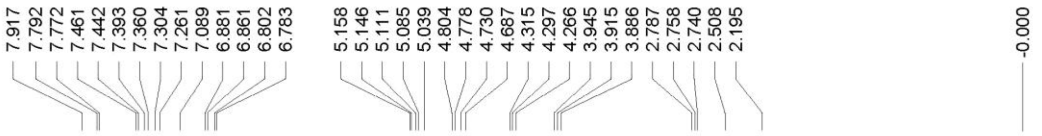
7.917
7.792
7.772
7.461
7.442
7.393
7.360
7.304
7.261
7.089
6.881
6.861
6.802
6.783
5.158
5.146
5.111
5.085
5.039
4.804
4.778
4.730
4.687
4.315
4.297
4.266
3.945
3.915
3.886
2.787
2.758
2.740
2.508
2.195
-0.000

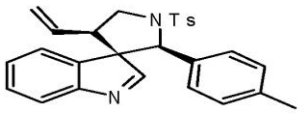
N Ts
N

8 e B

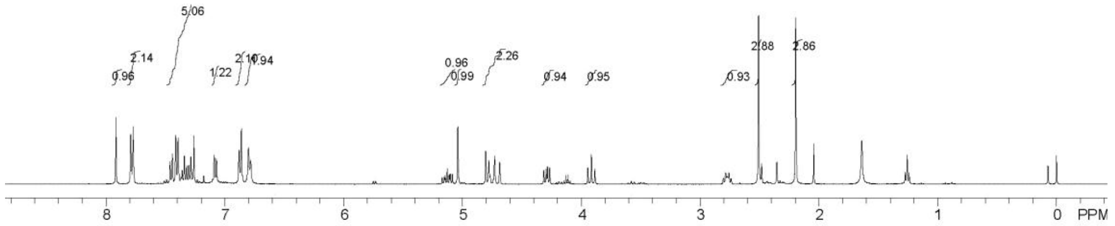
5.06
2.14
0.96
1.22
2.10
0.94
0.96
0.99
2.26
0.94
0.95
0.93
2.88
2.86
8
7
6
5
4
3
2
1
0
PPM

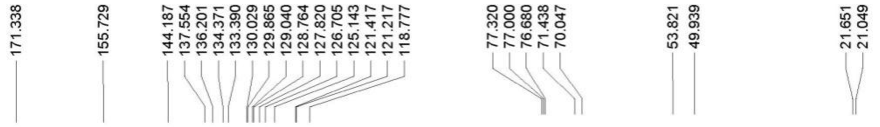
171.338
155.729
144.187
137.554
136.201
134.371
133.390
130.029
129.865
129.040
128.764
127.820
126.705
125.143
121.417
121.217
118.777
77.320
77.000
76.680
71.438
70.047
53.821
49.939
21.651
21.049

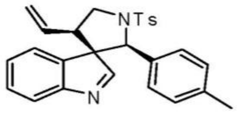
NTs
N

8eB

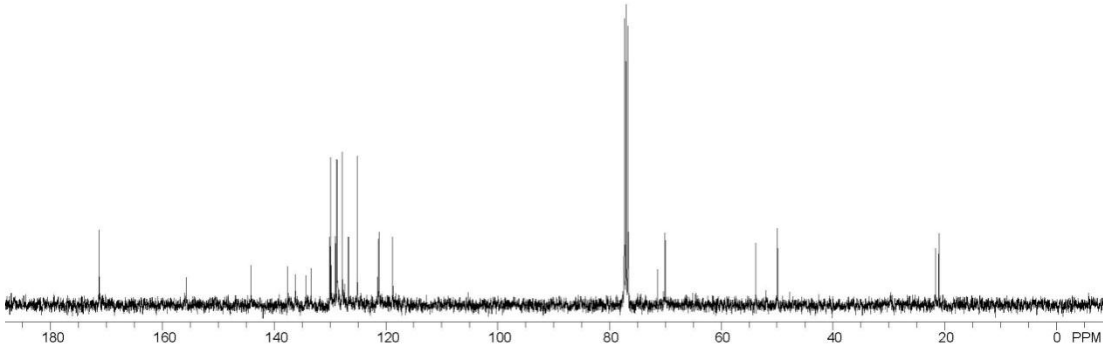
180
160
140
120
100
80
60
40
20
0
PPM

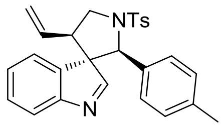

8eB

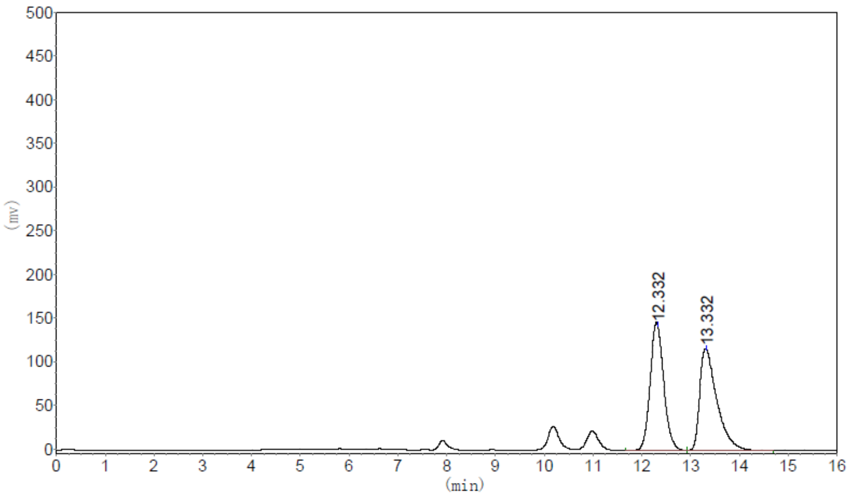

Peak No.	R. Time	Peak Height	Peak Area	Percent
1	12. 332	144673. 406	2821789. 250	49. 7228
2	13. 332	115939. 398	2853250. 500	50. 2772
Total		260612. 805	5675039. 750	100. 0000

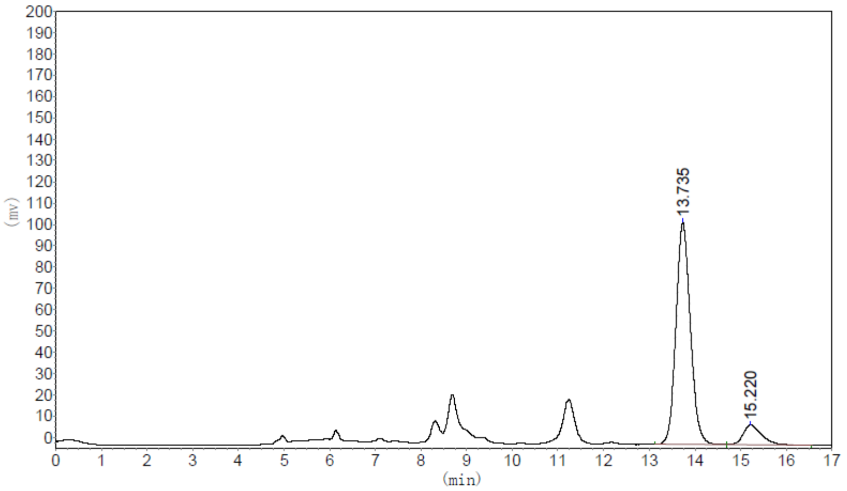

Peak No.	R. Time	Peak Height	Peak Area	Percent
1	13. 735	104133. 477	2270890. 250	89. 7503
2	15. 220	9089. 313	259342. 063	10. 2497
Total		113222. 790	2530232. 313	100. 0000

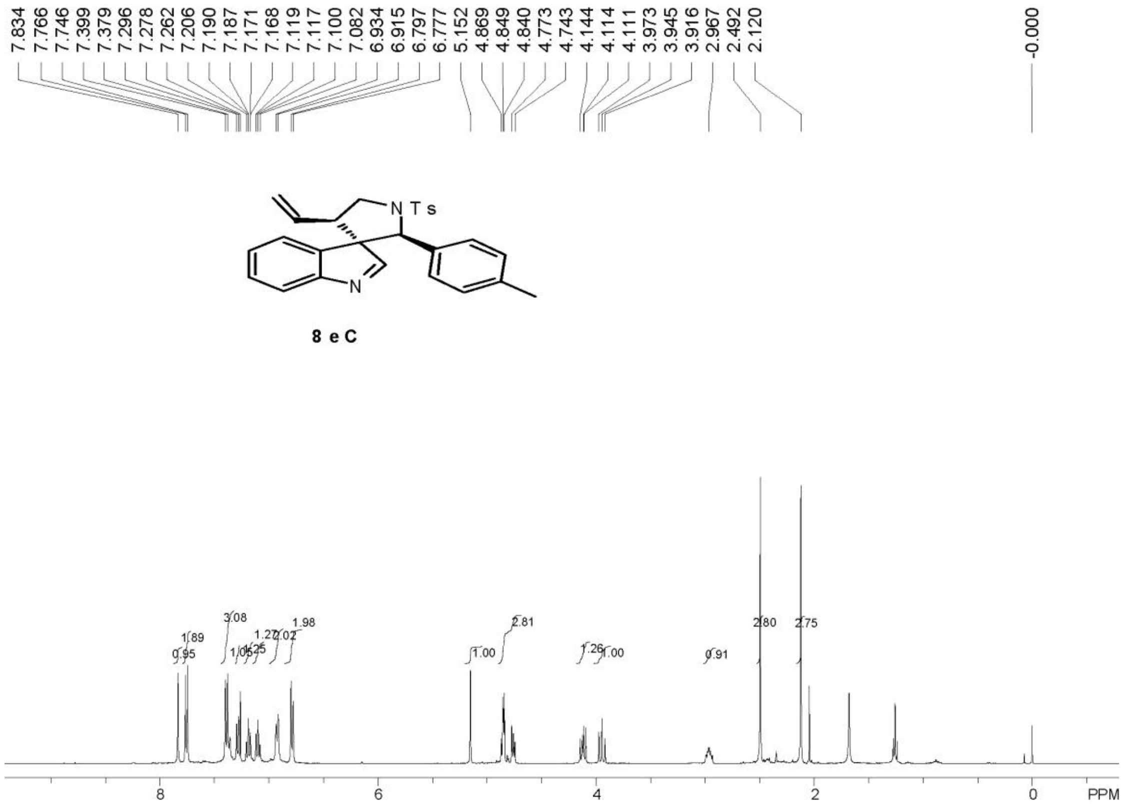

8 e C

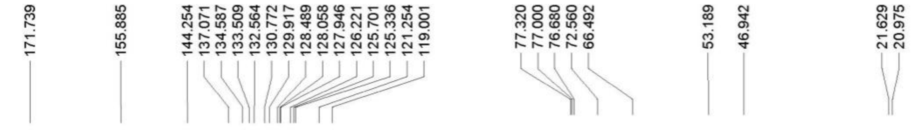

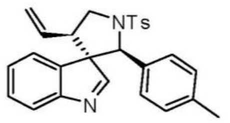

8eC

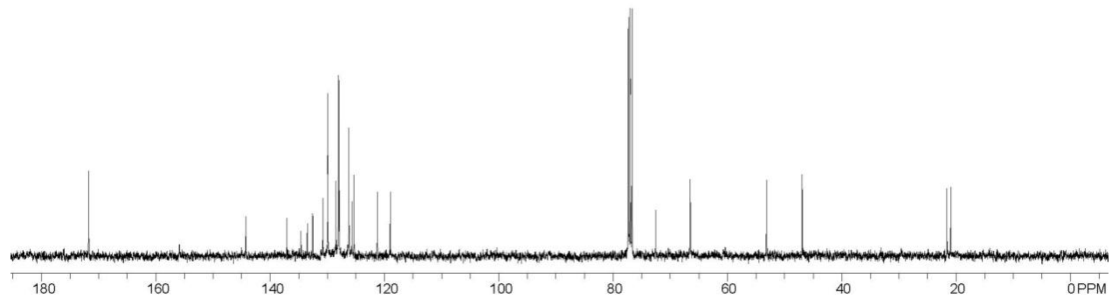

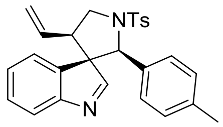

8eC

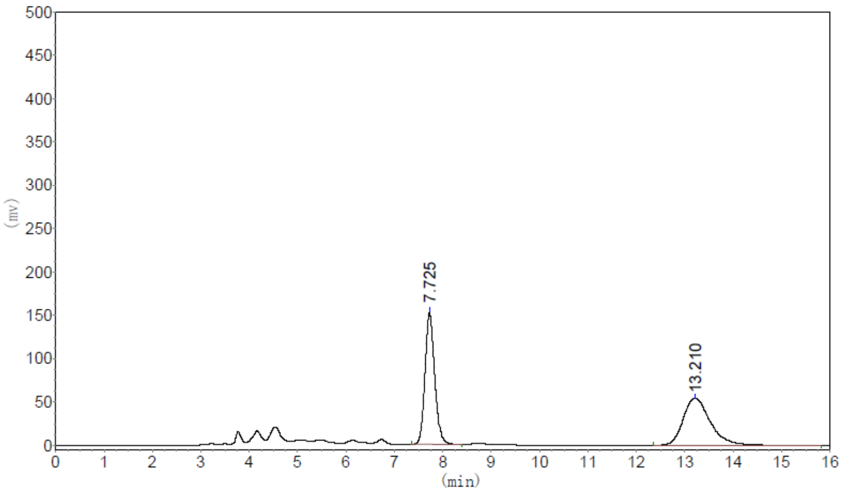

Peak No.	R. Time	Peak Height	Peak Area	Percent
1	7.725	152424.781	2122315.750	50.2289
2	13.210	54494.688	2102971.750	49.7711
Total		206919.469	4225287.500	100.0000

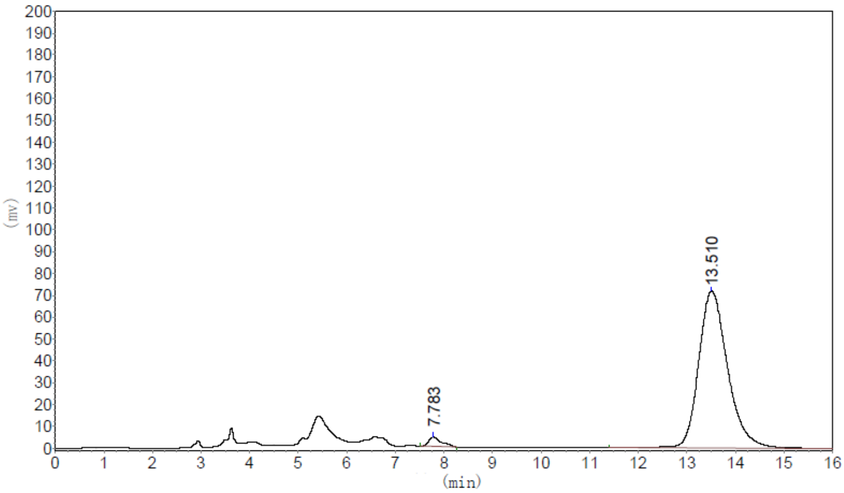

Peak No.	R. Time	Peak Height	Peak Area	Percent
1	7.783	4411.198	81080.898	2.6249
2	13.510	71900.344	3007857.250	97.3751
Total		76311.542	3088938.148	100.0000

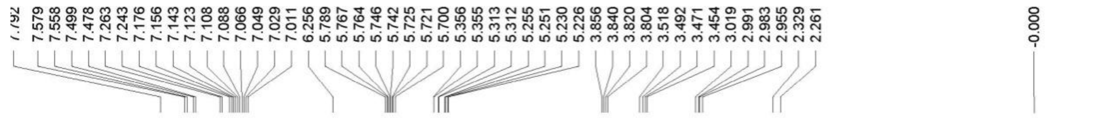
7.792
7.579
7.558
7.499
7.478
7.263
7.243
7.176
7.156
7.143
7.123
7.108
7.088
7.066
7.049
7.029
7.011
6.256
5.789
5.767
5.764
5.746
5.742
5.725
5.721
5.700
5.356
5.355
5.313
5.312
5.255
5.251
5.230
5.226
3.856
3.840
3.820
3.804
3.518
3.492
3.471
3.454
3.019
2.991
2.983
2.955
2.329
2.261
-0.000

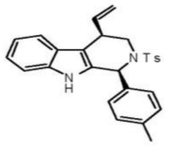
N Ts
N
H

***cis*-9e**

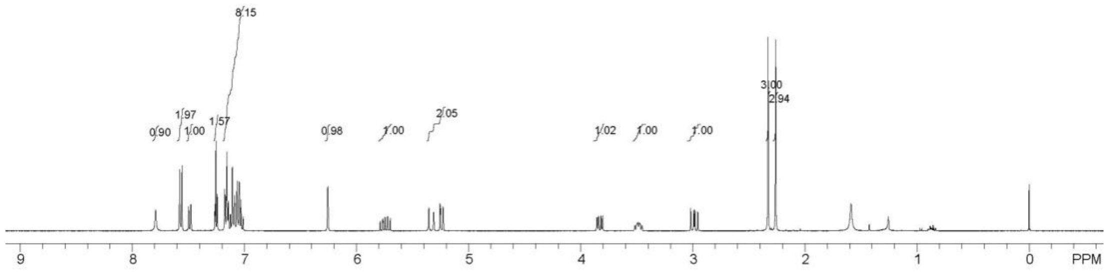
8.15
1.97
0.90
1.00
1.57
0.98
1.00
2.05
1.02
1.00
1.00
3.00
2.94
9
8
7
6
5
4
3
2
1
0
PPM

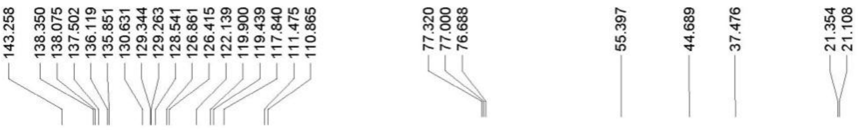
143.258
138.350
138.075
137.502
136.119
135.851
130.631
129.344
129.263
128.541
126.861
126.415
122.139
119.900
119.439
117.840
111.475
110.865
77.320
77.000
76.688
55.397
44.689
37.476
21.354
21.108

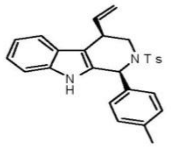
N Ts
N
H

***cis*-9e**

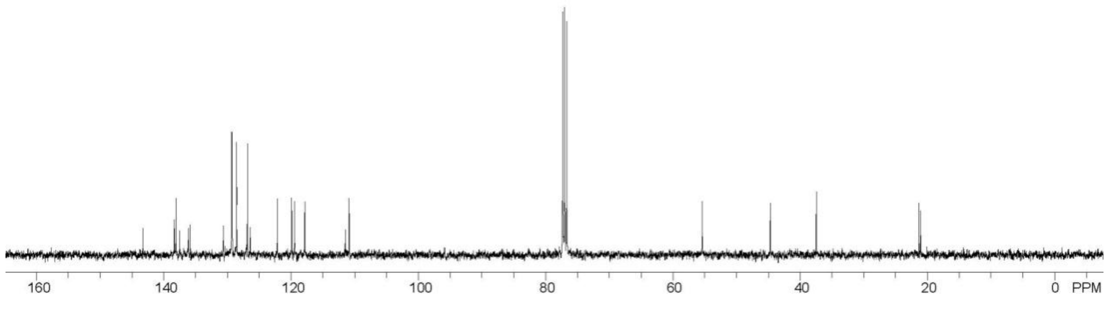
160
140
120
100
80
60
40
20
0
PPM

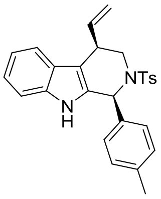

***cis*-9e**

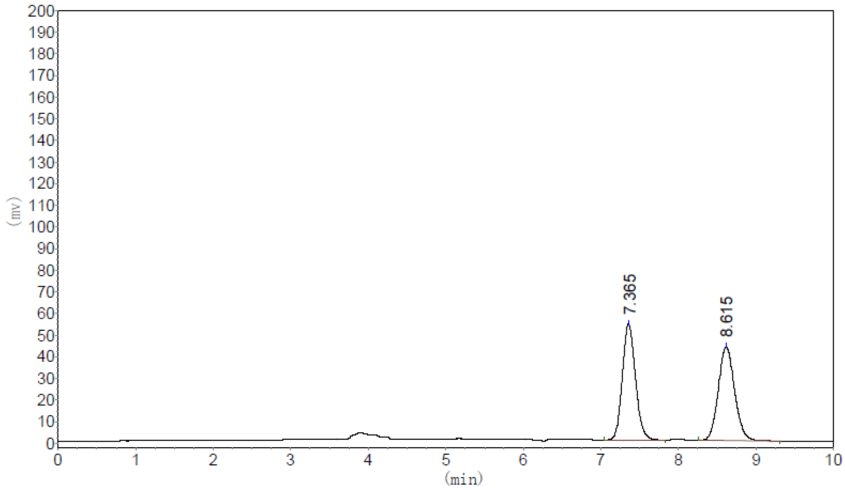

Peak No.	R. Time	Peak Height	Peak Area	Percent
1	7. 365	53870. 930	649361. 563	50. 2397
2	8. 615	43172. 000	643165. 875	49. 7603
Total		97042. 930	1292527. 438	100. 0000

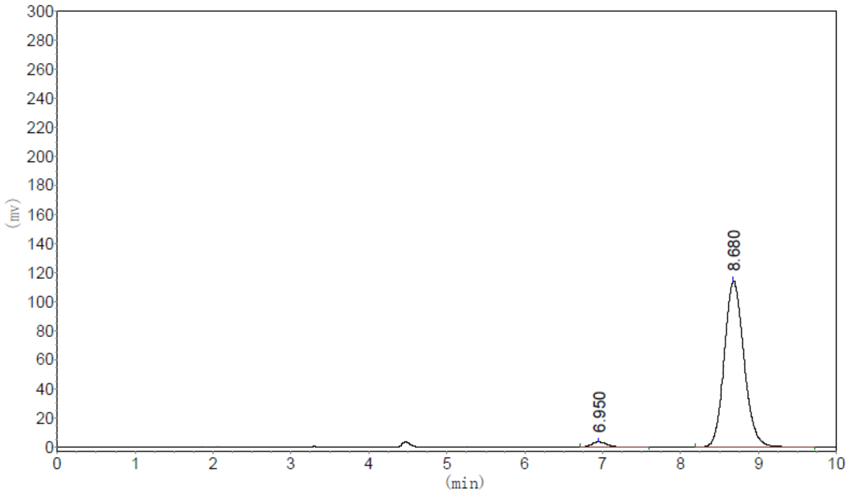

Peak No.	R. Time	Peak Height	Peak Area	Percent
1	6. 950	3264. 515	43214. 301	2. 1157
2	8. 680	114018. 180	1999304. 500	97. 8843
Total		117282. 695	2042518. 801	100. 0000

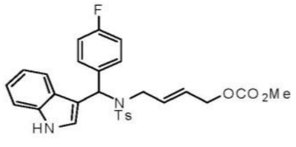

7f

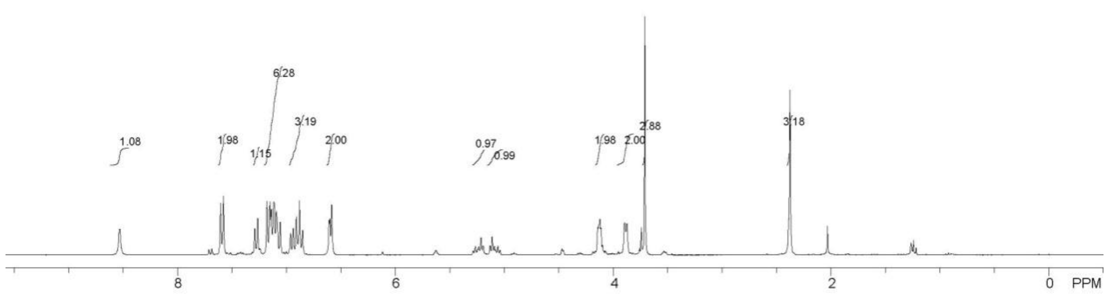
8.551
7.627
7.599
7.312
7.286
7.200
7.174
7.114
7.079
6.984
6.931
6.902
6.873
6.630
6.607
5.287
5.235
5.214
5.152
5.133
5.081
4.163
4.144
4.135
4.120
3.916
3.896
3.731
2.399
-0.000
1.08
1.98
1.15
6.28
3.19
2.00
0.97
0.99
1.98
2.00
2.88
3.18
8
6
4
2
0
PPM

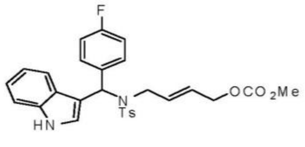

7f

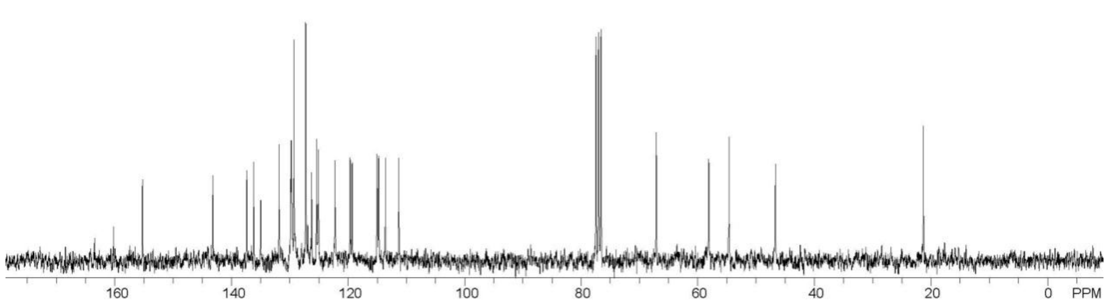
163.488
160.224
155.263
137.352
143.174
136.183
134.960
131.818
129.796
129.692
129.269
127.246
126.235
125.385
125.070
122.213
119.629
119.264
115.011
114.726
113.580
111.261
77.423
77.000
76.573
67.083
58.073
54.601
46.672
21.305
160
140
120
100
80
60
40
20
0
PPM

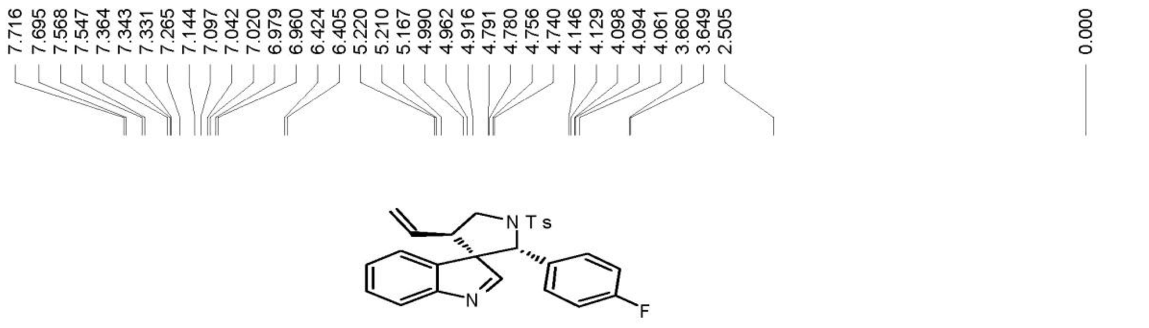
7.716
7.695
7.568
7.547
7.364
7.343
7.331
7.265
7.144
7.097
7.042
7.020
6.979
6.960
6.424
6.405
5.220
5.210
5.167
4.990
4.962
4.916
4.791
4.780
4.756
4.740
4.146
4.129
4.098
4.094
4.061
3.660
3.649
2.505
0.000
NTs
N
F

8 f A

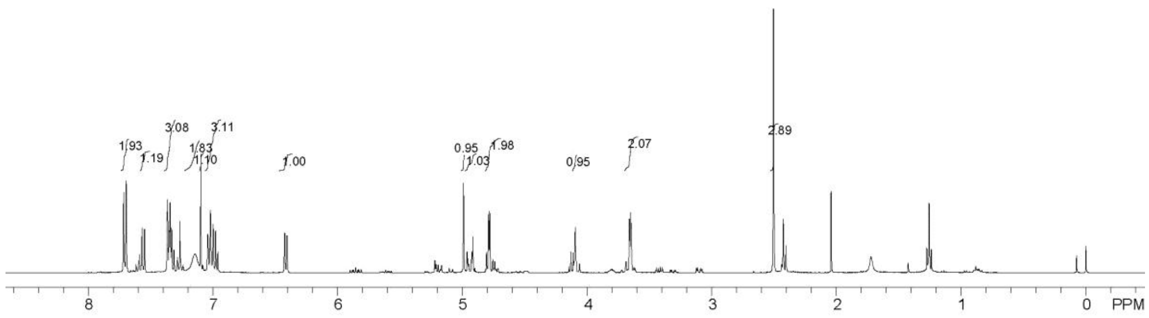
1.93
1.19
3.08
1.83
1.10
3.11
1.00
0.95
1.03
1.98
0.95
2.07
2.89
8
7
6
5
4
3
2
1
0
PPM

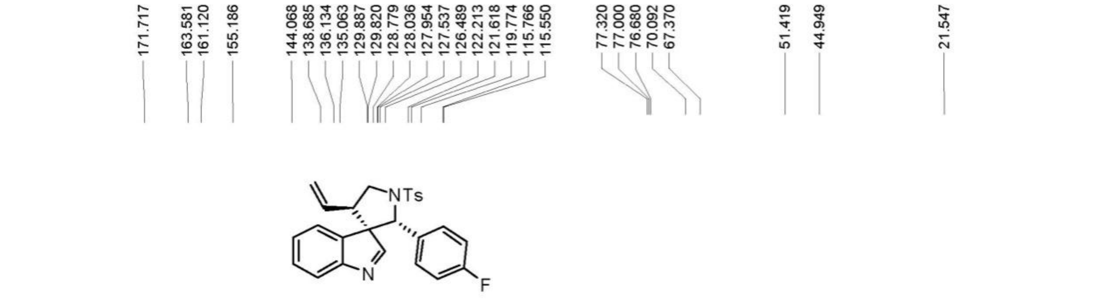
171.717
163.581
161.120
155.186
144.068
138.685
136.134
135.063
129.887
129.820
128.779
128.036
127.954
127.537
126.489
122.213
121.618
119.774
115.766
115.550
77.320
77.000
76.680
70.092
67.370
51.419
44.949
21.547
NTs
N
F

8fA

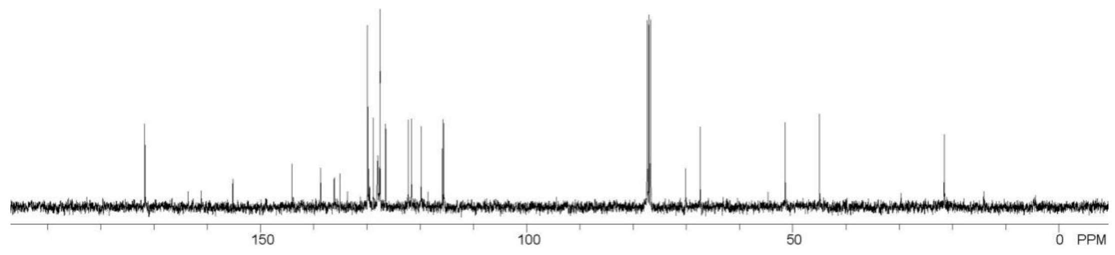
150
100
50
0
PPM

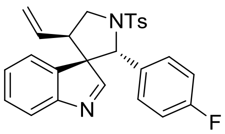

8fA

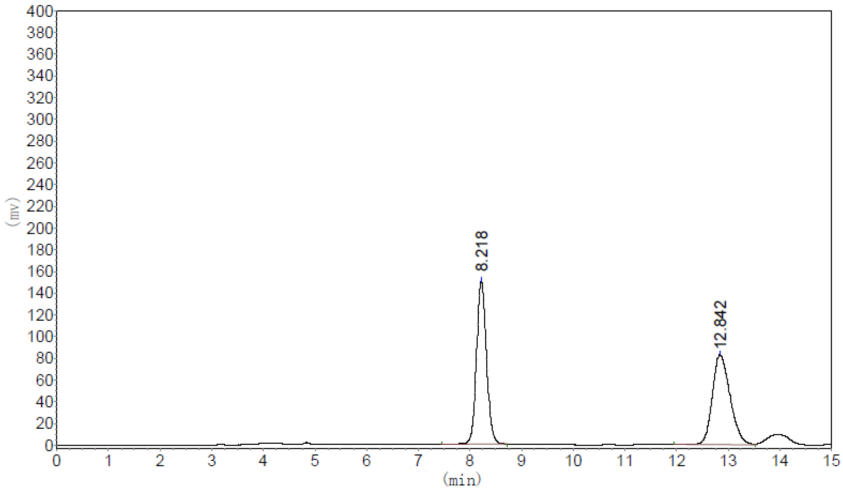

Peak No.	R. Time	Peak Height	Peak Area	Percent
1	8. 218	149884. 656	1999795. 875	50. 4897
2	12. 842	82581. 406	1961005. 500	49. 5103
Total		232466. 063	3960801. 375	100. 0000

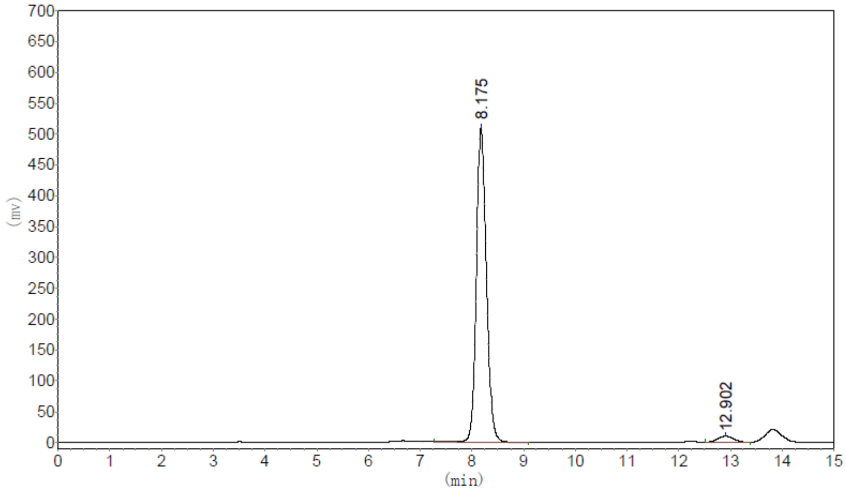

Peak No.	R. Time	Peak Height	Peak Area	Percent
1	8. 175	508400. 188	6885489. 000	96. 9121
2	12. 902	9670. 430	219389. 906	3. 0879
Total		518070. 617	7104878. 906	100. 0000

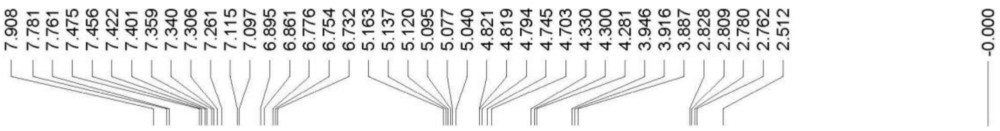
7.908
7.781
7.761
7.475
7.456
7.422
7.401
7.359
7.340
7.306
7.261
7.115
7.097
6.895
6.861
6.776
6.754
6.732
5.163
5.137
5.120
5.095
5.077
5.040
4.821
4.819
4.794
4.745
4.703
4.330
4.300
4.281
3.946
3.916
3.887
2.828
2.809
2.780
2.762
2.512
-0.000

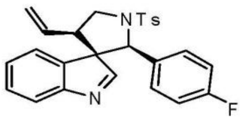
NTs
N
F

8fB

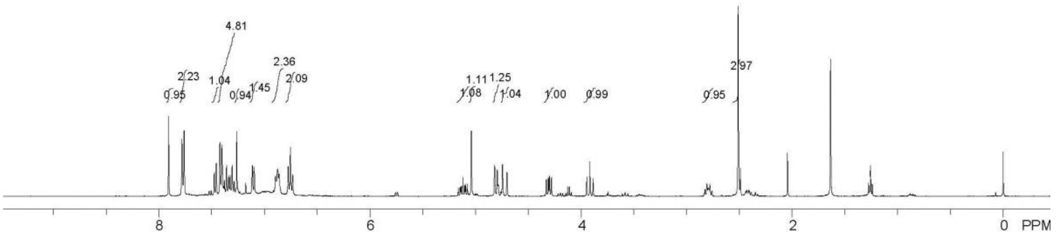
4.81
2.23
1.04
0.95
0.94
1.45
2.36
2.09
1.11
1.08
1.25
1.04
1.00
0.99
0.95
2.97
8
6
4
2
0
PPM

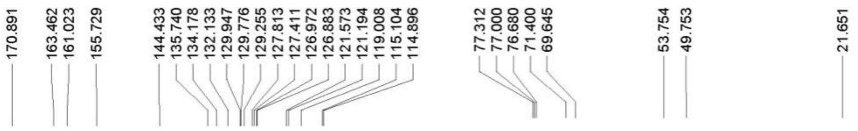
170.891
163.462
161.023
155.729
144.433
135.740
134.178
132.133
129.947
129.776
129.255
127.813
127.411
126.972
126.883
121.573
121.194
119.008
115.104
114.896
77.312
77.000
76.680
71.400
69.645
53.754
49.753
21.651

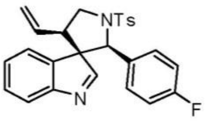
NTs
N
F

8fB

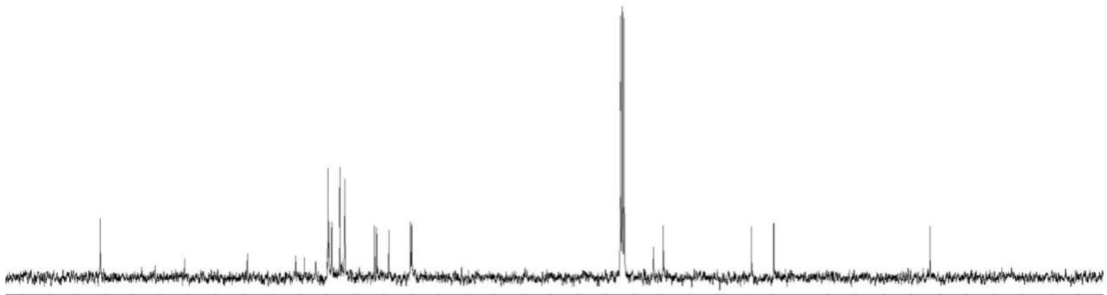
180
160
140
120
100
80
60
40
20
0
PPM

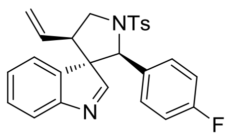

8fB

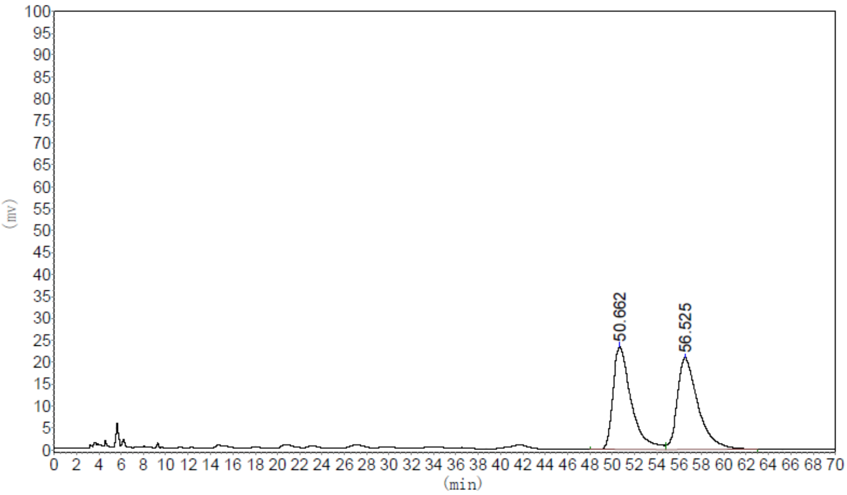

Peak No.	R. Time	Peak Height	Peak Area	Percent
1	50.662	23428.986	2657865.250	49.6198
2	56.525	20875.854	2698594.000	50.3802
Total		44304.840	5356459.250	100.0000

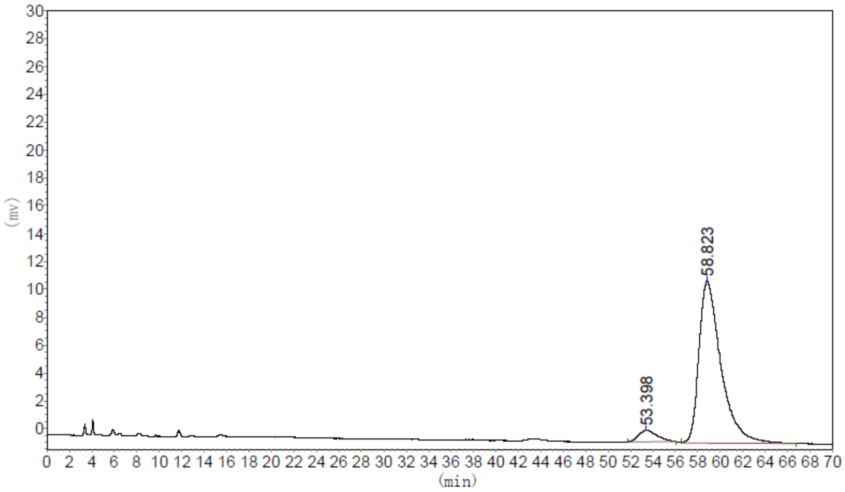

Peak No.	R. Time	Peak Height	Peak Area	Percent
1	53.398	819.390	94707.500	5.6500
2	58.823	11581.508	1581538.250	94.3500
Total		12400.898	1676245.750	100.0000

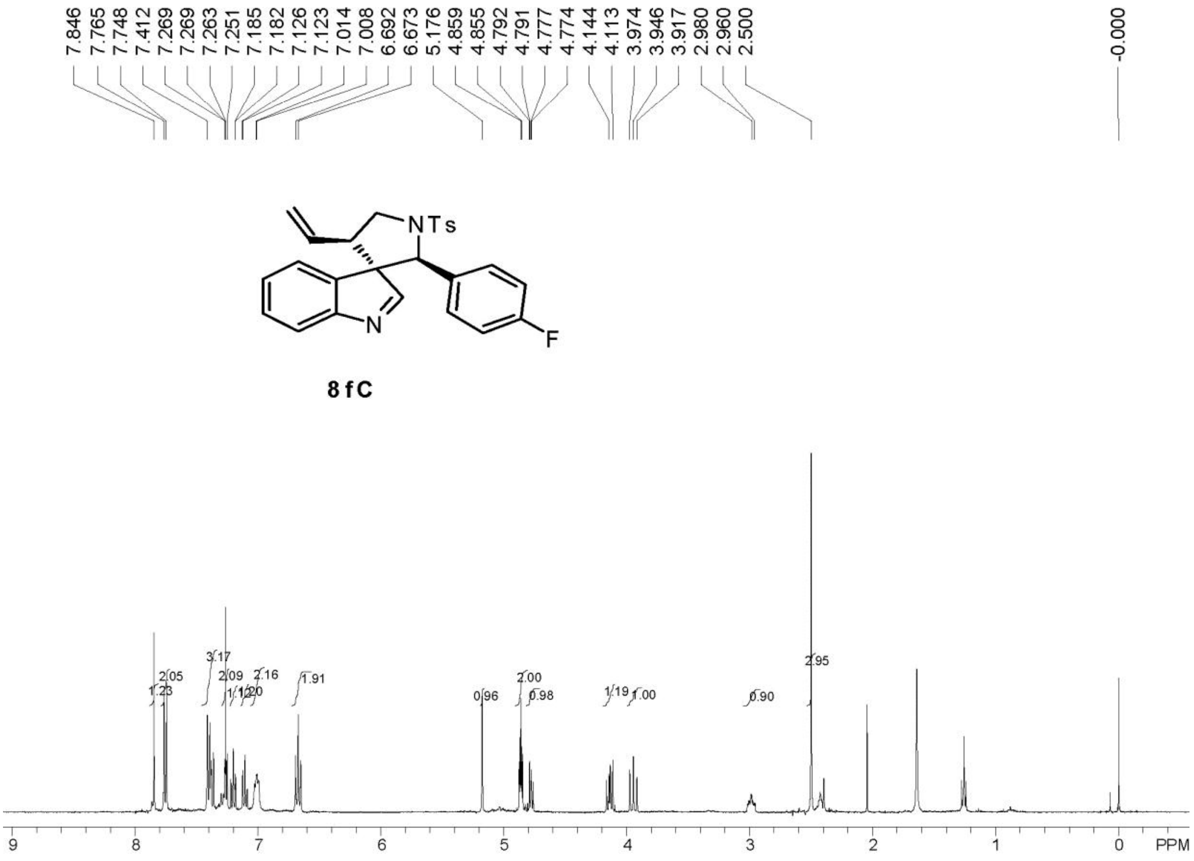
7.846 7.765 7.748 7.412 7.269 7.269 7.263 7.251 7.185 7.182 7.126 7.123 7.014 7.008 6.692 6.673 5.176 4.859 4.855 4.792 4.791 4.777 4.774 4.144 4.113 3.974 3.946 3.917 2.980 2.960 2.500
-0.000
NTs
N
F
8fC
PPM

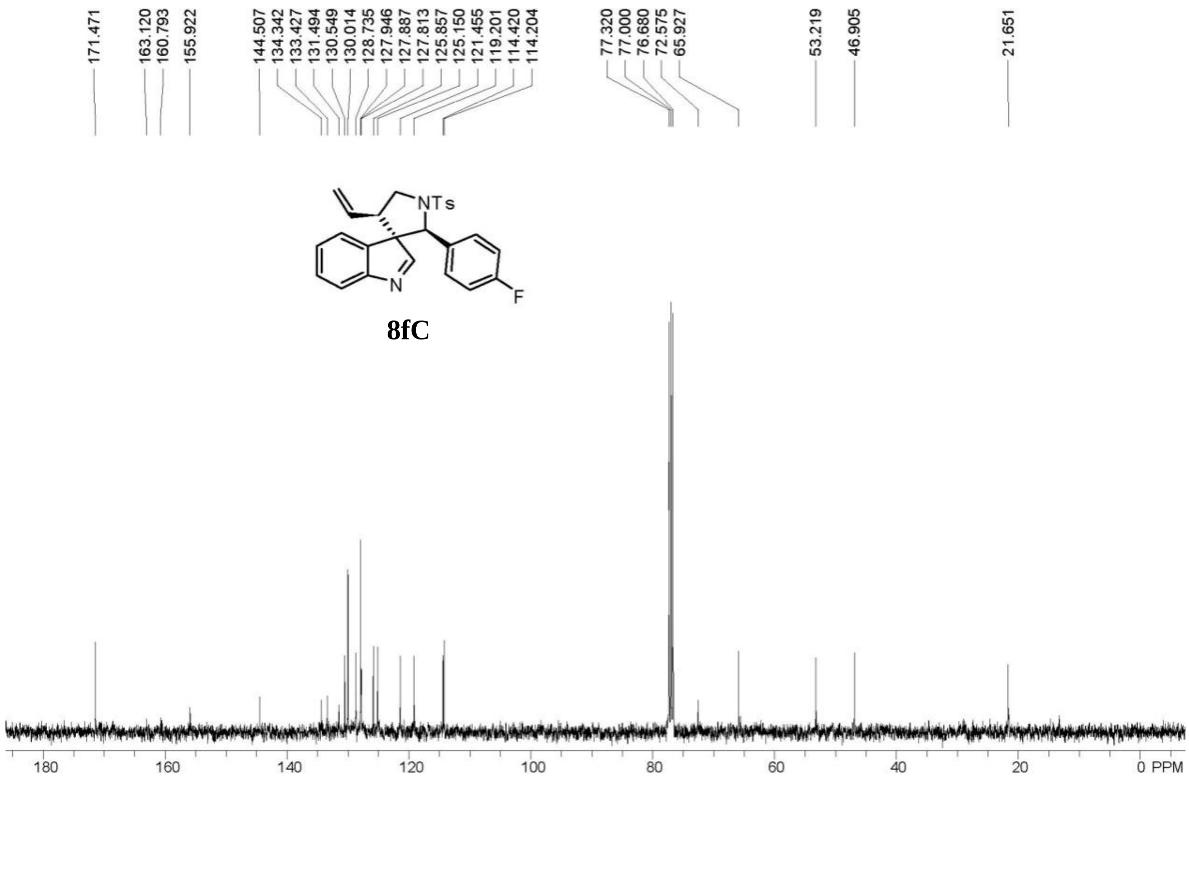
171.471 163.120 160.793 155.922 144.507 134.342 133.427 131.494 130.549 130.014 128.735 127.946 127.887 127.813 125.857 125.150 121.455 119.201 114.420 114.204 77.320 77.000 76.680 72.575 65.927 53.219 46.905 21.651
NTs
N
F
8fC
PPM

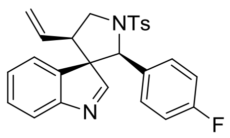

8fC

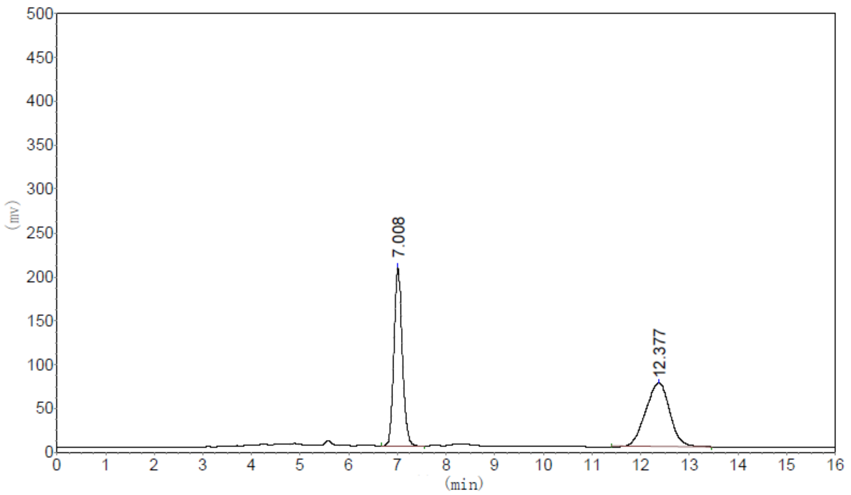

Peak No.	R. Time	Peak Height	Peak Area	Percent
1	7.008	203261.578	2436476.000	49.8675
2	12.377	71888.547	2449419.000	50.1325
Total		275150.125	4885895.000	100.0000

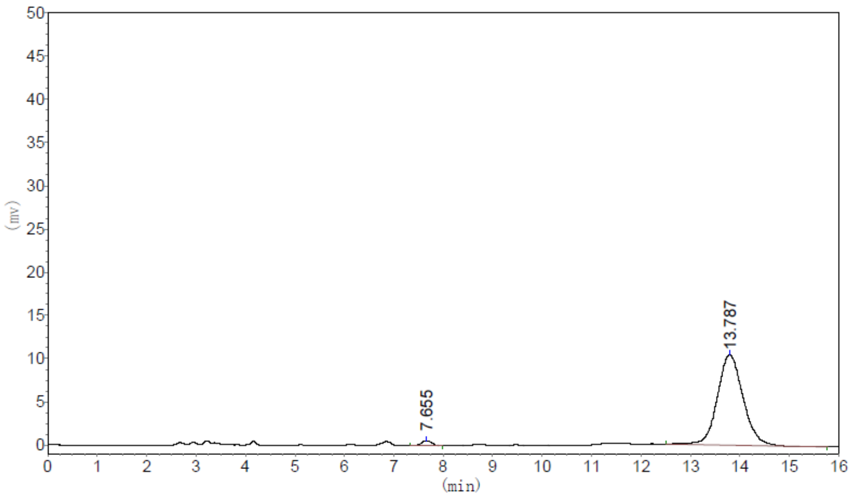

Peak No.	R. Time	Peak Height	Peak Area	Percent
1	7.655	601.563	8193.649	2.1281
2	13.787	10423.023	376819.188	97.8718
Total		11024.586	385012.837	100.0000

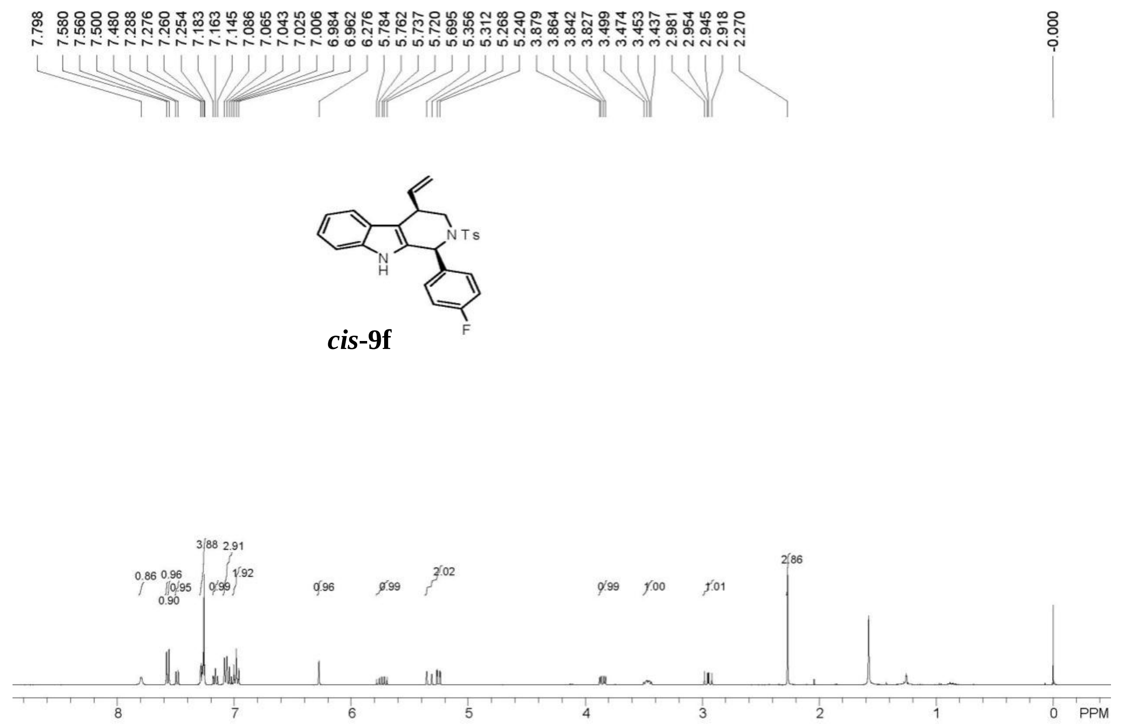
7.798 7.580 7.560 7.500 7.480 7.288 7.276 7.260 7.254 7.183 7.163 7.145 7.086 7.065 7.043 7.025 7.006 6.984 6.962 6.276 5.784 5.762 5.737 5.720 5.695 5.356 5.312 5.268 5.240 3.879 3.864 3.842 3.827 3.499 3.474 3.453 3.437 2.981 2.954 2.945 2.918 2.270
-0.000
NTs
N
H
F
cis-9f
0.86 0.96 0.95 0.90 3.88 2.91 1.92 0.99 0.96 0.99 2.02 0.99 1.00 1.01 2.86
8 7 6 5 4 3 2 1 0 PPM

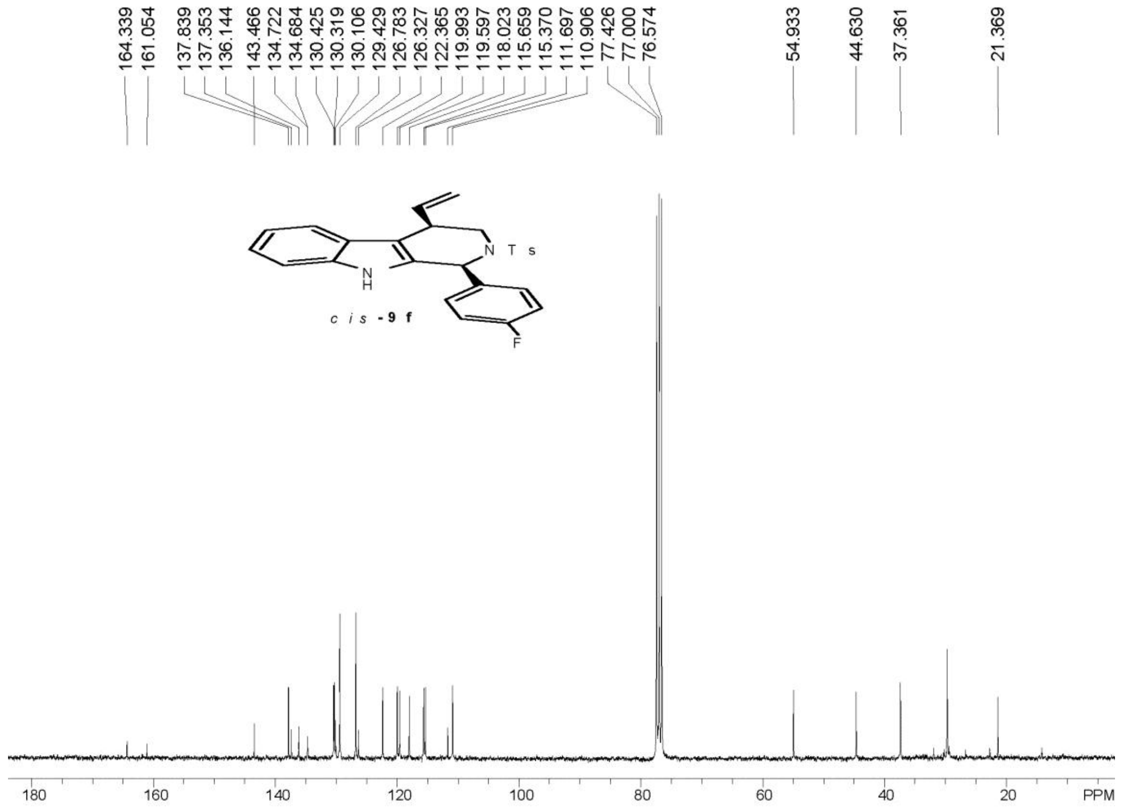
164.339 161.054 137.839 137.353 136.144 143.466 134.722 134.684 130.425 130.319 130.106 129.429 126.783 126.327 122.365 119.993 119.597 118.023 115.659 115.370 111.697 110.906 77.426 77.000 76.574
54.933
44.630
37.361
21.369
N T s
N
H
c i s - 9 f
F
180 160 140 120 100 80 60 40 20 PPM

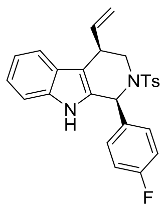

***cis*-9f**

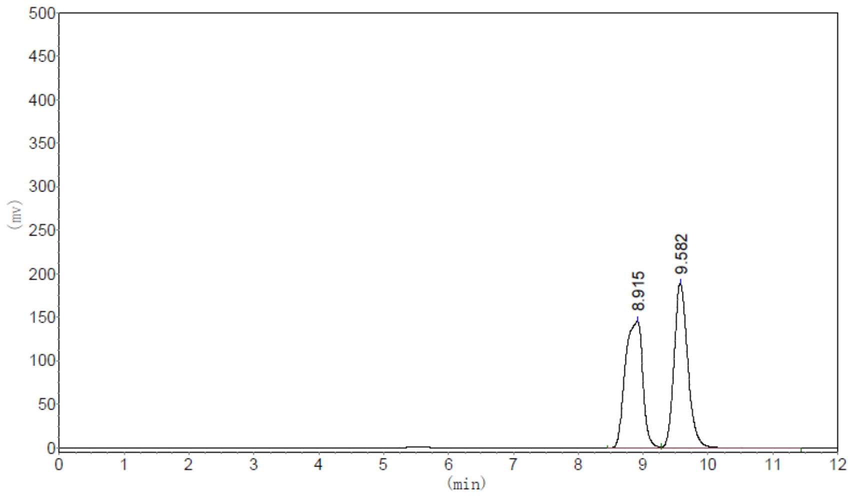

Peak No.	R. Time	Peak Height	Peak Area	Percent
1	8. 915	145273. 188	2725751. 500	49. 5844
2	9. 582	189216. 156	2771449. 000	50. 4156
Total		334489. 344	5497200. 500	100. 0000

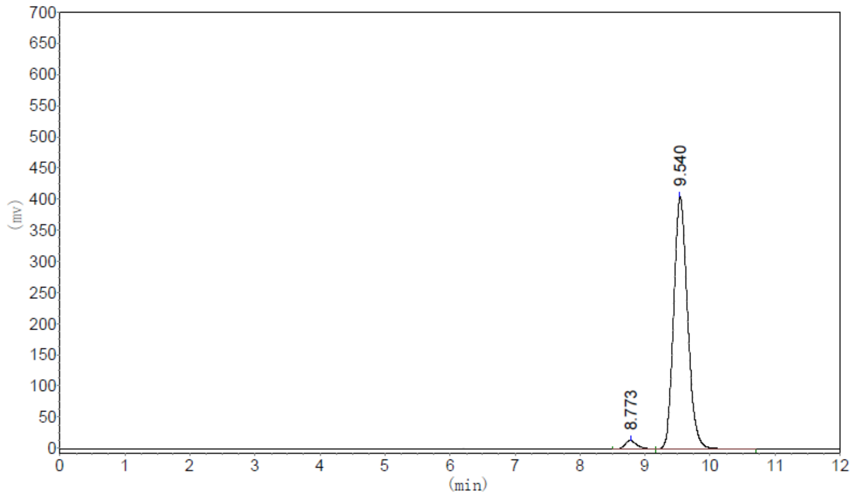

Peak No.	R. Time	Peak Height	Peak Area	Percent
1	8. 773	13566. 760	173903. 516	2. 8169
2	9. 540	404230. 594	5999770. 500	97. 1831
Total		417797. 354	6173674. 016	100. 0000

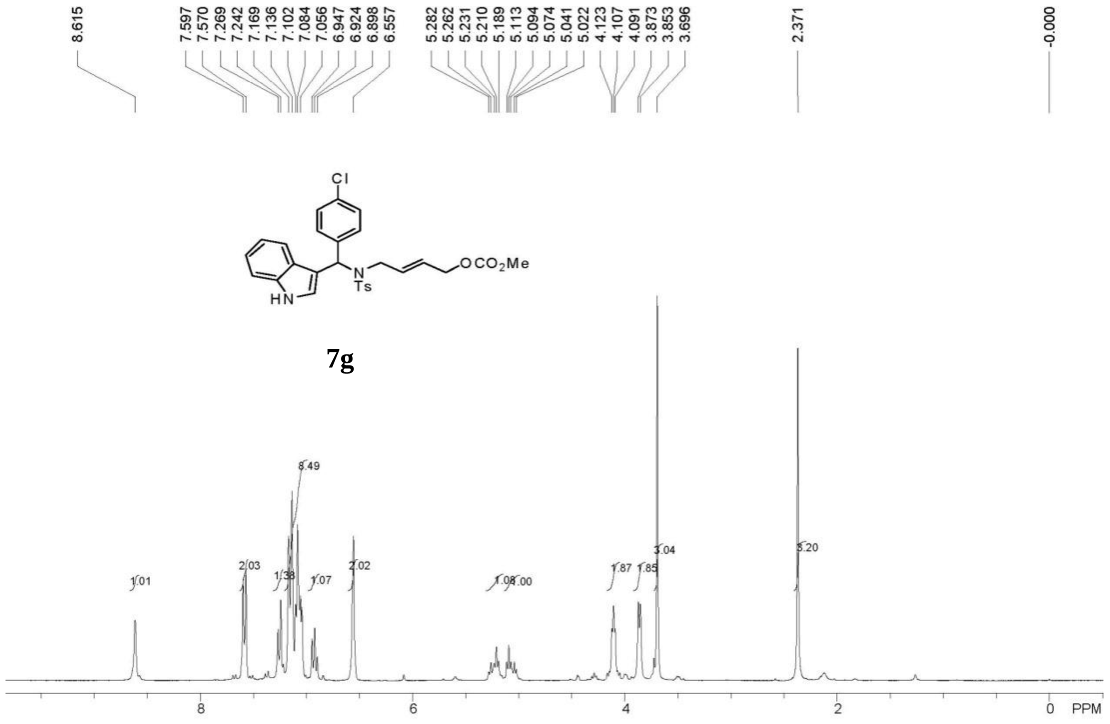
8.615
7.597 7.570 7.269 7.242 7.169 7.136 7.102 7.084 7.056 6.947 6.924 6.898 6.557
5.282 5.262 5.231 5.210 5.189 5.113 5.094 5.074 5.041 5.022 4.123 4.107 4.091 3.873 3.853 3.696
2.371
-0.000
Cl
N
Ts
HN
OCO2Me
1.01 2.03 1.33 8.49 1.07 2.02 1.08 1.00 1.87 1.85 3.04 3.20
8 6 4 2 0 PPM

7g

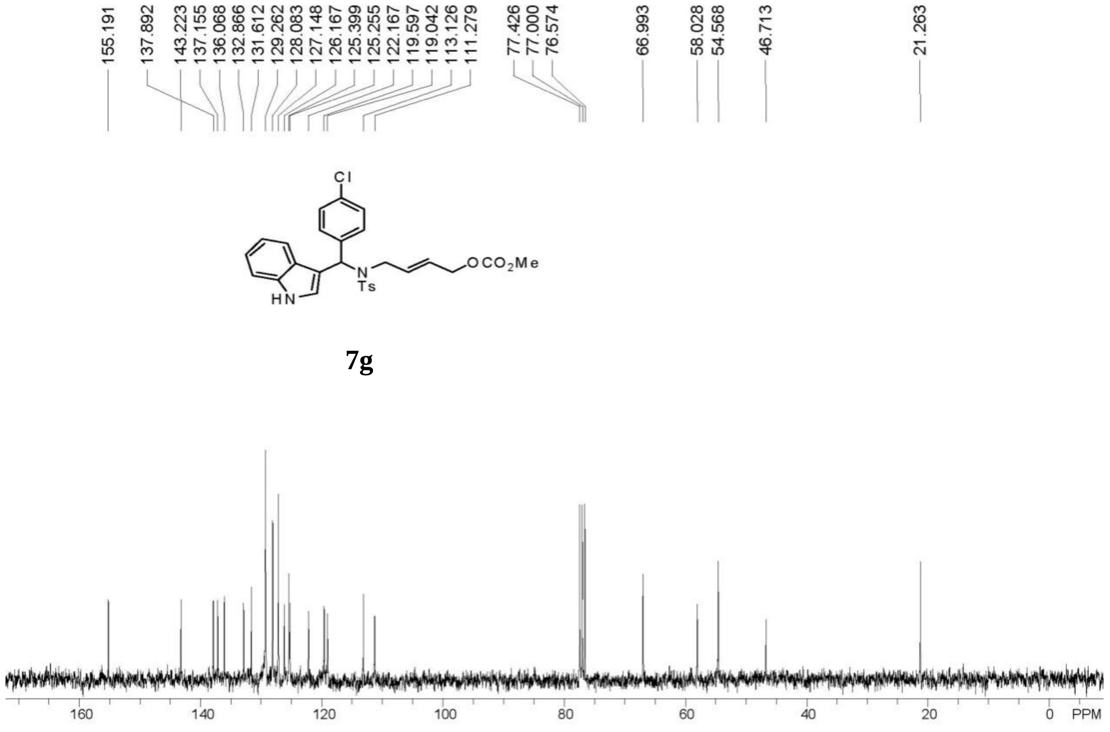
155.191
137.892 143.223 137.155 136.068 132.866 131.612 129.262 128.083 127.148 126.167 125.399 125.255 122.167 119.597 119.042 113.126 111.279
77.426 77.000 76.574
66.993
58.028 54.568
46.713
21.263
Cl
N
Ts
HN
OCO2Me
160 140 120 100 80 60 40 20 0 PPM

7g

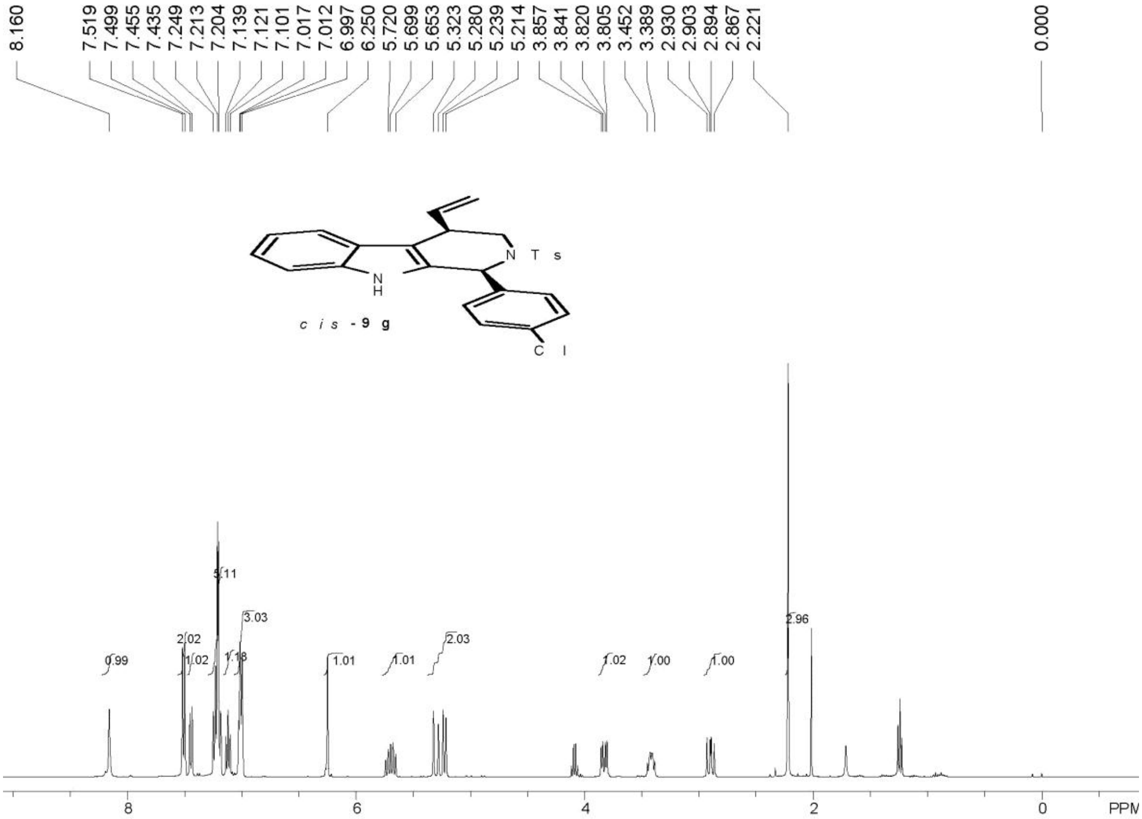
8.160
7.519
7.499
7.455
7.435
7.249
7.213
7.204
7.139
7.121
7.101
7.017
7.012
6.997
6.250
5.720
5.699
5.653
5.323
5.280
5.239
5.214
3.857
3.841
3.820
3.805
3.452
3.389
2.930
2.903
2.894
2.867
2.221
0.000
NTs
N
H
cis-9g
Cl
0.99
2.02
1.02
5.11
1.18
3.03
1.01
1.01
2.03
1.02
1.00
1.00
2.96
8
6
4
2
0
PPM

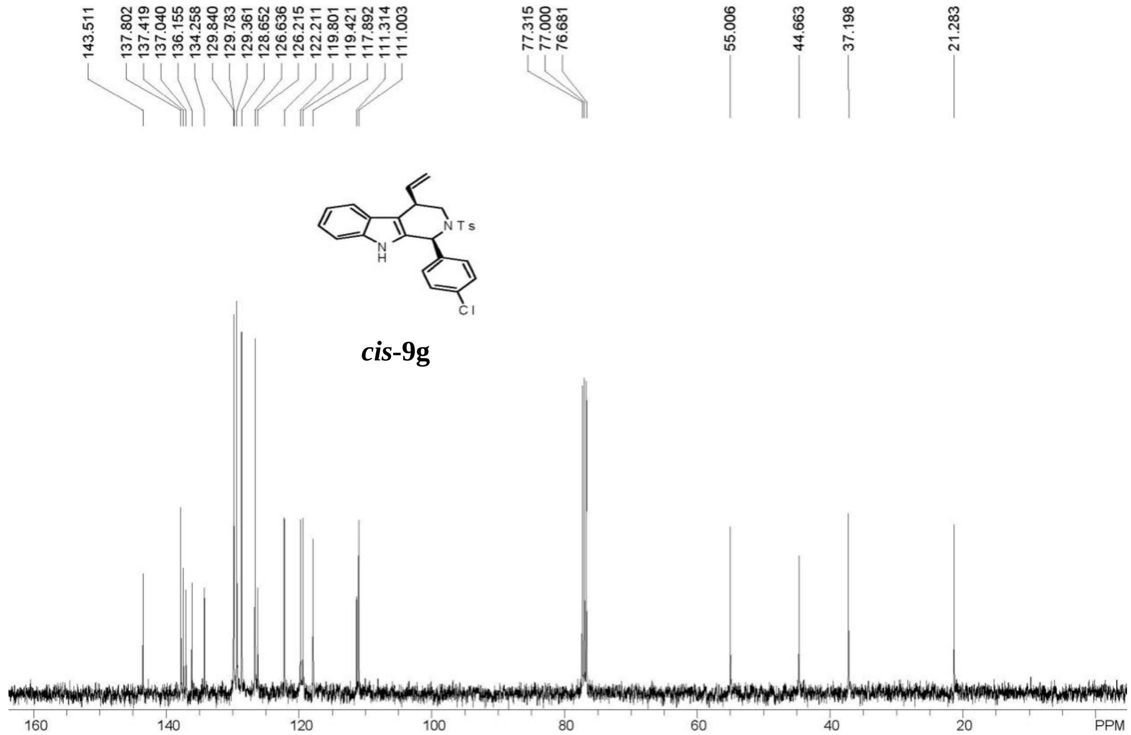
143.511
137.802
137.419
137.040
136.155
134.258
129.840
129.783
129.361
128.652
126.636
126.215
122.211
119.801
119.421
117.892
111.314
111.003
77.315
77.000
76.681
55.006
44.663
37.198
21.283
NTs
N
H
Cl
cis-9g
160
140
120
100
80
60
40
20
PPM

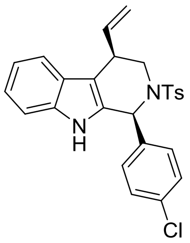

***cis*-9g**

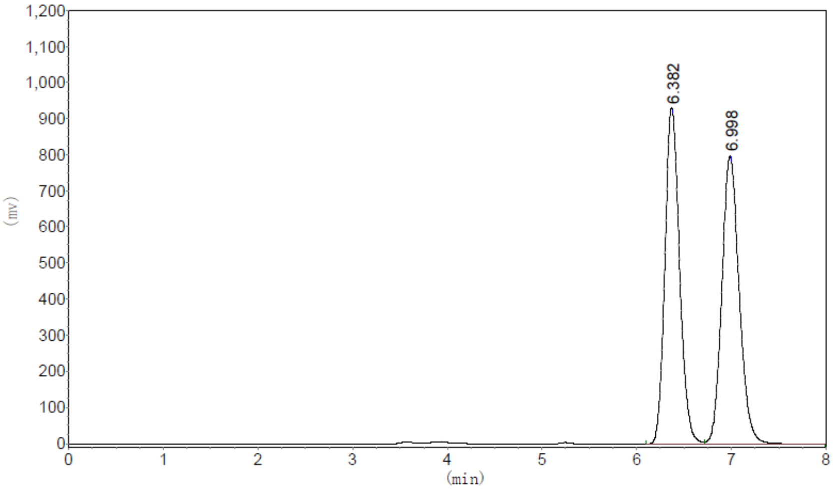

Peak No.	R. Time	Peak Height	Peak Area	Percent
1	6. 382	926508. 188	9992105. 000	49. 5386
2	6. 998	791864. 750	10178249. 000	50. 4614
Total		1718372. 938	20170354. 000	100. 0000

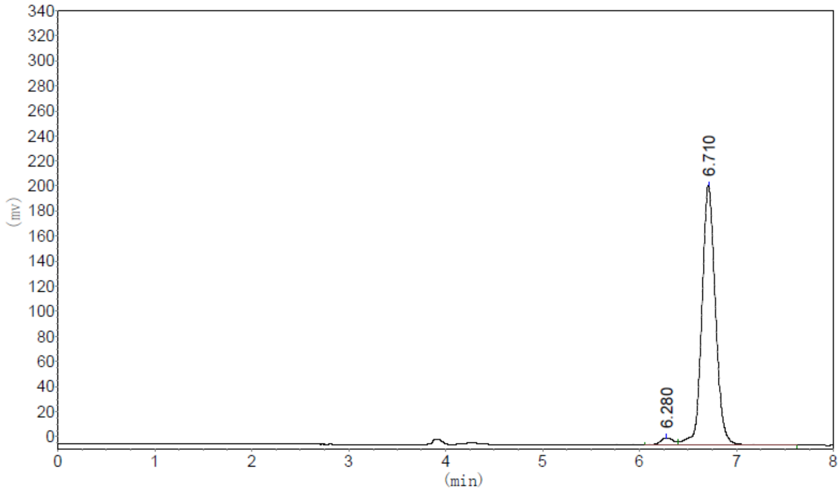

Peak No.	R. Time	Peak Height	Peak Area	Percent
1	6. 280	5426. 600	50634. 039	2. 4630
2	6. 710	206877. 516	2005138. 625	97. 5370
Total		212304. 116	2055772. 664	100. 0000

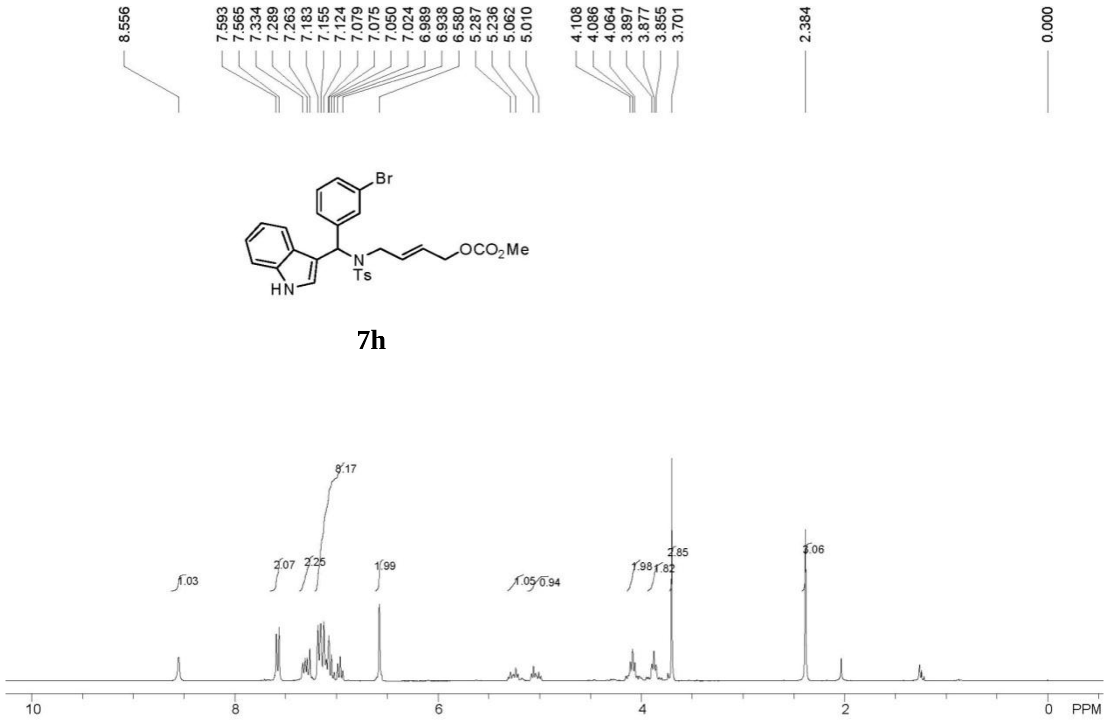
8.556
7.593 7.565 7.334 7.289 7.263 7.183 7.155 7.124 7.079 7.075 7.050 7.024 6.989 6.938 6.580 5.287 5.236 5.062 5.010
4.108 4.086 4.064 3.897 3.877 3.855 3.701
2.384
0.000
Br
N
Ts
OCO2Me
HN
1.03 2.07 2.25 8.17 1.99 1.05 0.94 1.98 1.82 2.85 3.06
10 8 6 4 2 0 PPM

7h

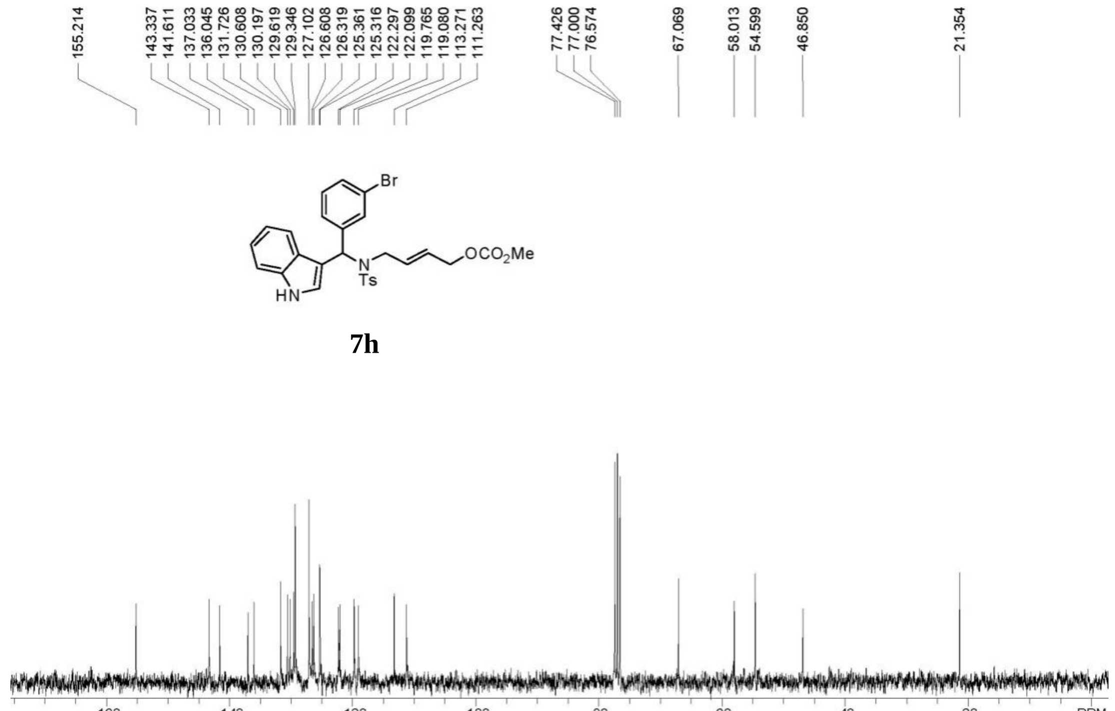
155.214
143.337 141.611 137.033 136.045 131.726 130.608 130.197 129.619 129.346 127.102 126.608 126.319 125.361 125.316 122.297 122.099 119.765 119.080 113.271 111.263
77.426 77.000 76.574
67.069
58.013
54.599
46.850
21.354
Br
N
Ts
OCO2Me
HN
160 140 120 100 80 60 40 20 PPM

7h

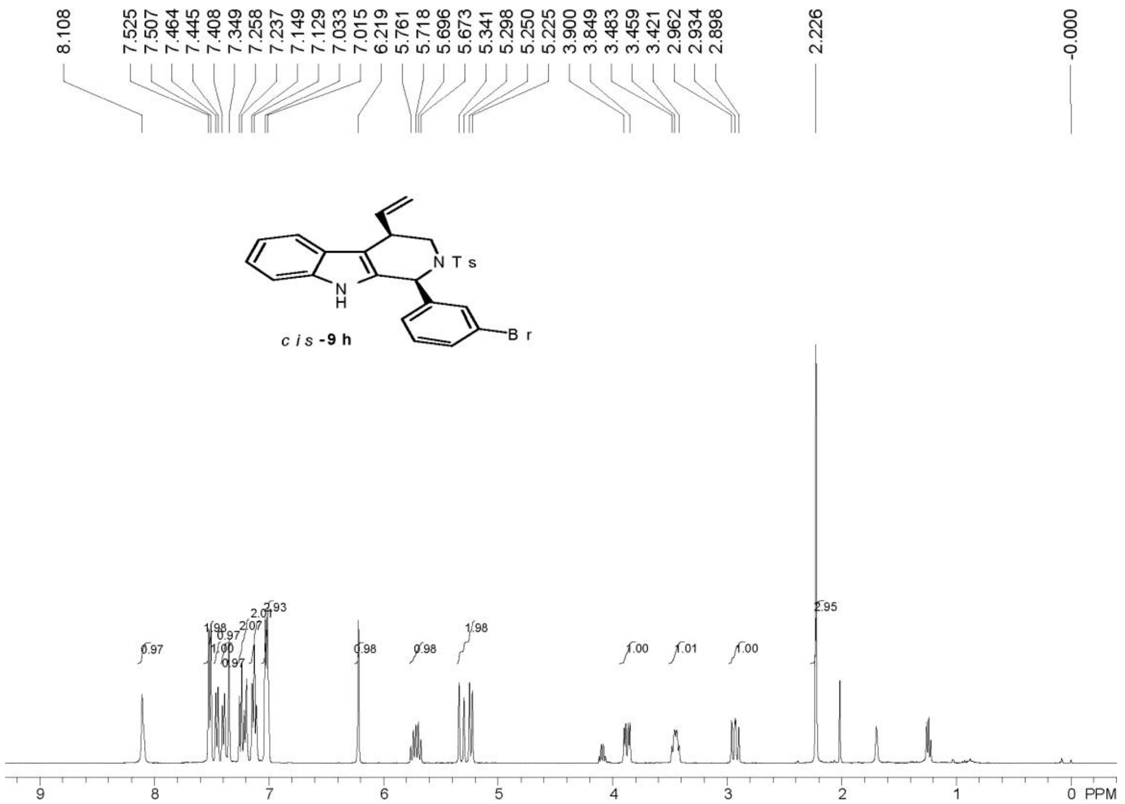

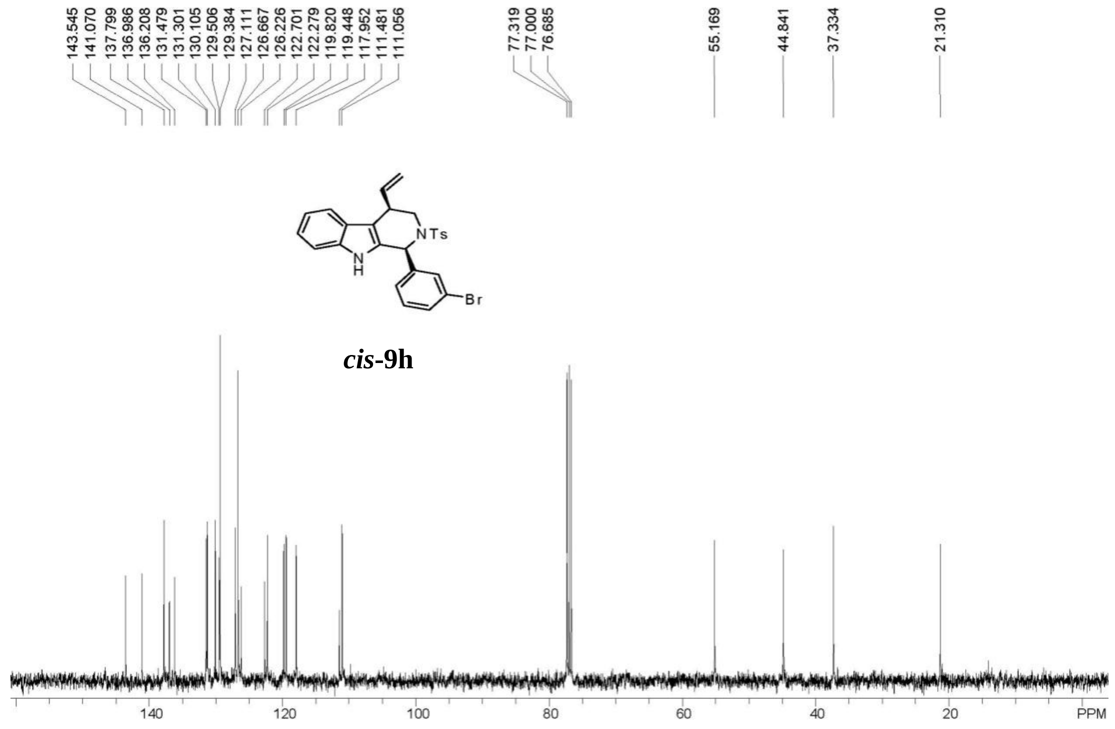

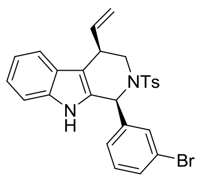

***cis*-9h**

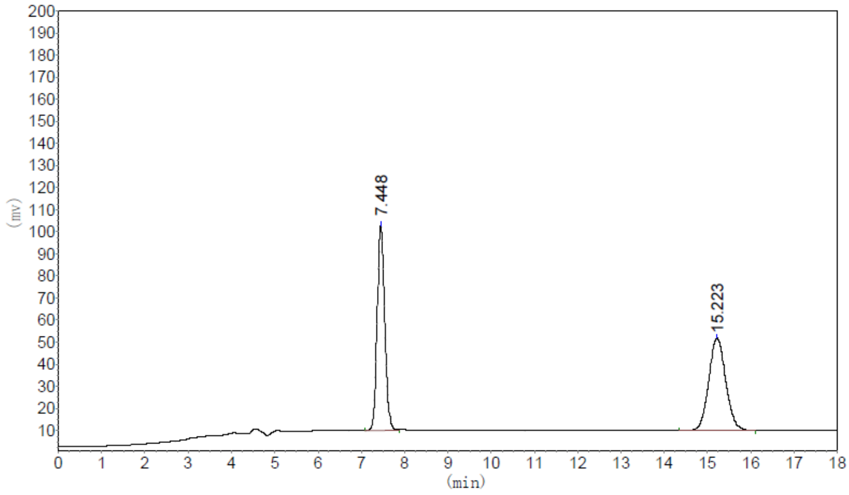

Peak No.	R. Time	Peak Height	Peak Area	Percent
1	7.448	92619.313	1121328.375	49.6144
2	15.223	41795.199	1138757.500	50.3856
Total		134414.512	2260085.875	100.0000

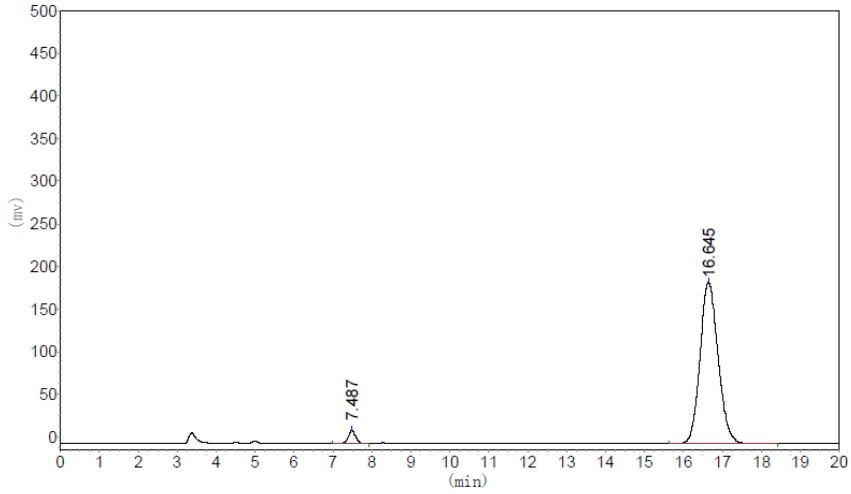

Peak No.	R. Time	Peak Height	Peak Area	Percent
1	7.487	15487.804	197709.406	3.1347
2	16.645	188836.359	6109501.000	96.8653
Total		204324.163	6307210.406	100.0000

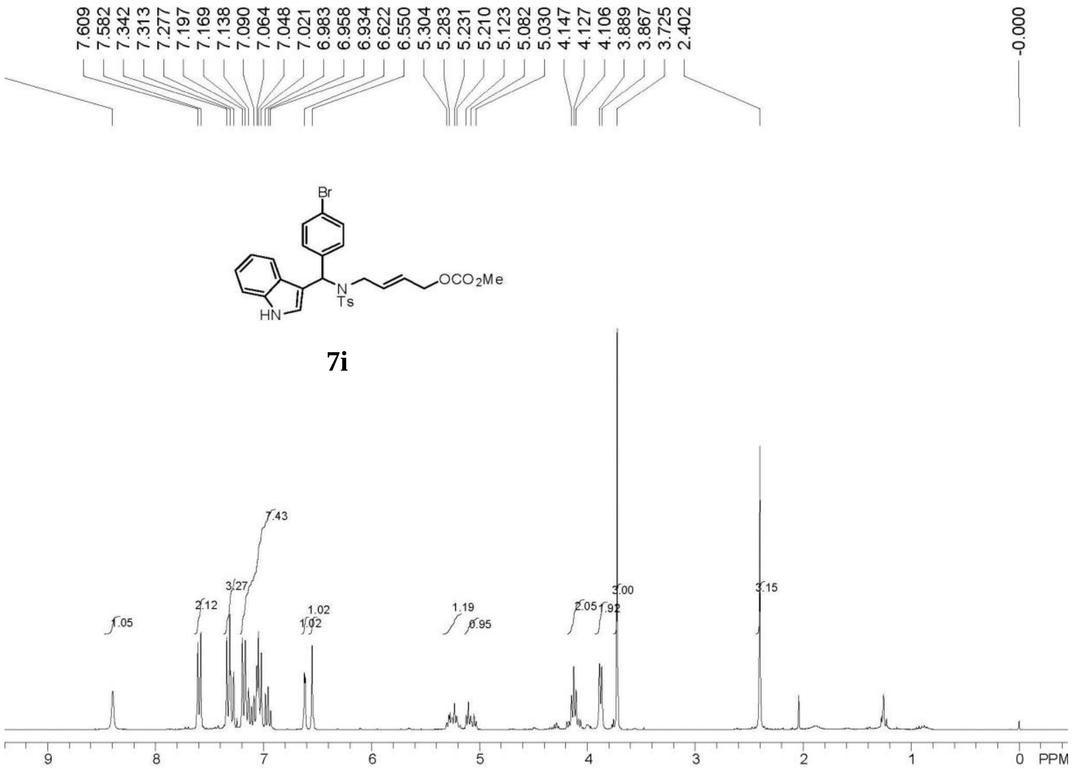
7.609 7.582 7.342 7.313 7.277 7.197 7.169 7.138 7.090 7.064 7.048 7.021 6.983 6.958 6.934 6.622 6.550 5.304 5.283 5.231 5.210 5.123 5.082 5.030 4.147 4.127 4.106 3.889 3.867 3.725 2.402
-0.000
Br
HN
N
Ts
OCO2Me
7i
1.05 2.12 3.27 7.43 1.02 1.02 1.19 0.95 2.05 1.92 3.00 3.15
9 8 7 6 5 4 3 2 1 0 PPM

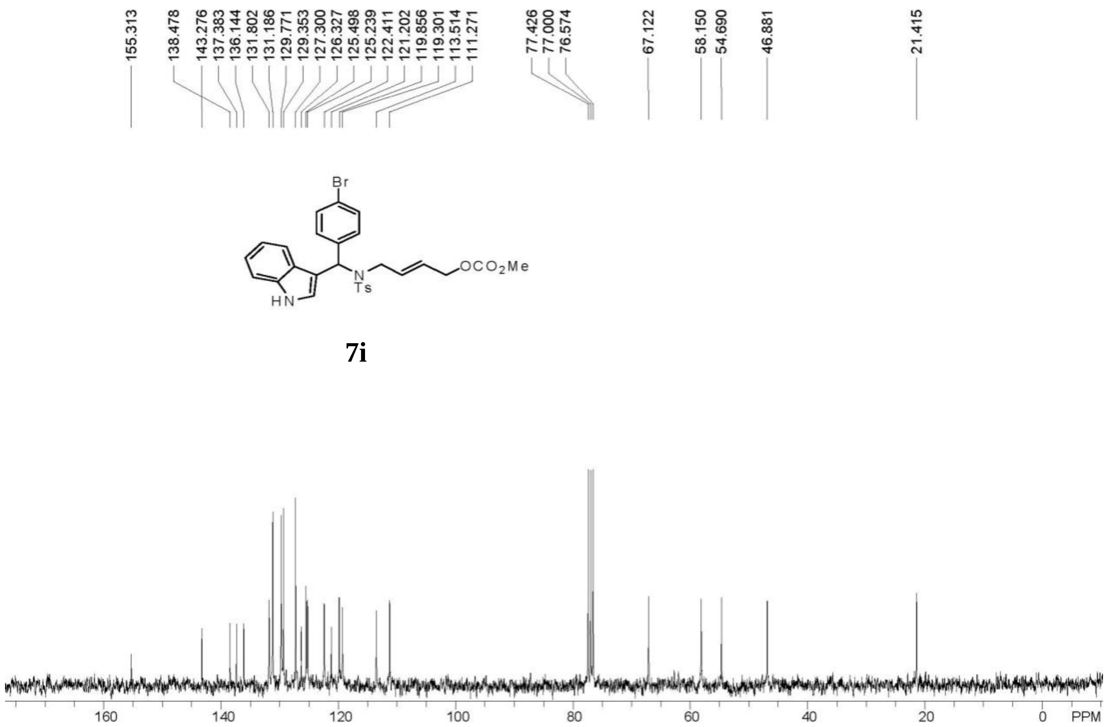
155.313
138.478 143.276 137.383 136.144 131.802 131.186 129.771 129.353 127.300 126.327 125.498 125.239 122.411 121.202 119.856 119.301 113.514 111.271
77.426 77.000 76.574
67.122
58.150 54.690
46.881
21.415
Br
HN
N
Ts
OCO2Me
7i
160 140 120 100 80 60 40 20 0 PPM

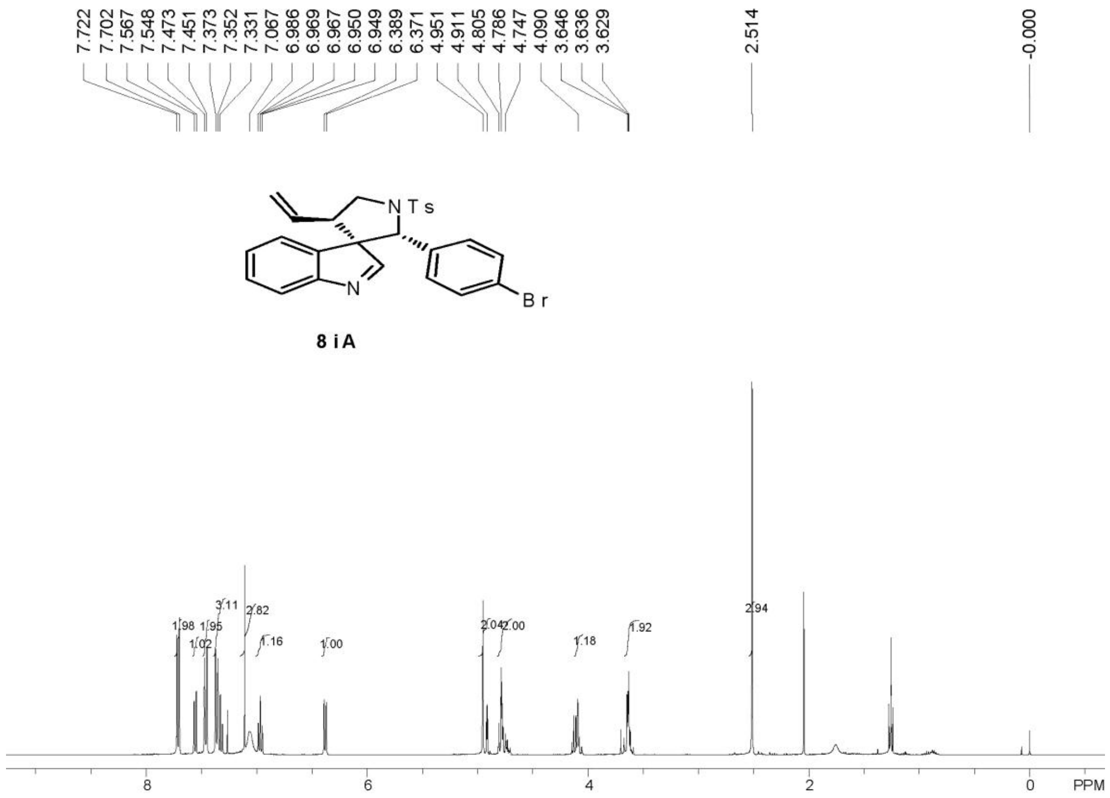
7.722
7.702
7.567
7.548
7.473
7.451
7.373
7.352
7.331
7.067
6.986
6.969
6.967
6.950
6.949
6.389
6.371
4.951
4.911
4.805
4.786
4.747
4.090
3.646
3.636
3.629
2.514
-0.000
NTs
N
Br
8 i A
1.98
1.02
1.95
3.11
2.82
1.16
1.00
2.04
2.00
1.18
1.92
2.94
8
6
4
2
0
PPM

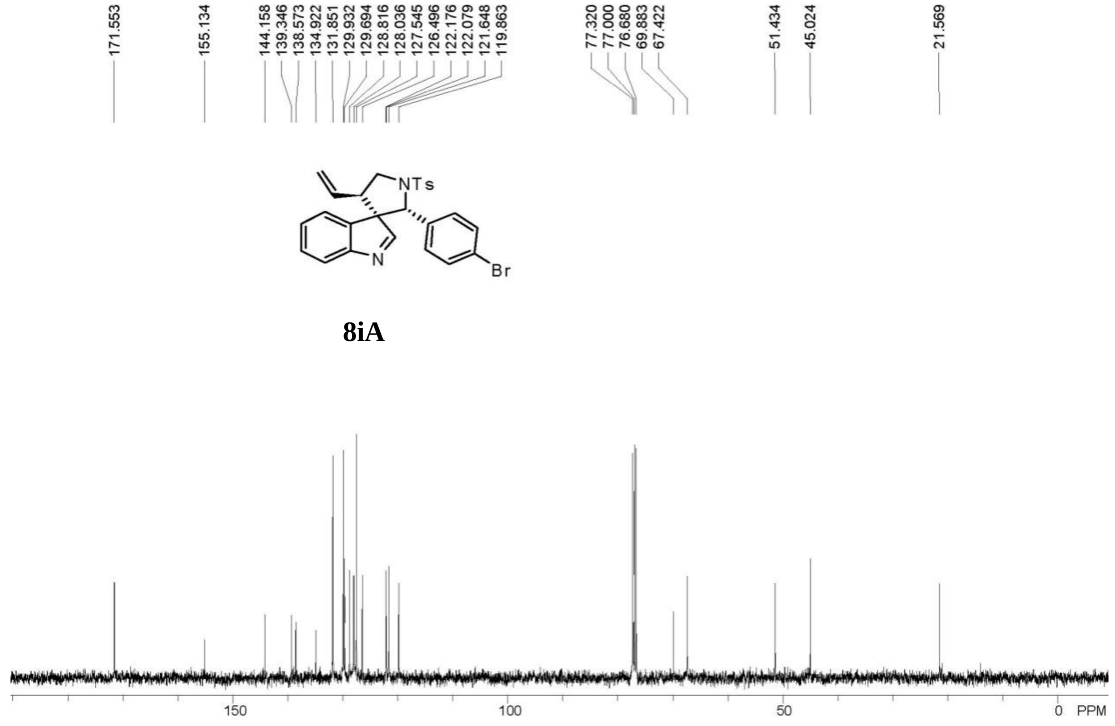
171.553
155.134
144.158
139.346
138.573
134.922
131.851
129.932
129.694
128.816
128.036
127.545
126.496
122.176
122.079
121.648
119.863
77.320
77.000
76.680
69.883
67.422
51.434
45.024
21.569
NTs
N
Br
8iA
150
100
50
0
PPM

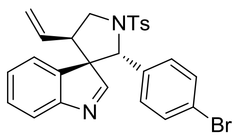

8iA

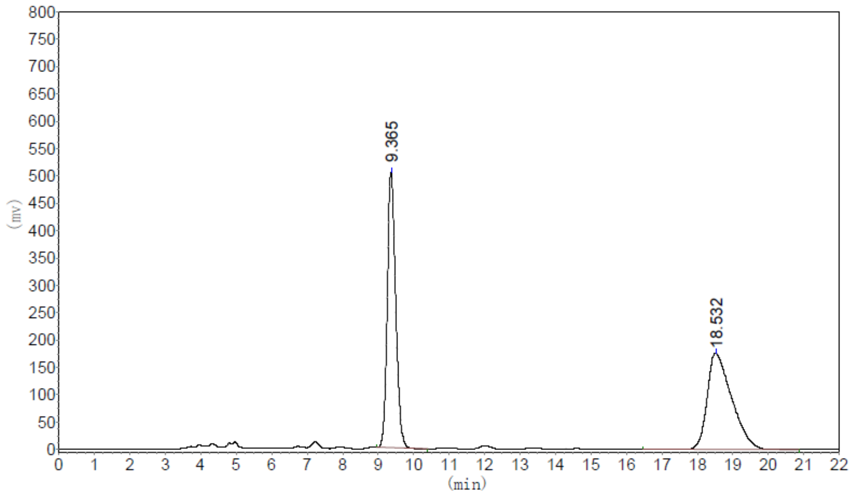

Peak No.	R. Time	Peak Height	Peak Area	Percent
1	9. 365	505561. 344	8505768. 000	50. 1171
2	18. 532	176429. 781	8466012. 000	49. 8829
Total		681991. 125	16971780. 000	100. 0000

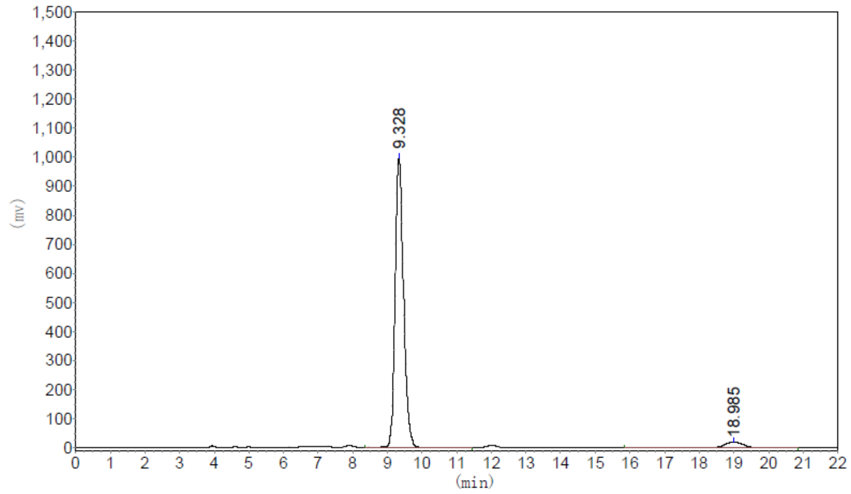

Peak No.	R. Time	Peak Height	Peak Area	Percent
1	9. 328	996017. 063	16882690. 000	95. 5679
2	18. 985	19754. 871	782962. 313	4. 4321
Total		1015771. 934	17665652. 313	100. 0000

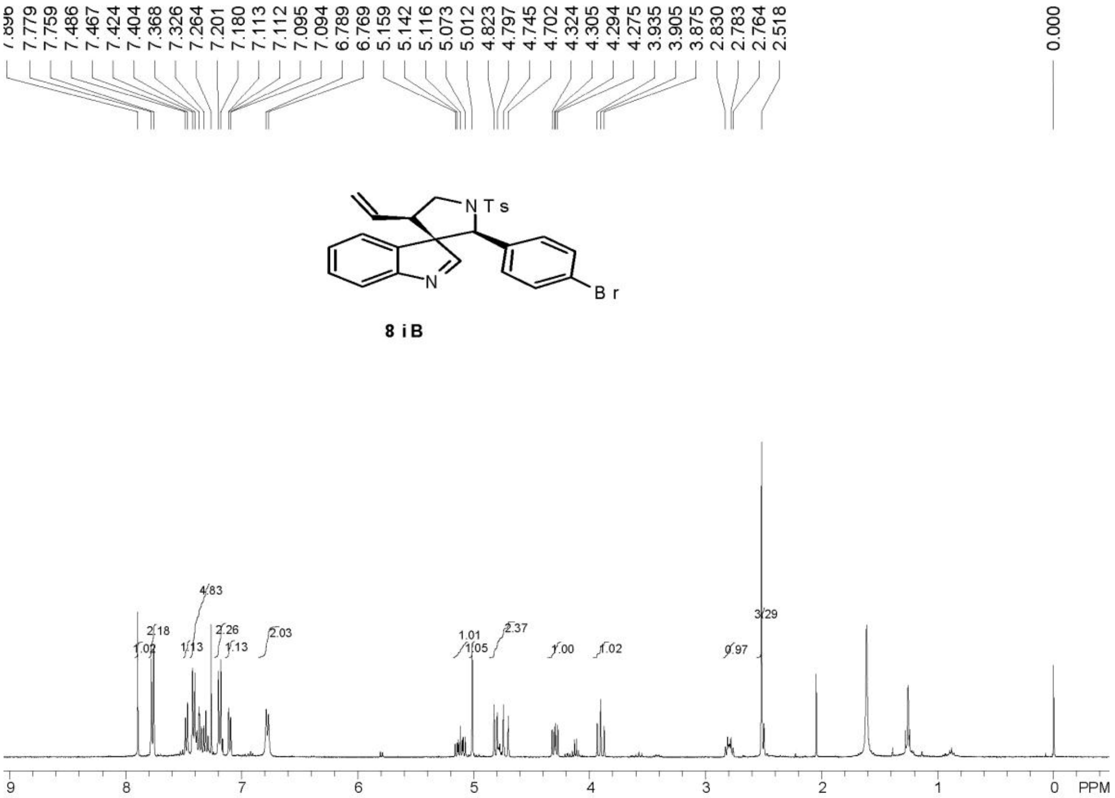
7.779 7.759 7.486 7.467 7.424 7.404 7.368 7.326 7.264 7.201 7.180 7.113 7.112 7.095 7.094 6.789 6.769 5.159 5.142 5.116 5.073 5.012 4.823 4.797 4.745 4.702 4.324 4.305 4.294 4.275 3.935 3.905 3.875 2.830 2.783 2.764 2.518
0.000
N Ts
N
B r
1.02 2.18 1.13 4.83 2.26 1.13 2.03 1.01 1.05 2.37 1.00 1.02 0.97 3.29
9 8 7 6 5 4 3 2 1 0 PPM

8 iB

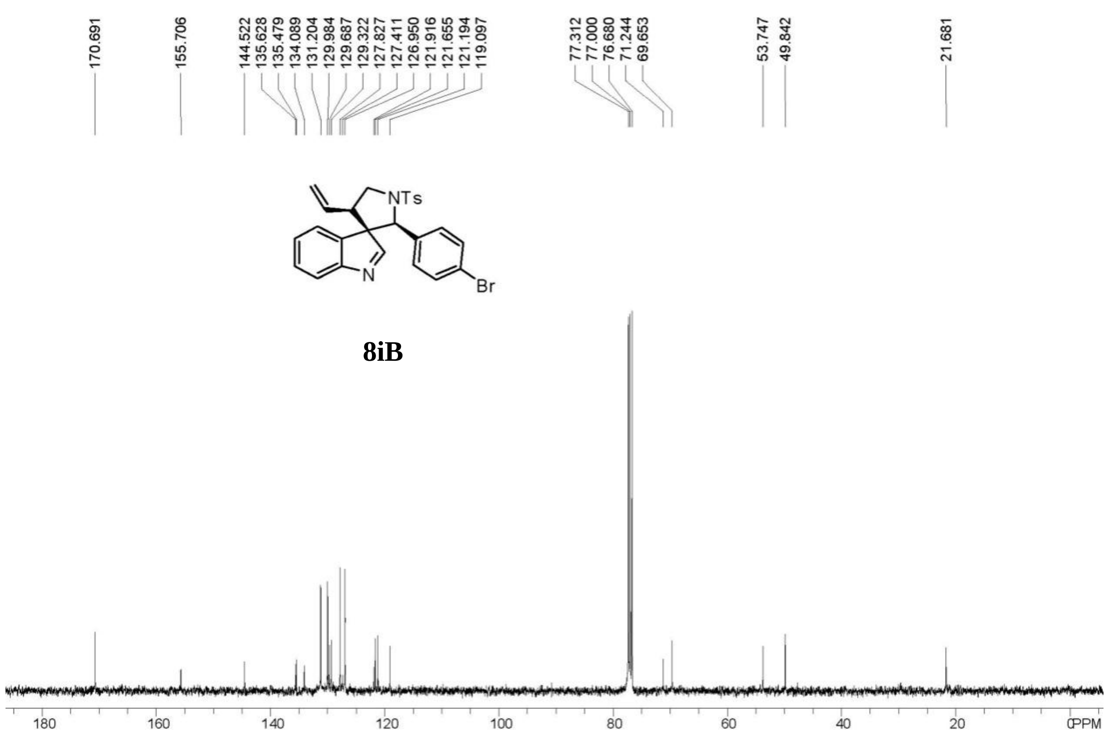
170.691
155.706
144.522 135.628 135.479 134.089 131.204 129.984 129.687 129.322 127.827 127.411 126.950 121.916 121.655 121.194 119.097
77.312 77.000 76.680 71.244 69.653
53.747 49.842
21.681
NTs
N
Br
180 160 140 120 100 80 60 40 20 0PPM

8iB

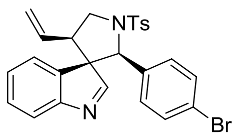

8iB

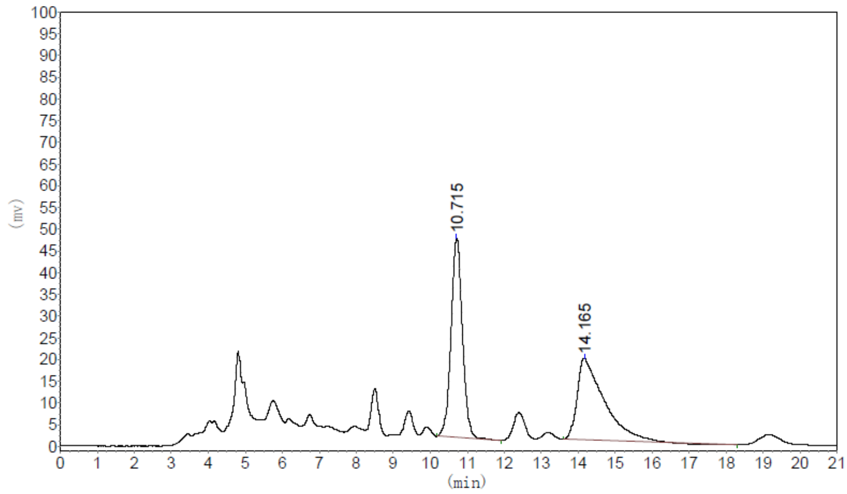

Peak No.	R. Time	Peak Height	Peak Area	Percent
1	10.715	47749.008	1151721.750	49.7414
2	14.165	20014.219	1163696.500	50.2586
Total		67763.227	2315418.250	100.0000

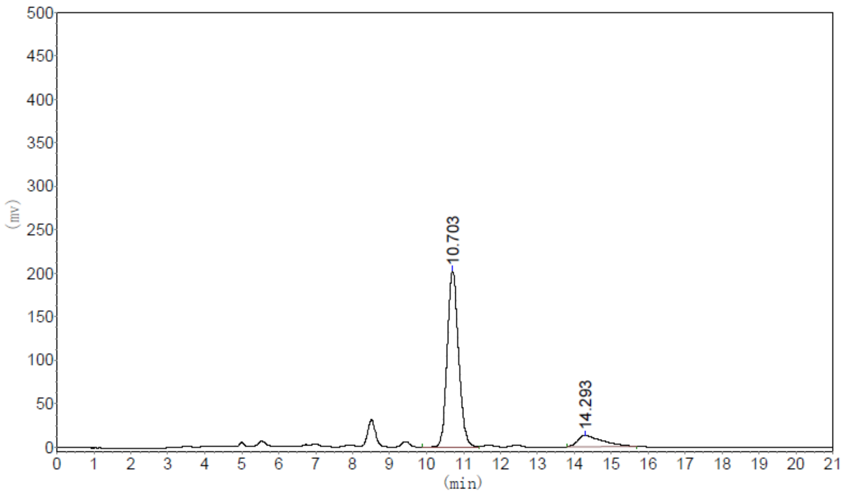

Peak No.	R. Time	Peak Height	Peak Area	Percent
1	10.703	203100.891	4327168.500	87.7635
2	14.293	13393.043	603321.375	12.2365
Total		216493.934	4930489.875	100.0000

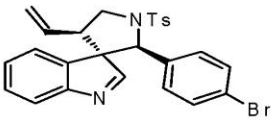

NTs
N
Br

8iC

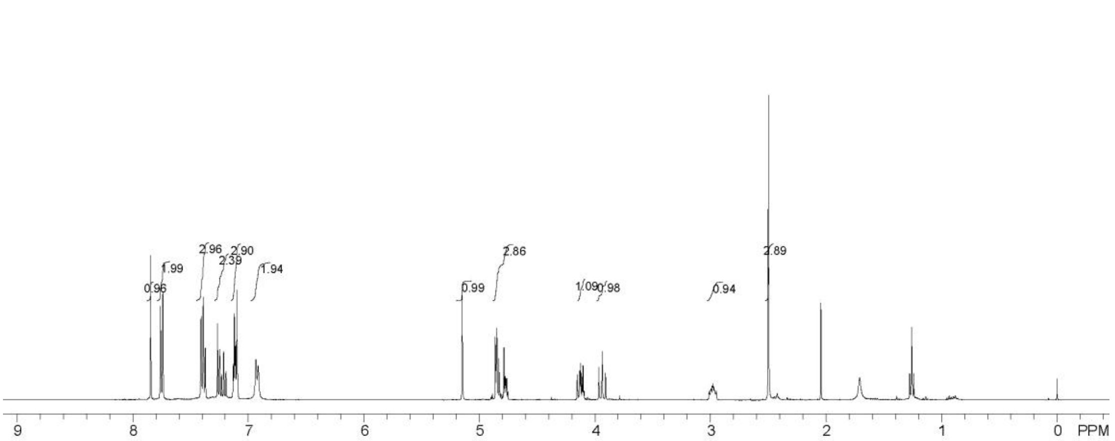

7.846
7.761
7.740
7.409
7.389
7.372
7.266
7.211
7.121
7.099
6.934
6.915
4.862
5.149
4.858
4.831
4.788
4.767
4.756
4.153
4.133
4.103
3.966
3.937
3.908
2.984
2.978
2.499
0.000
0.96
1.99
2.96
2.39
2.90
1.94
0.99
2.86
1.09
0.98
0.94
2.89
9
8
7
6
5
4
3
2
1
0
PPM

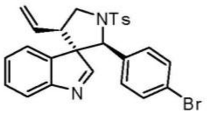

NTs
N
Br

8iC

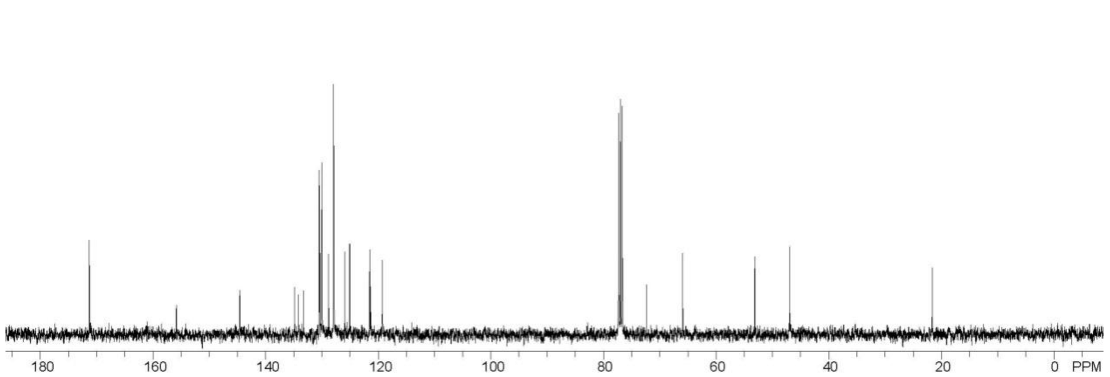

171.293
155.840
144.582
134.877
134.148
133.271
130.490
130.400
130.029
128.816
127.932
125.931
125.069
121.514
121.365
119.276
77.320
77.000
76.680
72.345
65.950
53.144
46.979
21.644
180
160
140
120
100
80
60
40
20
0
PPM

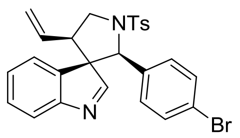

8iC

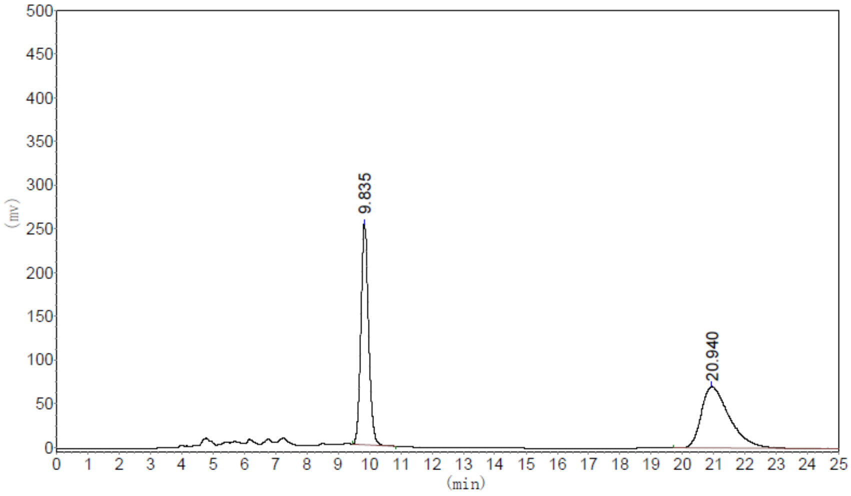

Peak No.	R. Time	Peak Height	Peak Area	Percent
1	9. 835	252805. 563	4335868. 000	49. 6175
2	20. 940	70418. 641	4402717. 500	50. 3825
Total		323224. 203	8738585. 500	100. 0000

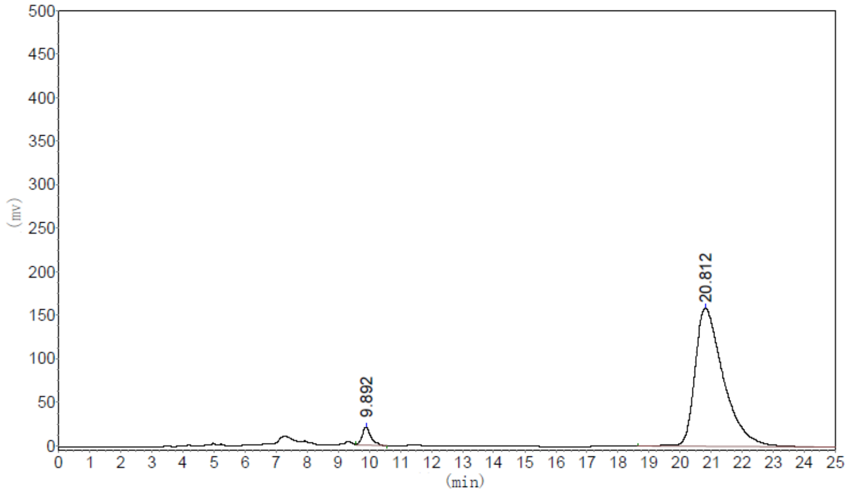

Peak No.	R. Time	Peak Height	Peak Area	Percent
1	9. 892	20879. 695	410943. 250	3. 9022
2	20. 812	158789. 000	10120081. 000	96. 0978
Total		179668. 695	10531024. 250	100. 0000

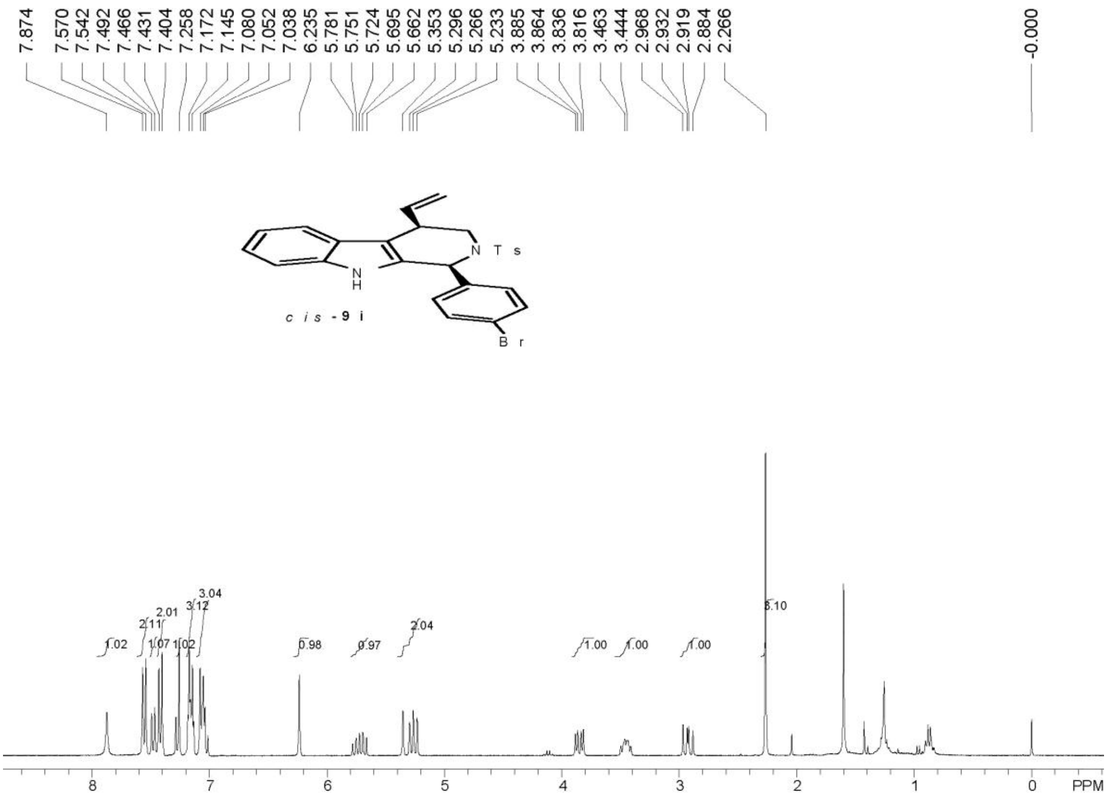
7.874 7.570 7.542 7.492 7.466 7.431 7.404 7.258 7.172 7.145 7.080 7.052 7.038 6.235 5.781 5.751 5.724 5.695 5.662 5.353 5.296 5.266 5.233 3.885 3.864 3.836 3.816 3.463 3.444 2.968 2.932 2.919 2.884 2.266
-0.000
NTs
N
H
cis-9i
Br
1.02 2.11 2.01 1.07 1.02 3.12 3.04 0.98 0.97 2.04 1.00 1.00 1.00 3.10
8 7 6 5 4 3 2 1 0 PPM

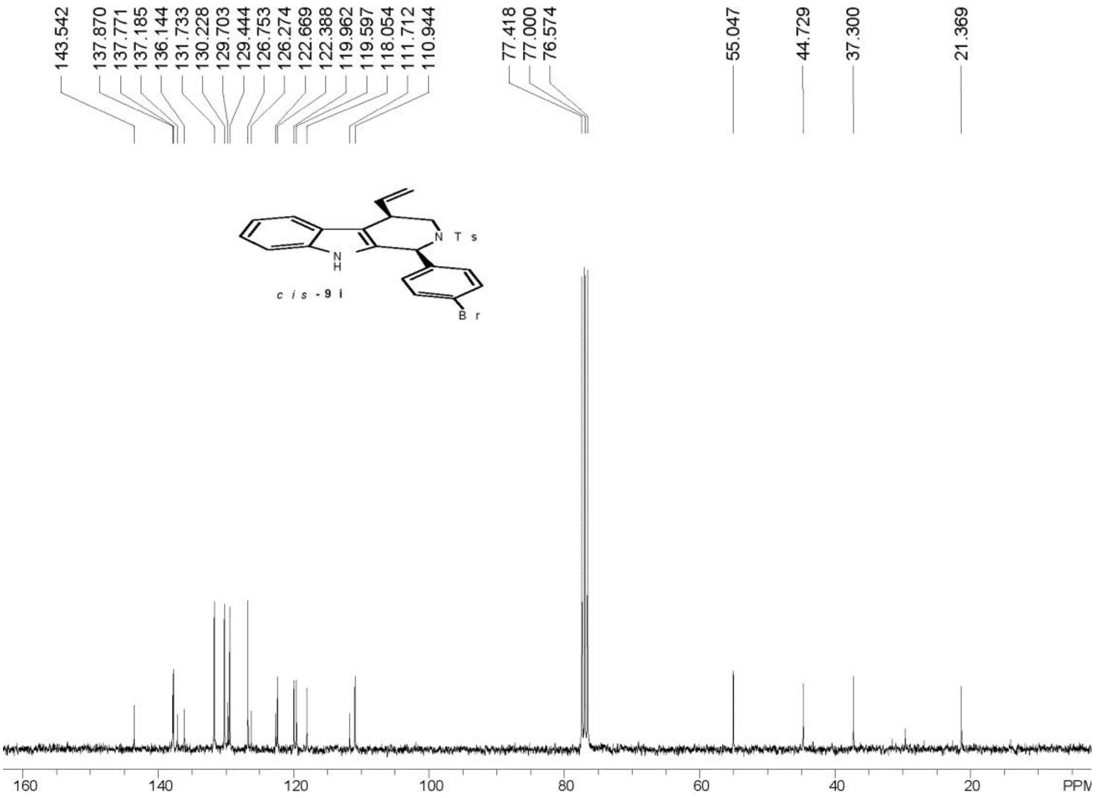
143.542 137.870 137.771 137.185 136.144 131.733 130.228 129.703 129.444 126.753 126.274 122.669 122.388 119.962 119.597 118.054 111.712 110.944
77.418 77.000 76.574
55.047
44.729
37.300
21.369
NTs
N
H
cis-9i
Br
160 140 120 100 80 60 40 20 PPM

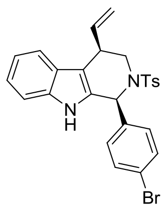

***cis*-9i**

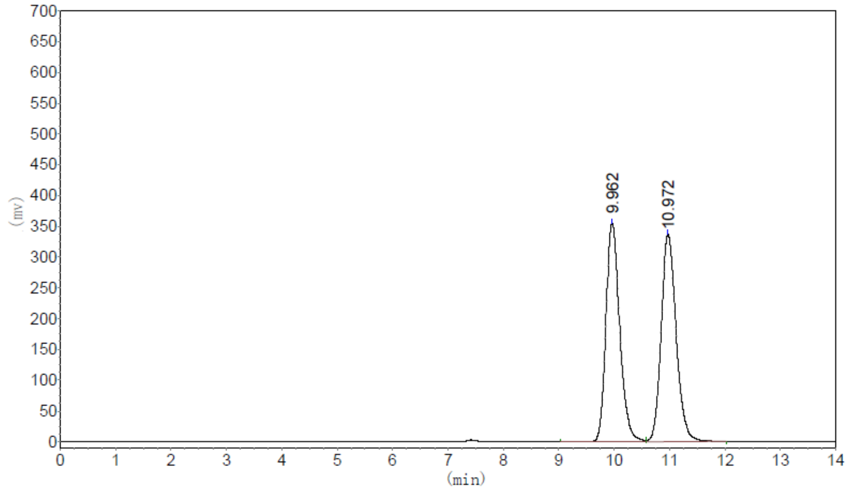

Peak No.	R. Time	Peak Height	Peak Area	Percent
1	9.962	355305.406	6126222.000	49.2754
2	10.972	336783.188	6306403.500	50.7246
Total		692088.594	12432625.500	100.0000

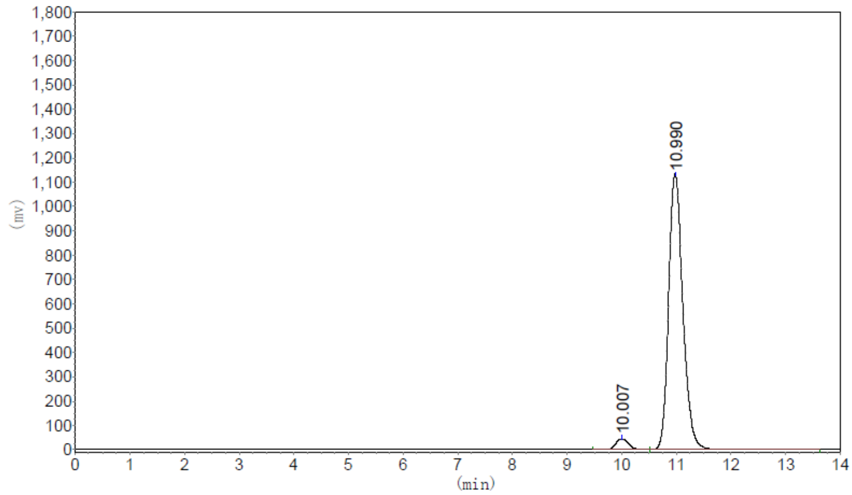

Peak No.	R. Time	Peak Height	Peak Area	Percent
1	10.007	45724.520	763733.250	3.5805
2	10.990	1131474.000	20566356.000	96.4195
Total		1177198.520	21330089.250	100.0000

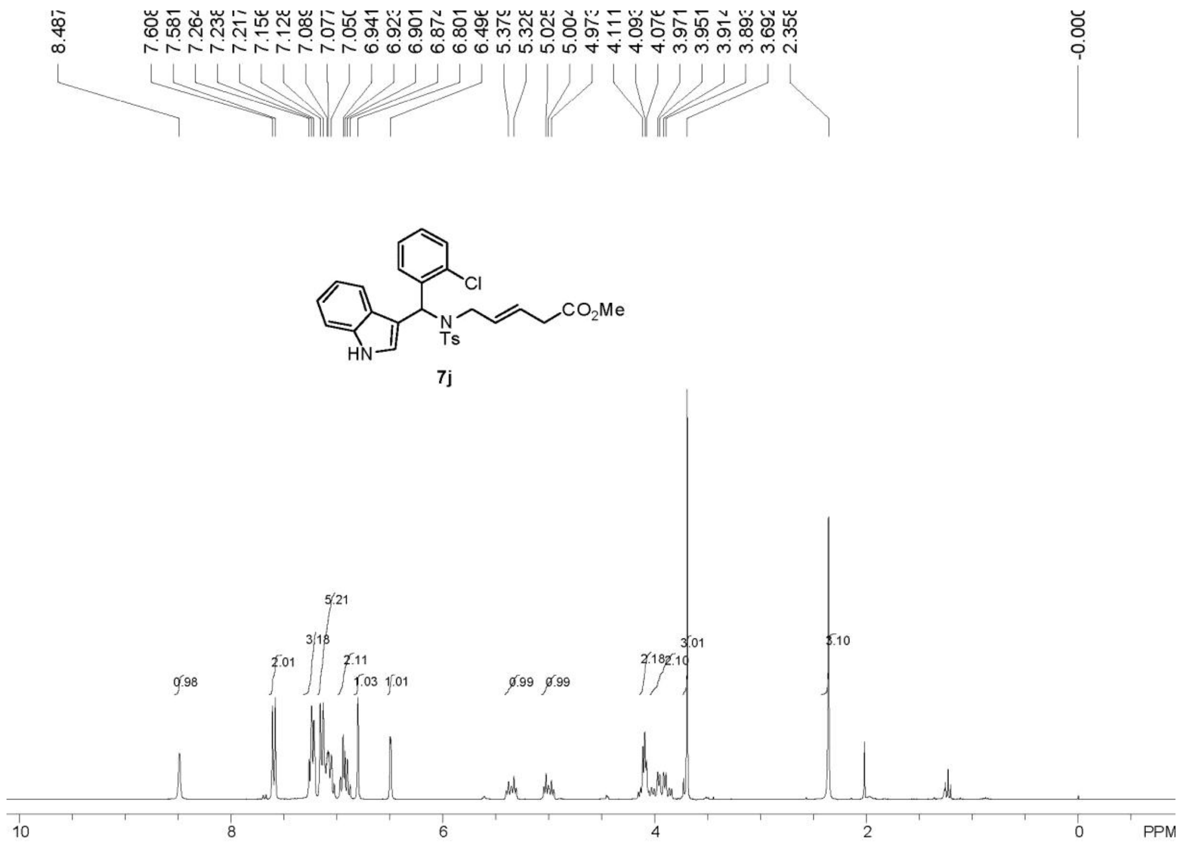

7j

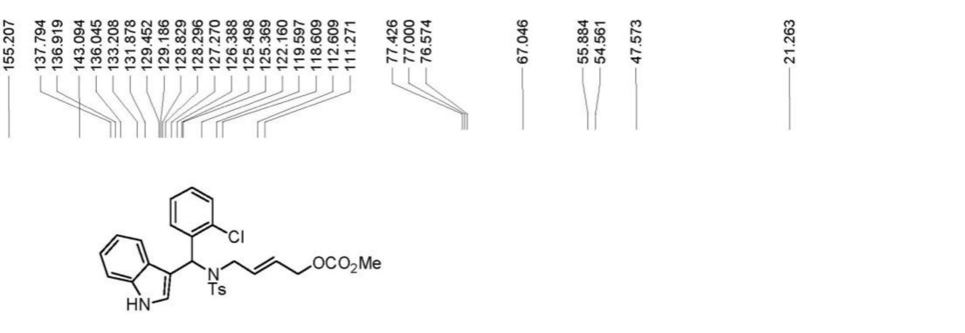

7j

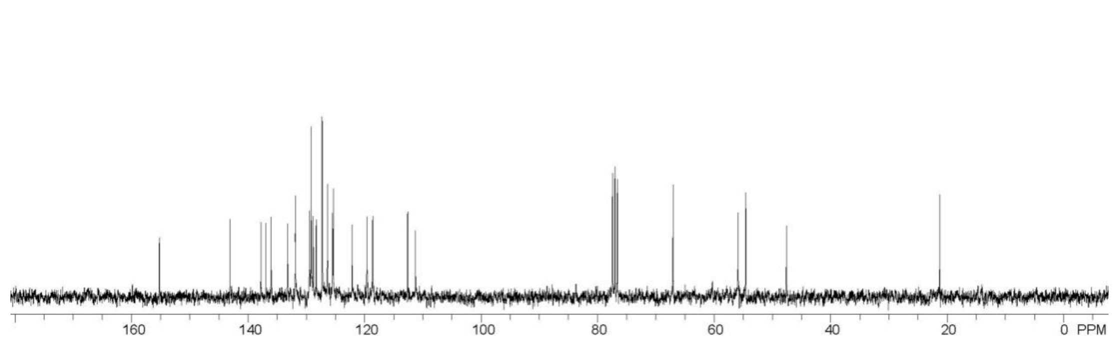

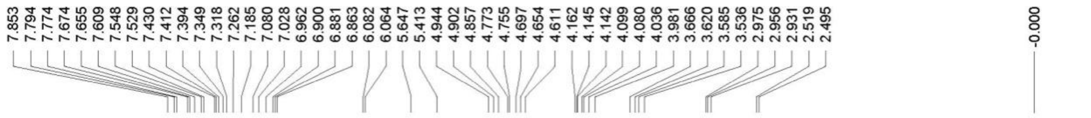

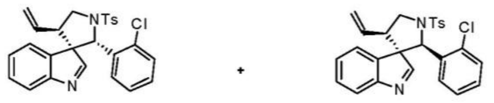

8jA+8jC

1:0.67

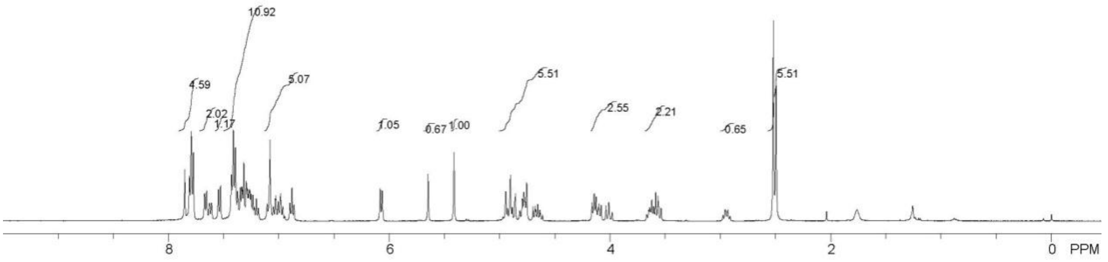

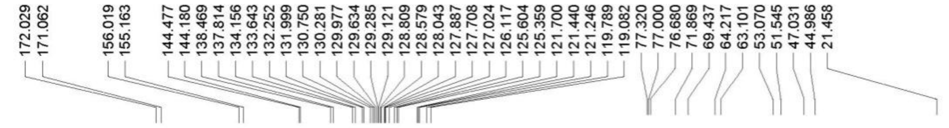

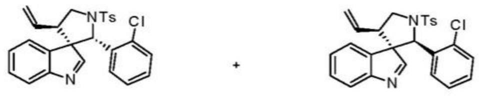

8jA+8jC

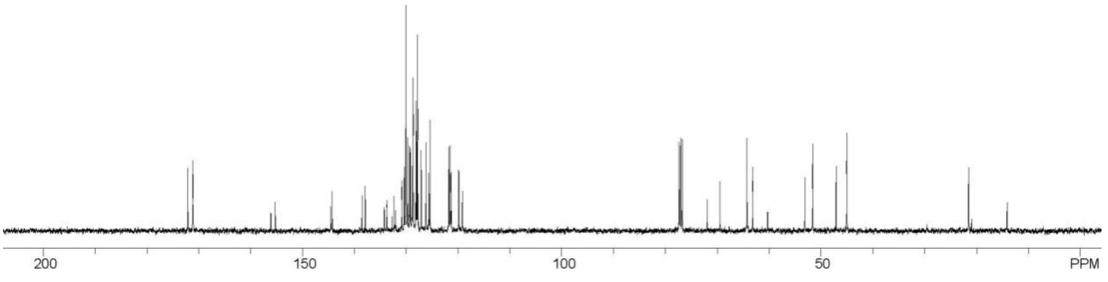

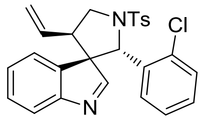

8jA

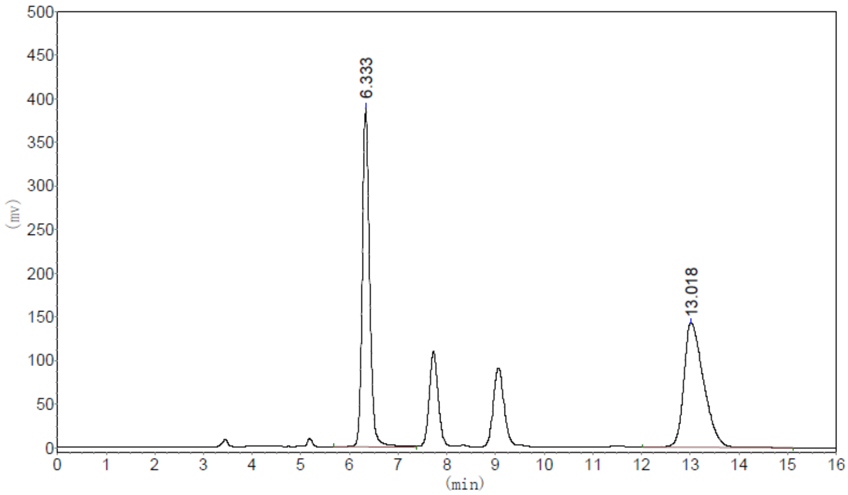

Peak No.	R. Time	Peak Height	Peak Area	Percent
1	6. 333	388195. 438	4084433. 750	50. 4636
2	13. 018	143045. 563	4009393. 750	49. 5364
Total		531241. 000	8093827. 500	100. 0000

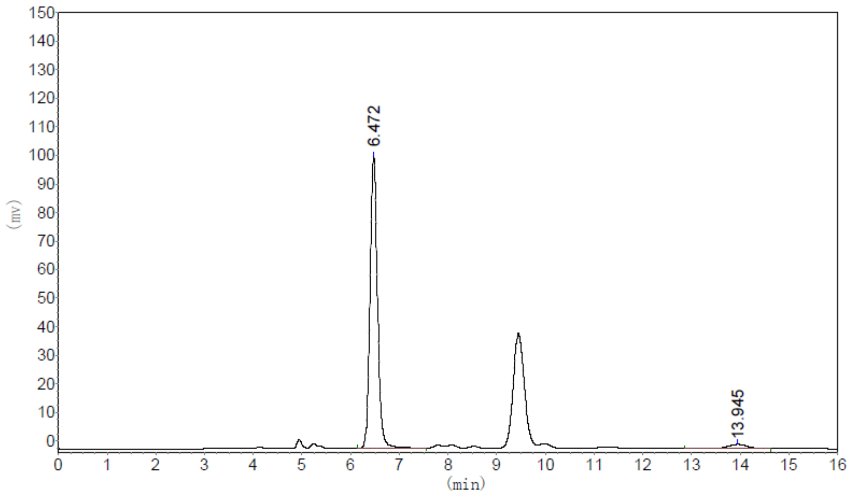

Peak No.	R. Time	Peak Height	Peak Area	Percent
1	6. 472	102123. 984	1089280. 000	96. 3931
2	13. 945	1442. 832	40759. 844	3. 6069
Total		103566. 817	1130039. 844	100. 0000

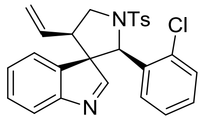

8jC

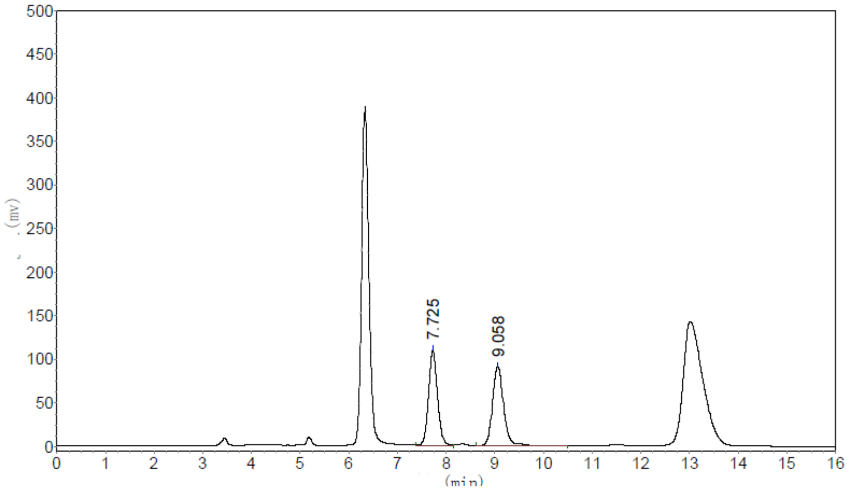

Peak No.	R. Time	Peak Height	Peak Area	Percent
1	7.725	110183.922	1414786.000	49.6169
2	9.058	90856.828	1436635.625	50.3831
Total		201040.750	2851421.625	100.0000

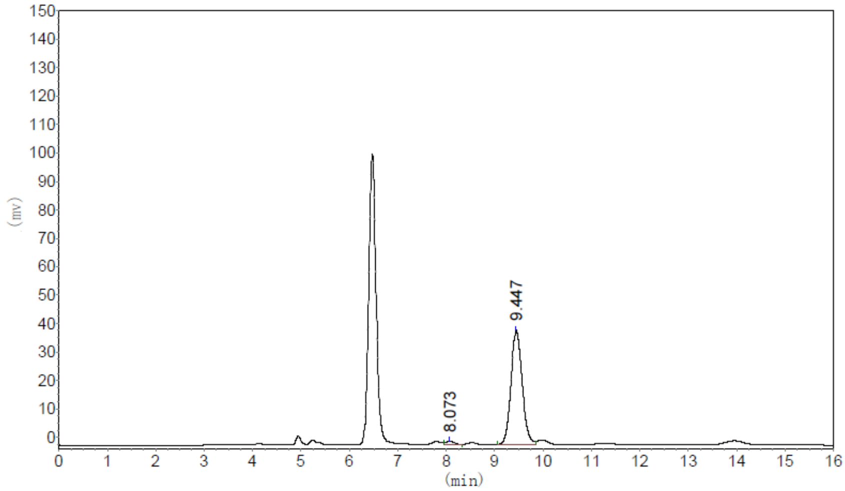

Peak No.	R. Time	Peak Height	Peak Area	Percent
1	8.073	1092.085	15492.970	2.3233
2	9.447	40077.773	651358.500	97.6767
Total		41169.858	666851.470	100.0000

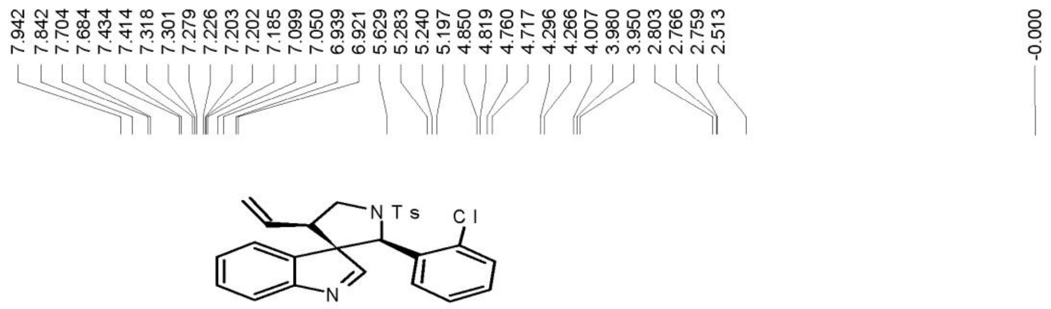
7.942
7.842
7.704
7.684
7.434
7.414
7.318
7.301
7.279
7.226
7.203
7.202
7.185
7.099
7.050
6.939
6.921
5.629
5.283
5.240
5.197
4.850
4.819
4.760
4.717
4.296
4.266
4.007
3.980
3.950
2.803
2.766
2.759
2.513
-0.000
NTs
Cl
N

8 j B

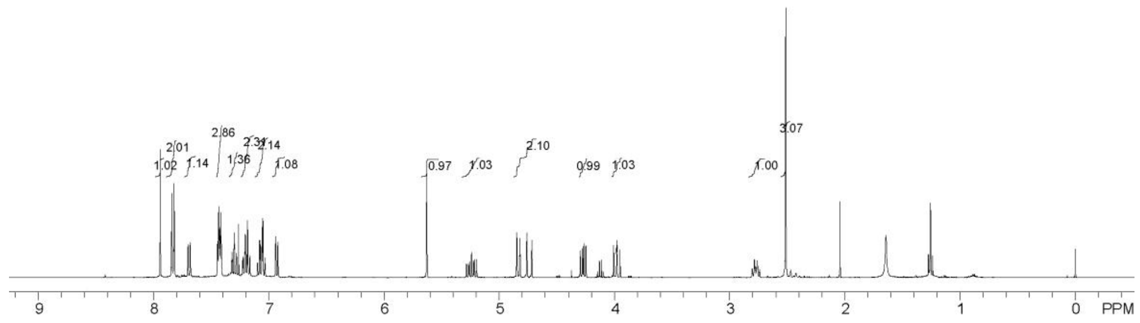
PPM

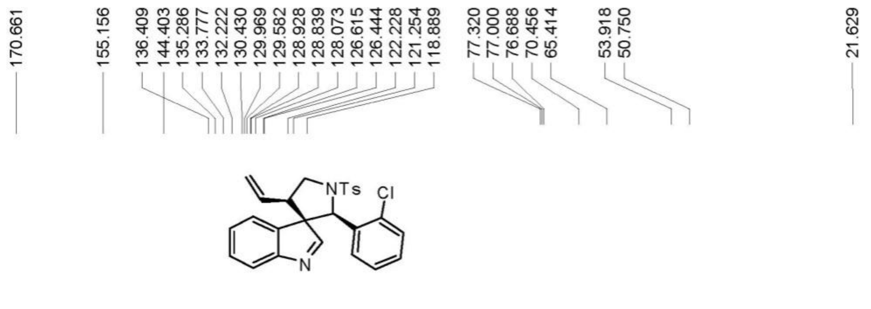
170.661
155.156
136.409
144.403
135.286
133.777
132.222
130.430
129.969
129.582
128.928
128.839
128.073
126.615
126.444
122.228
121.254
118.889
77.320
77.000
76.688
70.456
65.414
53.918
50.750
21.629
NTs
Cl
N

8jB

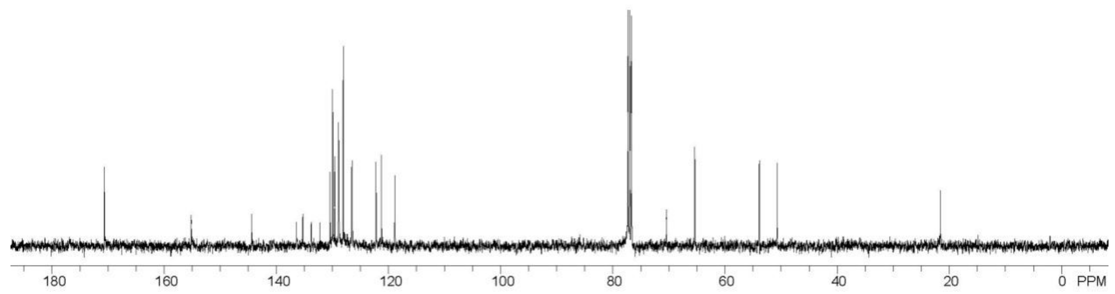
PPM

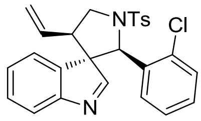

8jB

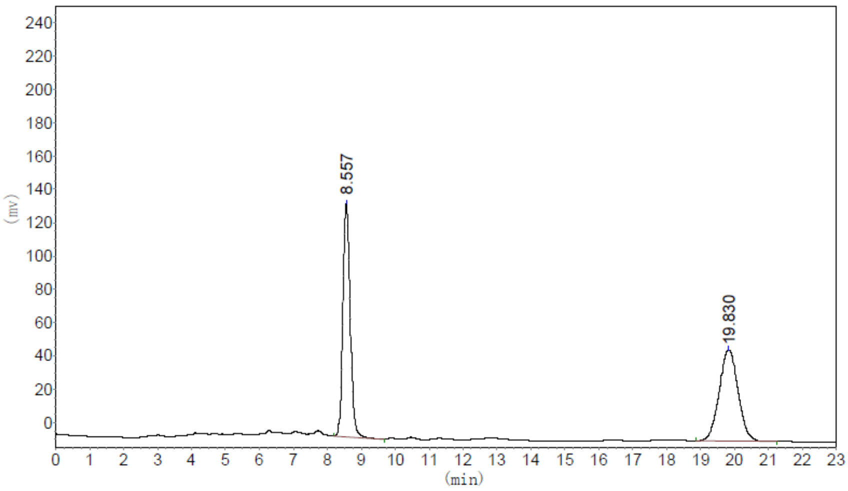

Peak No.	R. Time	Peak Height	Peak Area	Percent
1	8.557	139990.391	2080714.125	49.5766
2	19.830	54795.348	2116253.750	50.4234
Total		194785.738	4196967.875	100.0000

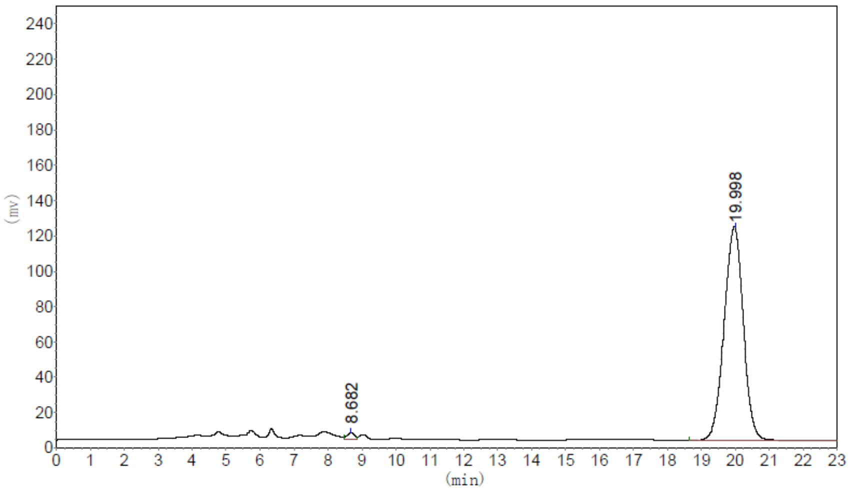

Peak No.	R. Time	Peak Height	Peak Area	Percent
1	8.682	3752.691	58354.723	1.2019
2	19.998	121116.586	4796821.500	98.7981
Total		124869.277	4855176.223	100.0000

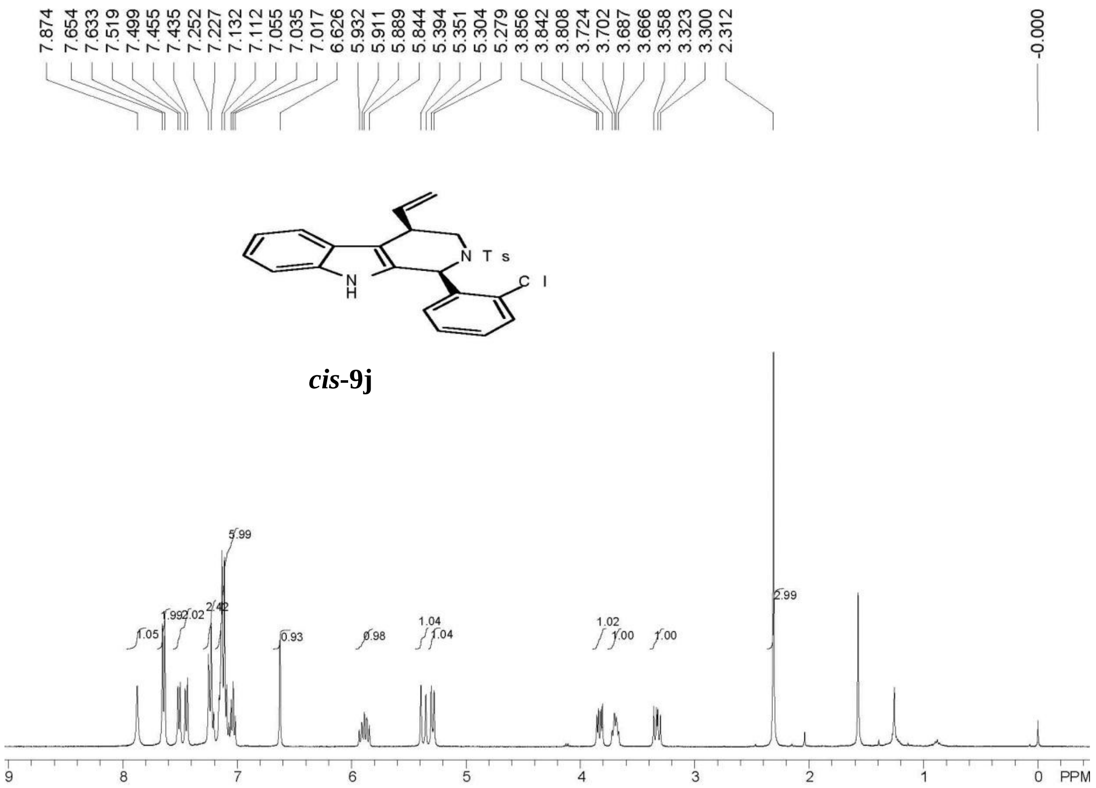
7.874 7.654 7.633 7.519 7.499 7.455 7.435 7.252 7.227 7.132 7.112 7.055 7.035 7.017 6.626 5.932 5.911 5.889 5.844 5.394 5.351 5.304 5.279 3.856 3.842 3.808 3.724 3.702 3.687 3.666 3.358 3.323 3.300 2.312
-0.000
NTs
Cl
H
1.05 1.99 2.02 2.42 5.99 0.93 0.98 1.04 1.04 1.02 1.00 1.00 2.99
9 8 7 6 5 4 3 2 1 0 PPM

***cis*-9j**

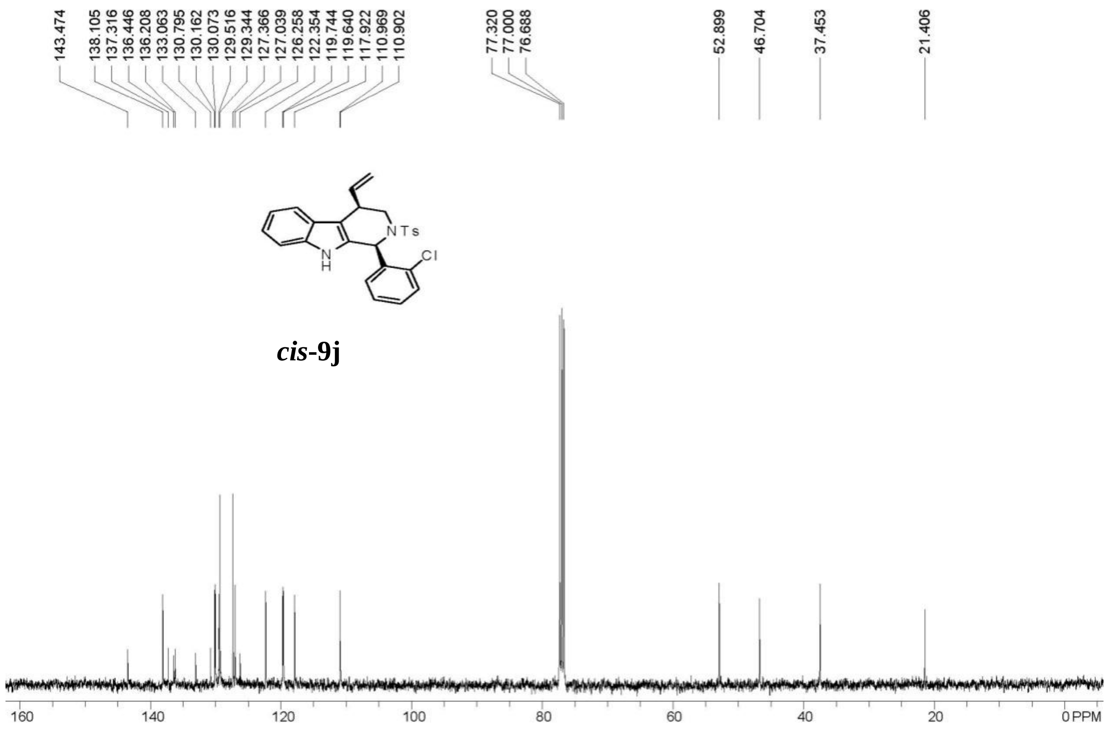
143.474 138.105 137.316 136.446 136.208 133.063 130.795 130.162 130.073 129.516 129.344 127.366 127.039 126.258 122.354 119.744 119.640 117.922 110.969 110.902
77.320 77.000 76.688
52.899 46.704 37.453 21.406
NTs
Cl
H
160 140 120 100 80 60 40 20 0 PPM

***cis*-9j**

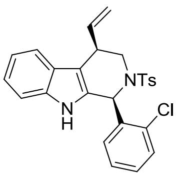

***cis*-9j**

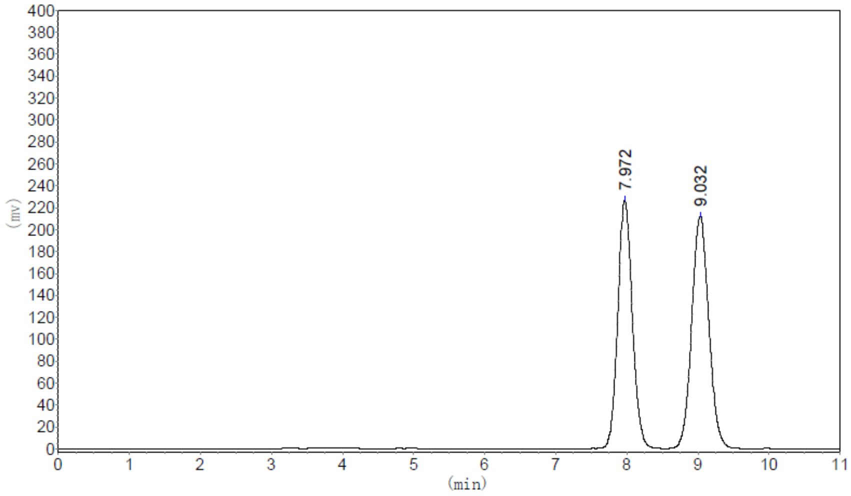

Peak No.	R. Time	Peak Height	Peak Area	Percent
1	7.972	226404.531	3208482.500	49.8604
2	9.032	204286.219	3226450.000	50.1396
Total		430690.750	6434932.500	100.0000

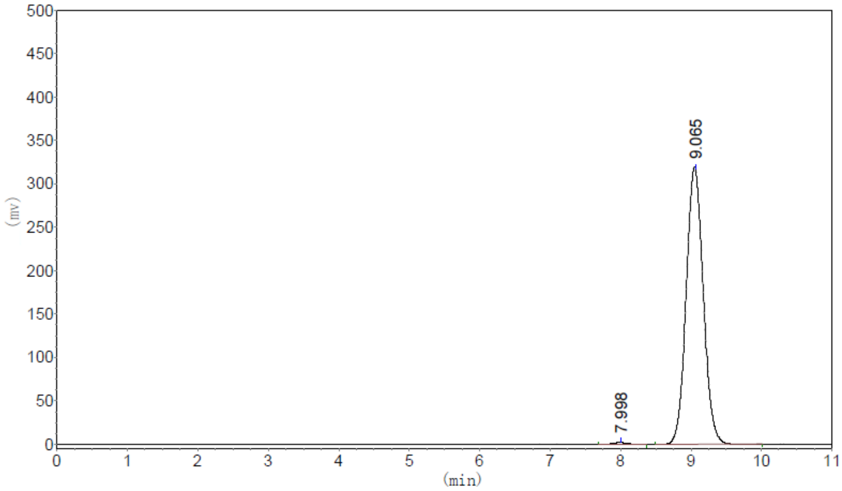

Peak No.	R. Time	Peak Height	Peak Area	Percent
1	7.998	1984.619	28369.109	0.5244
2	9.065	319035.094	5381088.000	99.4756
Total		321019.713	5409457.109	100.0000

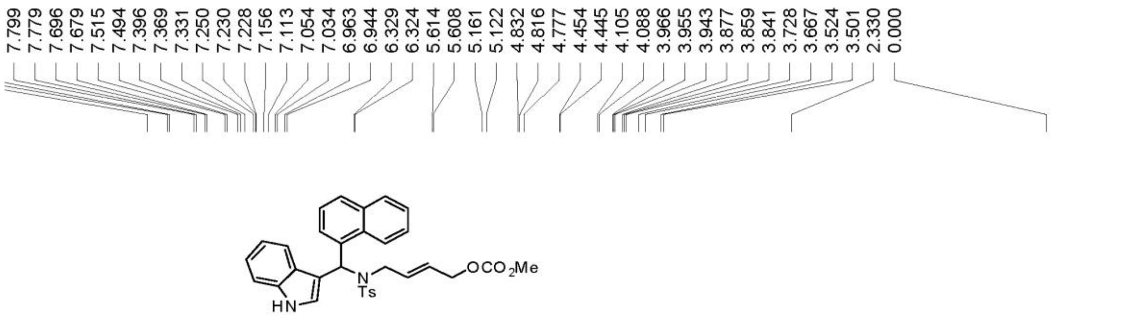
7.799
7.779
7.696
7.679
7.515
7.494
7.396
7.369
7.331
7.250
7.230
7.228
7.156
7.113
7.054
7.034
6.963
6.944
6.329
6.324
5.614
5.608
5.161
5.122
4.832
4.816
4.777
4.454
4.445
4.105
4.088
3.966
3.955
3.943
3.877
3.859
3.841
3.728
3.667
3.524
3.501
2.330
0.000
N
Ts
HN
OCO2Me

7k

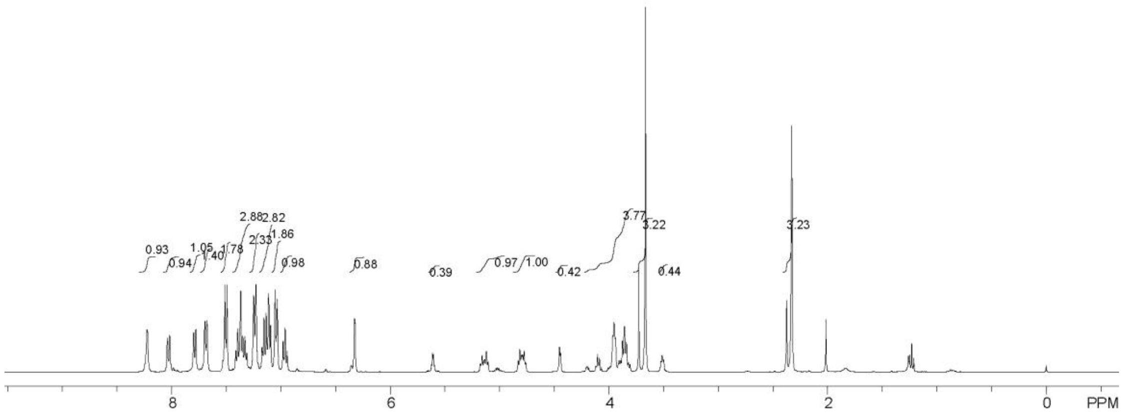
0.93
0.94
1.05
1.40
1.78
2.88
2.82
2.33
1.86
0.98
0.88
0.39
0.97
1.00
0.42
3.77
3.22
0.44
3.23
8
6
4
2
0
PPM

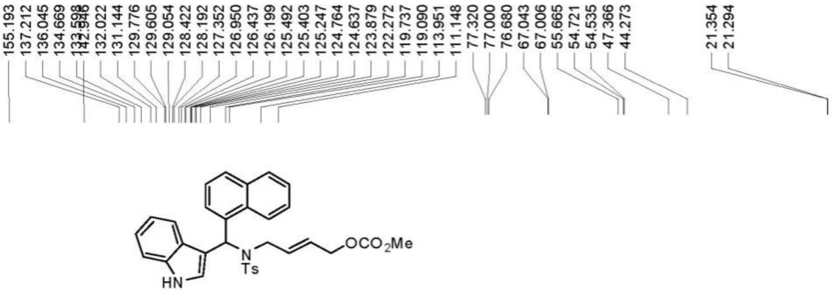
155.193
137.212
136.045
134.669
132.022
131.144
129.776
129.605
129.054
128.422
128.192
127.352
126.950
126.437
126.199
125.492
125.403
125.247
124.764
124.637
123.879
122.272
119.737
119.090
113.951
111.148
77.320
77.000
76.680
67.043
67.006
55.665
54.721
54.535
47.366
44.273
21.354
21.294
N
Ts
HN
OCO2Me

7k

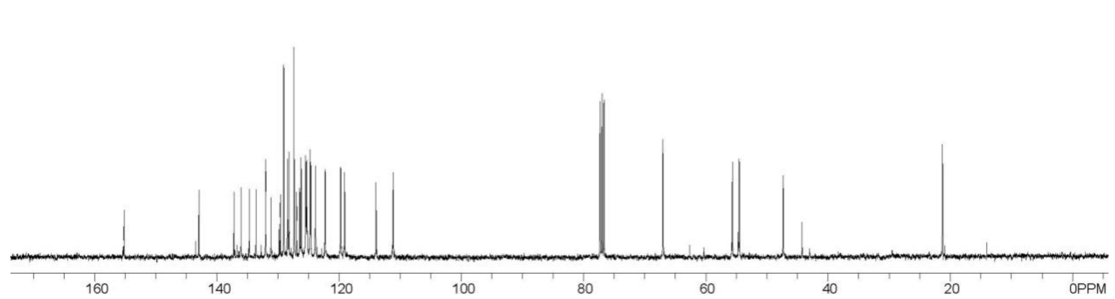
160
140
120
100
80
60
40
20
0PPM

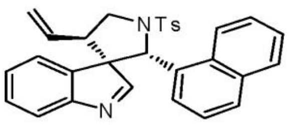

8 kA

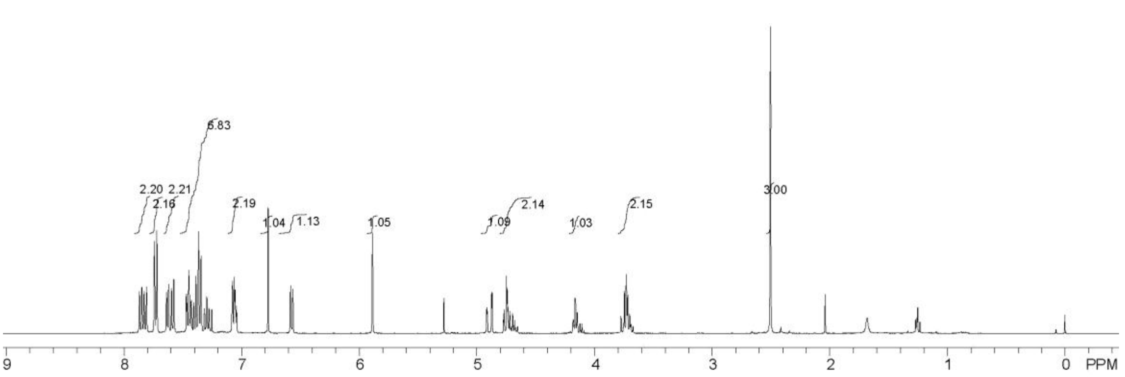
7.869 7.848 7.808 7.741 7.721 7.638 7.577 7.470 7.449 7.366 7.255 7.076 7.062 7.055 7.043 6.774 6.583 6.565 5.887 4.916 4.876 4.768 4.747 4.696 4.182 4.166 4.149 3.775 3.747 3.731 3.701 2.505 0.000
5.83
2.20 2.21
2.16
2.19
1.04 1.13
1.05
2.14
1.09
1.03
2.15
3.00
9 8 7 6 5 4 3 2 1 0 PPM

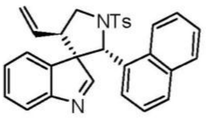

8kA

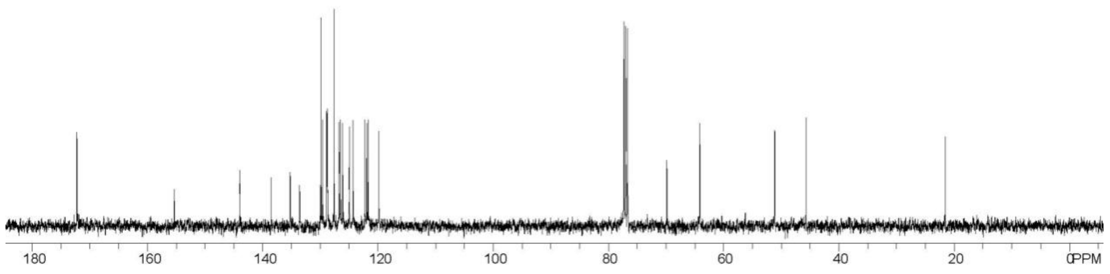
172.252 155.357 135.249 135.204 143.972 138.551 133.591 130.014 129.887 129.627 128.898 128.831 128.757 127.627 126.749 126.504 126.147 124.979 124.332 122.273 121.953 121.692 119.848 77.320 77.000 76.688 69.883 64.172 51.159 45.745 21.555
180 160 140 120 100 80 60 40 20 0PPM

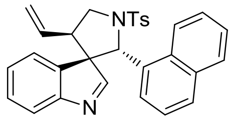

8kA

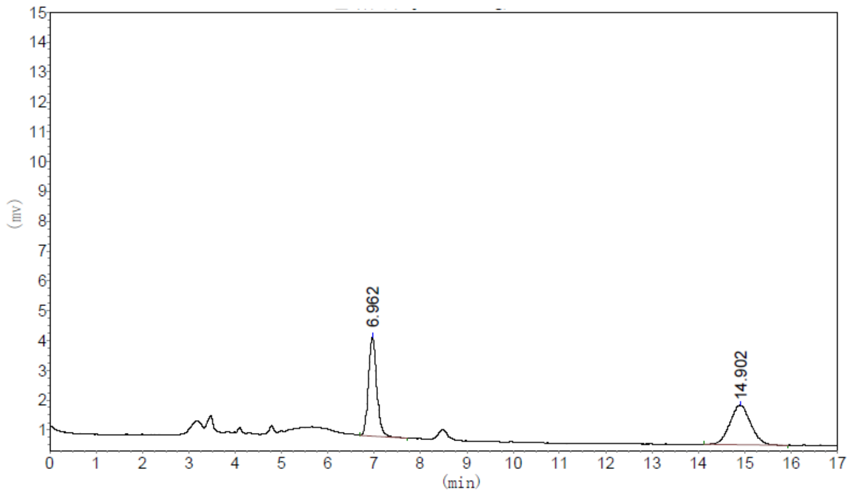

Peak No.	R. Time	Peak Height	Peak Area	Percent
1	6.962	3313.077	41673.527	50.4129
2	14.902	1315.531	40990.902	49.5871
Total		4628.608	82664.430	100.0000

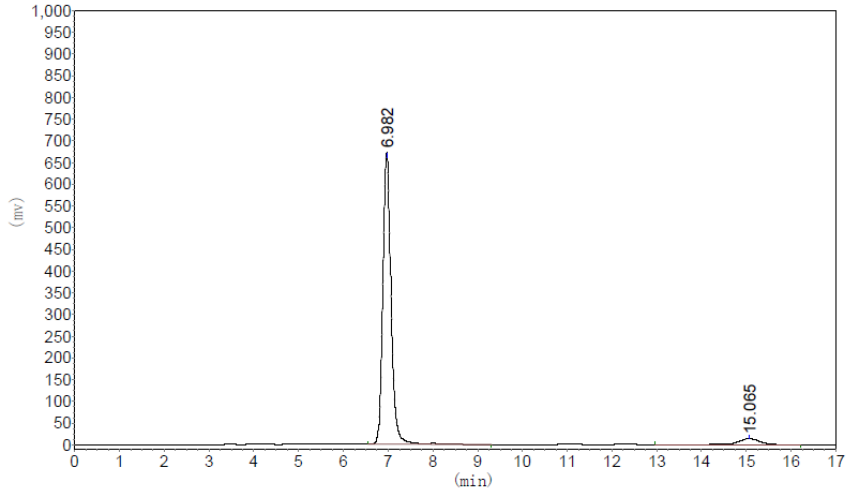

Peak No.	R. Time	Peak Height	Peak Area	Percent
1	6.982	667664.813	8600669.000	94.8133
2	15.065	13245.547	470495.500	5.1867
Total		680910.359	9071164.500	100.0000

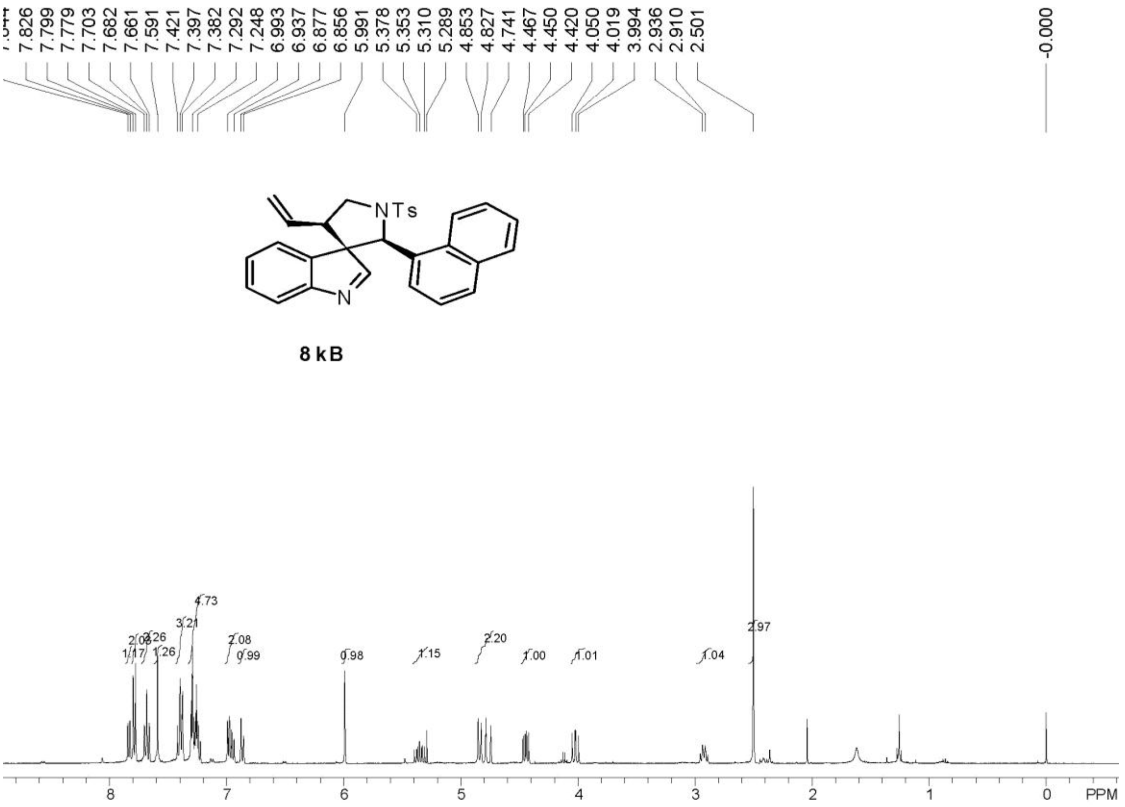
7.826 7.799 7.779 7.703 7.682 7.661 7.591 7.421 7.397 7.382 7.292 7.248 6.993 6.937 6.877 6.856 5.991 5.378 5.353 5.310 5.289 4.853 4.827 4.741 4.467 4.450 4.420 4.050 4.019 3.994 2.936 2.910 2.501
-0.000
NTs
N
1.17 2.08 3.26 1.26 3.21 4.73 2.08 0.99 0.98 1.15 2.20 1.00 1.01 1.04 2.97
8 7 6 5 4 3 2 1 0 PPM

8 kB

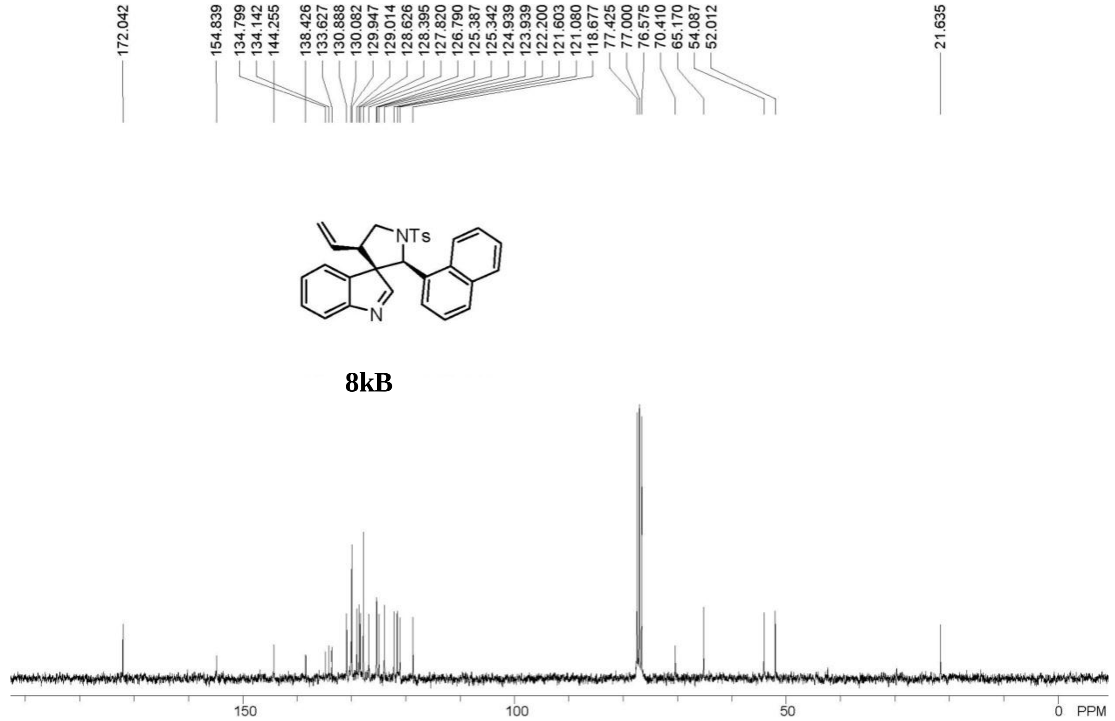
172.042 154.839 134.799 134.142 144.255 138.426 133.627 130.888 130.082 129.947 129.014 128.626 128.395 127.820 126.790 125.387 125.342 124.939 123.939 122.200 121.603 121.080 118.677 77.425 77.000 76.575 70.410 65.170 54.087 52.012 21.635
NTs
N
150 100 50 0 PPM

8kB

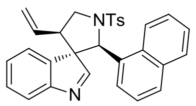

8kB

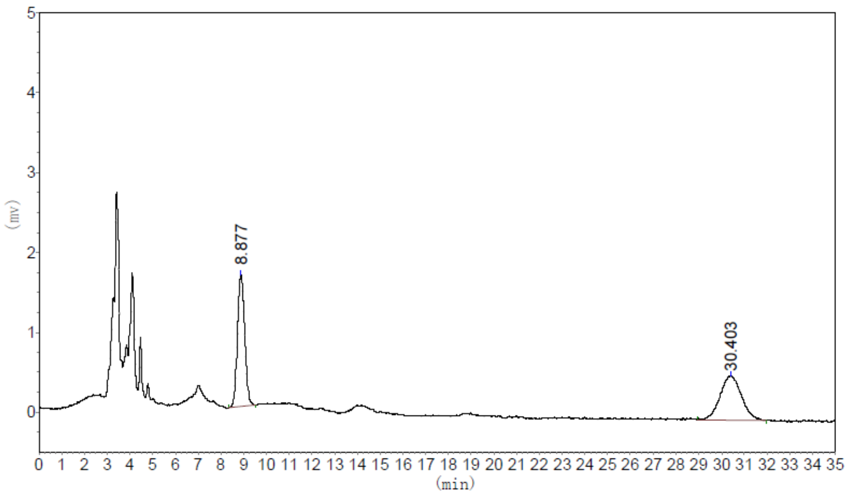

Peak No.	R. Time	Peak Height	Peak Area	Percent
1	8.877	1653.195	36181.801	50.4191
2	30.403	549.433	35580.250	49.5809
Total		2202.628	71762.051	100.0000

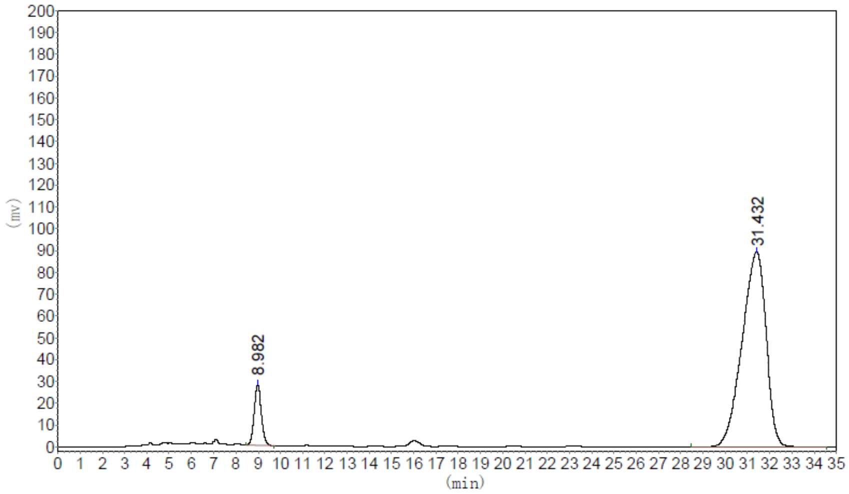

Peak No.	R. Time	Peak Height	Peak Area	Percent
1	8.982	27907.652	632888.500	8.6456
2	31.432	89573.070	6687426.000	91.3544
Total		117480.723	7320314.500	100.0000

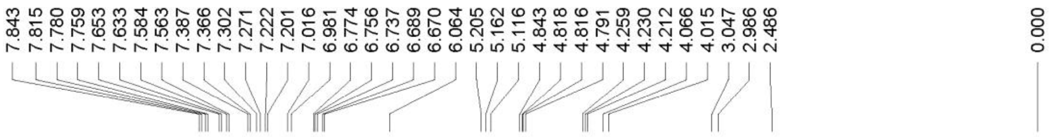
7.843
7.815
7.780
7.759
7.653
7.633
7.584
7.563
7.387
7.366
7.302
7.271
7.222
7.201
7.016
6.981
6.774
6.756
6.737
6.689
6.670
6.064
5.205
5.162
5.116
4.843
4.818
4.816
4.791
4.259
4.230
4.212
4.066
4.015
3.047
2.986
2.486
0.000

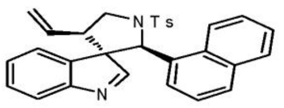
N Ts
N

8 k C

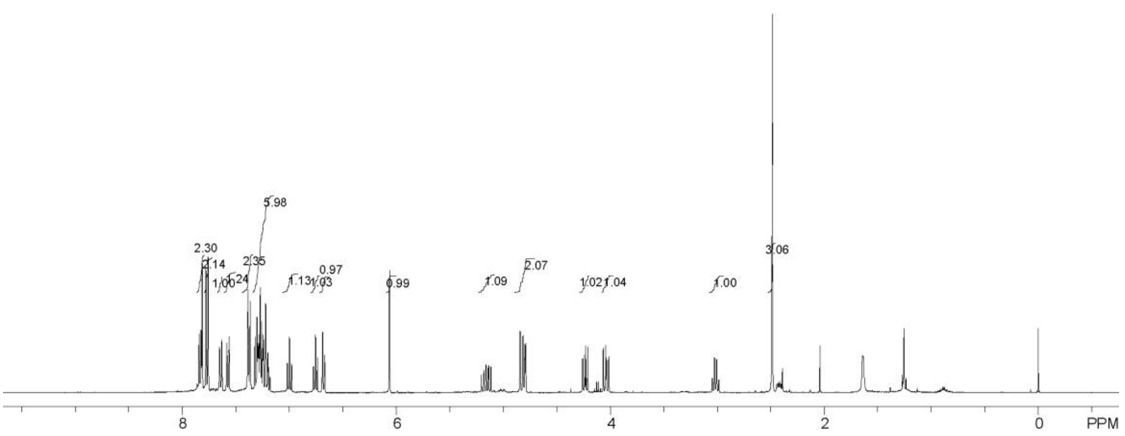
5.98
2.30
2.14
1.00
1.24
2.35
1.13
1.03
0.97
0.99
1.09
2.07
1.02
1.04
1.00
3.06
8
6
4
2
0
PPM

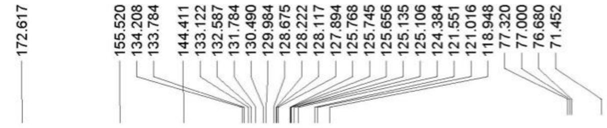
172.617
155.520
134.208
133.784
144.411
133.122
132.587
131.784
130.490
129.984
128.675
128.222
128.117
127.894
125.768
125.745
125.656
125.135
125.106
124.384
121.551
121.016
118.948
77.320
77.000
76.680
71.452
61.696
53.382
47.983
21.636

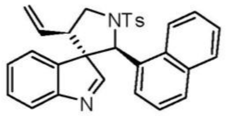
NTs
N

8kC

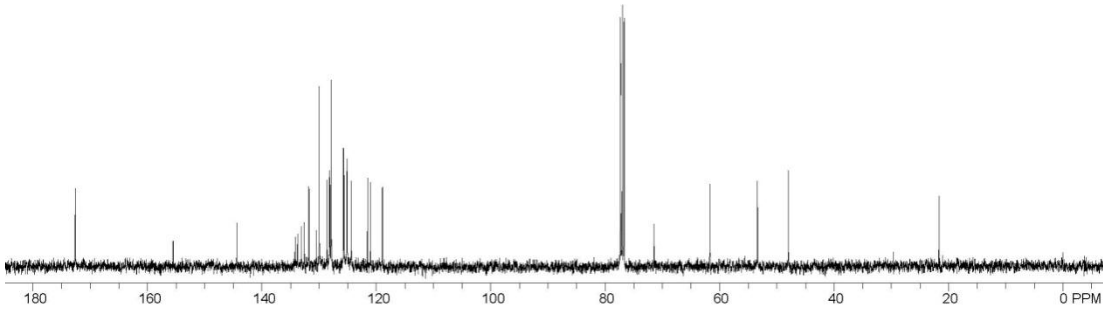
180
160
140
120
100
80
60
40
20
0 PPM

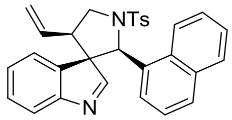

8kC

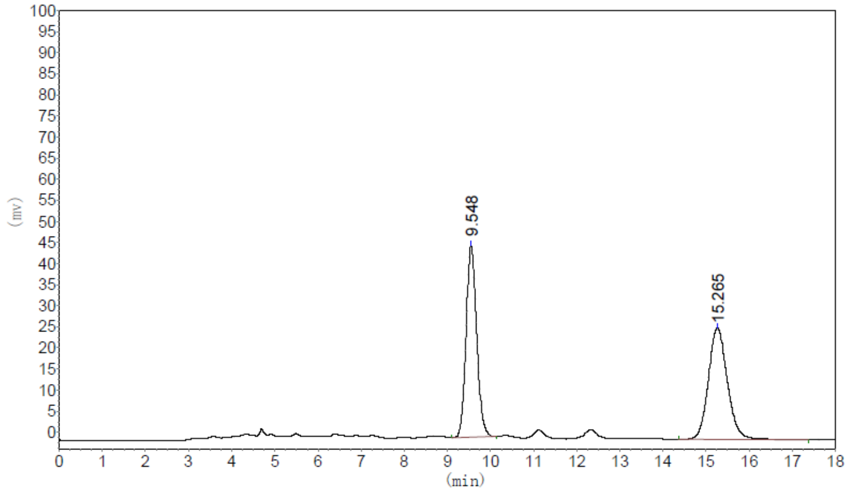

Peak No.	R. Time	Peak Height	Peak Area	Percent
1	9.548	45631.957	798076.563	49.6077
2	15.265	26356.775	810700.500	50.3923
Total		71988.732	1608777.063	100.0000

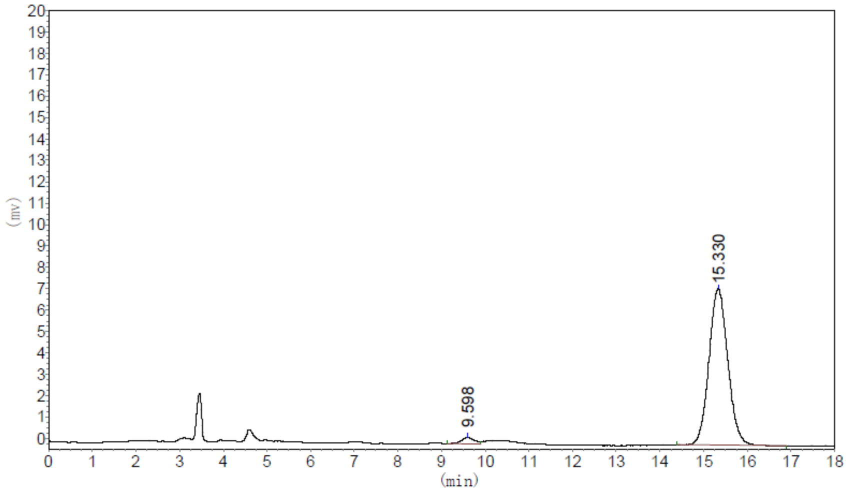

Peak No.	R. Time	Peak Height	Peak Area	Percent
1	9.598	300.883	6021.824	2.6762
2	15.330	7296.483	218993.250	97.3238
Total		7597.366	225015.074	100.0000

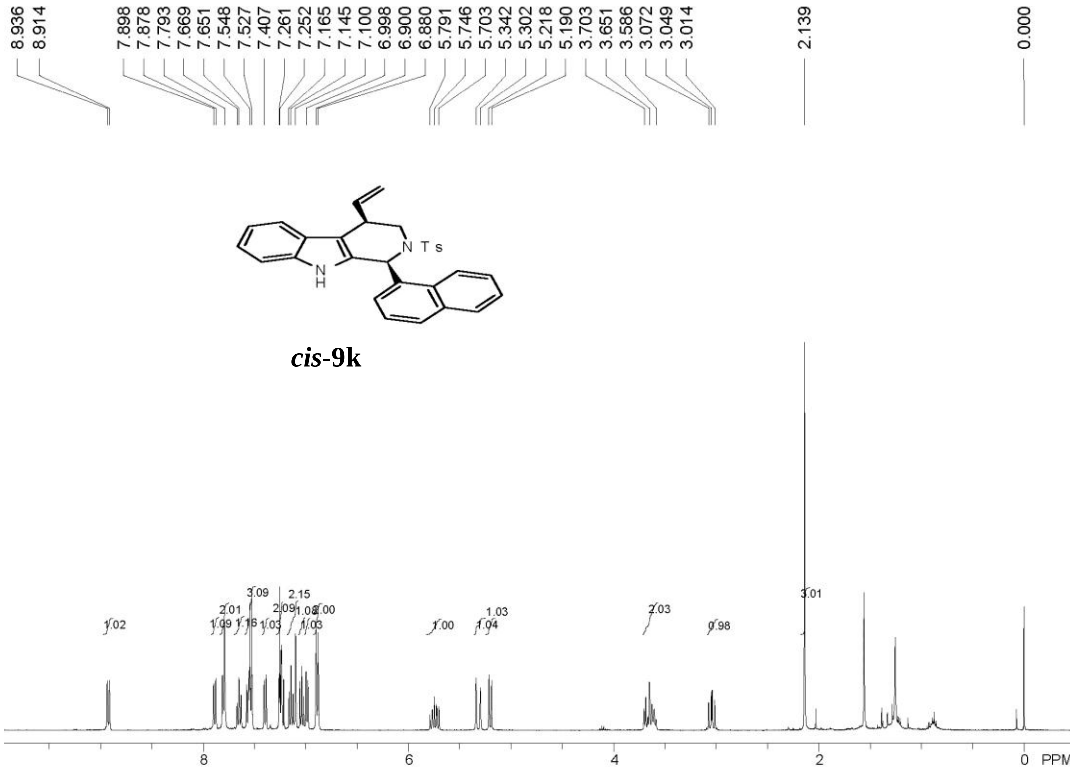

cis-**9k**

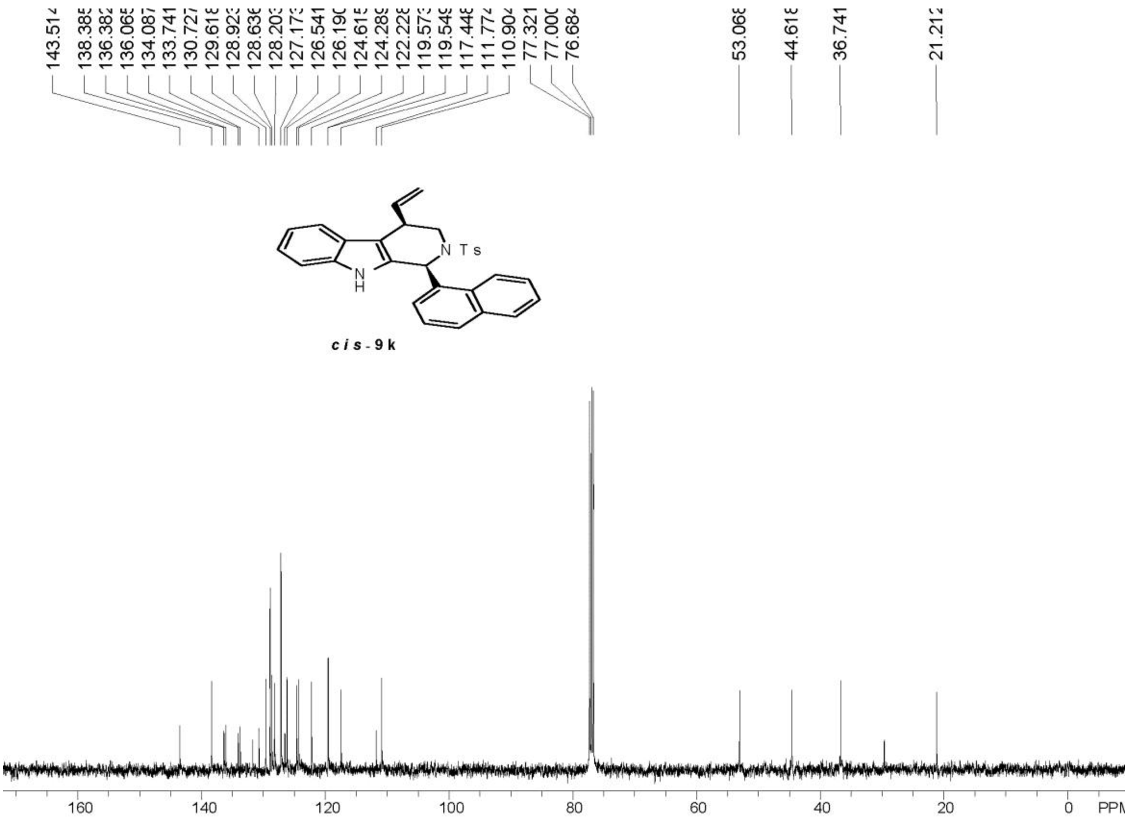

cis-9k

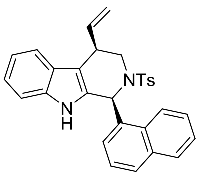

***cis*-9k**

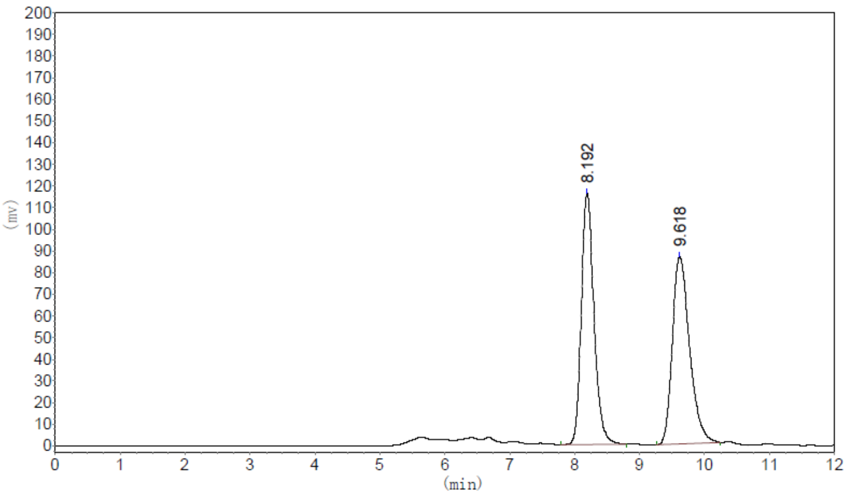

Peak No.	R. Time	Peak Height	Peak Area	Percent
1	8. 192	115865. 039	1564105. 875	49. 6055
2	9. 618	86486. 180	1588984. 000	50. 3945
Total		202351. 219	3153089. 875	100. 0000

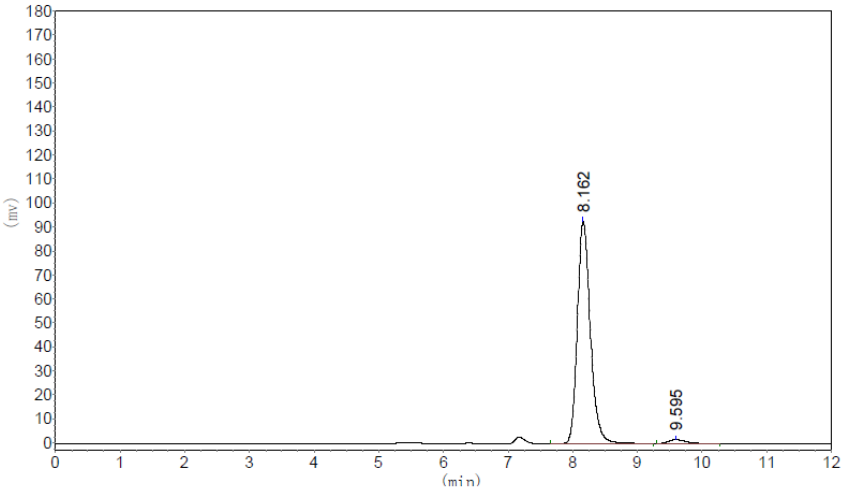

Peak No.	R. Time	Peak Height	Peak Area	Percent
1	8. 162	92437. 445	1291749. 375	97. 8256
2	9. 595	1531. 269	28712. 061	2. 1744
Total		93968. 714	1320461. 436	100. 0000

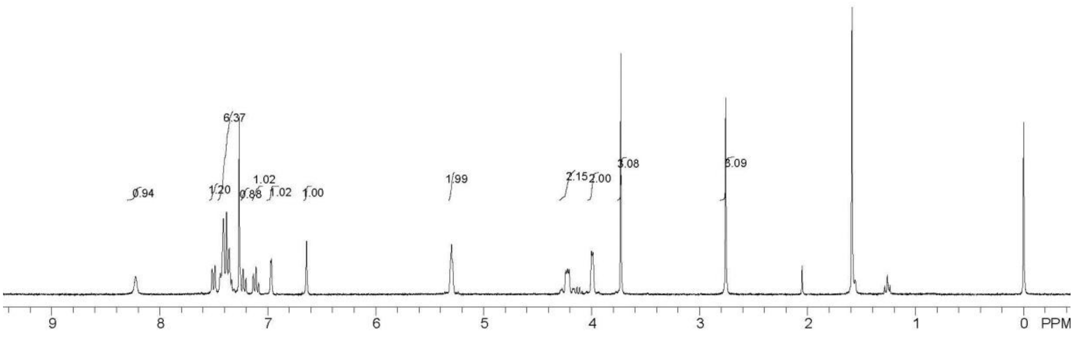
8.224
7.515
7.488
7.411
7.382
7.356
7.264
7.229
7.135
7.108
6.965
6.641
5.297
4.242
4.216
4.209
4.000
3.989
3.730
2.759
-0.000
0.94
1.20
6.37
0.88
1.02
1.02
1.00
1.99
2.15
2.00
3.08
3.09
9
8
7
6
5
4
3
2
1
0 PPM

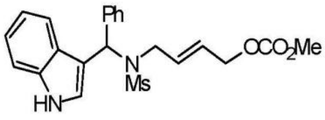
Ph
N
Ms
OCO2Me
HN

7l

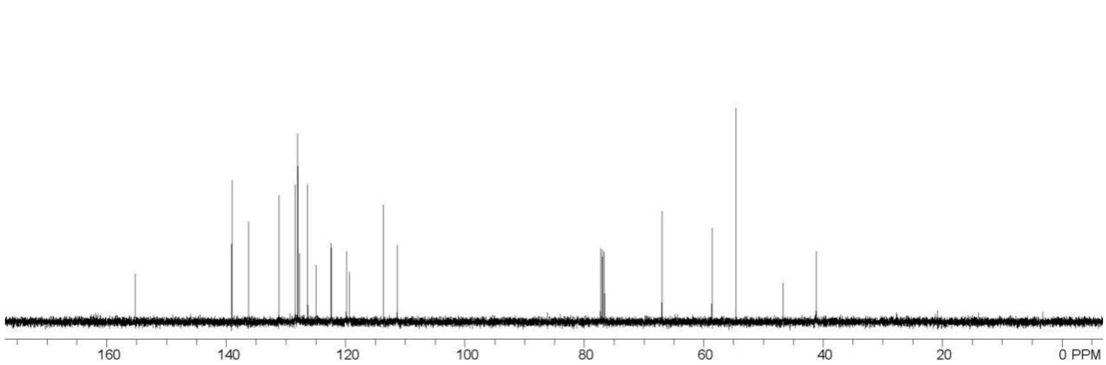
155.248
139.061
136.293
131.173
128.465
128.017
127.744
126.379
126.349
124.976
122.412
119.886
119.363
113.666
111.353
77.319
77.000
76.681
67.033
58.667
54.632
46.758
41.191
160
140
120
100
80
60
40
20
0 PPM

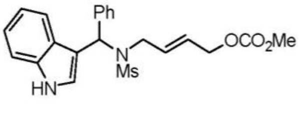
Ph
N
Ms
OCO2Me
HN

7l

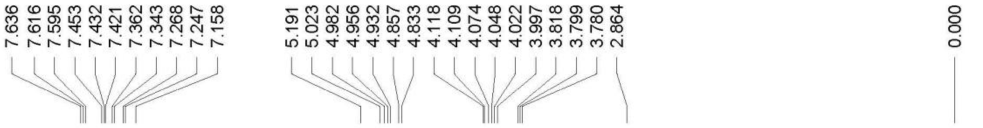
7.636
7.616
7.595
7.453
7.432
7.421
7.362
7.343
7.268
7.247
7.158
5.191
5.023
4.982
4.956
4.932
4.857
4.833
4.118
4.109
4.074
4.048
4.022
3.997
3.818
3.799
3.780
2.864
0.000

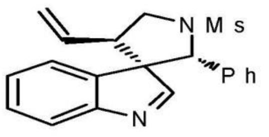
N
Ms
Ph
N

8lA

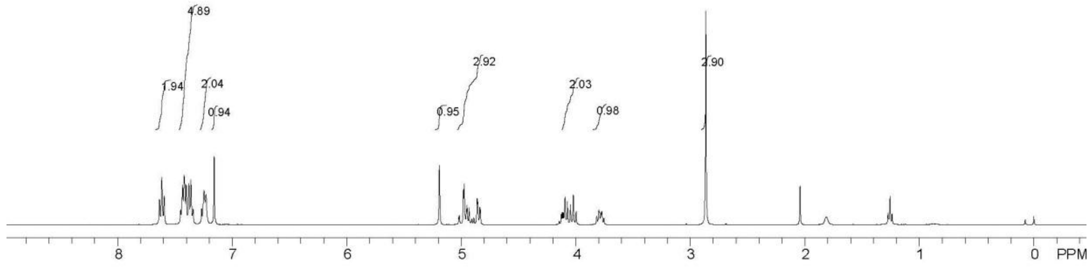
4.89
1.94
2.04
0.94
2.92
0.95
2.03
0.98
2.90
8
7
6
5
4
3
2
1
0
PPM

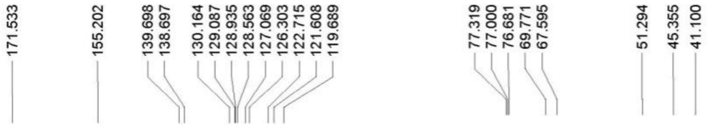
171.533
155.202
139.698
138.697
130.164
129.087
128.935
128.563
127.069
126.303
122.715
121.608
119.689
77.319
77.000
76.681
69.771
67.595
51.294
45.355
41.100

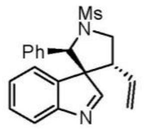
Ms
N
Ph
N

8lA

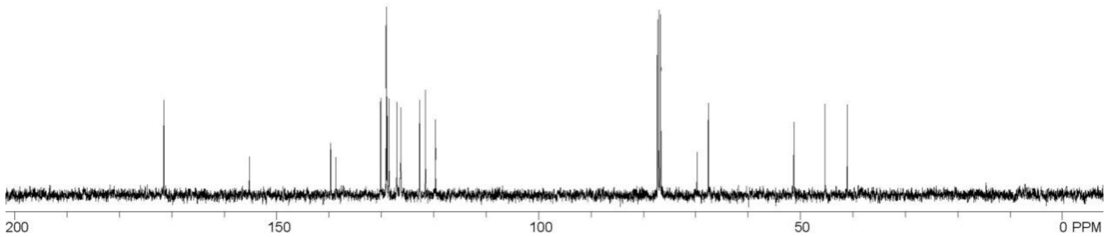
200
150
100
50
0 PPM

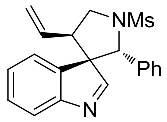

8lA

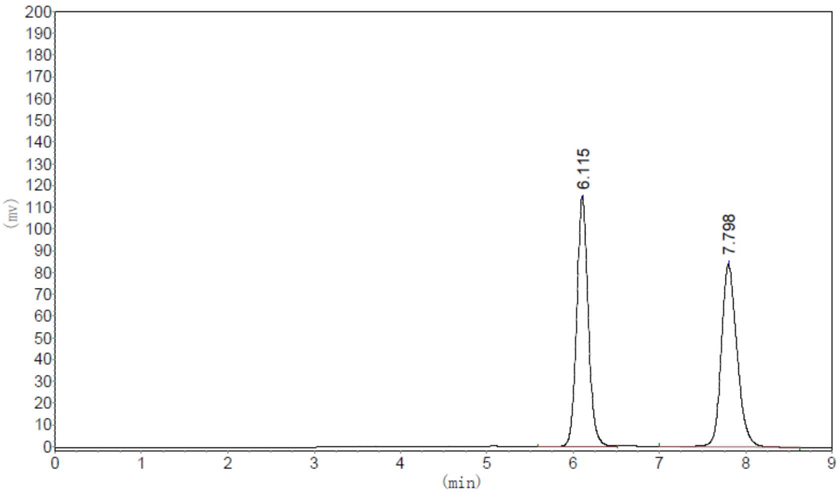

Peak No.	R. Time	Peak Height	Peak Area	Percent
1	6. 115	114879. 625	1074417. 000	50. 0532
2	7. 798	83417. 820	1072135. 000	49. 9468
Total		198297. 445	2146552. 000	100. 0000

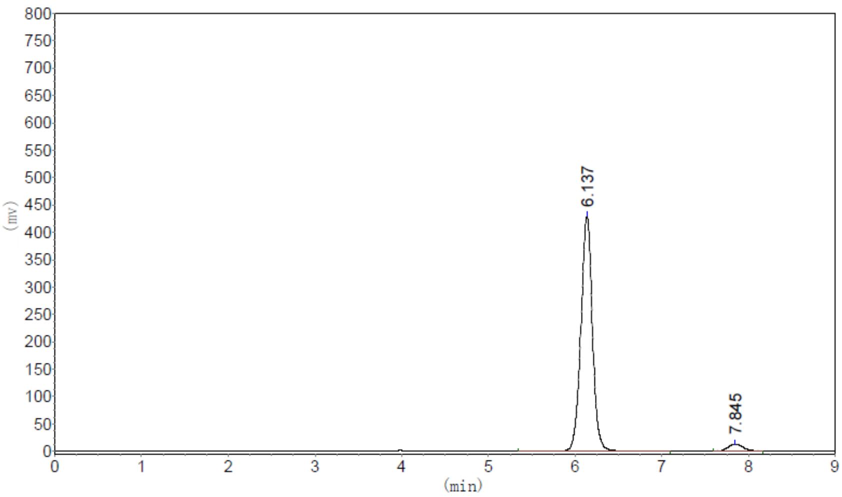

Peak No.	R. Time	Peak Height	Peak Area	Percent
1	6. 137	428757. 625	3988251. 500	96. 2866
2	7. 845	12457. 649	153809. 406	3. 7134
Total		441215. 274	4142060. 906	100. 0000

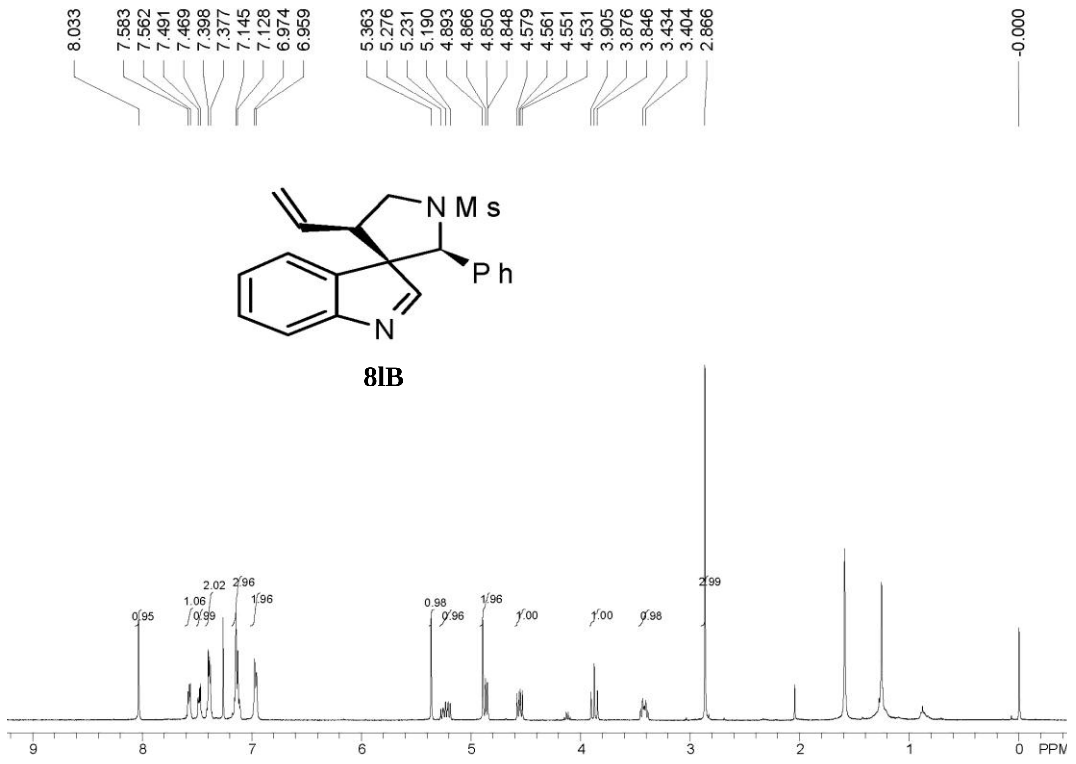
8lB

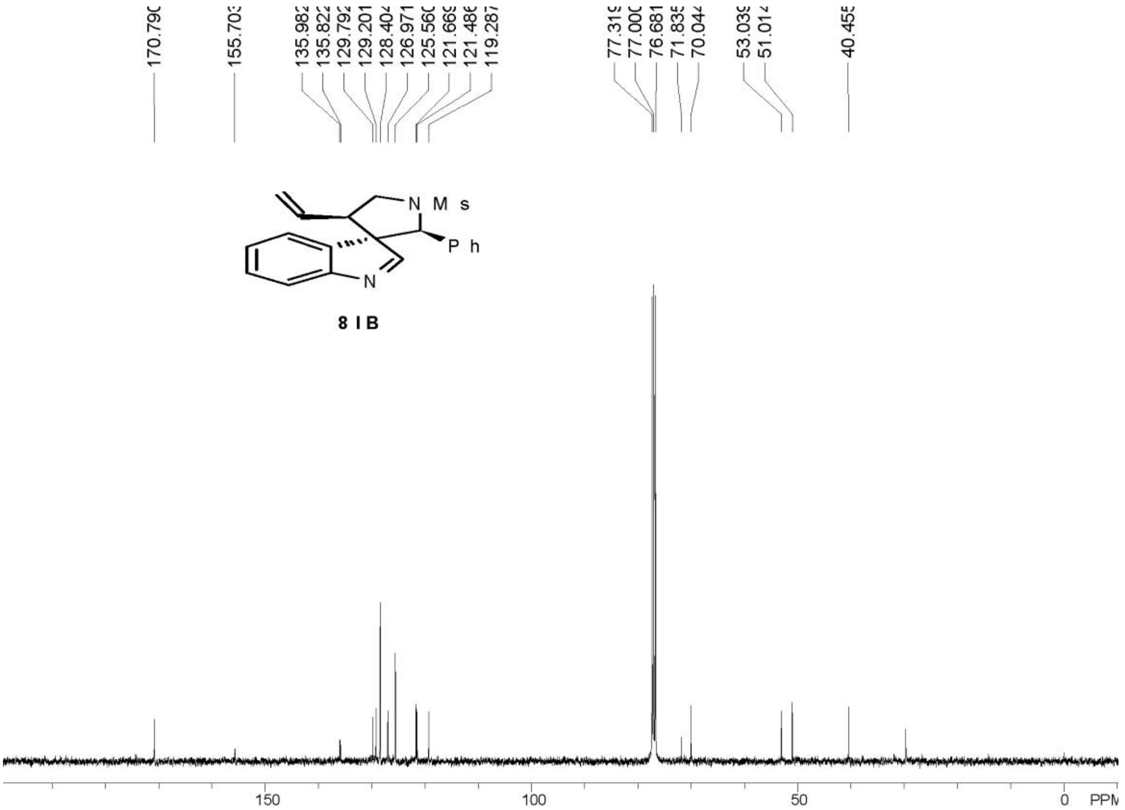
8 l B

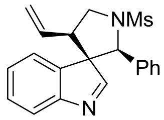

8lB

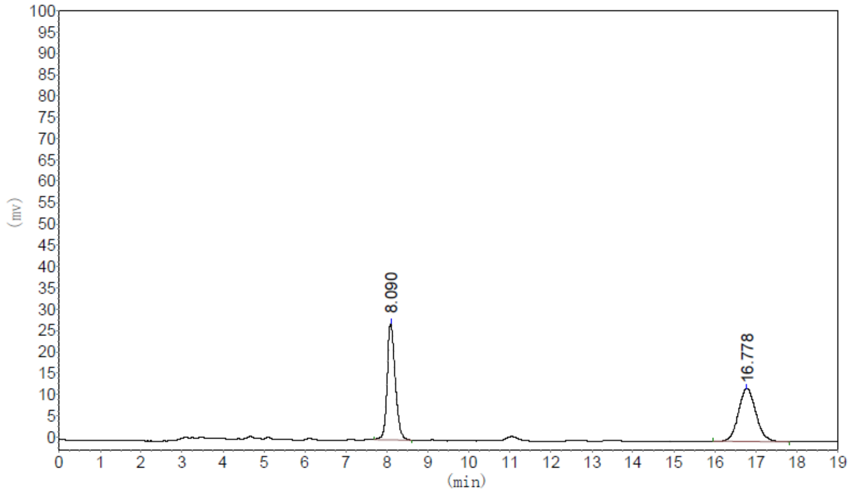

Peak No.	R. Time	Peak Height	Peak Area	Percent
1	8. 090	27276. 939	371226. 313	50. 2003
2	16. 778	12460. 710	368264. 125	49. 7997
Total		39737. 649	739490. 438	100. 0000

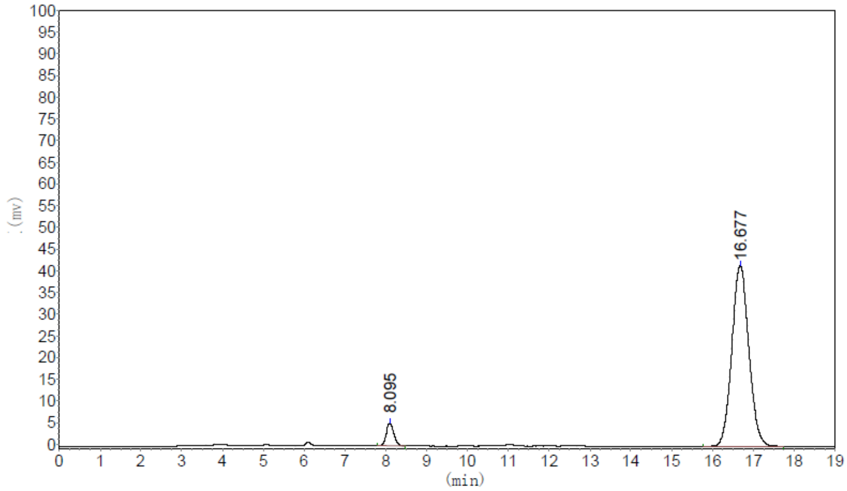

Peak No.	R. Time	Peak Height	Peak Area	Percent
1	8. 095	5227. 678	66847. 898	5. 2303
2	16. 677	41763. 570	1211234. 125	94. 7697
Total		46991. 249	1278082. 023	100. 0000

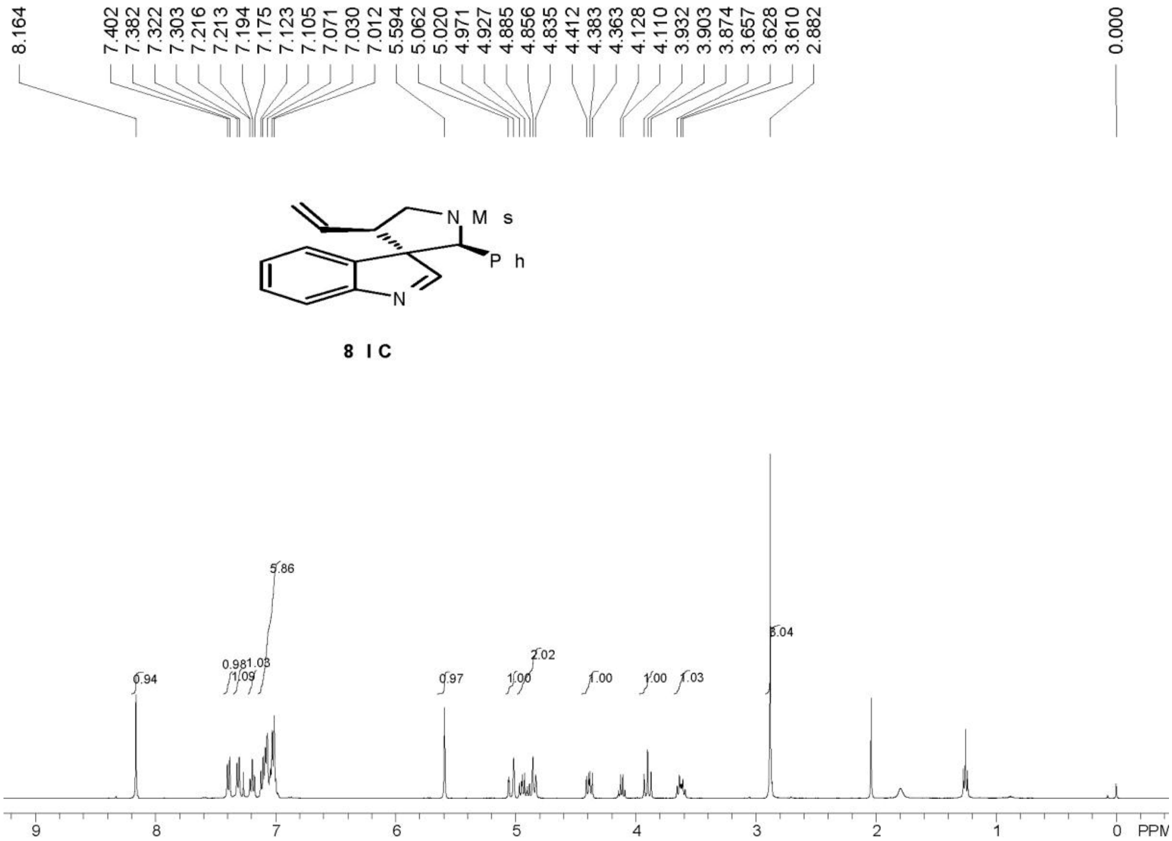
8.164
7.402
7.382
7.322
7.303
7.216
7.213
7.194
7.175
7.123
7.105
7.071
7.030
7.012
5.594
5.062
5.020
4.971
4.927
4.885
4.856
4.835
4.412
4.383
4.363
4.128
4.110
3.932
3.903
3.874
3.657
3.628
3.610
2.882
0.000
NMs
Ph
N
8IC
0.94
0.98
1.09
1.03
5.86
0.97
1.00
2.02
1.00
1.00
1.03
3.04
9
8
7
6
5
4
3
2
1
0
PPM

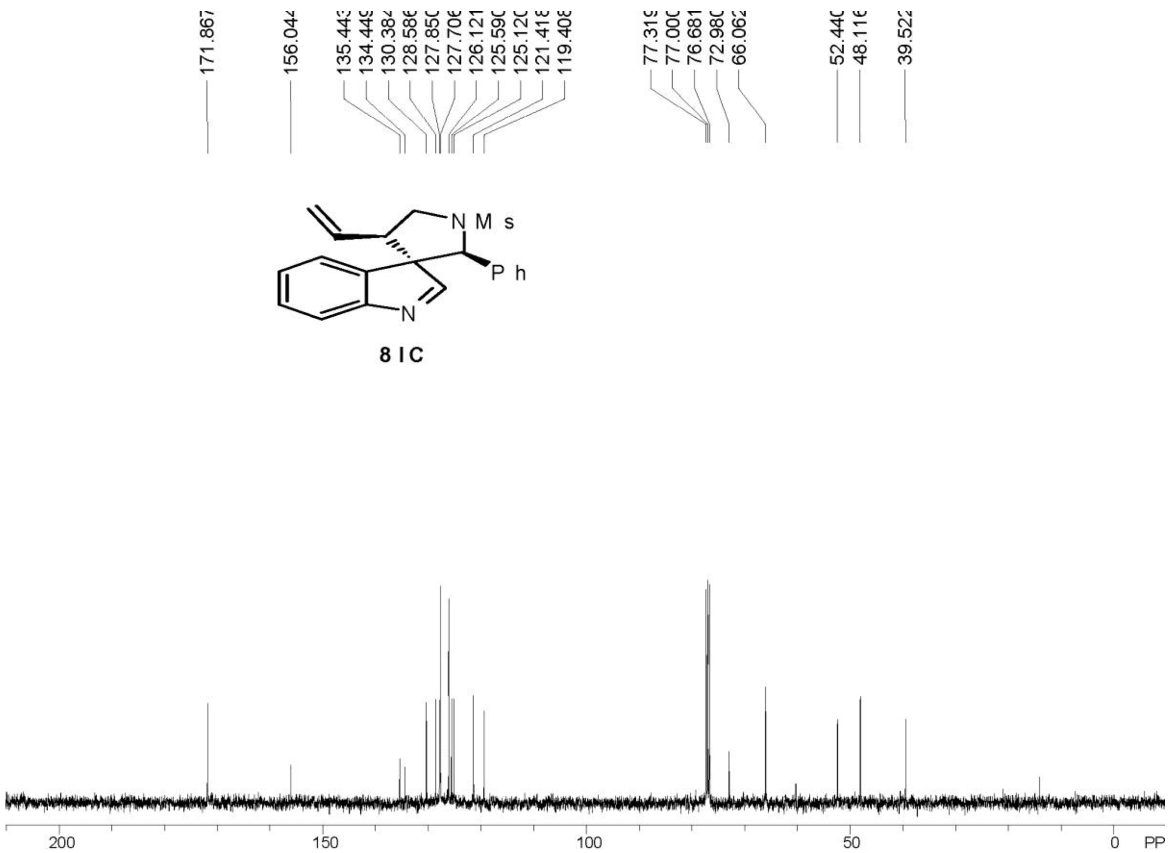
171.867
156.044
135.443
134.449
130.384
128.586
127.850
127.706
126.121
125.590
125.120
121.418
119.408
77.319
77.000
76.681
72.980
66.062
52.440
48.116
39.522
NMs
Ph
N
8IC
200
150
100
50
0
PP

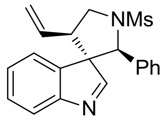

8lC

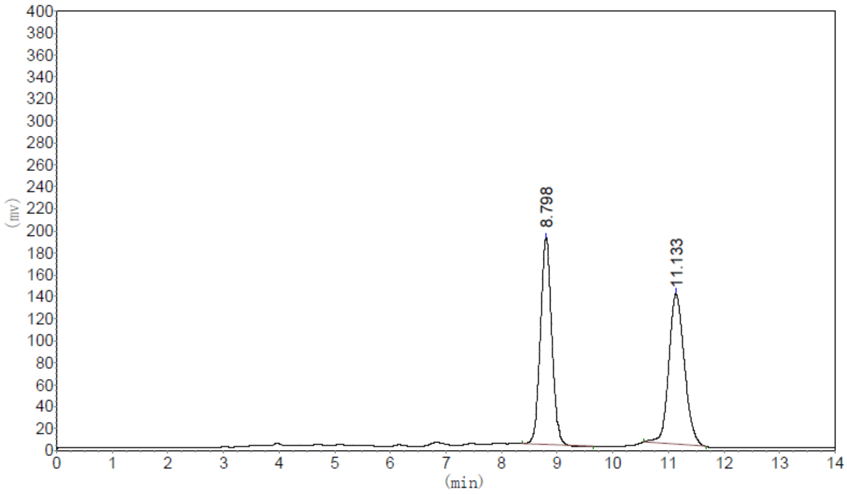

Peak No.	R. Time	Peak Height	Peak Area	Percent
1	8. 798	188486. 938	2663113. 750	50. 0970
2	11. 133	137173. 469	2652802. 250	49. 9030
Total		325660. 406	5315916. 000	100. 0000

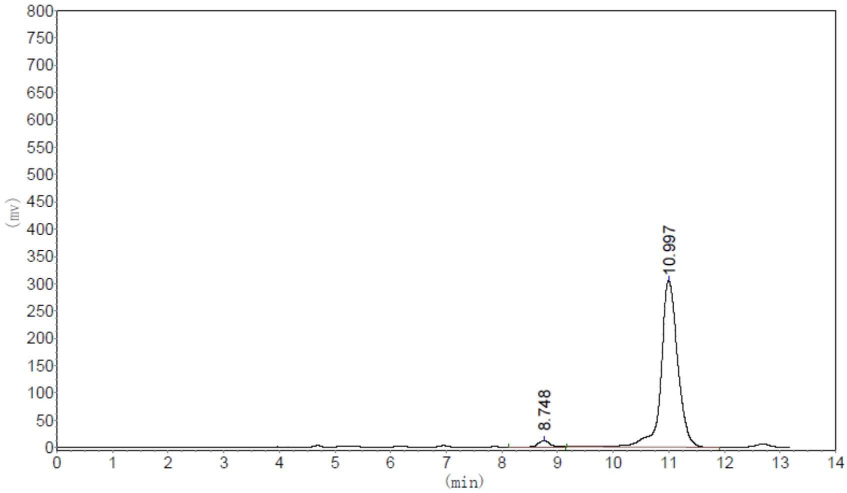

Peak No.	R. Time	Peak Height	Peak Area	Percent
1	8. 748	11183. 075	173611. 328	2. 6254
2	10. 997	305748. 625	6439116. 000	97. 3746
Total		316931. 700	6612727. 328	100. 0000

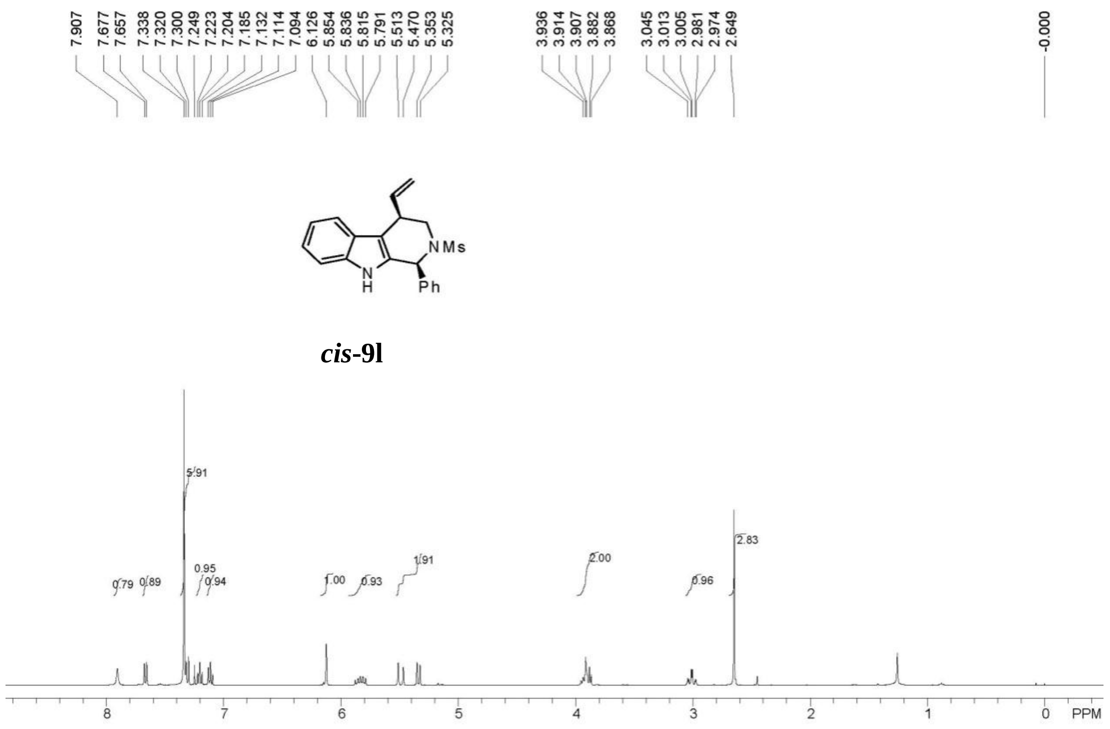
7.907
7.677
7.657
7.338
7.320
7.300
7.249
7.223
7.204
7.185
7.132
7.114
7.094
6.126
5.854
5.836
5.815
5.791
5.513
5.470
5.353
5.325
3.936
3.914
3.907
3.882
3.868
3.045
3.013
3.005
2.981
2.974
2.649
-0.000
NMs
N
H
Ph
cis-9l
5.91
0.95
0.79
0.89
0.94
1.00
0.93
1.91
2.00
0.96
2.83
8
7
6
5
4
3
2
1
0
PPM

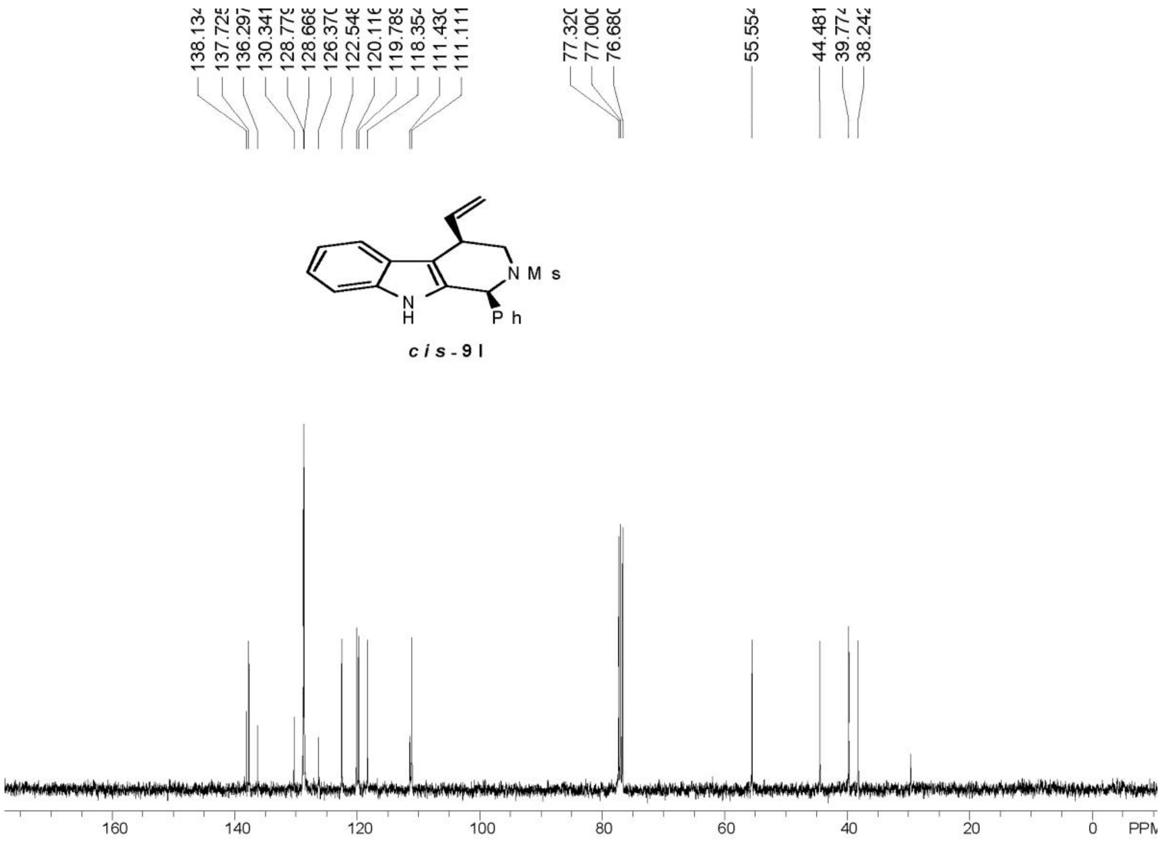
138.134
137.725
136.297
130.341
128.779
128.668
126.370
122.548
120.116
119.789
118.354
111.430
111.111
77.320
77.000
76.680
55.554
44.481
39.774
38.242
N Ms
N
H
Ph
cis-9l
160
140
120
100
80
60
40
20
0
PPM

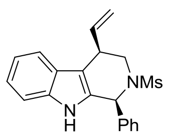

***cis*-9l**

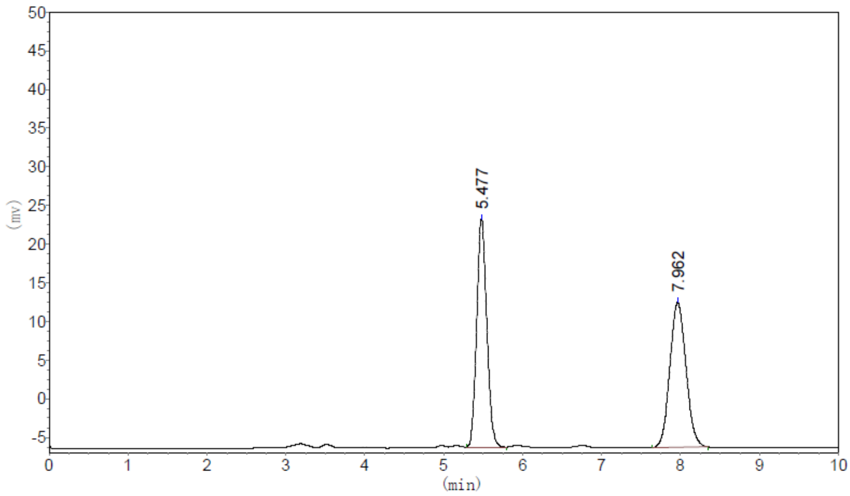

Peak No.	R. Time	Peak Height	Peak Area	Percent
1	5.477	29449.871	261695.750	49.5950
2	7.962	18788.588	265969.594	50.4050
Total		48238.459	527665.344	100.0000

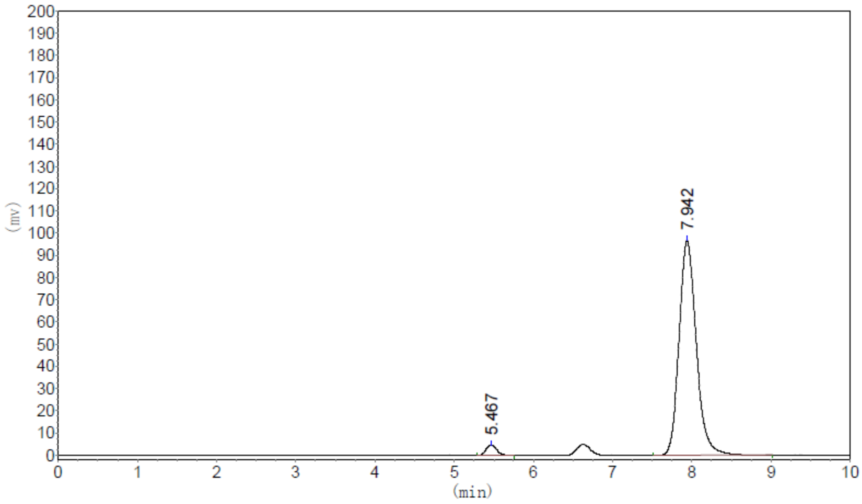

Peak No.	R. Time	Peak Height	Peak Area	Percent
1	5.467	4624.216	41947.398	2.7933
2	7.942	96830.805	1459743.250	97.2067
Total		101455.021	1501690.648	100.0000

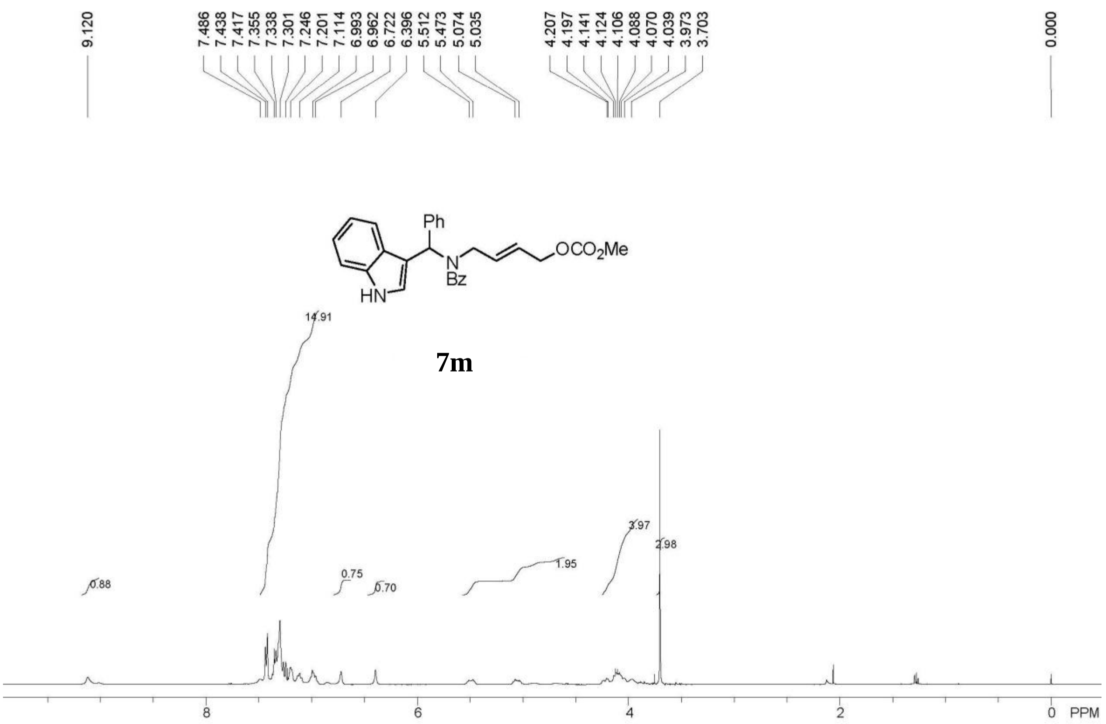

9.120
7.486
7.438
7.417
7.355
7.338
7.301
7.246
7.201
7.114
6.993
6.962
6.722
6.396
5.512
5.473
5.074
5.035
4.207
4.197
4.141
4.124
4.106
4.088
4.070
4.039
3.973
3.703
0.000
Ph
N
Bz
HN
OCO2Me
7m
14.91
0.88
0.75
0.70
1.95
3.97
2.98
8
6
4
2
0 PPM

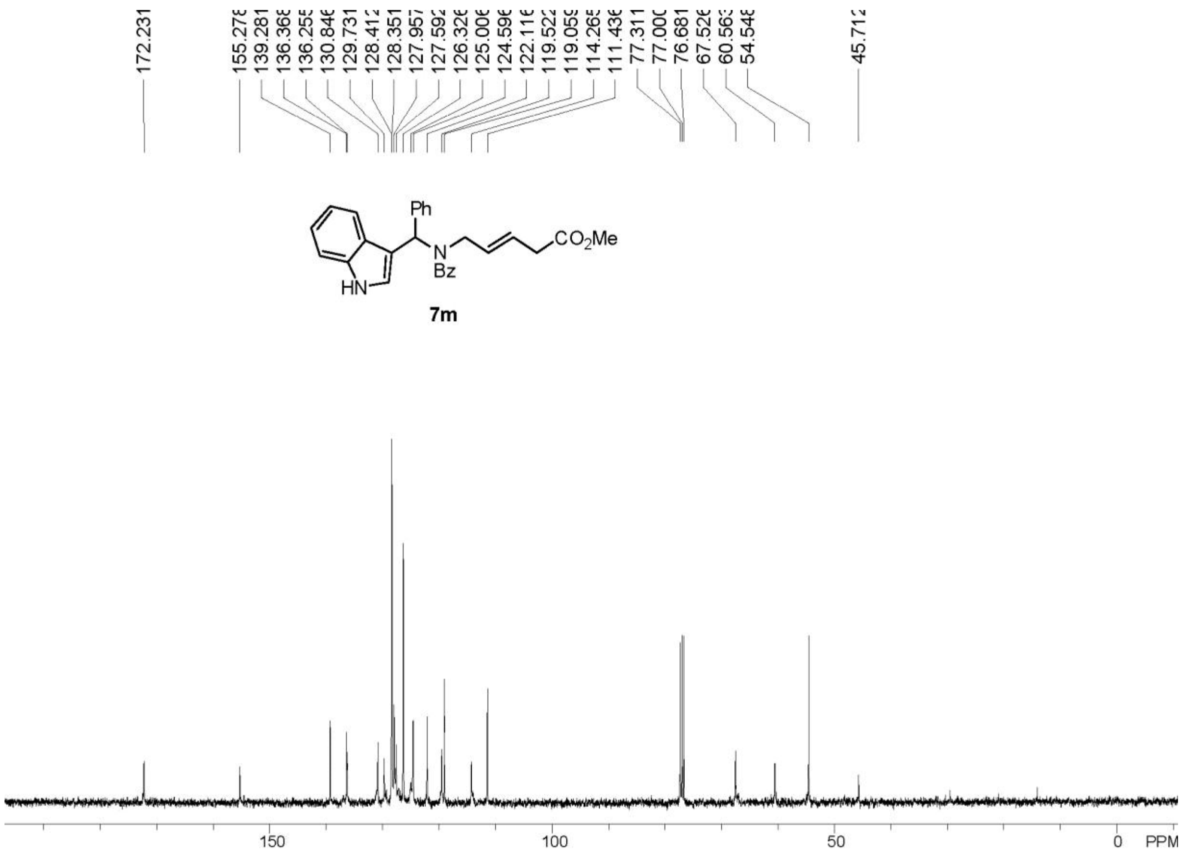

172.231
155.278
139.281
136.368
136.255
130.846
129.731
128.412
128.351
127.957
127.592
126.326
125.006
124.596
122.116
119.522
119.059
114.265
111.436
77.311
77.000
76.681
67.526
60.563
54.548
45.712
Ph
N
Bz
HN
CO2Me
7m
150
100
50
0 PPM

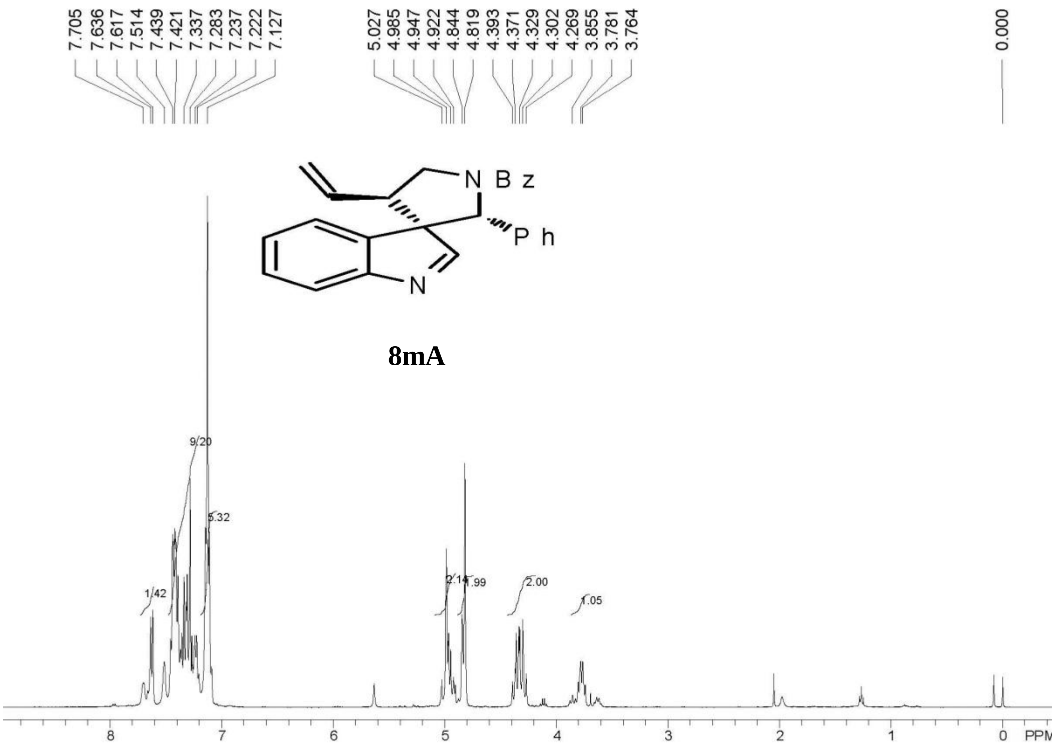
7.705
7.636
7.617
7.514
7.439
7.421
7.337
7.283
7.237
7.222
7.127
5.027
4.985
4.947
4.922
4.844
4.819
4.393
4.371
4.329
4.302
4.269
3.855
3.781
3.764
0.000
N Bz
Ph
N
1.42
9.20
5.32
2.14
1.99
2.00
1.05
PPM

8mA

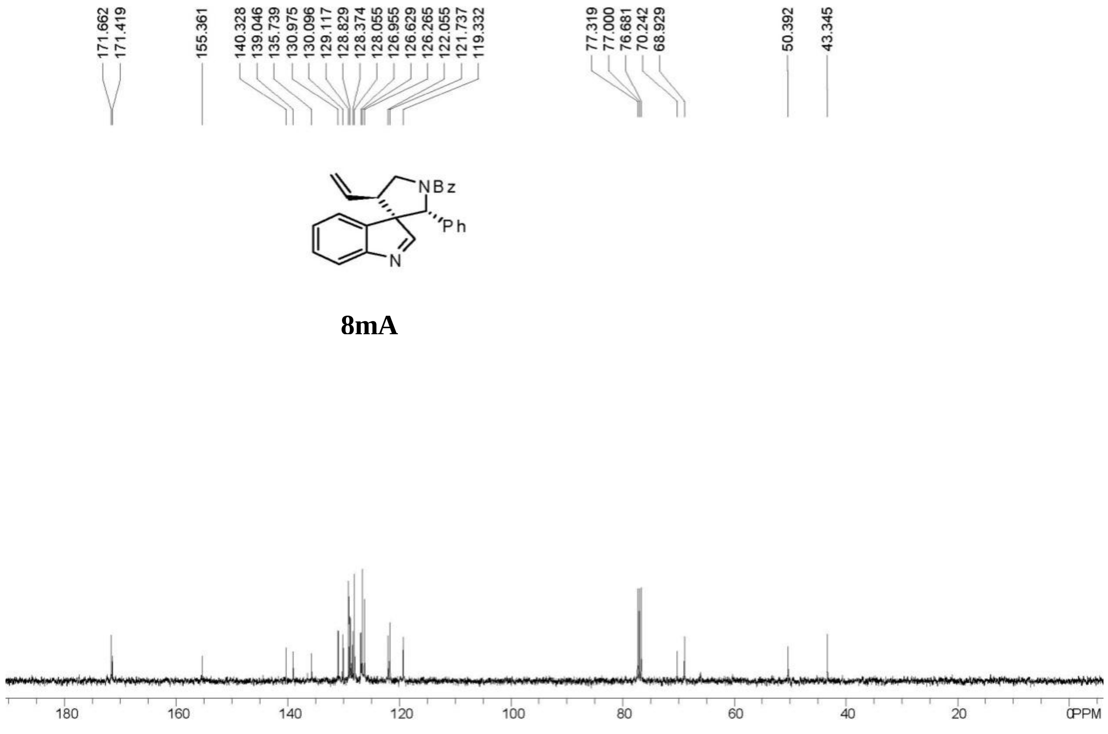
171.662
171.419
155.361
140.328
139.046
135.739
130.975
130.096
129.117
128.829
128.374
128.055
126.955
126.629
126.265
122.055
121.737
119.332
77.319
77.000
76.681
70.242
68.929
50.392
43.345
NBz
Ph
N
PPM

8mA

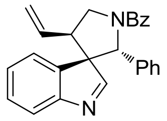

8mA

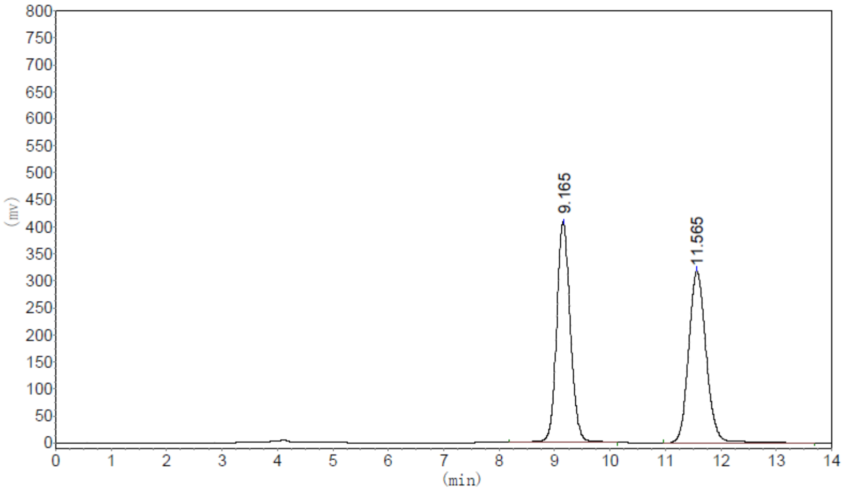

Peak No.	R. Time	Peak Height	Peak Area	Percent
1	9. 165	407913. 031	7108364. 500	50. 4217
2	11. 565	315481. 156	6989471. 000	49. 5783
Total		723394. 188	14097835. 500	100. 0000

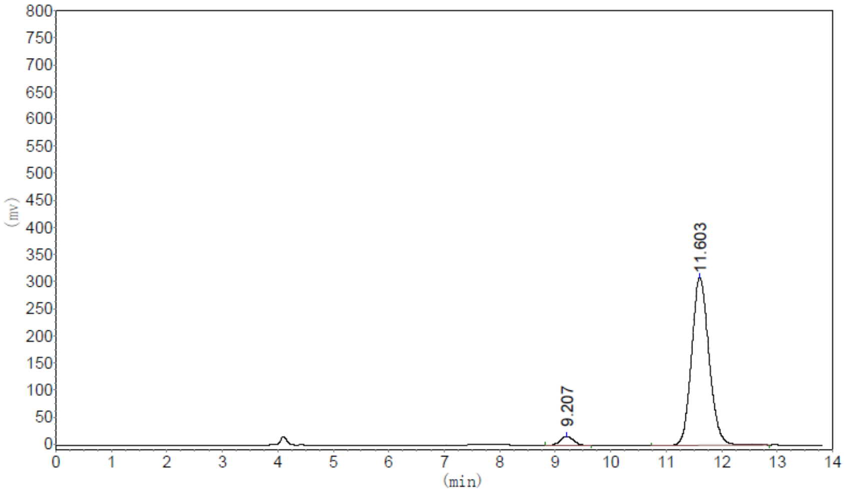

Peak No.	R. Time	Peak Height	Peak Area	Percent
1	9. 207	16021. 723	263334. 594	3. 7543
2	11. 603	308461. 688	6750815. 000	96. 2457
Total		324483. 410	7014149. 594	100. 0000

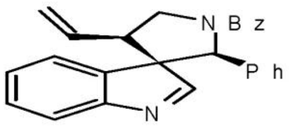
N Bz
Ph
N

8mB

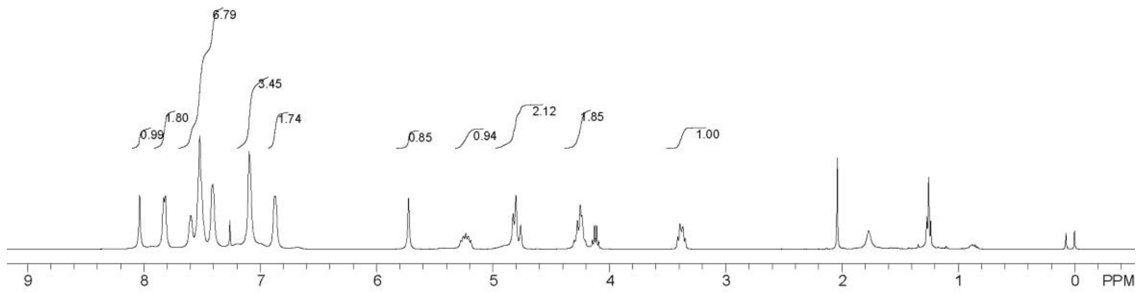
8.037
7.815
7.520
7.408
7.095
6.877
5.725
5.213
4.826
4.803
4.275
4.249
3.393
3.367
0.000
0.99
1.80
6.79
3.45
1.74
0.85
0.94
2.12
1.85
1.00
9
8
7
6
5
4
3
2
1
0
PPM

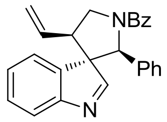

8mB

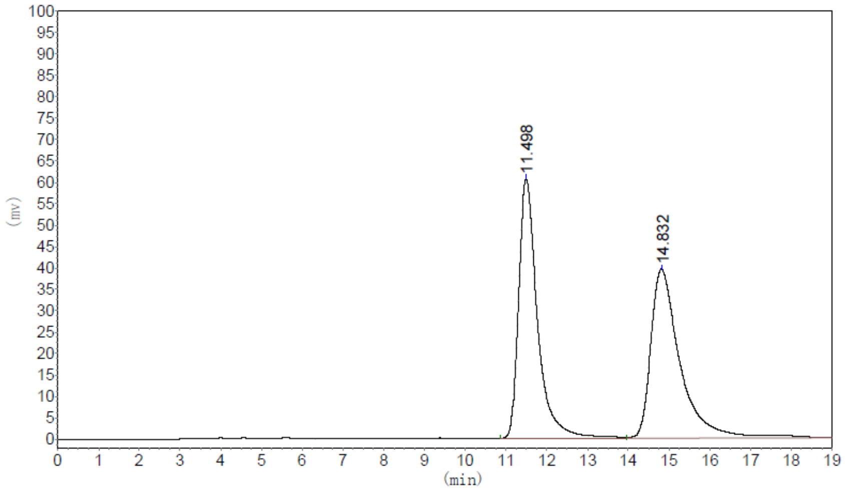

Peak No.	R. Time	Peak Height	Peak Area	Percent
1	11. 498	60363. 332	1939185. 250	49. 9968
2	14. 832	39475. 930	1939435. 250	50. 0032
Total		99839. 262	3878620. 500	100. 0000

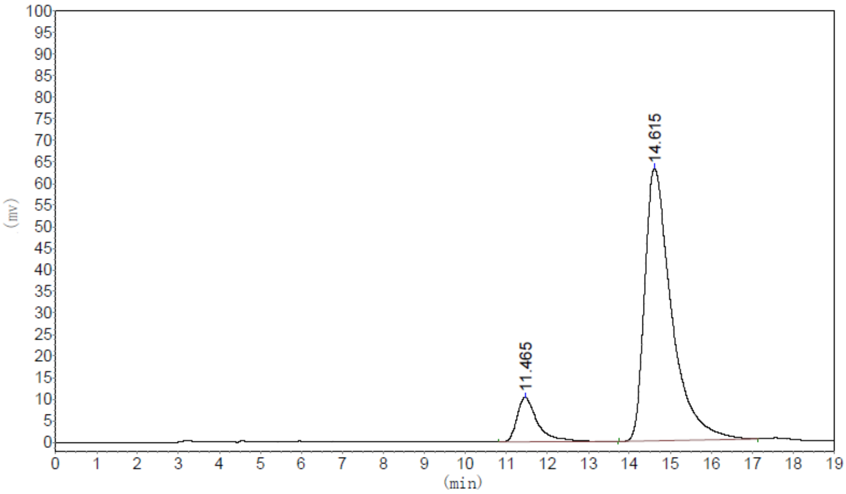

Peak No.	R. Time	Peak Height	Peak Area	Percent
1	11. 465	10396. 948	367165. 313	11. 0881
2	14. 615	63125. 441	2944166. 750	88. 9119
Total		73522. 390	3311332. 063	100. 0000

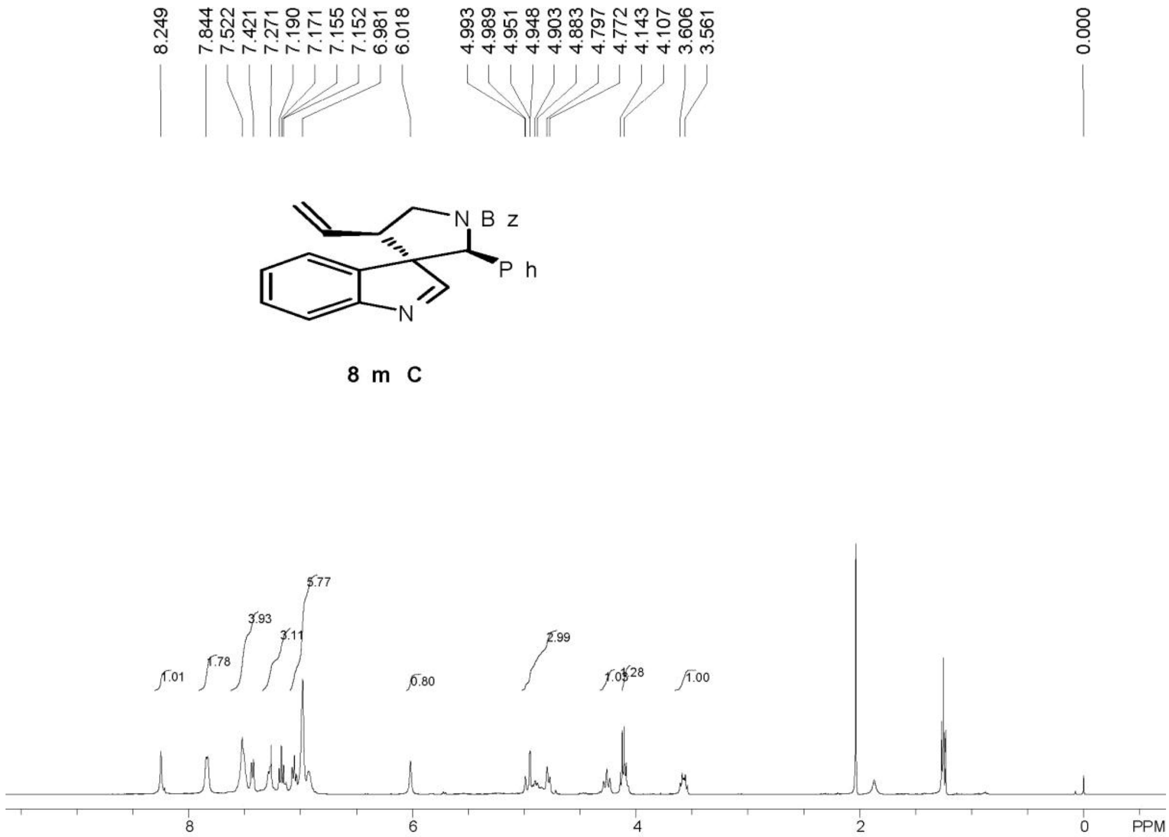
8.249
7.844
7.522
7.421
7.271
7.190
7.171
7.155
7.152
6.981
6.018
4.993
4.989
4.951
4.948
4.903
4.883
4.797
4.772
4.143
4.107
3.606
3.561
0.000
N Bz
Ph
N
8mC
1.01
1.78
3.93
3.11
5.77
0.80
2.99
1.05
1.28
1.00
8
6
4
2
0
PPM

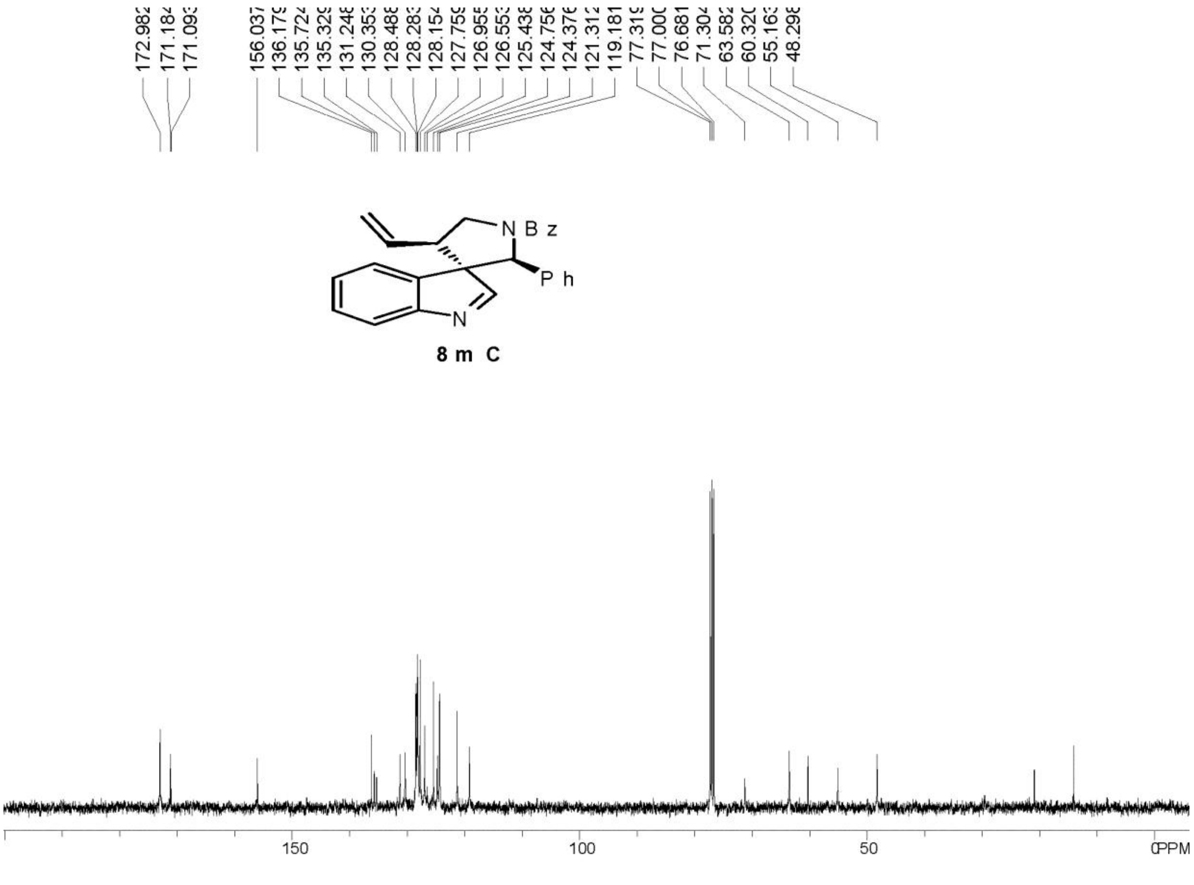
N Bz
Ph
N
8mC
150
100
50
0
PPM

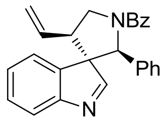

8mC

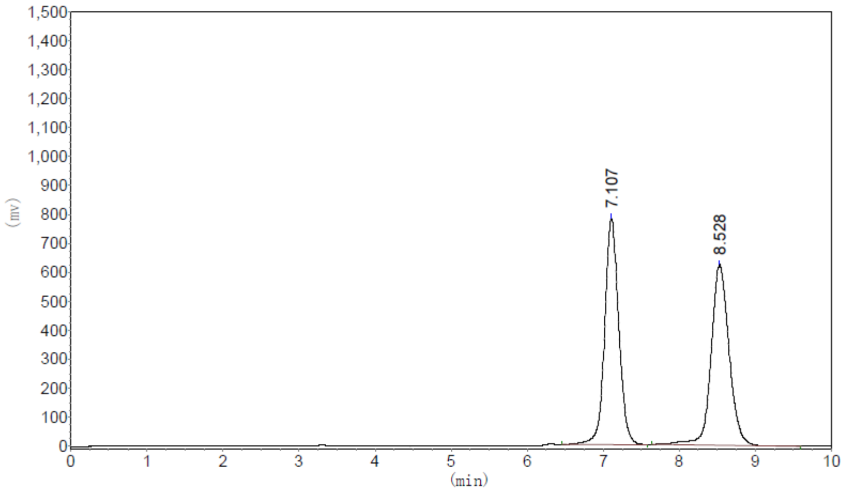

Peak No.	R. Time	Peak Height	Peak Area	Percent
1	7. 107	782312. 250	10088292. 000	49. 7134
2	8. 528	623578. 563	10204592. 000	50. 2866
Total		1405890. 813	20292884. 000	100. 0000

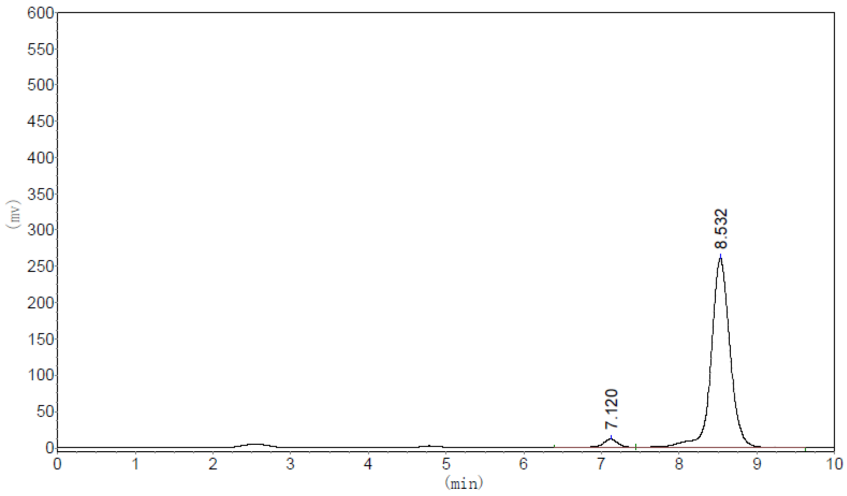

Peak No.	R. Time	Peak Height	Peak Area	Percent
1	7. 120	11586. 212	174044. 734	3. 7883
2	8. 532	260676. 781	4420179. 000	96. 2117
Total		272262. 993	4594223. 734	100. 0000

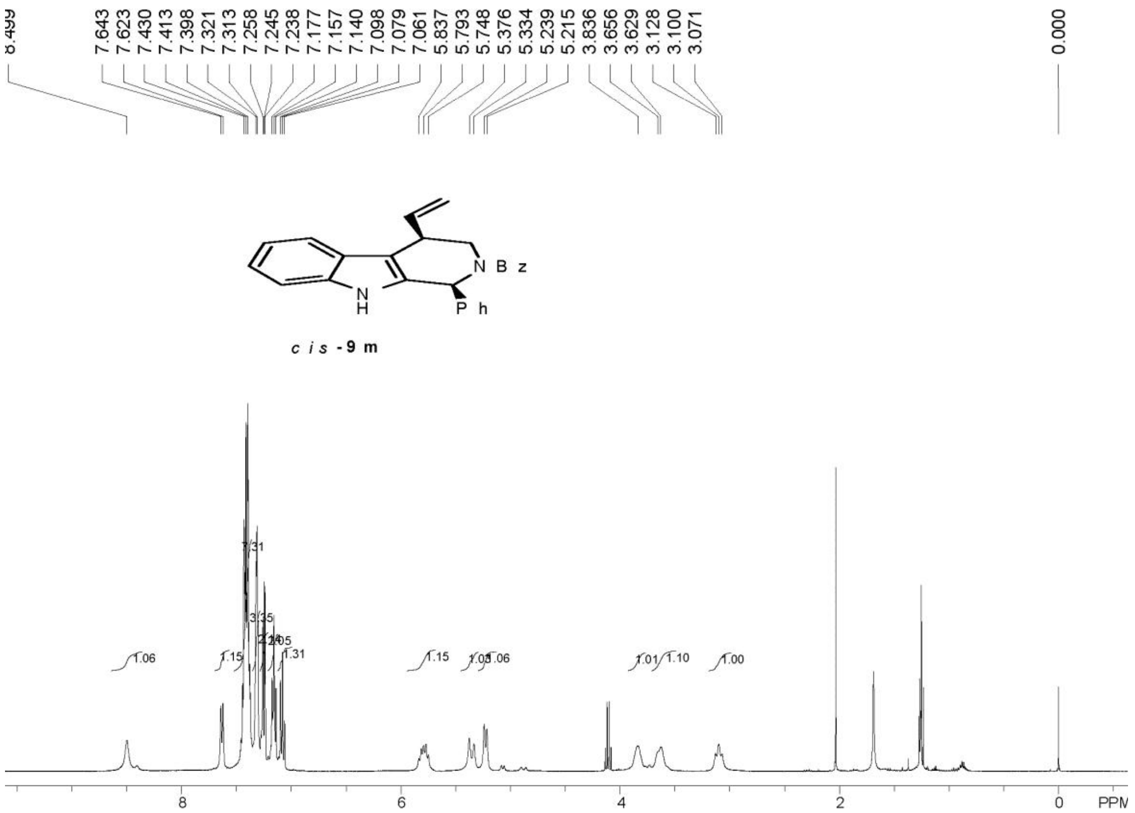
8.499
7.643
7.623
7.430
7.413
7.398
7.321
7.313
7.258
7.245
7.238
7.177
7.157
7.140
7.098
7.079
7.061
5.837
5.793
5.748
5.376
5.334
5.239
5.215
3.836
3.656
3.629
3.128
3.100
3.071
0.000
N Bz
N
H
Ph
cis-9m
1.06
1.15
7.31
3.35
2.14
2.05
1.31
1.15
1.03
1.06
1.01
1.10
1.00
8
6
4
2
0
PPM

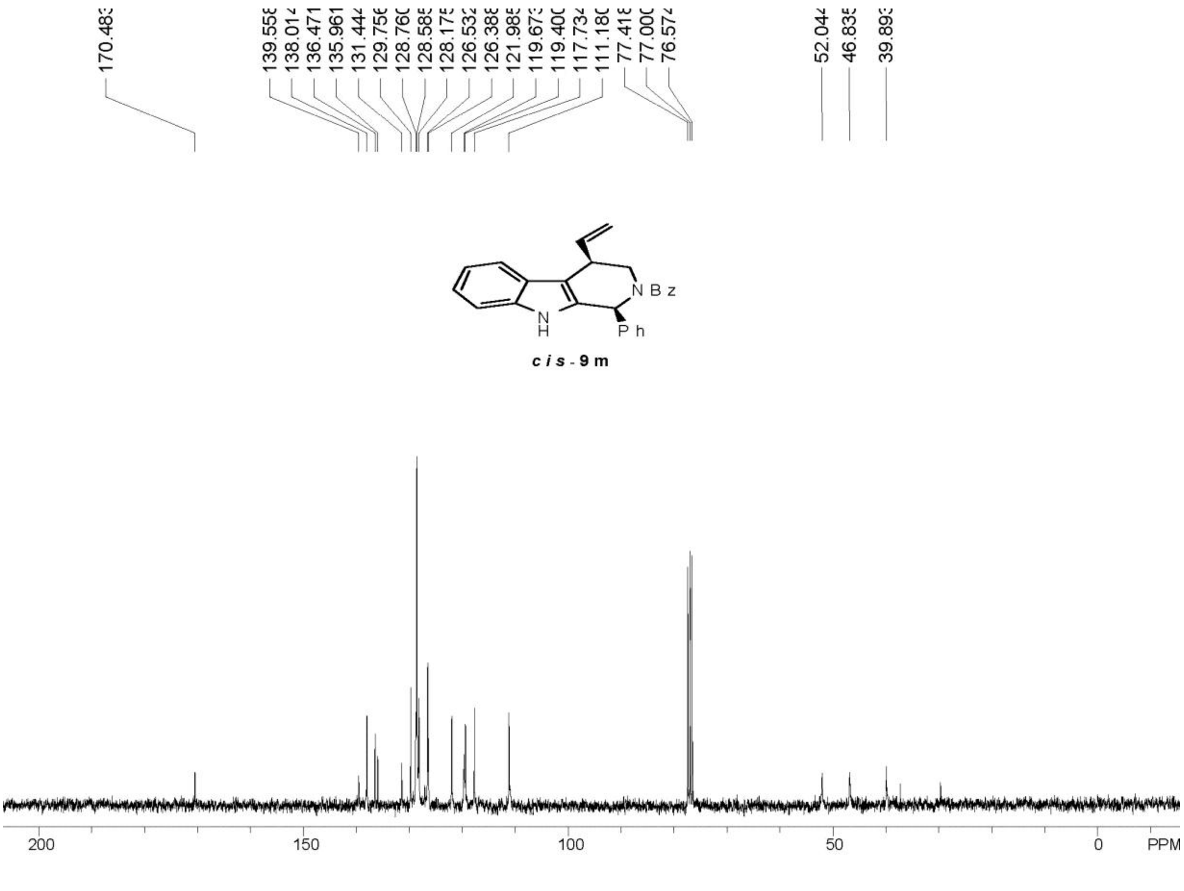
170.483
139.558
138.014
136.471
135.961
131.442
129.756
128.760
128.585
128.175
126.532
126.388
121.985
119.673
119.400
117.734
111.180
77.418
77.000
76.574
52.044
46.835
39.893
N Bz
N
H
Ph
cis-9m
200
150
100
50
0
PPM

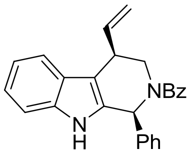

***cis*-9m**

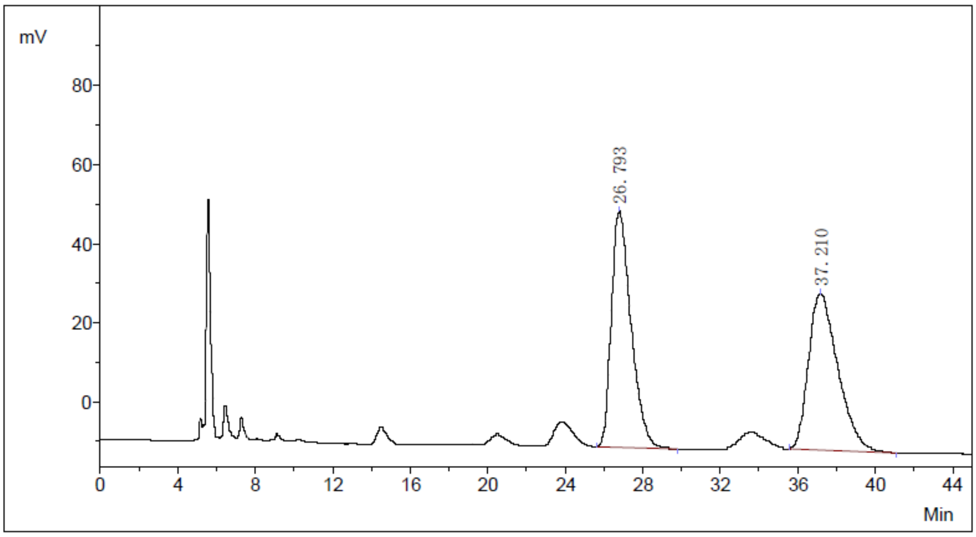

No.	PeakNo	ID. Name	R. Time	PeakHeight	PeakArea	PerCent
1	1	Unknown	26.793	59836.0	4207096.9	50.2602
2	2	Unknown	37.210	39581.9	4163539.3	49.7398
Total				99417.9	8370636.2	100.0000

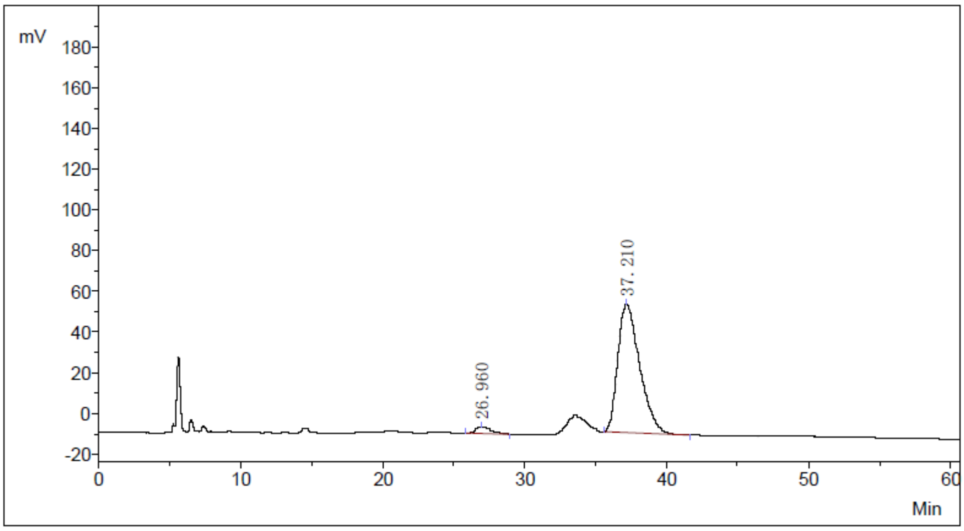

No.	PeakNo	ID. Name	R. Time	PeakHeight	PeakArea	PerCent
1	1	Unknown	26.960	3437.5	245284.0	3.4847
2	2	Unknown	37.210	63126.5	6793676.4	96.5153
Total				66564.0	7038960.4	100.0000

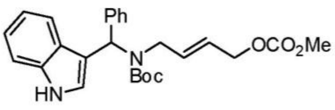
Ph
N
Boc
HN
OCO2Me

7n

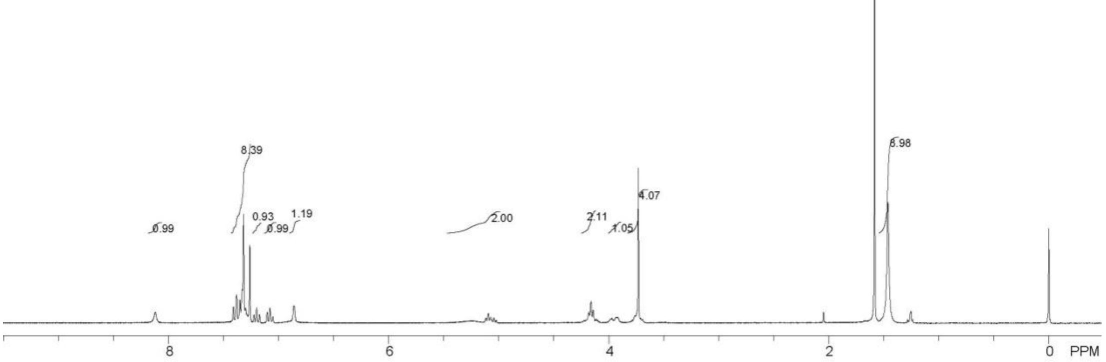
8.122
7.411
7.383
7.320
7.301
7.225
7.200
7.174
7.104
7.079
7.056
6.861
5.243
5.114
5.095
5.074
5.045
5.024
4.222
4.181
4.162
4.142
4.111
3.975
3.933
3.787
3.760
3.731
3.710
3.696
1.462
-0.000
0.99
8.39
0.93
0.99
1.19
2.00
2.11
1.05
4.07
8.98
8
6
4
2
0
PPM

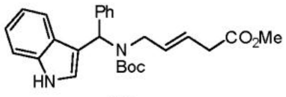
Ph
N
Boc
HN
CO2Me

7n

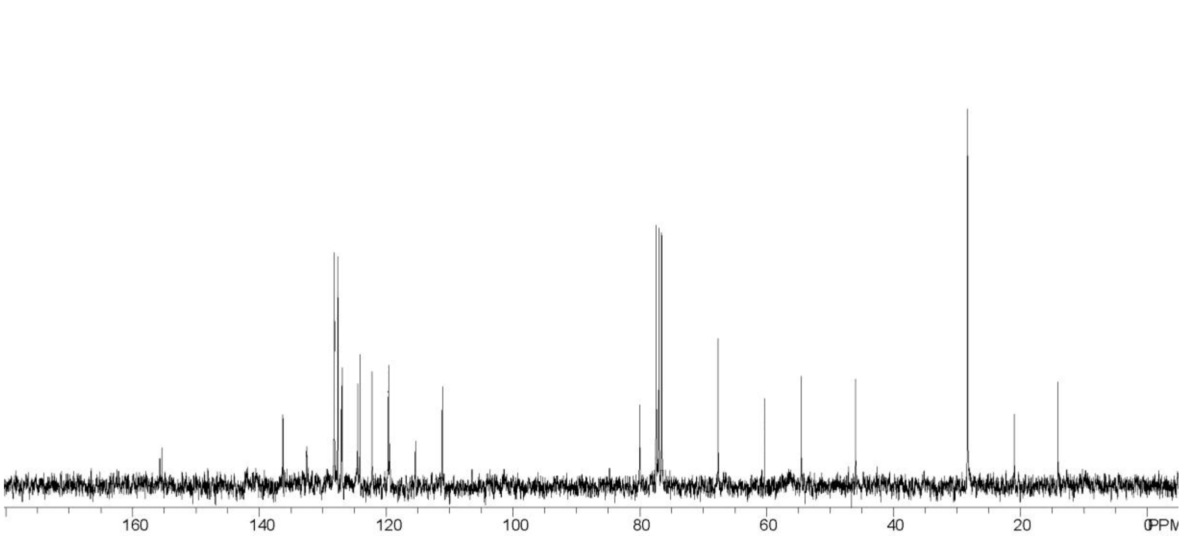
155.686
155.382
136.288
132.555
128.182
127.627
127.095
126.981
124.502
124.137
122.236
119.658
119.438
115.400
111.165
80.034
77.426
77.000
76.582
67.670
54.584
46.006
28.357
160
140
120
100
80
60
40
20
0
PPM

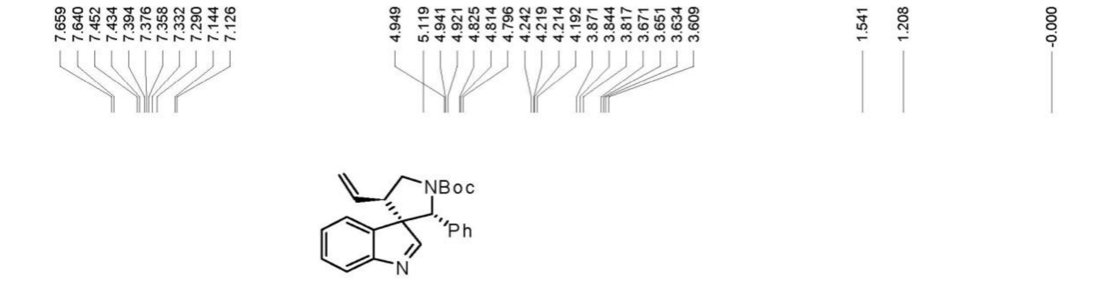
7.659
7.640
7.452
7.434
7.394
7.376
7.358
7.332
7.290
7.144
7.126
4.949
5.119
4.941
4.921
4.825
4.814
4.796
4.242
4.219
4.214
4.192
3.871
3.844
3.817
3.671
3.651
3.634
3.609
1.541
1.208
-0.000
NBoc
Ph
N

8nA

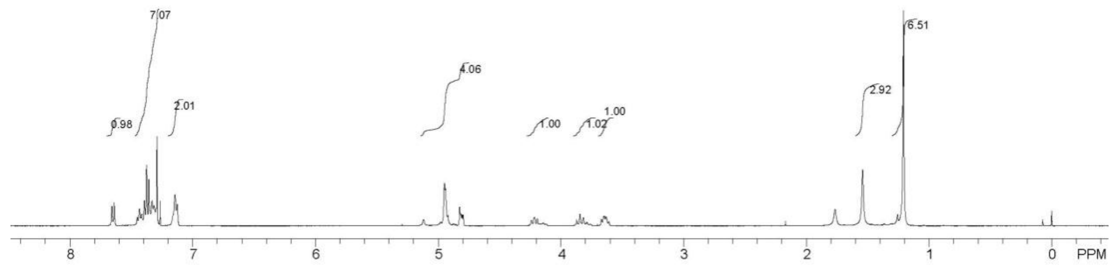
7.07
0.98
2.01
4.06
1.00
1.02
1.00
2.92
6.51
8
7
6
5
4
3
2
1
0
PPM

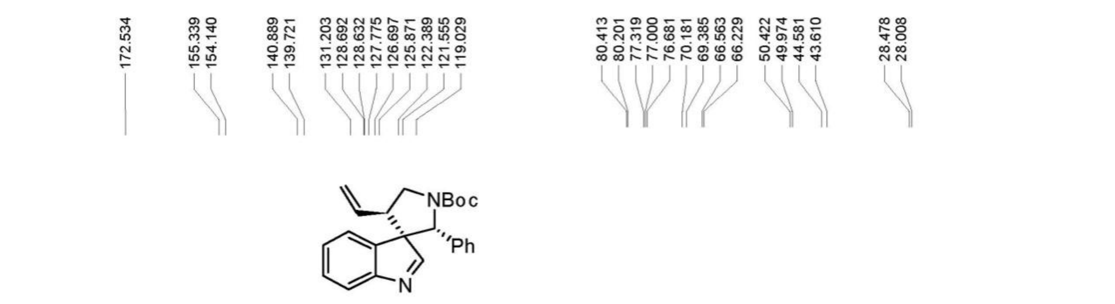
172.534
155.339
154.140
140.889
139.721
131.203
128.692
128.632
127.775
126.697
125.871
122.389
121.555
119.029
80.413
80.201
77.319
77.000
76.681
70.181
69.385
66.563
66.229
50.422
49.974
44.581
43.610
28.478
28.008
NBoc
Ph
N

8nA

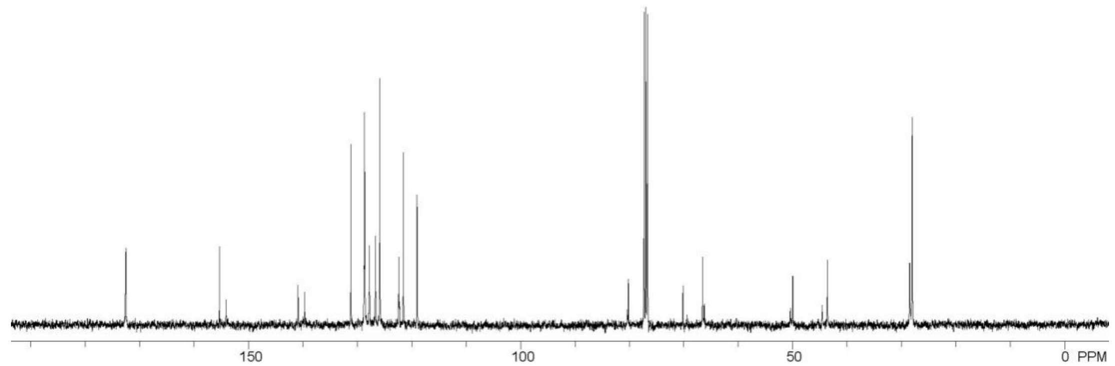
150
100
50
0
PPM

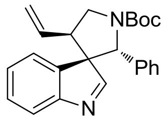

8nA

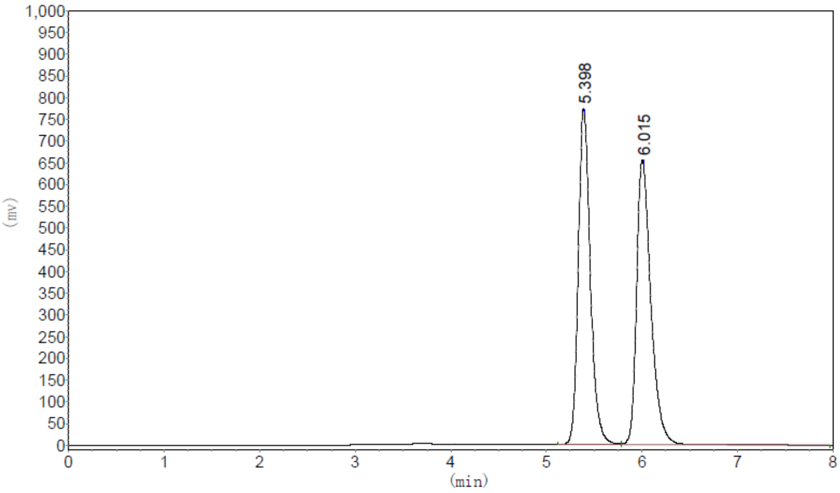

Peak No.	R. Time	Peak Height	Peak Area	Percent
1	5.398	771074.125	6798884.000	49.9517
2	6.015	652579.938	6812029.000	50.0483
Total		1423654.063	13610913.000	100.0000

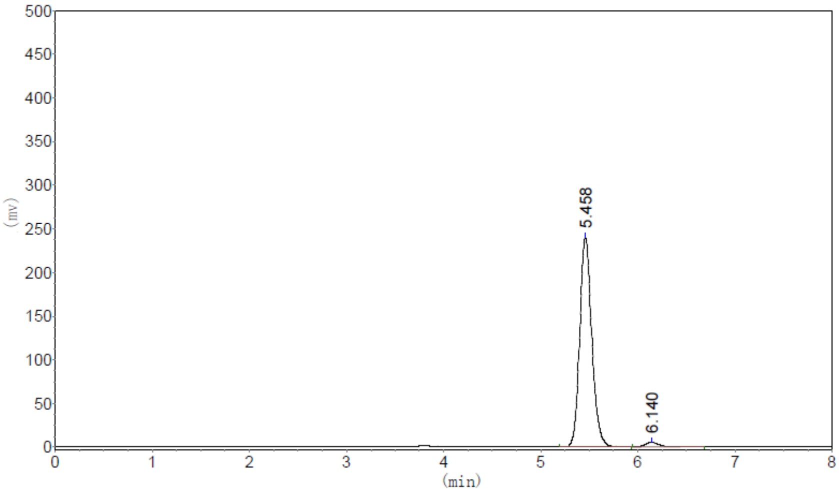

Peak No.	R. Time	Peak Height	Peak Area	Percent
1	5.458	239923.844	2081150.750	97.3714
2	6.140	5207.743	56182.129	2.6286
Total		245131.587	2137332.879	100.0000

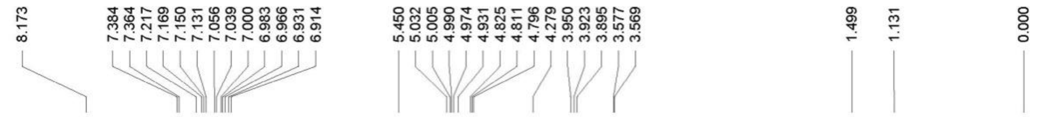
8.173
7.384
7.364
7.217
7.169
7.150
7.131
7.056
7.039
7.000
6.983
6.966
6.931
6.914
5.450
5.032
5.005
4.990
4.974
4.931
4.825
4.811
4.796
4.279
3.950
3.923
3.895
3.577
3.569
1.499
1.131
0.000

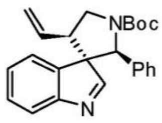
NBoc
Ph
N

8nC

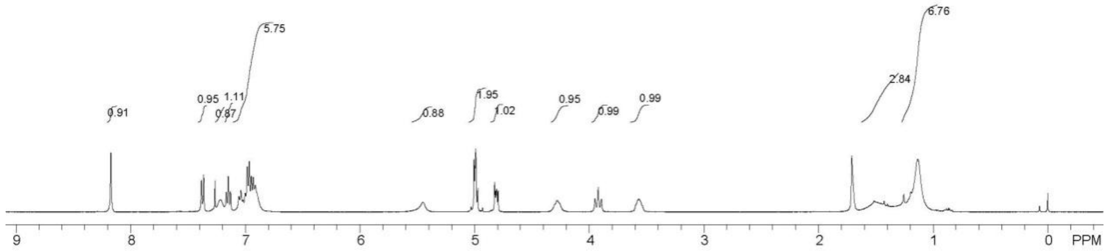
0.91
0.95
0.87
1.11
5.75
0.88
1.95
1.02
0.95
0.99
0.99
2.84
6.76
9
8
7
6
5
4
3
2
1
0
PPM

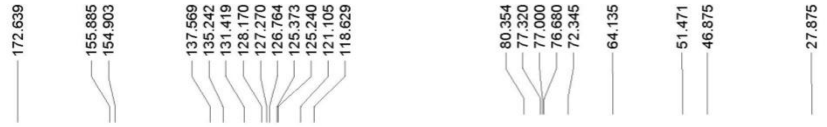
172.639
155.885
154.903
137.569
135.242
131.419
128.170
127.270
126.764
125.373
125.240
121.105
118.629
80.354
77.320
77.000
76.680
72.345
64.135
51.471
46.875
27.875

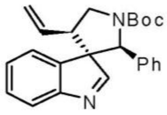
NBoc
Ph
N

8nC

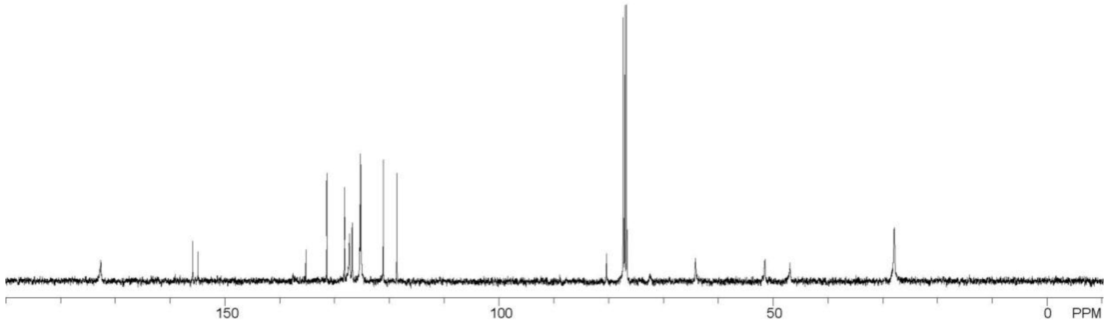
150
100
50
0
PPM

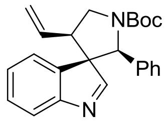

8nC

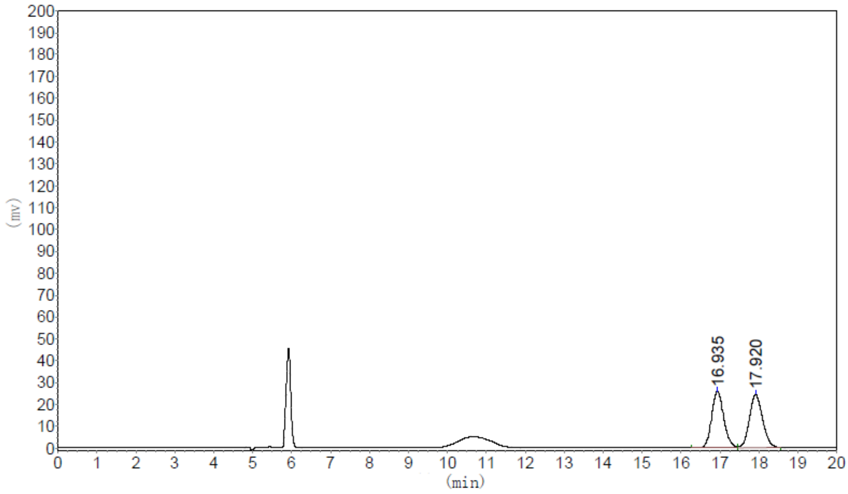

Peak No.	R. Time	Peak Height	Peak Area	Percent
1	16. 935	25836. 219	565400. 125	49. 5601
2	17. 920	24545. 121	575436. 688	50. 4399
Total		50381. 340	1140836. 813	100. 0000

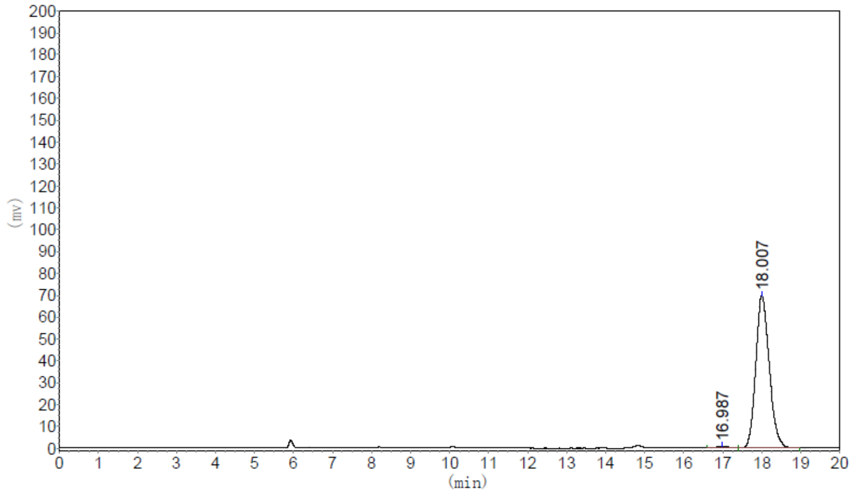

Peak No.	R. Time	Peak Height	Peak Area	Percent
1	16. 987	1063. 063	22093. 344	1. 2986
2	18. 007	69664. 531	1679247. 750	98. 7014
Total		70727. 594	1701341. 094	100. 0000

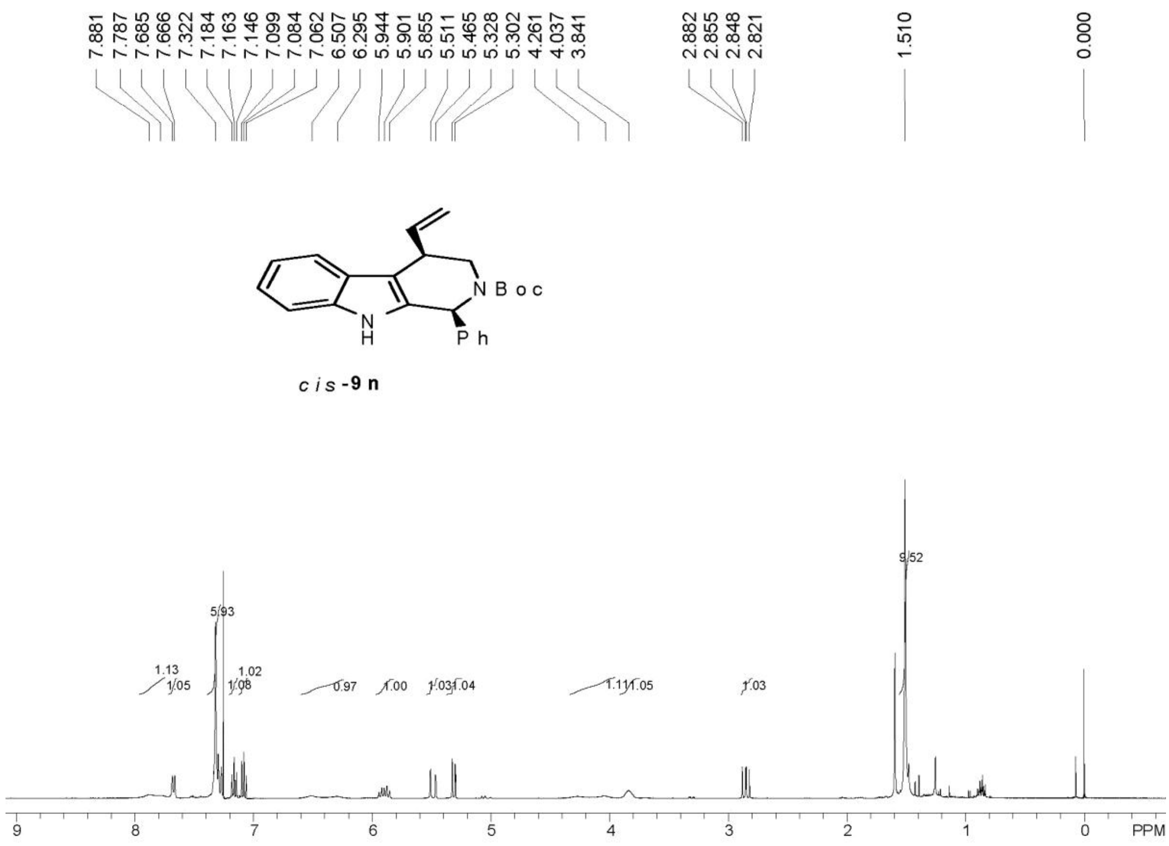
7.881 7.787 7.685 7.666 7.322 7.184 7.163 7.146 7.099 7.084 7.062 6.507 6.295 5.944 5.901 5.855 5.511 5.465 5.328 5.302 4.261 4.037 3.841
2.882 2.855 2.848 2.821
1.510
0.000
N Boc
N H
Ph
cis-9n
1.13 1.05 5.93 1.02 1.08 0.97 1.00 1.03 1.04 1.11 1.05 1.03 9.52
9 8 7 6 5 4 3 2 1 0 PPM

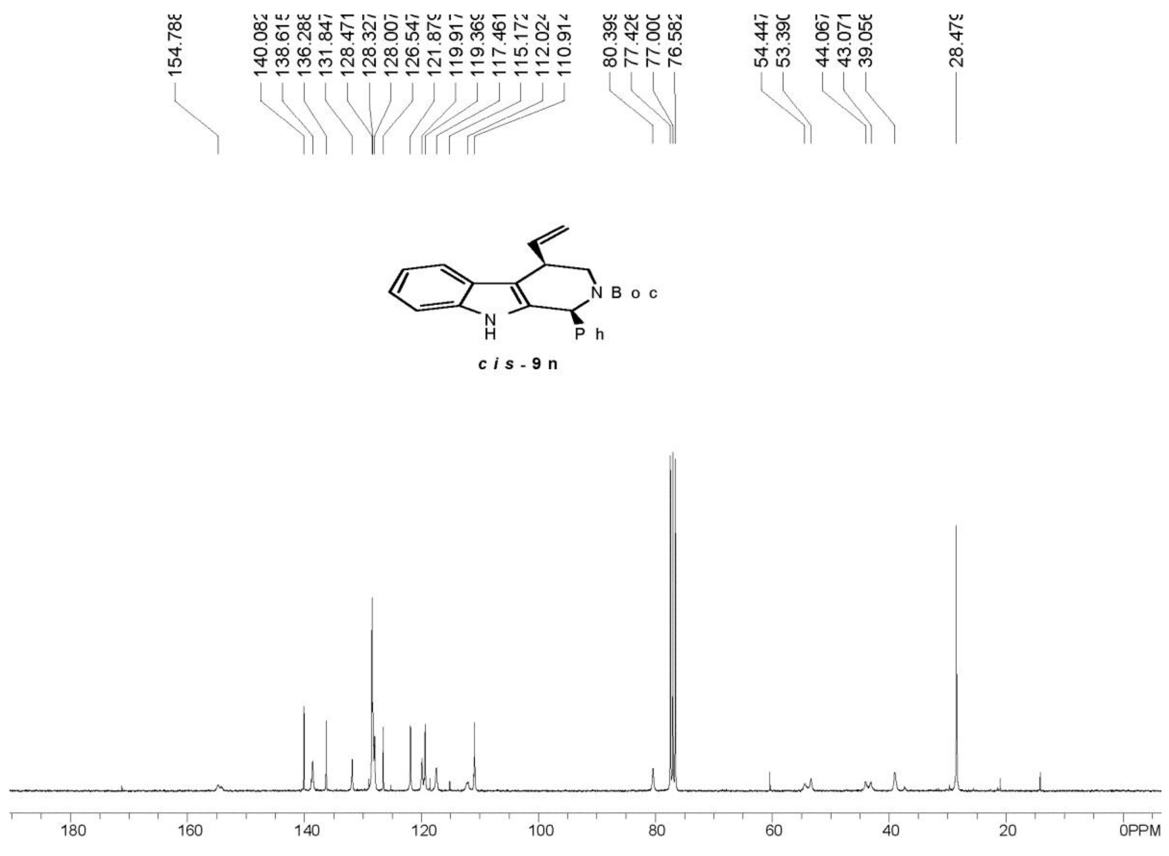
154.788
140.082 138.615 136.288 131.847 128.471 128.327 128.007 126.547 121.879 119.917 119.369 117.461 115.172 112.024 110.914
80.399 77.426 77.000 76.582
54.447 53.390 44.067 43.071 39.056
28.479
N Boc
N H
Ph
cis-9n
180 160 140 120 100 80 60 40 20 0PPM

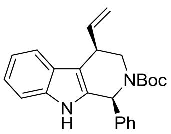

***cis*-9n**

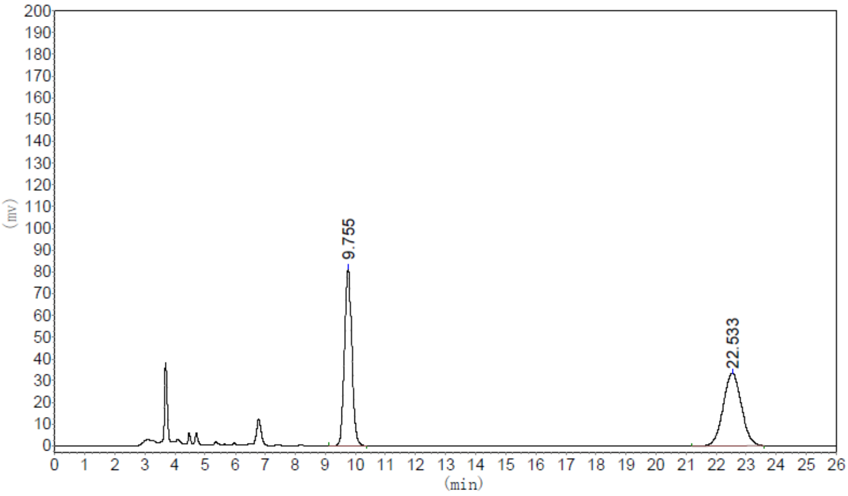

Peak No.	R. Time	Peak Height	Peak Area	Percent
1	9.755	80969.938	1371135.750	49.4939
2	22.533	33358.141	1399179.250	50.5061
Total		114328.078	2770315.000	100.0000

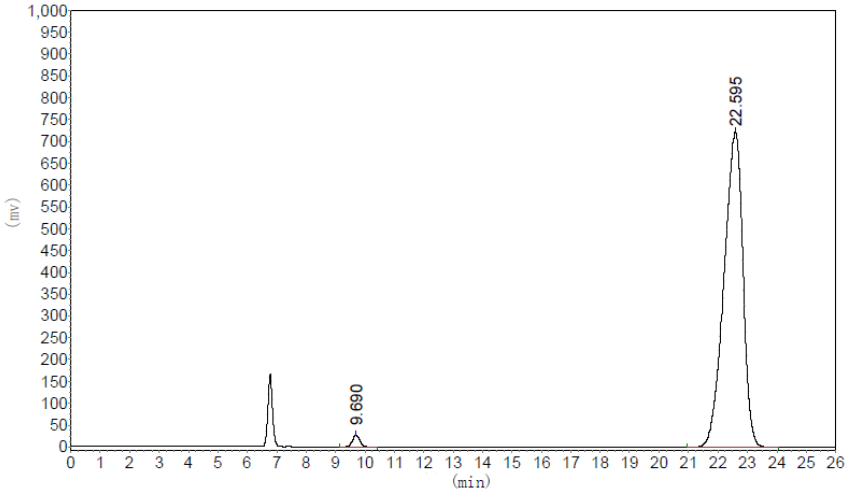

Peak No.	R. Time	Peak Height	Peak Area	Percent
1	9.690	27181.430	454634.156	1.3878
2	22.595	722963.438	32305676.000	98.6122
Total		750144.867	32760310.156	100.0000

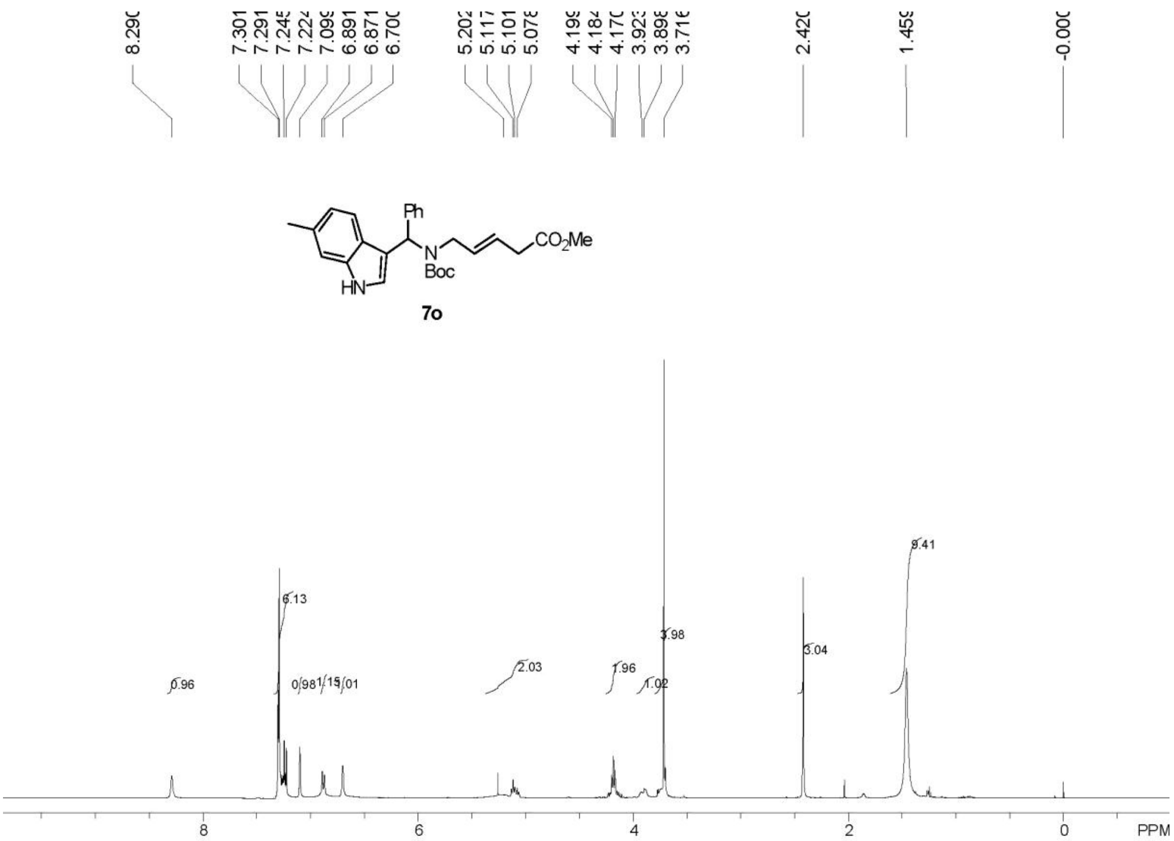
8.290
7.301
7.291
7.245
7.224
7.099
6.891
6.871
6.700
5.202
5.117
5.101
5.078
4.199
4.184
4.170
3.923
3.898
3.716
2.420
1.459
-0.000
0.96
6.13
0.98
1.15
1.01
2.03
1.96
1.02
3.98
3.04
9.41
8
6
4
2
0
PPM

7o

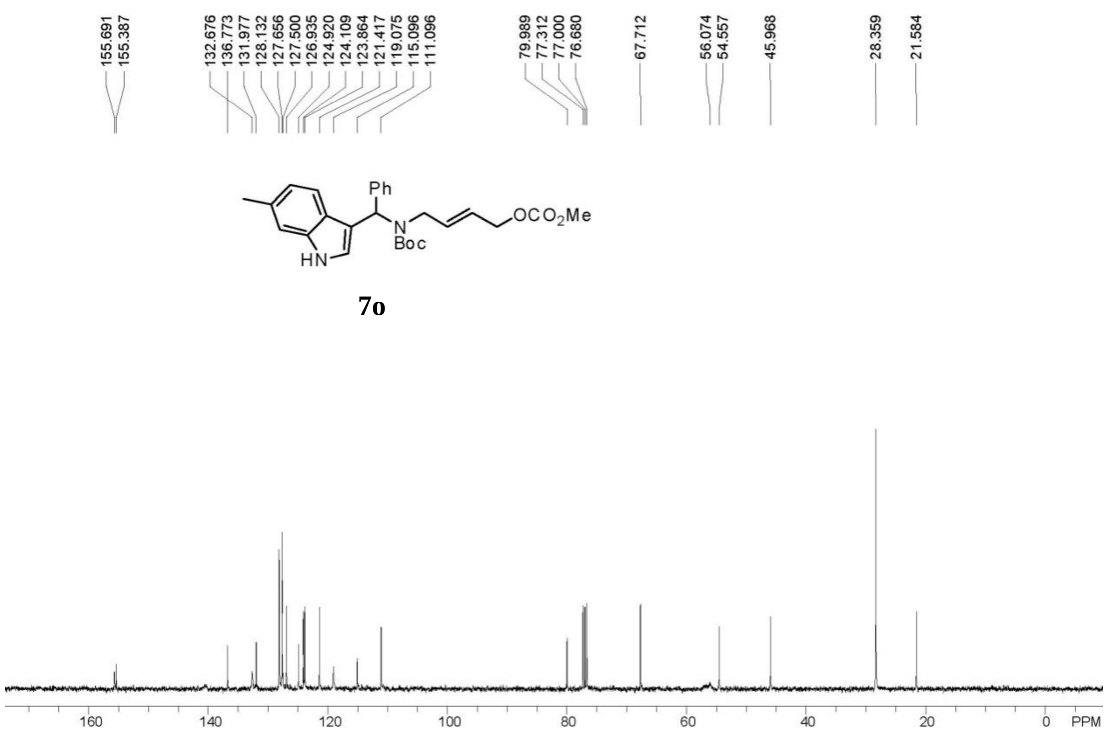
155.691
155.387
132.676
136.773
131.977
128.132
127.656
127.500
126.935
124.920
124.109
123.864
121.417
119.075
115.096
111.096
79.989
77.312
77.000
76.680
67.712
56.074
54.557
45.968
28.359
21.584
160
140
120
100
80
60
40
20
0
PPM

7o

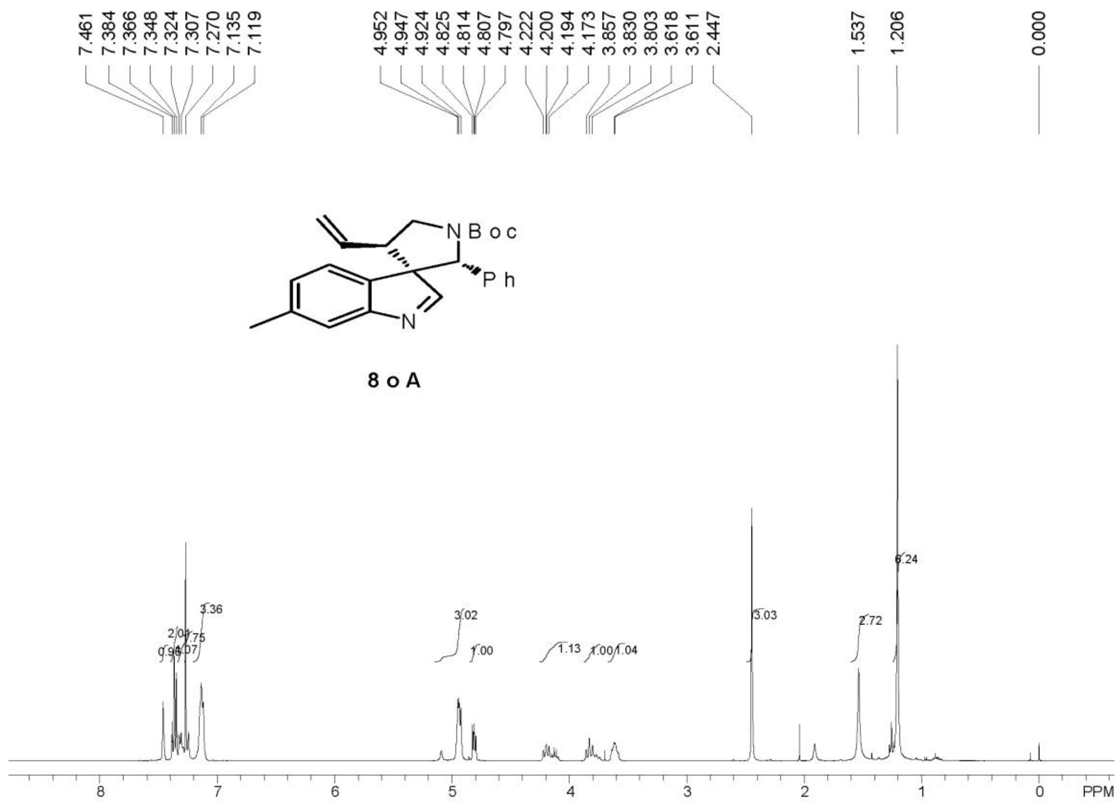
7.461
7.384
7.366
7.348
7.324
7.307
7.270
7.135
7.119
4.952
4.947
4.924
4.825
4.814
4.807
4.797
4.222
4.200
4.194
4.173
3.857
3.830
3.803
3.618
3.611
2.447
1.537
1.206
0.000
NBoc
Ph
N
PPM

8oA

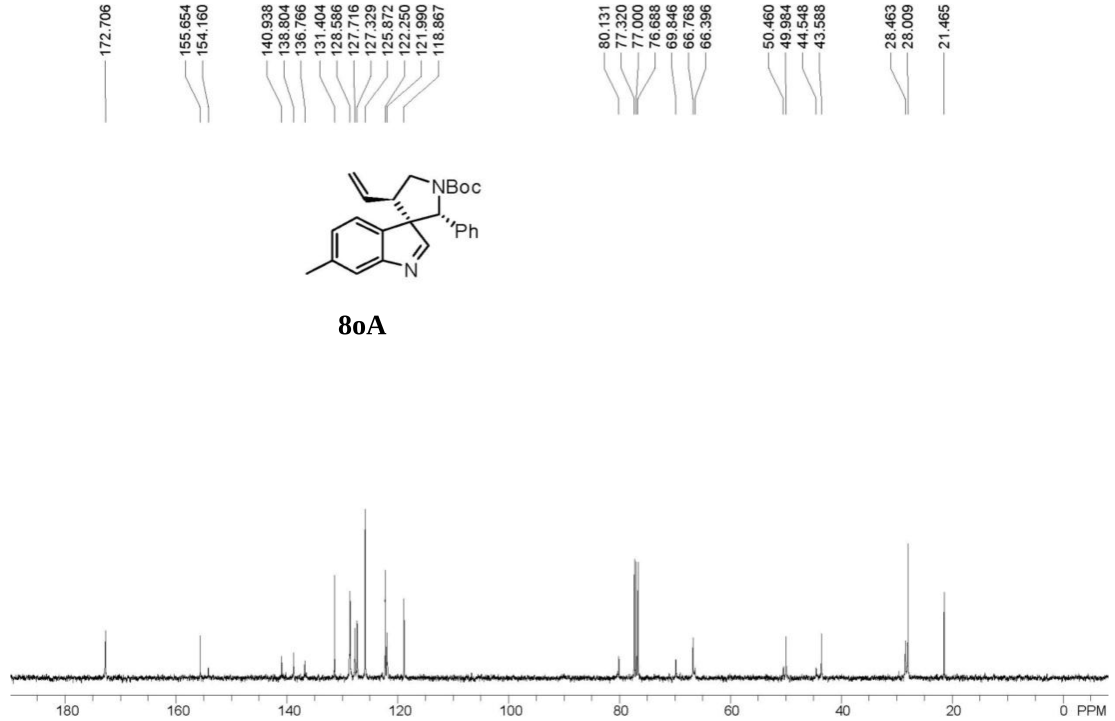
172.706
155.654
154.160
140.938
138.804
136.766
131.404
128.586
127.716
127.329
125.872
122.250
121.990
118.867
80.131
77.320
77.000
76.688
69.846
66.768
66.396
50.460
49.984
44.548
43.588
28.463
28.009
21.465
NBoc
Ph
N
PPM

8oA

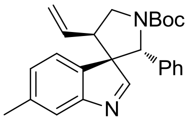

8oA

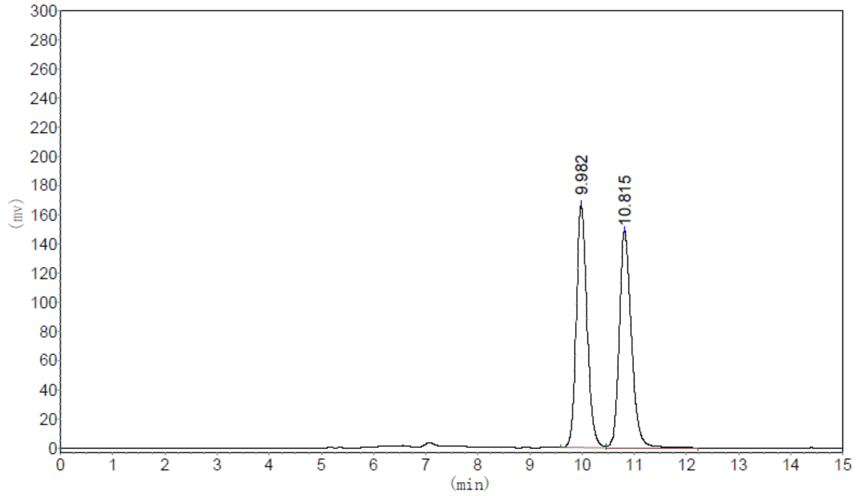

Peak No.	R. Time	Peak Height	Peak Area	Percent
1	9. 982	166007. 969	2372556. 000	49. 6709
2	10. 815	148490. 953	2403998. 000	50. 3291
Total		314498. 922	4776554. 000	100. 0000

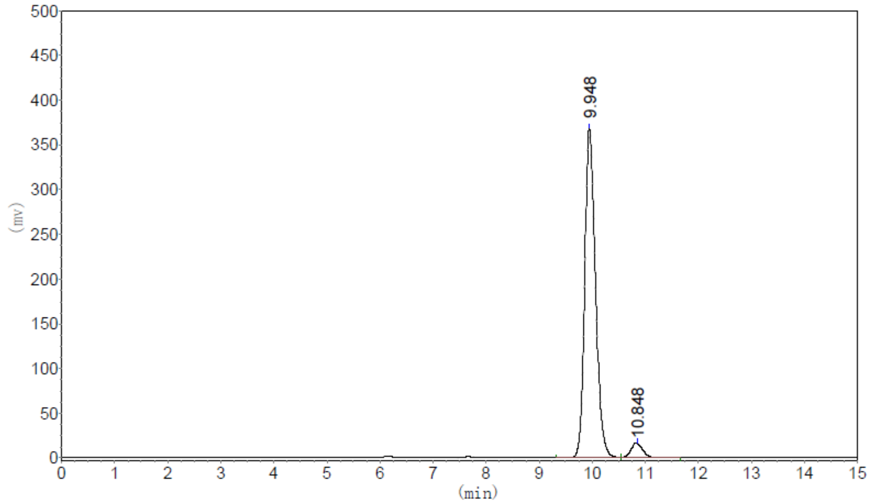

Peak No.	R. Time	Peak Height	Peak Area	Percent
1	9. 948	367604. 406	5214907. 500	95. 1020
2	10. 848	16795. 650	268582. 094	4. 8980
Total		384400. 057	5483489. 594	100. 0000

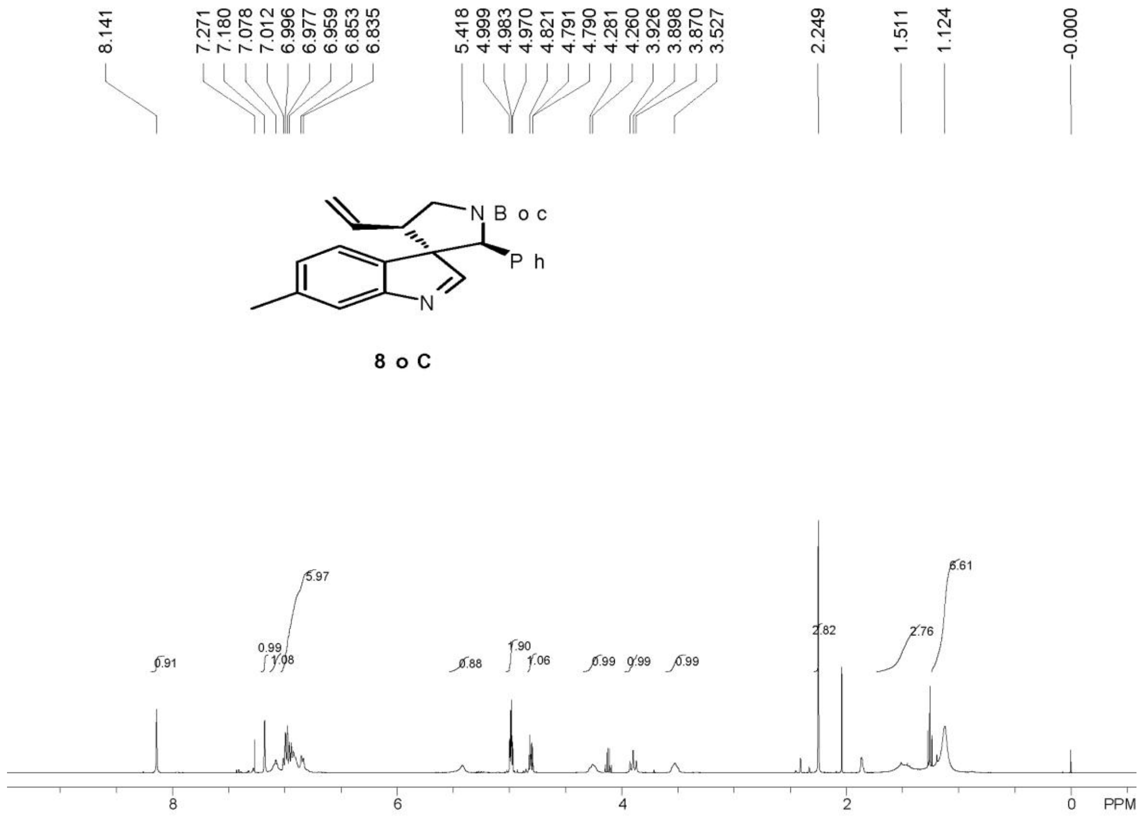
8.141
7.271
7.180
7.078
7.012
6.996
6.977
6.959
6.853
6.835
5.418
4.999
4.983
4.970
4.821
4.791
4.790
4.281
4.260
3.926
3.898
3.870
3.527
2.249
1.511
1.124
-0.000
NBoc
Ph
N
8oC
0.91
0.99
1.08
5.97
0.88
1.90
1.06
0.99
0.99
0.99
2.82
2.76
6.61
8
6
4
2
0
PPM

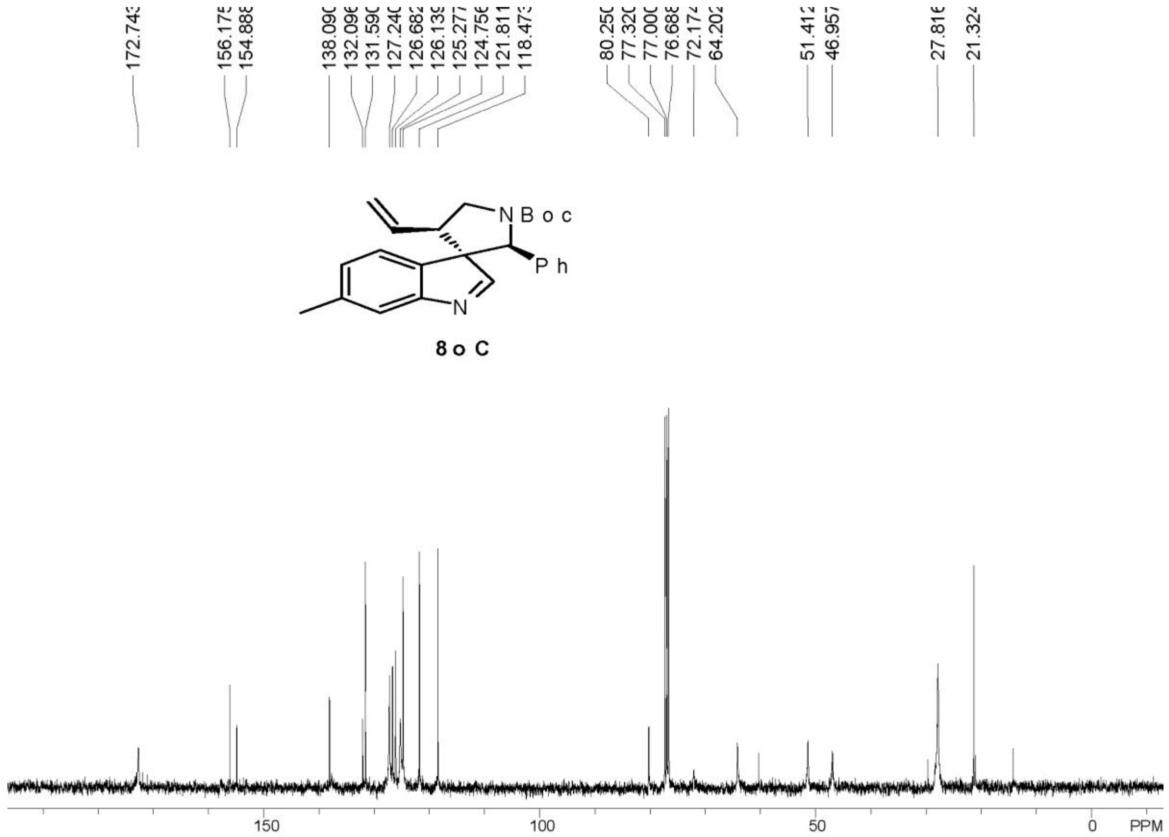
172.743
156.175
154.888
138.090
132.096
131.590
127.240
126.682
126.139
125.277
124.756
121.811
118.473
80.250
77.320
77.000
76.688
72.174
64.202
51.412
46.957
27.816
21.324
NBoc
Ph
N
8oC
150
100
50
0
PPM

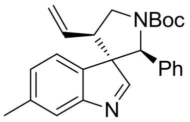

8oC

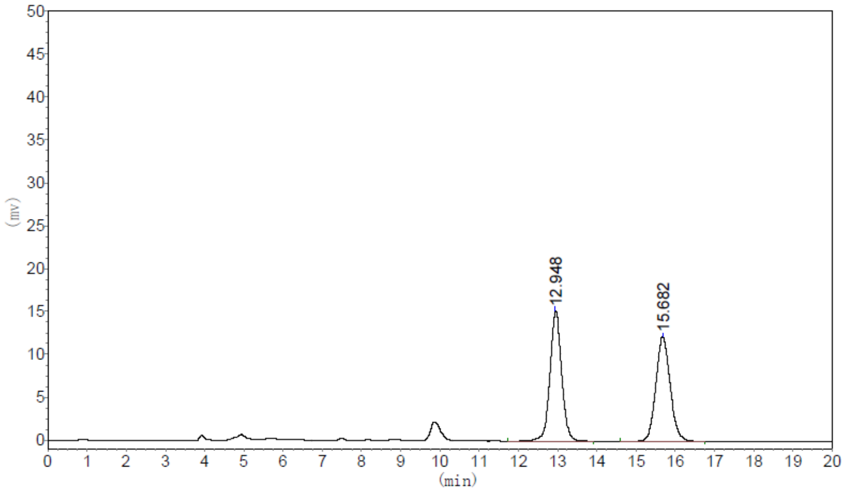

Peak No.	R. Time	Peak Height	Peak Area	Percent
1	12.948	15088.606	314379.813	50.3657
2	15.682	12201.031	309814.906	49.6343
Total		27289.638	624194.719	100.0000

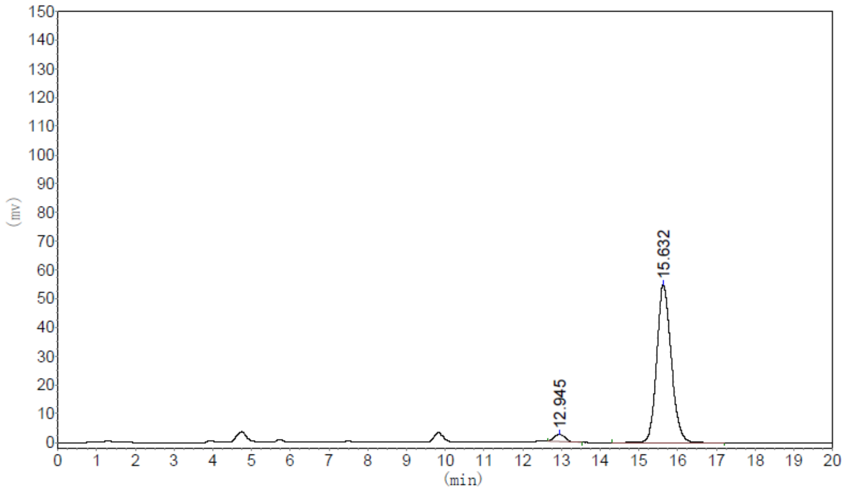

Peak No.	R. Time	Peak Height	Peak Area	Percent
1	12.945	2545.106	46810.098	3.1829
2	15.632	54930.598	1423856.000	96.8171
Total		57475.704	1470666.098	100.0000

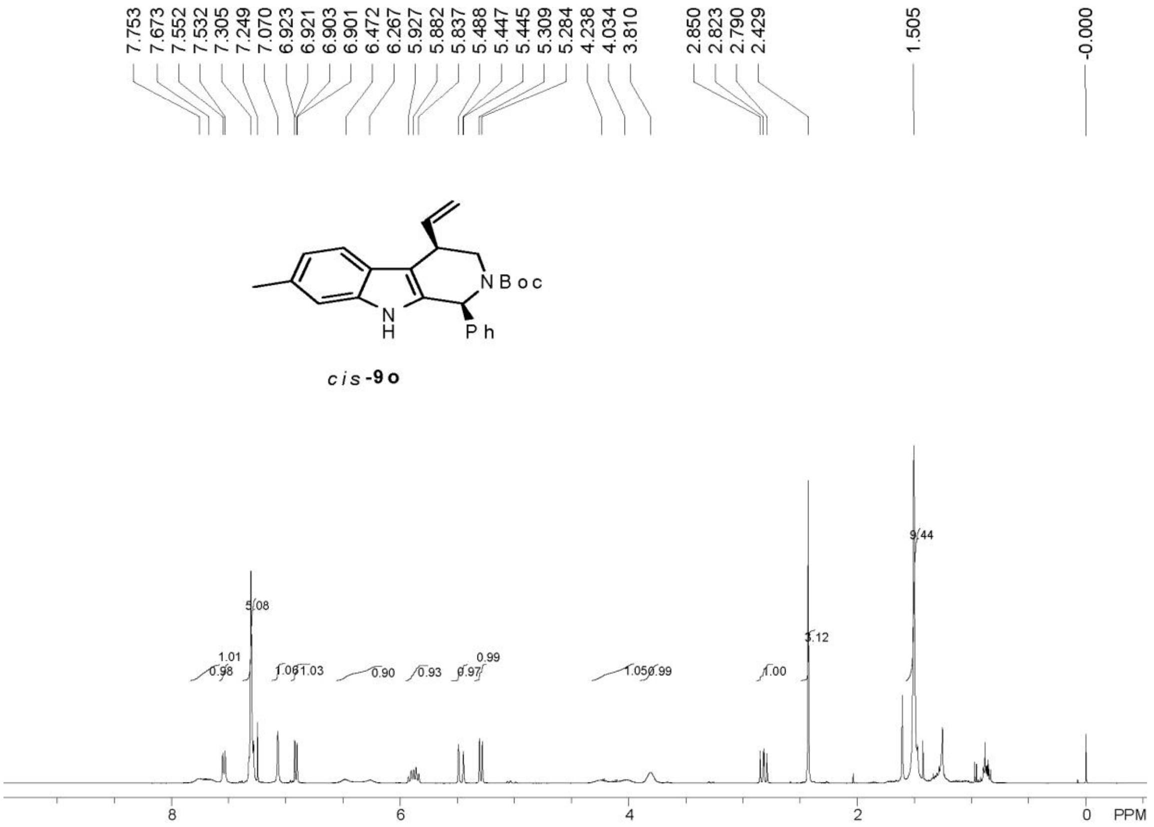
7.753 7.673 7.552 7.532 7.305 7.249 7.070 6.923 6.921 6.903 6.901 6.472 6.267 5.927 5.882 5.837 5.488 5.447 5.445 5.309 5.284 4.238 4.034 3.810 2.850 2.823 2.790 2.429 1.505 -0.000
NBoc
N
H
Ph
cis-9o
1.01 0.98 5.08 1.06 1.03 0.90 0.93 0.97 0.99 1.05 0.99 1.00 3.12 9.44
8 6 4 2 0 PPM

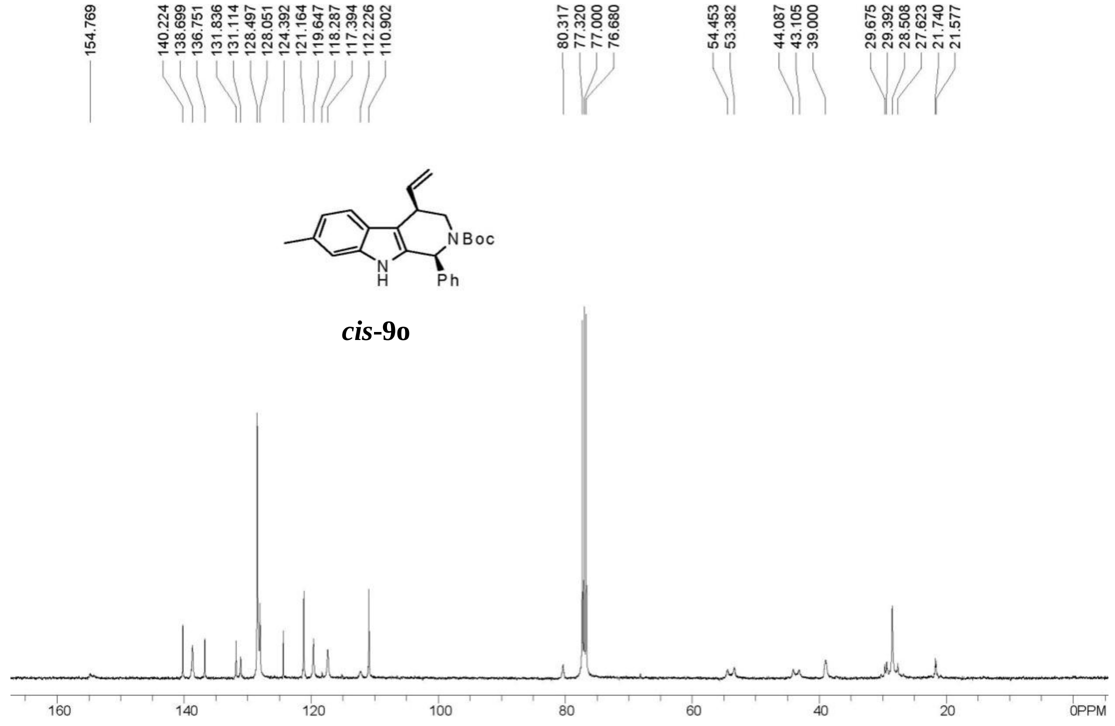
154.769 140.224 138.699 136.751 131.836 131.114 128.497 128.051 124.392 121.164 119.647 118.287 117.394 112.226 110.902 80.317 77.320 77.000 76.680 54.453 53.382 44.087 43.105 39.000 29.675 29.392 28.508 27.623 21.740 21.577
NBoc
N
H
Ph
cis-9o
160 140 120 100 80 60 40 20 0PPM

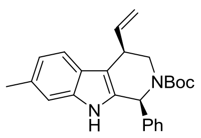

***cis*-9o**

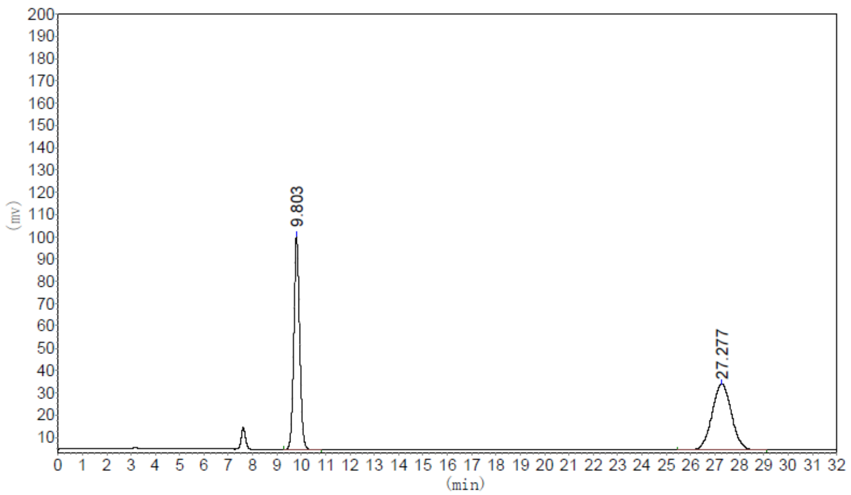

Peak No.	R. Time	Peak Height	Peak Area	Percent
1	9.803	95468.570	1617139.500	49.7585
2	27.277	29565.492	1632836.500	50.2415
Total		125034.063	3249976.000	100.0000

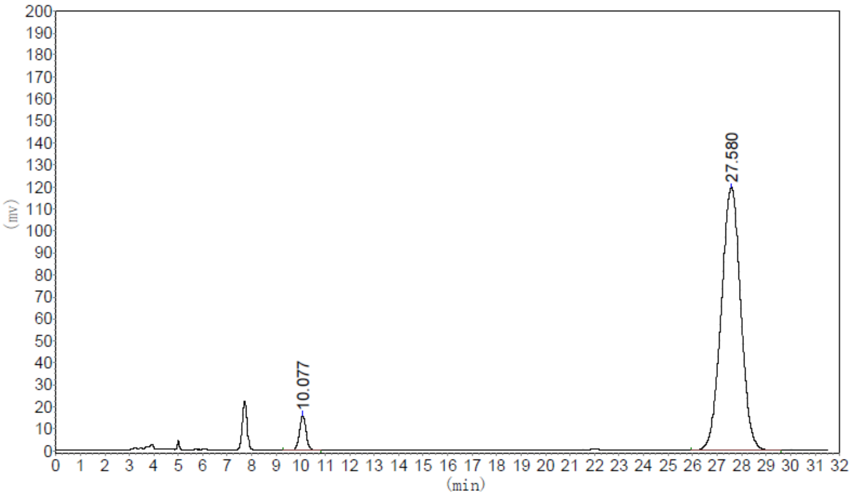

Peak No.	R. Time	Peak Height	Peak Area	Percent
1	10.077	15530.042	281440.000	4.1089
2	27.580	119232.758	6568115.500	95.8911
Total		134762.800	6849555.500	100.0000

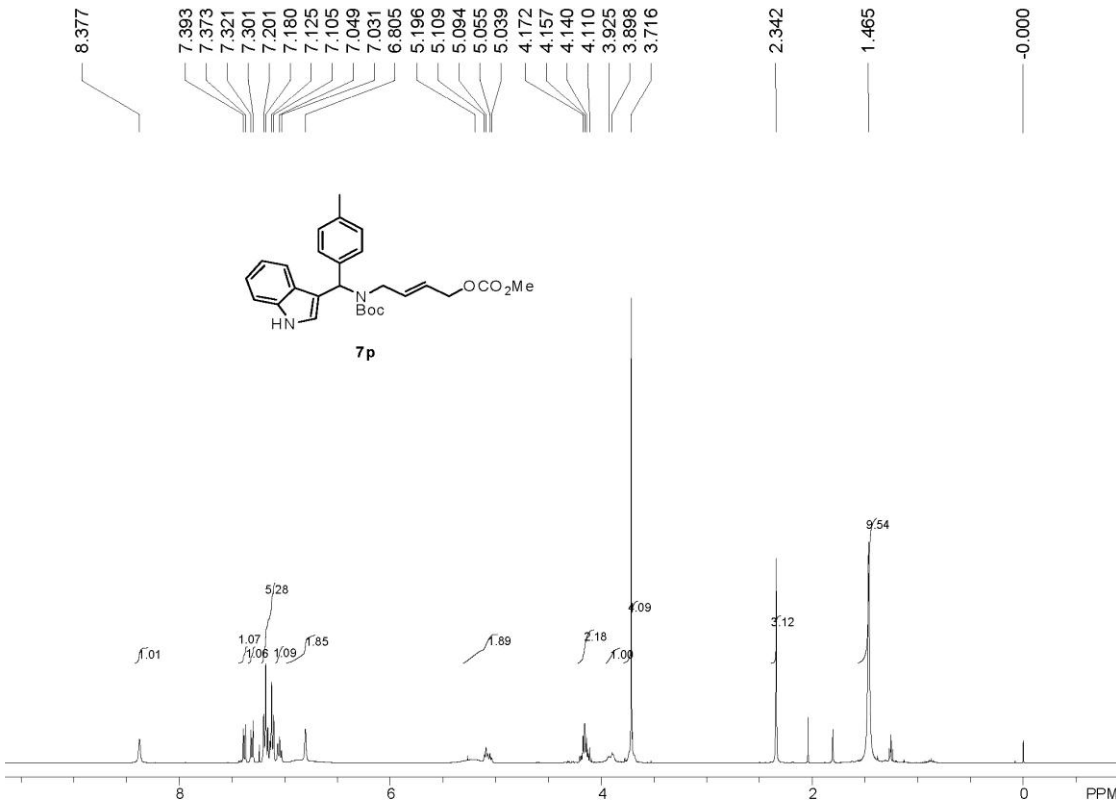
8.377
7.393 7.373 7.321 7.301 7.201 7.180 7.125 7.105 7.049 7.031 6.805 5.196 5.109 5.094 5.055 5.039 4.172 4.157 4.140 4.110 3.925 3.898 3.716
2.342
1.465
-0.000
OCO2Me
N
Boc
HN
7p
1.01
1.07
1.06
5.28
1.09
1.85
1.89
2.18
1.00
4.09
3.12
9.54
8
6
4
2
0
PPM

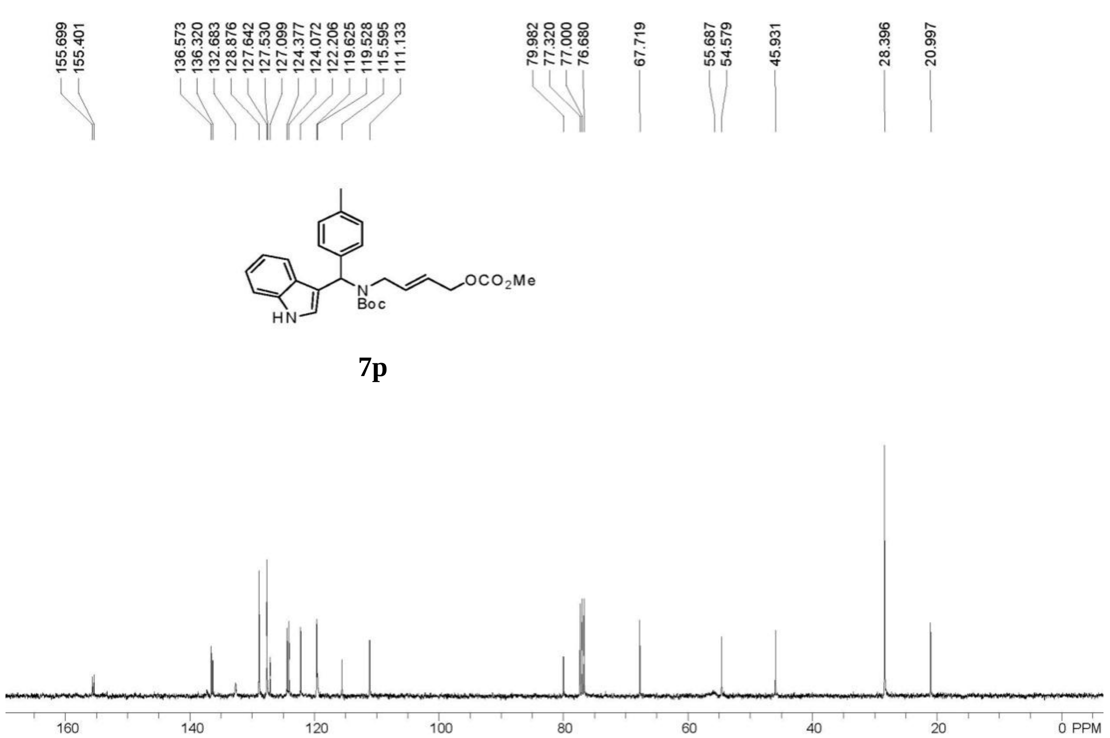
155.699 155.401
136.573 136.320 132.683 128.876 127.642 127.530 127.099 124.377 124.072 122.206 119.625 119.528 115.595 111.133
79.982 77.320 77.000 76.680
67.719
55.687 54.579
45.931
28.396
20.997
OCO2Me
N
Boc
HN
7p
160
140
120
100
80
60
40
20
0 PPM

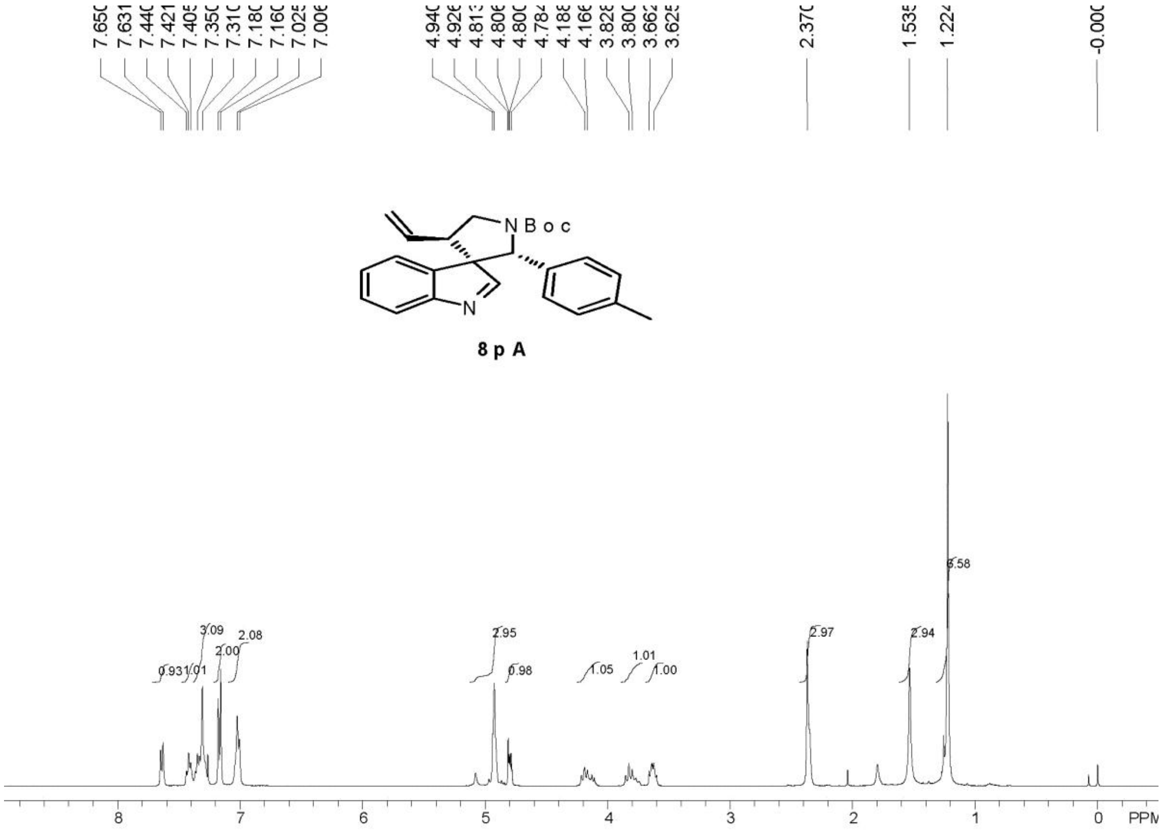

8pA

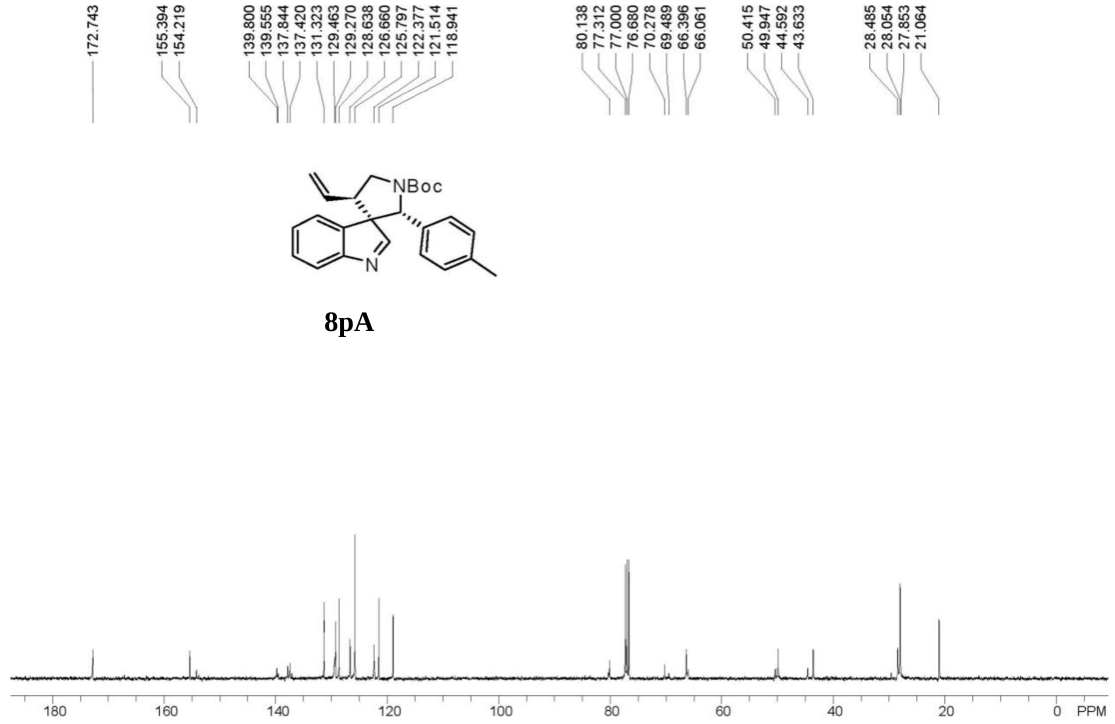

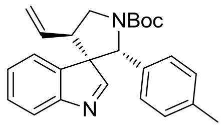

8pA

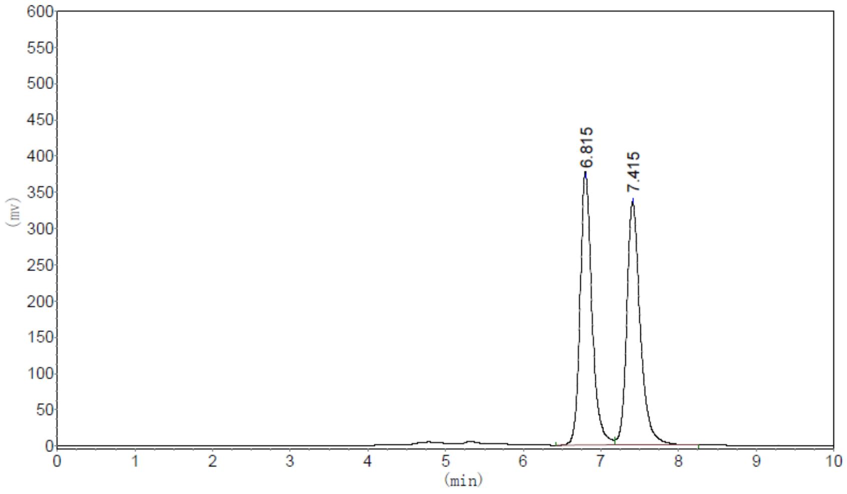

Peak No.	R. Time	Peak Height	Peak Area	Percent
1	6. 815	375170. 125	4056366. 250	49. 6426
2	7. 415	335751. 031	4114778. 250	50. 3574
Total		710921. 156	8171144. 500	100. 0000

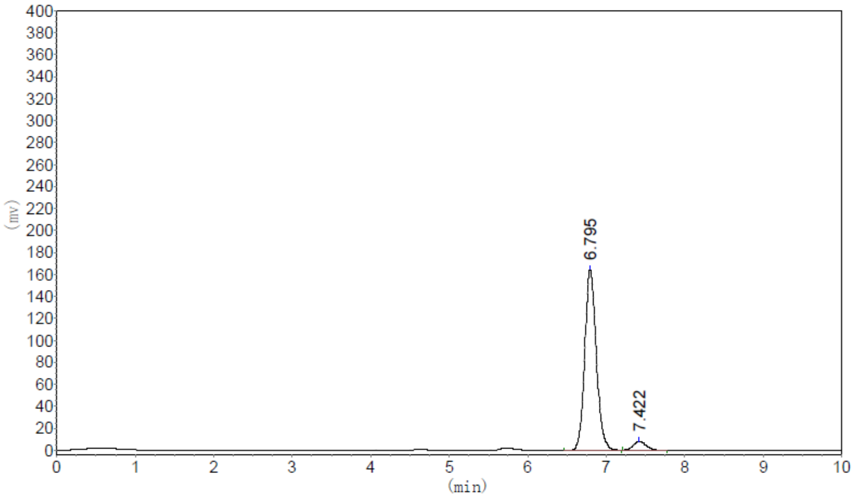

Peak No.	R. Time	Peak Height	Peak Area	Percent
1	6. 795	164808. 938	1689764. 625	95. 1127
2	7. 422	7816. 906	86827. 695	4. 8873
Total		172625. 843	1776592. 320	100. 0000

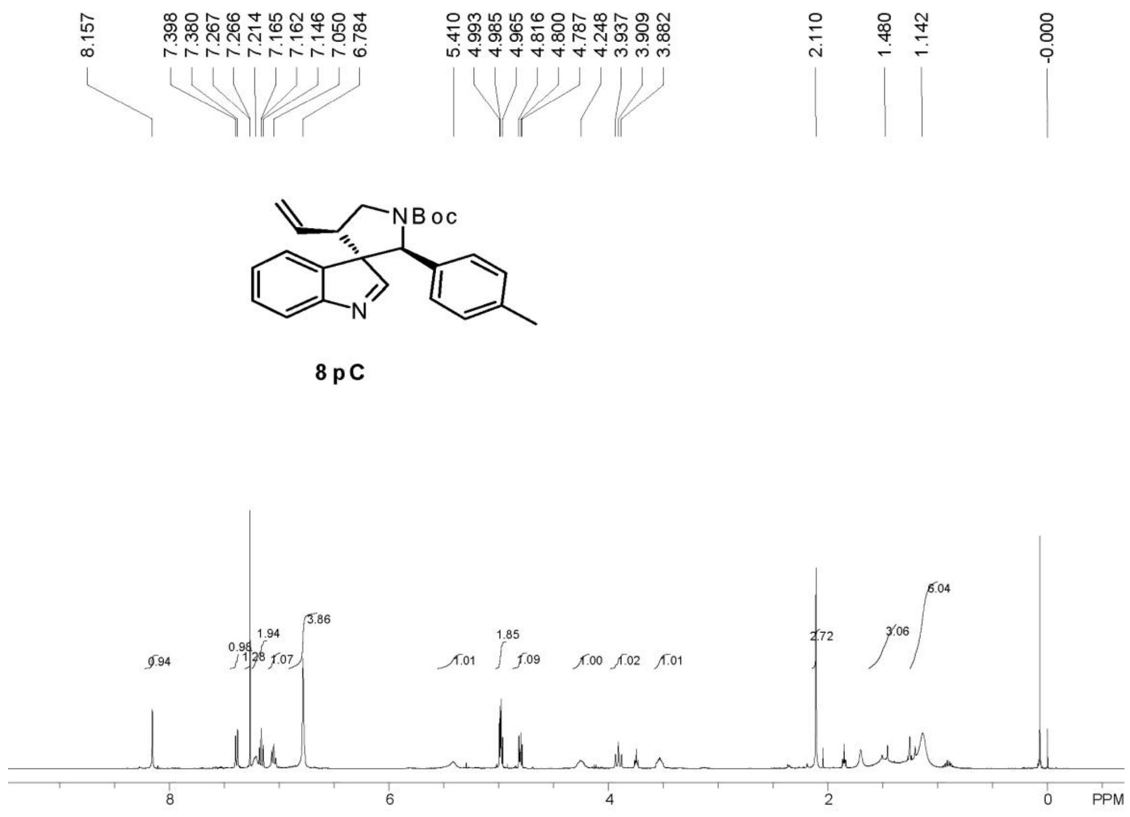
8.157
7.398
7.380
7.267
7.266
7.214
7.165
7.162
7.146
7.050
6.784
5.410
4.993
4.985
4.965
4.816
4.800
4.787
4.248
3.937
3.909
3.882
2.110
1.480
1.142
-0.000
NBoc
N
8pC
0.94
0.98
1.28
1.94
1.07
3.86
1.01
1.85
1.09
1.00
1.02
1.01
2.72
3.06
6.04
8
6
4
2
0
PPM

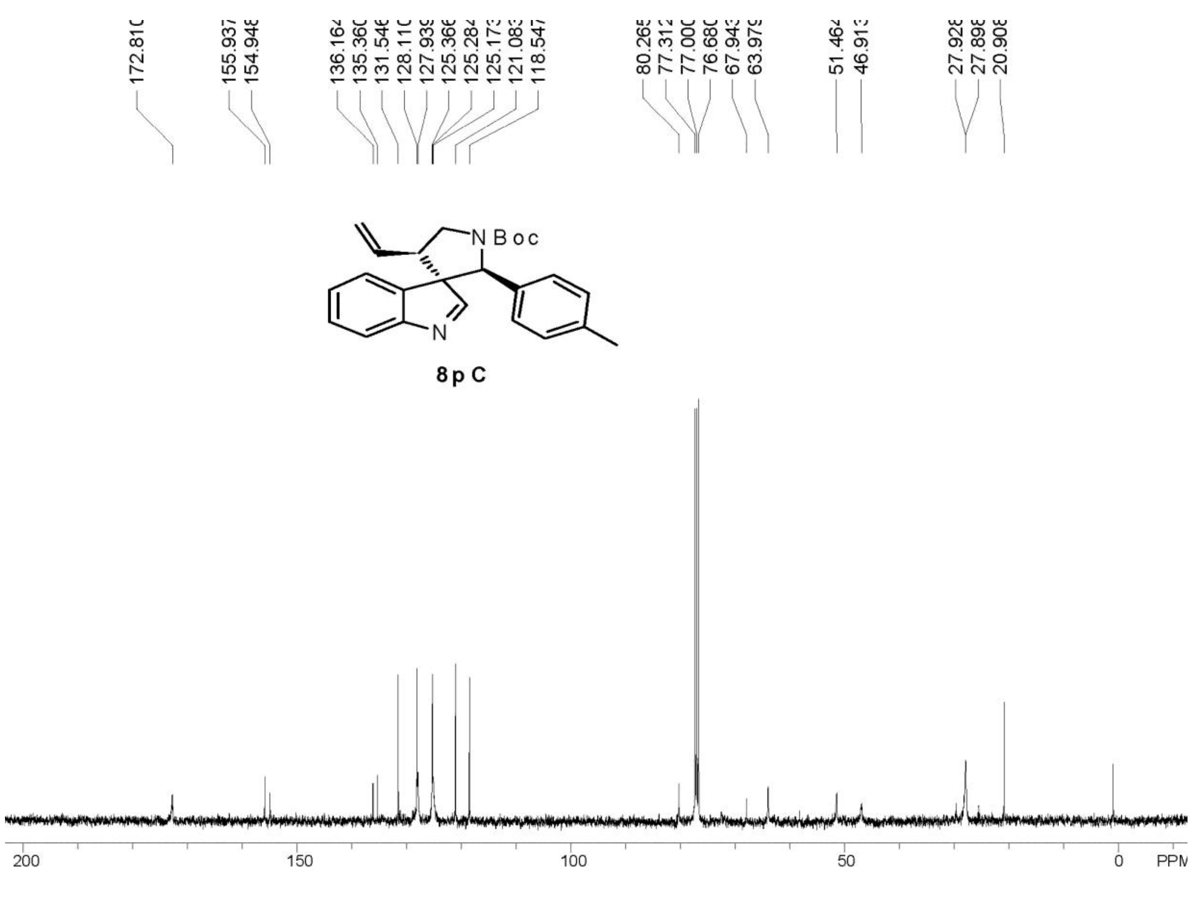
172.810
155.937
154.948
136.164
135.360
131.546
128.110
127.939
125.366
125.284
125.173
121.083
118.547
80.265
77.312
77.000
76.680
67.943
63.979
51.464
46.913
27.928
27.898
20.908
NBoc
N
8pC
200
150
100
50
0
PPM

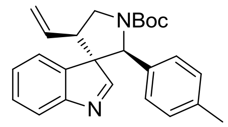

8pC

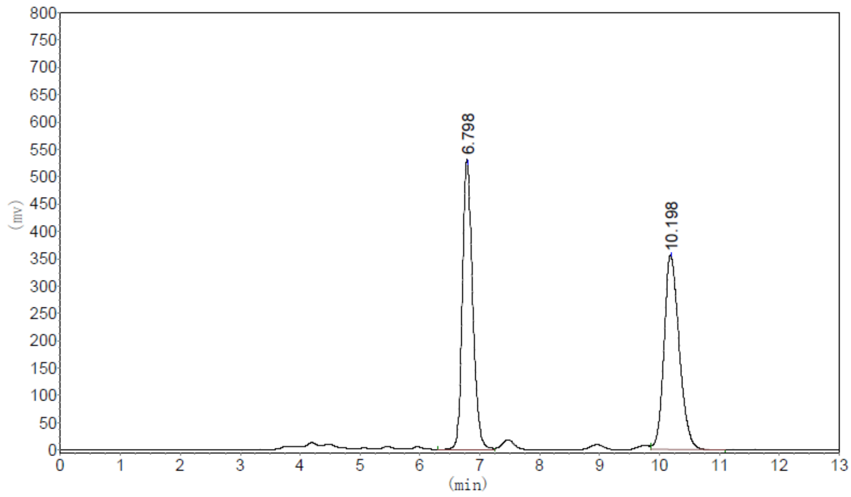

Peak No.	R. Time	Peak Height	Peak Area	Percent
1	6.798	528264.688	6353871.500	50.0621
2	10.198	355803.375	6338106.000	49.9379
Total		884068.063	12691977.500	100.0000

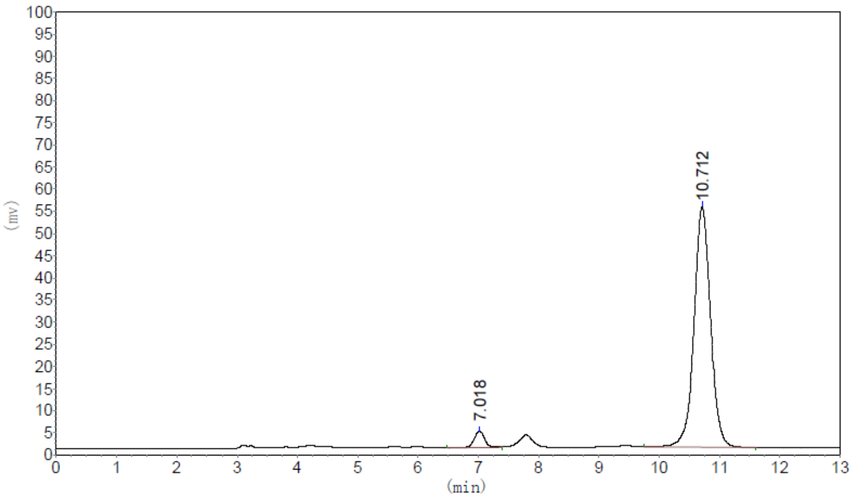

Peak No.	R. Time	Peak Height	Peak Area	Percent
1	7.018	3684.283	44025.281	4.0843
2	10.712	54371.801	1033876.938	95.9156
Total		58056.084	1077902.219	100.0000

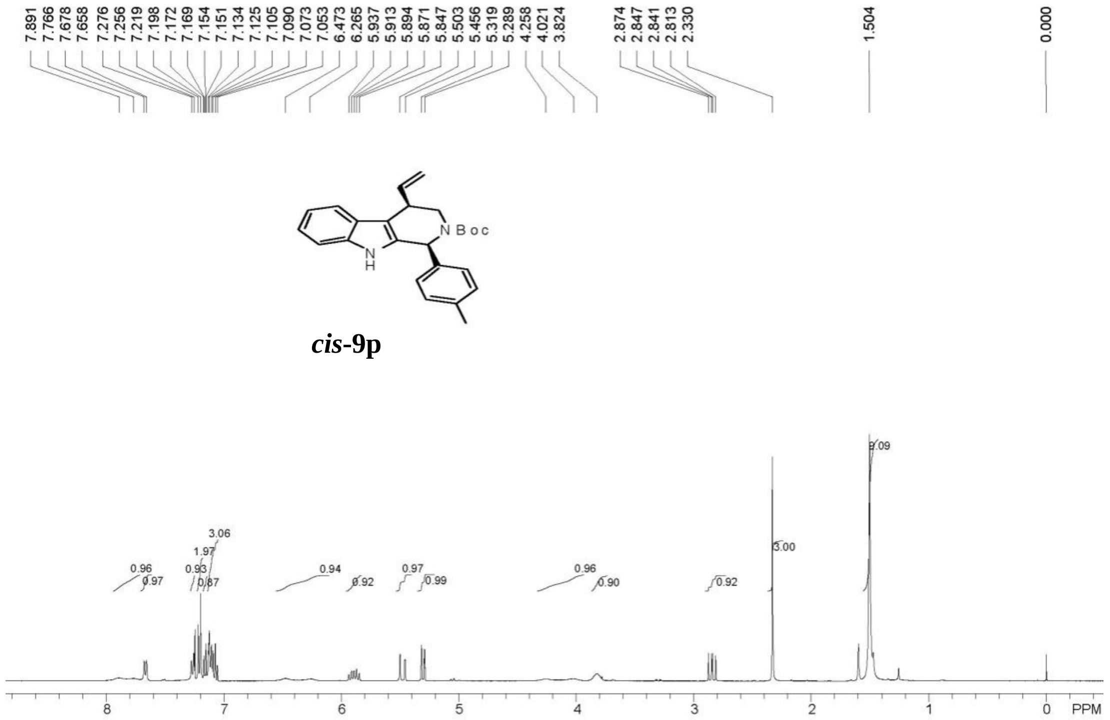
7.891 7.766 7.678 7.658 7.276 7.256 7.219 7.198 7.172 7.169 7.154 7.151 7.134 7.125 7.105 7.090 7.073 7.053 6.473 6.265 5.937 5.913 5.894 5.871 5.847 5.503 5.456 5.319 5.289 4.258 4.021 3.824
2.874 2.847 2.841 2.813 2.330
1.504
0.000
N Boc
N
H
cis-9p
0.96 0.97 0.93 1.97 0.87 3.06 0.94 0.92 0.97 0.99 0.96 0.90 0.92 3.00 9.09
8 7 6 5 4 3 2 1 0 PPM

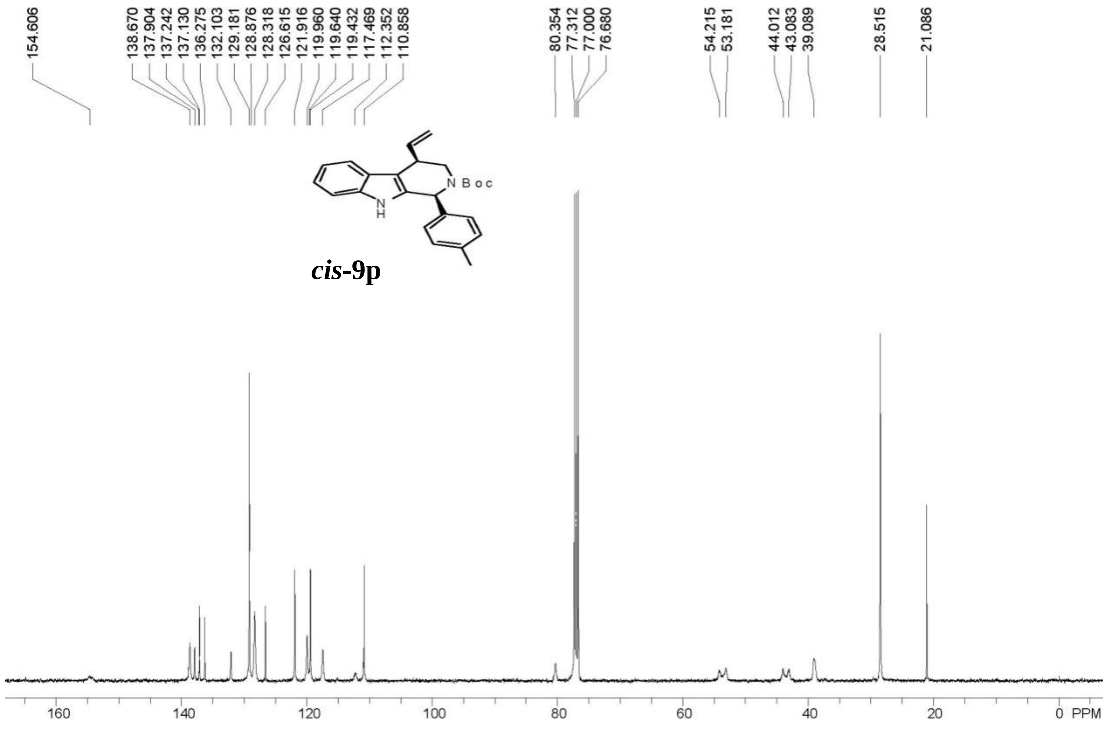
154.606
138.670 137.904 137.242 137.130 136.275 132.103 129.181 128.876 128.318 126.615 121.916 119.960 119.640 119.432 117.469 112.352 110.858
80.354 77.312 77.000 76.680
54.215 53.181
44.012 43.083 39.089
28.515
21.086
N Boc
N
H
cis-9p
160 140 120 100 80 60 40 20 0 PPM

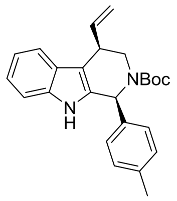

***cis*-9p**

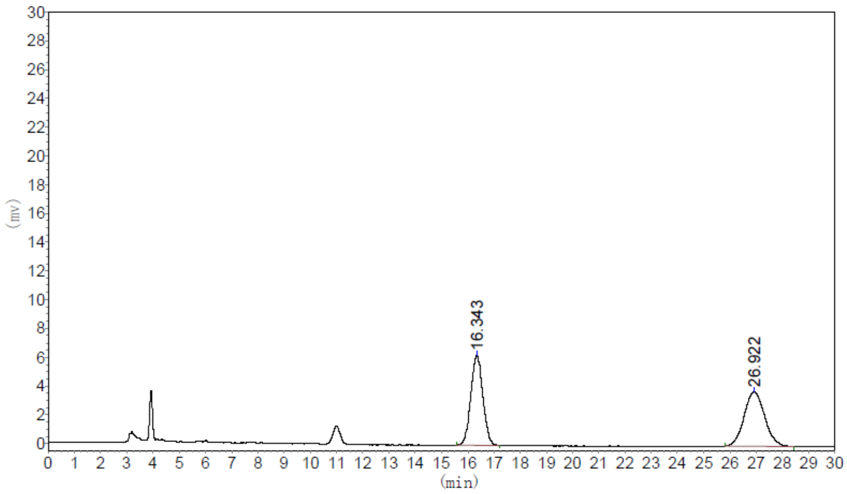

Peak No.	R. Time	Peak Height	Peak Area	Percent
1	16. 343	6266. 183	200439. 453	49. 4339
2	26. 922	3789. 044	205029. 891	50. 5661
Total		10055. 227	405469. 344	100. 0000

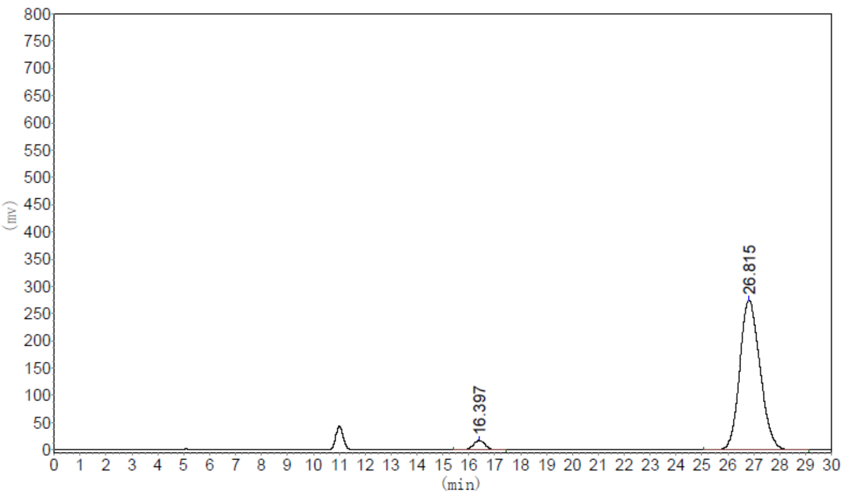

Peak No.	R. Time	Peak Height	Peak Area	Percent
1	16. 397	16924. 064	544821. 875	3. 5217
2	26. 815	274747. 563	14925622. 000	96. 4783
Total		291671. 627	15470443. 875	100. 0000

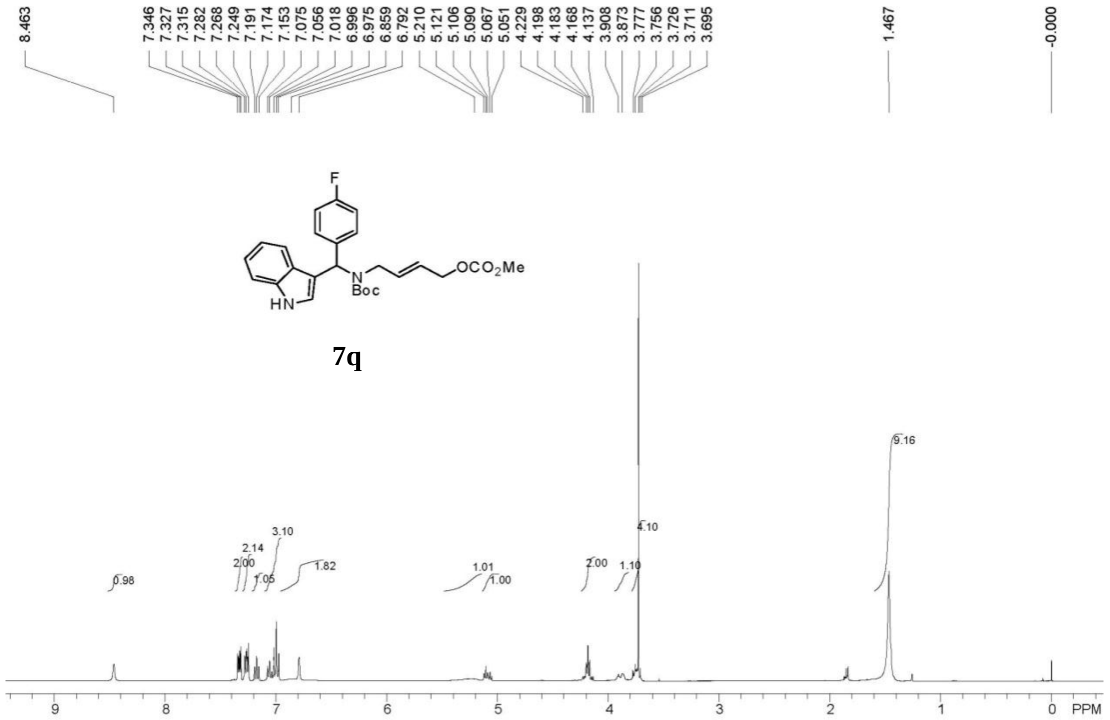
8.463
7.346
7.327
7.315
7.282
7.268
7.249
7.191
7.174
7.153
7.075
7.056
7.018
6.996
6.975
6.859
6.792
5.210
5.121
5.106
5.090
5.067
5.051
4.229
4.198
4.183
4.168
4.137
3.908
3.873
3.777
3.756
3.726
3.711
3.695
1.467
-0.000
F
N
Boc
OCO2Me
HN
0.98
2.00
2.14
1.05
3.10
1.82
1.01
1.00
2.00
1.10
4.10
9.16
9
8
7
6
5
4
3
2
1
0
PPM

7q

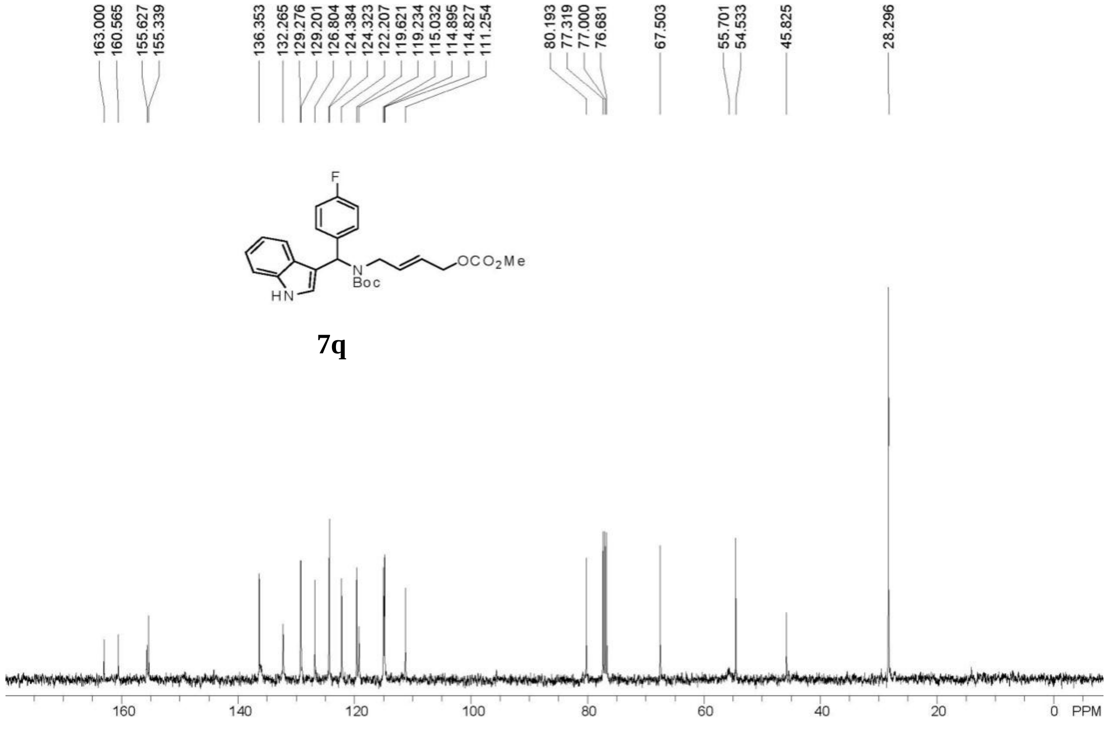
163.000
160.565
155.627
155.339
136.353
132.265
129.276
129.201
126.804
124.384
124.323
122.207
119.621
119.234
115.032
114.895
114.827
111.254
80.193
77.319
77.000
76.681
67.503
55.701
54.533
45.825
28.296
F
N
Boc
OCO2Me
HN
160
140
120
100
80
60
40
20
0
PPM

7q

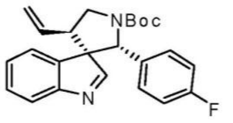
NBoc
N
F

8qA

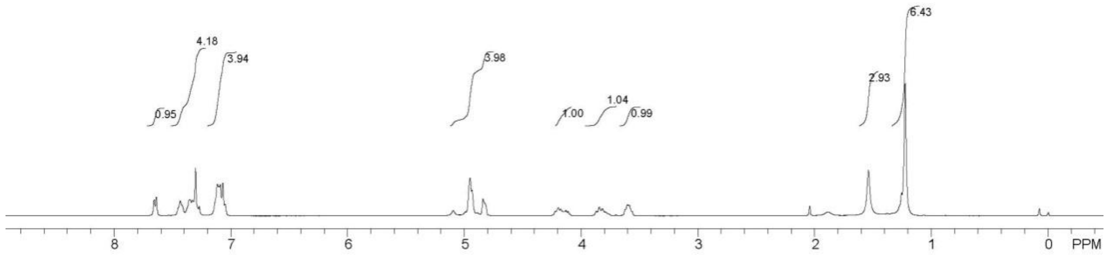
7.657
7.638
7.433
7.350
7.328
7.303
7.272
7.115
7.092
7.070
7.050
5.096
4.991
4.951
4.934
4.841
4.827
4.220
4.198
4.174
4.144
4.130
4.112
3.871
3.845
3.818
3.796
3.622
3.603
3.588
3.566
1.540
1.224
0.000
0.95
4.18
3.94
3.98
1.00
1.04
0.99
2.93
6.43
8
7
6
5
4
3
2
1
0
PPM

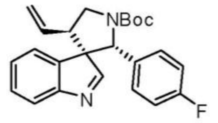
NBoc
N
F

8qA

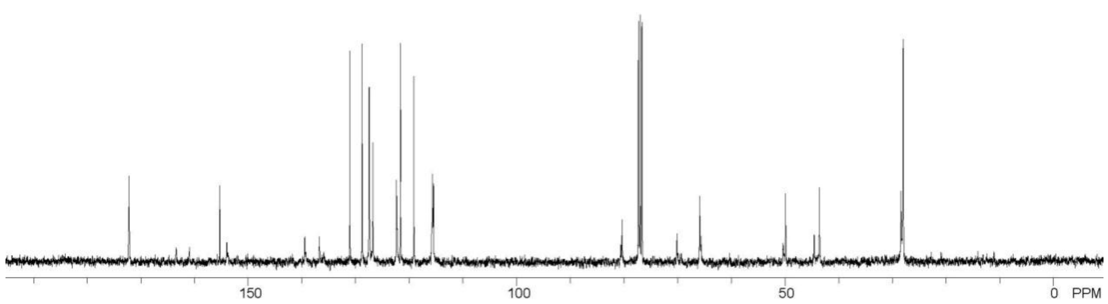
172.185
163.403
160.956
155.305
153.996
139.465
136.729
131.062
128.787
127.485
127.411
126.779
122.369
121.626
119.142
115.691
115.476
80.599
80.361
77.320
77.000
76.680
70.151
65.912
65.652
50.363
49.924
44.600
43.611
28.441
28.024
150
100
50
0
PPM

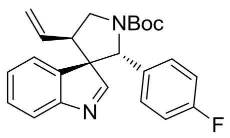

8qA

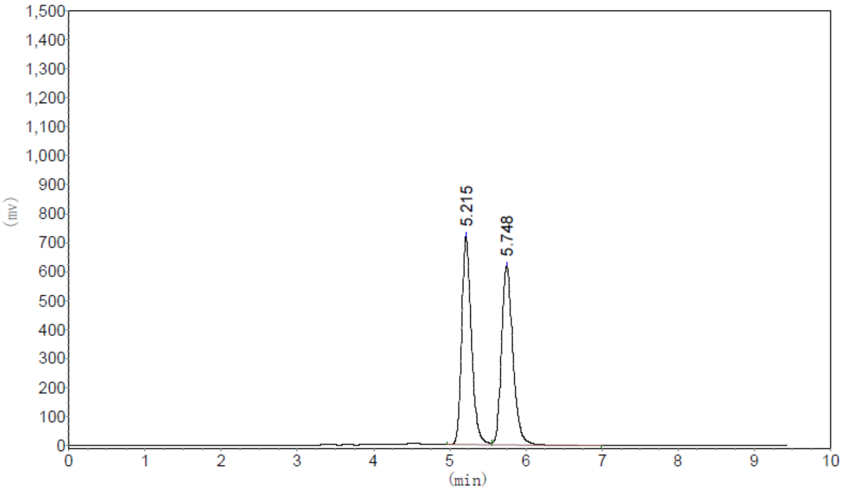

Peak No.	R. Time	Peak Height	Peak Area	Percent
1	5.215	715990.125	6481595.000	49.7867
2	5.748	615933.625	6537142.000	50.2133
Total		1331923.750	13018737.000	100.0000

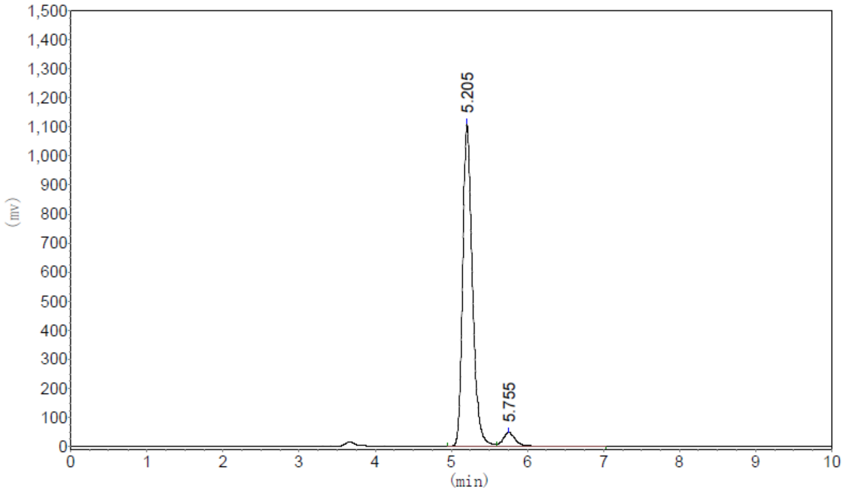

Peak No.	R. Time	Peak Height	Peak Area	Percent
1	5.205	1113130.500	9902280.000	94.5157
2	5.755	48289.785	574587.563	5.4843
Total		1161420.285	10476867.563	100.0000

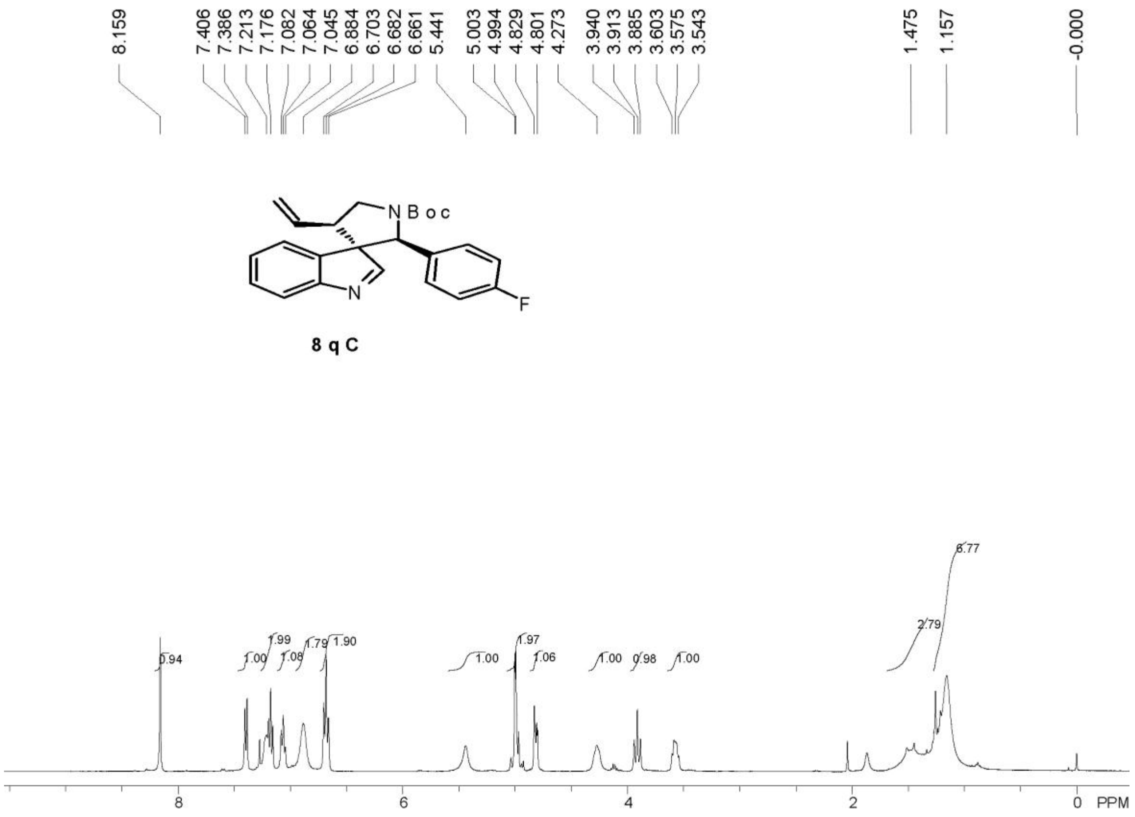
8.159
7.406 7.386 7.213 7.176 7.082 7.064 7.045 6.884 6.703 6.682 6.661 5.441
5.003 4.994 4.829 4.801 4.273 3.940 3.913 3.885 3.603 3.575 3.543
1.475 1.157
-0.000
N B o c
N
F
8 q C
0.94 1.00 1.99 1.08 1.79 1.90 1.00 1.97 1.06 1.00 0.98 1.00 2.79 6.77
8 6 4 2 0 PPM

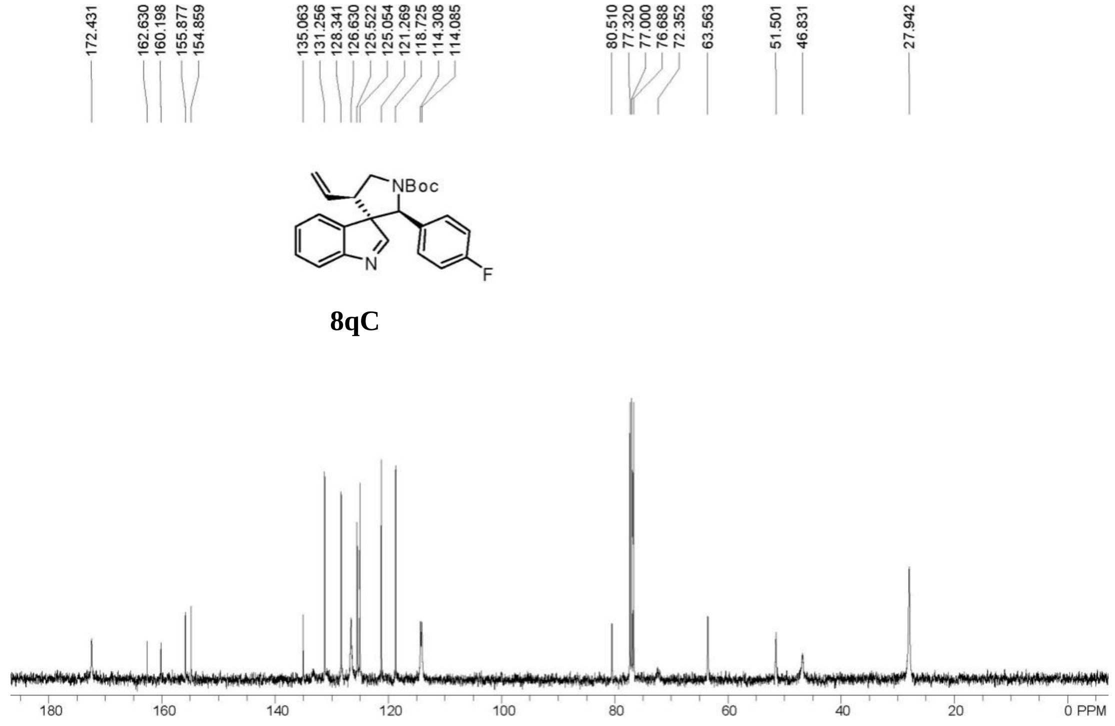
172.431
162.630 160.198 155.877 154.859
135.063 131.256 128.341 126.630 125.522 125.054 121.269 118.725 114.308 114.085
80.510 77.320 77.000 76.688 72.352 63.563
51.501 46.831
27.942
NBoc
N
F
180 160 140 120 100 80 60 40 20 0 PPM

8qC

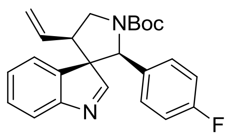

8qC

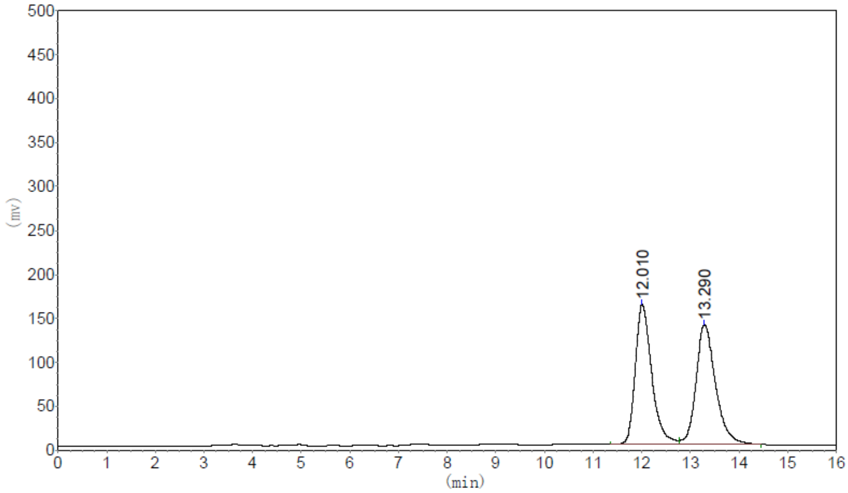

Peak No.	R. Time	Peak Height	Peak Area	Percent
1	12. 010	159088. 563	3729934. 000	49. 4231
2	13. 290	136327. 531	3817005. 500	50. 5769
Total		295416. 094	7546939. 500	100. 0000

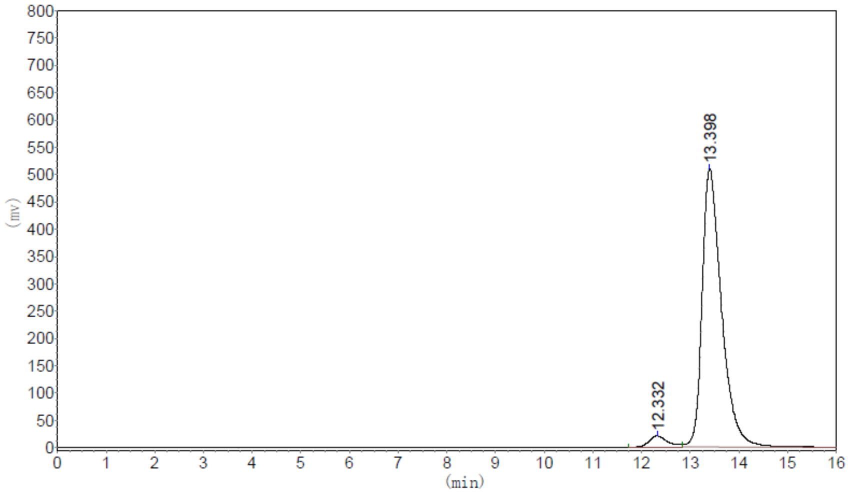

Peak No.	R. Time	Peak Height	Peak Area	Percent
1	12. 332	22560. 037	678070. 938	4. 4418
2	13. 398	508400. 938	14587509. 000	95. 5582
Total		530960. 975	15265579. 938	100. 0000

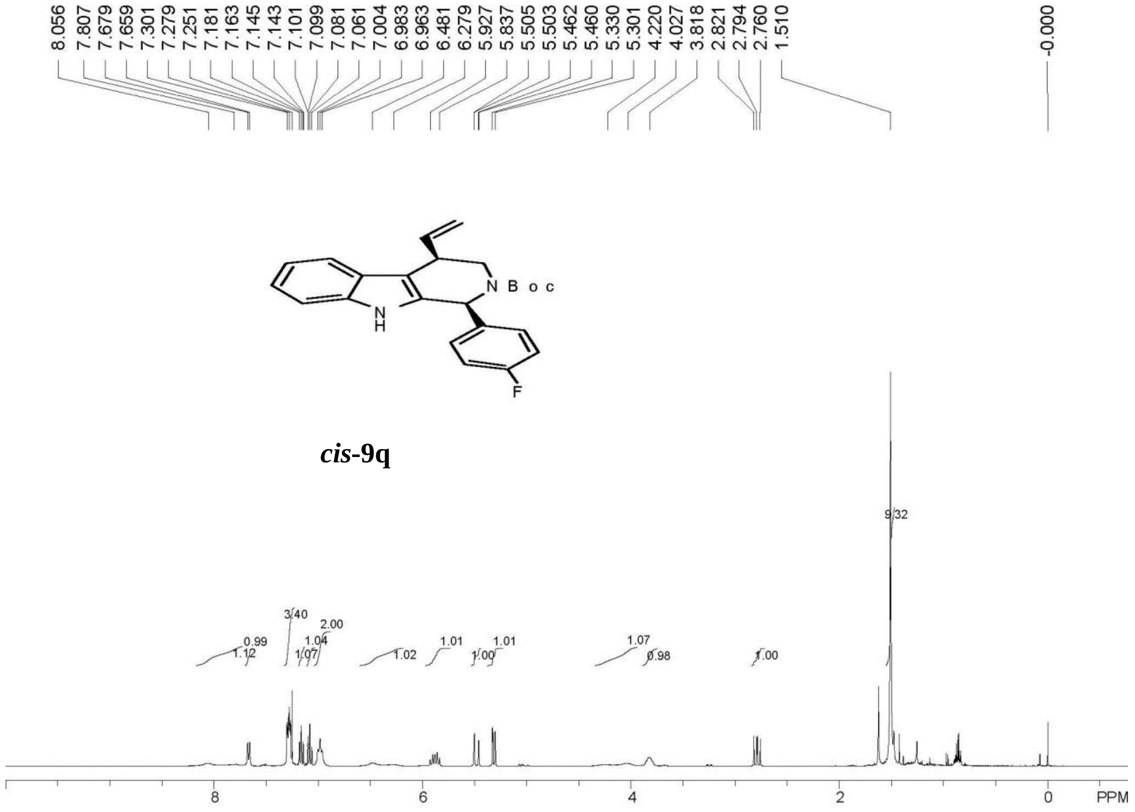
8.056
7.807
7.679
7.659
7.301
7.279
7.251
7.181
7.163
7.145
7.143
7.101
7.099
7.081
7.061
7.004
6.983
6.963
6.481
6.279
5.927
5.837
5.505
5.503
5.462
5.460
5.330
5.301
4.220
4.027
3.818
2.821
2.794
2.760
1.510
-0.000
NBoc
H
F
PPM

cis-**9q**

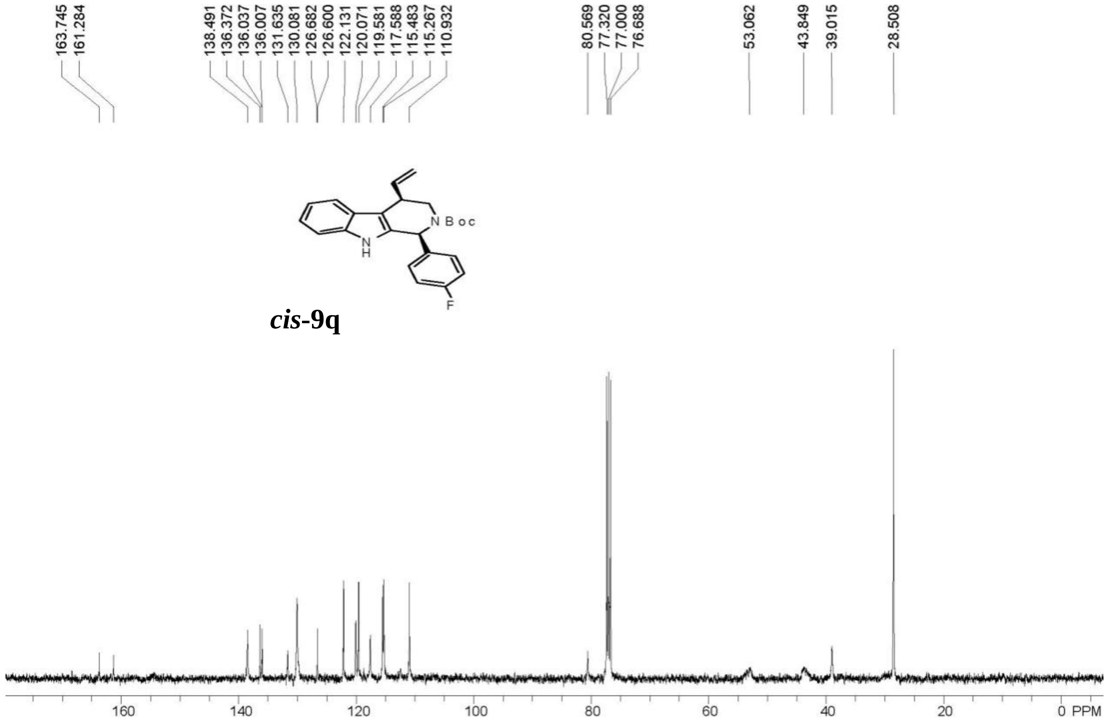
163.745
161.284
138.491
136.372
136.037
136.007
131.635
130.081
126.682
126.600
122.131
120.071
119.581
117.588
115.483
115.267
110.932
80.569
77.320
77.000
76.688
53.062
43.849
39.015
28.508
NBoc
H
F
PPM

cis-**9q**

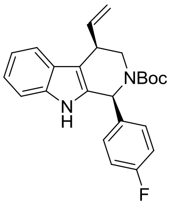

***cis*-9q**

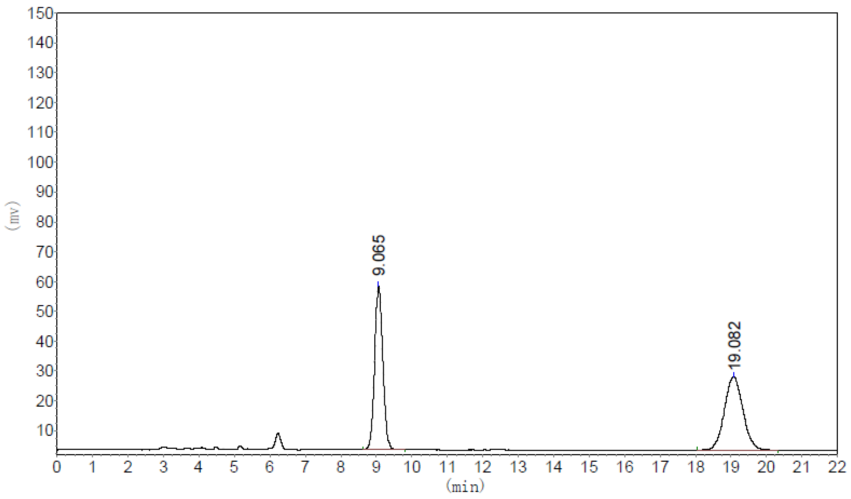

Peak No.	R. Time	Peak Height	Peak Area	Percent
1	9.065	54601.559	876749.250	49.8421
2	19.082	24524.379	882303.625	50.1579
**Total**		79125.938	1759052.875	100.0000

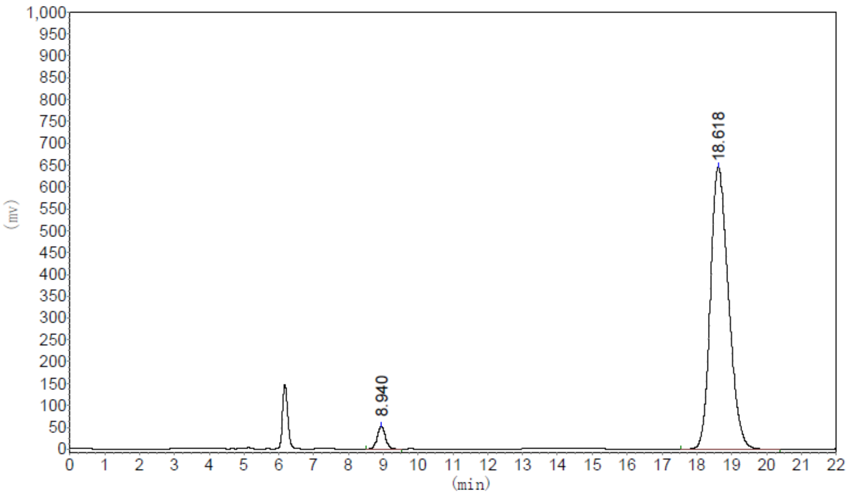

Peak No.	R. Time	Peak Height	Peak Area	Percent
1	8.940	51435.746	803826.313	3.3601
2	18.618	645143.188	23119218.000	96.6399
**Total**		696578.934	23923044.313	100.0000

References

1. For reviews on chiral phosphoramidite ligands: (a) Feringa, B. L. *Acc. Chem. Res.* **2000**, *33*, 346. (b) van den Berg, M.; Minnaard, A. J.; Haak, R. M.; Leeman, M.; Schudde, E. P.; Meetsma, A.; Feringa, B. L.; de Vries, A. H. M.; Maljaars, C. E. P.; Willans, C. E.; Hyett, D.; Boogers, J. A. F.; Henderickx, H. J. W.; de Vries, J. G. *Adv. Synth. Catal.* **2003**, *345*, 308. (c) Minnaard, A. J.; Feringa, B. L.; Lefort, L.; de Vries, J. G. *Acc. Chem. Res.* **2007**, *40*, 1267.
2. (a) Tissot-Croset, K.; Polet, D.; Alexakis, A. *Angew. Chem., Int. Ed.* **2004**, *43*, 2426. (b) Alexakis, A.; Polet, D. *Org. Lett.* **2004**, *6*, 3529. (c) Polet, D.; Alexakis, A. *Org. Lett.* **2005**, *7*, 1621.
3. (a) Liu, W.-B.; He, H.; Dai, L.-X.; You, S.-L. *Synthesis* **2009**, 2076. (b) Liu, W.-B.; Zheng, C.; Zhuo, C.-X.; Dai, L.-X.; You, S.-L. *J. Am. Chem. Soc.* **2012**, *134,* 4812.
4. Shu, C.; Leitner, A.; Hartwig, J. F. *Angew. Chem., Int. Ed.* **2004**, *43*, 4797.
5. The relative configuration of ***trans*-9a** is determined by X-ray crystallographic analysis of a racemic sample. Since the absolute configuration of the allylic position (C10) is known as (*R*) and preserved during the N–Ts iminium migration process, the absolute configuration of the benzylic position (C8) of ***trans*-9a** is then assigned as (*R*).
6. Alexakis, A.; Rosset, S.; Allamand, J.; March, S.; Guillen, F.; Benhaim, C. *Synlett* **2001**, 1375.
7. Wang, Y.-Q.; Song, J.; Hong, R.; Li, H.; Deng, L. *J. Am. Chem. Soc.* **2006**, *128*, 8156.
8. Trost, B. M.; Sacchi, K. L.; Schroeder, G. M.; Asakawa, N. *Org. Lett.* **2002**, *4*, 3427.

Computational methods

All calculations in this paper were performed with the Gaussian09 package.[1] The density functional theory (DFT) method was employed using the PBE1PBE functional.[2] The standard 6-311+G(d,p) basis sets were applied for all atoms. Optimizations were conducted without any constraint using SMD model[3] in THF (ε = 7.4257). These methods have been used in the calculations on the related acid-catalyzed stereoselective allyl migration reaction previously.[4] Frequency analyses were carried out to confirm each structure being a minimum (no imaginary frequency) or a transition state (only one imaginary frequency). The natural bond orbital (NBO) analyses were performed using NBO 3.1 implemented in Gaussian09. Throughout this paper, we mainly discuss the Gibbs free energies in THF (ΔG_{THF}), unless specified. The electronic energies in THF including zero-point energy corrections (ΔE_{THF}) are also given in parentheses for reference. All figures of the calculated 3D structures were prepared using CYLview.[5]

Calculated reaction profiles of the aryl N–Ts iminium migration processes of the isomers 8aA-C

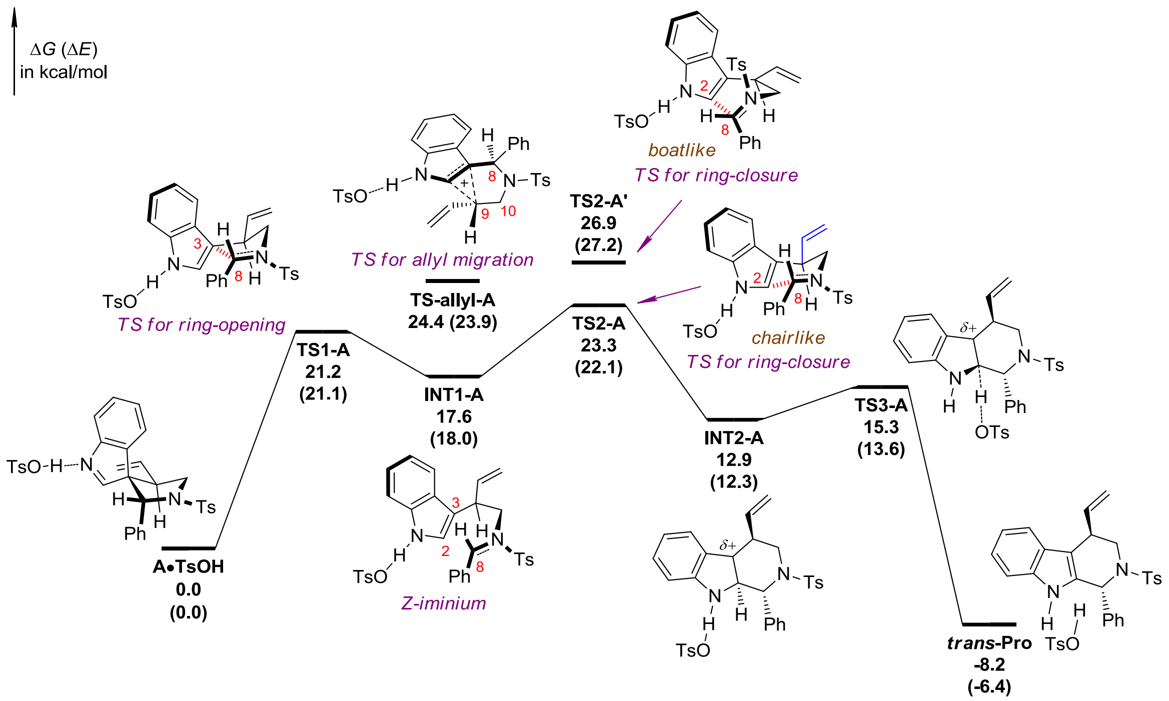

Scheme S1. The reaction profiles for the TsOH-catalyzed aryl N–Ts iminium migration of isomer **8aA**. Calculated at PBE1PBE/6-311+G(d,p) level of theory. Δ*G* and (Δ*E*) values are in kcal/mol.

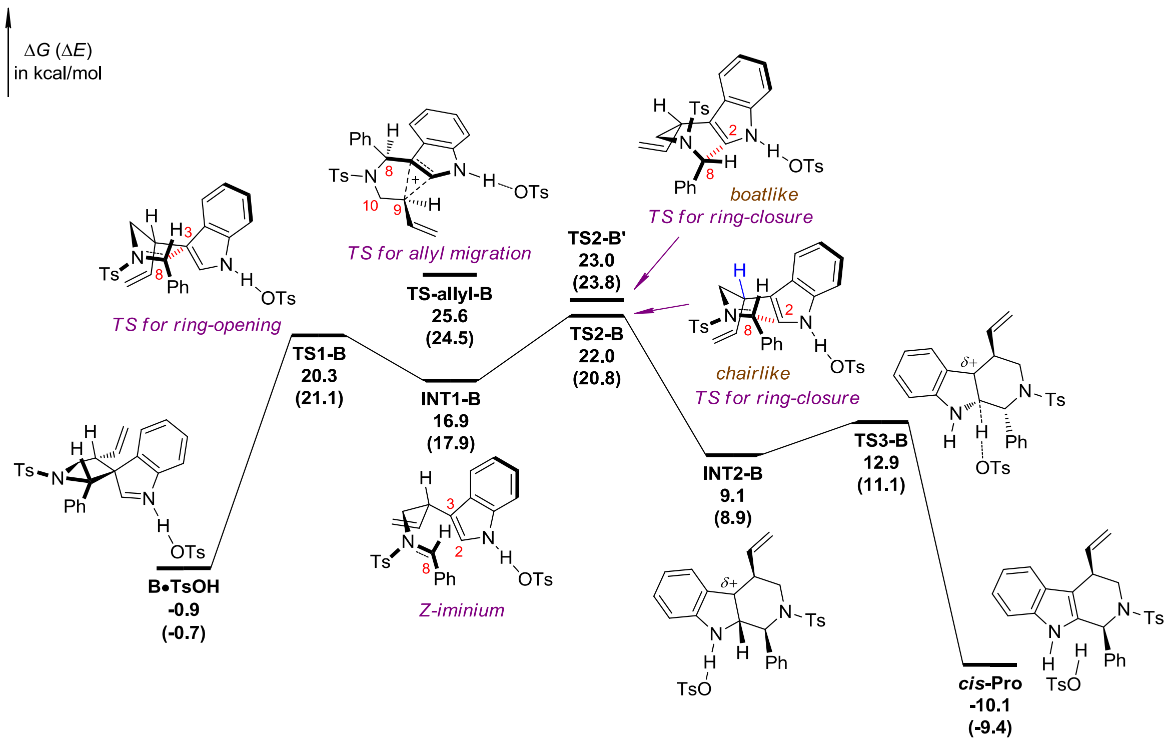

Scheme S2. The reaction profiles for the TsOH-catalyzed aryl N–Ts iminium migration of isomer **8aB**.

Calculated at PBE1PBE/6-311+G(d,p) level of theory. Δ*G* and (Δ*E*) values are in kcal/mol.

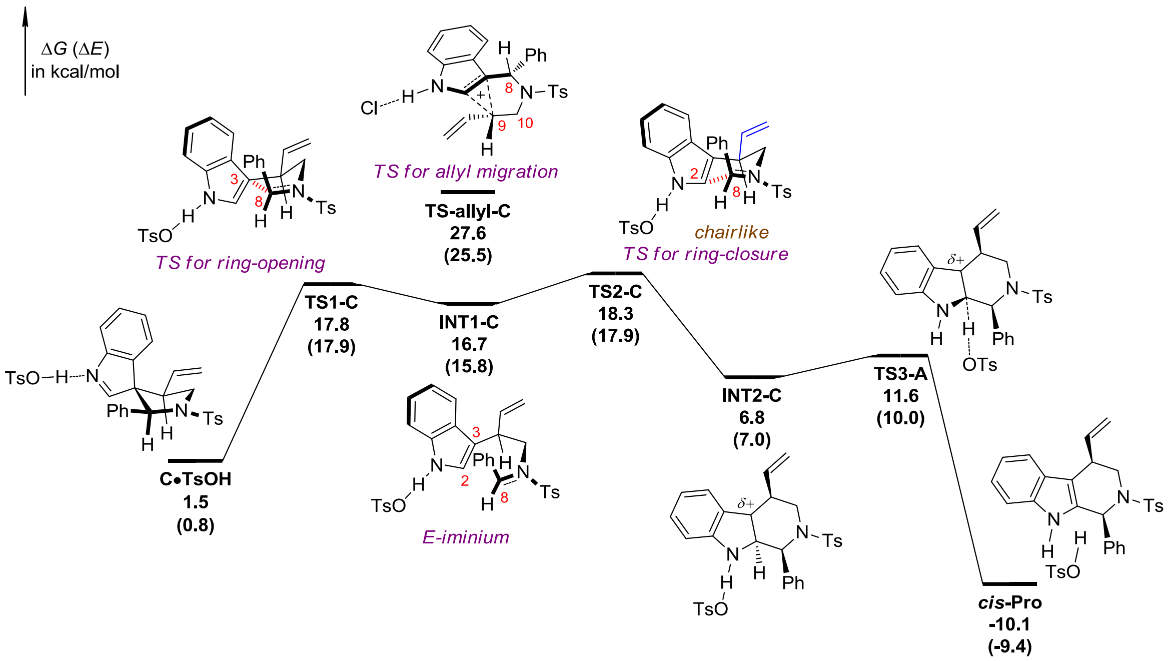

Scheme S3. The reaction profiles for the TsOH-catalyzed aryl N–Ts iminium migration of isomer **8aC**. Calculated at PBE1PBE/6-311+G(d,p) level of theory. Δ*G* and (Δ*E*) values are in kcal/mol.

NBO analyses of INT1-A, INT1-B and INT1-C

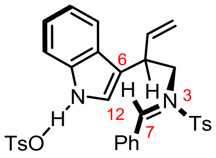

INT1-A

NATURAL POPULATIONS: Natural atomic orbital occupancies

NAO	Atom	No	lang	Type(AO)	Occupancy	Energy
53	N	3	S	Cor(1S)	1.99919	-14.32916
54	N	3	S	Val(2S)	1.30791	-0.64308
55	N	3	S	Ryd(4S)	0.00361	1.52007
56	N	3	S	Ryd(3S)	0.00073	1.03182
57	N	3	S	Ryd(5S)	0.00000	35.15778
58	N	3	px	Val(2p)	1.45507	-0.34567
59	N	3	px	Ryd(4p)	0.00675	0.92320
60	N	3	px	Ryd(3p)	0.00131	0.80260
61	N	3	px	Ryd(5p)	0.00009	4.01617
62	N	3	py	Val(2p)	1.33685	-0.32693
63	N	3	py	Ryd(4p)	0.00755	1.16165
64	N	3	py	Ryd(3p)	0.00119	1.05908
65	N	3	py	Ryd(5p)	0.00004	4.17171
66	N	3	pz	Val(2p)	1.46243	-0.34574
67	N	3	pz	Ryd(4p)	0.00534	0.87394
68	N	3	pz	Ryd(3p)	0.00107	0.78445
69	N	3	pz	Ryd(5p)	0.00007	3.96941
70	N	3	dxy	Ryd(3d)	0.00074	2.45070
71	N	3	dxz	Ryd(3d)	0.00063	2.64249
72	N	3	dyz	Ryd(3d)	0.00078	2.42494
73	N	3	dx2y2	Ryd(3d)	0.00071	2.58169
74	N	3	dz2	Ryd(3d)	0.00050	2.32882
119	C	6	S	Cor(1S)	1.99898	-10.08181
120	C	6	S	Val(2S)	0.86581	-0.12418
121	C	6	S	Ryd(3S)	0.00182	1.09466
122	C	6	S	Ryd(4S)	0.00096	1.50262
123	C	6	S	Ryd(5S)	0.00004	22.90551
124	C	6	px	Val(2p)	1.08008	-0.07047
125	C	6	px	Ryd(4p)	0.00470	1.10743
126	C	6	px	Ryd(3p)	0.00093	0.79865
127	C	6	px	Ryd(5p)	0.00014	3.17519
128	C	6	py	Val(2p)	1.09345	-0.09171
129	C	6	py	Ryd(3p)	0.00553	0.89923
130	C	6	py	Ryd(4p)	0.00063	0.92937
131	C	6	py	Ryd(5p)	0.00012	3.12788
132	C	6	pz	Val(2p)	1.06903	-0.05687
133	C	6	pz	Ryd(4p)	0.00444	0.83217
134	C	6	pz	Ryd(3p)	0.00112	0.80342
135	C	6	pz	Ryd(5p)	0.00011	3.38639
136	C	6	dxy	Ryd(3d)	0.00064	2.34499
137	C	6	dxz	Ryd(3d)	0.00084	2.42491
138	C	6	dyz	Ryd(3d)	0.00051	2.10413
139	C	6	dx2y2	Ryd(3d)	0.00101	2.03259

```
   140    C    6  dz2     Ryd( 3d)     0.00071      2.13993

   141    C    7  S      Cor( 1S)     1.99911    -10.19761
   142    C    7  S      Val( 2S)     0.91850     -0.22534
   143    C    7  S      Ryd( 3S)     0.00501      1.02279
   144    C    7  S      Ryd( 4S)     0.00153      1.77394
   145    C    7  S      Ryd( 5S)     0.00006     21.75981
   146    C    7  px     Val( 2p)     0.80470     -0.11468
   147    C    7  px     Ryd( 3p)     0.00479      0.70278
   148    C    7  px     Ryd( 4p)     0.00116      0.70750
   149    C    7  px     Ryd( 5p)     0.00010      3.14394
   150    C    7  py     Val( 2p)     1.05164     -0.10107
   151    C    7  py     Ryd( 4p)     0.00637      1.01565
   152    C    7  py     Ryd( 3p)     0.00116      0.70138
   153    C    7  py     Ryd( 5p)     0.00008      3.28519
   154    C    7  pz     Val( 2p)     0.94460     -0.12575
   155    C    7  pz     Ryd( 4p)     0.00709      0.79243
   156    C    7  pz     Ryd( 3p)     0.00087      0.58839
   157    C    7  pz     Ryd( 5p)     0.00007      3.08452
   158    C    7  dxy     Ryd( 3d)     0.00082      2.11888
   159    C    7  dxz     Ryd( 3d)     0.00068      2.09934
   160    C    7  dyz     Ryd( 3d)     0.00122      2.16132
   161    C    7  dx2y2    Ryd( 3d)     0.00136      2.06726
   162    C    7  dz2     Ryd( 3d)     0.00121      1.90253

   251    C   12  S      Cor( 1S)     1.99906    -10.11459
   252    C   12  S      Val( 2S)     0.91870     -0.16042
   253    C   12  S      Ryd( 3S)     0.00151      0.94852
   254    C   12  S      Ryd( 4S)     0.00005      1.71682
   255    C   12  S      Ryd( 5S)     0.00002     22.31929
   256    C   12  px     Val( 2p)     0.90141     -0.05282
   257    C   12  px     Ryd( 3p)     0.00527      0.78964
   258    C   12  px     Ryd( 4p)     0.00011      1.30080
   259    C   12  px     Ryd( 5p)     0.00011      2.49907
   260    C   12  py     Val( 2p)     1.03199     -0.09635
   261    C   12  py     Ryd( 3p)     0.00399      0.80021
   262    C   12  py     Ryd( 4p)     0.00012      1.28113
   263    C   12  py     Ryd( 5p)     0.00007      2.27864
   264    C   12  pz     Val( 2p)     1.08884     -0.06921
   265    C   12  pz     Ryd( 3p)     0.00478      1.04620
   266    C   12  pz     Ryd( 4p)     0.00010      1.28225
   267    C   12  pz     Ryd( 5p)     0.00008      2.43083
   268    C   12  dxy     Ryd( 3d)     0.00073      2.16079
   269    C   12  dxz     Ryd( 3d)     0.00099      2.36532
   270    C   12  dyz     Ryd( 3d)     0.00105      2.16885
   271    C   12  dx2y2    Ryd( 3d)     0.00136      1.89517
   272    C   12  dz2     Ryd( 3d)     0.00104      2.09773
------------------------------------------------------------


NATURAL BOND ORBITAL ANALYSIS:
      (Occupancy)   Bond orbital/ Coefficients/ Hybrids
------------------------------------------------------------
```

14. (1.74859) BD (2) C   6 - C 12

```
( 55.45%)   0.7447* C   6 s(  0.06%)p99.99( 99.87%)d 1.28(  0.07%)
                        0.0000 -0.0073  0.0224 -0.0017  0.0017
                       -0.4960 -0.0105 -0.0115  0.0033  0.7611
```

0.0188 0.0030 -0.0014 -0.4154 -0.0089
-0.0140 0.0009 -0.0065 0.0112 0.0029
0.0227 0.0049
(44.55%)   0.6674* C 12 s(0.04%)p99.99(99.87%)d 2.50(0.09%)
-0.0001 -0.0184 0.0054 0.0018 0.0010
-0.5250 -0.0113 0.0013 0.0007 0.7338
0.0134 -0.0004 0.0009 -0.4291 -0.0104
-0.0015 -0.0022 -0.0004 -0.0151 0.0083
-0.0200 -0.0153

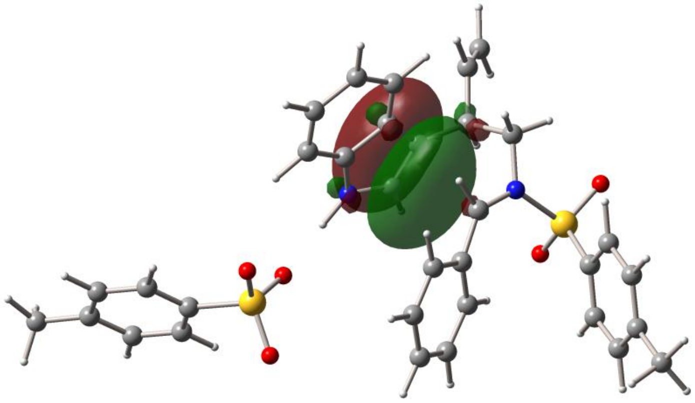

1051. (0.36171) BD*(2) N   3 - C   7

(23.87%)   0.4886* N   3 s(0.44%)p99.99(99.53%)d 0.06(0.03%)
0.0001 -0.0659 -0.0071 -0.0005 -0.0003
-0.6924 0.0113 -0.0008 0.0025 0.1759
0.0056 0.0003 0.0001 -0.6961 0.0140
-0.0054 0.0015 0.0033 0.0031 0.0080
0.0098 -0.0090
(76.13%) -0.8725* C   7 s(0.03%)p99.99(99.67%)d 9.78(0.30%)
0.0001 0.0119 -0.0122 -0.0034 0.0000
-0.7404 0.0131 -0.0124 0.0028 0.2699
0.0139 0.0018 0.0015 -0.6123 0.0167
-0.0069 0.0025 -0.0070 -0.0049 -0.0185
-0.0305 0.0403

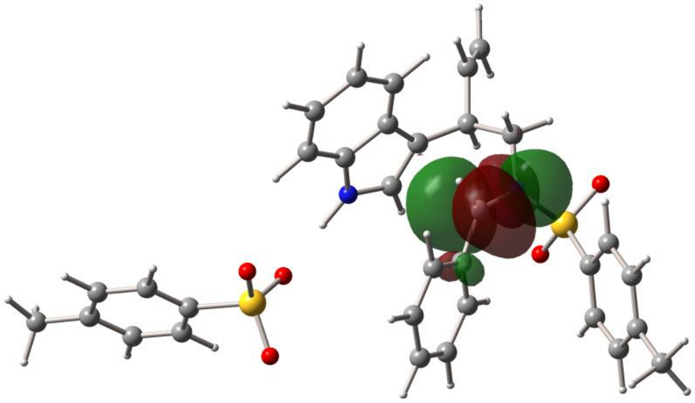

Second Order Perturbation Theory Analysis of Fock Matrix in NBO Basis

Threshold for printing: 0.50 kcal/mol
(Intermolecular threshold: 0.05 kcal/mol)

Donor NBO (i)	Acceptor NBO (j)	E(2) kcal/mol	E(j)-E(i) a.u.	F(i,j) a.u.
14. BD (2) C 6 - C 12	/147. LP (1) C 14	42.18	0.16	0.097
14. BD (2) C 6 - C 12	/182. RY*(1) N 2	1.17	0.76	0.028
14. BD (2) C 6 - C 12	/187. RY*(6) N 2	0.57	2.05	0.033
14. BD (2) C 6 - C 12	/390. RY*(5) C 14	0.70	1.15	0.027
14. BD (2) C 6 - C 12	/***. BD*(2) N 2 - C 13	8.93	0.25	0.048
**14. BD (2) C 6 - C 12**	**/***. BD*(2) N 3 - C 7**	**7.86**	**0.21**	**0.037**
14. BD (2) C 6 - C 12	/***. BD*(2) C 6 - C 12	1.69	0.30	0.021
14. BD (2) C 6 - C 12	/***. BD*(1) C 8 - C 9	4.43	0.62	0.049
14. BD (2) C 6 - C 12	/***. BD*(1) C 9 - C 10	2.76	0.71	0.042

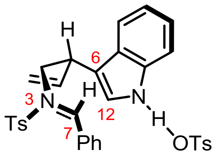

INT1-B

NATURAL POPULATIONS: Natural atomic orbital occupancies

NAO	Atom	No	lang	Type(AO)	Occupancy	Energy
53	N	3	S	Cor(1S)	1.99918	-14.32821
54	N	3	S	Val(2S)	1.30856	-0.64352
55	N	3	S	Ryd(4S)	0.00362	1.46160
56	N	3	S	Ryd(3S)	0.00083	1.10770
57	N	3	S	Ryd(5S)	0.00000	35.16696
58	N	3	px	Val(2p)	1.44966	-0.34664
59	N	3	px	Ryd(4p)	0.00659	0.92359
60	N	3	px	Ryd(3p)	0.00137	0.82200
61	N	3	px	Ryd(5p)	0.00009	4.01879
62	N	3	py	Val(2p)	1.34068	-0.32678
63	N	3	py	Ryd(4p)	0.00772	1.12405
64	N	3	py	Ryd(3p)	0.00110	1.06958
65	N	3	py	Ryd(5p)	0.00004	4.17425
66	N	3	pz	Val(2p)	1.47355	-0.34578
67	N	3	pz	Ryd(4p)	0.00523	0.86588
68	N	3	pz	Ryd(3p)	0.00106	0.84389
69	N	3	pz	Ryd(5p)	0.00006	3.97780
70	N	3	dxy	Ryd(3d)	0.00077	2.47794
71	N	3	dxz	Ryd(3d)	0.00063	2.62357
72	N	3	dyz	Ryd(3d)	0.00075	2.43031
73	N	3	dx2y2	Ryd(3d)	0.00070	2.57821

74	N	3	dz2	Ryd(3d)	0.00050	2.32188
119	C	6	S	Cor(1S)	1.99899	-10.08325
120	C	6	S	Val(2S)	0.86453	-0.12260
121	C	6	S	Ryd(3S)	0.00178	1.06021
122	C	6	S	Ryd(4S)	0.00078	1.52729
123	C	6	S	Ryd(5S)	0.00003	23.02283
124	C	6	px	Val(2p)	1.07998	-0.07179
125	C	6	px	Ryd(4p)	0.00441	1.07556
126	C	6	px	Ryd(3p)	0.00079	1.03055
127	C	6	px	Ryd(5p)	0.00016	2.94361
128	C	6	py	Val(2p)	1.08198	-0.08869
129	C	6	py	Ryd(3p)	0.00426	0.91855
130	C	6	py	Ryd(4p)	0.00055	1.04071
131	C	6	py	Ryd(5p)	0.00010	3.03997
132	C	6	pz	Val(2p)	1.07061	-0.06308
133	C	6	pz	Ryd(3p)	0.00516	0.81230
134	C	6	pz	Ryd(4p)	0.00105	1.00574
135	C	6	pz	Ryd(5p)	0.00010	3.19294
136	C	6	dxy	Ryd(3d)	0.00062	2.38579
137	C	6	dxz	Ryd(3d)	0.00078	2.37827
138	C	6	dyz	Ryd(3d)	0.00048	2.14110
139	C	6	dx2y2	Ryd(3d)	0.00098	2.04279
140	C	6	dz2	Ryd(3d)	0.00077	2.11224
141	C	7	S	Cor(1S)	1.99911	-10.19799
142	C	7	S	Val(2S)	0.91993	-0.22690
143	C	7	S	Ryd(3S)	0.00506	1.01751
144	C	7	S	Ryd(4S)	0.00129	1.91179
145	C	7	S	Ryd(5S)	0.00007	21.41947
146	C	7	px	Val(2p)	0.80754	-0.11511
147	C	7	px	Ryd(3p)	0.00480	0.70777
148	C	7	px	Ryd(4p)	0.00114	0.74066
149	C	7	px	Ryd(5p)	0.00009	3.12557
150	C	7	py	Val(2p)	1.03824	-0.09932
151	C	7	py	Ryd(4p)	0.00646	1.00761
152	C	7	py	Ryd(3p)	0.00110	0.69365
153	C	7	py	Ryd(5p)	0.00007	3.27911
154	C	7	pz	Val(2p)	0.95798	-0.12755
155	C	7	pz	Ryd(4p)	0.00729	0.79842
156	C	7	pz	Ryd(3p)	0.00083	0.57852
157	C	7	pz	Ryd(5p)	0.00006	3.10405
158	C	7	dxy	Ryd(3d)	0.00086	2.12736
159	C	7	dxz	Ryd(3d)	0.00070	2.08269

```
  160    C    7  dyz     Ryd( 3d)     0.00124      2.15294
  161    C    7  dx2y2   Ryd( 3d)     0.00135      2.07119
  162    C    7  dz2     Ryd( 3d)     0.00117      1.91924

  251    C   12  S      Cor( 1S)     1.99907    -10.11682
  252    C   12  S      Val( 2S)     0.91971     -0.16313
  253    C   12  S      Ryd( 3S)     0.00173      0.98122
  254    C   12  S      Ryd( 4S)     0.00004      1.64913
  255    C   12  S      Ryd( 5S)     0.00002     22.22129
  256    C   12  px     Val( 2p)     0.90384     -0.05791
  257    C   12  px     Ryd( 4p)     0.00525      0.74340
  258    C   12  px     Ryd( 3p)     0.00154      0.65194
  259    C   12  px     Ryd( 5p)     0.00011      3.00505
  260    C   12  py     Val( 2p)     1.04023     -0.09446
  261    C   12  py     Ryd( 4p)     0.00435      0.78357
  262    C   12  py     Ryd( 3p)     0.00108      0.56972
  263    C   12  py     Ryd( 5p)     0.00009      2.87892
  264    C   12  pz     Val( 2p)     1.08873     -0.07268
  265    C   12  pz     Ryd( 4p)     0.00433      1.04751
  266    C   12  pz     Ryd( 3p)     0.00073      0.46895
  267    C   12  pz     Ryd( 5p)     0.00011      3.04499
  268    C   12  dxy     Ryd( 3d)     0.00071      2.18788
  269    C   12  dxz     Ryd( 3d)     0.00095      2.33716
  270    C   12  dyz     Ryd( 3d)     0.00107      2.22265
  271    C   12  dx2y2   Ryd( 3d)     0.00140      1.90529
  272    C   12  dz2     Ryd( 3d)     0.00104      2.06268
-----------------------------------------------------------------


NATURAL BOND ORBITAL ANALYSIS:
       (Occupancy)   Bond orbital/ Coefficients/ Hybrids
-----------------------------------------------------------------
```

14. (1.73641) BD (2) C   6 - C 12

```
( 54.89%)   0.7409* C   6 s(  0.05%)p99.99( 99.88%)d 1.22(  0.07%)
                          0.0000 -0.0063  0.0224 -0.0025  0.0015
                          0.5165  0.0102  0.0040 -0.0015  0.7262
                          0.0152  0.0024 -0.0027 -0.4519 -0.0063
                         -0.0033  0.0001  0.0033 -0.0108  0.0042
                          0.0223  0.0052
( 45.11%)   0.6716* C  12 s(  0.08%)p99.99( 99.83%)d 1.21(  0.09%)
                         -0.0002 -0.0262  0.0084  0.0015  0.0010
                          0.5554  0.0131  0.0031  0.0017  0.6867
                          0.0121  0.0008  0.0003 -0.4666 -0.0126
                         -0.0067 -0.0023  0.0043  0.0155  0.0050
                         -0.0191 -0.0164
```

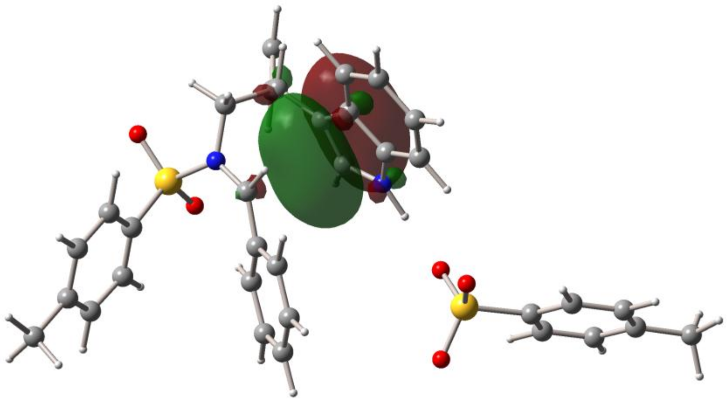

1051. (0.37171) BD*(2) N 3 - C 7

```
( 23.67%)   0.4865* N   3 s(  0.40%)p99.99( 99.58%)d 0.06(  0.03%)
                                       0.0001 -0.0625 -0.0064 -0.0012 -0.0002
                                       0.6676 -0.0114  0.0016 -0.0025  0.2101
                                       0.0046  0.0003 -0.0002 -0.7110  0.0157
                                      -0.0037  0.0015 -0.0032 -0.0033  0.0085
                                       0.0095 -0.0082
( 76.33%)  -0.8737* C   7 s(  0.03%)p99.99( 99.67%)d 9.46(  0.30%)
                                       0.0001  0.0091 -0.0148 -0.0034  0.0000
                                       0.7388 -0.0137  0.0124 -0.0017  0.2843
                                       0.0140  0.0029  0.0013 -0.6076  0.0179
                                      -0.0053  0.0030  0.0066  0.0069 -0.0199
                                      -0.0314  0.0386
```

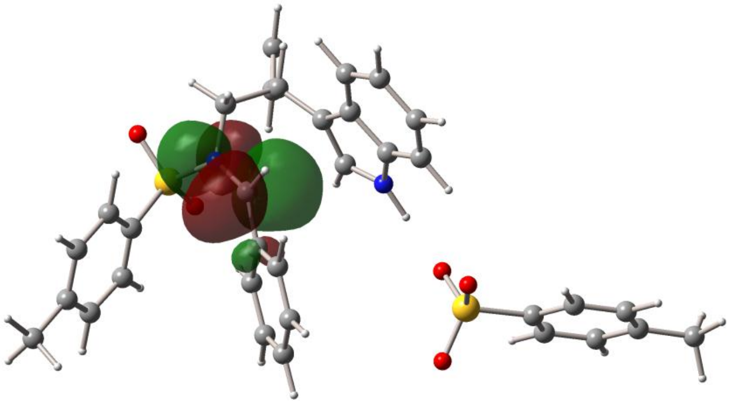

Second Order Perturbation Theory Analysis of Fock Matrix in NBO Basis

Threshold for printing: 0.50 kcal/mol
(Intermolecular threshold: 0.05 kcal/mol)

Donor NBO (i)	Acceptor NBO (j)	E(2) kcal/mol	E(j)-E(i) a.u.	F(i,j) a.u.
14. BD (2) C 6 - C 12	/147. LP (1) C 14	42.54	0.17	0.097
14. BD (2) C 6 - C 12	/182. RY*(1) N 2	1.37	0.76	0.031
14. BD (2) C 6 - C 12	/389. RY*(4) C 14	0.80	1.33	0.031
14. BD (2) C 6 - C 12	/***. BD*(2) N 2 - C 13	8.98	0.25	0.048

**14. BD (2) C 6 - C 12**	**/***. BD*(2) N 3 - C 7**	**9.45**	**0.21**	**0.041**
14. BD (2) C 6 - C 12	/***. BD*(2) C 6 - C 12	1.55	0.30	0.020
14. BD (2) C 6 - C 12	/***. BD*(1) C 8 - C 9	4.46	0.62	0.050
14. BD (2) C 6 - C 12	/***. BD*(1) C 9 - H 35	1.69	0.68	0.032

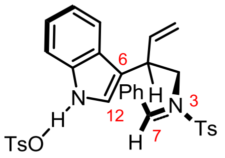

INT1-C

NATURAL POPULATIONS: Natural atomic orbital occupancies

NAO	Atom	No	lang	Type(AO)	Occupancy	Energy
53	N	3	S	Cor(1S)	1.99922	-14.32894
54	N	3	S	Val(2S)	1.29595	-0.63975
55	N	3	S	Ryd(4S)	0.00433	1.48945
56	N	3	S	Ryd(3S)	0.00086	1.05736
57	N	3	S	Ryd(5S)	0.00000	35.26012
58	N	3	px	Val(2p)	1.45626	-0.33948
59	N	3	px	Ryd(4p)	0.00577	0.86152
60	N	3	px	Ryd(3p)	0.00102	0.82998
61	N	3	px	Ryd(5p)	0.00007	4.06865
62	N	3	py	Val(2p)	1.34134	-0.34144
63	N	3	py	Ryd(4p)	0.00658	1.11862
64	N	3	py	Ryd(3p)	0.00100	0.89875
65	N	3	py	Ryd(5p)	0.00008	4.03237
66	N	3	pz	Val(2p)	1.44884	-0.33240
67	N	3	pz	Ryd(4p)	0.00621	0.88956
68	N	3	pz	Ryd(3p)	0.00091	0.82316
69	N	3	pz	Ryd(5p)	0.00006	4.03103
70	N	3	dxy	Ryd(3d)	0.00051	2.36027
71	N	3	dxz	Ryd(3d)	0.00071	2.75343
72	N	3	dyz	Ryd(3d)	0.00072	2.49262
73	N	3	dx2y2	Ryd(3d)	0.00092	2.59343
74	N	3	dz2	Ryd(3d)	0.00065	2.26327
119	C	6	S	Cor(1S)	1.99897	-10.08080
120	C	6	S	Val(2S)	0.86415	-0.12191
121	C	6	S	Ryd(3S)	0.00172	1.01628
122	C	6	S	Ryd(4S)	0.00058	2.07545
123	C	6	S	Ryd(5S)	0.00005	22.58792
124	C	6	px	Val(2p)	1.06705	-0.06215
125	C	6	px	Ryd(4p)	0.00470	1.21362

126	C	6	px	Ryd(3p)	0.00073	1.09730
127	C	6	px	Ryd(5p)	0.00017	3.12967
128	C	6	py	Val(2p)	1.06372	-0.06093
129	C	6	py	Ryd(3p)	0.00521	0.91352
130	C	6	py	Ryd(4p)	0.00096	0.95174
131	C	6	py	Ryd(5p)	0.00020	3.33192
132	C	6	pz	Val(2p)	1.09402	-0.09469
133	C	6	pz	Ryd(3p)	0.00453	0.77898
134	C	6	pz	Ryd(4p)	0.00097	1.04919
135	C	6	pz	Ryd(5p)	0.00009	2.89469
136	C	6	dxy	Ryd(3d)	0.00082	2.59929
137	C	6	dxz	Ryd(3d)	0.00082	2.18286
138	C	6	dyz	Ryd(3d)	0.00078	2.02099
139	C	6	dx2y2	Ryd(3d)	0.00073	2.41967
140	C	6	dz2	Ryd(3d)	0.00056	2.00063
141	C	7	S	Cor(1S)	1.99908	-10.19401
142	C	7	S	Val(2S)	0.91649	-0.21970
143	C	7	S	Ryd(3S)	0.00416	0.99467
144	C	7	S	Ryd(4S)	0.00108	2.72701
145	C	7	S	Ryd(5S)	0.00003	22.72699
146	C	7	px	Val(2p)	0.80719	-0.10479
147	C	7	px	Ryd(3p)	0.00600	0.77200
148	C	7	px	Ryd(4p)	0.00120	0.97728
149	C	7	px	Ryd(5p)	0.00012	3.02127
150	C	7	py	Val(2p)	1.17039	-0.12425
151	C	7	py	Ryd(4p)	0.00840	0.94719
152	C	7	py	Ryd(3p)	0.00188	0.68749
153	C	7	py	Ryd(5p)	0.00011	3.14144
154	C	7	pz	Val(2p)	0.83877	-0.11282
155	C	7	pz	Ryd(3p)	0.00361	0.66136
156	C	7	pz	Ryd(4p)	0.00130	0.80099
157	C	7	pz	Ryd(5p)	0.00011	2.93271
158	C	7	dxy	Ryd(3d)	0.00057	2.09264
159	C	7	dxz	Ryd(3d)	0.00079	2.29918
160	C	7	dyz	Ryd(3d)	0.00054	2.11100
161	C	7	dx2y2	Ryd(3d)	0.00129	2.16273
162	C	7	dz2	Ryd(3d)	0.00187	1.85754
251	C	12	S	Cor(1S)	1.99908	-10.11514
252	C	12	S	Val(2S)	0.92149	-0.16326
253	C	12	S	Ryd(3S)	0.00165	0.96103
254	C	12	S	Ryd(4S)	0.00005	1.00379
255	C	12	S	Ryd(5S)	0.00002	23.03267

256	C	12	px	Val(2p)	0.89500	-0.04437
257	C	12	px	Ryd(3p)	0.00579	0.81781
258	C	12	px	Ryd(4p)	0.00012	1.92269
259	C	12	px	Ryd(5p)	0.00010	1.92995
260	C	12	py	Val(2p)	1.16208	-0.07596
261	C	12	py	Ryd(3p)	0.00479	1.09883
262	C	12	py	Ryd(4p)	0.00017	1.74853
263	C	12	py	Ryd(5p)	0.00006	1.90847
264	C	12	pz	Val(2p)	0.97851	-0.09907
265	C	12	pz	Ryd(3p)	0.00349	0.76737
266	C	12	pz	Ryd(4p)	0.00008	1.78399
267	C	12	pz	Ryd(5p)	0.00009	1.79648
268	C	12	dxy	Ryd(3d)	0.00081	2.45515
269	C	12	dxz	Ryd(3d)	0.00120	2.02845
270	C	12	dyz	Ryd(3d)	0.00089	1.97878
271	C	12	dx2y2	Ryd(3d)	0.00117	2.32891
272	C	12	dz2	Ryd(3d)	0.00099	1.91198

```
------------------------------------------------------------

NATURAL BOND ORBITAL ANALYSIS:
      (Occupancy)   Bond orbital/ Coefficients/ Hybrids
------------------------------------------------------------
```

14. (1.74544) BD (2) C   6 - C 12

```
( 54.24%)   0.7365* C   6 s(  0.04%)p99.99( 99.89%)d 1.76(  0.07%)
                        0.0000  0.0084  0.0176  0.0003  0.0019
                        0.3538  0.0071  0.0074 -0.0007  0.3043
                        0.0108 -0.0076  0.0008  0.8834  0.0158
                        0.0151 -0.0033  0.0007  0.0164 -0.0151
                        0.0116 -0.0065
( 45.76%)   0.6765* C  12 s(  0.05%)p99.99( 99.87%)d 1.74(  0.09%)
                       -0.0002 -0.0200  0.0095  0.0014  0.0005
                        0.3988  0.0106  0.0001 -0.0001  0.2864
                        0.0064 -0.0004  0.0004  0.8700  0.0212
                        0.0010  0.0020  0.0058 -0.0119  0.0226
                       -0.0116 -0.0061
```

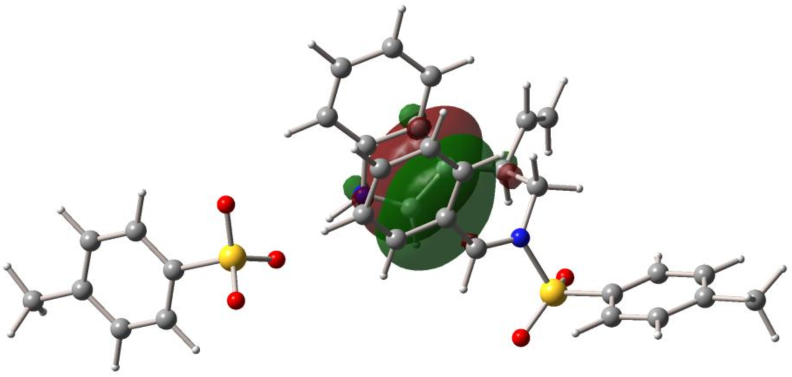

1051. (0.36742) BD*(2) N 3 - C 7

```
( 24.07%)   0.4906* N   3 s(  0.24%)p99.99( 99.73%)d 0.12(  0.03%)
                         -0.0001  0.0483  0.0099 -0.0025  0.0003
                         -0.7075  0.0104 -0.0036  0.0011  0.0223
                         -0.0093  0.0003 -0.0001 -0.7042  0.0081
                         -0.0038  0.0019  0.0036 -0.0038  0.0049
                         -0.0089  0.0127
( 75.93%)  -0.8714* C   7 s(  0.30%)p99.99( 99.41%)d 0.94(  0.28%)
                         -0.0001 -0.0546 -0.0072  0.0008  0.0006
                         -0.6502  0.0134 -0.0032  0.0051 -0.1386
                         -0.0107  0.0026 -0.0029 -0.7428  0.0046
                         -0.0065 -0.0007 -0.0044  0.0103 -0.0017
                          0.0272 -0.0444
```

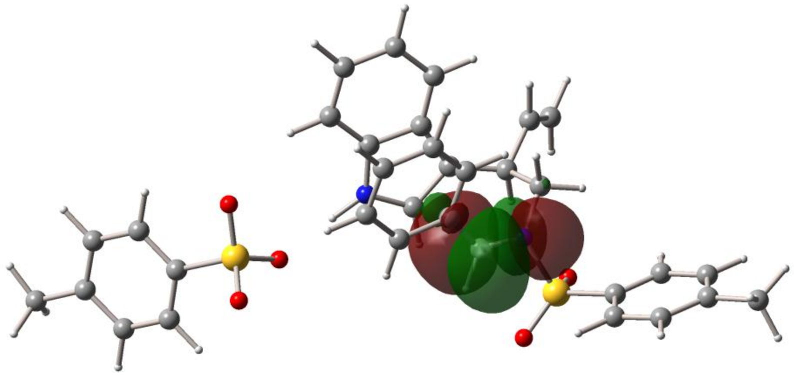

Second Order Perturbation Theory Analysis of Fock Matrix in NBO Basis

Threshold for printing: 0.50 kcal/mol
(Intermolecular threshold: 0.05 kcal/mol)

Donor NBO (i)	Acceptor NBO (j)	E(2) kcal/mol	E(j)-E(i) a.u.	F(i,j) a.u.
14. BD (2) C 6 - C 12	/148. LP (1) C 14	42.51	0.17	0.098
14. BD (2) C 6 - C 12	/149. LP (1) C 19	0.86	0.13	0.012
14. BD (2) C 6 - C 12	/181. RY*(1) N 2	1.11	0.79	0.028
14. BD (2) C 6 - C 12	/186. RY*(6) N 2	0.51	2.23	0.032
14. BD (2) C 6 - C 12	/387. RY*(3) C 14	0.52	1.39	0.026
14. BD (2) C 6 - C 12	/388. RY*(4) C 14	0.66	1.05	0.025
14. BD (2) C 6 - C 12	/***. BD*(2) N 2 - C 13	8.94	0.25	0.048
**14. BD (2) C 6 - C 12**	**/***. BD*(2) N 3 - C 7**	**5.69**	**0.21**	**0.031**
14. BD (2) C 6 - C 12	/***. BD*(2) C 6 - C 12	1.55	0.30	0.020
14. BD (2) C 6 - C 12	/***. BD*(1) C 8 - C 9	4.86	0.61	0.051
14. BD (2) C 6 - C 12	/***. BD*(1) C 9 - C 10	2.08	0.70	0.036

3D structures of the calculated stationary points

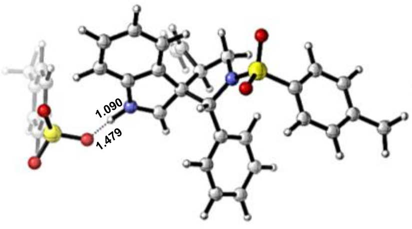

A•TsOH
0.0 (0.0)

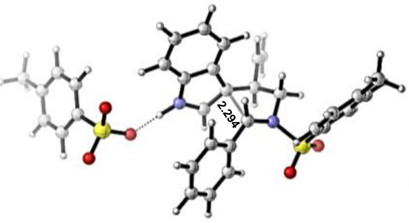

TS1-A
21.2 (21.1)

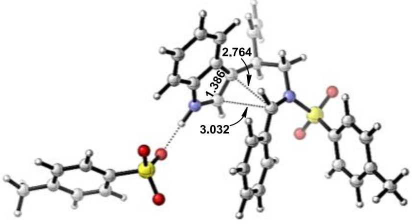

INT1-A
17.6 (18.0)

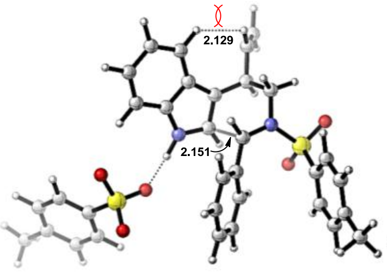

TS2-A
23.3 (22.1)

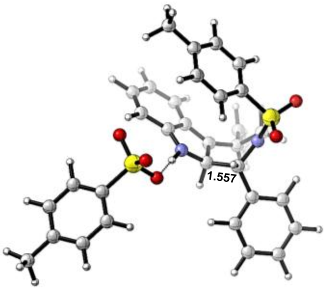

INT2-A
12.9 (12.3)

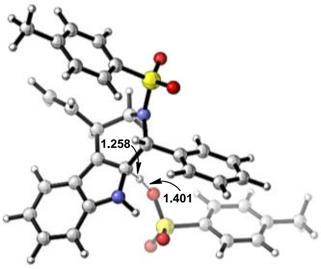

TS3-A
15.3 (13.6)

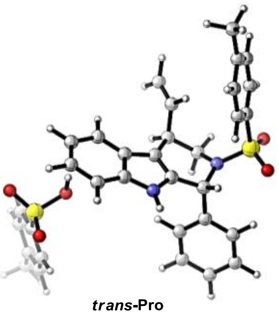

***trans*-Pro**
-8.2 (-6.4)

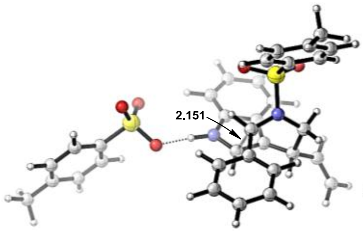

TS2-A'
26.9 (27.2)

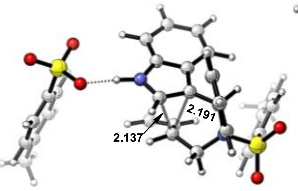

TS-allyl-A
24.4 (23.9)

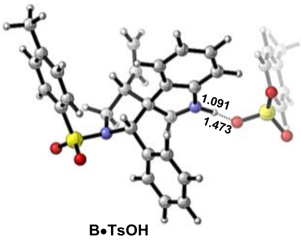

B•TsOH
-0.9 (-0.7)

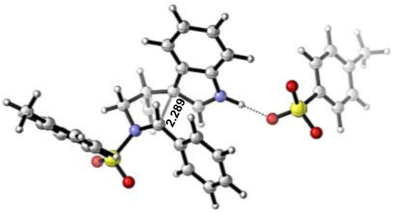

TS1-B
20.3 (21.1)

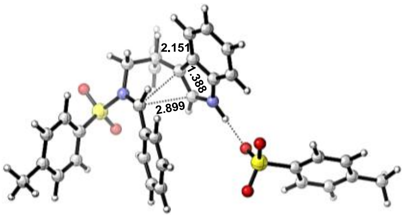

INT1-B
16.9 (17.9)

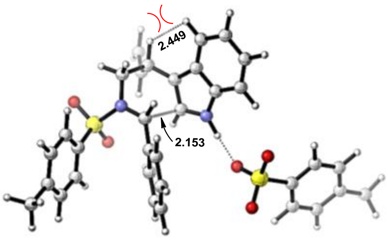

TS2-B
22.0 (20.8)

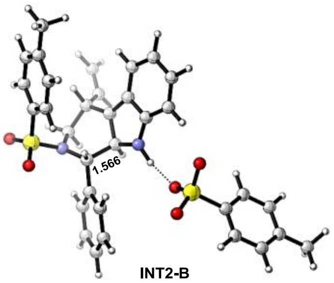

INT2-B
9.1 (8.9)

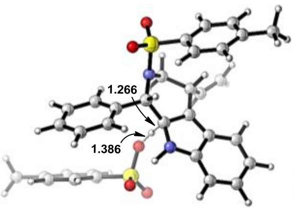

TS3-B
12.9 (11.1)

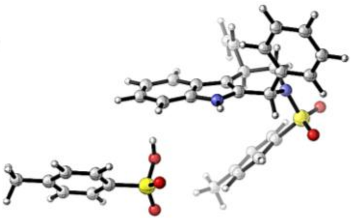

***cis*-Pro**
-10.1 (-9.4)

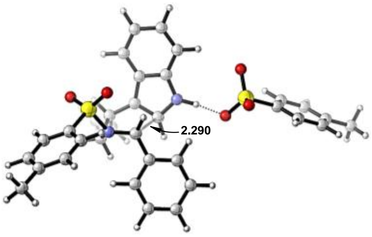

TS2-B'
23.0 (23.8)

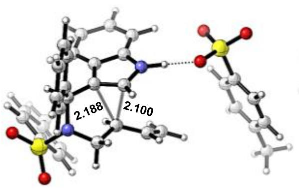

TS-allyl-B
25.6 (24.5)

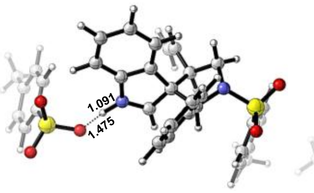

C•TsOH
1.5 (0.8)

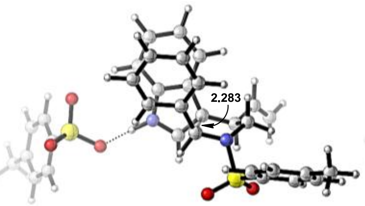

TS1-C
17.8 (17.9)

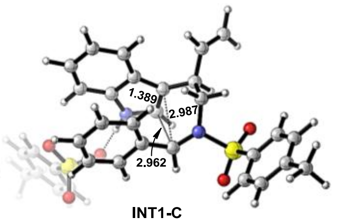

INT1-C
16.7 (15.8)

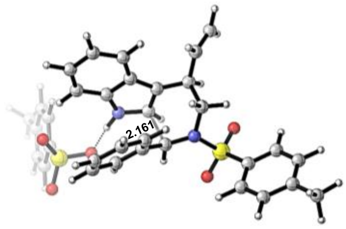

TS2-C
18.3 (17.9)

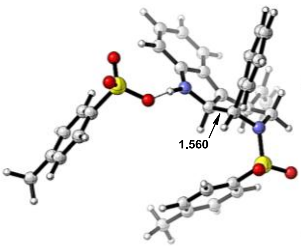

INT2-C
6.8 (7.0)

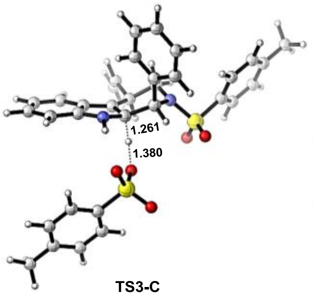

TS3-C
11.6 (10.0)

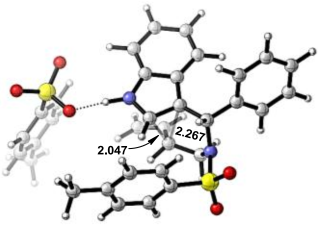

TS-allyl-C
27.6 (25.5)

Cartesian coordinates of the calculated stationary points

A•TsOH

SCF Done: E(RPBE1PBE) = -2556.87332002 a.u.
Zero-point correction = 0.588199 Hartree/Particle
Sum of electronic and thermal Free Energies = -2556.362833 a.u.

```
----------------------------------------------------------
S,0,-4.1189902349,1.5856867017,-0.2654942095
N,0,1.7115019999,0.1482262052,-0.7463044556
N,0,-2.711934815,0.8915419424,0.2461443268
O,0,-4.2906603102,2.8000840151,0.5211238261
O,0,-4.0566286729,1.6400154107,-1.7191736026
C,0,-0.432480997,0.5112402054,0.0088547608
C,0,-1.8076805827,0.1798800369,-0.6518767258
C,0,-2.1565285988,1.1691823593,1.5699491415
C,0,-0.7927522478,0.4741181887,1.5355693074
C,0,0.2226501347,1.0868308569,2.4444616652
C,0,0.8988747122,0.4000058988,3.36052314
C,0,0.6737523114,-0.4456441068,-0.2747334865
C,0,1.4739644854,1.5327608668,-0.9027637574
C,0,0.1761769606,1.8069371258,-0.4701554495
C,0,-0.3012684143,3.1045621492,-0.5514546213
C,0,0.5502707773,4.0892210567,-1.0575910191
C,0,1.8466131972,3.7881733587,-1.4728905003
C,0,2.33910099,2.4867079202,-1.4051963066
C,0,-2.0869007506,-1.3039333714,-0.7903675599
C,0,-1.6595304655,-1.9598457439,-1.946569736
C,0,-1.8680172307,-3.324973712,-2.1026996677
C,0,-2.5137330288,-4.0496812802,-1.1049253431
C,0,-2.9545551567,-3.3997589787,0.0426998578
C,0,-2.744492807,-2.0326858658,0.1986938878
C,0,-5.43440622,0.4789662289,0.1827826303
C,0,-5.903894229,-0.4422106609,-0.7457396245
C,0,-6.9149101306,-1.3188745516,-0.3731898631
C,0,-7.4618171112,-1.290133727,0.9111698851
C,0,-6.9712498897,-0.3492562091,1.8232742907
C,0,-5.96596405,0.5375832675,1.4688594476
C,0,-8.5692600841,-2.2212529914,1.3006001672
H,0,-1.8430679591,0.6336568619,-1.6440134121
H,0,-2.7756915093,0.7580362455,2.37283407
H,0,-2.0518493758,2.246707079,1.7266403381
H,0,-0.9206851816,-0.5804398517,1.7988757984
H,0,0.3936325662,2.1561851526,2.3305740155
H,0,0.7472772616,-0.6679801097,3.5003371541
H,0,1.6283446633,0.8842975393,4.002751272
H,0,0.6694486088,-1.5113750424,-0.0788807403
H,0,-1.3073098361,3.3632945458,-0.2408981627
H,0,0.1921626143,5.110982112,-1.1296512232
H,0,2.4815107047,4.5777220458,-1.8608585622
H,0,3.3403761163,2.2174453064,-1.7290312488
H,0,-1.1658853864,-1.3956897518,-2.7339983841
H,0,-1.5332014821,-3.8214717954,-3.007999841
H,0,-2.6815720063,-5.1151052908,-1.2269406592
H,0,-3.4704727944,-3.9560525308,0.8191320198
H,0,-3.1106972146,-1.5306616181,1.0889436581
H,0,-5.493223863,-0.4683253324,-1.7487010289
```

H,0,-7.2876449546,-2.0367655704,-1.0976587681
H,0,-7.390231212,-0.3040311863,2.8244307262
H,0,-5.6095624678,1.2765541068,2.1781017258
H,0,-9.5211591204,-1.682112583,1.3643044273
H,0,-8.3861623488,-2.6673061778,2.2824952454
H,0,-8.6896123412,-3.0260098931,0.5720987498
H,0,2.6474748389,-0.3585553274,-0.9835842372
S,0,5.1916006292,-0.6268590647,-1.6517452892
O,0,3.8418052292,-1.1845879294,-1.2660898442
O,0,5.0811792665,0.7847958929,-2.0594964103
O,0,5.8858789959,-1.5106922875,-2.5920687901
C,0,6.1398933406,-0.6465909148,-0.1340943434
C,0,6.125711483,0.4576062864,0.7088902743
C,0,6.8544759067,-1.7879595241,0.2144541271
C,0,6.8310643507,0.414567092,1.9061878371
H,0,5.5766104728,1.3491486752,0.4248207688
C,0,7.5523703352,-1.8190519591,1.4140763559
H,0,6.8684733582,-2.6419674343,-0.4542515906
C,0,7.5551666543,-0.719895603,2.2780621751
H,0,6.8225228754,1.2810944701,2.561536467
H,0,8.1111032667,-2.7116303227,1.6822258439
C,0,8.3378252881,-0.7530144093,3.5568393057
H,0,8.2540743187,-1.725569291,4.0501035628
H,0,9.4026923905,-0.5776031029,3.3655567506
H,0,7.997043759,0.0169148739,4.2533042056

TS1-A
SCF Done: E(RPBE1PBE) = -2556.83797159 a.u.
Zero-point correction = 0.586425 Hartree/Particle
Sum of electronic and thermal Free Energies = -2556.329079 a.u.
Imaginary Freq. = -173.0129 cm^{-1}

N,0,0.1111374176,1.4319678161,-0.8315693446
C,0,1.71184711,1.2522753208,0.7474461924
C,0,0.5742604914,1.9545123482,0.2948645871
C,0,0.8686875749,0.3164791265,-1.1703160887
C,0,1.8591457194,0.1447157524,-0.1819634574
C,0,2.768059102,-0.9076750652,-0.3090774506
C,0,2.6484889035,-1.7588240001,-1.4020591122
C,0,1.6531643989,-1.5671712061,-2.3688024573
C,0,0.7462720343,-0.5187820518,-2.2727490389
H,0,0.1143223374,2.8284798697,0.736946852
H,0,3.5483413018,-1.0718543634,0.4268874738
H,0,3.3398395898,-2.5887522563,-1.5090681494
H,0,1.591132571,-2.2479816465,-3.2117716139
H,0,-0.0197661943,-0.3353922794,-3.0209535246
S,0,-2.4956380629,1.9709341055,-3.475834366
O,0,-1.9834353946,2.4472878036,-2.1490553197
O,0,-1.6118079173,0.9434314164,-4.0607049397
O,0,-2.8350376077,3.0847122796,-4.3723337381
C,0,-4.0409820151,1.143122071,-3.1005319877
C,0,-4.053405503,-0.2203233526,-2.8369280828
C,0,-5.2195853594,1.8799198528,-3.0303322119
C,0,-5.2500296534,-0.8459352577,-2.5025510253
H,0,-3.1337837024,-0.7917825084,-2.9050683553
C,0,-6.4070622709,1.2449028844,-2.6939336449

H,0,-5.2052391578,2.9424768561,-3.2490013321
C,0,-6.4435989163,-0.1270921767,-2.424893643
H,0,-5.2554125877,-1.9136086316,-2.3012981905
H,0,-7.3257405404,1.8235434626,-2.6436277023
C,0,-7.7379820217,-0.8035061694,-2.0826160063
H,0,-8.2677273557,-0.2683832175,-1.2884460792
H,0,-8.4056893307,-0.8332046612,-2.9509331193
H,0,-7.5751783157,-1.8321706436,-1.7522329132
H,0,-0.6969611853,1.8101642423,-1.3860812956
S,0,4.8712251497,4.2494777368,1.3264260357
N,0,3.7387574645,2.9917004412,1.0259253631
O,0,4.784382105,4.5099026272,2.749883104
O,0,4.6204622443,5.2964411634,0.3599267217
C,0,3.2287635909,2.6793180146,-0.2147081747
C,0,3.792524557,1.8407208543,1.9229191389
C,0,2.3217581656,1.4173143468,2.1088334224
C,0,2.2142347463,0.1924455433,2.9726663805
C,0,1.625720226,0.1821134167,4.1644222261
C,0,2.6651391605,3.6264963,-1.1406032175
C,0,2.6640743908,3.2715615014,-2.4999021485
C,0,2.0908175633,4.1118250396,-3.4430739794
C,0,1.4951886553,5.2999066092,-3.0356500747
C,0,1.468869966,5.6504611768,-1.6833870682
C,0,2.0450595986,4.8234127323,-0.7396564395
C,0,6.4310574283,3.4913430854,0.9732889569
C,0,6.8502914056,3.402081516,-0.3500652244
C,0,8.0654269885,2.7912674329,-0.6245547548
C,0,8.8628956143,2.2728499658,0.3996708636
C,0,8.4129954178,2.3834268638,1.7195267944
C,0,7.2009959829,2.987080345,2.0174505262
C,0,10.1792818333,1.625098954,0.1003721502
H,0,3.5929060537,1.7508526832,-0.6482192536
H,0,4.3746669103,1.0269977655,1.473726975
H,0,4.2435179597,2.1284606065,2.8716600622
H,0,1.8114262998,2.2451372022,2.6110359569
H,0,2.6549526937,-0.7226820877,2.5823721574
H,0,1.1688894085,1.0761591599,4.5828672417
H,0,1.5767439833,-0.7252635281,4.7593420633
H,0,3.1258419784,2.3382131925,-2.8076608636
H,0,2.0962196325,3.8345381222,-4.4915737348
H,0,1.0315831992,5.9510771833,-3.7697655921
H,0,0.9824843882,6.5688729783,-1.3721735037
H,0,1.999327758,5.0826529041,0.3115771573
H,0,6.2478410415,3.814688159,-1.1523318749
H,0,8.4041934707,2.7232405725,-1.6536268981
H,0,9.0244710798,1.9942305346,2.528206337
H,0,6.863572929,3.0785515563,3.0436817536
H,0,11.0034372955,2.2146900073,0.5160715433
H,0,10.2376119765,0.6292711632,0.5503212854
H,0,10.3417152775,1.5279720684,-0.9749332294

INT1-A
SCF Done: E(RPBE1PBE) = -2556.84434191 a.u.
Zero-point correction = 0.587922 Hartree/Particle
Sum of electronic and thermal Free Energies = -2556.334823 a.u.

S,0,4.111197621,0.0774170302,-1.4084764725
N,0,-1.2387275011,1.5042842282,-0.1704956094
N,0,3.0909809462,0.9238501279,-0.2221170368
O,0,3.2839715473,-0.3678391142,-2.5112242608
O,0,5.1730457579,1.0310880852,-1.6506135087
C,0,0.7204223016,2.5995163389,-0.3819644875
C,0,2.1256510899,0.3946704219,0.5130369016
C,0,3.2094194331,2.3861341273,-0.1868571091
C,0,2.0278626538,3.0184411388,-0.974586286
C,0,2.2218278877,4.5102092135,-1.030079554
C,0,2.4952066242,5.1765484362,-2.1472532113
C,0,-0.1671781178,1.7125937829,-0.9698273833
C,0,-1.0874331915,2.2394603488,0.9777390205
C,0,0.1355689435,2.9548328985,0.8862789863
C,0,0.5263757371,3.7590743768,1.9699089923
C,0,-0.2995599471,3.836649316,3.0800773431
C,0,-1.5085502861,3.1215805272,3.1419839301
C,0,-1.9178654092,2.3104707054,2.0964126718
C,0,1.5075271618,-0.8990418482,0.5583002863
C,0,1.4055151848,-1.8190347635,-0.5023965864
C,0,0.712261222,-3.0009815528,-0.322035015
C,0,0.1163602169,-3.2916699033,0.9047095788
C,0,0.1933462502,-2.3847973473,1.956791244
C,0,0.8655181131,-1.1892125982,1.7805720453
C,0,4.709724533,-1.2910742177,-0.4798865938
C,0,5.3769001524,-1.0581811278,0.7198168055
C,0,5.919415533,-2.1391668643,1.3940582994
C,0,5.8193490172,-3.4373932483,0.88094287
C,0,5.157474379,-3.6303064685,-0.3348630593
C,0,4.5946772231,-2.5666964385,-1.0230247717
C,0,6.3954967591,-4.6015382742,1.6233189781
H,0,1.8138941575,1.0598109842,1.3148168656
H,0,4.1651816333,2.6762101101,-0.6169376979
H,0,3.185279603,2.6960812865,0.8610072384
H,0,2.0799780962,2.6356285488,-1.999486035
H,0,2.1291617688,5.0525860437,-0.0911930793
H,0,2.5894380932,4.6701257404,-3.1053503637
H,0,2.6249036404,6.2549307518,-2.1416725765
H,0,-0.0901379577,1.219225406,-1.9296355678
H,0,1.4559823825,4.318822428,1.9469027304
H,0,-0.0089886139,4.4608227099,3.9196387672
H,0,-2.1292145896,3.2021218979,4.029039025
H,0,-2.8336689499,1.7282133383,2.1390956582
H,0,1.8165373618,-1.5858665998,-1.4749347463
H,0,0.6145487,-3.6959802288,-1.1489757647
H,0,-0.4345635474,-4.2181118955,1.0301130563
H,0,-0.2946199453,-2.5983323897,2.9011382727
H,0,0.9018331071,-0.4629609032,2.5864209275
H,0,5.4725656522,-0.0553193789,1.1232971939
H,0,6.4395331259,-1.9724791007,2.3320241548
H,0,5.0791817758,-4.6302474429,-0.7501490657
H,0,4.0837025517,-2.7244669166,-1.9656697181
H,0,6.8743582621,-5.3073958064,0.9396890933
H,0,5.603403057,-5.1468737962,2.1491528611
H,0,7.1281165561,-4.2797122948,2.3662342743
S,0,-4.0651908188,-1.0075863729,0.202497962
O,0,-3.2637369064,-0.2248429356,-0.7868144535
O,0,-3.9616949971,-0.4567371136,1.5682142425

O,0,-3.814933965,-2.4568487979,0.107023596
C,0,-5.7695059455,-0.7633344909,-0.3014021255
C,0,-6.6981863691,-0.2573515352,0.5961057727
C,0,-6.1589961774,-1.0936372147,-1.5979417669
C,0,-8.0211202709,-0.0838902792,0.1960152161
H,0,-6.3820600941,0.0016626407,1.6007436657
C,0,-7.4781104865,-0.91479058,-1.9857323161
H,0,-5.4321791661,-1.4856631883,-2.3021111704
C,0,-8.4328176921,-0.4080155791,-1.0956931339
H,0,-8.7439117888,0.3143705516,0.9029620717
H,0,-7.7755003327,-1.1716028108,-2.9991749545
C,0,-9.8599746611,-0.2359931274,-1.5247450881
H,0,-9.9272759034,0.3130593075,-2.4691401381
H,0,-10.3417886729,-1.2076209156,-1.681391583
H,0,-10.4383314865,0.3062564431,-0.7729890669
H,0,-2.0219210228,0.8570521692,-0.3755592409

TS2-A
SCF Done: E(RPBE1PBE) = -2556.83755150 a.u.
Zero-point correction = 0.587686 Hartree/Particle
Sum of electronic and thermal Free Energies = -2556.325753 a.u.
Imaginary Freq. = -249.6795 cm^{-1}

S,0,3.8647843707,-0.1877533509,-1.5525533538
N,0,-0.8142486363,1.4327830325,-0.1084341046
N,0,3.0416900752,0.9195548586,-0.4930366943
O,0,2.9198169148,-0.7626464969,-2.4930364662
O,0,4.978590662,0.5906789024,-2.0621294921
C,0,0.9044167832,2.7263047047,-0.8414721602
C,0,1.9220238882,0.6405320606,0.2388432845
C,0,3.3247273445,2.3317153594,-0.6722309949
C,0,2.1954211913,3.0045024901,-1.524272372
C,0,2.5397807891,4.4511700265,-1.7332879583
C,0,2.6193121625,5.0153624796,-2.9335423448
C,0,0.2946586133,1.4488538138,-0.9116400143
C,0,-0.8960759261,2.6156283188,0.5514917923
C,0,0.1756579373,3.4591846944,0.1315186196
C,0,0.2941456725,4.7443933435,0.7083067873
C,0,-0.6352432967,5.1399835545,1.641881586
C,0,-1.6914670417,4.284762406,2.0344995109
C,0,-1.8379472829,3.0205163133,1.5077807834
C,0,1.4033879445,-0.681975401,0.6195601848
C,0,1.0802695891,-1.7232443498,-0.2594598756
C,0,0.5622467044,-2.910795697,0.2329345993
C,0,0.354885975,-3.0793871248,1.5987147966
C,0,0.6545253123,-2.0454191493,2.4779417094
C,0,1.15877759,-0.848788888,1.9909461034
C,0,4.457354212,-1.428179856,-0.4477250327
C,0,5.2236950472,-1.0514962332,0.6510610508
C,0,5.7613222415,-2.0431258809,1.4557049424
C,0,5.5557928942,-3.3972678442,1.1711365141
C,0,4.7950741701,-3.7386786644,0.0496314525
C,0,4.2383140867,-2.764030396,-0.7645591516
C,0,6.1230456406,-4.46253143,2.0566011553
H,0,1.7949652736,1.3727040426,1.0332356524
H,0,4.2871237145,2.4532600221,-1.1638944091

H,0,3.3631020593,2.7998583861,0.3150395526
H,0,2.192028484,2.5073147021,-2.4988657546
H,0,2.7635482507,5.0407051506,-0.846616601
H,0,2.4149638626,4.4538272112,-3.8422547305
H,0,2.8882260204,6.0613847782,-3.0467913539
H,0,0.3929241476,0.7302969127,-1.7141719622
H,0,1.0910790561,5.4165108944,0.4161928016
H,0,-0.5626182847,6.1272006324,2.0863366438
H,0,-2.4046271414,4.6367744185,2.7735964543
H,0,-2.6398975715,2.3500249975,1.8047716608
H,0,1.2073076964,-1.5980726997,-1.3269711642
H,0,0.3005097998,-3.7052436036,-0.4581323936
H,0,-0.0629385294,-4.0078333681,1.9742770516
H,0,0.4749533278,-2.1608707868,3.5415344866
H,0,1.3710879156,-0.0337985627,2.6759381929
H,0,5.3972148678,-0.0050019147,0.8804333643
H,0,6.356401743,-1.7617475763,2.3188769913
H,0,4.6316336959,-4.7852237068,-0.1889876858
H,0,3.6464821049,-3.0362681325,-1.6307325647
H,0,6.5436211755,-5.2827234903,1.4684802121
H,0,5.3376810678,-4.8890503731,2.6909927273
H,0,6.9020979237,-4.0657097524,2.7108952846
S,0,-3.7383612888,-0.7123585255,1.1006047717
O,0,-2.7257701734,-0.496293713,0.0218515722
O,0,-3.8745936715,0.4649069156,1.9825009723
O,0,-3.5423801683,-1.9860772041,1.8124823311
C,0,-5.299792387,-0.8603082438,0.2310518885
C,0,-6.3375154892,0.0187501029,0.5028564491
C,0,-5.4672560569,-1.8691174024,-0.7156307178
C,0,-7.5488342436,-0.11452034,-0.1719592785
H,0,-6.1939217731,0.800968849,1.2401725096
C,0,-6.6769136997,-1.9909707312,-1.3820389384
H,0,-4.6547689146,-2.5559875831,-0.929919794
C,0,-7.7396495001,-1.1171794269,-1.1212077339
H,0,-8.3585471926,0.5766114731,0.0456031151
H,0,-6.8025273806,-2.7796072566,-2.119318023
C,0,-9.0472454547,-1.2696083351,-1.8405971214
H,0,-8.9062803061,-1.2669933616,-2.9262171973
H,0,-9.5302528197,-2.2181517873,-1.5813587699
H,0,-9.7376617795,-0.4619641106,-1.5860655472
H,0,-1.509585536,0.6635312583,-0.0360382017

INT2-A
SCF Done: E(RPBE1PBE) = -2556.85467878 a.u.
Zero-point correction = 0.589153 Hartree/Particle
Sum of electronic and thermal Free Energies = -2556.342287 a.u.

S,0,3.1758392614,-0.1596265073,-1.9665334635
N,0,-0.0816044448,-0.0276949441,0.7443637719
N,0,2.9423002945,0.7478552576,-0.592349092
O,0,2.4874395275,0.536172263,-3.042797775
O,0,4.6091910456,-0.4053404238,-2.0274025237
C,0,1.921302307,0.3058249747,1.8445461394
C,0,1.6799191045,1.4485861562,-0.4029269241
C,0,3.9053241899,0.6144116645,0.5044840724
C,0,3.2730358415,0.9089697846,1.8978314345

C,0,4.1986360013,0.4348601383,2.9752648189
C,0,4.7155032492,1.234749241,3.901703336
C,0,0.976973037,0.9378014487,0.8894743225
C,0,0.0920120228,-1.0063213577,1.6139715215
C,0,1.3529135429,-0.8469659284,2.3188214526
C,0,1.7478985769,-1.829634544,3.2811520225
C,0,0.9125235787,-2.8758849969,3.5103297637
C,0,-0.3346682996,-3.0041174851,2.8110540379
C,0,-0.7653226932,-2.1023166992,1.8828202497
C,0,1.8390949453,2.9657714675,-0.4156750368
C,0,0.7396213858,3.7803914451,-0.133784498
C,0,0.8520879353,5.1641670392,-0.1845403389
C,0,2.067244331,5.7557563601,-0.5171799139
C,0,3.1608052601,4.951270063,-0.8119722989
C,0,3.0458672321,3.5642314757,-0.7676554751
C,0,2.3607473504,-1.7206917356,-1.7407357438
C,0,3.0411583504,-2.7686166193,-1.129220182
C,0,2.3824724289,-3.9759348606,-0.9342928165
C,0,1.0598246672,-4.1528449504,-1.3465353722
C,0,0.4049018577,-3.0845711471,-1.9681654946
C,0,1.0429436679,-1.8700671749,-2.1675768224
C,0,0.3584105027,-5.4625028494,-1.1546220171
H,0,1.033910827,1.1906297135,-1.2465434958
H,0,4.7499128876,1.2959184628,0.3711725751
H,0,4.2940498252,-0.4070199671,0.5166825482
H,0,3.1492981295,1.9949886205,1.9850353322
H,0,4.4736872469,-0.6182919448,2.9506081164
H,0,4.4720270342,2.2939027894,3.9417547675
H,0,5.4015166332,0.8547472424,4.6527854782
H,0,0.5494191788,1.8087866988,1.4133067653
H,0,2.6788039103,-1.7246889654,3.8241856474
H,0,1.1758534506,-3.6334300222,4.2402712985
H,0,-0.9596472733,-3.8623892254,3.039414772
H,0,-1.7041602315,-2.2015258873,1.3454650868
H,0,-0.222691179,3.3388515371,0.1114424891
H,0,-0.0148258384,5.7806562457,0.0315630564
H,0,2.1565841563,6.8368219411,-0.5538974858
H,0,4.1106903986,5.4006538619,-1.0843936361
H,0,3.9005983628,2.9472361291,-1.0209263295
H,0,4.0766429371,-2.651357236,-0.8290076293
H,0,2.9103928762,-4.7993789584,-0.462346334
H,0,-0.6214544823,-3.2047195989,-2.3020579806
H,0,0.5286723807,-1.0542682112,-2.6633281184
H,0,0.2166570358,-5.9689352273,-2.1158258673
H,0,-0.6349410121,-5.316697198,-0.7199319868
H,0,0.9282738921,-6.129924636,-0.5044729506
S,0,-3.3796411544,-0.5133420785,-0.964471536
O,0,-2.3477750042,0.4968700979,-0.5572019995
O,0,-3.1871082806,-1.8002970218,-0.2658440965
O,0,-3.5091001539,-0.6172773203,-2.4248093128
C,0,-4.9282594238,0.1530903436,-0.3590286572
C,0,-5.599705611,-0.4806577825,0.6761027004
C,0,-5.4465659922,1.3169690044,-0.9230683348
C,0,-6.7953009074,0.0531981397,1.1501010012
H,0,-5.1888961719,-1.3893553091,1.102333269
C,0,-6.6370010706,1.8394482569,-0.4411978003
H,0,-4.9245464837,1.8084037009,-1.737729733
C,0,-7.331946155,1.2177903807,0.6033282285

H,0,-7.3198123946,-0.4490638112,1.9583113816
H,0,-7.0393920605,2.746200726,-0.8852074422
C,0,-8.6219949986,1.7927025605,1.1081635844
H,0,-8.4893492716,2.8260847989,1.4449087611
H,0,-9.3817790156,1.8064420166,0.3193723895
H,0,-9.0159343813,1.2111517136,1.944880602
H,0,-0.9714751892,0.1468853062,0.2124630655

TS3-A
SCF Done: E(RPBE1PBE) = -2556.84715354 a.u.
Zero-point correction = 0.583751 Hartree/Particle
Sum of electronic and thermal Free Energies = -2556.338425 a.u.
Imaginary Freq. = -588.4162 cm^{-1}

S,0,-1.4023571869,-2.1905272737,0.4571797352
N,0,2.1191528242,1.2088226122,-1.0354714874
N,0,-0.1795205432,-1.1138531113,0.7670543549
O,0,-0.9146382071,-3.0563470788,-0.605031938
O,0,-1.8138111832,-2.7373991048,1.7420298372
C,0,0.6234361307,1.5615436701,0.6531881616
C,0,0.7838632577,-0.8150377763,-0.3014905535
C,0,-0.1795804727,-0.321370633,1.9952491564
C,0,-0.3214412329,1.1908476425,1.7436344443
C,0,-1.7246269125,1.6344558984,1.4228724695
C,0,-2.4521215365,2.4100161066,2.2203687613
C,0,1.4047948751,0.5549637364,0.0045256104
C,0,1.9556962552,2.5397745643,-0.8964188775
C,0,0.9913198074,2.802156808,0.1254188809
C,0,0.6410205061,4.1369961431,0.4418862482
C,0,1.2595200597,5.1476410672,-0.2435702091
C,0,2.2382234075,4.8683420348,-1.2358015411
C,0,2.607104775,3.5885591915,-1.5695674382
C,0,1.8334410151,-1.8880962439,-0.5146567795
C,0,2.3543695451,-2.6230694535,0.5477839004
C,0,3.328410157,-3.5871212463,0.320479624
C,0,3.7975948198,-3.8207587651,-0.9692352813
C,0,3.2842776303,-3.0866269725,-2.0323822331
C,0,2.3038682569,-2.1261212599,-1.8039281635
C,0,-2.7967660357,-1.297984037,-0.1953277484
C,0,-3.8775405372,-1.0107218364,0.6303401851
C,0,-4.9537483442,-0.301673559,0.112992048
C,0,-4.9651202204,0.1297420032,-1.2151740655
C,0,-3.8662622143,-0.1772349403,-2.0244785585
C,0,-2.7864826907,-0.8923205653,-1.5280611799
C,0,-6.1417478654,0.8698564934,-1.7737503972
H,0,0.2390132217,-0.6877008878,-1.24390063
H,0,-0.0070760284,1.7012368706,2.6633822905
H,0,-2.1388804939,1.2817846268,0.480968098
H,0,-2.0621656493,2.7846307846,3.1641386894
H,0,-3.4672081815,2.6911270678,1.9554980961
H,0,2.2964061132,0.4450663387,0.8847263626
H,0,-0.0986409881,4.3428728526,1.2074720008
H,0,1.0140198384,6.1817784928,-0.0276590758
H,0,2.7118905693,5.7019082981,-1.7451118469
H,0,3.3608484796,3.3886965442,-2.3227433928
H,0,1.9877418774,-2.4533663193,1.5550162063

H,0,3.7254708397,-4.1572337387,1.1546789133
H,0,4.5579223809,-4.5757458604,-1.1438521682
H,0,3.6385625448,-3.2674753093,-3.0426158141
H,0,1.8880246385,-1.5689597958,-2.6398832623
H,0,-3.8779232467,-1.3430866552,1.6621545217
H,0,-5.8012240228,-0.0796440822,0.7549361886
H,0,-3.8619675775,0.140842686,-3.0629983297
H,0,-1.9542878422,-1.1450706316,-2.1761251353
H,0,-6.8408276069,0.173430363,-2.2512604626
H,0,-5.8336466442,1.5923228826,-2.5337426267
H,0,-6.6895155991,1.398605891,-0.9901801985
H,0,2.9872653636,0.8169029574,-1.389500458
H,0,0.7618915897,-0.5034367191,2.525643254
H,0,-0.9821608616,-0.6799293195,2.6390120916
S,0,4.8015779002,0.5968822591,1.2891854579
O,0,5.4564534672,1.7252328885,1.9564032057
O,0,4.8626059839,0.6108227485,-0.1828805869
O,0,3.3710991975,0.4337404882,1.783663679
C,0,5.6277291387,-0.8902115786,1.8284465438
C,0,6.4994773545,-1.5387305625,0.9665110182
C,0,5.4375934582,-1.3562915241,3.1270158398
C,0,7.1865928168,-2.6647422398,1.4098745717
H,0,6.6306745999,-1.1721277446,-0.0454080712
C,0,6.1255672281,-2.4819802339,3.553286461
H,0,4.7517939175,-0.8476936537,3.796556495
C,0,7.0114093181,-3.1549133368,2.7033423624
H,0,7.8668484881,-3.1737507038,0.7329030832
H,0,5.972261571,-2.8491593192,4.5645396032
C,0,7.7455913706,-4.3724636397,3.1800159094
H,0,8.3559300762,-4.1445949534,4.0600574514
H,0,7.0475009,-5.164842524,3.4698935717
H,0,8.4046783488,-4.768484852,2.4041304171

***trans*-Pro**
SCF Done: E(RPBE1PBE) = -2556.88343294 a.u.
Zero-point correction = 0.588114 Hartree/Particle
Sum of electronic and thermal Free Energies = -2556.375870 a.u.

S,4.34579,1.63783,0.58005
N,0.62894,-0.16526,-2.12954
N,2.71598,1.41241,0.47605
O,4.72181,2.40728,-0.6013
O,4.61957,2.15074,1.91503
C,0.8655,-0.6097,0.06068
C,2.02455,1.48459,-0.81911
C,1.92028,0.98502,1.62424
C,1.32599,-0.43121,1.47639
C,2.27326,-1.52465,1.88938
C,2.08234,-2.32485,2.93505
C,1.20411,0.2443,-0.95374
C,-0.09263,-1.30744,-1.90027
C,0.03628,-1.62509,-0.52198
C,-0.62608,-2.75485,-0.02216
C,-1.38581,-3.52765,-0.89203
C,-1.49347,-3.19452,-2.25596
C,-0.8502,-2.08323,-2.77649

```
C,1.20714,2.76946,-0.93561
C,-0.17169,2.787,-0.73151
C,-0.88209,3.98225,-0.81435
C,-0.22096,5.16912,-1.10689
C,1.15573,5.15722,-1.31779
C,1.86443,3.9656,-1.23262
C,5.16787,0.06327,0.4557
C,5.73284,-0.5074,1.58896
C,6.37229,-1.73672,1.48029
C,6.44872,-2.40672,0.25837
C,5.87421,-1.80546,-0.86771
C,5.2389,-0.5769,-0.77954
C,7.11552,-3.74414,0.14993
H,2.78952,1.50733,-1.59993
H,0.46062,-0.46954,2.15037
H,3.15711,-1.65308,1.26644
H,1.20404,-2.2305,3.57066
H,2.79684,-3.1021,3.19104
H,-2.81292,-1.92787,-0.2904
H,-0.5438,-3.02563,1.02588
H,-1.89693,-4.41024,-0.51973
H,-2.09797,-3.81615,-2.90839
H,-0.94076,-1.82074,-3.8257
H,-0.6995,1.86387,-0.51005
H,-1.95539,3.9814,-0.65062
H,-0.77478,6.10047,-1.17384
H,1.6792,6.0796,-1.55033
H,2.93916,3.95736,-1.38677
H,5.67914,0.00814,2.54093
H,6.82236,-2.18048,2.36343
H,5.93527,-2.30449,-1.83051
H,4.8161,-0.11703,-1.66632
H,7.77413,-3.93254,1.00076
H,7.70363,-3.8213,-0.76868
H,6.36808,-4.5455,0.12419
H,0.70445,0.31113,-3.01592
H,1.09744,1.70118,1.72995
H,2.5333,1.06276,2.5223
S,-4.88338,-1.54552,-0.78192
O,-5.21574,-2.94655,-0.58271
O,-4.82777,-1.0249,-2.13539
O,-3.47959,-1.25111,-0.04307
C,-5.93356,-0.52234,0.19972
C,-6.29281,0.73469,-0.27058
C,-6.37892,-0.99149,1.43145
C,-7.11653,1.5314,0.51275
H,-5.93858,1.0814,-1.23498
C,-7.19941,-0.17972,2.19884
H,-6.09357,-1.97707,1.78289
C,-7.58221,1.09008,1.75378
H,-7.40263,2.51471,0.15175
H,-7.55088,-0.53839,3.16166
C,-8.49643,1.94416,2.57735
H,-9.54326,1.71212,2.34895
H,-8.3489,1.76969,3.64601
H,-8.34285,3.00608,2.37141
---------------------------------------------------------
```

TS2-A′

SCF Done: E(RPBE1PBE) = -2556.82846202 a.u.
Zero-point correction = 0.586737 Hartree/Particle
Sum of electronic and thermal Free Energies = -2556.319954 a.u.
Imaginary Freq. = -251.4552 cm^{-1}

S,0,3.4810074552,-0.8832641345,-1.3208934697
N,0,-0.0663176482,0.1343043513,1.0345234992
N,0,3.2769524611,0.6209905303,-0.4781879048
O,0,4.3326705435,-1.7134942759,-0.4915104514
O,0,2.1568977356,-1.3184213741,-1.7226597552
C,0,2.0252462628,0.4892299208,1.8531220412
C,0,2.0738014642,1.2584289448,-0.6351699346
C,0,4.1542447773,0.8943038518,0.654832271
C,0,3.3159514708,1.2230402768,1.940560249
C,0,4.1575338128,0.9482303887,3.1528925369
C,0,4.5569599993,1.8954074834,3.994954441
C,0,0.9188272202,1.0781278464,1.1926243806
C,0,0.392926803,-1.0559033951,1.4830009738
C,0,1.7208624859,-0.8806115059,1.9901612433
C,0,2.4168663037,-2.0093048686,2.4867499979
C,0,1.7834451464,-3.2259735251,2.4864853824
C,0,0.4641347808,-3.3690035342,1.9854947215
C,0,-0.2437159469,-2.3055584563,1.4749337928
C,0,1.9643008396,2.7072392548,-0.861717699
C,0,0.7113808137,3.1858436126,-1.276693843
C,0,0.5315332767,4.5256073831,-1.5751277465
C,0,1.6009135919,5.4122283546,-1.4664951694
C,0,2.8493824195,4.945743834,-1.0689913402
C,0,3.0351861667,3.6028729739,-0.7666613135
C,0,4.3859376471,-0.3888996234,-2.75640839
C,0,3.6894607991,0.0242358403,-3.8885227771
C,0,4.4095269298,0.4288669398,-5.0014067184
C,0,5.8084056446,0.4278096225,-4.9996009041
C,0,6.4756571285,0.0030829471,-3.8478148651
C,0,5.7758073073,-0.4060041208,-2.7216322767
C,0,6.5712001793,0.8437258584,-6.2189018764
H,0,1.3519373782,0.6684798614,-1.1945811079
H,0,4.8347932901,1.7230786954,0.4534615179
H,0,4.7558623678,0.000094498,0.8193889561
H,0,3.0867715549,2.2925977963,1.9183286702
H,0,4.4643806306,-0.0825787992,3.315864309
H,0,4.2743726926,2.9366938374,3.8585479659
H,0,5.1759606684,1.6581589467,4.855113957
H,0,0.6520917901,2.1243770953,1.2063879108
H,0,3.4270016743,-1.9187671329,2.8677742335
H,0,2.2973387265,-4.1012390595,2.8698480439
H,0,0.004344527,-4.3524872423,2.0005110139
H,0,-1.2481161577,-2.4071230992,1.074831199
H,0,-0.1229556482,2.4956163972,-1.364793877
H,0,-0.4421010088,4.8785579548,-1.8984200475
H,0,1.4621038943,6.4632187701,-1.6988520969
H,0,3.688535727,5.6300560756,-0.9982921075
H,0,4.0234339006,3.2648949143,-0.4819380777
H,0,2.6052426963,0.0186951753,-3.9057050135
H,0,3.8752119208,0.7485958096,-5.8908753978
H,0,7.5611917668,-0.0111934552,-3.8325906565

H,0,6.302176957,-0.7439088223,-1.8360301881
H,0,6.6140794166,0.0207169643,-6.9415696497
H,0,7.5981961579,1.1212602829,-5.9715940938
H,0,6.0904335473,1.6885327343,-6.7190705258
S,0,-3.4151824415,-0.2005660242,-0.6235249977
O,0,-2.4734047581,0.7835923362,0.0007134601
O,0,-3.2518243876,-1.5479030051,-0.0419985088
O,0,-3.3894662147,-0.1556667469,-2.0940456632
C,0,-5.0449501237,0.3658781264,-0.1365571206
C,0,-5.4145820628,0.3311519194,1.2066025603
C,0,-5.9290953293,0.8486699623,-1.090025587
C,0,-6.6691337771,0.7837080732,1.5855037288
H,0,-4.7257815182,-0.0528786185,1.9522004429
C,0,-7.1877194186,1.2977878787,-0.6977795005
H,0,-5.6323993929,0.8656853018,-2.1330153122
C,0,-7.577571603,1.2755413857,0.6403539765
H,0,-6.9529679267,0.7535295486,2.6343133061
H,0,-7.8781226076,1.671186526,-1.449071913
C,0,-8.9318941525,1.7617289441,1.0639863637
H,0,-8.849302464,2.6117171917,1.7498088013
H,0,-9.5262197985,2.0784374684,0.2039109243
H,0,-9.4869720947,0.9779949244,1.5898842647
H,0,-1.0129830006,0.3246967681,0.6421890514

TS-allyl-A
SCF Done: E(RPBE1PBE) = -2556.83387684 a.u.
Zero-point correction = 0.586920 Hartree/Particle
Sum of electronic and thermal Free Energies = -2556.323880 a.u.
Imaginary Freq. = -237.4856 cm^{-1}

S,0,-4.1823382829,0.6602937259,1.2452682887
N,0,-2.8219259393,-0.2722459536,1.0923352056
N,0,1.0251330243,-1.3711430934,-0.7955051295
O,0,-4.3154627737,0.9410805636,2.6668726656
O,0,-5.2233690087,-0.0384681347,0.5080016718
C,0,-1.688080547,-0.1662259382,1.984773227
C,0,-0.4056849608,0.0739484354,1.2305836026
C,0,-1.1886175875,-0.9924351524,-0.5162877199
C,0,-2.61008748,-1.169941545,-0.0419460766
C,0,-2.9370421605,-2.6278400264,0.2661858179
C,0,-2.6932362461,-3.591959865,-0.7142658764
C,0,-3.0001398601,-4.9266157096,-0.4818946065
C,0,-3.5513854652,-5.3145489378,0.7364835366
C,0,-3.7982100003,-4.3577276703,1.7133080709
C,0,-3.496330994,-3.0184381765,1.478044817
C,0,-0.0228159612,-1.6298328816,-0.0005437006
C,0,0.615468959,-0.582918656,-1.8486551101
C,0,-0.7692869016,-0.3395654029,-1.7167216178
C,0,-1.4362885199,0.4313806718,-2.6807105283
C,0,-0.7056333126,0.9175167577,-3.7456210468
C,0,0.676231934,0.6619364711,-3.8572062844
C,0,1.3623820456,-0.0822458547,-2.9161860472
C,0,-0.1129588415,1.3310079674,0.5791406592
C,0,1.1502852789,1.744690759,0.4135121202
C,0,-3.9018791034,2.2113419029,0.4255037482
C,0,-4.2880360745,2.3618816112,-0.9011507601

C,0,-4.049393818,3.5726344334,-1.5400258458
C,0,-3.4306296705,4.6321985445,-0.8743447833
C,0,-3.065571839,4.4556915798,0.4652379966
C,0,-3.2985970248,3.2571231592,1.1212285493
C,0,-3.1729752853,5.9367866872,-1.5637597369
H,0,-1.8421500742,0.6542877362,2.6917508051
H,0,-1.5922985413,-1.0837356926,2.5754629789
H,0,0.4565987726,-0.3393933125,1.752476239
H,0,-3.2669321846,-0.8466069678,-0.8545411449
H,0,-2.2632469064,-3.2983929201,-1.6683951215
H,0,-2.8087051405,-5.6648447896,-1.2542982227
H,0,-3.7896191073,-6.3574096871,0.9203197545
H,0,-4.2323428268,-4.6497383212,2.6644863619
H,0,-3.7047968514,-2.2777557204,2.2427404326
H,0,0.0398803715,-2.3598765982,0.7946757544
H,0,-2.4998548105,0.628527143,-2.598773254
H,0,-1.198313509,1.5047900721,-4.5133940015
H,0,1.2163020784,1.0618287477,-4.7097510737
H,0,2.42773974,-0.2818406055,-2.9845800174
H,0,-0.9423090237,1.9202427625,0.1973594577
H,0,1.9902081691,1.1594448759,0.7789036268
H,0,1.3744129799,2.6826608284,-0.0851262198
H,0,-4.7917209925,1.5543575203,-1.4203980846
H,0,-4.3577483382,3.6976179265,-2.5736809243
H,0,-2.5996816426,5.2751364997,1.0049575298
H,0,-3.0305445196,3.1395574199,2.1654777376
H,0,-2.1026554126,6.1672988322,-1.5754534221
H,0,-3.5293848382,5.920402991,-2.5956578052
H,0,-3.672833601,6.7594488821,-1.0422163036
H,0,2.0112183993,-1.6802270274,-0.6075767788
S,0,4.7485874804,-1.6928945286,-0.8980902617
O,0,4.4385987142,-1.0602559601,-2.1948819283
O,0,3.5061237821,-2.1464252558,-0.1898819204
O,0,5.769919493,-2.7476907034,-0.948202493
C,0,5.4119161281,-0.3918732192,0.1420857218
C,0,5.4745428959,0.9133108671,-0.323548914
C,0,5.8618519243,-0.7004879073,1.4244979583
C,0,5.9839078971,1.9148942875,0.4998256852
H,0,5.1313107239,1.1392301605,-1.3273064057
C,0,6.3646400956,0.3053909471,2.2353817734
H,0,5.8230974899,-1.7235255875,1.7846626428
C,0,6.434243779,1.630543678,1.7880351682
H,0,6.0345301664,2.9349877609,0.1291188372
H,0,6.7154400181,0.0600536757,3.2344417127
C,0,6.989444206,2.7055790392,2.6748230662
H,0,8.0375433242,2.5077604036,2.9233702476
H,0,6.4407422567,2.7618118228,3.6206754156
H,0,6.937151802,3.6849635427,2.1936243727

B•TsOH
SCF Done: E(RPBE1PBE) = -2556.87385178 a.u.
Zero-point correction = 0.587642 Hartree/Particle
Sum of electronic and thermal Free Energies = -2556.364289 a.u.

S,0,3.5926332002,1.1915489981,3.3628371421
N,0,2.4754822475,1.9464772192,2.3800051576

N,0,-0.250964557,5.0396627831,0.834133269
O,0,3.7313619422,-0.1598655001,2.8429979305
O,0,4.7325684074,2.0914116159,3.4304934896
C,0,1.194729287,1.2614545457,2.1568478187
C,0,0.1510382922,2.2099370154,2.7347874674
C,0,0.7427743773,3.6014543822,2.3427011721
C,0,2.2767093932,3.3955031345,2.5684289478
C,0,3.1306084934,4.2587458053,1.6654003944
C,0,3.3529655501,5.5906932538,2.0155523284
C,0,4.0788943288,6.4277610567,1.175171718
C,0,4.5952515966,5.9388754349,-0.0203802792
C,0,4.3874819613,4.6075795778,-0.3664974206
C,0,3.6590764923,3.7704653459,0.4715307146
C,0,0.4151945686,3.9448619117,0.9188724523
C,0,-0.4577010083,5.6067702095,2.1127629427
C,0,0.1372930449,4.7758280926,3.0595629416
C,0,0.0643547463,5.1112307521,4.399785997
C,0,-0.6063666844,6.2855361659,4.7470940156
C,0,-1.1917325714,7.1013471294,3.7786873166
C,0,-1.1298470478,6.7734680851,2.4260560775
C,0,-1.2453049678,1.9532122372,2.2736147061
C,0,-2.2885680582,1.8457770858,3.0908550323
C,0,2.8866655764,1.0787557849,4.9905099336
C,0,3.0946648387,2.1114294099,5.9012176565
C,0,2.5010240181,2.0370229803,7.1544026141
C,0,1.706212377,0.946216356,7.5156427156
C,0,1.5241545465,-0.0819063047,6.5852820585
C,0,2.1094677033,-0.0270152186,5.3289022995
C,0,1.0969813314,0.8564321502,8.8813443358
H,0,1.174147145,0.2813907781,2.6349935522
H,0,1.0442126558,1.1085411209,1.0826555042
H,0,0.1951949561,2.1648943735,3.8287701395
H,0,2.4719536424,3.675777558,3.6099472672
H,0,2.9628634576,5.9749574422,2.9541163185
H,0,4.2464980272,7.4618676272,1.4593898563
H,0,5.1652636248,6.5901941076,-0.6753416006
H,0,4.7985920902,4.2154771061,-1.2916090001
H,0,3.516101151,2.7271501446,0.2096137782
H,0,0.6644930266,3.3706261303,0.0341539722
H,0,0.5137315421,4.4866922063,5.1654877403
H,0,-0.6739406186,6.5694071432,5.7922512479
H,0,-1.7048266813,8.0078260323,4.0821275502
H,0,-1.5762375342,7.3872939656,1.6491077434
H,0,-1.3916252051,1.8458859514,1.1985608073
H,0,-2.1796270667,1.9429535621,4.1685491092
H,0,-3.286766575,1.6561200904,2.7078806737
H,0,3.7315675444,2.9504195209,5.6432834628
H,0,2.6677446875,2.8375797618,7.8691052843
H,0,0.9220981663,-0.9454582617,6.8527200862
H,0,1.9802038949,-0.8423081192,4.6257897173
H,0,0.1102542579,0.3872325364,8.8472963967
H,0,0.9992347279,1.8424362424,9.341345633
H,0,1.7249754601,0.2449639777,9.5395481678
H,0,-0.6076112995,5.4639233165,-0.1061670045
S,0,-1.8456981795,7.1302621969,-1.7457494045
O,0,-2.1188872083,7.8887432903,-0.5118715733
O,0,-1.0288963889,5.8944427911,-1.4500136546
O,0,-1.2754886275,7.9012878351,-2.853853917

C,0,-3.4189812433,6.4997461456,-2.3173475229
C,0,-4.4179767472,6.1977756744,-1.3994785195
C,0,-3.6195032585,6.2732859728,-3.6743019079
C,0,-5.6225606132,5.6692921267,-1.8471136294
H,0,-4.258424371,6.3868930483,-0.3431427319
C,0,-4.8272956571,5.7421349793,-4.108516693
H,0,-2.837399174,6.5203318854,-4.3842285806
C,0,-5.8481127361,5.4335704177,-3.2054344326
H,0,-6.4041436165,5.4392194847,-1.1282389467
H,0,-4.9819988599,5.5682248427,-5.1698512342
C,0,-7.1632118127,4.8974234795,-3.6871995063
H,0,-7.8243352216,5.7159190404,-3.9953574335
H,0,-7.0340911334,4.2413973846,-4.552237822
H,0,-7.6780183044,4.3382723441,-2.9020143291

TS1-B
SCF Done: E(RPBE1PBE) = -2556.83797610 a.u.
Zero-point correction = 0.586455 Hartree/Particle
Sum of electronic and thermal Free Energies = -2556.330477 a.u.
Imaginary Freq. = -171.2079 cm^{-1}

N,0,1.4116864576,-0.496030047,0.0974868652
C,0,-0.5132211071,-1.5350596696,0.6472338372
C,0,0.5371824229,-0.691895815,1.0745071594
C,0,1.0419809669,-1.2440690085,-1.0149010391
C,0,-0.1383942171,-1.9421202451,-0.6940985691
C,0,-0.7156175056,-2.7855636895,-1.6434173298
C,0,-0.0949831445,-2.9063698366,-2.881657784
C,0,1.0748356851,-2.1954922585,-3.1809055794
C,0,1.6655313805,-1.3474195551,-2.2512948133
H,0,0.6598268852,-0.207349999,2.0332444213
H,0,-1.6195724084,-3.3449397511,-1.4228482988
H,0,-0.5221386625,-3.564926531,-3.6313311469
H,0,1.5311700316,-2.3106162042,-4.158899625
H,0,2.5671603949,-0.7802160971,-2.4646226314
S,0,4.5574297633,1.3900313001,-0.6888231536
O,0,3.5324185225,1.1092320086,0.3694046844
O,0,4.1705708822,0.8036353361,-1.9872052484
O,0,4.9347662584,2.8095669293,-0.7331742836
C,0,6.0207902297,0.5008425813,-0.1574996807
C,0,6.2207986937,-0.8126459455,-0.5623937051
C,0,6.9297413761,1.1151689196,0.6984815382
C,0,7.3348600262,-1.5107372919,-0.1083701545
H,0,5.5149969766,-1.2824872808,-1.2392850766
C,0,8.0368322244,0.4075976455,1.1465211065
H,0,6.7732786285,2.1450354705,1.0016323016
C,0,8.2590899825,-0.9152550597,0.751409628
H,0,7.48943332,-2.5365708685,-0.4313383182
H,0,8.7460338183,0.8923174029,1.8124052902
C,0,9.472338664,-1.6597379715,1.2245621333
H,0,9.6062064079,-1.555559067,2.3055687015
H,0,10.3797597475,-1.2706356287,0.7490135965
H,0,9.4042032137,-2.7244538409,0.9887826296
H,0,2.250485992,0.1336417736,0.1583017631
S,0,-4.1314674696,0.7496154356,1.612198241
N,0,-2.7664034693,-0.1688426892,1.1082539101

O,0,-4.5481457303,0.1499149337,2.864292261
O,0,-3.7634328967,2.1469626608,1.5417649723
C,0,-1.8740318134,0.2324573286,0.1349804643
C,0,-2.9279139539,-1.6203897134,1.1295292131
C,0,-1.5454887367,-2.1942995591,1.516633457
C,0,-1.2821228162,-2.0197141464,2.984660122
C,0,-1.0067362651,-3.0221482347,3.8124304222
C,0,-1.2228357119,1.5151223497,0.070317054
C,0,-0.7667504411,1.9313893315,-1.1920607195
C,0,-0.0886715177,3.1334816377,-1.3321453578
C,0,0.1578830836,3.9193462342,-0.2126415607
C,0,-0.2693088086,3.5032423694,1.0508342772
C,0,-0.9502944641,2.3110044415,1.1975637737
C,0,-5.3507745148,0.4038376577,0.3765495324
C,0,-5.3303531835,1.1240597532,-0.8162599297
C,0,-6.2811284763,0.8383932936,-1.7820598738
C,0,-7.2509208772,-0.1504736736,-1.575642393
C,0,-7.2452807881,-0.8499084672,-0.3675653248
C,0,-6.30003483,-0.5825160235,0.6146403541
C,0,-8.2735892601,-0.4382338137,-2.6311803706
H,0,-1.9545977764,-0.2993565568,-0.8110847101
H,0,-3.2147004202,-1.9820666423,0.1350531375
H,0,-3.6920838079,-1.9027885174,1.8524124083
H,0,-1.5660790784,-3.2643266798,1.2841617797
H,0,-1.3422960924,-1.00315218,3.3712647346
H,0,-0.9380197995,-4.0493266277,3.461194487
H,0,-0.8353860031,-2.8486043861,4.8707000573
H,0,-0.959812346,1.3085265683,-2.0603263689
H,0,0.2586088113,3.449854657,-2.3096974605
H,0,0.701440907,4.8528953014,-0.3166681565
H,0,-0.0536001279,4.1127373798,1.9220058488
H,0,-1.2561160494,1.9784682525,2.1823272084
H,0,-4.5963645165,1.9057353177,-0.9801235935
H,0,-6.2784323371,1.3999255182,-2.7116119166
H,0,-7.9961035145,-1.6131851663,-0.1873118669
H,0,-6.3102537379,-1.1211772313,1.5555178034
H,0,-8.8817574689,0.449092251,-2.8348465797
H,0,-8.9410265105,-1.2478946903,-2.3297580745
H,0,-7.7930554277,-0.7207364997,-3.5733565202

INT1-B
SCF Done: E(RPBE1PBE) = -2556.84384487 a.u.
Zero-point correction = 0.587300 Hartree/Particle
Sum of electronic and thermal Free Energies = -2556.335939 a.u.

S,0,4.1194718172,0.3105094315,1.5168491923
N,0,-1.2141490938,1.4077357894,0.077067769
N,0,3.0578863138,1.0746422473,0.3132395761
O,0,3.3138747811,-0.1543857689,2.6276465643
O,0,5.1302522649,1.3224165187,1.737027082
C,0,0.6348379831,2.6718840706,0.3335386638
C,0,2.1249938938,0.4755840195,-0.4142418476
C,0,3.1311407821,2.5331328876,0.1837558854
C,0,1.9144650531,3.2209171231,0.8742013626
C,0,2.01185319,3.1464930594,2.3713932843
C,0,2.2212944211,4.2007380169,3.1532789354

C,0,-0.1656136124,1.6830863758,0.8894080745
C,0,-1.1348435474,2.1980882312,-1.0407281507
C,0,0.0162601195,3.0170293467,-0.916733937
C,0,0.3211628928,3.925229952,-1.9418267794
C,0,-0.5147762374,3.9889239453,-3.044799044
C,0,-1.647156305,3.1602856465,-3.1492685911
C,0,-1.9737894954,2.2533099996,-2.1540723109
C,0,1.5815643358,-0.8527742035,-0.4270375327
C,0,1.5177202861,-1.7460827691,0.6587464462
C,0,0.9091748426,-2.9768271761,0.50004544
C,0,0.3625565012,-3.342927079,-0.7295833215
C,0,0.4003844857,-2.4627932752,-1.8060316946
C,0,0.9868357854,-1.219686285,-1.6521504022
C,0,4.7904679955,-1.0464479897,0.6208503895
C,0,5.4457517376,-0.8069605994,-0.5841204382
C,0,6.0464212678,-1.872917448,-1.2323225193
C,0,6.0147332099,-3.1624765069,-0.6888649009
C,0,5.3630345913,-3.3611562286,0.5313579954
C,0,4.7429710602,-2.313027586,1.1938948863
C,0,6.651579699,-4.3113845255,-1.4053033612
H,0,1.7999267846,1.0940866661,-1.2477121721
H,0,4.068624011,2.8837996886,0.6093296735
H,0,3.1200149607,2.7605089998,-0.8847486084
H,0,2.0043749769,4.2721385193,0.5766582048
H,0,1.9040477809,2.1625186861,2.8259172523
H,0,2.3273440343,5.2030553123,2.7439068433
H,0,2.2877309121,4.0973137014,4.2322874186
H,0,-0.0599851226,1.1683593633,1.834252791
H,0,1.1890078325,4.575051679,-1.87185975
H,0,-0.2961084127,4.6904559329,-3.8440096599
H,0,-2.2777117878,3.2329697873,-4.0300559854
H,0,-2.8342838483,1.5948220152,-2.2281163879
H,0,1.8948733507,-1.4585931644,1.6306000699
H,0,0.8409276588,-3.6532421768,1.3451761696
H,0,-0.120360975,-4.3085395893,-0.8384546514
H,0,-0.05048705,-2.7358959606,-2.753409228
H,0,0.9947488515,-0.5163972725,-2.4788874014
H,0,5.4876782172,0.1895459878,-1.0118932281
H,0,6.5582492823,-1.7009615359,-2.1738962156
H,0,5.3382092004,-4.3531660541,0.971478392
H,0,4.2403880842,-2.4752480744,2.1402489474
H,0,7.1349455937,-4.9956308118,-0.7033856584
H,0,5.8936810664,-4.8865725299,-1.9495540264
H,0,7.392979434,-3.969239386,-2.1303372588
S,0,-3.9203755607,-1.2244839022,-0.3253137753
O,0,-3.1413416817,-0.4341369026,0.6756810966
O,0,-3.8357768512,-0.6477683364,-1.6818641765
O,0,-3.6261331789,-2.6667198815,-0.253788616
C,0,-5.6312332752,-1.0434122963,0.1825547037
C,0,-6.5781417614,-0.564961754,-0.7109175992
C,0,-6.0089897524,-1.4017219374,1.4751884676
C,0,-7.9074062278,-0.4483097609,-0.3112437329
H,0,-6.2716423456,-0.2838629946,-1.7125575901
C,0,-7.3346091326,-1.2792632651,1.8626655438
H,0,-5.2685175258,-1.7728467021,2.1764366134
C,0,-8.30790343,-0.8025270029,0.976071291
H,0,-8.6444469362,-0.0722905283,-1.0156026908
H,0,-7.6226247141,-1.5584178524,2.8728857072

C,0,-9.7414597714,-0.6928761406,1.4044380633
H,0,-9.8342706462,-0.1383354642,2.3435529338
H,0,-10.1773542753,-1.6842089468,1.5711832414
H,0,-10.3445936957,-0.1851894928,0.6480388674
H,0,-1.951663023,0.7056879375,0.2712654353

TS2-B
SCF Done: E(RPBE1PBE) = -2556.83910898 a.u.
Zero-point correction = 0.587163 Hartree/Particle
Sum of electronic and thermal Free Energies = -2556.327808 a.u.
Imaginary Freq. = -236.5240 cm^{-1}

S,-3.907151271978,0.437150588727,-1.402977598261
N,0.995802467951,1.268190451517,0.153300234248
N,-2.897344057301,1.209196045538,-0.216461742040
O,-3.090857872822,-0.019125780683,-2.512477559135
O,-4.953731684883,1.412068528086,-1.645327039502
C,-0.619024938050,2.826362990026,-0.185180935573
C,-1.800518303049,0.655004762614,0.379041019352
C,-3.059725683960,2.636519115588,-0.003275207292
C,-1.883504196992,3.431740965572,-0.665548951442
C,-2.007630629868,3.469896854350,-2.162769270341
C,-2.196993825434,4.587459600049,-2.856218522042
C,-0.111955490901,1.568897507005,-0.594723794295
C,1.171723266087,2.233565253943,1.093290769906
C,0.168983672747,3.228985604781,0.919342396562
C,0.124051048930,4.335105572131,1.791607093187
C,1.071501719407,4.423646811824,2.785548270187
C,2.062734893012,3.424979646761,2.938830313534
C,2.130343077569,2.323370607754,2.111905763157
C,-1.411465317715,-0.763653239650,0.381696996993
C,-1.163809754968,-1.545402438393,-0.753103653884
C,-0.758857431137,-2.863256327432,-0.612078934037
C,-0.596248215678,-3.420651563310,0.652793182055
C,-0.823216162218,-2.647907837137,1.785502061927
C,-1.209467875196,-1.322318313938,1.651601827810
C,-4.567034282790,-0.944129990310,-0.527075615670
C,-5.269050748826,-0.724725163321,0.656927225835
C,-5.850869480318,-1.806897021863,1.292095145525
C,-5.755183327217,-3.098892672639,0.757478008115
C,-5.055146158267,-3.279419151360,-0.435465480942
C,-4.454133350548,-2.209315748315,-1.086310326027
C,-6.402872544979,-4.253173281691,1.456520544082
H,-1.582905208768,1.145504678818,1.326267591434
H,-4.014015912318,2.963089507709,-0.409901449892
H,-3.044517291661,2.820218175621,1.074192796895
H,-1.968352994605,4.450766315723,-0.274930037519
H,-1.935059329977,2.518798835696,-2.688895436161
H,-2.265926477071,5.557004315253,-2.367949891793
H,-2.287190335080,4.569136917575,-3.938171823915
H,-0.273963900592,1.074579386421,-1.543008754417
H,-0.634138727984,5.102927868807,1.672005580965
H,1.065227396481,5.269072876211,3.465722062851
H,2.791586902629,3.530793276854,3.736618102688
H,2.877184737396,1.543915340042,2.232489834095
H,-1.262773964431,-1.118601765479,-1.742632957859

H,-0.551006315837,-3.455273561048,-1.497218495921
H,-0.268494159638,-4.450200310150,0.753888497619
H,-0.677415412734,-3.068765064562,2.774642035736
H,-1.362522289248,-0.711880779493,2.535928724809
H,-5.356821210768,0.271560785545,1.078180843231
H,-6.394184388787,-1.649929204970,2.219076442343
H,-4.972528335768,-4.273376713482,-0.863634802562
H,-3.908021717933,-2.357524873129,-2.010507782406
H,-6.181548688226,-5.198383378514,0.957052188376
H,-6.062240249278,-4.322650907694,2.494225617854
H,-7.490473097279,-4.127369523432,1.483023617473
S,3.689789310386,-1.359296510400,0.639653199505
O,2.740835006281,-0.751405669868,-0.344576624218
O,3.750112242741,-0.587038772896,1.897231475924
O,3.471185502277,-2.803942308728,0.813709744362
C,5.304720801342,-1.189984978029,-0.122157300629
C,6.195535079755,-0.228681496178,0.333590438063
C,5.649721577196,-2.010893548044,-1.193520389626
C,7.436552440693,-0.091073008421,-0.282937833319
H,5.920500548179,0.401038720166,1.172886250050
C,6.887434146953,-1.863047294547,-1.801321258757
H,4.954965729865,-2.767547242066,-1.543632022279
C,7.803227271955,-0.902551206768,-1.355899710839
H,8.132670072450,0.659481880515,0.081872593387
H,7.153163820292,-2.508757761632,-2.634426825546
C,9.144251397813,-0.765459455226,-2.014042595904
H,9.041146446817,-0.556049786081,-3.083845276003
H,9.723512432719,-1.690305354787,-1.921979554062
H,9.726021874366,0.042612913030,-1.564670172000
H,1.631920956890,0.459686397704,-0.002674196308

INT2-B
SCF Done: E(RPBE1PBE) = -2556.85970549 a.u.
Zero-point correction = 0.588813 Hartree/Particle
Sum of electronic and thermal Free Energies = -2556.348267 a.u.

S,-3.77581,1.97683,-0.4417
N,0.4011,-0.50628,0.43097
N,-2.58042,1.7119,0.69177
O,-3.1211,2.64595,-1.55369
O,-4.87332,2.60673,0.27625
C,-1.64352,-0.73575,1.4814
C,-1.23916,1.37564,0.1867
C,-2.96044,1.24641,2.02209
C,-2.89114,-0.28495,2.15409
C,-2.92257,-0.70138,3.60313
C,-3.88769,-1.44023,4.13969
C,-0.61539,0.26383,1.0966
C,0.05645,-1.78377,0.41908
C,-1.23385,-1.96776,1.05256
C,-1.81521,-3.27006,1.12765
C,-1.11565,-4.30935,0.60258
C,0.16777,-4.10794,-0.01098
C,0.76891,-2.88668,-0.11375
C,-0.33017,2.58131,0.08695
C,-0.40182,3.61725,1.01842

C,0.46328,4.70077,0.92811
C,1.40815,4.75899,-0.0931
C,1.48051,3.73018,-1.0248
C,0.61228,2.64695,-0.93577
C,-4.36146,0.40477,-1.03596
C,-5.47309,-0.18256,-0.44067
C,-5.91032,-1.41861,-0.89843
C,-5.2551,-2.07741,-1.94193
C,-4.14935,-1.45623,-2.53137
C,-3.69913,-0.22072,-2.08996
C,-5.72136,-3.41731,-2.4235
H,-1.32745,0.92403,-0.80559
H,-2.27981,1.71054,2.74346
H,-3.96168,1.61089,2.24859
H,-3.74325,-0.74834,1.64552
H,-2.09929,-0.34276,4.21969
H,-4.72203,-1.8099,3.5485
H,-3.87516,-1.69576,5.19508
H,-0.19536,0.73745,1.99623
H,-2.781,-3.41005,1.59989
H,-1.51714,-5.31601,0.64057
H,0.67827,-4.97726,-0.41468
H,1.73495,-2.74073,-0.59073
H,-1.15062,3.59007,1.80396
H,0.39567,5.5057,1.65358
H,2.08261,5.60667,-0.16371
H,2.21086,3.76805,-1.82664
H,0.66832,1.85136,-1.67263
H,-6.00082,0.32986,0.35631
H,-6.78571,-1.87356,-0.44427
H,-3.63965,-1.94129,-3.35878
H,-2.85854,0.26255,-2.57594
H,-5.73044,-3.46244,-3.51617
H,-5.0488,-4.2087,-2.0732
H,-6.72532,-3.64505,-2.05865
H,1.3581,-0.17019,0.16172
S,3.81891,-0.41892,-1.19864
O,2.94893,0.10578,-0.09823
O,3.47992,-1.8198,-1.5323
O,3.88866,0.48061,-2.35951
C,5.46249,-0.45505,-0.4879
C,5.68702,-1.16053,0.6925
C,6.50923,0.20059,-1.11894
C,6.96224,-1.20241,1.23471
H,4.86756,-1.67336,1.18595
C,7.78635,0.14907,-0.56588
H,6.32205,0.74779,-2.03639
C,8.03432,-0.54836,0.61537
H,7.13265-1.75375,2.15576
H,8.6036,0.6628,-1.06447
C,9.40615,-0.59085,1.22047
H,9.4367,-0.03129,2.16201
H,10.15058,-0.15645,0.54899
H,9.70844,-1.61805,1.44712

TS3-B

SCF Done: E(RPBE1PBE) = -2556.85149855 a.u.
Zero-point correction = 0.584094 Hartree/Particle
Sum of electronic and thermal Free Energies = -2556.342324 a.u.
Imaginary Freq. = -698.3008 cm^{-1}

S,0,0.1525902428,-2.2665214372,0.9611469543
N,0,-1.0383858478,1.9936345086,-1.3768765824
N,0,-0.5255439475,-0.7527092463,1.1110955999
O,0,-0.706822735,-2.9990671288,0.0446703975
O,0,0.3822761644,-2.7446868652,2.316243337
C,0,0.4443565729,1.7592273151,0.3425166009
C,0,-1.1617265733,-0.181122636,-0.0860303412
C,0,0.0023542715,0.1522128081,2.1301557775
C,0,1.0279469044,1.1517797316,1.5711509062
C,0,1.4022452587,2.1677834488,2.6168470884
C,0,2.605291346,2.2475117005,3.1757702084
C,0,-0.828025861,1.3182096139,-0.1441112399
C,0,-0.0779794043,2.9260679403,-1.5288714152
C,0,0.8953572955,2.7863031473,-0.4911694358
C,0,2.0307076016,3.6329667268,-0.470189524
C,0,2.1474240594,4.5838677576,-1.4479235478
C,0,1.1488615754,4.7336936924,-2.4481369671
C,0,0.0344818756,3.9336562832,-2.5034441223
C,0,-2.6447935993,-0.4821584001,-0.1748588478
C,0,-3.4220558716,-0.6829323452,0.9632443891
C,0,-4.7788828111,-0.9603321191,0.8454299661
C,0,-5.3737980777,-1.0360316427,-0.4104856907
C,0,-4.6028325426,-0.8358063185,-1.5503970395
C,0,-3.243966897,-0.5636401184,-1.4311851383
C,0,1.7446435589,-2.0966333972,0.1839370361
C,0,2.8843191666,-1.9711483988,0.9758183205
C,0,4.1159231865,-1.7993626575,0.362790397
C,0,4.2317593951,-1.7500488806,-1.0309921825
C,0,3.0748001905,-1.8908118647,-1.7996444216
C,0,1.8319521935,-2.0676795263,-1.2039767523
C,0,5.5729740699,-1.5762418258,-1.6754769921
H,0,-0.6891958038,-0.6100226575,-0.9765162662
H,0,1.9359678278,0.6110065692,1.2721798858
H,0,0.6085856812,2.8449428485,2.9293727706
H,0,3.4168887199,1.5857554198,2.881630464
H,0,2.8180477074,2.9815781261,3.9473319578
H,0,-1.580245646,1.9750944913,0.6331970879
H,0,2.7791690539,3.5265918257,0.3065345467
H,0,3.0058907118,5.2466290146,-1.4625263937
H,0,1.2752775518,5.5126558668,-3.1936286065
H,0,-0.7227431497,4.0598502461,-3.2689102168
H,0,-2.961138216,-0.6436860315,1.9447532897
H,0,-5.3736043419,-1.1212635951,1.7392935056
H,0,-6.4327852762,-1.2580497971,-0.4998207347
H,0,-5.0555340631,-0.9036171171,-2.534869843
H,0,-2.6402910477,-0.4329867477,-2.326106841
H,0,2.8069400184,-2.0242251795,2.0561816489
H,0,5.0073810645,-1.71080767,0.9773315616
H,0,3.1470076046,-1.8727864062,-2.8829843872
H,0,0.9456930618,-2.2040680012,-1.813923109
H,0,5.4856519466,-1.4879698382,-2.7603319811
H,0,6.078752349,-0.6823729933,-1.2969787819
H,0,6.2223329388,-2.4304479942,-1.4559187261

H,0,-1.9761904487,2.1278424757,-1.7431961527
H,0,-0.8416102292,0.6965572935,2.5671789528
H,0,0.4410535187,-0.4460377805,2.9279493487
S,0,-3.3918039459,3.7434104,0.683816528
O,0,-3.572265121,3.3813728209,-0.732338687
O,0,-3.0125810928,5.1333595571,0.9457101291
O,0,-2.4335783697,2.777392901,1.3740041922
C,0,-4.9506308406,3.4597202369,1.5022688119
C,0,-5.1532500348,3.9714926092,2.7787921461
C,0,-5.9416038707,2.71848795,0.8702382089
C,0,-6.3582735236,3.7297692873,3.4257987473
H,0,-4.3804420147,4.5611593336,3.2606808989
C,0,-7.1429746501,2.4890032473,1.5281629737
H,0,-5.7719261169,2.3323947383,-0.1284892281
C,0,-7.369932243,2.9847269466,2.8146076773
H,0,-6.518682211,4.1323571557,4.4219134773
H,0,-7.9202698016,1.9145524832,1.0313872823
C,0,-8.6657736413,2.7148949815,3.5193098232
H,0,-9.5218593582,2.9404162617,2.8762905931
H,0,-8.7443204292,1.6591526331,3.8021373409
H,0,-8.7546669593,3.3115508325,4.4299002662

***cis*-Pro**
SCF Done: E(RPBE1PBE) = -2556.88844853 a.u.
Zero-point correction = 0.588376 Hartree/Particle
Sum of electronic and thermal Free Energies = -2556.378945 a.u.

S,0,3.9864005697,1.73464024,-0.7980396081
N,0,0.7936916171,-1.7619079779,-0.5098617465
N,0,3.8818792552,0.2364651805,-0.0863091576
O,0,5.0462783664,2.4390122303,-0.090640935
O,0,4.0932087303,1.4846966584,-2.2306754906
C,0,1.5808988735,-0.6060572183,1.2508876619
C,0,3.1258280308,-0.8260390892,-0.7688163537
C,0,3.9335125544,0.1312389921,1.3714999095
C,0,2.5556633124,0.1982181106,2.0596071457
C,0,2.6981569699,-0.2999207282,3.4700607839
C,0,2.5509641702,0.4536829915,4.5559812126
C,0,1.861725486,-1.063306092,-0.0099647369
C,0,-0.2131320603,-1.7620304284,0.4185363884
C,0,0.242418805,-1.0320830518,1.548648256
C,0,-0.6228713097,-0.8668322362,2.640535487
C,0,-1.8898107217,-1.4234641485,2.5867881948
C,0,-2.3187837872,-2.1490809663,1.4593312986
C,0,-1.4868051651,-2.3272924079,0.3600963302
C,0,4.0050876173,-2.0584924148,-0.9466300541
C,0,3.7861068638,-3.2375181457,-0.2390185491
C,0,4.6229536644,-4.3363307787,-0.4248391627
C,0,5.6826627146,-4.263653127,-1.3201071988
C,0,5.9054556024,-3.0863778719,-2.0317432922
C,0,5.0715957535,-1.9920020739,-1.8458946042
C,0,2.4772723458,2.6363368717,-0.5214802505
C,0,1.3656406942,2.3770227626,-1.3181211154
C,0,0.1809557625,3.052719121,-1.0639645707
C,0,0.0893307753,3.9901306703,-0.030917979
C,0,1.2265287854,4.2434721418,0.7405678498

C,0,2.418824412,3.5725756801,0.5058118754
C,0,-1.1882547445,4.7308430659,0.2208561295
H,0,2.881195258,-0.4576089656,-1.7701570652
H,0,4.5969842854,0.9074267742,1.7530801598
H,0,4.4037459332,-0.8339030791,1.5915845778
H,0,2.2228556295,1.2432017982,2.0999733563
H,0,2.9591879456,-1.3535037462,3.5760119674
H,0,2.2865726008,1.5067336555,4.4861940241
H,0,2.6850712046,0.042141887,5.5523207328
H,0,-2.7309711156,-0.2830272179,0.2861009865
H,0,-0.3043238092,-0.3104851135,3.5157323156
H,0,-2.5679745399,-1.2993785343,3.4249250471
H,0,-3.3118317713,-2.5881795565,1.4532810557
H,0,-1.8122103889,-2.8912029106,-0.5083311585
H,0,2.9588057907,-3.3047784781,0.4611863567
H,0,4.4428282026,-5.2495830291,0.1340123239
H,0,6.3337229292,-5.1200048762,-1.4659595263
H,0,6.7300725457,-3.0228332725,-2.7350961678
H,0,5.2479924827,-1.0722796723,-2.3967550995
H,0,1.4231124777,1.6642794415,-2.1336157576
H,0,-0.6895689283,2.8491634251,-1.6801032007
H,0,1.1784294109,4.9774018747,1.5399342698
H,0,3.298220389,3.777899827,1.1059927766
H,0,-2.0556403361,4.1404810718,-0.0831613879
H,0,-1.2101008731,5.6662491372,-0.3505293778
H,0,-1.2957508482,4.9903586628,1.277058145
H,0,0.7484181554,-2.1874759954,-1.423850387
S,0,-3.9030777083,0.5493052387,-1.333816539
O,0,-3.0368249766,0.6101310829,0.0231013597
O,0,-3.2537124478,-0.3621696679,-2.258080044
O,0,-4.0998418754,1.9377356584,-1.6897490724
C,0,-5.444875345,-0.1505840964,-0.8241729123
C,0,-5.6930208301,-1.4983202197,-1.0491115019
C,0,-6.3791494465,0.6621851187,-0.1864720265
C,0,-6.8992543368,-2.0387392526,-0.6204655136
H,0,-4.9608931477,-2.1142370922,-1.5594942387
C,0,-7.5736766603,0.1032660822,0.235591347
H,0,-6.1755391325,1.7155891104,-0.0280359248
C,0,-7.853048443,-1.252885884,0.0280612986
H,0,-7.1032202648,-3.0903493146,-0.7976085031
H,0,-8.3077547055,0.7313889174,0.7315842124
C,0,-9.1549839798,-1.8351979412,0.4855726336
H,0,-9.3101629745,-1.655561528,1.5540582721
H,0,-9.9947416271,-1.3719581298,-0.0430677595
H,0,-9.1949316722,-2.9118177398,0.3084561674

TS2-B′
SCF Done: E(RPBE1PBE) = -2556.83351018 a.u.
Zero-point correction = 0.586382 Hartree/Particle
Sum of electronic and thermal Free Energies = -2556.326231 a.u.
Imaginary Freq. = -186.3832 cm^{-1}

S,0,-3.5343171133,0.6032646392,0.9017075483
N,0,1.0909756396,1.2934247826,-0.2811457777
N,0,-2.6410894511,0.1330567575,-0.5083400842
O,0,-4.1971197859,1.8521181547,0.578765965

O,0,-2.6220621683,0.4943172697,2.023825933
C,0,-0.8860056415,2.1199961154,-1.0220940038
C,0,-1.3945160463,-0.3975752468,-0.3342252767
C,0,-2.9148389279,0.8456350665,-1.7492560433
C,0,-2.1718182315,2.2244831612,-1.7613022685
C,0,-1.9868882626,2.6470380319,-3.1957166195
C,0,-2.5902893652,3.6987830494,-3.7389869752
C,0,0.0898376896,1.1166939546,-1.197998837
C,0,0.7634958897,2.3276058739,0.5383295091
C,0,-0.4809388091,2.8697342798,0.1065549512
C,0,-1.0453055089,3.9561529373,0.8069161564
C,0,-0.3646369613,4.459409405,1.8908372091
C,0,0.8669464012,3.8990015439,2.3067924394
C,0,1.4469008982,2.833276293,1.6502075815
C,0,-0.959224389,-1.5564187731,-1.1139949776
C,0,0.326881953,-2.0742913152,-0.8952049639
C,0,0.7379135886,-3.2196362079,-1.5561592302
C,0,-0.1264961233,-3.8701282458,-2.4343073332
C,0,-1.4110993333,-3.3751497108,-2.6398590097
C,0,-1.8297632489,-2.2257433813,-1.9858276953
C,0,-4.7414451655,-0.6813156519,0.9988150395
C,0,-4.4094072876,-1.8766513129,1.6308791413
C,0,-5.3578651873,-2.8852701754,1.6850476931
C,0,-6.6265050971,-2.7185743465,1.1186074771
C,0,-6.927073007,-1.5053011466,0.4941220637
C,0,-5.993535433,-0.4809727333,0.4274183074
C,0,-7.650668826,-3.8063034466,1.2128282838
H,0,-0.9911598733,-0.311467439,0.6723891925
H,0,-2.5463501342,0.2312401004,-2.5721953376
H,0,-3.9868054554,0.9875256247,-1.8833037586
H,0,-2.8038970528,2.9563512746,-1.2542071061
H,0,-1.318922972,2.0289577928,-3.7946461017
H,0,-3.2570833991,4.3347242156,-3.1614832653
H,0,-2.4300097104,3.9623662543,-4.7803433101
H,0,0.2592978266,0.5059511207,-2.0719834847
H,0,-1.9905245882,4.3867261042,0.4927563913
H,0,-0.7748546567,5.2977560906,2.4443394575
H,0,1.3679520575,4.3218765532,3.1722667656
H,0,2.3881074982,2.3931240706,1.966189223
H,0,1.0175233045,-1.5727016596,-0.22319947
H,0,1.7359185042,-3.6077134673,-1.3816115892
H,0,0.1977145708,-4.7686326122,-2.9495866046
H,0,-2.0951289925,-3.893597905,-3.3037304265
H,0,-2.8467922102,-1.8754854125,-2.1219999883
H,0,-3.4324955263,-2.0143461117,2.0816607705
H,0,-5.1105041387,-3.8199078675,2.1790703433
H,0,-7.9087345037,-1.3573782117,0.0546772185
H,0,-6.2386652688,0.4615716672,-0.0490954619
H,0,-8.1844332061,-3.7428237971,2.1682323325
H,0,-8.3928887424,-3.7267884354,0.4153396906
H,0,-7.1870759955,-4.7947681751,1.1655096753
S,0,4.114190862,-0.5179447295,1.0946055391
O,0,3.1399590314,-0.4700092613,-0.043846203
O,0,4.161986388,0.767276678,1.8201594214
O,0,3.9276808242,-1.7024673603,1.9469509434
C,0,5.712365837,-0.716782307,0.3074465774
C,0,6.2073081168,0.2972330497,-0.5101359287
C,0,6.4471942974,-1.8771869656,0.5006730939

C,0,7.436085918,0.139451062,-1.1322204125
H,0,5.6356550006,1.2081605572,-0.6563019677
C,0,7.6811505251,-2.0237089834,-0.1286796845
H,0,6.0544019987,-2.6571509449,1.143757264
C,0,8.1940125866,-1.0243792627,-0.9541096494
H,0,7.8177553319,0.9338795447,-1.7682517544
H,0,8.2539304368,-2.9336902125,0.0273973154
C,0,9.5214632743,-1.1814585101,-1.6350367816
H,0,9.4162371681,-1.1226540202,-2.7235784733
H,0,9.9814122829,-2.1418283588,-1.3911342843
H,0,10.2147348602,-0.3880818771,-1.3363744653
H,0,1.9255691254,0.6820130257,-0.1843390287

TS-allyl-B
SCF Done: E(RPBE1PBE) = -2556.83316428 a.u.
Zero-point correction = 0.587146 Hartree/Particle
Sum of electronic and thermal Free Energies = -2556.321973 a.u.
Imaginary Freq. = -261.5873 cm^{-1}

S,0,-4.0809327328,0.7042168001,-1.4788593464
N,0,-2.8580281956,-0.4215382238,-1.3476544079
N,0,0.9292820676,-1.3057442238,0.743026218
O,0,-4.0968938793,1.0887808157,-2.8820368807
O,0,-5.240879953,0.0932464028,-0.850782089
C,0,-1.6439294456,-0.2759469131,-2.1232605674
C,0,-0.50582104,0.1845001929,-1.260663029
C,0,-1.2865216637,-0.9495348509,0.4397747295
C,0,-2.6863551675,-1.154262862,-0.0876849807
C,0,-3.013462821,-2.6382873151,-0.2313961093
C,0,-2.7654233071,-3.4942831563,0.8434840505
C,0,-3.0722652085,-4.8460062593,0.7519113643
C,0,-3.6266242222,-5.3590095542,-0.4176370365
C,0,-3.8753926698,-4.5094941141,-1.488815719
C,0,-3.573157802,-3.1531181286,-1.3960891603
C,0,-0.0991936477,-1.5171517855,-0.099760924
C,0,0.4878555713,-0.5792078229,1.821037421
C,0,-0.901669964,-0.348206126,1.6753481954
C,0,-1.6068784568,0.3392310918,2.6747446703
C,0,-0.9049197183,0.7857345414,3.7753065612
C,0,0.4835710516,0.5685932765,3.8888444155
C,0,1.2043282678,-0.1086821051,2.9221308126
C,0,0.8253567814,0.2395956839,-1.8448435608
C,0,1.7092758911,1.181866206,-1.4985684628
C,0,-3.6522260655,2.1404301764,-0.5237022009
C,0,-4.19054239,2.3011081558,0.7474929452
C,0,-3.8343520816,3.4176474619,1.4948347651
C,0,-2.9489789937,4.3718447131,0.9923838365
C,0,-2.4364188936,4.1922344396,-0.2983284161
C,0,-2.7831749776,3.0891674957,-1.0614058396
C,0,-2.5534956323,5.5658220142,1.8055026922
H,0,-1.8087822735,0.4521152507,-2.9231365445
H,0,-1.4007220837,-1.2264111068,-2.6128125229
H,0,-0.7344592471,1.0409288288,-0.6298065443
H,0,-3.3765379048,-0.7295207099,0.6484411514
H,0,-2.3315011979,-3.104341134,1.7603159482
H,0,-2.8761884263,-5.5001046132,1.5956770701

H,0,-3.8643813459,-6.4155105604,-0.4909811543
H,0,-4.3111160959,-4.8995881029,-2.4034350662
H,0,-3.7795736601,-2.491972282,-2.2307534727
H,0,-0.0156145354,-2.2155435068,-0.9215671243
H,0,-2.6750822637,0.5062731331,2.5877554184
H,0,-1.4242274649,1.3127896142,4.5686691328
H,0,1.0015116793,0.9406324631,4.7673566344
H,0,2.2735467218,-0.2832524098,2.9988970483
H,0,1.0796044448,-0.5110233432,-2.5902896175
H,0,1.4729782866,1.9323336464,-0.7490529153
H,0,2.6879006149,1.2296092689,-1.9649750477
H,0,-4.8936561963,1.5746211259,1.1391256472
H,0,-4.2588058258,3.5509902429,2.4854146107
H,0,-1.7634144327,4.9361768107,-0.7148325102
H,0,-2.399526199,2.9780118147,-2.0697558899
H,0,-1.4827992768,5.5407652566,2.0350600889
H,0,-3.1017852615,5.6053510301,2.7489389741
H,0,-2.7428049514,6.4943460302,1.2580887443
H,0,1.9221911642,-1.589544652,0.5633727111
S,0,4.6601166281,-1.5776823037,0.9318018379
O,0,4.3168925241,-0.9362503848,2.2161797281
O,0,3.4411817204,-2.0807475145,0.2183777275
O,0,5.7148269583,-2.5978501832,1.0081902795
C,0,5.2998981775,-0.2715515121,-0.1172850061
C,0,5.228954361,1.0531611794,0.2897099593
C,0,5.8585069225,-0.5952687415,-1.3519134483
C,0,5.7186810443,2.0569481069,-0.5423232262
H,0,4.8012023034,1.2925845808,1.2572640876
C,0,6.3383421651,0.4133750099,-2.1740050339
H,0,5.9215779039,-1.632172373,-1.6661619624
C,0,6.279768349,1.7565289708,-1.7832585375
H,0,5.6666222642,3.0922236436,-0.2162113846
H,0,6.774550364,0.1555558527,-3.1355568699
C,0,6.8324454959,2.8338702279,-2.668515418
H,0,7.9282111058,2.8126324216,-2.6701370833
H,0,6.5069261181,2.7046530807,-3.7051496268
H,0,6.5203378184,3.8254966163,-2.3321730408

C•TsOH
SCF Done: E(RPBE1PBE) = -2556.87207737 a.u.
Zero-point correction = 0.588250 Hartree/Particle
Sum of electronic and thermal Free Energies = -2556.360451 a.u.

S,0,4.7458889753,0.6866197072,0.0290306387
N,0,3.4737377187,-0.1792371396,0.6845151559
N,0,-1.0707841897,-0.8228420266,-0.1559330478
O,0,5.1262753914,-0.0208551254,-1.1824004464
O,0,5.6931824125,0.8784697271,1.1160917578
C,0,2.4269857839,-0.640949088,-0.2476957244
C,0,2.8793582303,0.3410189103,1.9214713792
C,0,1.4496255458,0.7073494423,1.5345053579
C,0,1.0925273964,-0.4683903496,0.5582760976
C,0,-0.0454675009,-0.0659113849,-0.3153743449
C,0,-0.7976533573,-1.8437487638,0.782821886
C,0,0.5137187305,-1.6897240523,1.2349448458
C,0,1.0090479174,-2.5899674784,2.1636554189

C,0,0.1687528616,-3.611256631,2.6111234492
C,0,-1.1381471104,-3.7367737876,2.1429611407
C,0,-1.6526721814,-2.8438736531,1.2065330522
C,0,0.5210116035,0.8946431206,2.6877133796
C,0,-0.2085193346,1.9909300137,2.8736824656
C,0,2.644084358,-2.0340629924,-0.7901410419
C,0,1.9414774998,-2.4233032367,-1.9305591307
C,0,2.0798090278,-3.7088509795,-2.440821585
C,0,2.9336642195,-4.6155990139,-1.8205937335
C,0,3.6485525139,-4.2260659566,-0.6929042748
C,0,3.5043668205,-2.9416557585,-0.1785716269
C,0,4.1265688809,2.2846575934,-0.4432672063
C,0,4.1246166541,3.3249330835,0.4848711863
C,0,3.602393779,4.5553350837,0.1177628634
C,0,3.0858936939,4.7711995376,-1.1647738095
C,0,3.1116328561,3.7155036128,-2.0782946001
C,0,3.6302736594,2.4744147766,-1.7297982103
C,0,2.5457120123,6.1147764312,-1.5476723784
H,0,2.3639852783,0.0455307239,-1.1017526113
H,0,3.4346807502,1.1980781884,2.304025595
H,0,2.8973994283,-0.4338885811,2.6937503511
H,0,1.4776681168,1.6238390384,0.9343810159
H,0,-0.0601696362,0.7801620558,-0.9939374167
H,0,2.0235904115,-2.5210097588,2.5385220492
H,0,0.5458790425,-4.3260413068,3.3351850736
H,0,-1.763437489,-4.544605213,2.5083174265
H,0,-2.6631270553,-2.9164321134,0.8146440076
H,0,0.4688504877,0.0797603864,3.4077439278
H,0,-0.1766086648,2.8227728198,2.1736282699
H,0,-0.8627745592,2.0928719556,3.7342853216
H,0,1.2843283779,-1.7151796716,-2.4299885084
H,0,1.5261255153,-3.9986358846,-3.3283799215
H,0,3.0483049279,-5.6180635307,-2.2208061616
H,0,4.327004944,-4.923762775,-0.2116123194
H,0,4.0764578202,-2.634739109,0.69034547
H,0,4.545908302,3.1796311239,1.4731855304
H,0,3.6066419369,5.3696110384,0.836782841
H,0,2.7328044306,3.8683449085,-3.0843095203
H,0,3.6733297084,1.672089362,-2.4582359731
H,0,1.8150102999,6.4697460272,-0.8145817493
H,0,2.0662898978,6.0888117514,-2.5283114836
H,0,3.3495042516,6.8581597341,-1.5845708499
H,0,-2.0183164363,-0.679259837,-0.6773463606
S,0,-4.5284490635,-1.0904090005,-1.3847167367
O,0,-3.218920238,-0.341293381,-1.4651855024
O,0,-4.3942898519,-2.2895702593,-0.5386282515
O,0,-5.107431137,-1.3094815707,-2.7128509083
C,0,-5.630032427,0.0254181706,-0.5231209366
C,0,-6.5319230547,0.7994314584,-1.2407565703
C,0,-5.5573566372,0.1331282419,0.8626754928
C,0,-7.3629232015,1.686769688,-0.5648660383
H,0,-6.5906666176,0.6963763699,-2.3188251129
C,0,-6.3921190882,1.0218582816,1.5248097387
H,0,-4.8638857381,-0.486971029,1.4215367377
C,0,-7.3056986563,1.8158257958,0.8233248787
H,0,-8.073635842,2.2850729389,-1.1281446564
H,0,-6.3400139759,1.0968756542,2.6076189499
C,0,-8.1863761914,2.7898119919,1.5474209143

H,0,-8.5797958525,2.3604681324,2.4732440534
H,0,-7.6259618686,3.6912144839,1.8214679518
H,0,-9.0301716837,3.101469718,0.9272580479

TS1-C
SCF Done: E(RPBE1PBE) = -2556.84360081 a.u.
Zero-point correction = 0.587047 Hartree/Particle
Sum of electronic and thermal Free Energies = -2556.334434 a.u.
Imaginary Freq. = -184.1627 cm^{-1}

N,0,1.4069538626,0.2032138028,0.6856308138
C,0,-0.6634354393,0.4092484933,1.5703671724
C,0,0.336522189,-0.472160189,1.0820242434
C,0,1.2141161332,1.5578499043,0.9292674121
C,0,-0.0644277324,1.726158646,1.4969562246
C,0,-0.4981453278,3.0191272563,1.8087814856
C,0,0.3605722204,4.0826288815,1.5728458427
C,0,1.6329981141,3.882160387,1.0192258752
C,0,2.0814618975,2.6136911628,0.6800590144
H,0,0.2967694787,-1.5525594437,1.0255373189
H,0,-1.4810375003,3.1998205219,2.2290689781
H,0,0.041101079,5.090634466,1.8173389533
H,0,2.2776355728,4.7378678314,0.8450587932
H,0,3.0513859551,2.4365894742,0.2241907494
S,0,4.5838863148,-0.2203110516,-1.2711026169
O,0,3.5313770695,-0.9285969127,-0.472162984
O,0,4.5941912796,1.2313795094,-1.003996447
O,0,4.5349235967,-0.5834393443,-2.6960725322
C,0,6.1342956491,-0.8561050611,-0.6376584082
C,0,6.412190268,-2.2178550818,-0.7385515963
C,0,7.0533813606,0.0023804831,-0.0531041828
C,0,7.6126452138,-2.710149923,-0.2504333174
H,0,5.6941245945,-2.8891934056,-1.1983066933
C,0,8.2564884493,-0.5042282,0.4324464059
H,0,6.8261649043,1.0604634475,0.0171705116
C,0,8.5556249544,-1.8627225112,0.3442169126
H,0,7.8257680746,-3.7728184162,-0.3315531152
H,0,8.9742219489,0.1728404205,0.8873406282
C,0,9.848087971,-2.4121918932,0.8712445611
H,0,9.6711564402,-3.1190525531,1.6890590663
H,0,10.3950882918,-2.9522386455,0.0916428224
H,0,10.4943313819,-1.6163789501,1.249025687
H,0,2.2566477305,-0.2155756331,0.2342716183
S,0,-3.4505723794,-1.9525518155,0.0510809773
N,0,-2.8822894595,-0.3314448935,0.2335591458
O,0,-3.3991130884,-2.6242003185,1.3390956616
O,0,-2.7219749319,-2.4878444842,-1.0857234809
C,0,-1.7180644093,0.0103872382,-0.4153218868
C,0,-3.1207813815,0.2798950058,1.5340986389
C,0,-1.8551588989,-0.0214788599,2.3735072016
C,0,-1.9494164447,0.6045353198,3.7363170256
C,0,-1.9995568067,-0.1009252235,4.8616864128
C,0,-1.529107798,1.2772638349,-1.1031963584
C,0,-0.3715251149,1.4152539514,-1.8849532479
C,0,-0.1401778168,2.5837088751,-2.59493529
C,0,-1.069359008,3.6178118917,-2.5524879576

C,0,-2.2368901255,3.4768266315,-1.8041371949
C,0,-2.4656360774,2.32125526,-1.0748190663
C,0,-5.1399171736,-1.6756815374,-0.3776592009
C,0,-5.4470085324,-1.2219789247,-1.6594234253
C,0,-6.7737782658,-1.0040712249,-1.9859268636
C,0,-7.7966018968,-1.2276407265,-1.054544207
C,0,-7.4541071876,-1.684736348,0.2186098823
C,0,-6.1288152361,-1.9114002603,0.5687884295
C,0,-9.2255161656,-0.9904417263,-1.4321079041
H,0,-1.2143304989,-0.8269495072,-0.8925558361
H,0,-3.2611424874,1.3559327172,1.4201594966
H,0,-4.0229627172,-0.134561107,1.9858397021
H,0,-1.809563664,-1.1063239379,2.4999694792
H,0,-1.9995937274,1.6900434003,3.7821733794
H,0,-1.9527656542,-1.1875400568,4.8574219227
H,0,-2.0852935609,0.3883296234,5.8275299841
H,0,0.337685237,0.5954017221,-1.9425715717
H,0,0.7614430241,2.6814793314,-3.1903098616
H,0,-0.8935378297,4.52883095,-3.1156683274
H,0,-2.9739162421,4.2733576925,-1.7928926411
H,0,-3.3899186268,2.2153568922,-0.5183102252
H,0,-4.6644387419,-1.0507581495,-2.3908093831
H,0,-7.0248342197,-0.6568516527,-2.9837049519
H,0,-8.2352274276,-1.8701232622,0.94929299
H,0,-5.8697170465,-2.2745245557,1.5568721163
H,0,-9.5252523873,-1.6567752464,-2.247757994
H,0,-9.8953591064,-1.1627591798,-0.587522241
H,0,-9.3713942008,0.0351777181,-1.7854422859

INT1-C
SCF Done: E(RPBE1PBE) = -2556.84808360 a.u.
Zero-point correction = 0.588080 Hartree/Particle
Sum of electronic and thermal Free Energies = -2556.336227 a.u.

S,0,-3.8352248505,0.1440479333,1.2515071168
N,0,1.1942351153,1.6366543794,-0.2011201978
N,0,-3.0038214384,0.8968300582,-0.1357834879
O,0,-3.1929484488,-1.1431091088,1.418183447
O,0,-3.8285466217,1.1317184548,2.3086916892
C,0,-0.7491113516,2.7636800137,0.0020464299
C,0,-2.0617796962,0.1796056332,-0.7186230496
C,0,-3.2083253736,2.33393298,-0.2708742162
C,0,-2.1050253187,3.0964778806,0.5321576715
C,0,-2.4473036109,4.5612029927,0.5276025511
C,0,-2.7280186164,5.2522537499,1.6277368075
C,0,0.076793265,1.7902910323,0.5482945963
C,0,1.1352883924,2.4979375568,-1.2659991425
C,0,-0.0761991285,3.2356139046,-1.1738179578
C,0,-0.3606984512,4.1784469638,-2.1776596684
C,0,0.5435549135,4.3568113166,-3.2098040262
C,0,1.7326535346,3.608580433,-3.2764331573
C,0,2.0453797113,2.6687492762,-2.3085211817
C,0,-1.4127532889,0.3166710073,-1.9882971654
C,0,-1.8317370052,1.131673514,-3.0580006564
C,0,-1.1354967155,1.1103333012,-4.2513041886
C,0,-0.0018701377,0.3090067542,-4.3960265625

C,0,0.4181198714,-0.5084450964,-3.3524027007
C,0,-0.2892425856,-0.5181177173,-2.1635949478
C,0,-5.4641842656,-0.0500595392,0.6220785439
C,0,-6.4472217616,0.8600247469,0.9991612299
C,0,-7.7308874151,0.6888270154,0.5031336477
C,0,-8.0392603514,-0.3684648841,-0.3573287794
C,0,-7.0240739165,-1.2634670106,-0.715825632
C,0,-5.735423777,-1.1168001716,-0.2322557272
C,0,-9.4345541205,-0.5611182547,-0.86134416
H,0,-1.8271839209,-0.7405815048,-0.1861729293
H,0,-4.1982710446,2.5806208126,0.116436903
H,0,-3.1814749923,2.6175332436,-1.320096433
H,0,-2.1668781354,2.7438707602,1.5647052975
H,0,-2.4720974108,5.0623064093,-0.4374629148
H,0,-2.7138885768,4.7890534251,2.6117634534
H,0,-2.9695437673,6.3102058864,1.5806166155
H,0,-0.0656091089,1.2157074333,1.4543208852
H,0,-1.2710920938,4.7673621227,-2.1563253583
H,0,0.3318310971,5.0862924352,-3.9857353426
H,0,2.4180527465,3.7709815423,-4.1027311756
H,0,2.949259863,2.0677079687,-2.3515849897
H,0,-2.7216743744,1.7430417244,-2.9835405791
H,0,-1.4725040496,1.7258808672,-5.0783172524
H,0,0.5452427205,0.3139749711,-5.3332239221
H,0,1.2928327019,-1.1388070563,-3.4652362306
H,0,0.0221655307,-1.1684886957,-1.3524211997
H,0,-6.2170647243,1.6752746306,1.6756441685
H,0,-8.5087479693,1.3881487943,0.7931648624
H,0,-7.2500167704,-2.0912459991,-1.3805504639
H,0,-4.9603228128,-1.8237187957,-0.5070951636
H,0,-9.4400071674,-1.0150948643,-1.8551864722
H,0,-9.9785983242,0.3851121719,-0.9019311642
H,0,-9.9880633398,-1.2310146592,-0.1927872035
S,0,4.0863440568,-0.7691424449,-0.7071792886
O,0,3.1810268438,-0.1789342783,0.326008081
O,0,4.2726700747,0.1289130045,-1.8641375085
O,0,3.7092654628,-2.1501469284,-1.0586854362
C,0,5.6858782206,-0.8814663595,0.0958515453
C,0,6.7474599041,-0.1090165527,-0.3514373341
C,0,5.8561987494,-1.7464706504,1.1750050421
C,0,7.9845048848,-0.2040338486,0.2817385342
H,0,6.603244876,0.5574859196,-1.1947307611
C,0,7.0920027848,-1.8325720157,1.797564601
H,0,5.0269179284,-2.3539458335,1.5230309979
C,0,8.178180958,-1.063553027,1.361867502
H,0,8.8132588042,0.4016796298,-0.0746361264
H,0,7.2199649923,-2.5113474282,2.6368173619
C,0,9.5110400337,-1.17431883,2.0415398966
H,0,9.4355247874,-0.9199300933,3.1040041156
H,0,9.898706223,-2.1969067347,1.9826335975
H,0,10.2469555715,-0.5078181108,1.5856742462
H,0,1.9590349906,0.9642164427,-0.0130168728

TS2-C
SCF Done: E(RPBE1PBE) = -2556.84461963 a.u.
Zero-point correction = 0.587948 Hartree/Particle

Sum of electronic and thermal Free Energies = -2556.333686 a.u.
Imaginary Freq. = -200.6794 cm^{-1}

S,0,-3.3620888549,-0.5306152878,0.8652045291
N,0,1.1165375941,1.3506914265,-0.3843361485
N,0,-2.7295088496,0.541117044,-0.3398975963
O,0,-2.5930925275,-1.7552738238,0.736421922
O,0,-3.4053175655,0.1763790958,2.1340139132
C,0,-0.7938593792,2.5140067378,0.0131865554
C,0,-1.5169933354,0.2227240134,-0.8978723997
C,0,-3.1767381764,1.9149819733,-0.2301360628
C,0,-2.1434856777,2.752020392,0.5922639997
C,0,-2.6036645678,4.180781975,0.6271631807
C,0,-2.7923290974,4.861851068,1.7522346404
C,0,-0.0744159577,1.3183155576,0.2800435857
C,0,1.1547911887,2.4626366688,-1.1638600744
C,0,-0.0300091323,3.2271914958,-0.9417318491
C,0,-0.2019084371,4.4365534326,-1.6547763796
C,0,0.7804161576,4.8320587575,-2.5310964141
C,0,1.9439161382,4.0521029964,-2.7327652552
C,0,2.1497108063,2.8659763892,-2.0641454434
C,0,-1.1474647623,0.461301715,-2.2919365585
C,0,-1.896925144,1.2074742854,-3.2134528046
C,0,-1.4769371077,1.3132005349,-4.5301118669
C,0,-0.3004152283,0.6950486973,-4.9489899861
C,0,0.4453367611,-0.0562042951,-4.0484260341
C,0,0.0219201321,-0.1788139504,-2.7333776395
C,0,-5.0126664872,-0.769674421,0.2900193333
C,0,-6.0678630028,-0.3000469327,1.0606742101
C,0,-7.3658332613,-0.5174864334,0.6152495923
C,0,-7.6154433052,-1.1881930491,-0.5824486619
C,0,-6.5260007896,-1.6470591335,-1.3359183865
C,0,-5.2252041011,-1.4463194548,-0.9102771696
C,0,-9.0144177758,-1.4203910062,-1.0613766015
H,0,-1.1188572917,-0.7139292682,-0.5152591556
H,0,-4.1509408654,1.9406577922,0.2617122497
H,0,-3.2926275273,2.358652241,-1.2174014945
H,0,-2.1588419697,2.3540512816,1.6105190861
H,0,-2.822097046,4.6512608785,-0.3291680384
H,0,-2.5973588176,4.4198502098,2.7265340859
H,0,-3.1430764419,5.8894883553,1.734440626
H,0,-0.191056114,0.6706040959,1.1390770331
H,0,-1.0815422524,5.0519502133,-1.5128014937
H,0,0.6665056181,5.7601137132,-3.0816997593
H,0,2.6937125715,4.4012639114,-3.4360744901
H,0,3.031411503,2.2500444359,-2.2148497628
H,0,-2.8284880104,1.6792037515,-2.9284212083
H,0,-2.0730627085,1.8816778318,-5.236546691
H,0,0.0257359275,0.7910309875,-5.9797331698
H,0,1.3559789884,-0.5526048122,-4.3667059087
H,0,0.5984548872,-0.7831730426,-2.0398478447
H,0,-5.8780778016,0.2193733239,1.9931595142
H,0,-8.1981135304,-0.1590228418,1.2129047806
H,0,-6.7038256336,-2.1739170088,-2.2686204964
H,0,-4.3904503295,-1.8108694558,-1.5000151446
H,0,-9.1746430123,-0.9495831391,-2.036881899
H,0,-9.7492680813,-1.017124767,-0.3618622175
H,0,-9.2093890325,-2.4904158179,-1.1860379622

S,0,4.1007598171,-0.8484082776,-1.1278863665
O,0,2.8248677656,-0.7391486082,-0.3490540617
O,0,4.3092532579,0.3294320618,-1.9942147959
O,0,4.2335803878,-2.1463844103,-1.8055789561
C,0,5.3994667699,-0.7909221502,0.1063275177
C,0,5.7147223016,0.4207254146,0.7166218843
C,0,6.0682271636,-1.9503939334,0.4713911928
C,0,6.7025783919,0.4630885367,1.6888344766
H,0,5.1965235401,1.3282246732,0.4237110194
C,0,7.0584333668,-1.895414083,1.4487240007
H,0,5.8198401815,-2.887340171,-0.0152852318
C,0,7.3921915632,-0.6932752933,2.0715553106
H,0,6.9479012774,1.4119499131,2.1588706491
H,0,7.582400817,-2.8054654737,1.7277336402
C,0,8.4677236405,-0.6284028393,3.1152596459
H,0,8.0965172204,-0.172205548,4.038605473
H,0,8.8478362423,-1.6236529343,3.3566883838
H,0,9.3110561957,-0.019495622,2.7718182918
H,0,1.8289433755,0.5907602872,-0.3707914225

INT2-C
SCF Done: E(RPBE1PBE) = -2556.86298589 a.u.
Zero-point correction = 0.588978 Hartree/Particle
Sum of electronic and thermal Free Energies = -2556.351937 a.u.

S,0,2.9514801032,1.9058897642,-1.760068934
N,0,-0.0823443412,-1.1916497511,0.4959269435
N,0,2.9608889193,0.4456390642,-0.9452516685
O,0,4.2890377737,2.4536205502,-1.6004519384
O,0,2.4096914843,1.6243896884,-3.0796349389
C,0,1.8399830965,-0.3929280625,1.506179284
C,0,1.784048716,-0.4156085203,-1.0494982358
C,0,3.8344031563,0.2502585226,0.197088031
C,0,3.1107246921,0.3749248832,1.5711402896
C,0,4.0499860145,-0.0201945005,2.6701997534
C,0,4.5233253545,0.8250937883,3.579953852
C,0,0.9959624784,-0.2738797986,0.2889046701
C,0,0.01915783,-1.7362257685,1.695493518
C,0,1.2156954427,-1.2651215906,2.3681470316
C,0,1.5112171062,-1.7424024543,3.6831620028
C,0,0.6491290396,-2.6230513815,4.2578602126
C,0,-0.5293796788,-3.0698942597,3.5735188222
C,0,-0.8650503592,-2.6497306755,2.319316365
C,0,2.1451674852,-1.8266781214,-1.4648644154
C,0,2.6511837036,-2.7832335117,-0.5845839174
C,0,2.9949819796,-4.0519516017,-1.0401514413
C,0,2.836095207,-4.3807414223,-2.3814836674
C,0,2.327247811,-3.4352423315,-3.2654592904
C,0,1.9837577779,-2.1688091509,-2.8088336856
C,0,1.8053522339,2.990605166,-0.9412760881
C,0,0.4768656745,3.0189845042,-1.3566321041
C,0,-0.4203741928,3.8434870924,-0.69054121
C,0,-0.0101025559,4.6440718185,0.3781510869
C,0,1.3349986285,4.6082023164,0.7611839721
C,0,2.2459215827,3.7882430374,0.112366476
C,0,-0.9867517165,5.5163990924,1.1047701243

H,0,1.1494048312,-0.0070708483,-1.8373632621
H,0,4.6474140978,0.9731044058,0.1485565877
H,0,4.2792204069,-0.7460161262,0.1148842291
H,0,2.839082739,1.4323510658,1.6948073479
H,0,4.3790265073,-1.0588010712,2.6713799816
H,0,4.2192800288,1.8691481332,3.6006227506
H,0,5.2310151192,0.5000726374,4.3366508806
H,0,0.585050306,0.7486543631,0.2747879745
H,0,2.3954456241,-1.3981666968,4.2049090889
H,0,0.8418651242,-3.0000269363,5.2562572137
H,0,-1.1808354566,-3.7725882516,4.0843521217
H,0,-1.7601407543,-2.9784449748,1.7990139117
H,0,2.7739664572,-2.5571057139,0.4692618923
H,0,3.3835135285,-4.7857648379,-0.341054709
H,0,3.1015551346,-5.372123481,-2.7349013338
H,0,2.1908556823,-3.6842049963,-4.3130508551
H,0,1.582487057,-1.4370526219,-3.5036712733
H,0,0.1521903957,2.4208370309,-2.2008541398
H,0,-1.4558684489,3.8723620275,-1.01588634
H,0,1.6759748311,5.2433783657,1.5734686444
H,0,3.2908352932,3.7865061715,0.4029876407
H,0,-1.3577092098,5.0097384949,2.00335123
H,0,-1.8506589012,5.7539734882,0.4800842877
H,0,-0.5205352622,6.4508116447,1.4275790216
S,0,-3.4375548498,-1.9884681061,-0.944539247
O,0,-2.1217975271,-1.3179625277,-1.2147795098
O,0,-3.348413694,-2.9000186146,0.2136252443
O,0,-4.0211333972,-2.5733074648,-2.1578234724
C,0,-4.53660304,-0.6611515163,-0.4495424136
C,0,-4.6391269019,-0.3059173525,0.8899699013
C,0,-5.250958836,0.0399307649,-1.4154946362
C,0,-5.4593362938,0.7539591831,1.2599540689
H,0,-4.0911844946,-0.8659150139,1.6404618967
C,0,-6.0650388803,1.0984948237,-1.0340141807
H,0,-5.1754497107,-0.2491153943,-2.4583991326
C,0,-6.1848854942,1.4725875845,0.3074470618
H,0,-5.5414465598,1.0240480193,2.3093160751
H,0,-6.6234461442,1.6411178489,-1.7921721808
C,0,-7.0971304142,2.5916712564,0.7136581427
H,0,-6.8194455749,2.9990050959,1.6889620321
H,0,-7.0817524278,3.4041783542,-0.0180506505
H,0,-8.1330504878,2.2403975983,0.7859530012
H,0,-0.9011528322,-1.2926434352,-0.1572593218

TS3-C
SCF Done: E(RPBE1PBE) = -2556.85358527 a.u.
Zero-point correction = 0.584355 Hartree/Particle
Sum of electronic and thermal Free Energies = -2556.344414 a.u.
Imaginary Freq. = -664.4832 cm^{-1}

S,0,2.0315577129,-0.8513411479,1.1748397341
N,0,-2.6725975747,0.7104179155,1.4070400144
N,0,0.8383868742,-0.0502131928,0.3266933185
O,0,2.1737501956,-0.1487786367,2.4427518035
O,0,1.7983320475,-2.2914296055,1.1806316773
C,0,-1.8386639224,-0.8111728002,-0.0791488496

C,0,-0.1449757695,0.7738852902,1.0333351257
C,0,0.4507792781,-0.6223231847,-0.9509428854
C,0,-0.7875309895,-1.5433065065,-0.8473350243
C,0,-1.2019469847,-1.9901763568,-2.2176004564
C,0,-1.0734806555,-3.2359883311,-2.6625238038
C,0,-1.487137601,0.0277786418,1.0281255679
C,0,-3.7133106135,0.1705974806,0.7489901727
C,0,-3.2345644777,-0.7663856299,-0.2202036775
C,0,-4.1635130434,-1.450315732,-1.044219814
C,0,-5.4986711676,-1.1973426419,-0.8728539155
C,0,-5.9530794525,-0.2800634623,0.111798691
C,0,-5.0888497851,0.4026539932,0.9310232124
C,0,-0.2145819006,2.1640554009,0.4283298476
C,0,-0.9810038416,2.4507588097,-0.7011621366
C,0,-0.9756532521,3.7280197272,-1.2522089016
C,0,-0.2100654179,4.7356226261,-0.6740708696
C,0,0.5461909648,4.4612602765,0.4608086472
C,0,0.5422898021,3.1830547267,1.0085551079
C,0,3.4656177371,-0.5603178555,0.1628480816
C,0,4.2413775429,-1.6399909548,-0.2344957296
C,0,5.3929652686,-1.4051682489,-0.9782022251
C,0,5.7745549367,-0.1102503503,-1.3281761793
C,0,4.9702233572,0.9588975552,-0.9120837138
C,0,3.8212258772,0.7448089178,-0.1697984012
C,0,7.0104622248,0.1430042081,-2.1362779475
H,0,0.210581705,0.8754777505,2.0601235852
H,0,1.2891982508,-1.189077566,-1.3609476733
H,0,0.2481149646,0.1974675934,-1.6474880026
H,0,-0.4896624486,-2.4200233233,-0.2579433036
H,0,-1.5854354584,-1.2156741889,-2.8815001535
H,0,-0.6922577599,-4.0315084808,-2.0263525573
H,0,-1.3492241189,-3.5024223431,-3.6786899983
H,0,-1.3938048191,-0.7985987573,1.9756063812
H,0,-3.8210097624,-2.1642963802,-1.7836609212
H,0,-6.2303250672,-1.7098368737,-1.4881448977
H,0,-7.0212555338,-0.1176834279,0.2178142519
H,0,-5.442055481,1.0995257968,1.6830753455
H,0,-1.5955128374,1.6783096392,-1.1561310251
H,0,-1.5770329945,3.9376246027,-2.1313391184
H,0,-0.2107209365,5.7335661023,-1.1012575449
H,0,1.1362625111,5.2450582651,0.925719136
H,0,1.1270322457,2.9756814876,1.900449647
H,0,3.947665526,-2.6486847287,0.0330448638
H,0,6.0031533381,-2.2471268189,-1.2907656124
H,0,5.2507513958,1.9741669807,-1.1773833168
H,0,3.2033278545,1.5813942897,0.1398928719
H,0,6.7599919988,0.6064464343,-3.0964186751
H,0,7.5523657751,-0.7832429132,-2.3382283084
H,0,7.6861600577,0.8283622141,-1.6148329773
H,0,-2.7614657625,1.0775028467,2.354214312
S,0,-1.8887992244,-0.9483591155,4.4418275333
O,0,-1.3817369228,-1.5465959129,3.1357660688
O,0,-2.4599466401,0.3953261932,4.2146918436
O,0,-0.8790540892,-1.0341301811,5.4942687713
C,0,-3.2391423628,-2.0181723971,4.903815503
C,0,-4.5425008312,-1.5446216178,4.8905898733
C,0,-2.9663976442,-3.3366625528,5.262049997
C,0,-5.5834124366,-2.4022489184,5.2358567874

H,0,-4.7400277655,-0.5136992469,4.6190820844
C,0,-4.0133847939,-4.1790279631,5.5995523056
H,0,-1.9443849451,-3.7007555385,5.277435568
C,0,-5.3390822458,-3.7281664403,5.5907781677
H,0,-6.6039193322,-2.0300570257,5.2286346308
H,0,-3.7998329067,-5.2074600764,5.8776460594
C,0,-6.4591557777,-4.6568116205,5.9535667186
H,0,-6.4933460361,-5.5142529507,5.2732319176
H,0,-6.329007378,-5.0544634324,6.9653317621
H,0,-7.4263700314,-4.1510457757,5.9098625886

TS-allyl-C
SCF Done: E(RPBE1PBE) = -2556.83199672 a.u.
Zero-point correction = 0.587479 Hartree/Particle
Sum of electronic and thermal Free Energies = -2556.318898 a.u.
Imaginary Freq. = -286.9018 cm^{-1}

S,0,3.4417714541,2.2482779577,1.0684209996
N,0,2.9930569018,0.6441480107,1.1656573146
N,0,-0.486799925,-0.7070750718,-0.9019015599
O,0,4.6800802173,2.2647837022,0.3074832339
O,0,3.3874022902,2.7502417757,2.4325188654
C,0,3.0588467928,-0.1840384409,-0.0436412526
C,0,2.010721506,0.1946691552,2.1364492325
C,0,0.6228874351,0.0057954645,1.563059902
C,0,1.6912054413,-0.7840135819,-0.2734647253
C,0,0.5513968809,0.0468204582,-0.4818532813
C,0,-0.1142164962,-2.0215974125,-0.8896245089
C,0,1.2514946143,-2.1164878971,-0.499916831
C,0,1.8493576446,-3.3908431903,-0.394652865
C,0,1.0905311795,-4.4959773425,-0.7032925664
C,0,-0.2587473522,-4.3728412875,-1.1056894628
C,0,-0.884391606,-3.1484941295,-1.1971696629
C,0,-0.1349745167,-1.1303933372,2.0452064534
C,0,-1.4736503924,-1.150491857,2.0533837211
C,0,4.2074470792,-1.1746549292,-0.0411887731
C,0,4.6348774182,-1.6985493135,-1.2616079482
C,0,5.6803588582,-2.6123457484,-1.3094032008
C,0,6.3144441434,-3.0073021357,-0.134853142
C,0,5.903698401,-2.4733121193,1.0816112321
C,0,4.8576193777,-1.5562274557,1.1285466573
C,0,2.2171199641,3.104063332,0.108680649
C,0,1.0707248367,3.5916747208,0.7347955452
C,0,0.088812767,4.1943688578,-0.0350769214
C,0,0.2308751492,4.3233496775,-1.4224398827
C,0,1.3986178795,3.8442701611,-2.018412968
C,0,2.3955986574,3.2366492999,-1.2643003511
C,0,-0.845863872,4.9705642779,-2.2368665271
H,0,3.227048911,0.4855118982,-0.8977194673
H,0,1.974666549,0.9148034741,2.9573455168
H,0,2.3580950781,-0.7573826513,2.5506532921
H,0,0.0466430737,0.9269171777,1.4906809754
H,0,0.551798136,1.1173170169,-0.6348315387
H,0,2.8815975448,-3.4994119545,-0.0859966233
H,0,1.5327326905,-5.4845017568,-0.6383074191
H,0,-0.818495733,-5.2719064037,-1.3444559109

```
H,0,-1.9219280325,-3.0360158526,-1.4978010611
H,0,0.4265961508,-1.9940548185,2.3936376882
H,0,-2.0568736143,-0.3005570777,1.7099642341
H,0,-2.0205562826,-2.014183608,2.4185321839
H,0,4.1462531316,-1.3892879427,-2.1818601446
H,0,6.0056747277,-3.0096976543,-2.2656937045
H,0,7.1339762367,-3.7182736366,-0.1698124316
H,0,6.4048734752,-2.7628551451,2.0000375476
H,0,4.5630009267,-1.1245721723,2.0792412048
H,0,0.9559415699,3.5139060376,1.8106322158
H,0,-0.8041717252,4.5801714814,0.4481646075
H,0,1.5342345184,3.9509770313,-3.0903594198
H,0,3.3062080232,2.8840254955,-1.735777782
H,0,-1.7948897949,4.4373508397,-2.1208107092
H,0,-0.5896235308,4.9840206631,-3.2980099519
H,0,-1.0142896717,6.0024234216,-1.9116503505
H,0,-1.4391302898,-0.3353096083,-1.1322126435
S,0,-4.1081255707,-0.3911935842,-1.7873565997
O,0,-2.8642615244,0.3894099791,-1.4779663814
O,0,-3.8064914858,-1.8154538849,-2.0298354799
O,0,-4.9275183635,0.2598160023,-2.8181323529
C,0,-5.0695681933,-0.338972775,-0.2748145354
C,0,-5.7744424434,0.8164237854,0.0537312575
C,0,-5.0833964502,-1.4316373984,0.5808450938
C,0,-6.4824468687,0.8729064538,1.2453509692
H,0,-5.77350594,1.6634928533,-0.6244343943
C,0,-5.8001575948,-1.3643230419,1.7723501598
H,0,-4.5427369912,-2.3316053055,0.3085849588
C,0,-6.5080948572,-0.215336364,2.1248721603
H,0,-7.0328698439,1.7756046729,1.4972205531
H,0,-5.8125242881,-2.2237764738,2.4371902022
C,0,-7.2900740415,-0.1459570522,3.4028454813
H,0,-7.0787985531,-1.0041979614,4.0450513815
H,0,-7.0570119544,0.7654696561,3.9621300436
H,0,-8.3674245891,-0.1341958461,3.204048535
---------------------------------------------------------
```

References

1. Gaussian 09, Revision A.01, Frisch, M. J.; Trucks, G. W.; Schlegel, H. B.; Scuseria, G. E.; Robb, M. A.; Cheeseman, J. R.; Scalmani, G.; Barone, V.; Mennucci, B.; Petersson, G. A.; Nakatsuji, H.; Caricato, M.; Li, X.; Hratchian, H. P.; Izmaylov, A. F.; Bloino, J.; Zheng, G.; Sonnenberg, J. L.; Hada, M.; Ehara, M.; Toyota, K.; Fukuda, R.; Hasegawa, J.; Ishida, M.; Nakajima, T.; Honda, Y.; Kitao, O.; Nakai, H.; Vreven, T.; Montgomery, Jr., J. A.; Peralta, J. E.; Ogliaro, F.; Bearpark, M.; Heyd, J. J.; Brothers, E.; Kudin, K. N.; Staroverov, V. N.; Kobayashi, R.; Normand, J.; Raghavachari, K.; Rendell, A.; Burant, J. C.; Iyengar, S. S.; Tomasi, J.; Cossi, M.; Rega, N.; Millam, N. J.; Klene, M.; Knox, J. E.; Cross, J. B.; Bakken, V.; Adamo, C.; Jaramillo, J.; Gomperts, R.; Stratmann, R. E.; Yazyev, O.; Austin, A. J.; Cammi, R.; Pomelli, C.; Ochterski, J. W.; Martin, R. L.; Morokuma, K.; Zakrzewski, V. G.; Voth, G. A.; Salvador, P.; Dannenberg, J. J.; Dapprich, S.; Daniels, A. D.; Farkas, Ö.; Foresman, J. B.; Ortiz, J. V.; Cioslowski, J.; Fox, D. J. Gaussian, Inc., Wallingford CT, 2009.
2. (a) Perdew, J. P.; Burke, K.; Ernzerhof, M. *Phys. Rev. Lett.* **1996**, *77*, 3865. (b) Perdew, J. P.; Burke, K.; Ernzerhof, M. *Phys. Rev. Lett.* **1997**, *78*, 1396.
3. Marenich, A. V.; Cramer, C. J.; Truhlar, D. G. *J. Phys. Chem. B* **2009**, *113*, 6378.
4. Zheng, C.; Wu, Q.-F.; You, S.-L. *J. Org. Chem.* **2013**, *78*, 4357.
5. Legault, C. Y. CYLView, 1.0b; Université de Sherbrooke, Montreal, Québec, Canada, 2009; http://www.cylview.org.
